# Chicken rRNA Gene Cluster Structure

**DOI:** 10.1371/journal.pone.0157464

**Published:** 2016-06-14

**Authors:** Alexander G. Dyomin, Elena I. Koshel, Artem M. Kiselev, Alsu F. Saifitdinova, Svetlana A. Galkina, Tatsuo Fukagawa, Anna A. Kostareva, Elena R. Gaginskaya

**Affiliations:** 1 Saint Petersburg State University, Saint Petersburg, 199034, Russia; 2 Almazov Federal Medical Research Centre, Saint Petersburg, 197341, Russia; 3 ITMO University, Saint Petersburg, 197101, Russia; 4 Osaka University, Suita, Osaka, 565–0871, Japan; International Centre for Genetic Engineering and Biotechnology, ITALY

## Abstract

Ribosomal RNA (rRNA) genes, whose activity results in nucleolus formation, constitute an extremely important part of genome. Despite the extensive exploration into avian genomes, no complete description of avian rRNA gene primary structure has been offered so far. We publish a complete chicken rRNA gene cluster sequence here, including *5’ETS* (1836 bp), *18S rRNA* gene (1823 bp), *ITS1* (2530 bp), *5*.*8S rRNA* gene (157 bp), *ITS2* (733 bp), *28S rRNA* gene (4441 bp) and *3’ETS* (343 bp). The rRNA gene cluster sequence of 11863 bp was assembled from raw reads and deposited to GenBank under KT445934 accession number. The assembly was validated through *in situ* fluorescent hybridization analysis on chicken metaphase chromosomes using computed and synthesized specific probes, as well as through the reference assembly against *de novo* assembled rRNA gene cluster sequence using sequenced fragments of BAC-clone containing chicken NOR (nucleolus organizer region). The results have confirmed the chicken rRNA gene cluster validity.

## Background

**Fig 1:**
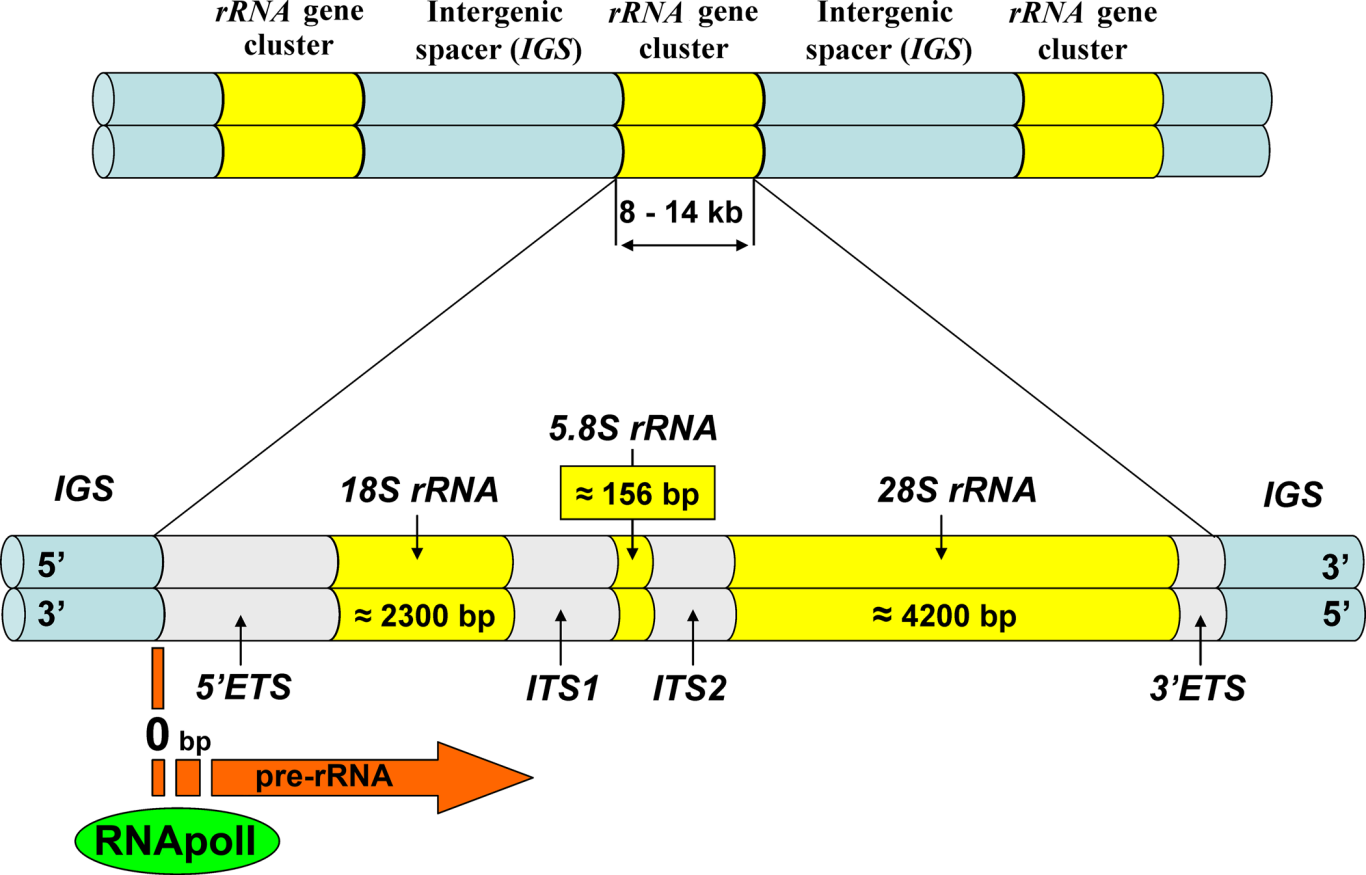
Ribosomal DNA structure (after Singer and Berg, 1991).

Ribosomal gene arrays constitute one of the most important genome components [1]. They form nucleolus organizing regions (NOR) in chromosomes, and the functional status of NOR is an indicator of the physiological status of cells, tissues and the entire organism at various ontogenetic stages [1–6]. In a commonly accepted approach that we follow here, the term “ribosomal gene array” refers to the sequence composed of repeated rRNA gene clusters separated by intergenic spacers (*IGS*) [7]. All animal rRNA gene clusters are known to have fundamentally similar structures (Fig 1). Each cluster comprises sequences of rRNA encoding conservative genes (*18S rRNA*, *5*.*8S rRNA* and *28S rRNA*) separated by internal transcribed spacers (*ITS1* and *ITS2*), and external transcribed spacers (*5’ETS* and *3’ETS*) situated downstream of *18S rRNA* gene and upstream of *28S rRNA* gene respectively. All these elements transcribe into a single RNA precursor, pre-rRNA. *18S rRNA*, *5*.*8S rRNA* and *28S rRNA* genes are quite conserved between organisms. Both internal and external transcribed spacers feature high structural variability, which accounts for ribosome cluster length variation within a wide range from 8 to 14 thousand base pairs (bp), promoter and terminator regions for RNA polymerase I (RNApolI) being located in the *IGS* [7].

In general, rRNA gene cluster structure is still underinvestigated [1]. Despite recent vertebrate genome-wide extensive studies [8], decoding of NOR structure and rRNA gene clusters remains a complicated task, primarily due to the high repetitivity and extensive length of the clusters as well as a faster spacer sequence evolution [1]. So far GenBank [9] has contained annotated complete rRNA gene cluster sequences only for a limited number of vertebrate species, in particular *Homo sapiens* (GenBank accession number: U13369), *Mus musculus* (GenBank accession number: BK000964) and *Nothobranchius furzeri* (GenBank accession number: EU780557). Yet, no description of the complete rRNA gene cluster sequence has been offered so far for such a major taxon as Aves.

Chicken rRNA gene cluster deserves special research focus among bird class representatives. Chicken plays an important role in agriculture and it has been widely used in an extensive range of biomedical research, including such areas as developmental biology, genetics, cell biology, histology, virology and so on [10, 11]. Chicken genome was the first to have been sequenced in avian and among the first in vertebrata genomes [12]. *Gallus gallus 4*.*0* assembly has been used as a reference genome for assembling sequenced genomes of other birds [13]. Chicken NOR location on chromosome 16 (GGA16) [14–17] has been recently confirmed by fluorescent *in situ* hybridization (FISH) using plasmid *ETS* [18] and NOR containing WAG137G04 BAC-clone [19] as probes. Noteworthy, only 5% of GGA16 chromosome sequence has been decoded according to the published assembly of *Gallus gallus 4*.*0* genome [12], and such decoding does not include the NOR region [19].

GenBank contains annotated sequences for individual fragments of chicken *18S rRNA* and *28S rRNA* genes. There are also two incompatible sequence variants, both annotated in GenBank as *ITS1-5*.*8S-ITS2* (Accession numbers: DQ018752 –DQ018755; FJ008990).

In the course of “ChIP-sequencing with CENP-A from chicken cells containing neocentromeres on Z chromosome” project [20], we obtained multiple raw chicken sequences. Based on these sequences as well as sequences of unannotated Whole Genome Shotgun (WGS) contigs from *Gallus gallus 4*.*0* genome assembly (NCBI WGS accession number: AADN00000000), we assembled and described the complete structure of chicken rRNA gene cluster. The assembled sequence relation to GGA16 NOR was confirmed by serial FISH to chicken mitotic chromosomes. For validation of rRNA gene cluster assembly a NOR-containing BAC clone [19], was sequenced and re-assembled on the basis of the *de novo* assembled chicken rRNA gene cluster.

## Material and Methods

### SRA data and BAC clone sample

To assemble a complete chicken rRNA gene cluster *de novo* we used a sequence library [20] which had been earlier generated of raw reads (NCBI SRA accession number: DRX001863) in the course of “ChIP-sequencing with CENP-A from chicken cells containing neocentromeres on Z chromosome” project (NCBI BioProject accession number: PRJDB2279). For assembly verification and adjustment we used 19 unannotated sequences of the WGS contigs from the *Gallus gallus 4*.*0* genome assembly (NCBI WGS accession number: AADN00000000.3) [12] and 11 sequences from Nucleotide database [21] annotated by the authors as chicken ribosomal cluster fragments (Table 1). WAG137G04 BAC clone containing chicken NOR was used as a probe to NOR [19].

**Table 1:** WGS contigs and annotated sequences used to verify the chicken ribosomal cluster assembly.

Cluster element	NCBI accession number	Reference
*5’ETS* (complete)	NW_003775878, AADN03015064, AADN03001778, AADN03014081, AADN03001785, AADN03001786, AADN03001788	[12]
	DQ112354	[11]
*18S rRNA* (complete)	AADN03000430, AADN03001677, AADN03001785, AADN03001786, AADN03001788, AADN03026634	[12]
	FM165414	Unpublished
	DQ018752, DQ018754	Unpublished
	HQ873432	Unpublished
	AF173612	[22]
*ITS1* (complete)	AADN03026634, AADN03000430, AADN03001782, AADN03001670, AADN03001783	[12]
	DQ018752, DQ018754	Unpublished
*5*.*8S rRNA* (complete)	AADN03001670, AADN03001783	[12]
	DQ018754	Unpublished
*ITS2* (complete)	AADN03001783, AADN03001784	[12]
*28S rRNA* (complete)	AADN03001784, AADN03001774, AADN03001775, AADN03001776, AADN03022685, AADN03019346	[12]
	EF552813	[23]
	FM165415	Unpublished
	JN639848	Unpublished
	DQ018756, DQ018757	Unpublished
*3’ETS*	Data is absente	

### BAC clone sequencing

WAG137G04 BAC clone high-throughput sequencing was performed using Illumina MiSeq sequencing machine and MiSeq Reagent Kit v3 2x300. DNA quality was analyzed using Nanodrop 1000 spectrophotometer (Thermo, USA). DNA was quantified with Quantifluor-ST fluorometer (Promega, USA) and dsDNA kit (Promega, USA). DNA fragment library was prepared from 1 ng of BAC clone DNA using NexteraXT kit (Illumina, USA) according to manufacturer’s guideline. Library quality was estimated using Agilent Bioanalyzer 2100 instrument (Agilent, USA) and RT-PCR according to Illumina Sequencing Library qPCR Quantification Guide.

### Cluster assembling and annotating

*De novo* assembling of chicken rRNA gene cluster sequence from SRA sequence library DRX00186 and searching for WGS contigs homologous to the rRNA gene cluster elements was performed using BLAST [24] and Unipro UGENE v. 1.16.1 software package [25].

Chicken rRNA gene cluster reference assembling was performed on the basis of BAC clone raw read library using Geneious 9.0.5 software package [26]. The *de novo* assembled sequence was used as a reference. For sequence alignment and nucleotide structure determination Mega 6.06 [27] was applied. Repeat searching and typing were performed in Repeatmasker 4.0.5 [28]. Nucleotide sequence secondary structure was computed in Mfold [29].

To define the boundaries between spacer (*5’ETS*, *ITS1*, *ITS2*, *3’ETS*) sequences and *rRNA* gene (*18S*, *5*.*8S*, *28S*) sequences we used annotated fragments of ribosomal clusters of *H*. *sapiens* (GenBank accession number: U13369), *Rattus norvegicus* (GenBank accession number: NR_046239), *M*. *musculus* (GenBank accession number: NR_046233), *Xenopus laevis* (GenBank accession number: X02995) and *Crocodylus porosus* (GenBank accession number: EU727191).

### Fluorescent *in situ* hybridization

To verify the relation of *de novo* assembled sequence to the NOR on GGA16 a serial FISH was applied to chicken mitotic chromosomes using the assembled sequence fragments and WAG137G04 BAC clone DNA as probes. Metaphase spreads were obtained from primary chicken embryo cell cultures according to standard procedures. Briefly, a chicken embryo of 4 day incubation was aseptically removed from the egg, washed in PBS and fragmented. Embryo fragmrnts were mechanically macerated and cell suspension has been placed into a tissue culture flask with Dulbecco's modified Eagle's medium (DMEM, Paneco) containing 10% fetal bovine serum (PAA Laboratories), 1% Streptomycin and 1% L-Glutamine. Settled cells were cultured overnight at 37°C in humidified 5% CO_2_ atmosphere and arrested with 0.05 μg/ml colcemid (CaryoMax, Invitrogen), followed by hypotonic treatment and fixation. Fertile eggs were provided by the Federal State Unitary research farm “Gene Pool”, Pushkin, Leningrad region, Russia (http://genofund.narod.ru/index.htm). Experimental procedures involving chicken embryos were conducted in accordance with International Guiding Principles for Biomedical Research Involving Animals established by Council for International Organizations of Medical Sciences (CIOMS) and approved by Saint-Petersburg State University Ethics Committee (statement # 131-03-2).

To prepare FISH probes, PCR primers were designed from the *de novo* assembled rRNA gene cluster using Unipro UGENE 1.16.1 software package; their identity to the related regions of the assembled rRNA gene cluster was validated by standard sequencing. WAG137G04 BAC clone probe was produced by standard DOP-PCR amplification using 6MW primers [30].

PCR amplification of the selected sequence areas was carried out in 20 μl of reaction mix as follows: Taq-pol 5U/μl (Sileks, Russia) – 0,5 μl; 10X Taq Buffer (Sileks, Russia) – 2 μl; MgCl2 25 mM (Sileks, Russia) – 2 μl; 10mM dNTP (2.5 mM each) (Sileks, Russia) – 1.6 μl; 10μM primer (10 pmol/μl) – 1 μl each; DNA – 0,5 μl; H_2_O – 11.4 μl. PCR protocol: 94°С–5’; (94°С–20”, 60°C–15”, 72°C–20”)35 сycles; 72°С–5’; 4°C–hold.

Fluorescent probes were generated by labeling PCR products with modified biotin-16-dUTP nucleotide (Sileks, Russia) during amplification procedure. Biotin was detected with streptavidin-Alexa488 (Invitrogen, USA). FISH was carried out according to a published protocol [31]. The preps were additionally stained with 4’,6-diamidino-2-phenylindole (DAPI) in concentration of 1μg/ml. For establishing whether the fluorescent probe hybridization had taken place at NOR on GGA16, re-FISH with WAG137G04 BAC clone probe was carried out on the same preparations.

FISH results were analyzed using DM4000В (Leica Microsystems CMS, GmbH) epifluorescent microscope. The images were processed using LAS AF software (Leica Microsystems CMS, GmbH) and superposed in Adobe Photoshop CS5.1 (Adobe Systems, Inc, USA).

## Results and Discussion

### *De novo* rRNA gene cluster assembly

We analyzed all GenBank sequences annotated as chicken rRNA gene cluster elements. Eight of them were completely rejected because of their short lengths or high homology to ribosomal DNA of other organisms (Table 2). Veracity of nine others and related annotations were also under doubt because in GenBank were represented only in unique copies. Among the annotated chicken rDNA sequences, 18S rDNA fragment was only deposited to GenBank in several identical copies. We found neither *ITS2/3’ETS* nor the major part of *28S rRNA* gene annotated sequences related to chicken in Genbank.

**Table 2:** The rejected sequences of chicken rDNA.

NCBI accession number	Annotation	Reference
FJ008990	*18S rRNA* gene, *ITS1*, *5*.*8S rRNA* gene, *ITS2*, *28S rRNA* gene	Unpublished
D38360	*18S rRNA* gene	[32]
K01379	*18S rRNA* gene	[33]
AH001604	*28S rRNA* gene	[34]
M59792	*28S rRNA* gene	[34]
M59414	*28S rRNA* gene	[34]
DQ018755	*5*.*8S rRNA* gene, *ITS2*, *28S rRNA* gene	Unpublished
DQ018753	*ITS1*, *5*.*8S rRNA* gene, *ITS2*	Unpublished

We selected the sequences reliably annotated as chicken rRNA gene cluster fragments to identify homologous WGS contigs in GenBank. *Gallus gallus* WGS project (accession number AADN00000000.3) in total contains 19 contigs. These contigs appeared to belong to chicken rRNA gene cluster. However, in the proposed rRNA gene cluster sequence, contig overlappings were absent at three sites and no contigs containing either *28S rRNA* gene 3’end or *3’ETS* sequence were found. The overlapping contig length was 9230 bp. The total length of overlapping annotated sequences and contigs from the transcription initial site was 9931 bp (S1 Table).

The maximum overlapped area was found in a *18S rRNA* gene sequence of 388 bp assembled from ten homologous sequences (six contigs and four annotated sequences). This 388 bp sequence served as a primary reference for our rRNA gene cluster *de novo* assembly using raw reads obtained earlier (NCBI SRA accession number DRX001863) [20]. 388 bp DNA fragment homologous reads were identified in BLAST and aligned in Unipro UGENE v. 1.16.1. The hanging ends of the consensus sequence were tiled in Geneious 9.0.5. As a result, we assembled a sequence of 12053 bp, a 11994 bp sequence being upstream of a pre-rRNA transcription initiator site representing a uninterrupted contig.

In total, we used 941443 forward and reverse reads. Sequence mean coverage with reads was 1767 per site.

### FISH co-localization assembled sequence fragments with NOR

**Fig 2:**
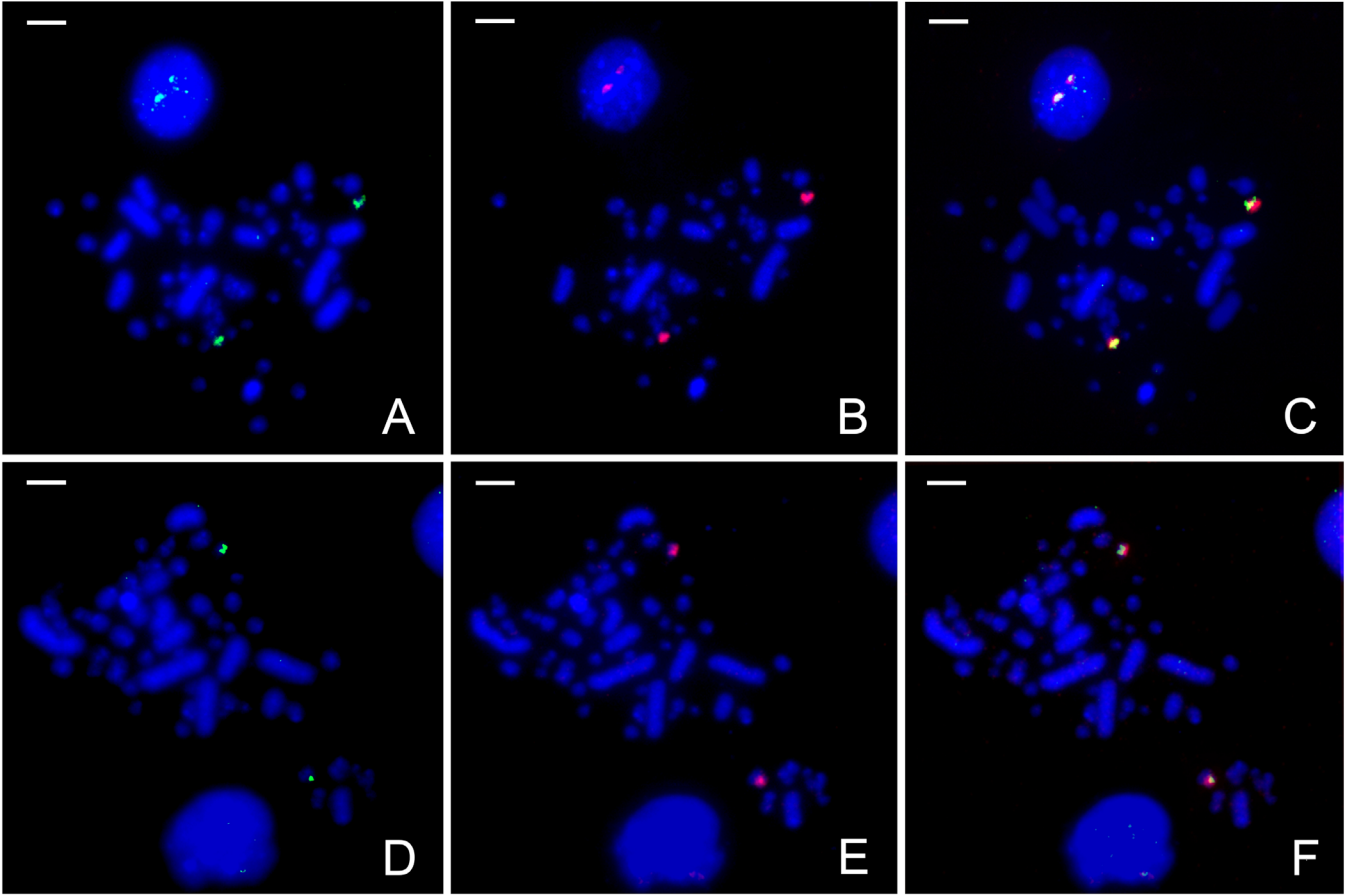
Localization of assembled rRNA gene cluster fragments on chicken mitotic chromosomes. FISH with (A) *5’ETS–18S* rDNA fragment probe, (D) *ITS1–5*.*8S* rDNA fragment probe, (B, E) WAG137G04 BAC probe, which contains NOR, marker of GGA16, (C, F) merge. A, D–green fluorescence; B, E–red fluorescence; C, F–merge. Chromosomes are counterstained with DAPI (blue). Bar – 5 μm.

To verify the assembled sequence relation to the NOR on GGA16, we applied serial re-FISH to chicken mitotic chromosomes. The probes to areas from 1829 to 2024 bp and from 6189 to 6358 bp, presumably associated with *5’ETS–18S* rDNA and *ITS1–5*.*8S* rDNA boundaries respectively within the assembled sequence, were obtained from genome DNA by primer synthesis (Table 3). Probe were 196 bp and 170 bp in length respectively. The primers were computed based on the assembled rRNA gene cluster. Probe adequacy was validated by standard sequencing. The NOR probe was produced by DOP-PCR amplification of WAG137G04 BAC-clone known to contain a chicken GGA16 fragment comprising NOR [19]. On chicken mitotic plates, all probes were hybridized to similar positions on two homologous microchromosomes (Fig 2). Our findings reliably confirm that our assembly strongly matches chicken NOR.

**Table 3:** The list of primers for amplification of target regions of the assembled ribosomal cluster sequence.

Primer designed	Fragment length
F1829 5’-AGAGAGGGAAGGAGCGAGAG-3’	196 bp
R2024 5’-GAGCGACCAAAGGAACCATA-3’	
F6189 5’-CAAGGCGAGAGAGAACGAG-3’	170 bp
R6358 5’-AGTGCGTTCGAAGTGTCGAT-3’	

### BAC clone sequencing and reference assembly of rRNA gene cluster

WAG137G04 BAC clone -containing chicken NOR was sequenced using Illumina MiSeq to check whether the assembled sequence was really a chicken rRNA gene cluster. In total, 941780 forward and reverse reads were obtained. The mean read length was 239 bp. The BAC clone raw reads library was submitted to SRA database (GenBank accession number: SRP069763; BioProject accession number PRJNA311164). The *de novo* assembled rRNA gene cluster was used as a reference sequence. The assembling was carried out in Geneious 9.0.5. 91444 reads were found to be homologous to the reference sequence. Mean reference coverage was 1713. A total of 98.3% of the reference sequence overlapped the reads. The *de novo* and re-assembled variants of the rRNA gene cluster sequence demonstrate 99.7% identity in the overlap area (S3 Table). Therefore, our results demonstrate that the assembled *de novo* sequence of 12053 bp represents a chicken rRNA gene cluster.

### Chicken rRNA gene cluster sequence annotation

Chicken rRNA gene cluster sequence was described through comparison with the most completely annotated Tetrapoda rDNA sequences in Genbank: *H*.*sapiens*, *R*.*norvegicus*, *M*.*musculus*, *X*.*laevis* and *C*.*porosus* (S2 Table).

#### 5’ETS

The validity of our chicken rRNA *5’ETS* assembly was confirmed using seven WGS contigs (Table 1). Additionally, we used a chicken sequence (GenBank accession number: DQ112354) containing the entire RNApolI promoter and a pre-rRNA initiation site [11]. These WGS contigs and annotated sequences overlapped 63.9% and 14.8% of the assembly respectively (S1 Table). Within the designated boundaries (S1 Fig) the length of *5’ETS* is 1836 bp (from 1 to 1836 bp) (S2 Table). Within the assembled *5’ETS*, repeating sequences account for 6.4% of its size and are composed of (GCGA)n and (GAGA)n nucleotide combinations and (CGG)n and (GCC)n reverse repeats, the latter being responsible for formation of hairpins related to rRNA processing. The *5’ETS* sequence was also found to feature increased GC pair content – 75.0%. At the same time, CpG content in chicken *5'ETS* (15.7%) is comparable to CpG content in *5'ETS* of human (16.6%) and xenopus (15.5%).

Despite significant variation in *5'ETS* length among these three species (1961, 3656 and 713 bp, respectively) and lack of a clear homology (S2 Table), their CpG content variability does not exceed 1.1%. The CpG dinucleotide content similarity in varying sequences from quite distant animal taxa probably suggests the importance of CpG role in promoter and spacer functions of *5'ETS*. It might also be indicative of the existence of a stabilizer for spacer nucleotide content.

#### *18S rRNA* gene

To confirm the accuracy of our assembly, six WGS contigs and five annotated sequences of fragments of *18S rRNA* gene were used in addition to raw sequences (Table 1). These WGS contigs and annotated sequences overlapped 100% and 99.4% of the assembly respectively (S1 Table). Within the designated boundaries (S2 Fig) the length of complete *18S rRNA* sequences was 1823 bp (from 1837 to 3659 bp starting from the transcription initiation site; S2 Table), which is virtually the same as the length of *X*. *laevis 18S rRNA* (1825 bp). GC pair content is 54.5% and is also comparable to other taxon data: *H*. *sapiens* (56.1%), *X*. *laevis* (53.8%), *C*. *porosus* (49.5%).

#### ITS1

ITS1 sequence deposited to GenBank by Tang et al. (DQ018754, complete sequence; DQ018752, partial sequence) and Chen et al. (FJ008990, complete sequence) were found to be mismatched. We did define chicken ITS1 structure more precisely through tiling assembly of raw sequences and 5 WGS contigs. WGS contigs overlapped 83% of ITS1 assembly (S1 Table). Within the designated boundaries (S3 Fig) the length of chicken 1TS1 is 2530 bp (from 3660 to 6189 bp) (S2 Table). Our entire ITS1 sequences are homologous to the version offered by Tang et al., with the exception of spacer boundaries and 430 bp insertion in position 4898–5328 bp starting from the transcription initiation site. ITS1 nucleotide content analysis resulted in high GC pair content – 82.2% and high CpG dinucleotide content – 20.1%. Repeats account for 13.4% of ITS1 size and are composed of the following nucleotide combinations: (CCGAGG)n, (CCGGT)n, (GC)n, (GGGGGCC)n, (GAG)n и (GGCGCG)n. High GC pair content in ITS1 sequence leads to formation of multiple hairpin structures. In accordance with the models produced for T – 60°C, [K^+^] – 50 mM PCR conditions, complete hairpin dissociation does not take place (S4 Fig). Under such conditions, the average increment of Gibbs free energy (ΔG) in ITS1 sequence in ssDNA form is – 183.23.

#### *5*.*8S rRNA* gene

For assembly validation two WGS contigs and an annotated sequence (GenBank accession number: DQ018754) (Table 1) were used. These WGS contigs and annotated sequences overlapped 100% and 66.2% of the assembly respectively (S1 Table). Upon alignment (S5 Fig) the length of chicken *5*.*8S rRNA* gene was 157 bp (from 6190 to 6346 bp) and similar to the equivalent human gene (S2 Table). The number of GC pars in chicken *5*.*8S rRNA* sequence was also similar to human (57.3% and 57.4% respectively).

#### ITS2

Chicken *ITS2* assembly was validated using two overlapping WGS contigs (Table 1), assembly overlapped being 100% (S1 Table). We excluded from the analysis sequences DQ018753, DQ018755 and FJ008990, which are annotated as containing chicken *ITS2*. The reason was that the related gene flank sequences were found non-homologous to 3’-end of *5*.*8S rRNA* sequence and to *5*’-end of chicken *28S rRNA*. Within the designated boundaries (S6 Fig) the length of chicken *1TS2* was 733 bp (from 6347 to 7079 bp) (S2 Table). Repeats account for 24.3% of spacer size. These repeats are composed of the following nucleotide combinations: (CCGT)n, (GCGCG)n, (CGTT)n, (CCGT)n, (GCG)n. The sequence features increased GC pair content – 82.0% and CpG dinucleotide content – 20.7% which is virtually similar to *ITS1* values (see above). Similarly to *ITS1*, *ITS2* also feature formation of extended hairpins with high melting temperature. According to our results obtained under standard PCR parameters, ΔG = -62.5 (S7 Fig).

#### *28S rRNA* gene

The assembled chicken *28S rRNA* sequence was verified using six WGS contigs and five annotated sequences from GenBank (Table 1). These WGS contigs and annotated sequences overlapped 76.7% and 69.7% of the assembly respectively (S1 Table). Within the designated boundaries (S8 Fig) chicken *28S rRNA* gene length is 4441 bp (from 7080 to 11520 bp) (S2 Table). GC pair content in *28S rRNA* chicken gene is much higher than in *18S* and *5*.*8S rRNA* genes and achieves as much as 68.0%.

#### 3’ETS

Chicken *3’ETS* sequence was assembled from raw reads. The length of the initial assembly was 474 bp. Yet we were unable to define precisely the position of the 3’-end of *3’ETS* due to lack of data on the sequence of Sal1-box, the variable element of RNApolI transcription termination [35].

Based on comparison of *3’ETS* length and nucleotide content in other organisms (345 bp / 82.9% GC in human, 521 bp / 75.62% GC in mouse, 236 bp / 84.32% GC in xenopus), we can assume that in chicken pre-rRNA transcription termination could occur at 11863 bp position. In chicken *3’ETS* sequence, the region between 11864 bp and 11994 bp is a poly-T region (S1 Table). This region has not been reported to exist in *3’ETS* of *H*.*sapiens*, *M*.*musculus*, and *X*.*laevis* (S9 Fig). An analysis of 343 bp (from 11521 to 11863 bp) of the *3’ETS* region has resulted in 79.9% and 18.9% of GC pair and CpG dinucleotide content respectively. (GC)n, (CGTT)n and (CGGC)n repeats constitute about 24.78% of the analyzed sequence. These results indicate a certain similarity between the 5’*ETS* and internal ribosomal cluster spacers. We believe that this phenomenon could be related to the existence of a general evolutionary mechanism supporting this stability of spacer nucleotide content within the ribosomal cluster sequence in birds.

### *ITS1* and *ITS2* features

We have shown chicken *ITS1* sequence to be more extended than that of most animals, with the exception of marsupials [36]. Chicken *ITS2* has also proved to be far more extended than reported earlier (GenBank accession numbers: DQ018753, DQ018755, FJ008990). Our attempts to amplify chicken *ITS1* and *ITS2* using routine PCR with different thermostable polymerases and GC-enhancers as well as to obtain their complete sequences have been unsuccessful. Our findings suggest that the key problem in avian *ITS1* and *ITS2* amplification and sequencing related to high CG pair content and secondary structure formation. These factors impact polymerase effect in PCR process and increase the probability of AT-enriched regions non-specific amplification. This is quite likely to be the main reason for the poor representation of annotated avian ribosomal cluster sequences in the current version of GenBank (Table 4).

**Table 4:** GenBank representation of *ITS1* and *ITS2* sequences.

Taxonomic group	Number of deposited sequences	
	*ITS1*	*ITS2*
Nematoda	7301	7283
Insecta	9616	19614
Mollusca	5729	4374
Chondrichthyes	22	193
Osteichthyes	1432	571
Amphibia	4	51
Reptilia	56	102
**Aves**	**5**	**3**
Mammalia	22	23

Notably, among the eight avian sequences deposited to GenBank, only three actually belong to ribosomal clusters, namely to *ITS1* sequence. At the same time, complete decoding of the ribosomal cluster sequence in different groups of organisms is of a great value for various fields of biology, primarily systematics, phylogeny, and ecology. Our alignment of chicken rRNA gene complete cluster might be useful for comparative research in these fields. In the majority of animals, including birds, the order of alternation of coding and spacer sequences within rRNA gene clusters is conserved. Yet spacer sequences, particularly *ITS1* and *ITS2*, are characterized by high variability. Due to this they are used extensively as DNA barcodes as well as nuclear markers of micro- and macro-evolutionary events [37–40]. However, the features of avian *ITS1* and *ITS2* do not allow treating their sequences as easily accessible and effective DNA barcodes or phylogenetic markers for this class.

## Conclusions

**Fig 3:**
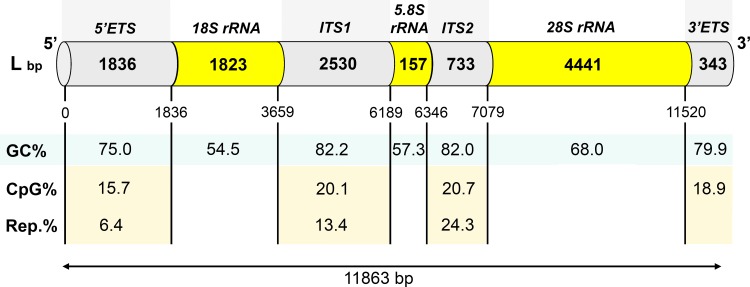
The chicken rRNA gene cluster structure and features.

In this work, we determined, verified, and featured the complete sequence of chicken ribosomal cluster **(**Fig 3**)**. The assembled chicken cluster is 11863 bp in length and contains complete *5’ETS*, *18S rRNA*, *ITSI*, *5*.*8S rRNA*, *ITS2*, *28S rRNA* sequences as well as *3’ETS* sequence 5’-end. This cluster sequence was deposited to GenBank under accession number KT445934. Chicken *18S*, *5*.*8S* and *28S rRNA* gene sequences are typical for orther tetrapods. However both *ITS1* and *ITS2* were found to be more extended in size and higher in GC content. As a result, they have a complicated secondary structure that prevents their PCR amplification and consequently their use as phylogenetic markers. It seems more promising to use a relatively less GC-enriched *5’ETS* sequence for phylogeny purposes. Meanwhile, *ITS1*, *ITS2* and *3’ETS* sequences revealed similarities in the GC, CpG and repeated sequence contents. Thus it seems possible to suggest the existence of a general evolutionary mechanism supporting the spacer constant nucleotide proportions within avian rDNA genome.

Chicken rRNA gene cluster structure knowledge extends the use of birds as a model object for exploration of NOR regulation, particularly in ontogenesis and cell differentiation. Our results could be useful for developing optimal solutions in the areas of population genetics and evolution.

## Supporting Information

S1 Fig Chicken *5’ETS* sequence boundaries resulted from a multiple sequence alignment. Black frames outline the elements of the ribosomal cluster. Cluster members are schematically indicated by colored boxes. Asterisk–chicken pre-rRNA transcription initiation site. Arrow–transcription direction. Double slashes schematically point the hidden area of alignment. Extreme right and left–accession numbers and taxa abbreviations relating to the sequences located before and after the hidden area of the alignment, respectively. G. gal–*Gallus gallus*; R. nor–*Rattus norvegicus*; C. por–*Crocodylus porosus*; X. laev–*Xenopus laevis*; H. sap–*Homo sapiens*; M. mus–*Mus musculus*. KT445934.2_G. gal–sequence assembled by raw read tiling.
(TIF)

S2 Fig *18S rRNA* gene sequence boundaries resulted from a multiple sequence alignment. Indications are the same as in S1 Fig.
(TIF)

S3 Fig Chicken *ITS1* sequence boundaries resulted from a multiple sequence alignment. Indications are the same as in S1 Fig.
(TIF)

S4 Fig Chicken *ITS1* ssDNA secondary structures under the conditions of T = 60°C, [K^+^] = 50 mM. (PDF)

S5 Fig Chicken *5*.*8S rRNA* gene sequence boundaries resulted from a multiple sequence alignment. Indications are the same as in S1 Fig.
(TIF)

S6 Fig Chicken *ITS2* sequence boundaries resulted from a multiple sequence alignment. Indications are the same as in S1 Fig.
(TIF)

S7 Fig Chicken *ITS2* ssDNA secondary structures under the conditions of T = 60°C, [K^+^] = 50 mM. (PDF)

S8 Fig Chicken *28S rRNA* gene sequence boundaries resulted from a multiple sequence alignment. Indications are the same as in S1 Fig.
(TIF)

S9 Fig Chicken *3’ETS* sequence boundaries resulted from a multiple sequence alignment. Indications are the same as in S1 Fig.
(TIF)

S1 Table Raw read assembly verification using WGS contigs and annotated sequences. (PDF)

S2 Table Multiple alignment matrix for chicken rRNA gene cluster boundaries search using annotated fragments of ribosomal clusters of *Homo sapiens*, *Rattus norvegicus*, *Mus musculus*, *Xenopus laevis* and *Crocodylus porosus*. (PDF)

S3 Table De novo and re-assembled rRNA gene cluster sequence alignment. (PDF)
