# Supplementary figures and images for: Chicken rRNA Gene Cluster Structure
Source: PLoS One. 2016 Jun 14;11(6):e0157464. doi: 10.1371/journal.pone.0157464 (PMC4907446; doi:10.1371/journal.pone.0157464)

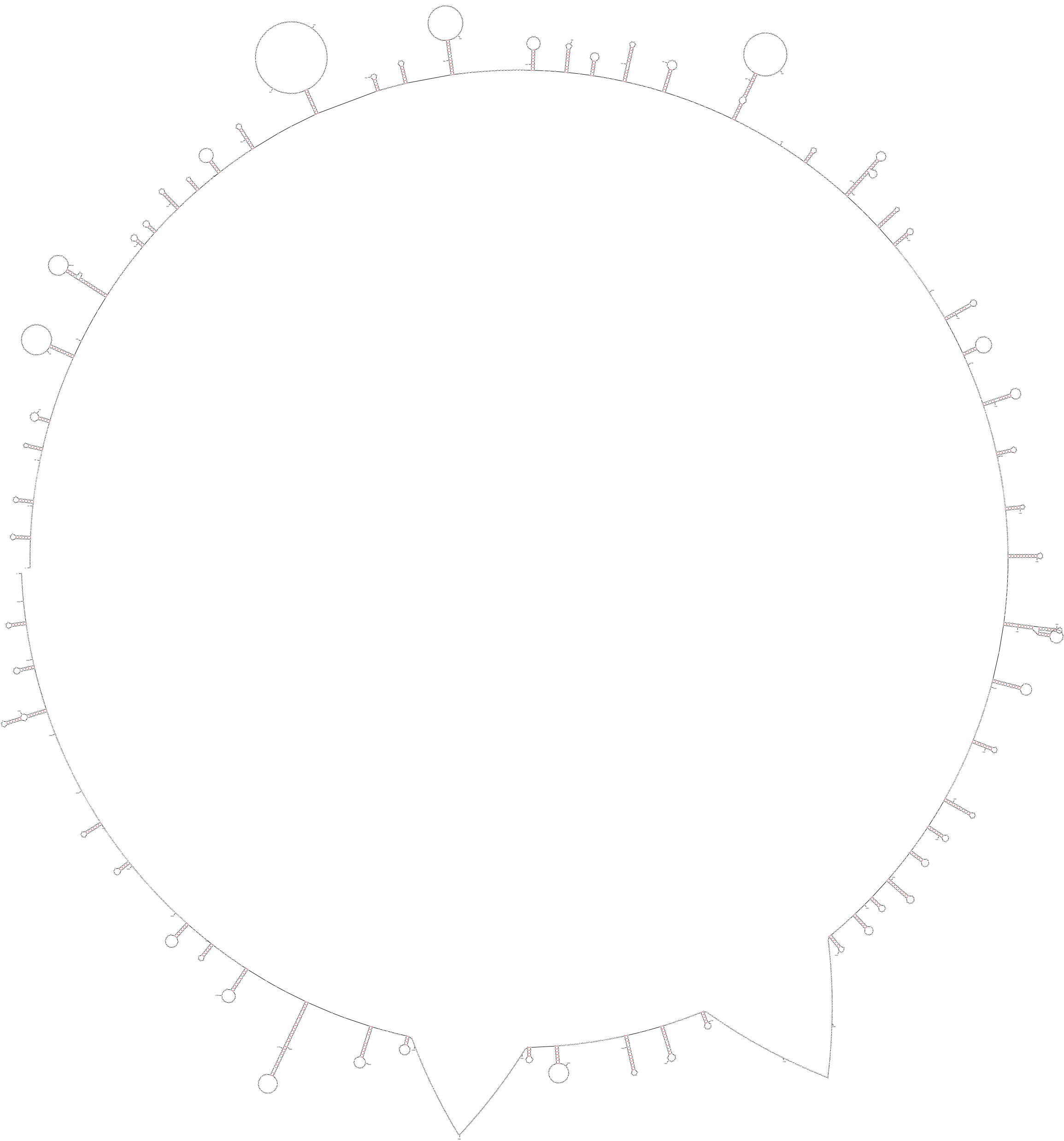

*dG = -183.23 16Feb08-06-54-32*

Supplement: S4 Fig — (PDF) [file pone.0157464.s004.pdf]

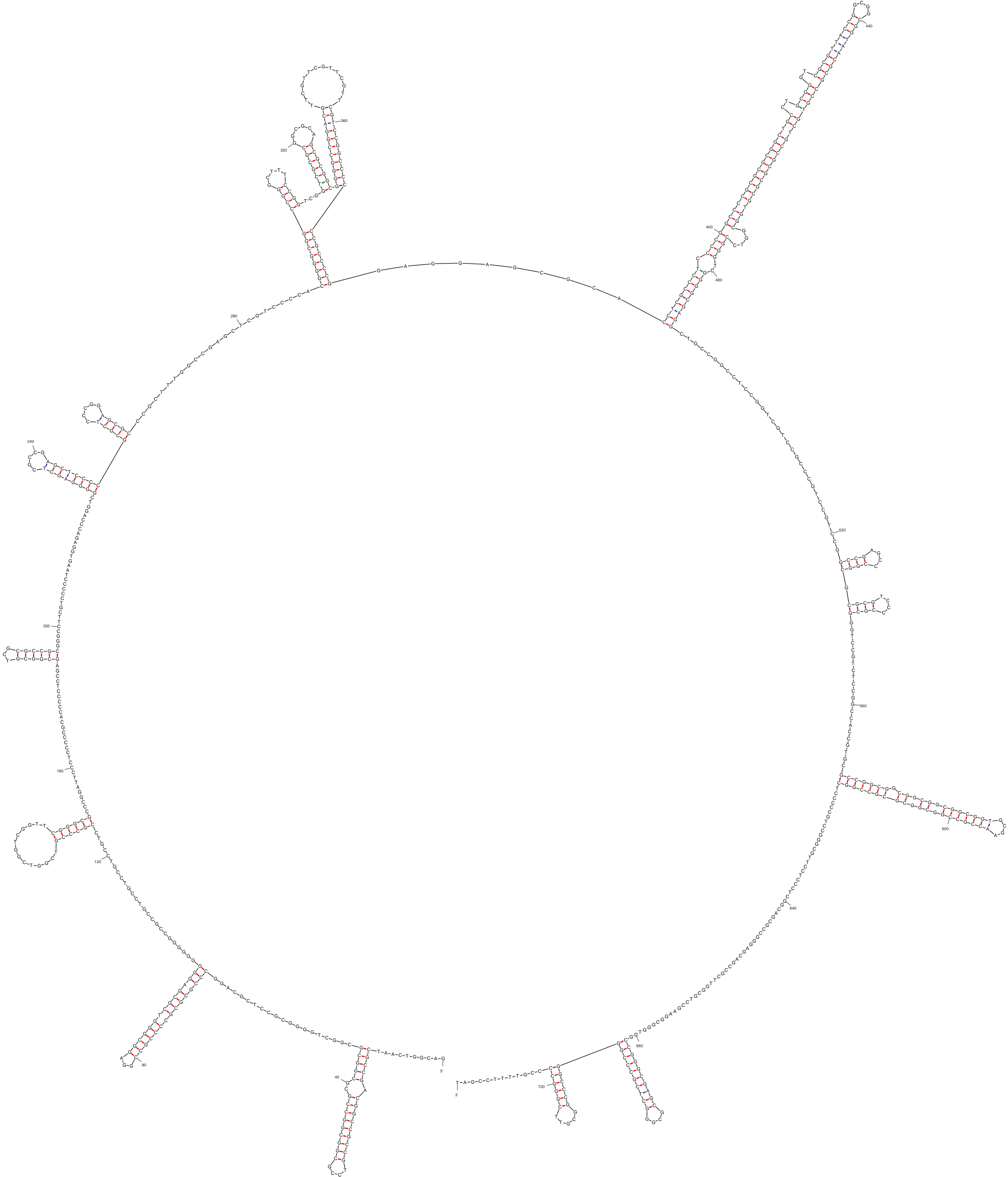

Supplement: S7 Fig — (PDF) [file pone.0157464.s007.pdf]
