# Supplementary material for: Chicken rRNA Gene Cluster Structure
Source: PLoS One. 2016 Jun 14;11(6):e0157464. doi: 10.1371/journal.pone.0157464 (PMC4907446; doi:10.1371/journal.pone.0157464)
Supplement: S1 Table — (PDF) [file pone.0157464.s010.pdf]

S1 Table

Raw read assembly verification using WGS contigs and annotated sequences

										1	1111111111
	1	1111111112	2222222223	3333333334	4444444445	5555555556	6666666667	7777777778	8888888889	9999999990	0000000001
	1234567890	1234567890	1234567890	1234567890	1234567890	1234567890	1234567890	1234567890	1234567890	1234567890	1234567890
AADN03001677	----------	----------	----------	----------	----------	----------	----------	----------	----------	----------	----------
AADN03001778	----------	----------	----------	----------	----------	----------	----------	AGGACTCGGC	TCCGGTAGTG	GCGGTGAGCG	GGCGCTCGCG
AADN03001785	----------	----------	----------	----------	----------	----------	----------	----------	----------	----------	----------
AADN03001786	----------	----------	----------	----------	----------	----------	----------	----------	----------	----------	----------
AADN03001788	----------	----------	----------	----------	----------	----------	----------	----------	----------	----------	----------
AADN03014081	GGTAGTCGGC	GCCTATGGGA	CTAGAACGTT	TTTTTCGGAT	GCCTTATATG	TTCGTCTGTA	GGAGCGAGTG	AGGACTCGGC	TCCAAGGGTG	GCGGTGAGCG	GGCGCTCG--
AADN03015064	GGTAGTCGGC	GCCTATGGGA	CTAGAACGTT	TTTTTCGGAT	GCCTTATATG	TTCGTCTGTA	GGAGCGAGTG	AGGACTCGGC	TCCGGTAGTG	GCGGTGAGCG	GGCGCTCGCG
AADN03026634	----------	----------	----------	----------	----------	----------	----------	----------	----------	----------	----------
NW_003775878	GGTAGTCGGC	GCCTATGGGA	CTAGAACGTT	TTTTTCGGAT	GCCTTATATG	TTCGTCTGTA	GGAGCGAGTG	AGGACTCGGC	TCCGGTAGTG	GCGGTGAGCG	GGCGCTCGCG
DQ018752	----------	----------	----------	----------	----------	----------	----------	----------	----------	----------	----------
DQ018757	----------	----------	----------	----------	----------	----------	----------	----------	----------	----------	----------
DQ112354	GGTAGTCGGC	GCCTATGGGG	CTAGAACGTT	TTTTTCGGAT	GCCTTATATG	TTCGTCTGTA	GGAGCGAGTG	AGGACTCGGC	TCCGGTAGTG	GCGGTGAGCG	GGCGCTCGCG
FM165414	----------	----------	----------	----------	----------	----------	----------	----------	----------	----------	----------
FM165415	----------	----------	----------	----------	----------	----------	----------	----------	----------	----------	----------
AADN03000430	----------	----------	----------	----------	----------	----------	----------	----------	----------	----------	----------
AADN03001784	----------	----------	----------	----------	----------	----------	----------	----------	----------	----------	----------
AADN03001783	----------	----------	----------	----------	----------	----------	----------	----------	----------	----------	----------
DQ018754	----------	----------	----------	----------	----------	----------	----------	----------	----------	----------	----------
AADN03001782	----------	----------	----------	----------	----------	----------	----------	----------	----------	----------	----------
JN639848	----------	----------	----------	----------	----------	----------	----------	----------	----------	----------	----------
AADN03001670	----------	----------	----------	----------	----------	----------	----------	----------	----------	----------	----------
HQ873432	----------	----------	----------	----------	----------	----------	----------	----------	----------	----------	----------
AADN03001774	----------	----------	----------	----------	----------	----------	----------	----------	----------	----------	----------
AADN03001775	----------	----------	----------	----------	----------	----------	----------	----------	----------	----------	----------
EF552813	----------	----------	----------	----------	----------	----------	----------	----------	----------	----------	----------
AADN03022685	----------	----------	----------	----------	----------	----------	----------	----------	----------	----------	----------
AADN03019346	----------	----------	----------	----------	----------	----------	----------	----------	----------	----------	----------
AADN03001776	----------	----------	----------	----------	----------	----------	----------	----------	----------	----------	----------
DQ018756	----------	----------	----------	----------	----------	----------	----------	----------	----------	----------	----------
AF173612	----------	----------	----------	----------	----------	----------	----------	----------	----------	----------	----------
Gallus_gallus_KT445934.2	GGTAGTCGGC	GCCTATGGGG	CTAGAACGTT	TTTTTCGGAT	GCCTTATATG	TTCGTCTGTA	GGAGCGAGTG	AGGACTCGGC	TCCGGTAGTG	GCGGTGAGCG	GGCGCTCGCG

	1111111111	1111111111	1111111111	1111111111	1111111111	1111111111	1111111111	1111111111	1111111112	2222222222	2222222222
	1111111112	2222222223	3333333334	4444444445	5555555556	6666666667	7777777778	8888888889	9999999990	0000000001	1111111112
	1234567890	1234567890	1234567890	1234567890	1234567890	1234567890	1234567890	1234567890	1234567890	1234567890	1234567890
AADN03001677	----------	----------	----------	----------	----------	----------	----------	----------	----------	----------	----------
AADN03001778	AGCAGGGTTG	ACCGGCCGGC	CGCCTAGAGA	GGGGA--GTC	GGCGGCGGCG	GCGGCGGCTT	TCTCGGGCAT	CGGTTCGTTC	GATCGGTCCG	GTCGCTTCGG	TTTGTCCGTC
AADN03001785	----------	----------	----------	----------	----------	----------	----------	----------	----------	----------	----------
AADN03001786	----------	----------	----------	----------	----------	----------	----------	----------	----------	----------	----------
AADN03001788	----------	----------	----------	----------	----------	----------	----------	----------	----------	----------	----------
AADN03014081	----------	----------	----------	----------	----------	----------	----------	----------	----------	----------	----------
AADN03015064	AGCAGGGTTG	ACCGGCCGGC	CGCCTAGAGA	GGGGATCGGC	GGGGGCGGCG	GCGGCGGCTT	TCTCGGGCAT	CGGTTCGTTC	GATCGGTCCG	GTCGCTTCGG	TTTGTCCGTC
AADN03026634	----------	----------	----------	----------	----------	----------	----------	----------	----------	----------	----------
NW_003775878	AGCAGGGTTG	ACCGGCCGGC	CGCCTAGAGA	GGGGATCGGC	GGGGGCGGCG	GCGGCGGCTT	TCTCGGGCAT	CGGTTCGTTC	GATCGGTCCG	GTCGCTTCGG	TTTGTCCGTC
DQ018752	----------	----------	----------	----------	----------	----------	----------	----------	----------	----------	----------
DQ018757	----------	----------	----------	----------	----------	----------	----------	----------	----------	----------	----------
DQ112354	AGCAGGGTTG	ACCGGCCGGC	CGCCTAGAGA	GGGGATCGGC	GGCGGCGGCG	GCGGC---TT	TCTCGGGCAT	CGGTTCGTTC	GATCGGTCCG	GTCGCTTCGG	TTTGTCCGTC
FM165414	----------	----------	----------	----------	----------	----------	----------	----------	----------	----------	----------

```
FM165415                    ---------- ---------- ---------- ---------- ---------- ---------- ---------- ---------- ---------- ---------- ----------
AADN03000430                ---------- ---------- ---------- ---------- ---------- ---------- ---------- ---------- ---------- ---------- ----------
AADN03001784                ---------- ---------- ---------- ---------- ---------- ---------- ---------- ---------- ---------- ---------- ----------
AADN03001783                ---------- ---------- ---------- ---------- ---------- ---------- ---------- ---------- ---------- ---------- ----------
DQ018754                    ---------- ---------- ---------- ---------- ---------- ---------- ---------- ---------- ---------- ---------- ----------
AADN03001782                ---------- ---------- ---------- ---------- ---------- ---------- ---------- ---------- ---------- ---------- ----------
JN639848                    ---------- ---------- ---------- ---------- ---------- ---------- ---------- ---------- ---------- ---------- ----------
AADN03001670                ---------- ---------- ---------- ---------- ---------- ---------- ---------- ---------- ---------- ---------- ----------
HQ873432                    ---------- ---------- ---------- ---------- ---------- ---------- ---------- ---------- ---------- ---------- ----------
AADN03001774                ---------- ---------- ---------- ---------- ---------- ---------- ---------- ---------- ---------- ---------- ----------
AADN03001775                ---------- ---------- ---------- ---------- ---------- ---------- ---------- ---------- ---------- ---------- ----------
EF552813                    ---------- ---------- ---------- ---------- ---------- ---------- ---------- ---------- ---------- ---------- ----------
AADN03022685                ---------- ---------- ---------- ---------- ---------- ---------- ---------- ---------- ---------- ---------- ----------
AADN03019346                ---------- ---------- ---------- ---------- ---------- ---------- ---------- ---------- ---------- ---------- ----------
AADN03001776                ---------- ---------- ---------- ---------- ---------- ---------- ---------- ---------- ---------- ---------- ----------
DQ018756                    ---------- ---------- ---------- ---------- ---------- ---------- ---------- ---------- ---------- ---------- ----------
AF173612                    ---------- ---------- ---------- ---------- ---------- ---------- ---------- ---------- ---------- ---------- ----------
Gallus_gallus_KT445934.2    AGCAGGGTTG ACCGGCCGGC CGCCTAGAGA GGGGATCGGC GGGGGCGGCG GCGGC---TT TCTCGGGCAT CGGTTCGTTC GATCGGTCCG GTCGCTTCGG TTTGTCCGTC

                            2222222222 2222222222 2222222222 2222222222 2222222222 2222222222 2222222222 2222222223 3333333333 3333333333 3333333333
                            2222222223 3333333334 4444444445 5555555556 6666666667 7777777778 8888888889 9999999990 0000000001 1111111112 2222222223
                            1234567890 1234567890 1234567890 1234567890 1234567890 1234567890 1234567890 1234567890 1234567890 1234567890 1234567890
AADN03001677                ---------- ---------- ---------- ---------- ---------- ---------- ---------- ---------- ---------- ---------- ----------
AADN03001778                GCTCCTCATC CCGCAGCTCT GTCCTGGGCT AAGGCGGTTT TGCAGGCGAG CAGCGAAAAA AAGCCGGAGA AGGCGAGAGA GAGGCAAGAA GCAAGCCGGC TCCCGCGCCG
AADN03001785                ---------- ---------- ---------- ---------- ---------- ---------- ---------- ---------- ---------- ---------- ----------
AADN03001786                ---------- ---------- ---------- ---------- ---------- ---------- ---------- ---------- ---------- ---------- ----------
AADN03001788                ---------- ---------- ---------- ---------- ---------- ---------- ---------- ---------- ---------- ---------- ----------
AADN03014081                ---------- ---------- ---------- ---------- ---------- ---------- ---------- ---------- ---------- ---------- ----------
AADN03015064                GCTCCTCATC CCGCAGCTCT GTCCTGGGCT AAGGCGGTTT TGCAGGCGAG CAGCGAAAAA AAGCCGGAGA AGGCGAGAGA GAGGCAAGAA GCAAGCCGGC TCCCGCGCCG
AADN03026634                ---------- ---------- ---------- ---------- ---------- ---------- ---------- ---------- ---------- ---------- ----------
NW_003775878                GCTCCTCATC CCGCAGCTCT GTCCTGGGCT AAGGCGGTTT TGCAGGCGAG CAGCGAAAAA AAGCCGGAGA AGGCGAGAGA GAGGCAAGAA GCAAGCCGGC TCCCGCGCCG
DQ018752                    ---------- ---------- ---------- ---------- ---------- ---------- ---------- ---------- ---------- ---------- ----------
DQ018757                    ---------- ---------- ---------- ---------- ---------- ---------- ---------- ---------- ---------- ---------- ----------
DQ112354                    GCTCCTCATC CCGCAGCTCT GTCCTGGGCT AAGGCGGTTT TGCAGGCGAG CAGCGAAAAA AAGCCGGAGA AGGCGA---- ---------- ---------- ----------
FM165414                    ---------- ---------- ---------- ---------- ---------- ---------- ---------- ---------- ---------- ---------- ----------
FM165415                    ---------- ---------- ---------- ---------- ---------- ---------- ---------- ---------- ---------- ---------- ----------
AADN03000430                ---------- ---------- ---------- ---------- ---------- ---------- ---------- ---------- ---------- ---------- ----------
AADN03001784                ---------- ---------- ---------- ---------- ---------- ---------- ---------- ---------- ---------- ---------- ----------
AADN03001783                ---------- ---------- ---------- ---------- ---------- ---------- ---------- ---------- ---------- ---------- ----------
DQ018754                    ---------- ---------- ---------- ---------- ---------- ---------- ---------- ---------- ---------- ---------- ----------
AADN03001782                ---------- ---------- ---------- ---------- ---------- ---------- ---------- ---------- ---------- ---------- ----------
JN639848                    ---------- ---------- ---------- ---------- ---------- ---------- ---------- ---------- ---------- ---------- ----------
AADN03001670                ---------- ---------- ---------- ---------- ---------- ---------- ---------- ---------- ---------- ---------- ----------
HQ873432                    ---------- ---------- ---------- ---------- ---------- ---------- ---------- ---------- ---------- ---------- ----------
AADN03001774                ---------- ---------- ---------- ---------- ---------- ---------- ---------- ---------- ---------- ---------- ----------
AADN03001775                ---------- ---------- ---------- ---------- ---------- ---------- ---------- ---------- ---------- ---------- ----------
EF552813                    ---------- ---------- ---------- ---------- ---------- ---------- ---------- ---------- ---------- ---------- ----------
AADN03022685                ---------- ---------- ---------- ---------- ---------- ---------- ---------- ---------- ---------- ---------- ----------
AADN03019346                ---------- ---------- ---------- ---------- ---------- ---------- ---------- ---------- ---------- ---------- ----------
AADN03001776                ---------- ---------- ---------- ---------- ---------- ---------- ---------- ---------- ---------- ---------- ----------
DQ018756                    ---------- ---------- ---------- ---------- ---------- ---------- ---------- ---------- ---------- ---------- ----------
AF173612                    ---------- ---------- ---------- ---------- ---------- ---------- ---------- ---------- ---------- ---------- ----------
Gallus_gallus_KT445934.2    GCTCCTCATC CCGCAGCTCT GTCCTGGGCT AAGGCGGTTT TGCAGGCGAG CAGCGAAAAA AAGCCGGAGA AGGCGAGAGA GAGGCAAGAA GCAAGCCGGC TCCCGCGCCG
```

```
                            3333333333 3333333333 3333333333 3333333333 3333333333 3333333333 3333333334 4444444444 4444444444 4444444444 4444444444
                            3333333334 4444444445 5555555556 6666666667 7777777778 8888888889 9999999990 0000000001 1111111112 2222222223 3333333334
                            1234567890 1234567890 1234567890 1234567890 1234567890 1234567890 1234567890 1234567890 1234567890 1234567890 1234567890
AADN03001677                ---------- ---------- ---------- ---------- ---------- ---------- ---------- ---------- ---------- ---------- ----------
AADN03001778                CCAGGGCGAA GGCGAGAGAG AGAGGGAGAC GAGAAGGGCA CGGGCCGGTC TGCCGGCACC CGAACGTAGG ATGGCCGGGG GCGTCCCCGG CGGGTCCCGC CGCGATGGAA
AADN03001785                ---------- ---------- ---------- ---------- ---------- ---------- ---------- ---------- ---------- ---------- ----------
AADN03001786                ---------- ---------- ---------- ---------- ---------- ---------- ---------- ---------- ---------- ---------- ----------
AADN03001788                ---------- ---------- ---------- ---------- ---------- ---------- ---------- ---------- ---------- ---------- ----------
AADN03014081                ---------- ---------- ---------- ---------- ---------- ---------- ---------- ---------- ---------- ---------- ----------
AADN03015064                CCAGGGCGAA GGCGAGAGAG AGAGGGAGAC GAGAAGGGCA CGGGCCGGTC TGCCGGCACC CGAACGTAGG ATGGCCGGGG GCGTCCCCGG CGGGTCCCGC CGCGATGGAA
AADN03026634                ---------- ---------- ---------- ---------- ---------- ---------- ---------- ---------- ---------- ---------- ----------
NW_003775878                CCAGGGCGAA GGCGAGAGAG AGAGGGAGAC GAGAAGGGCA CGGGCCGGTC TGCCGGCACC CGAACGTAGG ATGGCCGGGG GCGTCCCCGG CGGGTCCCGC CGCGATGGAA
DQ018752                    ---------- ---------- ---------- ---------- ---------- ---------- ---------- ---------- ---------- ---------- ----------
DQ018757                    ---------- ---------- ---------- ---------- ---------- ---------- ---------- ---------- ---------- ---------- ----------
DQ112354                    ---------- ---------- ---------- ---------- ---------- ---------- ---------- ---------- ---------- ---------- ----------
FM165414                    ---------- ---------- ---------- ---------- ---------- ---------- ---------- ---------- ---------- ---------- ----------
FM165415                    ---------- ---------- ---------- ---------- ---------- ---------- ---------- ---------- ---------- ---------- ----------
AADN03000430                ---------- ---------- ---------- ---------- ---------- ---------- ---------- ---------- ---------- ---------- ----------
AADN03001784                ---------- ---------- ---------- ---------- ---------- ---------- ---------- ---------- ---------- ---------- ----------
AADN03001783                ---------- ---------- ---------- ---------- ---------- ---------- ---------- ---------- ---------- ---------- ----------
DQ018754                    ---------- ---------- ---------- ---------- ---------- ---------- ---------- ---------- ---------- ---------- ----------
AADN03001782                ---------- ---------- ---------- ---------- ---------- ---------- ---------- ---------- ---------- ---------- ----------
JN639848                    ---------- ---------- ---------- ---------- ---------- ---------- ---------- ---------- ---------- ---------- ----------
AADN03001670                ---------- ---------- ---------- ---------- ---------- ---------- ---------- ---------- ---------- ---------- ----------
HQ873432                    ---------- ---------- ---------- ---------- ---------- ---------- ---------- ---------- ---------- ---------- ----------
AADN03001774                ---------- ---------- ---------- ---------- ---------- ---------- ---------- ---------- ---------- ---------- ----------
AADN03001775                ---------- ---------- ---------- ---------- ---------- ---------- ---------- ---------- ---------- ---------- ----------
EF552813                    ---------- ---------- ---------- ---------- ---------- ---------- ---------- ---------- ---------- ---------- ----------
AADN03022685                ---------- ---------- ---------- ---------- ---------- ---------- ---------- ---------- ---------- ---------- ----------
AADN03019346                ---------- ---------- ---------- ---------- ---------- ---------- ---------- ---------- ---------- ---------- ----------
AADN03001776                ---------- ---------- ---------- ---------- ---------- ---------- ---------- ---------- ---------- ---------- ----------
DQ018756                    ---------- ---------- ---------- ---------- ---------- ---------- ---------- ---------- ---------- ---------- ----------
AF173612                    ---------- ---------- ---------- ---------- ---------- ---------- ---------- ---------- ---------- ---------- ----------
Gallus_gallus_KT445934.2    CCAGGGCGAA GGCGAGAGAG AGAGGGAGAC GAGAAGGGCA CGGGCCGGTC TGCCGGCACC CGAACGTAGG ATGGCCGGGG GCGTCCCCGG CGGGTCCCGC CGCGATGGAA

                            4444444444 4444444444 4444444444 4444444444 4444444444 4444444445 5555555555 5555555555 5555555555 5555555555 5555555555
                            4444444445 5555555556 6666666667 7777777778 8888888889 9999999990 0000000001 1111111112 2222222223 3333333334 4444444445
                            1234567890 1234567890 1234567890 1234567890 1234567890 1234567890 1234567890 1234567890 1234567890 1234567890 1234567890
AADN03001677                ---------- ---------- ---------- ---------- ---------- ---------- ---------- ---------- ---------- ---------- ----------
AADN03001778                GAGGGGGACC CGGAGGTCGT AGGTCGTGGC GGCGTCGCCT CGTCCTCCTT TCGCACCGCA TTCTCACCCG CACGCGGGAG CCCCGGCCGA TTCGTGGCGC TCCTCGGGCG
AADN03001785                ---------- ---------- ---------- ---------- ---------- ---------- ---------- ---------- ---------- ---------- ----------
AADN03001786                ---------- ---------- ---------- ---------- ---------- ---------- ---------- ---------- ---------- ---------- ----------
AADN03001788                ---------- ---------- ---------- ---------- ---------- ---------- ---------- ---------- ---------- ---------- ----------
AADN03014081                ---------- ---------- ---------- ---------- ---------- ---------- ---------- ---------- ---------- ---------- ----------
AADN03015064                GAGGGGGACC CGGAGGTCGT AGGTCGTGGC GGCGTCGCCT CGTCCTCCTT TCGCACCGCA TTCTCACCCG CACGCGGGAG CCCCGGCCGA TTCGTGGCGC TCCTCGGGCG
AADN03026634                ---------- ---------- ---------- ---------- ---------- ---------- ---------- ---------- ---------- ---------- ----------
NW_003775878                GAGGGGGACC CGGAGGTCGT AGGTCGTGGC GGCGTCGCCT CGTCCTCCTT TCGCACCGCA TTCTCACCCG CACGCGGGAG CCCCGGCCGA TTCGTGGCGC TCCTCGGGCG
DQ018752                    ---------- ---------- ---------- ---------- ---------- ---------- ---------- ---------- ---------- ---------- ----------
DQ018757                    ---------- ---------- ---------- ---------- ---------- ---------- ---------- ---------- ---------- ---------- ----------
DQ112354                    ---------- ---------- ---------- ---------- ---------- ---------- ---------- ---------- ---------- ---------- ----------
FM165414                    ---------- ---------- ---------- ---------- ---------- ---------- ---------- ---------- ---------- ---------- ----------
FM165415                    ---------- ---------- ---------- ---------- ---------- ---------- ---------- ---------- ---------- ---------- ----------
AADN03000430                ---------- ---------- ---------- ---------- ---------- ---------- ---------- ---------- ---------- ---------- ----------
AADN03001784                ---------- ---------- ---------- ---------- ---------- ---------- ---------- ---------- ---------- ---------- ----------
AADN03001783                ---------- ---------- ---------- ---------- ---------- ---------- ---------- ---------- ---------- ---------- ----------
```

```
DQ018754                     ---------- ---------- ---------- ---------- ---------- ---------- ---------- ---------- ---------- ---------- ----------
AADN03001782                 ---------- ---------- ---------- ---------- ---------- ---------- ---------- ---------- ---------- ---------- ----------
JN639848                     ---------- ---------- ---------- ---------- ---------- ---------- ---------- ---------- ---------- ---------- ----------
AADN03001670                 ---------- ---------- ---------- ---------- ---------- ---------- ---------- ---------- ---------- ---------- ----------
HQ873432                     ---------- ---------- ---------- ---------- ---------- ---------- ---------- ---------- ---------- ---------- ----------
AADN03001774                 ---------- ---------- ---------- ---------- ---------- ---------- ---------- ---------- ---------- ---------- ----------
AADN03001775                 ---------- ---------- ---------- ---------- ---------- ---------- ---------- ---------- ---------- ---------- ----------
EF552813                     ---------- ---------- ---------- ---------- ---------- ---------- ---------- ---------- ---------- ---------- ----------
AADN03022685                 ---------- ---------- ---------- ---------- ---------- ---------- ---------- ---------- ---------- ---------- ----------
AADN03019346                 ---------- ---------- ---------- ---------- ---------- ---------- ---------- ---------- ---------- ---------- ----------
AADN03001776                 ---------- ---------- ---------- ---------- ---------- ---------- ---------- ---------- ---------- ---------- ----------
DQ018756                     ---------- ---------- ---------- ---------- ---------- ---------- ---------- ---------- ---------- ---------- ----------
AF173612                     ---------- ---------- ---------- ---------- ---------- ---------- ---------- ---------- ---------- ---------- ----------
Gallus_gallus_KT445934.2     GAGGGGGACC CGGAGGTCGT AGGTCGTGGC GGCGTCGCCT CGTCCTCCTT TCGCACCGCA TTCTCACCCG CACGCGGGAG CCCCGGCCGA TTCGTGGCGC TCCTCGGGCG

                             5555555555 5555555555 5555555555 5555555555 5555555556 6666666666 6666666666 6666666666 6666666666 6666666666 6666666666
                             5555555556 6666666667 7777777778 8888888889 9999999990 0000000001 1111111112 2222222223 3333333334 4444444445 5555555556
                             1234567890 1234567890 1234567890 1234567890 1234567890 1234567890 1234567890 1234567890 1234567890 1234567890 1234567890
AADN03001677                 ---------- ---------- ---------- ---------- ---------- ---------- ---------- ---------- ---------- ---------- ----------
AADN03001778                 CGTCGGGGAG GCTTCCCGGC GGGCCGGCTC TATCCCGCTC C--------- ---------- ---------- ---------- ---------- ---------- ----------
AADN03001785                 ---------- ---------- ---------- ---------- ---------- ---------- ---------- ---------- ---------- ---------- ----------
AADN03001786                 ---------- ---------- ---------- ---------- ---------- ---------- ---------- ---------- ---------- ---------- ----------
AADN03001788                 ---------- ---------- ---------- ---------- ---------- ---------- ---------- ---------- ---------- ---------- ----------
AADN03014081                 ---------- ---------- ---------- ---------- ---------- ---------- ---------- ---------- ---------- ---------- ----------
AADN03015064                 CGTCGGGGAG GCTTCCCGGC GGGCCGGCTC TATCCCGCTC CCCGGCTCGT TCGGGGTGGC GTGGGGCGGG CCGGTGTTCA GGCACGGGCG AGCACCTCTC GTCGGACGTT
AADN03026634                 ---------- ---------- ---------- ---------- ---------- ---------- ---------- ---------- ---------- ---------- ----------
NW_003775878                 CGTCGGGGAG GCTTCCCGGC GGGCCGGCTC TATCCCGCTC CCCGGCTCGT TCGGGGTGGC GTGGGGCGGG CCGGTGTTCA GGCACGGGCG AGCACCTCTC GTCGGACGTT
DQ018752                     ---------- ---------- ---------- ---------- ---------- ---------- ---------- ---------- ---------- ---------- ----------
DQ018757                     ---------- ---------- ---------- ---------- ---------- ---------- ---------- ---------- ---------- ---------- ----------
DQ112354                     ---------- ---------- ---------- ---------- ---------- ---------- ---------- ---------- ---------- ---------- ----------
FM165414                     ---------- ---------- ---------- ---------- ---------- ---------- ---------- ---------- ---------- ---------- ----------
FM165415                     ---------- ---------- ---------- ---------- ---------- ---------- ---------- ---------- ---------- ---------- ----------
AADN03000430                 ---------- ---------- ---------- ---------- ---------- ---------- ---------- ---------- ---------- ---------- ----------
AADN03001784                 ---------- ---------- ---------- ---------- ---------- ---------- ---------- ---------- ---------- ---------- ----------
AADN03001783                 ---------- ---------- ---------- ---------- ---------- ---------- ---------- ---------- ---------- ---------- ----------
DQ018754                     ---------- ---------- ---------- ---------- ---------- ---------- ---------- ---------- ---------- ---------- ----------
AADN03001782                 ---------- ---------- ---------- ---------- ---------- ---------- ---------- ---------- ---------- ---------- ----------
JN639848                     ---------- ---------- ---------- ---------- ---------- ---------- ---------- ---------- ---------- ---------- ----------
AADN03001670                 ---------- ---------- ---------- ---------- ---------- ---------- ---------- ---------- ---------- ---------- ----------
HQ873432                     ---------- ---------- ---------- ---------- ---------- ---------- ---------- ---------- ---------- ---------- ----------
AADN03001774                 ---------- ---------- ---------- ---------- ---------- ---------- ---------- ---------- ---------- ---------- ----------
AADN03001775                 ---------- ---------- ---------- ---------- ---------- ---------- ---------- ---------- ---------- ---------- ----------
EF552813                     ---------- ---------- ---------- ---------- ---------- ---------- ---------- ---------- ---------- ---------- ----------
AADN03022685                 ---------- ---------- ---------- ---------- ---------- ---------- ---------- ---------- ---------- ---------- ----------
AADN03019346                 ---------- ---------- ---------- ---------- ---------- ---------- ---------- ---------- ---------- ---------- ----------
AADN03001776                 ---------- ---------- ---------- ---------- ---------- ---------- ---------- ---------- ---------- ---------- ----------
DQ018756                     ---------- ---------- ---------- ---------- ---------- ---------- ---------- ---------- ---------- ---------- ----------
AF173612                     ---------- ---------- ---------- ---------- ---------- ---------- ---------- ---------- ---------- ---------- ----------
Gallus_gallus_KT445934.2     CGTCGGGGAG GCTTCCCGGC GGGCCGGCTC TATCCCGCTC CCCGGCTCGT TCGGGGTGGC GTGGGGCGGG CCGGTGTTCA GGCACGGGCG AGCACCTCTC GTCGGACGTT

                             6666666666 6666666666 6666666666 6666666667 7777777777 7777777777 7777777777 7777777777 7777777777 7777777777 7777777777
                             6666666667 7777777778 8888888889 9999999990 0000000001 1111111112 2222222223 3333333334 4444444445 5555555556 6666666667
                             1234567890 1234567890 1234567890 1234567890 1234567890 1234567890 1234567890 1234567890 1234567890 1234567890 1234567890
AADN03001677                 ---------- ---------- ---------- ---------- ---------- ---------- ---------- ---------- ---------- ---------- ----------
AADN03001778                 ---------- ---------- ---------- ---------- ---------- ---------- ---------- ---------- ---------- ---------- ----------
```

```
AADN03001785              ---------- ---------- ---------- ---------- ---------- ---------- ---------- ---------- ---------- ---------- ----------
AADN03001786              ---------- ---------- ---------- ---------- ---------- ---------- ---------- ---------- ---------- ---------- ----------
AADN03001788              ---------- ---------- ---------- ---------- ---------- ---------- ---------- ---------- ---------- ---------- ----------
AADN03014081              ---------- ---------- ---------- ---------- ---------- ---------- ---------- ---------- ---------- ---------- ----------
AADN03015064              GCCCACGCAC ACCCACCTGC ACGTGCGCGT GCGGTCTTTC CGCCGCGCCT GGGGGAAGGG CTCGCGCCTT CTCCCTCCTT TCTTTCTCCT CCCCCCCACC CCCTTTCTCC
AADN03026634              ---------- ---------- ---------- ---------- ---------- ---------- ---------- ---------- ---------- ---------- ----------
NW_003775878              GCCCACGCAC ACCCACCTGC ACGTGCGCGT GCGGTCTTTC CGCCGCGCCT GGGGGAAGGG CTCGCGCCTT CTCCCTCCTT TCTTTCTCCT CCCCCCCACC CCCTTTCTCC
DQ018752                  ---------- ---------- ---------- ---------- ---------- ---------- ---------- ---------- ---------- ---------- ----------
DQ018757                  ---------- ---------- ---------- ---------- ---------- ---------- ---------- ---------- ---------- ---------- ----------
DQ112354                  ---------- ---------- ---------- ---------- ---------- ---------- ---------- ---------- ---------- ---------- ----------
FM165414                  ---------- ---------- ---------- ---------- ---------- ---------- ---------- ---------- ---------- ---------- ----------
FM165415                  ---------- ---------- ---------- ---------- ---------- ---------- ---------- ---------- ---------- ---------- ----------
AADN03000430              ---------- ---------- ---------- ---------- ---------- ---------- ---------- ---------- ---------- ---------- ----------
AADN03001784              ---------- ---------- ---------- ---------- ---------- ---------- ---------- ---------- ---------- ---------- ----------
AADN03001783              ---------- ---------- ---------- ---------- ---------- ---------- ---------- ---------- ---------- ---------- ----------
DQ018754                  ---------- ---------- ---------- ---------- ---------- ---------- ---------- ---------- ---------- ---------- ----------
AADN03001782              ---------- ---------- ---------- ---------- ---------- ---------- ---------- ---------- ---------- ---------- ----------
JN639848                  ---------- ---------- ---------- ---------- ---------- ---------- ---------- ---------- ---------- ---------- ----------
AADN03001670              ---------- ---------- ---------- ---------- ---------- ---------- ---------- ---------- ---------- ---------- ----------
HQ873432                  ---------- ---------- ---------- ---------- ---------- ---------- ---------- ---------- ---------- ---------- ----------
AADN03001774              ---------- ---------- ---------- ---------- ---------- ---------- ---------- ---------- ---------- ---------- ----------
AADN03001775              ---------- ---------- ---------- ---------- ---------- ---------- ---------- ---------- ---------- ---------- ----------
EF552813                  ---------- ---------- ---------- ---------- ---------- ---------- ---------- ---------- ---------- ---------- ----------
AADN03022685              ---------- ---------- ---------- ---------- ---------- ---------- ---------- ---------- ---------- ---------- ----------
AADN03019346              ---------- ---------- ---------- ---------- ---------- ---------- ---------- ---------- ---------- ---------- ----------
AADN03001776              ---------- ---------- ---------- ---------- ---------- ---------- ---------- ---------- ---------- ---------- ----------
DQ018756                  ---------- ---------- ---------- ---------- ---------- ---------- ---------- ---------- ---------- ---------- ----------
AF173612                  ---------- ---------- ---------- ---------- ---------- ---------- ---------- ---------- ---------- ---------- ----------
Gallus_gallus_KT445934.2  GCCCACGCAC ACCCACCTGC ACGTGCGCGT GCGGTCTTTC CGCCGCGCCT GGGGGAAGGG CTCGCGCCTT CTCCCTCCTT TCTTTCTCCT CCCCCCCACC CCCTTTCTCC

                          7777777777 7777777777 7777777778 8888888888 8888888888 8888888888 8888888888 8888888888 8888888888 8888888888 8888888888
                          7777777778 8888888889 9999999990 0000000001 1111111112 2222222223 3333333334 4444444445 5555555556 6666666667 7777777778
                          1234567890 1234567890 1234567890 1234567890 1234567890 1234567890 1234567890 1234567890 1234567890 1234567890 1234567890
AADN03001677              ---------- ---------- ---------- ---------- ---------- ---------- ---------- ---------- ---------- ---------- ----------
AADN03001778              ---------- ---------- ---------- ---------- ---------- ---------- ---------- ---------- ---------- ---------- ----------
AADN03001785              ---------- ---------- ---------- ---------- ---------- ---------- ---------- ---------- ---------- ---------- ----------
AADN03001786              ---------- ---------- ---------- ---------- ---------- ---------- ---------- ---------- ---------- ---------- ----------
AADN03001788              ---------- ---------- ---------- ---------- ---------- ---------- ---------- ---------- ---------- ---------- ----------
AADN03014081              ---------- ---------- ---------- ---------- ---------- ---------- ---------- ---------- ---------- ---------- ----------
AADN03015064              CACCGATCGA TGAGGCCACT CGGGTCGCGT CGGAGAGGGC CCCCGGCGGG CCGGCGCTCT GCGCTCCCTG TCCCAGGGAA GCCGCGGCGG CGTCCGGTGT TCAGGCACGG
AADN03026634              ---------- ---------- ---------- ---------- ---------- ---------- ---------- ---------- ---------- ---------- ----------
NW_003775878              CACCGATCGA TGAGGCCACT CGGGTCGCGT CGGAGAGGGC CCCCGGCGGG CCGGCGCTCT GCGCTCCCTG TCCCAGGGAA GCCGCGGCGG CGTCCGGTGT TCAGGCACGG
DQ018752                  ---------- ---------- ---------- ---------- ---------- ---------- ---------- ---------- ---------- ---------- ----------
DQ018757                  ---------- ---------- ---------- ---------- ---------- ---------- ---------- ---------- ---------- ---------- ----------
DQ112354                  ---------- ---------- ---------- ---------- ---------- ---------- ---------- ---------- ---------- ---------- ----------
FM165414                  ---------- ---------- ---------- ---------- ---------- ---------- ---------- ---------- ---------- ---------- ----------
FM165415                  ---------- ---------- ---------- ---------- ---------- ---------- ---------- ---------- ---------- ---------- ----------
AADN03000430              ---------- ---------- ---------- ---------- ---------- ---------- ---------- ---------- ---------- ---------- ----------
AADN03001784              ---------- ---------- ---------- ---------- ---------- ---------- ---------- ---------- ---------- ---------- ----------
AADN03001783              ---------- ---------- ---------- ---------- ---------- ---------- ---------- ---------- ---------- ---------- ----------
DQ018754                  ---------- ---------- ---------- ---------- ---------- ---------- ---------- ---------- ---------- ---------- ----------
AADN03001782              ---------- ---------- ---------- ---------- ---------- ---------- ---------- ---------- ---------- ---------- ----------
JN639848                  ---------- ---------- ---------- ---------- ---------- ---------- ---------- ---------- ---------- ---------- ----------
AADN03001670              ---------- ---------- ---------- ---------- ---------- ---------- ---------- ---------- ---------- ---------- ----------
HQ873432                  ---------- ---------- ---------- ---------- ---------- ---------- ---------- ---------- ---------- ---------- ----------
```

```
AADN03001774                  ---------- ---------- ---------- ---------- ---------- ---------- ---------- ---------- ---------- ---------- ----------
AADN03001775                  ---------- ---------- ---------- ---------- ---------- ---------- ---------- ---------- ---------- ---------- ----------
EF552813                      ---------- ---------- ---------- ---------- ---------- ---------- ---------- ---------- ---------- ---------- ----------
AADN03022685                  ---------- ---------- ---------- ---------- ---------- ---------- ---------- ---------- ---------- ---------- ----------
AADN03019346                  ---------- ---------- ---------- ---------- ---------- ---------- ---------- ---------- ---------- ---------- ----------
AADN03001776                  ---------- ---------- ---------- ---------- ---------- ---------- ---------- ---------- ---------- ---------- ----------
DQ018756                      ---------- ---------- ---------- ---------- ---------- ---------- ---------- ---------- ---------- ---------- ----------
AF173612                      ---------- ---------- ---------- ---------- ---------- ---------- ---------- ---------- ---------- ---------- ----------
Gallus_gallus_KT445934.2      CACCGATCGA TGAGGCCACT CGGGTCGCGT CGGAGAGGGC CCCCGGCGGG CCGGCGCTCT GCGCTCCCTG TCCCAGGGAA GCCGCGGCGG CGTCCGGTGT TCAGGCACGG

                              8888888888 8888888889 9999999999 9999999999 9999999999 9999999999 9999999999 9999999999 9999999999 9999999999 9999999999
                              8888888889 9999999990 0000000001 1111111112 2222222223 3333333334 4444444445 5555555556 6666666667 7777777778 8888888889
                              1234567890 1234567890 1234567890 1234567890 1234567890 1234567890 1234567890 1234567890 1234567890 1234567890 1234567890
AADN03001677                  ---------- ---------- ---------- ---------- ---------- ---------- ---------- ---------- ---------- ---------- ----------
AADN03001778                  ---------- ---------- ---------- ---------- ---------- ---------- ---------- ---------- ---------- ---------- ----------
AADN03001785                  ---------- ---------- ---------- ---------- ---------- ---------- ---------- ---------- ---------- ---------- ----------
AADN03001786                  ---------- ---------- ---------- ---------- ---------- ---------- ---------- ---------- ---------- ---------- ----------
AADN03001788                  ---------- ---------- ---------- ---------- ---------- ---------- ---------- ---------- ---------- ---------- ----------
AADN03014081                  ---------- ---------- ---------- ---------- ---------- ---------- ---------- ---------- ---------- ---------- ----------
AADN03015064                  GCGGCCTCCT CTCCAGTTCG CTTCCCGTCG TTCGCGAGGT GAGGCGCTCG CCCGCTTGGG CCGAGGGCGG CGGCGGCGGC GGCTTCGGGG CGCGTGGCCT CGCCGTGCCG
AADN03026634                  ---------- ---------- ---------- ---------- ---------- ---------- ---------- ---------- ---------- ---------- ----------
NW_003775878                  GCGGCCTCCT CTCCAGTTCG CTTCCCGTCG TTCGCGAGGT GAGGCGCTCG CCCGCTTGGG CCGAGGGCGG CGGCGGCGGC GGCTTCGGGG CGCGTGGCCT CGCCGTGCCG
DQ018752                      ---------- ---------- ---------- ---------- ---------- ---------- ---------- ---------- ---------- ---------- ----------
DQ018757                      ---------- ---------- ---------- ---------- ---------- ---------- ---------- ---------- ---------- ---------- ----------
DQ112354                      ---------- ---------- ---------- ---------- ---------- ---------- ---------- ---------- ---------- ---------- ----------
FM165414                      ---------- ---------- ---------- ---------- ---------- ---------- ---------- ---------- ---------- ---------- ----------
FM165415                      ---------- ---------- ---------- ---------- ---------- ---------- ---------- ---------- ---------- ---------- ----------
AADN03000430                  ---------- ---------- ---------- ---------- ---------- ---------- ---------- ---------- ---------- ---------- ----------
AADN03001784                  ---------- ---------- ---------- ---------- ---------- ---------- ---------- ---------- ---------- ---------- ----------
AADN03001783                  ---------- ---------- ---------- ---------- ---------- ---------- ---------- ---------- ---------- ---------- ----------
DQ018754                      ---------- ---------- ---------- ---------- ---------- ---------- ---------- ---------- ---------- ---------- ----------
AADN03001782                  ---------- ---------- ---------- ---------- ---------- ---------- ---------- ---------- ---------- ---------- ----------
JN639848                      ---------- ---------- ---------- ---------- ---------- ---------- ---------- ---------- ---------- ---------- ----------
AADN03001670                  ---------- ---------- ---------- ---------- ---------- ---------- ---------- ---------- ---------- ---------- ----------
HQ873432                      ---------- ---------- ---------- ---------- ---------- ---------- ---------- ---------- ---------- ---------- ----------
AADN03001774                  ---------- ---------- ---------- ---------- ---------- ---------- ---------- ---------- ---------- ---------- ----------
AADN03001775                  ---------- ---------- ---------- ---------- ---------- ---------- ---------- ---------- ---------- ---------- ----------
EF552813                      ---------- ---------- ---------- ---------- ---------- ---------- ---------- ---------- ---------- ---------- ----------
AADN03022685                  ---------- ---------- ---------- ---------- ---------- ---------- ---------- ---------- ---------- ---------- ----------
AADN03019346                  ---------- ---------- ---------- ---------- ---------- ---------- ---------- ---------- ---------- ---------- ----------
AADN03001776                  ---------- ---------- ---------- ---------- ---------- ---------- ---------- ---------- ---------- ---------- ----------
DQ018756                      ---------- ---------- ---------- ---------- ---------- ---------- ---------- ---------- ---------- ---------- ----------
AF173612                      ---------- ---------- ---------- ---------- ---------- ---------- ---------- ---------- ---------- ---------- ----------
Gallus_gallus_KT445934.2      GCGGCCTCCT CTCCAGTTCG CTTCCCGTCG TTCGCGAGGT GAGGCGCTCG CCCGCTTGGG CCGAGGGCGG CGGCGGCGGC GGCTTCGGGG CGCGTGGCCT CGCCGTGCCG

                                       1 1111111111 1111111111 1111111111 1111111111 1111111111 1111111111 1111111111 1111111111 1111111111 1111111111
                              9999999990 0000000000 0000000000 0000000000 0000000000 0000000000 0000000000 0000000000 0000000000 0000000000 0000000001
                              9999999990 0000000001 1111111112 2222222223 3333333334 4444444445 5555555556 6666666667 7777777778 8888888889 9999999990
                              1234567890 1234567890 1234567890 1234567890 1234567890 1234567890 1234567890 1234567890 1234567890 1234567890 1234567890
AADN03001677                  ---------- ---------- ---------- ---------- ---------- ---------- ---------- ---------- ---------- ---------- ----------
AADN03001778                  ---------- ---------- ---------- ---------- ---------- ---------- ---------- ---------- ---------- ---------- ----------
AADN03001785                  ---------- ---------- ---------- ---------- ---------- ---------- ---------- ---------- ---------- ---------- ----------
AADN03001786                  ---------- ---------- ---------- ---------- ---------- ---------- ---------- ---------- ---------- ---------- ----------
AADN03001788                  ---------- ---------- ---------- ---------- ---------- ---------- ---------- ---------- ---------- ---------- ----------
AADN03014081                  ---------- ---------- ---------- ---------- ---------- ---------- ---------- ---------- ---------- ---------- ----------
```

```
AADN03015064                ACTCGTCTGT CCGCCCGCCC TGTCGGTGCC CCAGG----- ---------- ---------- ---------- ---------- ---------- ---------- ----------
AADN03026634                ---------- ---------- ---------- ---------- ---------- ---------- ---------- ---------- ---------- ---------- ----------
NW_003775878                ACTCGTCTGT CCGCCCGCCC TGTCGGTGCC CCAGGNNNNN NNNNNNNNNN NNNNNNNNNN NNNNNNNNNN NNNNNNNNNN NNNNNNNNNN NNNNNNNNNN NNNNNNNNNN
DQ018752                    ---------- ---------- ---------- ---------- ---------- ---------- ---------- ---------- ---------- ---------- ----------
DQ018757                    ---------- ---------- ---------- ---------- ---------- ---------- ---------- ---------- ---------- ---------- ----------
DQ112354                    ---------- ---------- ---------- ---------- ---------- ---------- ---------- ---------- ---------- ---------- ----------
FM165414                    ---------- ---------- ---------- ---------- ---------- ---------- ---------- ---------- ---------- ---------- ----------
FM165415                    ---------- ---------- ---------- ---------- ---------- ---------- ---------- ---------- ---------- ---------- ----------
AADN03000430                ---------- ---------- ---------- ---------- ---------- ---------- ---------- ---------- ---------- ---------- ----------
AADN03001784                ---------- ---------- ---------- ---------- ---------- ---------- ---------- ---------- ---------- ---------- ----------
AADN03001783                ---------- ---------- ---------- ---------- ---------- ---------- ---------- ---------- ---------- ---------- ----------
DQ018754                    ---------- ---------- ---------- ---------- ---------- ---------- ---------- ---------- ---------- ---------- ----------
AADN03001782                ---------- ---------- ---------- ---------- ---------- ---------- ---------- ---------- ---------- ---------- ----------
JN639848                    ---------- ---------- ---------- ---------- ---------- ---------- ---------- ---------- ---------- ---------- ----------
AADN03001670                ---------- ---------- ---------- ---------- ---------- ---------- ---------- ---------- ---------- ---------- ----------
HQ873432                    ---------- ---------- ---------- ---------- ---------- ---------- ---------- ---------- ---------- ---------- ----------
AADN03001774                ---------- ---------- ---------- ---------- ---------- ---------- ---------- ---------- ---------- ---------- ----------
AADN03001775                ---------- ---------- ---------- ---------- ---------- ---------- ---------- ---------- ---------- ---------- ----------
EF552813                    ---------- ---------- ---------- ---------- ---------- ---------- ---------- ---------- ---------- ---------- ----------
AADN03022685                ---------- ---------- ---------- ---------- ---------- ---------- ---------- ---------- ---------- ---------- ----------
AADN03019346                ---------- ---------- ---------- ---------- ---------- ---------- ---------- ---------- ---------- ---------- ----------
AADN03001776                ---------- ---------- ---------- ---------- ---------- ---------- ---------- ---------- ---------- ---------- ----------
DQ018756                    ---------- ---------- ---------- ---------- ---------- ---------- ---------- ---------- ---------- ---------- ----------
AF173612                    ---------- ---------- ---------- ---------- ---------- ---------- ---------- ---------- ---------- ---------- ----------
Gallus_gallus_KT445934.2    ACTCGTCTGT CCGCCCGCCC TGTCGGTGCC CCAGGGCTCG CCCGACCGAA TCCAGCTGTG TGACGGCCGA GCGGCCCCGC GAGCCGCAGG CGTACCCATT TCGTTGTGAG

                            1111111111 1111111111 1111111111 1111111111 1111111111 1111111111 1111111111 1111111111 1111111111 1111111111 1111111111
                            1111111111 1111111111 1111111111 1111111111 1111111111 1111111111 1111111111 1111111111 1111111111 1111111112 2222222222
                            0000000001 1111111112 2222222223 3333333334 4444444445 5555555556 6666666667 7777777778 8888888889 9999999990 0000000001
                            1234567890 1234567890 1234567890 1234567890 1234567890 1234567890 1234567890 1234567890 1234567890 1234567890 1234567890
AADN03001677                ---------- ---------- ---------- ---------- ---------- ---------- ---------- ---------- ---------- ---------- ----------
AADN03001778                ---------- ---------- ---------- ---------- ---------- ---------- ---------- ---------- ---------- ---------- ----------
AADN03001785                ---------- ---------- ---------- ---------- ---------- ---------- ---------- ---------- ---------- ---------- ----------
AADN03001786                ---------- ---------- ---------- ---------- ---------- ---------- ---------- ---------- ---------- ---------- ----------
AADN03001788                ---------- ---------- ---------- ---------- ---------- ---------- ---------- ---------- ---------- ---------- ----------
AADN03014081                ---------- ---------- ---------- ---------- ---------- ---------- ---------- ---------- ---------- ---------- ----------
AADN03015064                ---------- ---------- ---------- ---------- ---------- ---------- ---------- ---------- ---------- ---------- ----------
AADN03026634                ---------- ---------- ---------- ---------- ---------- ---------- ---------- ---------- ---------- ---------- ----------
NW_003775878                NNNNNNNNNN NNNNNNNNNN NNNNNNNNNN NNNNNNNNNN NNNNNNNNNN NNNNNNNNNN NNNNNNNNNN NNNNNNNNNN NNNNNNNNNN NNNNNNNNNN NNNNNNNNNN
DQ018752                    ---------- ---------- ---------- ---------- ---------- ---------- ---------- ---------- ---------- ---------- ----------
DQ018757                    ---------- ---------- ---------- ---------- ---------- ---------- ---------- ---------- ---------- ---------- ----------
DQ112354                    ---------- ---------- ---------- ---------- ---------- ---------- ---------- ---------- ---------- ---------- ----------
FM165414                    ---------- ---------- ---------- ---------- ---------- ---------- ---------- ---------- ---------- ---------- ----------
FM165415                    ---------- ---------- ---------- ---------- ---------- ---------- ---------- ---------- ---------- ---------- ----------
AADN03000430                ---------- ---------- ---------- ---------- ---------- ---------- ---------- ---------- ---------- ---------- ----------
AADN03001784                ---------- ---------- ---------- ---------- ---------- ---------- ---------- ---------- ---------- ---------- ----------
AADN03001783                ---------- ---------- ---------- ---------- ---------- ---------- ---------- ---------- ---------- ---------- ----------
DQ018754                    ---------- ---------- ---------- ---------- ---------- ---------- ---------- ---------- ---------- ---------- ----------
AADN03001782                ---------- ---------- ---------- ---------- ---------- ---------- ---------- ---------- ---------- ---------- ----------
JN639848                    ---------- ---------- ---------- ---------- ---------- ---------- ---------- ---------- ---------- ---------- ----------
AADN03001670                ---------- ---------- ---------- ---------- ---------- ---------- ---------- ---------- ---------- ---------- ----------
HQ873432                    ---------- ---------- ---------- ---------- ---------- ---------- ---------- ---------- ---------- ---------- ----------
AADN03001774                ---------- ---------- ---------- ---------- ---------- ---------- ---------- ---------- ---------- ---------- ----------
AADN03001775                ---------- ---------- ---------- ---------- ---------- ---------- ---------- ---------- ---------- ---------- ----------
EF552813                    ---------- ---------- ---------- ---------- ---------- ---------- ---------- ---------- ---------- ---------- ----------
```

```
AADN03022685                 ---------- ---------- ---------- ---------- ---------- ---------- ---------- ---------- ---------- ---------- ----------
AADN03019346                 ---------- ---------- ---------- ---------- ---------- ---------- ---------- ---------- ---------- ---------- ----------
AADN03001776                 ---------- ---------- ---------- ---------- ---------- ---------- ---------- ---------- ---------- ---------- ----------
DQ018756                     ---------- ---------- ---------- ---------- ---------- ---------- ---------- ---------- ---------- ---------- ----------
AF173612                     ---------- ---------- ---------- ---------- ---------- ---------- ---------- ---------- ---------- ---------- ----------
Gallus_gallus_KT445934.2     CGAGGCGTCG GCGCTGCCCT CGTTTCGGGG CCCGGCGAGT GCCGGCCGCG AGCAGCAAGC CGGCGGGGTG GCAACCGAGG GAAACCGCGG GGAACCGAGG CGAAGCGAGC

                             1111111111 1111111111 1111111111 1111111111 1111111111 1111111111 1111111111 1111111111 1111111111 1111111111 1111111111
                             2222222222 2222222222 2222222222 2222222222 2222222222 2222222222 2222222222 2222222222 2222222223 3333333333 3333333333
                             1111111112 2222222223 3333333334 4444444445 5555555556 6666666667 7777777778 8888888889 9999999990 0000000001 1111111112
                             1234567890 1234567890 1234567890 1234567890 1234567890 1234567890 1234567890 1234567890 1234567890 1234567890 1234567890
AADN03001677                 ---------- ---------- ---------- ---------- ---------- ---------- ---------- ---------- ---------- ---------- ----------
AADN03001778                 ---------- ---------- ---------- ---------- ---------- ---------- ---------- ---------- ---------- ---------- ----------
AADN03001785                 ---------- ---------- ---------- ---------- ---------- ---------- ---------- ---------- ---------- ---------- ----------
AADN03001786                 ---------- ---------- ---------- ---------- ---------- ---------- ---------- ---------- ---------- ---------- ----------
AADN03001788                 ---------- ---------- ---------- ---------- ---------- ---------- ---------- ---------- ---------- ---------- ----------
AADN03014081                 ---------- ---------- ---------- ---------- ---------- ---------- ---------- ---------- ---------- ---------- ----------
AADN03015064                 ---------- ---------- ---------- ---------- ---------- ---------- ---------- ---------- ---------- ---------- ----------
AADN03026634                 ---------- ---------- ---------- ---------- ---------- ---------- ---------- ---------- ---------- ---------- ----------
NW_003775878                 NNNNNNNNNN NNNNNNNNNN NNNNNNNNNN NNNNNNNNNN NNNNNNNNNN NNNNNNNNNN NNNNNNNNNN NNNNNNNNNN NNNNNNNNNN NNNNNNNNNN NNNNNNNNNN
DQ018752                     ---------- ---------- ---------- ---------- ---------- ---------- ---------- ---------- ---------- ---------- ----------
DQ018757                     ---------- ---------- ---------- ---------- ---------- ---------- ---------- ---------- ---------- ---------- ----------
DQ112354                     ---------- ---------- ---------- ---------- ---------- ---------- ---------- ---------- ---------- ---------- ----------
FM165414                     ---------- ---------- ---------- ---------- ---------- ---------- ---------- ---------- ---------- ---------- ----------
FM165415                     ---------- ---------- ---------- ---------- ---------- ---------- ---------- ---------- ---------- ---------- ----------
AADN03000430                 ---------- ---------- ---------- ---------- ---------- ---------- ---------- ---------- ---------- ---------- ----------
AADN03001784                 ---------- ---------- ---------- ---------- ---------- ---------- ---------- ---------- ---------- ---------- ----------
AADN03001783                 ---------- ---------- ---------- ---------- ---------- ---------- ---------- ---------- ---------- ---------- ----------
DQ018754                     ---------- ---------- ---------- ---------- ---------- ---------- ---------- ---------- ---------- ---------- ----------
AADN03001782                 ---------- ---------- ---------- ---------- ---------- ---------- ---------- ---------- ---------- ---------- ----------
JN639848                     ---------- ---------- ---------- ---------- ---------- ---------- ---------- ---------- ---------- ---------- ----------
AADN03001670                 ---------- ---------- ---------- ---------- ---------- ---------- ---------- ---------- ---------- ---------- ----------
HQ873432                     ---------- ---------- ---------- ---------- ---------- ---------- ---------- ---------- ---------- ---------- ----------
AADN03001774                 ---------- ---------- ---------- ---------- ---------- ---------- ---------- ---------- ---------- ---------- ----------
AADN03001775                 ---------- ---------- ---------- ---------- ---------- ---------- ---------- ---------- ---------- ---------- ----------
EF552813                     ---------- ---------- ---------- ---------- ---------- ---------- ---------- ---------- ---------- ---------- ----------
AADN03022685                 ---------- ---------- ---------- ---------- ---------- ---------- ---------- ---------- ---------- ---------- ----------
AADN03019346                 ---------- ---------- ---------- ---------- ---------- ---------- ---------- ---------- ---------- ---------- ----------
AADN03001776                 ---------- ---------- ---------- ---------- ---------- ---------- ---------- ---------- ---------- ---------- ----------
DQ018756                     ---------- ---------- ---------- ---------- ---------- ---------- ---------- ---------- ---------- ---------- ----------
AF173612                     ---------- ---------- ---------- ---------- ---------- ---------- ---------- ---------- ---------- ---------- ----------
Gallus_gallus_KT445934.2     AGCAGCAGAA GAAGGAACGA GAAGACAACG GGGGGCTGCG CCCGGCCGAG CGGGCGAGCC CGGAGCAGCG CGGCGCGTCC CGCTCCGGAT CCGTCGGGGT GTGGGGGCCG

                             1111111111 1111111111 1111111111 1111111111 1111111111 1111111111 1111111111 1111111111 1111111111 1111111111 1111111111
                             3333333333 3333333333 3333333333 3333333333 3333333333 3333333333 3333333333 3333333334 4444444444 4444444444 4444444444
                             2222222223 3333333334 4444444445 5555555556 6666666667 7777777778 8888888889 9999999990 0000000001 1111111112 2222222223
                             1234567890 1234567890 1234567890 1234567890 1234567890 1234567890 1234567890 1234567890 1234567890 1234567890 1234567890
AADN03001677                 ---------- ---------- ---------- ---------- ---------- ---------- ---------- ---------- ---------- ---------- ----------
AADN03001778                 ---------- ---------- ---------- ---------- ---------- ---------- ---------- ---------- ---------- ---------- ----------
AADN03001785                 ---------- ---------- ---------- ---------- ---------- ---------- ---------- ---------- ---------- ---------- ----------
AADN03001786                 ---------- ---------- ---------- ---------- ---------- ---------- ---------- ---------- ---------- ---------- ----------
AADN03001788                 ---------- ---------- ---------- ---------- ---------- ---------- ---------- ---------- ---------- ---------- ----------
AADN03014081                 ---------- ---------- ---------- ---------- ---------- ---------- ---------- ---------- ---------- ---------- ----------
AADN03015064                 ---------- ---------- ---------- ---------- ---------- ---------- ---------- ---------- ---------- ---------- ----------
AADN03026634                 ---------- ---------- ---------- ---------- ---------- ---------- ---------- ---------- ---------- ---------- ----------
```

```
NW_003775878                NNNNNNNNNN NNNNNNNNNN NNNNNNNNNN NNNNNNNNNN NNNNNNNNNN NNNNNNNNNN NNNNNNNNNN NNNNNNNNNN NNNNNNNNNN NNNNNNNNNN NNNNNNNNNN
DQ018752                    ---------- ---------- ---------- ---------- ---------- ---------- ---------- ---------- ---------- ---------- ----------
DQ018757                    ---------- ---------- ---------- ---------- ---------- ---------- ---------- ---------- ---------- ---------- ----------
DQ112354                    ---------- ---------- ---------- ---------- ---------- ---------- ---------- ---------- ---------- ---------- ----------
FM165414                    ---------- ---------- ---------- ---------- ---------- ---------- ---------- ---------- ---------- ---------- ----------
FM165415                    ---------- ---------- ---------- ---------- ---------- ---------- ---------- ---------- ---------- ---------- ----------
AADN03000430                ---------- ---------- ---------- ---------- ---------- ---------- ---------- ---------- ---------- ---------- ----------
AADN03001784                ---------- ---------- ---------- ---------- ---------- ---------- ---------- ---------- ---------- ---------- ----------
AADN03001783                ---------- ---------- ---------- ---------- ---------- ---------- ---------- ---------- ---------- ---------- ----------
DQ018754                    ---------- ---------- ---------- ---------- ---------- ---------- ---------- ---------- ---------- ---------- ----------
AADN03001782                ---------- ---------- ---------- ---------- ---------- ---------- ---------- ---------- ---------- ---------- ----------
JN639848                    ---------- ---------- ---------- ---------- ---------- ---------- ---------- ---------- ---------- ---------- ----------
AADN03001670                ---------- ---------- ---------- ---------- ---------- ---------- ---------- ---------- ---------- ---------- ----------
HQ873432                    ---------- ---------- ---------- ---------- ---------- ---------- ---------- ---------- ---------- ---------- ----------
AADN03001774                ---------- ---------- ---------- ---------- ---------- ---------- ---------- ---------- ---------- ---------- ----------
AADN03001775                ---------- ---------- ---------- ---------- ---------- ---------- ---------- ---------- ---------- ---------- ----------
EF552813                    ---------- ---------- ---------- ---------- ---------- ---------- ---------- ---------- ---------- ---------- ----------
AADN03022685                ---------- ---------- ---------- ---------- ---------- ---------- ---------- ---------- ---------- ---------- ----------
AADN03019346                ---------- ---------- ---------- ---------- ---------- ---------- ---------- ---------- ---------- ---------- ----------
AADN03001776                ---------- ---------- ---------- ---------- ---------- ---------- ---------- ---------- ---------- ---------- ----------
DQ018756                    ---------- ---------- ---------- ---------- ---------- ---------- ---------- ---------- ---------- ---------- ----------
AF173612                    ---------- ---------- ---------- ---------- ---------- ---------- ---------- ---------- ---------- ---------- ----------
Gallus_gallus_KT445934.2    GGGGCGTCCG CCGGCCTCTC CTCCGCCTTC GGGCCGCCGC AGCCCGTGTC GGTTTTCCTG CCGCGTCCCC GCCCGCTGCG GAGCGTGCCG CCCCGGGAAA GGGTCTCCGA

                            1111111111 1111111111 1111111111 1111111111 1111111111 1111111111 1111111111 1111111111 1111111111 1111111111 1111111111
                            4444444444 4444444444 4444444444 4444444444 4444444444 4444444444 4444444445 5555555555 5555555555 5555555555 5555555555
                            3333333334 4444444445 5555555556 6666666667 7777777778 8888888889 9999999990 0000000001 1111111112 2222222223 3333333334
                            1234567890 1234567890 1234567890 1234567890 1234567890 1234567890 1234567890 1234567890 1234567890 1234567890 1234567890
AADN03001677                ---------- ---------- ---------- ---------- ---------- ---------- ---------- ---------- ---------- ---------- ----------
AADN03001778                ---------- ---------- ---------- ---------- ---------- ---------- ---------- ---------- ---------- ---------- ----------
AADN03001785                ---------- ---------- ---------- ---------- ---------- ---------- ---------- ---------- ---------- ---------- ----------
AADN03001786                ---------- ---------- ---------- ---------- ---------- ---------- ---------- ---------- ---------- ---------- ----------
AADN03001788                ---------- ---------- ---------- ---------- ---------- ---------- ---------- ---------- ---------- ---------- ----------
AADN03014081                ---------- ---------- ---------- ---------- ---------- ---------- ---------- ---------- ---------- ---------- ----------
AADN03015064                ---------- ---------- ---------- ---------- ---------- ---------- ---------- ---------- ---------- ---------- ----------
AADN03026634                ---------- ---------- ---------- ---------- ---------- ---------- ---------- ---------- ---------- ---------- ----------
NW_003775878                NNNNNNNNNN NNNNNNNNNN NNNNNNNNNN NNNNNNNNNN NNNNNNNNNN NNNNNNNNNN NNNNNNNNNN NNNNNNNNNN NNNNNNNNNN NNNNNNNNNN NNNNNNNNNN
DQ018752                    ---------- ---------- ---------- ---------- ---------- ---------- ---------- ---------- ---------- ---------- ----------
DQ018757                    ---------- ---------- ---------- ---------- ---------- ---------- ---------- ---------- ---------- ---------- ----------
DQ112354                    ---------- ---------- ---------- ---------- ---------- ---------- ---------- ---------- ---------- ---------- ----------
FM165414                    ---------- ---------- ---------- ---------- ---------- ---------- ---------- ---------- ---------- ---------- ----------
FM165415                    ---------- ---------- ---------- ---------- ---------- ---------- ---------- ---------- ---------- ---------- ----------
AADN03000430                ---------- ---------- ---------- ---------- ---------- ---------- ---------- ---------- ---------- ---------- ----------
AADN03001784                ---------- ---------- ---------- ---------- ---------- ---------- ---------- ---------- ---------- ---------- ----------
AADN03001783                ---------- ---------- ---------- ---------- ---------- ---------- ---------- ---------- ---------- ---------- ----------
DQ018754                    ---------- ---------- ---------- ---------- ---------- ---------- ---------- ---------- ---------- ---------- ----------
AADN03001782                ---------- ---------- ---------- ---------- ---------- ---------- ---------- ---------- ---------- ---------- ----------
JN639848                    ---------- ---------- ---------- ---------- ---------- ---------- ---------- ---------- ---------- ---------- ----------
AADN03001670                ---------- ---------- ---------- ---------- ---------- ---------- ---------- ---------- ---------- ---------- ----------
HQ873432                    ---------- ---------- ---------- ---------- ---------- ---------- ---------- ---------- ---------- ---------- ----------
AADN03001774                ---------- ---------- ---------- ---------- ---------- ---------- ---------- ---------- ---------- ---------- ----------
AADN03001775                ---------- ---------- ---------- ---------- ---------- ---------- ---------- ---------- ---------- ---------- ----------
EF552813                    ---------- ---------- ---------- ---------- ---------- ---------- ---------- ---------- ---------- ---------- ----------
AADN03022685                ---------- ---------- ---------- ---------- ---------- ---------- ---------- ---------- ---------- ---------- ----------
AADN03019346                ---------- ---------- ---------- ---------- ---------- ---------- ---------- ---------- ---------- ---------- ----------
```

```
AADN03001776                    ---------- ---------- ---------- ---------- ---------- ---------- ---------- ---------- ---------- ---------- ----------
DQ018756                        ---------- ---------- ---------- ---------- ---------- ---------- ---------- ---------- ---------- ---------- ----------
AF173612                        ---------- ---------- ---------- ---------- ---------- ---------- ---------- ---------- ---------- ---------- ----------
Gallus_gallus_KT445934.2        TCGTGGGGTC GCGCCCGTCT CGAGGTCGCG TTCTCCTCTA GCACGTCCGT CTCCGGCGGC GGGGCTTCGT TTCCCCGTCC GCTTCTCCGC CGGTCCCGGA GGGCGGGTCA

                                1111111111 1111111111 1111111111 1111111111 1111111111 1111111111 1111111111 1111111111 1111111111 1111111111 1111111111
                                5555555555 5555555555 5555555555 5555555555 5555555555 5555555556 6666666666 6666666666 6666666666 6666666666 6666666666
                                4444444445 5555555556 6666666667 7777777778 8888888889 9999999990 0000000001 1111111112 2222222223 3333333334 4444444445
                                1234567890 1234567890 1234567890 1234567890 1234567890 1234567890 1234567890 1234567890 1234567890 1234567890 1234567890
AADN03001677                    ---------- ---------- ---------- ---------- ---------- ---------- ---------- ---------- ---------- ---------- ----------
AADN03001778                    ---------- ---------- ---------- ---------- ---------- ---------- ---------- ---------- ---------- ---------- ----------
AADN03001785                    ---------- ---------- ---------- ---------- ---------- ---------- ---------- ---------- ---------- ---------- ----------
AADN03001786                    ---------- ---------- ---------- ---------- ---------- ---------- ---------- ---------- ---------- ---------- ----------
AADN03001788                    ---------- ---------- ---------- ---------- ---------- ---------- ---------- ---------- ---------- ---------- ----------
AADN03014081                    ---------- ---------- ---------- ---------- ---------- ---------- ---------- ---------- ---------- ---------- ----------
AADN03015064                    ---------- ---------- ---------- ---------- ---------- ---------- ---------- ---------- ---------- ---------- ----------
AADN03026634                    ---------- ---------- ---------- ---------- ---------- ---------- ---------- ---------- ---------- ---------- ----------
NW_003775878                    NNNNNNNNNN NNNNNNNNNN NNNNNNNNNN NNNNNNNNNN NNNNNNNNNN NNNNNNNNNN NNNNNNNNNN NNNNNNNNNN NNNNNNNNNN NNNNNNNNNN NNNNNNNNNN
DQ018752                        ---------- ---------- ---------- ---------- ---------- ---------- ---------- ---------- ---------- ---------- ----------
DQ018757                        ---------- ---------- ---------- ---------- ---------- ---------- ---------- ---------- ---------- ---------- ----------
DQ112354                        ---------- ---------- ---------- ---------- ---------- ---------- ---------- ---------- ---------- ---------- ----------
FM165414                        ---------- ---------- ---------- ---------- ---------- ---------- ---------- ---------- ---------- ---------- ----------
FM165415                        ---------- ---------- ---------- ---------- ---------- ---------- ---------- ---------- ---------- ---------- ----------
AADN03000430                    ---------- ---------- ---------- ---------- ---------- ---------- ---------- ---------- ---------- ---------- ----------
AADN03001784                    ---------- ---------- ---------- ---------- ---------- ---------- ---------- ---------- ---------- ---------- ----------
AADN03001783                    ---------- ---------- ---------- ---------- ---------- ---------- ---------- ---------- ---------- ---------- ----------
DQ018754                        ---------- ---------- ---------- ---------- ---------- ---------- ---------- ---------- ---------- ---------- ----------
AADN03001782                    ---------- ---------- ---------- ---------- ---------- ---------- ---------- ---------- ---------- ---------- ----------
JN639848                        ---------- ---------- ---------- ---------- ---------- ---------- ---------- ---------- ---------- ---------- ----------
AADN03001670                    ---------- ---------- ---------- ---------- ---------- ---------- ---------- ---------- ---------- ---------- ----------
HQ873432                        ---------- ---------- ---------- ---------- ---------- ---------- ---------- ---------- ---------- ---------- ----------
AADN03001774                    ---------- ---------- ---------- ---------- ---------- ---------- ---------- ---------- ---------- ---------- ----------
AADN03001775                    ---------- ---------- ---------- ---------- ---------- ---------- ---------- ---------- ---------- ---------- ----------
EF552813                        ---------- ---------- ---------- ---------- ---------- ---------- ---------- ---------- ---------- ---------- ----------
AADN03022685                    ---------- ---------- ---------- ---------- ---------- ---------- ---------- ---------- ---------- ---------- ----------
AADN03019346                    ---------- ---------- ---------- ---------- ---------- ---------- ---------- ---------- ---------- ---------- ----------
AADN03001776                    ---------- ---------- ---------- ---------- ---------- ---------- ---------- ---------- ---------- ---------- ----------
DQ018756                        ---------- ---------- ---------- ---------- ---------- ---------- ---------- ---------- ---------- ---------- ----------
AF173612                        ---------- ---------- ---------- ---------- ---------- ---------- ---------- ---------- ---------- ---------- ----------
Gallus_gallus_KT445934.2        GCCCCGGCCG GCCGTGCGGC GCGAGCGCGA GTCCGGCTCC CGCGGGGGGG GCCCGGAGCG TGCCGCCGAA AGCAGCTGCG CAGCGGTCCC CGCTCCTTCC CCGCGGGGGG

                                1111111111 1111111111 1111111111 1111111111 1111111111 1111111111 1111111111 1111111111 1111111111 1111111111 1111111111
                                6666666666 6666666666 6666666666 6666666666 6666666667 7777777777 7777777777 7777777777 7777777777 7777777777 7777777777
                                5555555556 6666666667 7777777778 8888888889 9999999990 0000000001 1111111112 2222222223 3333333334 4444444445 5555555556
                                1234567890 1234567890 1234567890 1234567890 1234567890 1234567890 1234567890 1234567890 1234567890 1234567890 1234567890
AADN03001677                    ---------- ---------- ---------- ---------- ---------- ---------- ---------- ---------- ---------- ---------- ----------
AADN03001778                    ---------- ---------- ---------- ---------- ---------- ---------- ---------- ---------- ---------- ---------- ----------
AADN03001785                    ---------- ---------- ---------- ---------- ---------- ---------- ---------- ---------- ---------- -CGCCCCGCC GAGAGGCGTT
AADN03001786                    ---------- ---------- ---------- -------CTC CGTCGTGGTC GGCGAGCGAG CGAGCGAGCG AGGGAACGAC GGAGGGCCGC CCGCCCCGCC GAGAGGCGTT
AADN03001788                    ---------- ---------- ---------- ---------- ---------- ---------- ---------- ---------- ---------- --GCCCCGCC GAGAGGCGTT
AADN03014081                    ---------- ---------- ---------- ---------- ---------- ---------- ---------- ---------- ---------- ---------- ----------
AADN03015064                    ---------- ---------- ---------- ---------- ---------- ---------- ---------- ---------- ---------- ---------- ----------
AADN03026634                    ---------- ---------- ---------- ---------- ---------- ---------- ---------- ---------- ---------- ---------- ----------
NW_003775878                    NNNNNNNNNN NNNNNNNNNN NNNNNNNNNN NNNNNNNNNN NNNNNNNNNN NNNNNNNNNN NNNNNNNNNN NNNNNNNNNN NNNNNNNNNN NNNNNNNNNN NNNNNNNNNN
DQ018752                        ---------- ---------- ---------- ---------- ---------- ---------- ---------- ---------- ---------- ---------- ----------
```

```
DQ018757                       ---------- ---------- ---------- ---------- ---------- ---------- ---------- ---------- ---------- ---------- ----------
DQ112354                       ---------- ---------- ---------- ---------- ---------- ---------- ---------- ---------- ---------- ---------- ----------
FM165414                       ---------- ---------- ---------- ---------- ---------- ---------- ---------- ---------- ---------- ---------- ----------
FM165415                       ---------- ---------- ---------- ---------- ---------- ---------- ---------- ---------- ---------- ---------- ----------
AADN03000430                   ---------- ---------- ---------- ---------- ---------- ---------- ---------- ---------- ---------- ---------- ----------
AADN03001784                   ---------- ---------- ---------- ---------- ---------- ---------- ---------- ---------- ---------- ---------- ----------
AADN03001783                   ---------- ---------- ---------- ---------- ---------- ---------- ---------- ---------- ---------- ---------- ----------
DQ018754                       ---------- ---------- ---------- ---------- ---------- ---------- ---------- ---------- ---------- ---------- ----------
AADN03001782                   ---------- ---------- ---------- ---------- ---------- ---------- ---------- ---------- ---------- ---------- ----------
JN639848                       ---------- ---------- ---------- ---------- ---------- ---------- ---------- ---------- ---------- ---------- ----------
AADN03001670                   ---------- ---------- ---------- ---------- ---------- ---------- ---------- ---------- ---------- ---------- ----------
HQ873432                       ---------- ---------- ---------- ---------- ---------- ---------- ---------- ---------- ---------- ---------- ----------
AADN03001774                   ---------- ---------- ---------- ---------- ---------- ---------- ---------- ---------- ---------- ---------- ----------
AADN03001775                   ---------- ---------- ---------- ---------- ---------- ---------- ---------- ---------- ---------- ---------- ----------
EF552813                       ---------- ---------- ---------- ---------- ---------- ---------- ---------- ---------- ---------- ---------- ----------
AADN03022685                   ---------- ---------- ---------- ---------- ---------- ---------- ---------- ---------- ---------- ---------- ----------
AADN03019346                   ---------- ---------- ---------- ---------- ---------- ---------- ---------- ---------- ---------- ---------- ----------
AADN03001776                   ---------- ---------- ---------- ---------- ---------- ---------- ---------- ---------- ---------- ---------- ----------
DQ018756                       ---------- ---------- ---------- ---------- ---------- ---------- ---------- ---------- ---------- ---------- ----------
AF173612                       ---------- ---------- ---------- ---------- ---------- ---------- ---------- ---------- ---------- ---------- ----------
Gallus_gallus_KT445934.2       GAGGTCGGCG GGGCCGCCCC GGGGATCGGG CGCGCCTCTC CGTCGTGGTC GGCGAGCGAG CGAGCGAGCG AGGGAACGAC GGAGGGCCGC CCGCCCCGCC GAGAGGCGTT

                               1111111111 1111111111 1111111111 1111111111 1111111111 1111111111 1111111111 1111111111 1111111111 1111111111 1111111111
                               7777777777 7777777777 7777777777 7777777778 8888888888 8888888888 8888888888 8888888888 8888888888 8888888888 8888888888
                               6666666667 7777777778 8888888889 9999999990 0000000001 1111111112 2222222223 3333333334 4444444445 5555555556 6666666667
                               1234567890 1234567890 1234567890 1234567890 1234567890 1234567890 1234567890 1234567890 1234567890 1234567890 1234567890
AADN03001677                   ---------- ---------- ---------- ---------- ---------- ---------- ---------- ---------- ---------- ---------- ----------
AADN03001778                   ---------- ---------- ---------- ---------- ---------- ---------- ---------- ---------- ---------- ---------- ----------
AADN03001785                   CGCCCCGGCG GCCGCCGCCG TCGACCCGGC AAGGGCCAGA CGGGAAAGCC GAGCGAGCAG GCGAGAGAGA GAGAGAGGGA AGGAGCGAGA GCGGTCGGCG GCGGGCCGGG
AADN03001786                   CGCCCCGGCG GCCGCCGCCG TCGACCCGGC AAGGGCCAGA CGGGAAAGCC GAGCGAGCAG GCG--AGAGA GAGAGAGGGA AGGAGCGAGA GCGGTCGGCG GCGGGCCGGG
AADN03001788                   CGCCCCGGCG GCCGCCGCCG TCGACCCGGC AAGGGCCAGA CGGGAAAGCC GAGCGAGCAG GCG--AGAGA GAGAGAGGGA AGGAGCGAGA GCGGTCGGCG GCGGGCCGGG
AADN03014081                   ---------- ---------- ---------- ---------- ---------- ---------- ---------- ---------- ---------- ---------- ----------
AADN03015064                   ---------- ---------- ---------- ---------- ---------- ---------- ---------- ---------- ---------- ---------- ----------
AADN03026634                   ---------- ---------- ---------- ---------- ---------- ---------- ---------- ---------- ---------- ---------- ----------
NW_003775878                   NNNNNNNNNN NNNNNNNNNN NNNNNNNNNN NNNNNNNNNN NNNNNNNNNN NNNNNNNNNN NNNNNNNNNN NNNNNNNNNN NNNNNNNNNN NNNNNNNNNN NNNNNNNNNN
DQ018752                       ---------- ---------- ---------- ---------- ---------- ---------- ---------- ---------- ---------- ---------- ----------
DQ018757                       ---------- ---------- ---------- ---------- ---------- ---------- ---------- ---------- ---------- ---------- ----------
DQ112354                       ---------- ---------- ---------- ---------- ---------- ---------- ---------- ---------- ---------- ---------- ----------
FM165414                       ---------- ---------- ---------- ---------- ---------- ---------- ---------- ---------- ---------- ---------- ----------
FM165415                       ---------- ---------- ---------- ---------- ---------- ---------- ---------- ---------- ---------- ---------- ----------
AADN03000430                   ---------- ---------- ---------- ---------- ---------- ---------- ---------- ---------- ---------- ---------- ----------
AADN03001784                   ---------- ---------- ---------- ---------- ---------- ---------- ---------- ---------- ---------- ---------- ----------
AADN03001783                   ---------- ---------- ---------- ---------- ---------- ---------- ---------- ---------- ---------- ---------- ----------
DQ018754                       ---------- ---------- ---------- ---------- ---------- ---------- ---------- ---------- ---------- ---------- ----------
AADN03001782                   ---------- ---------- ---------- ---------- ---------- ---------- ---------- ---------- ---------- ---------- ----------
JN639848                       ---------- ---------- ---------- ---------- ---------- ---------- ---------- ---------- ---------- ---------- ----------
AADN03001670                   ---------- ---------- ---------- ---------- ---------- ---------- ---------- ---------- ---------- ---------- ----------
HQ873432                       ---------- ---------- ---------- ---------- ---------- ---------- ---------- ---------- ---------- ---------- ----------
AADN03001774                   ---------- ---------- ---------- ---------- ---------- ---------- ---------- ---------- ---------- ---------- ----------
AADN03001775                   ---------- ---------- ---------- ---------- ---------- ---------- ---------- ---------- ---------- ---------- ----------
EF552813                       ---------- ---------- ---------- ---------- ---------- ---------- ---------- ---------- ---------- ---------- ----------
AADN03022685                   ---------- ---------- ---------- ---------- ---------- ---------- ---------- ---------- ---------- ---------- ----------
AADN03019346                   ---------- ---------- ---------- ---------- ---------- ---------- ---------- ---------- ---------- ---------- ----------
AADN03001776                   ---------- ---------- ---------- ---------- ---------- ---------- ---------- ---------- ---------- ---------- ----------
DQ018756                       ---------- ---------- ---------- ---------- ---------- ---------- ---------- ---------- ---------- ---------- ----------
```

```
AF173612                      ---------- ---------- ---------- ---------- ---------- ---------- ---------- ---------- ---------- ---------- ----------
Gallus_gallus_KT445934.2      CGCCCCGGCG GCCGCCGCCG TCGACCCGGC AAGGGCCAGA CGGGAAAGCC GAGCGAGCAG GCGAGAGAGA GAGAGAGGGA AGGAGCGAGA GCGGTCGGCG GCGGGCCGGG

                              1111111111 1111111111 1111111111 1111111111 1111111111 1111111111 1111111111 1111111111 1111111111 1111111111 1111111111
                              8888888888 8888888888 8888888889 9999999999 9999999999 9999999999 9999999999 9999999999 9999999999 9999999999 9999999999
                              7777777778 8888888889 9999999990 0000000001 1111111112 2222222223 3333333334 4444444445 5555555556 6666666667 7777777778
                              1234567890 1234567890 1234567890 1234567890 1234567890 1234567890 1234567890 1234567890 1234567890 1234567890 1234567890
AADN03001677                  ---------- ---------- ---------- ---------- ---------- ---------- ---------- ---------- ---------- ---------- ----------
AADN03001778                  ---------- ---------- ---------- ---------- ---------- ---------- ---------- ---------- ---------- ---------- ----------
AADN03001785                  CCCGTCGGGT CGTGCCCCGT GGCGCGGCTA CCTGGTTGAT CCTGCCAGTA GCATATGCTT GTCTCAAAGA TTAAGCCATG CATGTCTAAG TACACACGGG CGGTACAGTG
AADN03001786                  CCCGTCGGGT CGTGCCCCGT GGCGCGGCTA CCTGGTTGAT CCTGCCAGTA GCATATGCTT GTCTCAAAGA TTAAGCCATG CATGTCTAAG TACACACGGG CGGTACAGTG
AADN03001788                  CCCGTCGGGT CGTGCCCCGT GGCGCGGCTA CCTGGTTGAT CCTGCCAGTA GCATATGCTT GTCTCAAAGA TTAAGCCATG CATGTCTAAG TACACACGGG CGGTACAGTG
AADN03014081                  ---------- ---------- ---------- ---------- ---------- ---------- ---------- ---------- ---------- ---------- ----------
AADN03015064                  ---------- ---------- ---------- ---------- ---------- ---------- ---------- ---------- ---------- ---------- ----------
AADN03026634                  ---------- ---------- ---------- ---------- ---------- ---------- ---------- ---------- ---------- ---------- ----------
NW_003775878                  NNNNNNNNNN NNNNNNNNNN NNNNNNNNNN NNNNNNNNNN NNNNNNNNNN NNNNNNNNNN NNNNNNNNNN NNNNNNNNNN NNNNNNNNNN NNNNNNNNNN NNNNNNNNNN
DQ018752                      ---------- ---------- ---------- ---------- ---------- ---------- ---------- ---------- ---------- ---------- ----------
DQ018757                      ---------- ---------- ---------- ---------- ---------- ---------- ---------- ---------- ---------- ---------- ----------
DQ112354                      ---------- ---------- ---------- ---------- ---------- ---------- ---------- ---------- ---------- ---------- ----------
FM165414                      ---------- ---------- ---------- ---------T CCTGCCAGTA GCATATGCTT GTCTCAAAGA TTAAGCCATG CATGTCTAAG TACACACGGG CGGTACAGTG
FM165415                      ---------- ---------- ---------- ---------- ---------- ---------- ---------- ---------- ---------- ---------- ----------
AADN03000430                  ---------- ---------- ---------- ---------- ---------- ---------- ---------- ---------- ---------- ---------- ----------
AADN03001784                  ---------- ---------- ---------- ---------- ---------- ---------- ---------- ---------- ---------- ---------- ----------
AADN03001783                  ---------- ---------- ---------- ---------- ---------- ---------- ---------- ---------- ---------- ---------- ----------
DQ018754                      ---------- ---------- ---------- ---------- ---------- ---------- ---------- ---------- ---------- ---------- ----------
AADN03001782                  ---------- ---------- ---------- ---------- ---------- ---------- ---------- ---------- ---------- ---------- ----------
JN639848                      ---------- ---------- ---------- ---------- ---------- ---------- ---------- ---------- ---------- ---------- ----------
AADN03001670                  ---------- ---------- ---------- ---------- ---------- ---------- ---------- ---------- ---------- ---------- ----------
HQ873432                      ---------- ---------- ---------- ---------T CCTGCCAGTA GCATATGCTT GTCTCAAAGA TTAAGCCATG CATGTCTAAG TACACACGGG CGGTACAG--
AADN03001774                  ---------- ---------- ---------- ---------- ---------- ---------- ---------- ---------- ---------- ---------- ----------
AADN03001775                  ---------- ---------- ---------- ---------- ---------- ---------- ---------- ---------- ---------- ---------- ----------
EF552813                      ---------- ---------- ---------- ---------- ---------- ---------- ---------- ---------- ---------- ---------- ----------
AADN03022685                  ---------- ---------- ---------- ---------- ---------- ---------- ---------- ---------- ---------- ---------- ----------
AADN03019346                  ---------- ---------- ---------- ---------- ---------- ---------- ---------- ---------- ---------- ---------- ----------
AADN03001776                  ---------- ---------- ---------- ---------- ---------- ---------- ---------- ---------- ---------- ---------- ----------
DQ018756                      ---------- ---------- ---------- ---------- ---------- ---------- ---------- ---------- ---------- ---------- ----------
AF173612                      ---------- ---------- ---------- ---------- ---------- ---------- ---------A TTAAGCCATG CATGTCTAAG TACACACGGG CGGTACAGTG
Gallus_gallus_KT445934.2      CCCGTCGGGT CGTGCCCCGT GGCGCGGCTA CCTGGTTGAT CCTGCCAGTA GCATATGCTT GTCTCAAAGA TTAAGCCATG CATGTCTAAG TACACACGGG CGGTACAGTG

                              1111111111 1111111112 2222222222 2222222222 2222222222 2222222222 2222222222 2222222222 2222222222 2222222222 2222222222
                              9999999999 9999999990 0000000000 0000000000 0000000000 0000000000 0000000000 0000000000 0000000000 0000000000 0000000000
                              8888888889 9999999990 0000000001 1111111112 2222222223 3333333334 4444444445 5555555556 6666666667 7777777778 8888888889
                              1234567890 1234567890 1234567890 1234567890 1234567890 1234567890 1234567890 1234567890 1234567890 1234567890 1234567890
AADN03001677                  ---------- --------TA AATCAGTTAT GGTTCCTTTG GTCGCTCCCC TCCCGTTACT TGGATAACTG TGGTAATTCT AGAGCTAATA CATGCCGACG AGCGCCGACC
AADN03001778                  ---------- ---------- ---------- ---------- ---------- ---------- ---------- ---------- ---------- ---------- ----------
AADN03001785                  AAACTGCGAA TGGCTCATTA AATCAGTTAT GGTTCCTTTG GTCGCTCCCC TCCCGTTACT TGGATAACTG TGGTAATTCT AGAGCTAATA CATGCCGACG AGCGCCGACC
AADN03001786                  AAACTGCGAA TGGCTCATTA AATCAGTTAT GGTTCCTTTG GTCGCTCCCC TCCCGTTACT TGGATAACTG TGGTAATTCT AGAGCTAATA CATGCCGACG AGCGCCGACC
AADN03001788                  AAACTGCGAA TGGCTCATTA AATCAGTTAT GGTTCCTTTG GTCGCTCCCC TCCCGTTACT TGGATAACTG TGGTAATTCT AGAGCTAATA CATGCCGACG AGCGCCGACC
AADN03014081                  ---------- ---------- ---------- ---------- ---------- ---------- ---------- ---------- ---------- ---------- ----------
AADN03015064                  ---------- ---------- ---------- ---------- ---------- ---------- ---------- ---------- ---------- ---------- ----------
AADN03026634                  -------GAA TGGCTCATTA AATCAGTTAT GGTTCCTTTG GTCGCTCCCC TCCCGTTACT TGGATAACTG TGGTAATTCT AGAGGTAATA CATGCCGACG AGCGCCGACC
NW_003775878                  NNNNNNNGAA TGGCTCATTA AATCAGTTAT GGTTCCTTTG GTCGCTCCCC TCCCGTTACT TGGATAACTG TGGTAATTCT AGAGGTAATA CATGCCGACG AGCGCCGACC
DQ018752                      ---------- ---------- ---------- --TTCCTTTG GTCGCTCCCC TCCCGTTACT TGGATAACTG TGGTAATTCT AGAGCTAATA CATGCCGACG AGCGCCGACC
DQ018757                      ---------- ---------- ---------- ---------- ---------- ---------- ---------- ---------- ---------- ---------- ----------
DQ112354                      ---------- ---------- ---------- ---------- ---------- ---------- ---------- ---------- ---------- ---------- ----------
```

```
FM165414                      AAACTGCGAA TGGCTCATTA AATCAGTTAT GGTTCCTTTG GTCGCTCCCC TCCCGTTACT TGGATAACTG TGGTAATTCT AGAGCTAATA CATGCCGACG AGCGCCGACC
FM165415                      ---------- ---------- ---------- ---------- ---------- ---------- ---------- ---------- ---------- ---------- ----------
AADN03000430                  ---------- ---------- ---------- ---------- ---------- ---------- ---------- ---------- ---------- ---------- ----------
AADN03001784                  ---------- ---------- ---------- ---------- ---------- ---------- ---------- ---------- ---------- ---------- ----------
AADN03001783                  ---------- ---------- ---------- ---------- ---------- ---------- ---------- ---------- ---------- ---------- ----------
DQ018754                      ---------- ---------- ---------- ---------- ---------- ---------- ---------- ---------- ---------- ---------- ----------
AADN03001782                  ---------- ---------- ---------- ---------- ---------- ---------- ---------- ---------- ---------- ---------- ----------
JN639848                      ---------- ---------- ---------- ---------- ---------- ---------- ---------- ---------- ---------- ---------- ----------
AADN03001670                  ---------- ---------- ---------- ---------- ---------- ---------- ---------- ---------- ---------- ---------- ----------
HQ873432                      -GCGTGCGAA TGGCTCATTA AATCAGTTAT GGTTCCTTTG GTCGCTCCCC TCCCGT-GCG TGGATAACTG TGGTAATTCT AGAGCTAATA CATGCCGACG AGCGCCGACC
AADN03001774                  ---------- ---------- ---------- ---------- ---------- ---------- ---------- ---------- ---------- ---------- ----------
AADN03001775                  ---------- ---------- ---------- ---------- ---------- ---------- ---------- ---------- ---------- ---------- ----------
EF552813                      ---------- ---------- ---------- ---------- ---------- ---------- ---------- ---------- ---------- ---------- ----------
AADN03022685                  ---------- ---------- ---------- ---------- ---------- ---------- ---------- ---------- ---------- ---------- ----------
AADN03019346                  ---------- ---------- ---------- ---------- ---------- ---------- ---------- ---------- ---------- ---------- ----------
AADN03001776                  ---------- ---------- ---------- ---------- ---------- ---------- ---------- ---------- ---------- ---------- ----------
DQ018756                      ---------- ---------- ---------- ---------- ---------- ---------- ---------- ---------- ---------- ---------- ----------
AF173612                      AAACTGCGAA TGGCTCATTA AATCAGTTAT GGTTCCTTTG GTCGCTCCCC TCCCGTTACT TGGATAACTG TGGTAATTCT AGAGCTAATA CATGCCGACG AGCGCCGACC
Gallus_gallus_KT445934.2      AAACTGCGAA TGGCTCATTA AATCAGTTAT GGTTCCTTTG GTCGCTCCCC TCCCGTTACT TGGATAACTG TGGTAATTCT AGAGCTAATA CATGCCGACG AGCGCCGACC

                              2222222222 2222222222 2222222222 2222222222 2222222222 2222222222 2222222222 2222222222 2222222222 2222222222 2222222222
                              0000000001 1111111111 1111111111 1111111111 1111111111 1111111111 1111111111 1111111111 1111111111 1111111111 1111111112
                              9999999990 0000000001 1111111112 2222222223 3333333334 4444444445 5555555556 6666666667 7777777778 8888888889 9999999990
                              1234567890 1234567890 1234567890 1234567890 1234567890 1234567890 1234567890 1234567890 1234567890 1234567890 1234567890
AADN03001677                  TCCGGGGACG CGTGCATTTA TCAGACCAAA ACCAACCCGG GCTCGCCCGG CGGCTTTGGT GACTCTAGAT AACCTCGAGC CGATCGCACG CCCCCGTGGC GGCGACGACC
AADN03001778                  ---------- ---------- ---------- ---------- ---------- ---------- ---------- ---------- ---------- ---------- ----------
AADN03001785                  TCCGGGGACG CGTGCATTTA TCAGACCAAA ACCAACCCGG GCTCGCCCGG CGGCTTTGGT GACTCTAGAT AACCTCGAGC CGATCGCACG CCCCCGTGGC GGCGACGACC
AADN03001786                  TCCGGGGACG CGTGCATTTA TCAGACCAAA ACCAACCCGG GCTCGCCCGG CGGCTTTGGT GACTCTAGAT AACCTCGAGC CGATCGCACG CCCCCGTGGC GGCGACGACC
AADN03001788                  TCCGGGGACG CGTGCATTTA TCAGACCAAA ACCAACCCGG GCTCGCCCGG CGGCTTTGGT GACTCTAGAT AACCTCGAGC CGATCGCACG CCCCCGTGGC GGCGACGACC
AADN03014081                  ---------- ---------- ---------- ---------- ---------- ---------- ---------- ---------- ---------- ---------- ----------
AADN03015064                  ---------- ---------- ---------- ---------- ---------- ---------- ---------- ---------- ---------- ---------- ----------
AADN03026634                  TCCGGGGACG CGTGCATTTA TCAGACCAAA ACCAACCCGG GCTCGCCCGG CGGCTTTGGT GACTCTAGAT AACCTCGAGC CGATCGCACG CCCCCGTGGC GGCGACGACC
NW_003775878                  TCCGGGGACG CGTGCATTTA TCAGACCAAA ACCAACCCGG GCTCGCCCGG CGGCTTTGGT GACTCTAGAT AACCTCGAGC CGATCGCACG CCCCCGTGGC GGCGACGACC
DQ018752                      TCCGGGGACG CGTGCATTTA TCAGACCAAA ACCAACCCGG GCTCGCCCGG CGGCTTTGGT GACTCTAGAT AACCTCGAGC CGATCGCACG CCCCCGTGGC GGCGACGACC
DQ018757                      ---------- ---------- ---------- ---------- ---------- ---------- ---------- ---------- ---------- ---------- ----------
DQ112354                      ---------- ---------- ---------- ---------- ---------- ---------- ---------- ---------- ---------- ---------- ----------
FM165414                      TCCGGGGACG CGTGCATTTA TCAGACCAAA ACCAACCCGG GCTCGCCCGG CGGCTTTGGT GACTCTAGAT AACCTCGAGC CGATCGCACG CCCCCGTGGC GGCGACGACC
FM165415                      ---------- ---------- ---------- ---------- ---------- ---------- ---------- ---------- ---------- ---------- ----------
AADN03000430                  ---------- ---------- ---------- ---------- ---------- ---------- ---------- ---------- ---------- ---------- ----------
AADN03001784                  ---------- ---------- ---------- ---------- ---------- ---------- ---------- ---------- ---------- ---------- ----------
AADN03001783                  ---------- ---------- ---------- ---------- ---------- ---------- ---------- ---------- ---------- ---------- ----------
DQ018754                      ---------- ---------- ---------- ---------- ---------- ---------- ---------- ---------- ---------- ---------- ----------
AADN03001782                  ---------- ---------- ---------- ---------- ---------- ---------- ---------- ---------- ---------- ---------- ----------
JN639848                      ---------- ---------- ---------- ---------- ---------- ---------- ---------- ---------- ---------- ---------- ----------
AADN03001670                  ---------- ---------- ---------- ---------- ---------- ---------- ---------- ---------- ---------- ---------- ----------
HQ873432                      TCCGGGGACG CGTGCATTTA TCAGACCAAA ACCAACCCGG GCTCGCCCGG CGGCTTTGGT GACTCTAGAT AACCTCGAGC CGATCGCACG CCCCCGTGGC GGCGACGACC
AADN03001774                  ---------- ---------- ---------- ---------- ---------- ---------- ---------- ---------- ---------- ---------- ----------
AADN03001775                  ---------- ---------- ---------- ---------- ---------- ---------- ---------- ---------- ---------- ---------- ----------
EF552813                      ---------- ---------- ---------- ---------- ---------- ---------- ---------- ---------- ---------- ---------- ----------
AADN03022685                  ---------- ---------- ---------- ---------- ---------- ---------- ---------- ---------- ---------- ---------- ----------
AADN03019346                  ---------- ---------- ---------- ---------- ---------- ---------- ---------- ---------- ---------- ---------- ----------
AADN03001776                  ---------- ---------- ---------- ---------- ---------- ---------- ---------- ---------- ---------- ---------- ----------
DQ018756                      ---------- ---------- ---------- ---------- ---------- ---------- ---------- ---------- ---------- ---------- ----------
AF173612                      TCCGGGGACG CGTGCATTTA TCAGACCAAA ACCAACCCGG GCTCGCCCGG CGGCTTTGGT GACTCTAGAT AACCTCGAGC CGATCGCACG CCCCCGTGGC GGCGACGACC
Gallus_gallus_KT445934.2      TCCGGGGACG CGTGCATTTA TCAGACCAAA ACCAACCCGG GCTCGCCCGG CGGCTTTGGT GACTCTAGAT AACCTCGAGC CGATCGCACG CCCCCGTGGC GGCGACGACC
```

```
                         2222222222 2222222222 2222222222 2222222222 2222222222 2222222222 2222222222 2222222222 2222222222 2222222222 2222222222
                         2222222222 2222222222 2222222222 2222222222 2222222222 2222222222 2222222222 2222222222 2222222222 2222222223 3333333333
                         0000000001 1111111112 2222222223 3333333334 4444444445 5555555556 6666666667 7777777778 8888888889 9999999990 0000000001
                         1234567890 1234567890 1234567890 1234567890 1234567890 1234567890 1234567890 1234567890 1234567890 1234567890 1234567890
AADN03001677             CATTCGAATG TCTGCCCTAT CAACTTTCGA TGGTACTGTC TGTGCCTACC ATGGTGACCA CGGGTAACGG GGAATCAGGG TTCGATTCCG GAGAGGGAGC CTGAGAAACG
AADN03001778             ---------- ---------- ---------- ---------- ---------- ---------- ---------- ---------- ---------- ---------- ----------
AADN03001785             CATTCGAATG TCTGCCCTAT CAACTTTCGA TGGTACTGTC TGTGCCTACC ATGGTGACCA CGGGTAACGG GGAATCAGGG TTCGATTCCG GAGAGGGAGC CTGAGAAACG
AADN03001786             CATTCGAATG TCTGCCCTAT CAACTTTCGA TGGTACTGTC TGTGCCTACC ATGGTGACCA CGGGTAACGG GGAATCAGGG TTCGATTCCG GAGAGGGAGC CTGAGAAACG
AADN03001788             CATTCGAATG TCTGCCCTAT CAACTTTCGA TGGTACTGTC TGTGCCTACC ATGGTGACCA CGGGTAACGG GGAATCAGGG TTCGATTCCG GAGAGGGAGC CTGAGAAACG
AADN03014081             ---------- ---------- ---------- ---------- ---------- ---------- ---------- ---------- ---------- ---------- ----------
AADN03015064             ---------- ---------- ---------- ---------- ---------- ---------- ---------- ---------- ---------- ---------- ----------
AADN03026634             CATTCGAATG TCTGCCCTAT CAACTTTCGA TGGTACTGTC TGTGCCTACC ATGGTGACCA CGGGTAACGG GGAATCAGGG TTCGATTCCG GAGAGGGAGC CTGAGAAACG
NW_003775878             CATTCGAATG TCTGCCCTAT CAACTTTCGA TGGTACTGTC TGTGCCTACC ATGGTGACCA CGGGTAACGG GGAATCAGGG TTCGATTCCG GAGAGGGAGC CTGAGAAACG
DQ018752                 CATTCGAATG TCTGCCCTAT CAACTTTCGA TGGTACTGTC TGTGCCTACC ATGGTGACCA CGGGTAACGG GGAATCAGGG TTCGATTCCG GAGAGGGAGC CTGAGAAACG
DQ018757                 ---------- ---------- ---------- ---------- ---------- ---------- ---------- ---------- ---------- ---------- ----------
DQ112354                 ---------- ---------- ---------- ---------- ---------- ---------- ---------- ---------- ---------- ---------- ----------
FM165414                 CATTCGAATG TCTGCCCTAT CAACTTTCGA TGGTACTGTC TGTGCCTACC ATGGTGACCA CGGGTAACGG GGAATCAGGG TTCGATTCCG GAGAGGGAGC CTGAGAAACG
FM165415                 ---------- ---------- ---------- ---------- ---------- ---------- ---------- ---------- ---------- ---------- ----------
AADN03000430             ---------- ---------- ---------- ---------- ---------- ---------- ---------- ---------- ---------- ---------- ----------
AADN03001784             ---------- ---------- ---------- ---------- ---------- ---------- ---------- ---------- ---------- ---------- ----------
AADN03001783             ---------- ---------- ---------- ---------- ---------- ---------- ---------- ---------- ---------- ---------- ----------
DQ018754                 ---------- ---------- ---------- ---------- ---------- ---------- ---------- ---------- ---------- ---------- ----------
AADN03001782             ---------- ---------- ---------- ---------- ---------- ---------- ---------- ---------- ---------- ---------- ----------
JN639848                 ---------- ---------- ---------- ---------- ---------- ---------- ---------- ---------- ---------- ---------- ----------
AADN03001670             ---------- ---------- ---------- ---------- ---------- ---------- ---------- ---------- ---------- ---------- ----------
HQ873432                 CATTCGAATG TCTGCCCTAT CAACTTTCGA TGGTACTGTC TGTGCCTACC ATGGTGACCA CGGGTAACGG GGAATCAGGG TTCGATTCCG GAGAGGGAGC CTGAGAAACG
AADN03001774             ---------- ---------- ---------- ---------- ---------- ---------- ---------- ---------- ---------- ---------- ----------
AADN03001775             ---------- ---------- ---------- ---------- ---------- ---------- ---------- ---------- ---------- ---------- ----------
EF552813                 ---------- ---------- ---------- ---------- ---------- ---------- ---------- ---------- ---------- ---------- ----------
AADN03022685             ---------- ---------- ---------- ---------- ---------- ---------- ---------- ---------- ---------- ---------- ----------
AADN03019346             ---------- ---------- ---------- ---------- ---------- ---------- ---------- ---------- ---------- ---------- ----------
AADN03001776             ---------- ---------- ---------- ---------- ---------- ---------- ---------- ---------- ---------- ---------- ----------
DQ018756                 ---------- ---------- ---------- ---------- ---------- ---------- ---------- ---------- ---------- ---------- ----------
AF173612                 CATTCGAATG TCTGCCCTAT CAACTTTCGA TGGTACTGTC TGTGCCTACC ATGGTGACCA CGGGTAACGG GGAATCAGGG TTCGATTCCG GAGAGGGAGC CTGAGAAACG
Gallus_gallus_KT445934.2 CATTCGAATG TCTGCCCTAT CAACTTTCGA TGGTACTGTC TGTGCCTACC ATGGTGACCA CGGGTAACGG GGAATCAGGG TTCGATTCCG GAGAGGGAGC CTGAGAAACG

                         2222222222 2222222222 2222222222 2222222222 2222222222 2222222222 2222222222 2222222222 2222222222 2222222222 2222222222
                         3333333333 3333333333 3333333333 3333333333 3333333333 3333333333 3333333333 3333333333 3333333334 4444444444 4444444444
                         1111111112 2222222223 3333333334 4444444445 5555555556 6666666667 7777777778 8888888889 9999999990 0000000001 1111111112
                         1234567890 1234567890 1234567890 1234567890 1234567890 1234567890 1234567890 1234567890 1234567890 1234567890 1234567890
AADN03001677             GCTACCACAT CCAAGGAAGG CAGCAGGCGC GCAAATTACC CACTCCCGAC CCGGGGAGGT AGTGACGAAA AATAACAATA CAGGACTCTT TCGAGGCCCT GTAATTGGAA
AADN03001778             ---------- ---------- ---------- ---------- ---------- ---------- ---------- ---------- ---------- ---------- ----------
AADN03001785             GCTACCACAT CCAAGGAAGG CAGCAGGCGC GCAAATTACC CACTCCCGAC CCGGGGAGGT AGTGACGAAA AATAACAATA CAGGACTCTT -CGAGGCCCT GTAAT-GGAA
AADN03001786             GCTACCACAT CCAAGGAAGG CAGCAGGCGC GCAAATTACC CACTCCCGAC CCGGGGAGGT AGTGACGAAA AATAACAATA CAGGACTCTT ---------- ----------
AADN03001788             GCTACCACAT CCAAGGAAGG CAGCAGGCGC GCAAATTACC CACTCCCGAC CCGGGGAGGT AGTGACGAAA AATAACAATA CAGGACTCTT TCGAGGCCCT GTAATTGGAA
AADN03014081             ---------- ---------- ---------- ---------- ---------- ---------- ---------- ---------- ---------- ---------- ----------
AADN03015064             ---------- ---------- ---------- ---------- ---------- ---------- ---------- ---------- ---------- ---------- ----------
AADN03026634             GCTACCACAT CCAAGGAAGG CAGCAGGCGC GCAAATTACC CACTCCCGAC CCGGGGAGGT AGTGACGAAA AATAACAATA CAGGACTCTT TCGAGGCCCT GTAATTGGAA
NW_003775878             GCTACCACAT CCAAGGAAGG CAGCAGGCGC GCAAATTACC CACTCCCGAC CCGGGGAGGT AGTGACGAAA AATAACAATA CAGGACTCTT TCGAGGCCCT GTAATTGGAA
DQ018752                 GCTACCACAT CCAAGGAAGG CAGCAGGCGC GCAAATTACC CACTCCCGAC CCGGGGAGGT AGTGACGAAA AATAACAATA CAGGACTCTT TCGAGGCCCT GTAATTGGAA
DQ018757                 ---------- ---------- ---------- ---------- ---------- ---------- ---------- ---------- ---------- ---------- ----------
DQ112354                 ---------- ---------- ---------- ---------- ---------- ---------- ---------- ---------- ---------- ---------- ----------
FM165414                 GCTACCACAT CCAAGGAAGG CAGCAGGCGC GCAAATTACC CACTCCCGAC CCGGGGAGGT AGTGACGAAA AATAACAATA CAGGACTCTT TCGAGGCCCT GTAATTGGAA
FM165415                 ---------- ---------- ---------- ---------- ---------- ---------- ---------- ---------- ---------- ---------- ----------
```

```
AADN03000430                ---------- ---------- ---------- ---------- ---------- ---------- ---------- ---------- ---------- ---------- ----------
AADN03001784                ---------- ---------- ---------- ---------- ---------- ---------- ---------- ---------- ---------- ---------- ----------
AADN03001783                ---------- ---------- ---------- ---------- ---------- ---------- ---------- ---------- ---------- ---------- ----------
DQ018754                    ---------- ---------- ---------- ---------- ---------- ---------- ---------- ---------- ---------- ---------- ----------
AADN03001782                ---------- ---------- ---------- ---------- ---------- ---------- ---------- ---------- ---------- ---------- ----------
JN639848                    ---------- ---------- ---------- ---------- ---------- ---------- ---------- ---------- ---------- ---------- ----------
AADN03001670                ---------- ---------- ---------- ---------- ---------- ---------- ---------- ---------- ---------- ---------- ----------
HQ873432                    GCTACCACAT CCAAGGAAGG CAGCAGGCGC GCAAATTACC CACTCCCGAC CCGGGGAGGT AGTGACGAAA AATAACAATA CAGGACTCTT TCGAGGCCCT GTAATTGGAA
AADN03001774                ---------- ---------- ---------- ---------- ---------- ---------- ---------- ---------- ---------- ---------- ----------
AADN03001775                ---------- ---------- ---------- ---------- ---------- ---------- ---------- ---------- ---------- ---------- ----------
EF552813                    ---------- ---------- ---------- ---------- ---------- ---------- ---------- ---------- ---------- ---------- ----------
AADN03022685                ---------- ---------- ---------- ---------- ---------- ---------- ---------- ---------- ---------- ---------- ----------
AADN03019346                ---------- ---------- ---------- ---------- ---------- ---------- ---------- ---------- ---------- ---------- ----------
AADN03001776                ---------- ---------- ---------- ---------- ---------- ---------- ---------- ---------- ---------- ---------- ----------
DQ018756                    ---------- ---------- ---------- ---------- ---------- ---------- ---------- ---------- ---------- ---------- ----------
AF173612                    GCTACCACAT CCAAGGAAGG CAGCAGGCGC GCAAATTACC CACTCCCGAC CCGGGGAGGT AGTGACGAAA AATAACAATA CAGGACTCTT TCGAGGCCCT GTAATTGGAA
Gallus_gallus_KT445934.2    GCTACCACAT CCAAGGAAGG CAGCAGGCGC GCAAATTACC CACTCCCGAC CCGGGGAGGT AGTGACGAAA AATAACAATA CAGGACTCTT TCGAGGCCCT GTAATTGGAA

                            2222222222 2222222222 2222222222 2222222222 2222222222 2222222222 2222222222 2222222222 2222222222 2222222222 2222222222
                            4444444444 4444444444 4444444444 4444444444 4444444444 4444444444 4444444444 4444444445 5555555555 5555555555 5555555555
                            2222222223 3333333334 4444444445 5555555556 6666666667 7777777778 8888888889 9999999990 0000000001 1111111112 2222222223
                            1234567890 1234567890 1234567890 1234567890 1234567890 1234567890 1234567890 1234567890 1234567890 1234567890 1234567890
AADN03001677                TGAGTCCACT TTAAATCCTT TAACGAGGAT CCATTGGAGG GCAAGTCTGG TGCCAGCAGC CGCGGTAATT CCAGCTCCAA TAGCGTATAT TAAAGTTGCT GCAGTTAAAA
AADN03001778                ---------- ---------- ---------- ---------- ---------- ---------- ---------- ---------- ---------- ---------- ----------
AADN03001785                TGAGTCCACT TTAAATCCTT TAACGAGGAT CCATTGGAGG ---------- ---------- ---------- ---------- ---------- ---------- ----------
AADN03001786                ---------- ---------- ---------- ---------- ---------- ---------- ---------- ---------- ---------- ---------- ----------
AADN03001788                TGAGTCCACT TTAAATCCTT TAACGAGGAT CCATTGGAGG GCAAGTCTGG TGCCAGCAGC CGCGGTAAT- CCAGCTCCAA TA-------- ---------- ----------
AADN03014081                ---------- ---------- ---------- ---------- ---------- ---------- ---------- ---------- ---------- ---------- ----------
AADN03015064                ---------- ---------- ---------- ---------- ---------- ---------- ---------- ---------- ---------- ---------- ----------
AADN03026634                TGAGTCCACT TTAAATCCTT TAACGAGGAT CCATTGGAGG GCAAGTCTGG TGCCAGCAGC CGCGGTAATT CCAGCTCCAA TAGCGTATAT TAAAGTTGCT GCAGTTAAAA
NW_003775878                TGAGTCCACT TTAAATCCTT TAACGAGGAT CCATTGGAGG GCAAGTCTGG TGCCAGCAGC CGCGGTAATT CCAGCTCCAA TAGCGTATAT TAAAGTTGCT GCAGTTAAAA
DQ018752                    TGAGTCCACT TTAAATCCTT TAACGAGGAT CCATTGGAGG GCAAGTCTGG TGCCAGCAGC CGCGGTAATT CCAGCTCCAA TAGCGTATAT TAAAGTTGCT GCAGTTAAAA
DQ018757                    ---------- ---------- ---------- ---------- ---------- ---------- ---------- ---------- ---------- ---------- ----------
DQ112354                    ---------- ---------- ---------- ---------- ---------- ---------- ---------- ---------- ---------- ---------- ----------
FM165414                    TGAGTCCACT TTAAATCCTT TAACGAGGAT CCATTGGAGG GCAAGTCTGG TGCCAGCAGC CGCGGTAATT CCAGCTCCAA TAGCGTATAT TAAAGTTGCT GCAGTTAAAA
FM165415                    ---------- ---------- ---------- ---------- ---------- ---------- ---------- ---------- ---------- ---------- ----------
AADN03000430                ---------- ---------- ---------- ---------- ---------- ---------- ---------- ---------- ---------- ---------- ----------
AADN03001784                ---------- ---------- ---------- ---------- ---------- ---------- ---------- ---------- ---------- ---------- ----------
AADN03001783                ---------- ---------- ---------- ---------- ---------- ---------- ---------- ---------- ---------- ---------- ----------
DQ018754                    ---------- ---------- ---------- ---------- ---------- ---------- ---------- ---------- ---------- ---------- ----------
AADN03001782                ---------- ---------- ---------- ---------- ---------- ---------- ---------- ---------- ---------- ---------- ----------
JN639848                    ---------- ---------- ---------- ---------- ---------- ---------- ---------- ---------- ---------- ---------- ----------
AADN03001670                ---------- ---------- ---------- ---------- ---------- ---------- ---------- ---------- ---------- ---------- ----------
HQ873432                    TGAGTCCACT TTAAATCCTT TAACGAGGAT CCATTGGAGG GCAAGTCTGG TGCCAGCAGC CGCGGTAATT CCAGCTCCAA TAGCGTATAT TAAAGTTGCT GCAGTTAAAA
AADN03001774                ---------- ---------- ---------- ---------- ---------- ---------- ---------- ---------- ---------- ---------- ----------
AADN03001775                ---------- ---------- ---------- ---------- ---------- ---------- ---------- ---------- ---------- ---------- ----------
EF552813                    ---------- ---------- ---------- ---------- ---------- ---------- ---------- ---------- ---------- ---------- ----------
AADN03022685                ---------- ---------- ---------- ---------- ---------- ---------- ---------- ---------- ---------- ---------- ----------
AADN03019346                ---------- ---------- ---------- ---------- ---------- ---------- ---------- ---------- ---------- ---------- ----------
AADN03001776                ---------- ---------- ---------- ---------- ---------- ---------- ---------- ---------- ---------- ---------- ----------
DQ018756                    ---------- ---------- ---------- ---------- ---------- ---------- ---------- ---------- ---------- ---------- ----------
AF173612                    TGAGTCCACT TTAAATCCTT TAACGAGGAT CCATTGGAGG GCAAGTCTGG TGCCAGCAGC CGCGGTAATT CCAGCTCCAA TAGCGTATAT TAAAGTTGCT GCAGTTAAAA
Gallus_gallus_KT445934.2    TGAGTCCACT TTAAATCCTT TAACGAGGAT CCATTGGAGG GCAAGTCTGG TGCCAGCAGC CGCGGTAATT CCAGCTCCAA TAGCGTATAT TAAAGTTGCT GCAGTTAAAA
```

```
                             2222222222 2222222222 2222222222 2222222222 2222222222 2222222222 2222222222 2222222222 2222222222 2222222222 2222222222
                             5555555555 5555555555 5555555555 5555555555 5555555555 5555555555 5555555556 6666666666 6666666666 6666666666 6666666666
                             3333333334 4444444445 5555555556 6666666667 7777777778 8888888889 9999999990 0000000001 1111111112 2222222223 3333333334
                             1234567890 1234567890 1234567890 1234567890 1234567890 1234567890 1234567890 1234567890 1234567890 1234567890 1234567890
AADN03001677                 AGCTCGTAGT TGGATCTTGG GATCGAGCTG GCGGTCCGCC GCGAGGCGAG CTACCGCCTG TCCCAGCCCC TGTCTCTCGG CGCCCCCTCG ATGCTCTTAA CTGAGTGTCC
AADN03001778                 ---------- ---------- ---------- ---------- ---------- ---------- ---------- ---------- ---------- ---------- ----------
AADN03001785                 ---------- ---------- ---------- ---------- ---------- ---------- ---------- ---------- ---------- ---------- ----------
AADN03001786                 ---------- ---------- ---------- ---------- ---------- ---------- ---------- ---------- ---------- ---------- ----------
AADN03001788                 ---------- ---------- ---------- ---------- ---------- ---------- ---------- ---------- ---------- ---------- ----------
AADN03014081                 ---------- ---------- ---------- ---------- ---------- ---------- ---------- ---------- ---------- ---------- ----------
AADN03015064                 ---------- ---------- ---------- ---------- ---------- ---------- ---------- ---------- ---------- ---------- ----------
AADN03026634                 AGCTCGTAGT TGGATCTTGG GATCGAGCTG GCGGTCCGCC GCGAGGCGAG CTACCGCCTG TCCCAGCCCC TGTCTCTCGG CGCCCCCTCG ATGCTCTTAA CTGAGTGTCC
NW_003775878                 AGCTCGTAGT TGGATCTTGG GATCGAGCTG GCGGTCCGCC GCGAGGCGAG CTACCGCCTG TCCCAGCCCC TGTCTCTCGG CGCCCCCTCG ATGCTCTTAA CTGAGTGTCC
DQ018752                     AGCTCGTAGT TGGATCTTGG GATCGAGCTG GCGGTCCGCC GCGAGGCGAG CTACCGCCTG TCCCAGCCCC TGTCTCTCGG CGCCCCCTCG ATGCTCTTAA CTGAGTGTCC
DQ018757                     ---------- ---------- ---------- ---------- ---------- ---------- ---------- ---------- ---------- ---------- ----------
DQ112354                     ---------- ---------- ---------- ---------- ---------- ---------- ---------- ---------- ---------- ---------- ----------
FM165414                     AGCTCGTAGT TGGATCTTGG GATCGAGCTG GCGGTCCGCC GCGAGGCGAG CTACCGCCTG TCCCAGCCCC TGTCTCTCGG CGCCCCCTCG ATGCTCTTAA CTGAGTGTCC
FM165415                     ---------- ---------- ---------- ---------- ---------- ---------- ---------- ---------- ---------- ---------- ----------
AADN03000430                 ---------- ---------- ---------- ---------- ---------- ---------- ---------- ---------- ---------- ---------- ----------
AADN03001784                 ---------- ---------- ---------- ---------- ---------- ---------- ---------- ---------- ---------- ---------- ----------
AADN03001783                 ---------- ---------- ---------- ---------- ---------- ---------- ---------- ---------- ---------- ---------- ----------
DQ018754                     ---------- ---------- ---------- ---------- ---------- ---------- ---------- ---------- ---------- ---------- ----------
AADN03001782                 ---------- ---------- ---------- ---------- ---------- ---------- ---------- ---------- ---------- ---------- ----------
JN639848                     ---------- ---------- ---------- ---------- ---------- ---------- ---------- ---------- ---------- ---------- ----------
AADN03001670                 ---------- ---------- ---------- ---------- ---------- ---------- ---------- ---------- ---------- ---------- ----------
HQ873432                     AGCTCGTAGT TGGATCTTGG GATCGAGCTG GCGGTCCGCC GCGAGGCGAG CTACCGCCTG TCCCAGCCCC TGTCTCTCGG CGCCCCCTCG ATGCTCTTAA CTGAGTGTCC
AADN03001774                 ---------- ---------- ---------- ---------- ---------- ---------- ---------- ---------- ---------- ---------- ----------
AADN03001775                 ---------- ---------- ---------- ---------- ---------- ---------- ---------- ---------- ---------- ---------- ----------
EF552813                     ---------- ---------- ---------- ---------- ---------- ---------- ---------- ---------- ---------- ---------- ----------
AADN03022685                 ---------- ---------- ---------- ---------- ---------- ---------- ---------- ---------- ---------- ---------- ----------
AADN03019346                 ---------- ---------- ---------- ---------- ---------- ---------- ---------- ---------- ---------- ---------- ----------
AADN03001776                 ---------- ---------- ---------- ---------- ---------- ---------- ---------- ---------- ---------- ---------- ----------
DQ018756                     ---------- ---------- ---------- ---------- ---------- ---------- ---------- ---------- ---------- ---------- ----------
AF173612                     AGCTCGTAGT TGGATCTTGG GATCGAGCTG GCGGTCCGCC GCGAGGCGAG CTACCGCCTG TCCCAGCCCC TGTCTCTCGG CGCCCCCTCG ATGCTCTTAA CTGAGTGTCC
Gallus_gallus_KT445934.2     AGCTCGTAGT TGGATCTTGG GATCGAGCTG GCGGTCCGCC GCGAGGCGAG CTACCGCCTG TCCCAGCCCC TGTCTCTCGG CGCCCCCTCG ATGCTCTTAA CTGAGTGTCC

                             2222222222 2222222222 2222222222 2222222222 2222222222 2222222222 2222222222 2222222222 2222222222 2222222222 2222222222
                             6666666666 6666666666 6666666666 6666666666 6666666666 6666666667 7777777777 7777777777 7777777777 7777777777 7777777777
                             4444444445 5555555556 6666666667 7777777778 8888888889 9999999990 0000000001 1111111112 2222222223 3333333334 4444444445
                             1234567890 1234567890 1234567890 1234567890 1234567890 1234567890 1234567890 1234567890 1234567890 1234567890 1234567890
AADN03001677                 CGCGGGGCCC GAAGCGTTTA CTTTGAAAAA ATTAGAGTGT TCAAAGCAGG CTGGCCGCCG GAATACTCCA GCTAGGAATA ATGGAATAGG ACTCCGGTTC TATTTTGTTG
AADN03001778                 ---------- ---------- ---------- ---------- ---------- ---------- ---------- ---------- ---------- ---------- ----------
AADN03001785                 ---------- ---------- ---------- ---------- ---------- ---------- ---------- ---------- ---------- ---------- ----------
AADN03001786                 ---------- ---------- ---------- ---------- ---------- ---------- ---------- ---------- ---------- ---------- ----------
AADN03001788                 ---------- ---------- ---------- ---------- ---------- ---------- ---------- ---------- ---------- ---------- ----------
AADN03014081                 ---------- ---------- ---------- ---------- ---------- ---------- ---------- ---------- ---------- ---------- ----------
AADN03015064                 ---------- ---------- ---------- ---------- ---------- ---------- ---------- ---------- ---------- ---------- ----------
AADN03026634                 CGCGGGGCCC GAAGCGTTTA CTTTGAAAAA ATTAGAGTGT TCAAAGCAGG CTGGCCGCCG GAATACTCCA GCTAGGAATA ATGGAATAGG ACTCCGGTTC TATTTTGTTG
NW_003775878                 CGCGGGGCCC GAAGCGTTTA CTTTGAAAAA ATTAGAGTGT TCAAAGCAGG CTGGCCGCCG GAATACTCCA GCTAGGAATA ATGGAATAGG ACTCCGGTTC TATTTTGTTG
DQ018752                     CGCGGGGCCC GACCCGTTTA CTTTGAAAAA ATTAGAGTGG TCAAAGCAGG CTGGCCGCCG GAATACTCCA GCTAGGAATA ATGGAATAGG ACTCCGGTTC TATCTTGTTG
DQ018757                     ---------- ---------- ---------- ---------- ---------- ---------- ---------- ---------- ---------- ---------- ----------
DQ112354                     ---------- ---------- ---------- ---------- ---------- ---------- ---------- ---------- ---------- ---------- ----------
FM165414                     CGCGGGGCCC GAAGCGTTTA CTTTGAAAAA ATTAGAGTGT TCAAAGCAGG CTGGCCGCCG GAATACTCCA GCTAGGAATA ATGGAATAGG ACTCCGGTTC TATTTTGTTG
FM165415                     ---------- ---------- ---------- ---------- ---------- ---------- ---------- ---------- ---------- ---------- ----------
AADN03000430                 ---------- ---------- ---------- ---------- ---------- ---------- ---------- ---------- ---------- ---------- ----------
```

```
AADN03001784              ---------- ---------- ---------- ---------- ---------- ---------- ---------- ---------- ---------- ---------- ----------
AADN03001783              ---------- ---------- ---------- ---------- ---------- ---------- ---------- ---------- ---------- ---------- ----------
DQ018754                  ---------- ---------- ---------- ---------- ---------- ---------- ---------- ---------- ---------- ---------- ----------
AADN03001782              ---------- ---------- ---------- ---------- ---------- ---------- ---------- ---------- ---------- ---------- ----------
JN639848                  ---------- ---------- ---------- ---------- ---------- ---------- ---------- ---------- ---------- ---------- ----------
AADN03001670              ---------- ---------- ---------- ---------- ---------- ---------- ---------- ---------- ---------- ---------- ----------
HQ873432                  CGCGGGGCCC GAAGCGTTTA CTTTGAAAAA ATTAGAGTGT TCAAAGCAGG CTGGCCGCCG GAATACTCCA GCTAGGAATA ATGGAATAGG ACTCCGGTTC TATTTTGTTG
AADN03001774              ---------- ---------- ---------- ---------- ---------- ---------- ---------- ---------- ---------- ---------- ----------
AADN03001775              ---------- ---------- ---------- ---------- ---------- ---------- ---------- ---------- ---------- ---------- ----------
EF552813                  ---------- ---------- ---------- ---------- ---------- ---------- ---------- ---------- ---------- ---------- ----------
AADN03022685              ---------- ---------- ---------- ---------- ---------- ---------- ---------- ---------- ---------- ---------- ----------
AADN03019346              ---------- ---------- ---------- ---------- ---------- ---------- ---------- ---------- ---------- ---------- ----------
AADN03001776              ---------- ---------- ---------- ---------- ---------- ---------- ---------- ---------- ---------- ---------- ----------
DQ018756                  ---------- ---------- ---------- ---------- ---------- ---------- ---------- ---------- ---------- ---------- ----------
AF173612                  CGCGGGGCCC GAAGCGTTTA CTTTGAAAAA ATTAGAGTGT TCAAAGCAGG CTGGCCGCCG GAATACTCCA GCTAGGAATA ATGGAATAGG ACTCCGGTTC TATTTTGTTG
Gallus_gallus_KT445934.2  CGCGGGGCCC GAAGCGTTTA CTTTGAAAAA ATTAGAGTGT TCAAAGCAGG CTGGCCGCCG GAATACTCCA GCTAGGAATA ATGGAATAGG ACTCCGGTTC TATTTTGTTG

                          2222222222 2222222222 2222222222 2222222222 2222222222 2222222222 2222222222 2222222222 2222222222 2222222222 2222222222
                          7777777777 7777777777 7777777777 7777777777 7777777778 8888888888 8888888888 8888888888 8888888888 8888888888 8888888888
                          5555555556 6666666667 7777777778 8888888889 9999999990 0000000001 1111111112 2222222223 3333333334 4444444445 5555555556
                          1234567890 1234567890 1234567890 1234567890 1234567890 1234567890 1234567890 1234567890 1234567890 1234567890 1234567890
AADN03001677              GTTTTCGGAA ACGGGGCCAT GATTAAGAGG GACGGCCGGG GGCATTCGTA TTGTGCCGCT AGAGGTGAAA TTCTTGGACC GGCGCAAGAC GAACTAAAGC GAAAG-----
AADN03001778              ---------- ---------- ---------- ---------- ---------- ---------- ---------- ---------- ---------- ---------- ----------
AADN03001785              ---------- ---------- ---------- ---------- ---------- ---------- ---------- ---------- ---------- ---------- ----------
AADN03001786              ---------- ---------- ---------- ---------- ---------- ---------- ---------- ---------- ---------- ---------- ----------
AADN03001788              ---------- ---------- ---------- ---------- ---------- ---------- ---------- ---------- ---------- ---------- ----------
AADN03014081              ---------- ---------- ---------- ---------- ---------- ---------- ---------- ---------- ---------- ---------- ----------
AADN03015064              ---------- ---------- ---------- ---------- ---------- ---------- ---------- ---------- ---------- ---------- ----------
AADN03026634              GTTTTCGGAA ACGGGGCCAT GATTAAGAGG GACGGCCGGG GGCATTCGTA TTGTGCCGCT AGAGGTGAAA TTCTTGGACC GGCGCAAGAC GAACTAAAGC GAAAGCATTT
NW_003775878              GTTTTCGGAA ACGGGGCCAT GATTAAGAGG GACGGCCGGG GGCATTCGTA TTGTGCCGCT AGAGGTGAAA TTCTTGGACC GGCGCAAGAC GAACTAAAGC GAAAGCATTT
DQ018752                  GTTTTCGGAA ACGGGGCCAT GATTAAGAGG GACGGCCGGG GGCATTCGTA TTGTGCCGCT AGAGGTGAAA TTCTTGGACC GGCGCAAGAC GAACTAAAGC GAAAGCATTT
DQ018757                  ---------- ---------- ---------- ---------- ---------- ---------- ---------- ---------- ---------- ---------- ----------
DQ112354                  ---------- ---------- ---------- ---------- ---------- ---------- ---------- ---------- ---------- ---------- ----------
FM165414                  GTTTTCGGAA ACGGGGCCAT GATTAAGAGG GACGGCCGGG GGCATTCGTA TTGTGCCGCT AGAGGTGAAA TTCTTGGACC GGCGCAAGAC GAACTAAAGC GAAAGCATTT
FM165415                  ---------- ---------- ---------- ---------- ---------- ---------- ---------- ---------- ---------- ---------- ----------
AADN03000430              ---------- ---------- ---------- ---------- ---------- ---------- ---------- ---------- ---------- ---------- ----------
AADN03001784              ---------- ---------- ---------- ---------- ---------- ---------- ---------- ---------- ---------- ---------- ----------
AADN03001783              ---------- ---------- ---------- ---------- ---------- ---------- ---------- ---------- ---------- ---------- ----------
DQ018754                  ---------- ---------- ---------- ---------- ---------- ---------- ---------- ---------- ---------- ---------- ----------
AADN03001782              ---------- ---------- ---------- ---------- ---------- ---------- ---------- ---------- ---------- ---------- ----------
JN639848                  ---------- ---------- ---------- ---------- ---------- ---------- ---------- ---------- ---------- ---------- ----------
AADN03001670              ---------- ---------- ---------- ---------- ---------- ---------- ---------- ---------- ---------- ---------- ----------
HQ873432                  GTTTTCGGAA ACGGGGCCAT GATTAAGAGG GACGGCCGGG GGCATTCGTA TTGTGCCGCT AGAGGTGAAA TTCTTGGACC GGCGCAAGAC GAACTAAAGC GAAAGCATTT
AADN03001774              ---------- ---------- ---------- ---------- ---------- ---------- ---------- ---------- ---------- ---------- ----------
AADN03001775              ---------- ---------- ---------- ---------- ---------- ---------- ---------- ---------- ---------- ---------- ----------
EF552813                  ---------- ---------- ---------- ---------- ---------- ---------- ---------- ---------- ---------- ---------- ----------
AADN03022685              ---------- ---------- ---------- ---------- ---------- ---------- ---------- ---------- ---------- ---------- ----------
AADN03019346              ---------- ---------- ---------- ---------- ---------- ---------- ---------- ---------- ---------- ---------- ----------
AADN03001776              ---------- ---------- ---------- ---------- ---------- ---------- ---------- ---------- ---------- ---------- ----------
DQ018756                  ---------- ---------- ---------- ---------- ---------- ---------- ---------- ---------- ---------- ---------- ----------
AF173612                  GTTTTCGGAA ACGGGGCCAT GATTAAGAGG GACGGCCGGG GGCATTCGTA TTGTGCCGCT AGAGGTGAAA TTCTTGGACC GGCGCAAGAC GAACTAAAGC GAAAGCATTT
Gallus_gallus_KT445934.2  GTTTTCGGAA ACGGGGCCAT GATTAAGAGG GACGGCCGGG GGCATTCGTA TTGTGCCGCT AGAGGTGAAA TTCTTGGACC GGCGCAAGAC GAACTAAAGC GAAAGCATTT
```

```
                           2222222222 2222222222 2222222222 2222222222 2222222222 2222222222 2222222222 2222222222 2222222222 2222222222 2222222222
                           8888888888 8888888888 8888888888 8888888889 9999999999 9999999999 9999999999 9999999999 9999999999 9999999999 9999999999
                           6666666667 7777777778 8888888889 9999999990 0000000001 1111111112 2222222223 3333333334 4444444445 5555555556 6666666667
                           1234567890 1234567890 1234567890 1234567890 1234567890 1234567890 1234567890 1234567890 1234567890 1234567890 1234567890
AADN03001677               ---------- ---------- ---------- ---------- ---------- ---------- ---------- ---------- ---------- ---------- ----------
AADN03001778               ---------- ---------- ---------- ---------- ---------- ---------- ---------- ---------- ---------- ---------- ----------
AADN03001785               ---------- ---------- ---------- ---------- ---------- ---------- ---------- ---------- ---------- ---------- ----------
AADN03001786               ---------- ---------- ---------- ---------- ---------- ---------- ---------- ---------- ---------- ---------- ----------
AADN03001788               ---------- ---------- ---------- ---------- ---------- ---------- ---------- ---------- ---------- ---------- ----------
AADN03014081               ---------- ---------- ---------- ---------- ---------- ---------- ---------- ---------- ---------- ---------- ----------
AADN03015064               ---------- ---------- ---------- ---------- ---------- ---------- ---------- ---------- ---------- ---------- ----------
AADN03026634               GCCAAGAATG TTTTCATTAA TCAAGAACGA AAGTCGGAGG TTCGAAGACG ATCAGATACC GTCGTAGTTC CGACCATAAA CGATGCCGAC TCGCGATCCG GCGGCGTTAT
NW_003775878               GCCAAGAATG TTTTCATTAA TCAAGAACGA AAGTCGGAGG TTCGAAGACG ATCAGATACC GTCGTAGTTC CGACCATAAA CGATGCCGAC TCGCGATCCG GCGGCGTTAT
DQ018752                   GCCAAGAATG TTTTCATTAA TCAAGAACGA AAGTCGGAGG TTCGAAGACG ATCAGATACC GTCGTAGTTC CGACCATAAA CGATGCCGAC TCGCGATCCG GCGGCGTTAT
DQ018757                   ---------- ---------- ---------- ---------- ---------- ---------- ---------- ---------- ---------- ---------- ----------
DQ112354                   ---------- ---------- ---------- ---------- ---------- ---------- ---------- ---------- ---------- ---------- ----------
FM165414                   GCCAAGAATG TTTTCATTAA TCAAGAACGA AAGTCGGAGG TTCGAAGACG ATCAGATACC GTCGTAGTTC CGACCATAAA CGATGCCGAC TCGCGATCCG GCGGCGTTAT
FM165415                   ---------- ---------- ---------- ---------- ---------- ---------- ---------- ---------- ---------- ---------- ----------
AADN03000430               ---------- ---------- ---------- ---------- ---------- ---------- ---------- ---------- ---------- ---------- ----------
AADN03001784               ---------- ---------- ---------- ---------- ---------- ---------- ---------- ---------- ---------- ---------- ----------
AADN03001783               ---------- ---------- ---------- ---------- ---------- ---------- ---------- ---------- ---------- ---------- ----------
DQ018754                   ---------- ---------- ---------- ---------- ---------- ---------- ---------- ---------- ---------- ---------- ----------
AADN03001782               ---------- ---------- ---------- ---------- ---------- ---------- ---------- ---------- ---------- ---------- ----------
JN639848                   ---------- ---------- ---------- ---------- ---------- ---------- ---------- ---------- ---------- ---------- ----------
AADN03001670               ---------- ---------- ---------- ---------- ---------- ---------- ---------- ---------- ---------- ---------- ----------
HQ873432                   GCCAAGAATG TTTTCATTAA TCAAGAACGA AAGTCGGAGG TTCGAAGACG ATCAGATACC GTCGTAGTTC CGACCATAAA CGATGCCGAC TCGCGATCCG GCGGCGTTAT
AADN03001774               ---------- ---------- ---------- ---------- ---------- ---------- ---------- ---------- ---------- ---------- ----------
AADN03001775               ---------- ---------- ---------- ---------- ---------- ---------- ---------- ---------- ---------- ---------- ----------
EF552813                   ---------- ---------- ---------- ---------- ---------- ---------- ---------- ---------- ---------- ---------- ----------
AADN03022685               ---------- ---------- ---------- ---------- ---------- ---------- ---------- ---------- ---------- ---------- ----------
AADN03019346               ---------- ---------- ---------- ---------- ---------- ---------- ---------- ---------- ---------- ---------- ----------
AADN03001776               ---------- ---------- ---------- ---------- ---------- ---------- ---------- ---------- ---------- ---------- ----------
DQ018756                   ---------- ---------- ---------- ---------- ---------- ---------- ---------- ---------- ---------- ---------- ----------
AF173612                   GCCAAGAATG TTTTCATTAA TCAAGAACGA AAGTCGGAGG TTCGAAGACG ATCAGATACC GTCGTAGTTC CGACCATAAA CGATGCCGAC TCGCGATCCG GCGGCGTTAT
Gallus_gallus_KT445934.2   GCCAAGAATG TTTTCATTAA TCAAGAACGA AAGTCGGAGG TTCGAAGACG ATCAGATACC GTCGTAGTTC CGACCATAAA CGATGCCGAC TCGCGATCCG GCGGCGTTAT

                           2222222222 2222222222 2222222223 3333333333 3333333333 3333333333 3333333333 3333333333 3333333333 3333333333 3333333333
                           9999999999 9999999999 9999999990 0000000000 0000000000 0000000000 0000000000 0000000000 0000000000 0000000000 0000000000
                           7777777778 8888888889 9999999990 0000000001 1111111112 2222222223 3333333334 4444444445 5555555556 6666666667 7777777778
                           1234567890 1234567890 1234567890 1234567890 1234567890 1234567890 1234567890 1234567890 1234567890 1234567890 1234567890
AADN03001677               ---------- ---------- ---------- ---------- ---------- ---------- ---------- ---------- ---------- ---------- ----------
AADN03001778               ---------- ---------- ---------- ---------- ---------- ---------- ---------- ---------- ---------- ---------- ----------
AADN03001785               ---------- ---------- ---------- ---------- ---------- ---------- ---------- ---------- ---------- ---------- ----------
AADN03001786               ---------- ---------- ---------- ---------- ---------- ---------- ---------- ---------- ---------- ---------- ----------
AADN03001788               ---------- ---------- ---------- ---------- ---------- ---------- ---------- ---------- ---------- ---------- ----------
AADN03014081               ---------- ---------- ---------- ---------- ---------- ---------- ---------- ---------- ---------- ---------- ----------
AADN03015064               ---------- ---------- ---------- ---------- ---------- ---------- ---------- ---------- ---------- ---------- ----------
AADN03026634               TCCCATGACC CGCCGGGCAG CTCCCGGGAA ACCCAAGTCT TTGGGTTCCG GGGGGAGTAT GGTTGCAAAG CTGAAACTTA AAGGAATTGA CGGAAGGGCA CCACCAGGAG
NW_003775878               TCCCATGACC CGCCGGGCAG CTCCCGGGAA ACCCAAGTCT TTGGGTTCCG GGGGGAGTAT GGTTGCAAAG CTGAAACTTA AAGGAATTGA CGGAAGGGCA CCACCAGGAG
DQ018752                   TCCCATGACC CGCCGGGCAG CTCCCGGGAA ACCCAAGTCT TTGGGTTCCG GGGGGAGTAT GGTTGCAAAG CTGAAACTTA AAGGAATTGA CGGAAGGGCA CCACCAGGAG
DQ018757                   ---------- ---------- ---------- ---------- ---------- ---------- ---------- ---------- ---------- ---------- ----------
DQ112354                   ---------- ---------- ---------- ---------- ---------- ---------- ---------- ---------- ---------- ---------- ----------
FM165414                   TCCCATGACC CGCCGGGCAG CTCCCGGGAA ACCCAAGTCT TTGGGTTCCG GGGGGAGTAT GGTTGCAAAG CTGAAACTTA AAGGAATTGA CGGAAGGGCA CCACCAGGAG
FM165415                   ---------- ---------- ---------- ---------- ---------- ---------- ---------- ---------- ---------- ---------- ----------
AADN03000430               ---------- ---------- ---------- ---------- ---------- ---------- ---------- ---------- ---------- ---------- ----------
```

```
AADN03001784               ---------- ---------- ---------- ---------- ---------- ---------- ---------- ---------- ---------- ---------- ----------
AADN03001783               ---------- ---------- ---------- ---------- ---------- ---------- ---------- ---------- ---------- ---------- ----------
DQ018754                   ---------- ---------- ---------- ---------- ---------- ---------- ---------- ---------- ---------- ---------- ----------
AADN03001782               ---------- ---------- ---------- ---------- ---------- ---------- ---------- ---------- ---------- ---------- ----------
JN639848                   ---------- ---------- ---------- ---------- ---------- ---------- ---------- ---------- ---------- ---------- ----------
AADN03001670               ---------- ---------- ---------- ---------- ---------- ---------- ---------- ---------- ---------- ---------- ----------
HQ873432                   TCCCATGACC CGCCGGGCAG CTCCCGGGAA ACCCAAGTCT TTGGGTTCCG GGGGGAGTAT GGTTGCAAAG CTGAAACTTA AAGGAATTGA CGGAAGGGCA CCACCAGGAG
AADN03001774               ---------- ---------- ---------- ---------- ---------- ---------- ---------- ---------- ---------- ---------- ----------
AADN03001775               ---------- ---------- ---------- ---------- ---------- ---------- ---------- ---------- ---------- ---------- ----------
EF552813                   ---------- ---------- ---------- ---------- ---------- ---------- ---------- ---------- ---------- ---------- ----------
AADN03022685               ---------- ---------- ---------- ---------- ---------- ---------- ---------- ---------- ---------- ---------- ----------
AADN03019346               ---------- ---------- ---------- ---------- ---------- ---------- ---------- ---------- ---------- ---------- ----------
AADN03001776               ---------- ---------- ---------- ---------- ---------- ---------- ---------- ---------- ---------- ---------- ----------
DQ018756                   ---------- ---------- ---------- ---------- ---------- ---------- ---------- ---------- ---------- ---------- ----------
AF173612                   TCCCATGACC CGCCGGGCAG CTCCCGGGAA ACCCAAGTCT TTGGGTTCCG GGGGGAGTAT GGTTGCAAAG CTGAAACTTA AAGGAATTGA CGGAAGGGCA CCACCAGGAG
Gallus_gallus_KT445934.2   TCCCATGACC CGCCGGGCAG CTCCCGGGAA ACCCAAGTCT TTGGGTTCCG GGGGGAGTAT GGTTGCAAAG CTGAAACTTA AAGGAATTGA CGGAAGGGCA CCACCAGGAG

                           3333333333 3333333333 3333333333 3333333333 3333333333 3333333333 3333333333 3333333333 3333333333 3333333333 3333333333
                           0000000000 0000000001 1111111111 1111111111 1111111111 1111111111 1111111111 1111111111 1111111111 1111111111 1111111111
                           8888888889 9999999990 0000000001 1111111112 2222222223 3333333334 4444444445 5555555556 6666666667 7777777778 8888888889
                           1234567890 1234567890 1234567890 1234567890 1234567890 1234567890 1234567890 1234567890 1234567890 1234567890 1234567890
AADN03001677               ---------- ---------- ---------- ---------- ---------- ---------- ---------- ---------- ---------- ---------- ----------
AADN03001778               ---------- ---------- ---------- ---------- ---------- ---------- ---------- ---------- ---------- ---------- ----------
AADN03001785               ---------- ---------- ---------- ---------- ---------- ---------- ---------- ---------- ---------- ---------- ----------
AADN03001786               ---------- ---------- ---------- ---------- ---------- ---------- ---------- ---------- ---------- ---------- ----------
AADN03001788               ---------- ---------- ---------- ---------- ---------- ---------- ---------- ---------- ---------- ---------- ----------
AADN03014081               ---------- ---------- ---------- ---------- ---------- ---------- ---------- ---------- ---------- ---------- ----------
AADN03015064               ---------- ---------- ---------- ---------- ---------- ---------- ---------- ---------- ---------- ---------- ----------
AADN03026634               TGGAGCCTGC GGCTTAATTT GACTCAACAC GGGAAACCTC ACCCGGCCCG GACACGGACA GGATTGACAG ATTGAGAGCT CTTTCTCGAT TCCGTGGGTG GTGGTGCATG
NW_003775878               TGGAGCCTGC GGCTTAATTT GACTCAACAC GGGAAACCTC ACCCGGCCCG GACACGGACA GGATTGACAG ATTGAGAGCT CTTTCTCGAT TCCGTGGGTG GTGGTGCATG
DQ018752                   TGGAGCCTGC GGCTTAATTT GACTCAACAC GGGAAACCTC ACCCGGCCCG GACACGGACA GGATTGACAG ATTGAGAGCT CTTTCTCGAT TCCGTGGGTG GTGGTGCATG
DQ018757                   ---------- ---------- ---------- ---------- ---------- ---------- ---------- ---------- ---------- ---------- ----------
DQ112354                   ---------- ---------- ---------- ---------- ---------- ---------- ---------- ---------- ---------- ---------- ----------
FM165414                   TGGAGCCTGC GGCTTAATTT GACTCAACAC GGGAAACCTC ACCCGGCCCG GACACGGACA GGATTGACAG ATTGAGAGCT CTTTCTCGAT TCCGTGGGTG GTGGTGCATG
FM165415                   ---------- ---------- ---------- ---------- ---------- ---------- ---------- ---------- ---------- ---------- ----------
AADN03000430               ---------- ---------- ---------- ---------- ---------- ---------- ---------- ---------- ---------- ---------- ----------
AADN03001784               ---------- ---------- ---------- ---------- ---------- ---------- ---------- ---------- ---------- ---------- ----------
AADN03001783               ---------- ---------- ---------- ---------- ---------- ---------- ---------- ---------- ---------- ---------- ----------
DQ018754                   ---------- ---------- ---------- ---------- ---------- ---------- ---------- ---------- ---------- ---------- ----------
AADN03001782               ---------- ---------- ---------- ---------- ---------- ---------- ---------- ---------- ---------- ---------- ----------
JN639848                   ---------- ---------- ---------- ---------- ---------- ---------- ---------- ---------- ---------- ---------- ----------
AADN03001670               ---------- ---------- ---------- ---------- ---------- ---------- ---------- ---------- ---------- ---------- ----------
HQ873432                   TGGAGCCTGC GGCTTAATTT GACTCAACAC GGGAAACCTC ACCCGGCCCG GACACGGACA GGATTGACAG ATTGAGAGCT CTTTCTCGAT TCCGTGGGTG GTGGTGCATG
AADN03001774               ---------- ---------- ---------- ---------- ---------- ---------- ---------- ---------- ---------- ---------- ----------
AADN03001775               ---------- ---------- ---------- ---------- ---------- ---------- ---------- ---------- ---------- ---------- ----------
EF552813                   ---------- ---------- ---------- ---------- ---------- ---------- ---------- ---------- ---------- ---------- ----------
AADN03022685               ---------- ---------- ---------- ---------- ---------- ---------- ---------- ---------- ---------- ---------- ----------
AADN03019346               ---------- ---------- ---------- ---------- ---------- ---------- ---------- ---------- ---------- ---------- ----------
AADN03001776               ---------- ---------- ---------- ---------- ---------- ---------- ---------- ---------- ---------- ---------- ----------
DQ018756                   ---------- ---------- ---------- ---------- ---------- ---------- ---------- ---------- ---------- ---------- ----------
AF173612                   TGGAGCCTGC GGCTTAATTT GACTCAACAC GGGAAACCTC ACCCGGCCCG GACACGGACA GGATTGACAG ATTGAGAGCT CTTTCTCGAT TCCGTGGGTG GTGGTGCATG
Gallus_gallus_KT445934.2   TGGAGCCTGC GGCTTAATTT GACTCAACAC GGGAAACCTC ACCCGGCCCG GACACGGACA GGATTGACAG ATTGAGAGCT CTTTCTCGAT TCCGTGGGTG GTGGTGCATG
```

```
                            3333333333 3333333333 3333333333 3333333333 3333333333 3333333333 3333333333 3333333333 3333333333 3333333333 3333333333
                            1111111112 2222222222 2222222222 2222222222 2222222222 2222222222 2222222222 2222222222 2222222222 2222222222 2222222223
                            9999999990 0000000001 1111111112 2222222223 3333333334 4444444445 5555555556 6666666667 7777777778 8888888889 9999999990
                            1234567890 1234567890 1234567890 1234567890 1234567890 1234567890 1234567890 1234567890 1234567890 1234567890 1234567890
AADN03001677                ---------- ---------- ---------- ---------- ---------- ---------- ---------- ---------- ---------- ---------- ----------
AADN03001778                ---------- ---------- ---------- ---------- ---------- ---------- ---------- ---------- ---------- ---------- ----------
AADN03001785                ---------- ---------- ---------- ---------- ---------- ---------- ---------- ---------- ---------- ---------- ----------
AADN03001786                ---------- ---------- ---------- ---------- ---------- ---------- ---------- ---------- ---------- ---------- ----------
AADN03001788                ---------- ---------- ---------- ---------- ---------- ---------- ---------- ---------- ---------- ---------- ----------
AADN03014081                ---------- ---------- ---------- ---------- ---------- ---------- ---------- ---------- ---------- ---------- ----------
AADN03015064                ---------- ---------- ---------- ---------- ---------- ---------- ---------- ---------- ---------- ---------- ----------
AADN03026634                GCCGTTCTTA GTTGGTGGAG CGATTTGTCT GGTTAATTCC GATAACGAAC GAGACTCTGG CATGCTAACT AGTTACGCGA CCCCCGAGCG GTCGGCGTCC AACTTCTTAG
NW_003775878                GCCGTTCTTA GTTGGTGGAG CGATTTGTCT GGTTAATTCC GATAACGAAC GAGACTCTGG CATGCTAACT AGTTACGCGA CCCCCGAGCG GTCGGCGTCC AACTTCTTAG
DQ018752                    GCCGTTCTTA GTTGGTGGAG CGATTTGTCT GGTTAATTCC GATAACGAAC GAGACTCTGG CATGCTAACT AGTTACGCGA CCCCCGAGCG GTCGGCGTCC AACTTCTTAG
DQ018757                    ---------- ---------- ---------- ---------- ---------- ---------- ---------- ---------- ---------- ---------- ----------
DQ112354                    ---------- ---------- ---------- ---------- ---------- ---------- ---------- ---------- ---------- ---------- ----------
FM165414                    GCCGTTCTTA GTTGGTGGAG CGATTTGTCT GGTTAATTCC GATAACGAAC GAGACTCTGG CATGCTAACT AGTTACGCGA CCCCCGAGCG GTCGGCGTCC AACTTCTTAG
FM165415                    ---------- ---------- ---------- ---------- ---------- ---------- ---------- ---------- ---------- ---------- ----------
AADN03000430                ---------- ---------- ---------- ---------- ---------- ---------- ---------- ---------- ---------- ---------- ----------
AADN03001784                ---------- ---------- ---------- ---------- ---------- ---------- ---------- ---------- ---------- ---------- ----------
AADN03001783                ---------- ---------- ---------- ---------- ---------- ---------- ---------- ---------- ---------- ---------- ----------
DQ018754                    ---------- ---------- ---------- ---------- ---------- ---------- ---------- ---------- ---------- ---------- ----------
AADN03001782                ---------- ---------- ---------- ---------- ---------- ---------- ---------- ---------- ---------- ---------- ----------
JN639848                    ---------- ---------- ---------- ---------- ---------- ---------- ---------- ---------- ---------- ---------- ----------
AADN03001670                ---------- ---------- ---------- ---------- ---------- ---------- ---------- ---------- ---------- ---------- ----------
HQ873432                    GCCGTTCTTA GTTGGTGGAG CGATTTGTCT GGTTAATTCC GATAACGAAC GAGACTCTGG CATGCTAACT AGTTACGCGA CCCCCGAGCG GTCGGCGTCC AACTTCTTAG
AADN03001774                ---------- ---------- ---------- ---------- ---------- ---------- ---------- ---------- ---------- ---------- ----------
AADN03001775                ---------- ---------- ---------- ---------- ---------- ---------- ---------- ---------- ---------- ---------- ----------
EF552813                    ---------- ---------- ---------- ---------- ---------- ---------- ---------- ---------- ---------- ---------- ----------
AADN03022685                ---------- ---------- ---------- ---------- ---------- ---------- ---------- ---------- ---------- ---------- ----------
AADN03019346                ---------- ---------- ---------- ---------- ---------- ---------- ---------- ---------- ---------- ---------- ----------
AADN03001776                ---------- ---------- ---------- ---------- ---------- ---------- ---------- ---------- ---------- ---------- ----------
DQ018756                    ---------- ---------- ---------- ---------- ---------- ---------- ---------- ---------- ---------- ---------- ----------
AF173612                    GCCGTTCTTA GTTGGTGGAG CGATTTGTCT GGTTAATTCC GATAACGAAC GAGACTCTGG CATGCTAACT AGTTACGCGA CCCCCGAGCG GTCGGCGTCC AACTTCTTAG
Gallus_gallus_KT445934.2    GCCGTTCTTA GTTGGTGGAG CGATTTGTCT GGTTAATTCC GATAACGAAC GAGACTCTGG CATGCTAACT AGTTACGCGA CCCCCGAGCG GTCGGCGTCC AACTTCTTAG

                            3333333333 3333333333 3333333333 3333333333 3333333333 3333333333 3333333333 3333333333 3333333333 3333333333 3333333333
                            3333333333 3333333333 3333333333 3333333333 3333333333 3333333333 3333333333 3333333333 3333333333 3333333334 4444444444
                            0000000001 1111111112 2222222223 3333333334 4444444445 5555555556 6666666667 7777777778 8888888889 9999999990 0000000001
                            1234567890 1234567890 1234567890 1234567890 1234567890 1234567890 1234567890 1234567890 1234567890 1234567890 1234567890
AADN03001677                ---------- ---------- ---------- ---------- ---------- ---------- ---------- ---------- ---------- ---------- ----------
AADN03001778                ---------- ---------- ---------- ---------- ---------- ---------- ---------- ---------- ---------- ---------- ----------
AADN03001785                ---------- ---------- ---------- ---------- ---------- ---------- ---------- ---------- ---------- ---------- ----------
AADN03001786                ---------- ---------- ---------- ---------- ---------- ---------- ---------- ---------- ---------- ---------- ----------
AADN03001788                ---------- ---------- ---------- ---------- ---------- ---------- ---------- ---------- ---------- ---------- ----------
AADN03014081                ---------- ---------- ---------- ---------- ---------- ---------- ---------- ---------- ---------- ---------- ----------
AADN03015064                ---------- ---------- ---------- ---------- ---------- ---------- ---------- ---------- ---------- ---------- ----------
AADN03026634                AGGGACAAGT G-GCGTTCAG CCACCCGAGA TTGAGCAATA ACAGGTCTGT GATGC-CCTT AGATGTCCGG GGCTGCACGC GCGCTACACT GACTGGCTCA GCTTGTGTCT
NW_003775878                AGGGACAAGT G-GCGTTCAG CCACCCGAGA TTGAGCAATA ACAGGTCTGT GATGC-CCTT AGATGTCCGG GGCTGCACGC GCGCTACACT GACTGGCTCA GCTTGTGTCT
DQ018752                    AGGGACAAGT G-GCGTTCAG CCACCCGAGA TTGAGCAATA ACAGGTCTGT GATGC-CCTT AGATGTCCGG GGCTGCACGC GCGCTACACT GACTGGCTCA GCTTGTGTCT
DQ018757                    ---------- ---------- ---------- ---------- ---------- ---------- ---------- ---------- ---------- ---------- ----------
DQ112354                    ---------- ---------- ---------- ---------- ---------- ---------- ---------- ---------- ---------- ---------- ----------
FM165414                    AGGGACAAGT G-GCGTTCAG CCACCCGAGA TTGAGCAATA ACAGGTCTGT GATGC-CCTT AGATGTCCGG GGCTGCACGC GCGCTACACT GACTGGCTCA GCTTGTGTCT
FM165415                    ---------- ---------- ---------- ---------- ---------- ---------- ---------- ---------- ---------- ---------- ----------
AADN03000430                ---------- ---------- ---------- ---------- ---------- ---------- ---------- ---------- ---------- ---------- ----------
```

```
AADN03001784              ---------- ---------- ---------- ---------- ---------- ---------- ---------- ---------- ---------- ---------- ----------
AADN03001783              ---------- ---------- ---------- ---------- ---------- ---------- ---------- ---------- ---------- ---------- ----------
DQ018754                  ---------- ---------- ---------- ---------- ---------- ---------- ---------- ---------- ---------- ---------- ----------
AADN03001782              ---------- ---------- ---------- ---------- ---------- ---------- ---------- ---------- ---------- ---------- ----------
JN639848                  ---------- ---------- ---------- ---------- ---------- ---------- ---------- ---------- ---------- ---------- ----------
AADN03001670              ---------- ---------- ---------- ---------- ---------- ---------- ---------- ---------- ---------- ---------- ----------
HQ873432                  AGGGACAAGT GCGCGTTCAG CCACCCGAGA TTGAGCAATA ACAGGTCTGT GATGCGCCTT AGATGTCCGG GGCTGCACGC GCGCTACACT GACTGGCTCA GCTTGTGTCT
AADN03001774              ---------- ---------- ---------- ---------- ---------- ---------- ---------- ---------- ---------- ---------- ----------
AADN03001775              ---------- ---------- ---------- ---------- ---------- ---------- ---------- ---------- ---------- ---------- ----------
EF552813                  ---------- ---------- ---------- ---------- ---------- ---------- ---------- ---------- ---------- ---------- ----------
AADN03022685              ---------- ---------- ---------- ---------- ---------- ---------- ---------- ---------- ---------- ---------- ----------
AADN03019346              ---------- ---------- ---------- ---------- ---------- ---------- ---------- ---------- ---------- ---------- ----------
AADN03001776              ---------- ---------- ---------- ---------- ---------- ---------- ---------- ---------- ---------- ---------- ----------
DQ018756                  ---------- ---------- ---------- ---------- ---------- ---------- ---------- ---------- ---------- ---------- ----------
AF173612                  AGGGACAAGT G-GCGTTCAG CCACCCGAGA TTGAGCAATA ACAGGTCTGT GATGC-CCTT AGATGTCCGG GGCTGCACGC GCGCTACACT GACTGGCTCA GCTTGTGTCT
Gallus_gallus_KT445934.2  AGGGACAAGT G-GCGTTCAG CCACCCGAGA TTGAGCAATA ACAGGTCTGT GATGC-CCTT AGATGTCCGG GGCTGCACGC GCGCTACACT GACTGGCTCA GCTTGTGTCT

                          3333333333 3333333333 3333333333 3333333333 3333333333 3333333333 3333333333 3333333333 3333333333 3333333333 3333333333
                          4444444444 4444444444 4444444444 4444444444 4444444444 4444444444 4444444444 4444444444 4444444445 5555555555 5555555555
                          1111111112 2222222223 3333333334 4444444445 5555555556 6666666667 7777777778 8888888889 9999999990 0000000001 1111111112
                          1234567890 1234567890 1234567890 1234567890 1234567890 1234567890 1234567890 1234567890 1234567890 1234567890 1234567890
AADN03001677              ---------- ---------- ---------- ---------- ---------- ---------- ---------- ---------- ---------- ---------- ----------
AADN03001778              ---------- ---------- ---------- ---------- ---------- ---------- ---------- ---------- ---------- ---------- ----------
AADN03001785              ---------- ---------- ---------- ---------- ---------- ---------- ---------- ---------- ---------- ---------- ----------
AADN03001786              ---------- ---------- ---------- ---------- ---------- ---------- ---------- ---------- ---------- ---------- ----------
AADN03001788              ---------- ---------- ---------- ---------- ---------- ---------- ---------- ---------- ---------- ---------- ----------
AADN03014081              ---------- ---------- ---------- ---------- ---------- ---------- ---------- ---------- ---------- ---------- ----------
AADN03015064              ---------- ---------- ---------- ---------- ---------- ---------- ---------- ---------- ---------- ---------- ----------
AADN03026634              ACCCTACGCC GGCAGGCGCG GGTAACCCGT TGAACCCCAT TCGTGATGGG GATCGGGGAT TGCAATTATT CCCCATGAAC GAGGAATTCC CAGTAAGTGC GGGTCATAAG
NW_003775878              ACCCTACGCC GGCAGGCGCG GGTAACCCGT TGAACCCCAT TCGTGATGGG GATCGGGGAT TGCAATTATT CCCCATGAAC GAGGAATTCC CAGTAAGTGC GGGTCATAAG
DQ018752                  ACCCTACGCC GGCAGACGCG GGTAACCCGT TGAACCCCAT TCGTGATGGG GATCGGGGAT TGCAATTATT CCCCATGAAC GAGGAATTCC CAGTAAGTGC GGGTCATAAG
DQ018757                  ---------- ---------- ---------- ---------- ---------- ---------- ---------- ---------- ---------- ---------- ----------
DQ112354                  ---------- ---------- ---------- ---------- ---------- ---------- ---------- ---------- ---------- ---------- ----------
FM165414                  ACCCTACGCC GGCAGGCGCG GGTAACCCGT TGAACCCCAT TCGTGATGGG GATCGGGGAT TGCAATTATT CCCCATGAAC GAGGAATTCC CAGTAAGTGC GGGTCATAAG
FM165415                  ---------- ---------- ---------- ---------- ---------- ---------- ---------- ---------- ---------- ---------- ----------
AADN03000430              ---------- ---------- ---------- ---------- --GTGATGGG GATCGGGGAT TGCAATTATT CCCCATGAAC GAGGAATTCC CAGTAAGTGC GGGTCATAAG
AADN03001784              ---------- ---------- ---------- ---------- ---------- ---------- ---------- ---------- ---------- ---------- ----------
AADN03001783              ---------- ---------- ---------- ---------- ---------- ---------- ---------- ---------- ---------- ---------- ----------
DQ018754                  ---------- ---------- ---------- ---------- ---------- ---------- ---------- ---------- ---------- ---------- ----------
AADN03001782              ---------- ---------- ---------- ---------- ---------- ---------- ---------- ---------- ---------- ---------- ----------
JN639848                  ---------- ---------- ---------- ---------- ---------- ---------- ---------- ---------- ---------- ---------- ----------
AADN03001670              ---------- ---------- ---------- ---------- ---------- ---------- ---------- ---------- ---------- ---------- ----------
HQ873432                  ACCCTACGCC GGCAGGCGCG GGTAACCCGT TGAACCCCAT TCGTGATGGG GATCGGGGAT TGCAATTATT CCCCATGAAC GAGGAATTCC CAGTAAGTGC GGGTCATAAG
AADN03001774              ---------- ---------- ---------- ---------- ---------- ---------- ---------- ---------- ---------- ---------- ----------
AADN03001775              ---------- ---------- ---------- ---------- ---------- ---------- ---------- ---------- ---------- ---------- ----------
EF552813                  ---------- ---------- ---------- ---------- ---------- ---------- ---------- ---------- ---------- ---------- ----------
AADN03022685              ---------- ---------- ---------- ---------- ---------- ---------- ---------- ---------- ---------- ---------- ----------
AADN03019346              ---------- ---------- ---------- ---------- ---------- ---------- ---------- ---------- ---------- ---------- ----------
AADN03001776              ---------- ---------- ---------- ---------- ---------- ---------- ---------- ---------- ---------- ---------- ----------
DQ018756                  ---------- ---------- ---------- ---------- ---------- ---------- ---------- ---------- ---------- ---------- ----------
AF173612                  ACCCTACGCC GGCAGGCGCG GGTAACCCGT TGAACCCCAT TCGTGATGGG GATCGGGGAT TGCAATTATT CCCCATGAAC GAGGAATTCC CAGTAAGTGC GGGTCATAAG
Gallus_gallus_KT445934.2  ACCCTACGCC GGCAGGCGCG GGTAACCCGT TGAACCCCAT TCGTGATGGG GATCGGGGAT TGCAATTATT CCCCATGAAC GAGGAATTCC CAGTAAGTGC GGGTCATAAG
```

```
                              3333333333 3333333333 3333333333 3333333333 3333333333 3333333333 3333333333 3333333333 3333333333 3333333333 3333333333
                              5555555555 5555555555 5555555555 5555555555 5555555555 5555555555 5555555555 5555555556 6666666666 6666666666 6666666666
                              2222222223 3333333334 4444444445 5555555556 6666666667 7777777778 8888888889 9999999990 0000000001 1111111112 2222222223
                              1234567890 1234567890 1234567890 1234567890 1234567890 1234567890 1234567890 1234567890 1234567890 1234567890 1234567890
AADN03001677                  ---------- ---------- ---------- ---------- ---------- ---------- ---------- ---------- ---------- ---------- ----------
AADN03001778                  ---------- ---------- ---------- ---------- ---------- ---------- ---------- ---------- ---------- ---------- ----------
AADN03001785                  ---------- ---------- ---------- ---------- ---------- ---------- ---------- ---------- ---------- ---------- ----------
AADN03001786                  ---------- ---------- ---------- ---------- ---------- ---------- ---------- ---------- ---------- ---------- ----------
AADN03001788                  ---------- ---------- ---------- ---------- ---------- ---------- ---------- ---------- ---------- ---------- ----------
AADN03014081                  ---------- ---------- ---------- ---------- ---------- ---------- ---------- ---------- ---------- ---------- ----------
AADN03015064                  ---------- ---------- ---------- ---------- ---------- ---------- ---------- ---------- ---------- ---------- ----------
AADN03026634                  CTCGCGTTGA TTAAGTCCCT GCCCTTTGTA CACACCGCCC GTCGCTACTA AGGGTTGGAT GGTTTAGTGA GGTCCTCGGA TCGGCCCCGG CGGGGTCGGC CACGGCCCTG
NW_003775878                  CTCGCGTTGA TTAAGTCCCT GCCCTTTGTA CACACCGCCC GTCGCTACTA AGGGTTGGAT GGTTTAGTGA GGTCCTCGGA TCGGCCCCGG CGGGGTCGGC CACGGCCCTG
DQ018752                      CTCGCGTTGA TTAAGTCCCT GCCCTTTGTA CACACCGCCC GTCGCTACTA CCGATTGGAT GGTTTAGTGA GGTCCTCGGA TCGGCCCCGG CGGGGTCGGC CACGGCCCTG
DQ018757                      ---------- ---------- ---------- ---------- ---------- ---------- ---------- ---------- ---------- ---------- ----------
DQ112354                      ---------- ---------- ---------- ---------- ---------- ---------- ---------- ---------- ---------- ---------- ----------
FM165414                      CTCGCGTTGA TTAAGTCCCT GCCCTTTGTA CACACCGCCC GTCGCTACTA CCGATTGGAT GGTTTAGTGA GGTCCTCGGA TCGGCCCCGG CGGGGTCGGC CACGGCCCTG
FM165415                      ---------- ---------- ---------- ---------- ---------- ---------- ---------- ---------- ---------- ---------- ----------
AADN03000430                  CTCGCGTTGA TTAAGTCCCT GCCCTTTGTA CACACCGCCC GTCGCTACTA CCGATTGGAT GGTTTAGTGA GGTCCTCGGA TCGGCCCCGG CGGGGTCGGC CACGGCCCTG
AADN03001784                  ---------- ---------- ---------- ---------- ---------- ---------- ---------- ---------- ---------- ---------- ----------
AADN03001783                  ---------- ---------- ---------- ---------- ---------- ---------- ---------- ---------- ---------- ---------- ----------
DQ018754                      CTCGCGTTGA TTAAGTCCCT GCCCTTTGTA CACACCGCCC GTCGCTACTA CCGATTGGAT GGTTTAGTGA GGTCCTCGGA TCGGCCCCGG CGGGGTCGGC CACGGCCCTG
AADN03001782                  ---------- ---------- ---------- ---------- ---------- ---------- ---------- ---------- ---------- ---------- ----------
JN639848                      ---------- ---------- ---------- ---------- ---------- ---------- ---------- ---------- ---------- ---------- ----------
AADN03001670                  ---------- ---------- ---------- ---------- ---------- ---------- ---------- ---------- ---------- ---------- ----------
HQ873432                      CTCGCGTTGA TTAAGTCCCT GCCCTTTGTA CACACCGCCC GTCGCTACTA CCGATTGGAT GGTTTAGTGA GGTCCTCGGA TCGGCCCCGG CGGGGTCGGC CACGGCCCTG
AADN03001774                  ---------- ---------- ---------- ---------- ---------- ---------- ---------- ---------- ---------- ---------- ----------
AADN03001775                  ---------- ---------- ---------- ---------- ---------- ---------- ---------- ---------- ---------- ---------- ----------
EF552813                      ---------- ---------- ---------- ---------- ---------- ---------- ---------- ---------- ---------- ---------- ----------
AADN03022685                  ---------- ---------- ---------- ---------- ---------- ---------- ---------- ---------- ---------- ---------- ----------
AADN03019346                  ---------- ---------- ---------- ---------- ---------- ---------- ---------- ---------- ---------- ---------- ----------
AADN03001776                  ---------- ---------- ---------- ---------- ---------- ---------- ---------- ---------- ---------- ---------- ----------
DQ018756                      ---------- ---------- ---------- ---------- ---------- ---------- ---------- ---------- ---------- ---------- ----------
AF173612                      CTCGCGTTGA TTAAGTCCCT GCCCTTTGTA CACACCGCCC GTCGCTACTA CCGATTGGAT GGTTTAGTGA GGTCCTCGGA TCGGCCCCGG CGGGGTCGGC CACGGCCCTG
Gallus_gallus_KT445934.2      CTCGCGTTGA TTAAGTCCCT GCCCTTTGTA CACACCGCCC GTCGCTACTA CCGATTGGAT GGTTTAGTGA GGTCCTCGGA TCGGCCCCGG CGGGGTCGGC CACGGCCCTG

                              3333333333 3333333333 3333333333 3333333333 3333333333 3333333333 3333333333 3333333333 3333333333 3333333333 3333333333
                              6666666666 6666666666 6666666666 6666666666 6666666666 6666666666 6666666667 7777777777 7777777777 7777777777 7777777777
                              3333333334 4444444445 5555555556 6666666667 7777777778 8888888889 9999999990 0000000001 1111111112 2222222223 3333333334
                              1234567890 1234567890 1234567890 1234567890 1234567890 1234567890 1234567890 1234567890 1234567890 1234567890 1234567890
AADN03001677                  ---------- ---------- ---------- ---------- ---------- ---------- ---------- ---------- ---------- ---------- ----------
AADN03001778                  ---------- ---------- ---------- ---------- ---------- ---------- ---------- ---------- ---------- ---------- ----------
AADN03001785                  ---------- ---------- ---------- ---------- ---------- ---------- ---------- ---------- ---------- ---------- ----------
AADN03001786                  ---------- ---------- ---------- ---------- ---------- ---------- ---------- ---------- ---------- ---------- ----------
AADN03001788                  ---------- ---------- ---------- ---------- ---------- ---------- ---------- ---------- ---------- ---------- ----------
AADN03014081                  ---------- ---------- ---------- ---------- ---------- ---------- ---------- ---------- ---------- ---------- ----------
AADN03015064                  ---------- ---------- ---------- ---------- ---------- ---------- ---------- ---------- ---------- ---------- ----------
AADN03026634                  CCGGAGCGTC GAGAAGACGG TCGAACTTGA CTATCTAGAG GAAGTAAAAG TCGTAACAAG GTTTCCGTAG GTGAACCTGC GGAAGGATCA TTACCGGGGC CGAGGCCGGG
NW_003775878                  CCGGAGCGTC GAGAAGACGG TCGAACTTGA CTATCTAGAG GAAGTAAAAG TCGTAACAAG GTTTCCGTAG GTGAACCTGC GGAAGGATCA TTACCGGGGC CGAGGCCGGG
DQ018752                      CCGGAGCGTC GAGAAGACGG TCGAACTTGA CTATCTAGAG GAAGTAAAAG TCGTAACAAG GTTTCCGTAG GTGAACCTGC GGAAGGATCA TTACCGGGGC CGAGGCCGGG
DQ018757                      ---------- ---------- ---------- ---------- ---------- ---------- ---------- ---------- ---------- ---------- ----------
DQ112354                      ---------- ---------- ---------- ---------- ---------- ---------- ---------- ---------- ---------- ---------- ----------
FM165414                      CCGGAGCGTC GAGAAGACGG TCGAACTTGA CTATCTAGAG GAAGTAAAAG TCGTAACAAG GTTTCCGTAG GTGAACCTGC GGAAGGATCA ---------- ----------
FM165415                      ---------- ---------- ---------- ---------- ---------- ---------- ---------- ---------- ---------- ---------- ----------
AADN03000430                  CCGGAGCGTC GAGAAGACGG TCGAACTTGA CTATCTAGAG GAAGTAAAAG TCGTAACAAG GTTTCCGTAG GTGAACCTGC GGAAGGATCA TTACCGGGGC CGAGGCCGGG
```

```
AADN03001784                ---------- ---------- ---------- ---------- ---------- ---------- ---------- ---------- ---------- ---------- ----------
AADN03001783                ---------- ---------- ---------- ---------- ---------- ---------- ---------- ---------- ---------- ---------- ----------
DQ018754                    CCGGAGCGTC GAGAAGACGG TCGAACTTGA CTATCTAGAG GAAGTAAAAG TCGTAACAAG GTTTCCGTAG GTGAACCTGC GGAAGGATCA TTACCGGGGC CGAGGCCGGG
AADN03001782                ---------- ---------- ---------- ---------- ---------- ---------- ---------- ---------- ---------- ---------- ----------
JN639848                    ---------- ---------- ---------- ---------- ---------- ---------- ---------- ---------- ---------- ---------- ----------
AADN03001670                ---------- ---------- ---------- ---------- ---------- ---------- ---------- ---------- ---------- ---------- ----------
HQ873432                    CCGGAGCGTC GAGAAGACGG TCGAACTTGA CTATCTAGAG GAAGTAAAAG TCGTAACAAG GTTTCCGTAG GTGAACCTGC GGAAGGATCA ---------- ----------
AADN03001774                ---------- ---------- ---------- ---------- ---------- ---------- ---------- ---------- ---------- ---------- ----------
AADN03001775                ---------- ---------- ---------- ---------- ---------- ---------- ---------- ---------- ---------- ---------- ----------
EF552813                    ---------- ---------- ---------- ---------- ---------- ---------- ---------- ---------- ---------- ---------- ----------
AADN03022685                ---------- ---------- ---------- ---------- ---------- ---------- ---------- ---------- ---------- ---------- ----------
AADN03019346                ---------- ---------- ---------- ---------- ---------- ---------- ---------- ---------- ---------- ---------- ----------
AADN03001776                ---------- ---------- ---------- ---------- ---------- ---------- ---------- ---------- ---------- ---------- ----------
DQ018756                    ---------- ---------- ---------- ---------- ---------- ---------- ---------- ---------- ---------- ---------- ----------
AF173612                    CCGGAGCGTC GAGAAGACGG TCGAACTTGA CTATCTAGAG GAAGTAAA-- ---------- ---------- ---------- ---------- ---------- ----------
Gallus_gallus_KT445934.2    CCGGAGCGTC GAGAAGACGG TCGAACTTGA CTATCTAGAG GAAGTAAAAG TCGTAACAAG GTTTCCGTAG GTGAACCTGC GGAAGGATCA TTACCGGGGC CGAGGCCGGG

                            3333333333 3333333333 3333333333 3333333333 3333333333 3333333333 3333333333 3333333333 3333333333 3333333333 3333333333
                            7777777777 7777777777 7777777777 7777777777 7777777777 7777777778 8888888888 8888888888 8888888888 8888888888 8888888888
                            4444444445 5555555556 6666666667 7777777778 8888888889 9999999990 0000000001 1111111112 2222222223 3333333334 4444444445
                            1234567890 1234567890 1234567890 1234567890 1234567890 1234567890 1234567890 1234567890 1234567890 1234567890 1234567890
AADN03001677                ---------- ---------- ---------- ---------- ---------- ---------- ---------- ---------- ---------- ---------- ----------
AADN03001778                ---------- ---------- ---------- ---------- ---------- ---------- ---------- ---------- ---------- ---------- ----------
AADN03001785                ---------- ---------- ---------- ---------- ---------- ---------- ---------- ---------- ---------- ---------- ----------
AADN03001786                ---------- ---------- ---------- ---------- ---------- ---------- ---------- ---------- ---------- ---------- ----------
AADN03001788                ---------- ---------- ---------- ---------- ---------- ---------- ---------- ---------- ---------- ---------- ----------
AADN03014081                ---------- ---------- ---------- ---------- ---------- ---------- ---------- ---------- ---------- ---------- ----------
AADN03015064                ---------- ---------- ---------- ---------- ---------- ---------- ---------- ---------- ---------- ---------- ----------
AADN03026634                CGTCCGGCCG AGCCGTGGCA CGAGCGCGCG CGGGCGCGCA GCCTTCC--- ---------- ---------- ---------- ---------- ---------- ----------
NW_003775878                CGTCCGGCCG AGCCGTGGCA CGAGCGCGCG CGGGCGCGCA GCCTTCC--- ---------- ---------- ---------- ---------- ---------- ----------
DQ018752                    CGTCCGGCCG AGCCGTGGCA CGAGCGCGCG CGGGCGCGCA GCCTTCCCTT CCCTTCCCCG AGCCCGCTCC GCGCGGAGCG CGGCTCCTCT CCCCCGGTCG AAACGGGGAA
DQ018757                    ---------- ---------- ---------- ---------- ---------- ---------- ---------- ---------- ---------- ---------- ----------
DQ112354                    ---------- ---------- ---------- ---------- ---------- ---------- ---------- ---------- ---------- ---------- ----------
FM165414                    ---------- ---------- ---------- ---------- ---------- ---------- ---------- ---------- ---------- ---------- ----------
FM165415                    ---------- ---------- ---------- ---------- ---------- ---------- ---------- ---------- ---------- ---------- ----------
AADN03000430                CGTCCGGCCG AGCCGTGGCA CGAGCGCGCG CGGGCGCGCA GCCTTCCCTT CCCTTCCCCG AGCCCGCTCC GCGCGGAGCG CGGCTCCTCT CCCCCGGTCG AAACGGGGAA
AADN03001784                ---------- ---------- ---------- ---------- ---------- ---------- ---------- ---------- ---------- ---------- ----------
AADN03001783                ---------- ---------- ---------- ---------- ---------- ---------- ---------- ---------- ---------- ---------- ----------
DQ018754                    CGTCCGGCCG AGCCGTGGCA CGAGCGCGCG CGGGCGCGCA GCCTTCCCTT CCC-----CG AGCCCGCTCC GCGCGGAGCG CGGCTCCTCC CCCCCGGTCG AAACGGGGAA
AADN03001782                ---------- ---------- ---------- ---------- ---------- ---------- ---------- ---------- ---------- ---------- ----------
JN639848                    ---------- ---------- ---------- ---------- ---------- ---------- ---------- ---------- ---------- ---------- ----------
AADN03001670                ---------- ---------- ---------- ---------- ---------- ---------- ---------- ---------- ---------- ---------- ----------
HQ873432                    ---------- ---------- ---------- ---------- ---------- ---------- ---------- ---------- ---------- ---------- ----------
AADN03001774                ---------- ---------- ---------- ---------- ---------- ---------- ---------- ---------- ---------- ---------- ----------
AADN03001775                ---------- ---------- ---------- ---------- ---------- ---------- ---------- ---------- ---------- ---------- ----------
EF552813                    ---------- ---------- ---------- ---------- ---------- ---------- ---------- ---------- ---------- ---------- ----------
AADN03022685                ---------- ---------- ---------- ---------- ---------- ---------- ---------- ---------- ---------- ---------- ----------
AADN03019346                ---------- ---------- ---------- ---------- ---------- ---------- ---------- ---------- ---------- ---------- ----------
AADN03001776                ---------- ---------- ---------- ---------- ---------- ---------- ---------- ---------- ---------- ---------- ----------
DQ018756                    ---------- ---------- ---------- ---------- ---------- ---------- ---------- ---------- ---------- ---------- ----------
AF173612                    ---------- ---------- ---------- ---------- ---------- ---------- ---------- ---------- ---------- ---------- ----------
Gallus_gallus_KT445934.2    CGTCCGGCCG AGCCGTGGCA CGAGCGCGCG CGGGCGCGCA GCCTTCCCTT CCCTTCCCCG AGCCCGCTCC GCGCGGAGCG CGGCTCCTCT CCCCCGGTCG AAACGGGGAA
```

```
                              3333333333 3333333333 3333333333 3333333333 3333333333 3333333333 3333333333 3333333333 3333333333 3333333333 3333333333
                              8888888888 8888888888 8888888888 8888888888 8888888889 9999999999 9999999999 9999999999 9999999999 9999999999 9999999999
                              5555555556 6666666667 7777777778 8888888889 9999999990 0000000001 1111111112 2222222223 3333333334 4444444445 5555555556
                              1234567890 1234567890 1234567890 1234567890 1234567890 1234567890 1234567890 1234567890 1234567890 1234567890 1234567890
AADN03001677                  ---------- ---------- ---------- ---------- ---------- ---------- ---------- ---------- ---------- ---------- ----------
AADN03001778                  ---------- ---------- ---------- ---------- ---------- ---------- ---------- ---------- ---------- ---------- ----------
AADN03001785                  ---------- ---------- ---------- ---------- ---------- ---------- ---------- ---------- ---------- ---------- ----------
AADN03001786                  ---------- ---------- ---------- ---------- ---------- ---------- ---------- ---------- ---------- ---------- ----------
AADN03001788                  ---------- ---------- ---------- ---------- ---------- ---------- ---------- ---------- ---------- ---------- ----------
AADN03014081                  ---------- ---------- ---------- ---------- ---------- ---------- ---------- ---------- ---------- ---------- ----------
AADN03015064                  ---------- ---------- ---------- ---------- ---------- ---------- ---------- ---------- ---------- ---------- ----------
AADN03026634                  ---------- ---------- ---------- ---------- ---------- ---------- ---------- ---------- ---------- ---------- ----------
NW_003775878                  ---------- ---------- ---------- ---------- ---------- ---------- ---------- ---------- ---------- ---------- ----------
DQ018752                      AGAAAAAAAA AA-------- ---------- ---------- ---------- ---------- ---------- ---------- ---------- ---------- ----------
DQ018757                      ---------- ---------- ---------- ---------- ---------- ---------- ---------- ---------- ---------- ---------- ----------
DQ112354                      ---------- ---------- ---------- ---------- ---------- ---------- ---------- ---------- ---------- ---------- ----------
FM165414                      ---------- ---------- ---------- ---------- ---------- ---------- ---------- ---------- ---------- ---------- ----------
FM165415                      ---------- ---------- ---------- ---------- ---------- ---------- ---------- ---------- ---------- ---------- ----------
AADN03000430                  AGAAAAAAAA AACACCGCAA GTCGCTCCGC GCGCCTGCCG GCGAGAGAGA AGGGAGACGA GGGCGCGGAG CGCAGCTCCG GGGGGGGAGG CGCGTGTGGG GCGCTCCGGC
AADN03001784                  ---------- ---------- ---------- ---------- ---------- ---------- ---------- ---------- ---------- ---------- ----------
AADN03001783                  ---------- ---------- ---------- ---------- ---------- ---------- ---------- ---------- ---------- ---------- ----------
DQ018754                      AGAAAAAAAA -CCACCGCAA GTCGCTCCGC GCGCCTGCCG GCGAGAGAGA AGGGAGACGA GGGCGCGGAG CGCAGCTCCG GGGGGGGAGG CGCGTGTGGG GCGCGC----
AADN03001782                  ---------- ---------- ---------- ---------- ---------- ---------- ---------- ---------- ---------- ---------- ----------
JN639848                      ---------- ---------- ---------- ---------- ---------- ---------- ---------- ---------- ---------- ---------- ----------
AADN03001670                  ---------- ---------- ---------- ---------- ---------- ---------- ---------- ---------- ---------- ---------- ----------
HQ873432                      ---------- ---------- ---------- ---------- ---------- ---------- ---------- ---------- ---------- ---------- ----------
AADN03001774                  ---------- ---------- ---------- ---------- ---------- ---------- ---------- ---------- ---------- ---------- ----------
AADN03001775                  ---------- ---------- ---------- ---------- ---------- ---------- ---------- ---------- ---------- ---------- ----------
EF552813                      ---------- ---------- ---------- ---------- ---------- ---------- ---------- ---------- ---------- ---------- ----------
AADN03022685                  ---------- ---------- ---------- ---------- ---------- ---------- ---------- ---------- ---------- ---------- ----------
AADN03019346                  ---------- ---------- ---------- ---------- ---------- ---------- ---------- ---------- ---------- ---------- ----------
AADN03001776                  ---------- ---------- ---------- ---------- ---------- ---------- ---------- ---------- ---------- ---------- ----------
DQ018756                      ---------- ---------- ---------- ---------- ---------- ---------- ---------- ---------- ---------- ---------- ----------
AF173612                      ---------- ---------- ---------- ---------- ---------- ---------- ---------- ---------- ---------- ---------- ----------
Gallus_gallus_KT445934.2      AGAAAAAAAA AACACCGCAA GTCGCTCCGC GCGCCTGCCG GCGAGAGAGA AGGGAGACGA GGGCGCGGAG CGCAGCTCCG GGGGGGGAGG CGCGTGTGGG GCGCGC----

                              3333333333 3333333333 3333333333 3333333334 4444444444 4444444444 4444444444 4444444444 4444444444 4444444444 4444444444
                              9999999999 9999999999 9999999999 9999999990 0000000000 0000000000 0000000000 0000000000 0000000000 0000000000 0000000000
                              6666666667 7777777778 8888888889 9999999990 0000000001 1111111112 2222222223 3333333334 4444444445 5555555556 6666666667
                              1234567890 1234567890 1234567890 1234567890 1234567890 1234567890 1234567890 1234567890 1234567890 1234567890 1234567890
AADN03001677                  ---------- ---------- ---------- ---------- ---------- ---------- ---------- ---------- ---------- ---------- ----------
AADN03001778                  ---------- ---------- ---------- ---------- ---------- ---------- ---------- ---------- ---------- ---------- ----------
AADN03001785                  ---------- ---------- ---------- ---------- ---------- ---------- ---------- ---------- ---------- ---------- ----------
AADN03001786                  ---------- ---------- ---------- ---------- ---------- ---------- ---------- ---------- ---------- ---------- ----------
AADN03001788                  ---------- ---------- ---------- ---------- ---------- ---------- ---------- ---------- ---------- ---------- ----------
AADN03014081                  ---------- ---------- ---------- ---------- ---------- ---------- ---------- ---------- ---------- ---------- ----------
AADN03015064                  ---------- ---------- ---------- ---------- ---------- ---------- ---------- ---------- ---------- ---------- ----------
AADN03026634                  ---------- ---------- ---------- ---------- ---------- ---------- ---------- ---------- ---------- ---------- ----------
NW_003775878                  ---------- ---------- ---------- ---------- ---------- ---------- ---------- ---------- ---------- ---------- ----------
DQ018752                      ---------- ---------- ---------- ---------- ---------- ---------- ---------- ---------- ---------- ---------- ----------
DQ018757                      ---------- ---------- ---------- ---------- ---------- ---------- ---------- ---------- ---------- ---------- ----------
DQ112354                      ---------- ---------- ---------- ---------- ---------- ---------- ---------- ---------- ---------- ---------- ----------
FM165414                      ---------- ---------- ---------- ---------- ---------- ---------- ---------- ---------- ---------- ---------- ----------
FM165415                      ---------- ---------- ---------- ---------- ---------- ---------- ---------- ---------- ---------- ---------- ----------
AADN03000430                  GCTCCGGCGC GTCTCTCCCC CCC-GGCGCC GGTCCGCCGT CGGTCCGCAC GCCGCGGGTC CGGTCCGTCC GGTCGCCTCG CCGGCGCGCG CCCGCGCGCG CGCGTCCCGC
```

```
AADN03001784                ---------- ---------- ---------- ---------- ---------- ---------- ---------- ---------- ---------- ---------- ----------
AADN03001783                ---------- ---------- ---------- ---------- ---------- ---------- ---------- ---------- ---------- ---------- ----------
DQ018754                    GCTCCGGCGC GTCTCTCCCC CCCCGGCGCC GGTCCGCCGT CGGTCCGCAC GCCGCGGGTC CGGTCCGTCC GGTCGCCTCG CCGGCGCGCG CCCGCGCGCG CGCGTCCCGC
AADN03001782                ---------- ---------- ---------- ---------- ---------- ---------- ---------- ---------- ---------- ---------- ----------
JN639848                    ---------- ---------- ---------- ---------- ---------- ---------- ---------- ---------- ---------- ---------- ----------
AADN03001670                ---------- ---------- ---------- ---------- ---------- ---------- ---------- ---------- ---------- ---------- ----------
HQ873432                    ---------- ---------- ---------- ---------- ---------- ---------- ---------- ---------- ---------- ---------- ----------
AADN03001774                ---------- ---------- ---------- ---------- ---------- ---------- ---------- ---------- ---------- ---------- ----------
AADN03001775                ---------- ---------- ---------- ---------- ---------- ---------- ---------- ---------- ---------- ---------- ----------
EF552813                    ---------- ---------- ---------- ---------- ---------- ---------- ---------- ---------- ---------- ---------- ----------
AADN03022685                ---------- ---------- ---------- ---------- ---------- ---------- ---------- ---------- ---------- ---------- ----------
AADN03019346                ---------- ---------- ---------- ---------- ---------- ---------- ---------- ---------- ---------- ---------- ----------
AADN03001776                ---------- ---------- ---------- ---------- ---------- ---------- ---------- ---------- ---------- ---------- ----------
DQ018756                    ---------- ---------- ---------- ---------- ---------- ---------- ---------- ---------- ---------- ---------- ----------
AF173612                    ---------- ---------- ---------- ---------- ---------- ---------- ---------- ---------- ---------- ---------- ----------
Gallus_gallus_KT445934.2    GCTCCGGCGC GTCTCTCCCC CCCCGGCGCC GGTCCGCCGT CGGTCCGCAC GCCGCGGGTC CGGTCCGTCC GGTCGCCTCG CCGGCGCGCG CCCGCGCGCG CGCGTCCCGC

                            4444444444 4444444444 4444444444 4444444444 4444444444 4444444444 4444444444 4444444444 4444444444 4444444444 4444444444
                            0000000000 0000000000 0000000001 1111111111 1111111111 1111111111 1111111111 1111111111 1111111111 1111111111 1111111111
                            7777777778 8888888889 9999999990 0000000001 1111111112 2222222223 3333333334 4444444445 5555555556 6666666667 7777777778
                            1234567890 1234567890 1234567890 1234567890 1234567890 1234567890 1234567890 1234567890 1234567890 1234567890 1234567890
AADN03001677                ---------- ---------- ---------- ---------- ---------- ---------- ---------- ---------- ---------- ---------- ----------
AADN03001778                ---------- ---------- ---------- ---------- ---------- ---------- ---------- ---------- ---------- ---------- ----------
AADN03001785                ---------- ---------- ---------- ---------- ---------- ---------- ---------- ---------- ---------- ---------- ----------
AADN03001786                ---------- ---------- ---------- ---------- ---------- ---------- ---------- ---------- ---------- ---------- ----------
AADN03001788                ---------- ---------- ---------- ---------- ---------- ---------- ---------- ---------- ---------- ---------- ----------
AADN03014081                ---------- ---------- ---------- ---------- ---------- ---------- ---------- ---------- ---------- ---------- ----------
AADN03015064                ---------- ---------- ---------- ---------- ---------- ---------- ---------- ---------- ---------- ---------- ----------
AADN03026634                ---------- ---------- ---------- ---------- ---------- ---------- ---------- ---------- ---------- ---------- ----------
NW_003775878                ---------- ---------- ---------- ---------- ---------- ---------- ---------- ---------- ---------- ---------- ----------
DQ018752                    ---------- ---------- ---------- ---------- ---------- ---------- ---------- ---------- ---------- ---------- ----------
DQ018757                    ---------- ---------- ---------- ---------- ---------- ---------- ---------- ---------- ---------- ---------- ----------
DQ112354                    ---------- ---------- ---------- ---------- ---------- ---------- ---------- ---------- ---------- ---------- ----------
FM165414                    ---------- ---------- ---------- ---------- ---------- ---------- ---------- ---------- ---------- ---------- ----------
FM165415                    ---------- ---------- ---------- ---------- ---------- ---------- ---------- ---------- ---------- ---------- ----------
AADN03000430                GGGCCTCGCC CGGGTCGCCG CGCTCCGGAG CGTCCCGCGG CCGAGTCCCG CTCCGACCGC GGGGTCGGGG TCGGGAGGTG GCGGCGGTGC GGAGGGTGGA AGGACGGCTC
AADN03001784                ---------- ---------- ---------- ---------- ---------- ---------- ---------- ---------- ---------- ---------- ----------
AADN03001783                ---------- ---------- ---------- ---------- ---------- ---------- ---------- ---------- ---------- ---------- ----------
DQ018754                    GGGCCTCGCC CCGGCCGCCG CGCTCCGGAG CGTCCCGCGG CCGAGTCCCG CTCCGACCGC GGGGTCGGGG TCGGGAGGTG GCGGCGGTGC GGAGGGTGGA AGGACGGCTC
AADN03001782                ---------- ---------- ---------- ---------- ---------- ---------- ---------- ---------- ---------- ---------- ----------
JN639848                    ---------- ---------- ---------- ---------- ---------- ---------- ---------- ---------- ---------- ---------- ----------
AADN03001670                ---------- ---------- ---------- ---------- ---------- ---------- ---------- ---------- ---------- ---------- ----------
HQ873432                    ---------- ---------- ---------- ---------- ---------- ---------- ---------- ---------- ---------- ---------- ----------
AADN03001774                ---------- ---------- ---------- ---------- ---------- ---------- ---------- ---------- ---------- ---------- ----------
AADN03001775                ---------- ---------- ---------- ---------- ---------- ---------- ---------- ---------- ---------- ---------- ----------
EF552813                    ---------- ---------- ---------- ---------- ---------- ---------- ---------- ---------- ---------- ---------- ----------
AADN03022685                ---------- ---------- ---------- ---------- ---------- ---------- ---------- ---------- ---------- ---------- ----------
AADN03019346                ---------- ---------- ---------- ---------- ---------- ---------- ---------- ---------- ---------- ---------- ----------
AADN03001776                ---------- ---------- ---------- ---------- ---------- ---------- ---------- ---------- ---------- ---------- ----------
DQ018756                    ---------- ---------- ---------- ---------- ---------- ---------- ---------- ---------- ---------- ---------- ----------
AF173612                    ---------- ---------- ---------- ---------- ---------- ---------- ---------- ---------- ---------- ---------- ----------
Gallus_gallus_KT445934.2    GGGCCTCGCC CGGGTCGCCG CGCTCCGGAG CGTCCCGCGG CCGAGTCCCG CTCCGACCGC GGGGTCGGGG TCGGGAGGTG GCGGCGGTGC GGAGGGTGGA AGGACGGCTC
```

```
                              4444444444 4444444444 4444444444 4444444444 4444444444 4444444444 4444444444 4444444444 4444444444 4444444444 4444444444
                              1111111111 1111111112 2222222222 2222222222 2222222222 2222222222 2222222222 2222222222 2222222222 2222222222 2222222222
                              8888888889 9999999990 0000000001 1111111112 2222222223 3333333334 4444444445 5555555556 6666666667 7777777778 8888888889
                              1234567890 1234567890 1234567890 1234567890 1234567890 1234567890 1234567890 1234567890 1234567890 1234567890 1234567890
AADN03001677                  ---------- ---------- ---------- ---------- ---------- ---------- ---------- ---------- ---------- ---------- ----------
AADN03001778                  ---------- ---------- ---------- ---------- ---------- ---------- ---------- ---------- ---------- ---------- ----------
AADN03001785                  ---------- ---------- ---------- ---------- ---------- ---------- ---------- ---------- ---------- ---------- ----------
AADN03001786                  ---------- ---------- ---------- ---------- ---------- ---------- ---------- ---------- ---------- ---------- ----------
AADN03001788                  ---------- ---------- ---------- ---------- ---------- ---------- ---------- ---------- ---------- ---------- ----------
AADN03014081                  ---------- ---------- ---------- ---------- ---------- ---------- ---------- ---------- ---------- ---------- ----------
AADN03015064                  ---------- ---------- ---------- ---------- ---------- ---------- ---------- ---------- ---------- ---------- ----------
AADN03026634                  ---------- ---------- ---------- ---------- ---------- ---------- ---------- ---------- ---------- ---------- ----------
NW_003775878                  ---------- ---------- ---------- ---------- ---------- ---------- ---------- ---------- ---------- ---------- ----------
DQ018752                      ---------- ---------- ---------- ---------- ---------- ---------- ---------- ---------- ---------- ---------- ----------
DQ018757                      ---------- ---------- ---------- ---------- ---------- ---------- ---------- ---------- ---------- ---------- ----------
DQ112354                      ---------- ---------- ---------- ---------- ---------- ---------- ---------- ---------- ---------- ---------- ----------
FM165414                      ---------- ---------- ---------- ---------- ---------- ---------- ---------- ---------- ---------- ---------- ----------
FM165415                      ---------- ---------- ---------- ---------- ---------- ---------- ---------- ---------- ---------- ---------- ----------
AADN03000430                  CCCGCTTCGT CGCTCGGCCG GAAACTCGCC ACCGGCCCCC GCCGCTGTCG ACGCCGGCAC CCCGAGTCCG CTCGGAGGGA AGCCGCGCGG GCGGCCGCGC GCGGGGGAGG
AADN03001784                  ---------- ---------- ---------- ---------- ---------- ---------- ---------- ---------- ---------- ---------- ----------
AADN03001783                  ---------- ---------- ---------- ---------- ---------- ---------- ---------- ---------- ---------- ---------- ----------
DQ018754                      CCCGCTTCGT CGCTCGGCCG GAAACTCGCC ACCGGCCCTC GCCGCTGTCG ACGCCGGCAC CCCGAGTCCG CTCGGAGGGA AGCCGCGCGG GCGGCCGCGC GCGGGGGAGG
AADN03001782                  ---------- ---------- ---------- ---------- ---------- ---------- ---------- ---------- ---------- ---------- ----------
JN639848                      ---------- ---------- ---------- ---------- ---------- ---------- ---------- ---------- ---------- ---------- ----------
AADN03001670                  ---------- ---------- ---------- ---------- ---------- ---------- ---------- ---------- ---------- ---------- ----------
HQ873432                      ---------- ---------- ---------- ---------- ---------- ---------- ---------- ---------- ---------- ---------- ----------
AADN03001774                  ---------- ---------- ---------- ---------- ---------- ---------- ---------- ---------- ---------- ---------- ----------
AADN03001775                  ---------- ---------- ---------- ---------- ---------- ---------- ---------- ---------- ---------- ---------- ----------
EF552813                      ---------- ---------- ---------- ---------- ---------- ---------- ---------- ---------- ---------- ---------- ----------
AADN03022685                  ---------- ---------- ---------- ---------- ---------- ---------- ---------- ---------- ---------- ---------- ----------
AADN03019346                  ---------- ---------- ---------- ---------- ---------- ---------- ---------- ---------- ---------- ---------- ----------
AADN03001776                  ---------- ---------- ---------- ---------- ---------- ---------- ---------- ---------- ---------- ---------- ----------
DQ018756                      ---------- ---------- ---------- ---------- ---------- ---------- ---------- ---------- ---------- ---------- ----------
AF173612                      ---------- ---------- ---------- ---------- ---------- ---------- ---------- ---------- ---------- ---------- ----------
Gallus_gallus_KT445934.2      CCCGCTTCGT CGCTCGGCCG GAAACTCGCC ACCCGCCCCC GCCGCTGTCG ACGCCGGCAC CCCGAGTCCG CTCGGAGGGA AGCCGCGCGG GCGGCCGCGC GCGGGGGAGG

                              4444444444 4444444444 4444444444 4444444444 4444444444 4444444444 4444444444 4444444444 4444444444 4444444444 4444444444
                              2222222223 3333333333 3333333333 3333333333 3333333333 3333333333 3333333333 3333333333 3333333333 3333333333 3333333334
                              9999999990 0000000001 1111111112 2222222223 3333333334 4444444445 5555555556 6666666667 7777777778 8888888889 9999999990
                              1234567890 1234567890 1234567890 1234567890 1234567890 1234567890 1234567890 1234567890 1234567890 1234567890 1234567890
AADN03001677                  ---------- ---------- ---------- ---------- ---------- ---------- ---------- ---------- ---------- ---------- ----------
AADN03001778                  ---------- ---------- ---------- ---------- ---------- ---------- ---------- ---------- ---------- ---------- ----------
AADN03001785                  ---------- ---------- ---------- ---------- ---------- ---------- ---------- ---------- ---------- ---------- ----------
AADN03001786                  ---------- ---------- ---------- ---------- ---------- ---------- ---------- ---------- ---------- ---------- ----------
AADN03001788                  ---------- ---------- ---------- ---------- ---------- ---------- ---------- ---------- ---------- ---------- ----------
AADN03014081                  ---------- ---------- ---------- ---------- ---------- ---------- ---------- ---------- ---------- ---------- ----------
AADN03015064                  ---------- ---------- ---------- ---------- ---------- ---------- ---------- ---------- ---------- ---------- ----------
AADN03026634                  ---------- ---------- ---------- ---------- ---------- ---------- ---------- ---------- ---------- ---------- ----------
NW_003775878                  ---------- ---------- ---------- ---------- ---------- ---------- ---------- ---------- ---------- ---------- ----------
DQ018752                      ---------- ---------- ---------- ---------- ---------- ---------- ---------- ---------- ---------- ---------- ----------
DQ018757                      ---------- ---------- ---------- ---------- ---------- ---------- ---------- ---------- ---------- ---------- ----------
DQ112354                      ---------- ---------- ---------- ---------- ---------- ---------- ---------- ---------- ---------- ---------- ----------
FM165414                      ---------- ---------- ---------- ---------- ---------- ---------- ---------- ---------- ---------- ---------- ----------
FM165415                      ---------- ---------- ---------- ---------- ---------- ---------- ---------- ---------- ---------- ---------- ----------
AADN03000430                  CGGCGGGCGG CGGGTCCGAG CGCGGGGCGC GGGAAGTCGG CCGCTTCCCC CGGCCTCACC CCCCACCCCC TTCGCCCGGC CCGTCGCGGG GACGGGGCCG GGTCGCGGGC
```

```
AADN03001784                ---------- ---------- ---------- ---------- ---------- ---------- ---------- ---------- ---------- ---------- ----------
AADN03001783                ---------- ---------- ---------- ---------- ---------- ---------- ---------- ---------- ---------- ---------- ----------
DQ018754                    CGGCGGGCGG CGGGTCCGAG CGCGGGGCGC GGGAAGTCGG CCGCTTCCCC CGGCCTCACC CCCCACCCCC TTCGCCCGGC CCGTCGCGGG GACGGGGCCG GGTCGCGGGC
AADN03001782                ---------- ---------- ---------- ---------- ---------- ---------- ---------- ---------- ---------- ---------- ----------
JN639848                    ---------- ---------- ---------- ---------- ---------- ---------- ---------- ---------- ---------- ---------- ----------
AADN03001670                ---------- ---------- ---------- ---------- ---------- ---------- ---------- ---------- ---------- ---------- ----------
HQ873432                    ---------- ---------- ---------- ---------- ---------- ---------- ---------- ---------- ---------- ---------- ----------
AADN03001774                ---------- ---------- ---------- ---------- ---------- ---------- ---------- ---------- ---------- ---------- ----------
AADN03001775                ---------- ---------- ---------- ---------- ---------- ---------- ---------- ---------- ---------- ---------- ----------
EF552813                    ---------- ---------- ---------- ---------- ---------- ---------- ---------- ---------- ---------- ---------- ----------
AADN03022685                ---------- ---------- ---------- ---------- ---------- ---------- ---------- ---------- ---------- ---------- ----------
AADN03019346                ---------- ---------- ---------- ---------- ---------- ---------- ---------- ---------- ---------- ---------- ----------
AADN03001776                ---------- ---------- ---------- ---------- ---------- ---------- ---------- ---------- ---------- ---------- ----------
DQ018756                    ---------- ---------- ---------- ---------- ---------- ---------- ---------- ---------- ---------- ---------- ----------
AF173612                    ---------- ---------- ---------- ---------- ---------- ---------- ---------- ---------- ---------- ---------- ----------
Gallus_gallus_KT445934.2    CGGCGGGCGG CGGGTCCGAG CGCGGGGCGC GGGAAGTCGG CCGCTTCCCC CGGCCTCACC CCCCACCCCC TTCGCCCGGC CCGTCGCGGG GACGGGGCCG GGTCGCGGGC

                            4444444444 4444444444 4444444444 4444444444 4444444444 4444444444 4444444444 4444444444 4444444444 4444444444 4444444444
                            4444444444 4444444444 4444444444 4444444444 4444444444 4444444444 4444444444 4444444444 4444444444 4444444445 5555555555
                            0000000001 1111111112 2222222223 3333333334 4444444445 5555555556 6666666667 7777777778 8888888889 9999999990 0000000001
                            1234567890 1234567890 1234567890 1234567890 1234567890 1234567890 1234567890 1234567890 1234567890 1234567890 1234567890
AADN03001677                ---------- ---------- ---------- ---------- ---------- ---------- ---------- ---------- ---------- ---------- ----------
AADN03001778                ---------- ---------- ---------- ---------- ---------- ---------- ---------- ---------- ---------- ---------- ----------
AADN03001785                ---------- ---------- ---------- ---------- ---------- ---------- ---------- ---------- ---------- ---------- ----------
AADN03001786                ---------- ---------- ---------- ---------- ---------- ---------- ---------- ---------- ---------- ---------- ----------
AADN03001788                ---------- ---------- ---------- ---------- ---------- ---------- ---------- ---------- ---------- ---------- ----------
AADN03014081                ---------- ---------- ---------- ---------- ---------- ---------- ---------- ---------- ---------- ---------- ----------
AADN03015064                ---------- ---------- ---------- ---------- ---------- ---------- ---------- ---------- ---------- ---------- ----------
AADN03026634                ---------- ---------- ---------- ---------- ---------- ---------- ---------- ---------- ---------- ---------- ----------
NW_003775878                ---------- ---------- ---------- ---------- ---------- ---------- ---------- ---------- ---------- ---------- ----------
DQ018752                    ---------- ---------- ---------- ---------- ---------- ---------- ---------- ---------- ---------- ---------- ----------
DQ018757                    ---------- ---------- ---------- ---------- ---------- ---------- ---------- ---------- ---------- ---------- ----------
DQ112354                    ---------- ---------- ---------- ---------- ---------- ---------- ---------- ---------- ---------- ---------- ----------
FM165414                    ---------- ---------- ---------- ---------- ---------- ---------- ---------- ---------- ---------- ---------- ----------
FM165415                    ---------- ---------- ---------- ---------- ---------- ---------- ---------- ---------- ---------- ---------- ----------
AADN03000430                GGCTGCGGAG CCGGCCGACT CCGGGCGAGC GCCGGAGGGA CGCGCGCGCC GCGTACGCGC GGCAGGCGCG AGGTGCCCCG GGCGGCTTCG GTCCCGCGCG GGCGGTCCGA
AADN03001784                ---------- ---------- ---------- ---------- ---------- ---------- ---------- ---------- ---------- ---------- ----------
AADN03001783                ---------- ---------- ---------- ---------- ---------- ---------- ---------- ---------- ---------- ---------- ----------
DQ018754                    GGCTGCGGAG CCGGCCGACT CCGGGCGAGC GCCGGAGGGG CGCGCGCGCC GCGTACGCGC GGCAGGCGCG AGGTGCCCCG GGCGGCTTCG GTCCCGCGCG GGCGGTCCGA
AADN03001782                ---------- ---------- ---------- ---------- ---------- ---------- ---------- ---------- ---------- ---------- ----------
JN639848                    ---------- ---------- ---------- ---------- ---------- ---------- ---------- ---------- ---------- ---------- ----------
AADN03001670                ---------- ---------- ---------- ---------- ---------- ---------- ---------- ---------- ---------- ---------- ----------
HQ873432                    ---------- ---------- ---------- ---------- ---------- ---------- ---------- ---------- ---------- ---------- ----------
AADN03001774                ---------- ---------- ---------- ---------- ---------- ---------- ---------- ---------- ---------- ---------- ----------
AADN03001775                ---------- ---------- ---------- ---------- ---------- ---------- ---------- ---------- ---------- ---------- ----------
EF552813                    ---------- ---------- ---------- ---------- ---------- ---------- ---------- ---------- ---------- ---------- ----------
AADN03022685                ---------- ---------- ---------- ---------- ---------- ---------- ---------- ---------- ---------- ---------- ----------
AADN03019346                ---------- ---------- ---------- ---------- ---------- ---------- ---------- ---------- ---------- ---------- ----------
AADN03001776                ---------- ---------- ---------- ---------- ---------- ---------- ---------- ---------- ---------- ---------- ----------
DQ018756                    ---------- ---------- ---------- ---------- ---------- ---------- ---------- ---------- ---------- ---------- ----------
AF173612                    ---------- ---------- ---------- ---------- ---------- ---------- ---------- ---------- ---------- ---------- ----------
Gallus_gallus_KT445934.2    GGCTGCGGAG CCGGCCGACT CCGGGCGAGC GCCGGAGGGA CGCGCGCGCC GCGTACGCGC GGCAGGCGCG AGGTGCCCCG GGCGGCTTCG GTCCCGCGCG GGCGGTCCGA
```

```
                            4444444444 4444444444 4444444444 4444444444 4444444444 4444444444 4444444444 4444444444 4444444444 4444444444 4444444444
                            5555555555 5555555555 5555555555 5555555555 5555555555 5555555555 5555555555 5555555555 5555555556 6666666666 6666666666
                            1111111112 2222222223 3333333334 4444444445 5555555556 6666666667 7777777778 8888888889 9999999990 0000000001 1111111112
                            1234567890 1234567890 1234567890 1234567890 1234567890 1234567890 1234567890 1234567890 1234567890 1234567890 1234567890
AADN03001677                ---------- ---------- ---------- ---------- ---------- ---------- ---------- ---------- ---------- ---------- ----------
AADN03001778                ---------- ---------- ---------- ---------- ---------- ---------- ---------- ---------- ---------- ---------- ----------
AADN03001785                ---------- ---------- ---------- ---------- ---------- ---------- ---------- ---------- ---------- ---------- ----------
AADN03001786                ---------- ---------- ---------- ---------- ---------- ---------- ---------- ---------- ---------- ---------- ----------
AADN03001788                ---------- ---------- ---------- ---------- ---------- ---------- ---------- ---------- ---------- ---------- ----------
AADN03014081                ---------- ---------- ---------- ---------- ---------- ---------- ---------- ---------- ---------- ---------- ----------
AADN03015064                ---------- ---------- ---------- ---------- ---------- ---------- ---------- ---------- ---------- ---------- ----------
AADN03026634                ---------- ---------- ---------- ---------- ---------- ---------- ---------- ---------- ---------- ---------- ----------
NW_003775878                ---------- ---------- ---------- ---------- ---------- ---------- ---------- ---------- ---------- ---------- ----------
DQ018752                    ---------- ---------- ---------- ---------- ---------- ---------- ---------- ---------- ---------- ---------- ----------
DQ018757                    ---------- ---------- ---------- ---------- ---------- ---------- ---------- ---------- ---------- ---------- ----------
DQ112354                    ---------- ---------- ---------- ---------- ---------- ---------- ---------- ---------- ---------- ---------- ----------
FM165414                    ---------- ---------- ---------- ---------- ---------- ---------- ---------- ---------- ---------- ---------- ----------
FM165415                    ---------- ---------- ---------- ---------- ---------- ---------- ---------- ---------- ---------- ---------- ----------
AADN03000430                GCCTCGCGGC TCCTCCCGGG TGCAGCTGCC GCCCGGCGCC GGGTTGCCGA GGGAAACCCC GGGCCCCGGG AGGAACGCGA GGTGGTGGCG GCGGACGTCG GGCGCGCCCC
AADN03001784                ---------- ---------- ---------- ---------- ---------- ---------- ---------- ---------- ---------- ---------- ----------
AADN03001783                ---------- ---------- ---------- ---------- ---------- ---------- ---------- ---------- ---------- ---------- ----------
DQ018754                    GCCTCGCGGC TCCTCCCGGG TGCAGCTGCC GCCCGGCGCC GGGTTGCCGG GGGAAACCCC GGGCCCCGGG AGGAACGCGA GGTGGTGGCG GCGGACGTCG GGCGCGCCCC
AADN03001782                ---------- ---------- ---------- ---------- ---------- ---------- ---------- ---------- ---------- ---------- ----------
JN639848                    ---------- ---------- ---------- ---------- ---------- ---------- ---------- ---------- ---------- ---------- ----------
AADN03001670                ---------- ---------- ---------- ---------- ---------- ---------- ---------- ---------- ---------- ---------- ----------
HQ873432                    ---------- ---------- ---------- ---------- ---------- ---------- ---------- ---------- ---------- ---------- ----------
AADN03001774                ---------- ---------- ---------- ---------- ---------- ---------- ---------- ---------- ---------- ---------- ----------
AADN03001775                ---------- ---------- ---------- ---------- ---------- ---------- ---------- ---------- ---------- ---------- ----------
EF552813                    ---------- ---------- ---------- ---------- ---------- ---------- ---------- ---------- ---------- ---------- ----------
AADN03022685                ---------- ---------- ---------- ---------- ---------- ---------- ---------- ---------- ---------- ---------- ----------
AADN03019346                ---------- ---------- ---------- ---------- ---------- ---------- ---------- ---------- ---------- ---------- ----------
AADN03001776                ---------- ---------- ---------- ---------- ---------- ---------- ---------- ---------- ---------- ---------- ----------
DQ018756                    ---------- ---------- ---------- ---------- ---------- ---------- ---------- ---------- ---------- ---------- ----------
AF173612                    ---------- ---------- ---------- ---------- ---------- ---------- ---------- ---------- ---------- ---------- ----------
Gallus_gallus_KT445934.2    GCCTCGCGGC TCCTCCCGGG TGCAGCTGCC GCCCGGCGCC GGGTTGCCGA GGGAAACCCC GGGCCCCGGG AGGAACGCGA GGTGGTGGCG GCGGACGTCG GGCGCGCCCC

                            4444444444 4444444444 4444444444 4444444444 4444444444 4444444444 4444444444 4444444444 4444444444 4444444444 4444444444
                            6666666666 6666666666 6666666666 6666666666 6666666666 6666666666 6666666666 6666666667 7777777777 7777777777 7777777777
                            2222222223 3333333334 4444444445 5555555556 6666666667 7777777778 8888888889 9999999990 0000000001 1111111112 2222222223
                            1234567890 1234567890 1234567890 1234567890 1234567890 1234567890 1234567890 1234567890 1234567890 1234567890 1234567890
AADN03001677                ---------- ---------- ---------- ---------- ---------- ---------- ---------- ---------- ---------- ---------- ----------
AADN03001778                ---------- ---------- ---------- ---------- ---------- ---------- ---------- ---------- ---------- ---------- ----------
AADN03001785                ---------- ---------- ---------- ---------- ---------- ---------- ---------- ---------- ---------- ---------- ----------
AADN03001786                ---------- ---------- ---------- ---------- ---------- ---------- ---------- ---------- ---------- ---------- ----------
AADN03001788                ---------- ---------- ---------- ---------- ---------- ---------- ---------- ---------- ---------- ---------- ----------
AADN03014081                ---------- ---------- ---------- ---------- ---------- ---------- ---------- ---------- ---------- ---------- ----------
AADN03015064                ---------- ---------- ---------- ---------- ---------- ---------- ---------- ---------- ---------- ---------- ----------
AADN03026634                ---------- ---------- ---------- ---------- ---------- ---------- ---------- ---------- ---------- ---------- ----------
NW_003775878                ---------- ---------- ---------- ---------- ---------- ---------- ---------- ---------- ---------- ---------- ----------
DQ018752                    ---------- ---------- ---------- ---------- ---------- ---------- ---------- ---------- ---------- ---------- ----------
DQ018757                    ---------- ---------- ---------- ---------- ---------- ---------- ---------- ---------- ---------- ---------- ----------
DQ112354                    ---------- ---------- ---------- ---------- ---------- ---------- ---------- ---------- ---------- ---------- ----------
FM165414                    ---------- ---------- ---------- ---------- ---------- ---------- ---------- ---------- ---------- ---------- ----------
FM165415                    ---------- ---------- ---------- ---------- ---------- ---------- ---------- ---------- ---------- ---------- ----------
AADN03000430                CGCGGGCGGA CGCTCCCCCG AGGGGCGCCG GGGCCGGCTG GCGGGTGCCG GGTCTCCCCT CGGCGCCCCG TCCCGCCCCG CCGAGCGGGG CGGGCGGGGG AGGCACCCCC
```

```
AADN03001784              ---------- ---------- ---------- ---------- ---------- ---------- ---------- ---------- ---------- ---------- ----------
AADN03001783              ---------- ---------- ---------- ---------- ---------- ---------- ---------- ---------- ---------- ---------- ----------
DQ018754                  CGCGGGCGGA CGCTCCCCCG AGGGGCGCCG GGGCCGGCTG GCGGGTGCCG GGTCTCCCCT CGGCGCCCCG TCCCGCCCCG CCGAGCGGGG CGGGCGGGGG AGGCACCCCC
AADN03001782              ---------- ---------- ---------- ---------- ---------- ---------- ---------- ---------- ---------- ---------- ----------
JN639848                  ---------- ---------- ---------- ---------- ---------- ---------- ---------- ---------- ---------- ---------- ----------
AADN03001670              ---------- ---------- ---------- ---------- ---------- ---------- ---------- ---------- ---------- ---------- ----------
HQ873432                  ---------- ---------- ---------- ---------- ---------- ---------- ---------- ---------- ---------- ---------- ----------
AADN03001774              ---------- ---------- ---------- ---------- ---------- ---------- ---------- ---------- ---------- ---------- ----------
AADN03001775              ---------- ---------- ---------- ---------- ---------- ---------- ---------- ---------- ---------- ---------- ----------
EF552813                  ---------- ---------- ---------- ---------- ---------- ---------- ---------- ---------- ---------- ---------- ----------
AADN03022685              ---------- ---------- ---------- ---------- ---------- ---------- ---------- ---------- ---------- ---------- ----------
AADN03019346              ---------- ---------- ---------- ---------- ---------- ---------- ---------- ---------- ---------- ---------- ----------
AADN03001776              ---------- ---------- ---------- ---------- ---------- ---------- ---------- ---------- ---------- ---------- ----------
DQ018756                  ---------- ---------- ---------- ---------- ---------- ---------- ---------- ---------- ---------- ---------- ----------
AF173612                  ---------- ---------- ---------- ---------- ---------- ---------- ---------- ---------- ---------- ---------- ----------
Gallus_gallus_KT445934.2  CGCGGGCGGA CGCTCCCCCG AGGGGCGCCG GGGCCGGCTG GCGGGTGCCG GGTCTCCCCT CGGCGCCCCG TCCCGCCCCG CCGAGCGGGG CGGGCGGGGG AGGCACCCCC

                          4444444444 4444444444 4444444444 4444444444 4444444444 4444444444 4444444444 4444444444 4444444444 4444444444 4444444444
                          7777777777 7777777777 7777777777 7777777777 7777777777 7777777777 7777777778 8888888888 8888888888 8888888888 8888888888
                          3333333334 4444444445 5555555556 6666666667 7777777778 8888888889 9999999990 0000000001 1111111112 2222222223 3333333334
                          1234567890 1234567890 1234567890 1234567890 1234567890 1234567890 1234567890 1234567890 1234567890 1234567890 1234567890
AADN03001677              ---------- ---------- ---------- ---------- ---------- ---------- ---------- ---------- ---------- ---------- ----------
AADN03001778              ---------- ---------- ---------- ---------- ---------- ---------- ---------- ---------- ---------- ---------- ----------
AADN03001785              ---------- ---------- ---------- ---------- ---------- ---------- ---------- ---------- ---------- ---------- ----------
AADN03001786              ---------- ---------- ---------- ---------- ---------- ---------- ---------- ---------- ---------- ---------- ----------
AADN03001788              ---------- ---------- ---------- ---------- ---------- ---------- ---------- ---------- ---------- ---------- ----------
AADN03014081              ---------- ---------- ---------- ---------- ---------- ---------- ---------- ---------- ---------- ---------- ----------
AADN03015064              ---------- ---------- ---------- ---------- ---------- ---------- ---------- ---------- ---------- ---------- ----------
AADN03026634              ---------- ---------- ---------- ---------- ---------- ---------- ---------- ---------- ---------- ---------- ----------
NW_003775878              ---------- ---------- ---------- ---------- ---------- ---------- ---------- ---------- ---------- ---------- ----------
DQ018752                  ---------- ---------- ---------- ---------- ---------- ---------- ---------- ---------- ---------- ---------- ----------
DQ018757                  ---------- ---------- ---------- ---------- ---------- ---------- ---------- ---------- ---------- ---------- ----------
DQ112354                  ---------- ---------- ---------- ---------- ---------- ---------- ---------- ---------- ---------- ---------- ----------
FM165414                  ---------- ---------- ---------- ---------- ---------- ---------- ---------- ---------- ---------- ---------- ----------
FM165415                  ---------- ---------- ---------- ---------- ---------- ---------- ---------- ---------- ---------- ---------- ----------
AADN03000430              GCGGGGCCTT CGGGTCGTTT CCCTCACCCC AGGGCCAGGT ACCTAGCGTC CGCGCCTCCG CGCGTCCGGG GGGCGGGGAG GAAGGAGCGC GGCGCCGGTC CCGAGCGGGC
AADN03001784              ---------- ---------- ---------- ---------- ---------- ---------- ---------- ---------- ---------- ---------- ----------
AADN03001783              ---------- ---------- ---------- ---------- ---------- ---------- ---------- ---------- ---------- ---------- ----------
DQ018754                  GCGGGGCCTT CGGGTCGTTT CCCTCACCCC AGGGCCAGGT ACCTAGCGTC CGCGCCTCCG CGCGTCCGGG GGGCGGGGAG GAAGGAGCGC GGCGCCGGTC CCGAGCGGGC
AADN03001782              ---------- ---------- ---------- ---------- ---------- ---------- ---------- ---------- ---------- ---------- ----------
JN639848                  ---------- ---------- ---------- ---------- ---------- ---------- ---------- ---------- ---------- ---------- ----------
AADN03001670              ---------- ---------- ---------- ---------- ---------- ---------- ---------- ---------- ---------- ---------- ----------
HQ873432                  ---------- ---------- ---------- ---------- ---------- ---------- ---------- ---------- ---------- ---------- ----------
AADN03001774              ---------- ---------- ---------- ---------- ---------- ---------- ---------- ---------- ---------- ---------- ----------
AADN03001775              ---------- ---------- ---------- ---------- ---------- ---------- ---------- ---------- ---------- ---------- ----------
EF552813                  ---------- ---------- ---------- ---------- ---------- ---------- ---------- ---------- ---------- ---------- ----------
AADN03022685              ---------- ---------- ---------- ---------- ---------- ---------- ---------- ---------- ---------- ---------- ----------
AADN03019346              ---------- ---------- ---------- ---------- ---------- ---------- ---------- ---------- ---------- ---------- ----------
AADN03001776              ---------- ---------- ---------- ---------- ---------- ---------- ---------- ---------- ---------- ---------- ----------
DQ018756                  ---------- ---------- ---------- ---------- ---------- ---------- ---------- ---------- ---------- ---------- ----------
AF173612                  ---------- ---------- ---------- ---------- ---------- ---------- ---------- ---------- ---------- ---------- ----------
Gallus_gallus_KT445934.2  GCGGGGCCTT CGGGTCGTTT CCCTCACCCC AGGGCCAGGT ACCTAGCGTC CGCGCCTCCG CGCGTCCGGG GGGCGGGGAG GAAGGAGCGC GGCGCCGGTC CCGAGCGGGC
```

```
                              4444444444 4444444444 4444444444 4444444444 4444444444 4444444444 4444444444 4444444444 4444444444 4444444444 4444444444
                              8888888888 8888888888 8888888888 8888888888 8888888888 8888888889 9999999999 9999999999 9999999999 9999999999 9999999999
                              4444444445 5555555556 6666666667 7777777778 8888888889 9999999990 0000000001 1111111112 2222222223 3333333334 4444444445
                              1234567890 1234567890 1234567890 1234567890 1234567890 1234567890 1234567890 1234567890 1234567890 1234567890 1234567890
AADN03001677                  ---------- ---------- ---------- ---------- ---------- ---------- ---------- ---------- ---------- ---------- ----------
AADN03001778                  ---------- ---------- ---------- ---------- ---------- ---------- ---------- ---------- ---------- ---------- ----------
AADN03001785                  ---------- ---------- ---------- ---------- ---------- ---------- ---------- ---------- ---------- ---------- ----------
AADN03001786                  ---------- ---------- ---------- ---------- ---------- ---------- ---------- ---------- ---------- ---------- ----------
AADN03001788                  ---------- ---------- ---------- ---------- ---------- ---------- ---------- ---------- ---------- ---------- ----------
AADN03014081                  ---------- ---------- ---------- ---------- ---------- ---------- ---------- ---------- ---------- ---------- ----------
AADN03015064                  ---------- ---------- ---------- ---------- ---------- ---------- ---------- ---------- ---------- ---------- ----------
AADN03026634                  ---------- ---------- ---------- ---------- ---------- ---------- ---------- ---------- ---------- ---------- ----------
NW_003775878                  ---------- ---------- ---------- ---------- ---------- ---------- ---------- ---------- ---------- ---------- ----------
DQ018752                      ---------- ---------- ---------- ---------- ---------- ---------- ---------- ---------- ---------- ---------- ----------
DQ018757                      ---------- ---------- ---------- ---------- ---------- ---------- ---------- ---------- ---------- ---------- ----------
DQ112354                      ---------- ---------- ---------- ---------- ---------- ---------- ---------- ---------- ---------- ---------- ----------
FM165414                      ---------- ---------- ---------- ---------- ---------- ---------- ---------- ---------- ---------- ---------- ----------
FM165415                      ---------- ---------- ---------- ---------- ---------- ---------- ---------- ---------- ---------- ---------- ----------
AADN03000430                  CGCGTCGCCC ACACCCCCCT CCTCCCCCCG GGCCGCGGAG CCGGGCGGAG GTTTAAAGAC TCGGG-CGGT CCGCTGCGCG CGCCGCGAGG TCGGGGGCCG GGGGCGGTCT
AADN03001784                  ---------- ---------- ---------- ---------- ---------- ---------- ---------- ---------- ---------- ---------- ----------
AADN03001783                  ---------- ---------- ---------- ---------- ---------- ---------- ---------- ---------- ---------- ---------- ----------
DQ018754                      CGCGTCGCCC ACACCCCCCT CCTCCCCCCG GGCCGCGGAG CCGGGCGGAG GTTTAAAGAC TCGGGGCGGC CCGCGGCGCG CGCCGCGAGG TCGGGGGCCG GGGGCGGTCT
AADN03001782                  ---------- ---------- ---------- ---------- ---------- ---------- ---------- ---------- ---------- ---------- ----------
JN639848                      ---------- ---------- ---------- ---------- ---------- ---------- ---------- ---------- ---------- ---------- ----------
AADN03001670                  ---------- ---------- ---------- ---------- ---------- ---------- ---------- ---------- ---------- ---------- ----------
HQ873432                      ---------- ---------- ---------- ---------- ---------- ---------- ---------- ---------- ---------- ---------- ----------
AADN03001774                  ---------- ---------- ---------- ---------- ---------- ---------- ---------- ---------- ---------- ---------- ----------
AADN03001775                  ---------- ---------- ---------- ---------- ---------- ---------- ---------- ---------- ---------- ---------- ----------
EF552813                      ---------- ---------- ---------- ---------- ---------- ---------- ---------- ---------- ---------- ---------- ----------
AADN03022685                  ---------- ---------- ---------- ---------- ---------- ---------- ---------- ---------- ---------- ---------- ----------
AADN03019346                  ---------- ---------- ---------- ---------- ---------- ---------- ---------- ---------- ---------- ---------- ----------
AADN03001776                  ---------- ---------- ---------- ---------- ---------- ---------- ---------- ---------- ---------- ---------- ----------
DQ018756                      ---------- ---------- ---------- ---------- ---------- ---------- ---------- ---------- ---------- ---------- ----------
AF173612                      ---------- ---------- ---------- ---------- ---------- ---------- ---------- ---------- ---------- ---------- ----------
Gallus_gallus_KT445934.2      CGCGTCGCCC ACACCCCCCT CCTCCCCCCG GGCCGCGGAG CCGGGCGGAG GTTTAAAGAC TCGGG-CGGC CCGCGGCGCG CGCCGCGAGG TCGGGGGCCG GGGGCGGTCT

                              4444444444 4444444444 4444444444 4444444444 4444444445 5555555555 5555555555 5555555555 5555555555 5555555555 5555555555
                              9999999999 9999999999 9999999999 9999999999 9999999990 0000000000 0000000000 0000000000 0000000000 0000000000 0000000000
                              5555555556 6666666667 7777777778 8888888889 9999999990 0000000001 1111111112 2222222223 3333333334 4444444445 5555555556
                              1234567890 1234567890 1234567890 1234567890 1234567890 1234567890 1234567890 1234567890 1234567890 1234567890 1234567890
AADN03001677                  ---------- ---------- ---------- ---------- ---------- ---------- ---------- ---------- ---------- ---------- ----------
AADN03001778                  ---------- ---------- ---------- ---------- ---------- ---------- ---------- ---------- ---------- ---------- ----------
AADN03001785                  ---------- ---------- ---------- ---------- ---------- ---------- ---------- ---------- ---------- ---------- ----------
AADN03001786                  ---------- ---------- ---------- ---------- ---------- ---------- ---------- ---------- ---------- ---------- ----------
AADN03001788                  ---------- ---------- ---------- ---------- ---------- ---------- ---------- ---------- ---------- ---------- ----------
AADN03014081                  ---------- ---------- ---------- ---------- ---------- ---------- ---------- ---------- ---------- ---------- ----------
AADN03015064                  ---------- ---------- ---------- ---------- ---------- ---------- ---------- ---------- ---------- ---------- ----------
AADN03026634                  ---------- ---------- ---------- ---------- ---------- ---------- ---------- ---------- ---------- ---------- ----------
NW_003775878                  ---------- ---------- ---------- ---------- ---------- ---------- ---------- ---------- ---------- ---------- ----------
DQ018752                      ---------- ---------- ---------- ---------- ---------- ---------- ---------- ---------- ---------- ---------- ----------
DQ018757                      ---------- ---------- ---------- ---------- ---------- ---------- ---------- ---------- ---------- ---------- ----------
DQ112354                      ---------- ---------- ---------- ---------- ---------- ---------- ---------- ---------- ---------- ---------- ----------
FM165414                      ---------- ---------- ---------- ---------- ---------- ---------- ---------- ---------- ---------- ---------- ----------
FM165415                      ---------- ---------- ---------- ---------- ---------- ---------- ---------- ---------- ---------- ---------- ----------
AADN03000430                  TCTGCCC--- ---------- ---------- ---------- ---------- ---------- ---------- ---------- ---------- ---------- ----------
```

```
AADN03001784              ---------- ---------- ---------- ---------- ---------- ---------- ---------- ---------- ---------- ---------- ----------
AADN03001783              ---------- ---------- ---------- ---------- ---------- ---------- ---------- ---------- ---------- ---------- ----------
DQ018754                  TCTGCCCGCC GGC------- ---------- ---------- ---------- ---------- ---------- ---------- ---------- ---------- ----------
AADN03001782              ---------- ---------- ---------- ---------- ---------- ---------- ---------- ---------- ---------- ---------- ----------
JN639848                  ---------- ---------- ---------- ---------- ---------- ---------- ---------- ---------- ---------- ---------- ----------
AADN03001670              ---------- ---------- ---------- ---------- ---------- ---------- ---------- ---------- ---------- ---------- ----------
HQ873432                  ---------- ---------- ---------- ---------- ---------- ---------- ---------- ---------- ---------- ---------- ----------
AADN03001774              ---------- ---------- ---------- ---------- ---------- ---------- ---------- ---------- ---------- ---------- ----------
AADN03001775              ---------- ---------- ---------- ---------- ---------- ---------- ---------- ---------- ---------- ---------- ----------
EF552813                  ---------- ---------- ---------- ---------- ---------- ---------- ---------- ---------- ---------- ---------- ----------
AADN03022685              ---------- ---------- ---------- ---------- ---------- ---------- ---------- ---------- ---------- ---------- ----------
AADN03019346              ---------- ---------- ---------- ---------- ---------- ---------- ---------- ---------- ---------- ---------- ----------
AADN03001776              ---------- ---------- ---------- ---------- ---------- ---------- ---------- ---------- ---------- ---------- ----------
DQ018756                  ---------- ---------- ---------- ---------- ---------- ---------- ---------- ---------- ---------- ---------- ----------
AF173612                  ---------- ---------- ---------- ---------- ---------- ---------- ---------- ---------- ---------- ---------- ----------
Gallus_gallus_KT445934.2  TCTGCCCGCC GGCGGGACGC CGGGATGGAA GAGAGGACTC CGGGCGGGGC GCGGCGGCGC GCCCCGCCGG CCCTCTCCCT CCCGAGCCCG CCGGCGGCGT CGGCCGTCGC

                          5555555555 5555555555 5555555555 5555555555 5555555555 5555555555 5555555555 5555555555 5555555555 5555555555 5555555555
                          0000000000 0000000000 0000000000 0000000001 1111111111 1111111111 1111111111 1111111111 1111111111 1111111111 1111111111
                          6666666667 7777777778 8888888889 9999999990 0000000001 1111111112 2222222223 3333333334 4444444445 5555555556 6666666667
                          1234567890 1234567890 1234567890 1234567890 1234567890 1234567890 1234567890 1234567890 1234567890 1234567890 1234567890
AADN03001677              ---------- ---------- ---------- ---------- ---------- ---------- ---------- ---------- ---------- ---------- ----------
AADN03001778              ---------- ---------- ---------- ---------- ---------- ---------- ---------- ---------- ---------- ---------- ----------
AADN03001785              ---------- ---------- ---------- ---------- ---------- ---------- ---------- ---------- ---------- ---------- ----------
AADN03001786              ---------- ---------- ---------- ---------- ---------- ---------- ---------- ---------- ---------- ---------- ----------
AADN03001788              ---------- ---------- ---------- ---------- ---------- ---------- ---------- ---------- ---------- ---------- ----------
AADN03014081              ---------- ---------- ---------- ---------- ---------- ---------- ---------- ---------- ---------- ---------- ----------
AADN03015064              ---------- ---------- ---------- ---------- ---------- ---------- ---------- ---------- ---------- ---------- ----------
AADN03026634              ---------- ---------- ---------- ---------- ---------- ---------- ---------- ---------- ---------- ---------- ----------
NW_003775878              ---------- ---------- ---------- ---------- ---------- ---------- ---------- ---------- ---------- ---------- ----------
DQ018752                  ---------- ---------- ---------- ---------- ---------- ---------- ---------- ---------- ---------- ---------- ----------
DQ018757                  ---------- ---------- ---------- ---------- ---------- ---------- ---------- ---------- ---------- ---------- ----------
DQ112354                  ---------- ---------- ---------- ---------- ---------- ---------- ---------- ---------- ---------- ---------- ----------
FM165414                  ---------- ---------- ---------- ---------- ---------- ---------- ---------- ---------- ---------- ---------- ----------
FM165415                  ---------- ---------- ---------- ---------- ---------- ---------- ---------- ---------- ---------- ---------- ----------
AADN03000430              ---------- ---------- ---------- ---------- ---------- ---------- ---------- ---------- ---------- ---------- ----------
AADN03001784              ---------- ---------- ---------- ---------- ---------- ---------- ---------- ---------- ---------- ---------- ----------
AADN03001783              ---------- ---------- ---------- ---------- ---------- ---------- ---------- ---------- ---------- ---------- ----------
DQ018754                  ---------- ---------- ---------- ---------- ---------- ---------- ---------- ---------- ---------- ---------- ----------
AADN03001782              ---------- ---------- ---------- ---------- ---------- ---------- ---------- ---------- ---------- ---------- ----------
JN639848                  ---------- ---------- ---------- ---------- ---------- ---------- ---------- ---------- ---------- ---------- ----------
AADN03001670              ---------- ---------- ---------- ---------- ---------- ---------- ---------- ---------- ---------- ---------- ----------
HQ873432                  ---------- ---------- ---------- ---------- ---------- ---------- ---------- ---------- ---------- ---------- ----------
AADN03001774              ---------- ---------- ---------- ---------- ---------- ---------- ---------- ---------- ---------- ---------- ----------
AADN03001775              ---------- ---------- ---------- ---------- ---------- ---------- ---------- ---------- ---------- ---------- ----------
EF552813                  ---------- ---------- ---------- ---------- ---------- ---------- ---------- ---------- ---------- ---------- ----------
AADN03022685              ---------- ---------- ---------- ---------- ---------- ---------- ---------- ---------- ---------- ---------- ----------
AADN03019346              ---------- ---------- ---------- ---------- ---------- ---------- ---------- ---------- ---------- ---------- ----------
AADN03001776              ---------- ---------- ---------- ---------- ---------- ---------- ---------- ---------- ---------- ---------- ----------
DQ018756                  ---------- ---------- ---------- ---------- ---------- ---------- ---------- ---------- ---------- ---------- ----------
AF173612                  ---------- ---------- ---------- ---------- ---------- ---------- ---------- ---------- ---------- ---------- ----------
Gallus_gallus_KT445934.2  CGCGCCCTCG GTCCTCCGCG GGGCGGGCCC GGGCCGGAGA GGGGGTCATC CCGTCCCCCC TCTCCGCGGC CTCGGTCTCG GGCGGAGAGC TCGGCGCGCG CGCGGGCGCG
```

```
                          5555555555 5555555555 5555555555 5555555555 5555555555 5555555555 5555555555 5555555555 5555555555 5555555555 5555555555
                          1111111111 1111111111 1111111112 2222222222 2222222222 2222222222 2222222222 2222222222 2222222222 2222222222 2222222222
                          7777777778 8888888889 9999999990 0000000001 1111111112 2222222223 3333333334 4444444445 5555555556 6666666667 7777777778
                          1234567890 1234567890 1234567890 1234567890 1234567890 1234567890 1234567890 1234567890 1234567890 1234567890 1234567890
AADN03001677              ---------- ---------- ---------- ---------- ---------- ---------- ---------- ---------- ---------- ---------- ----------
AADN03001778              ---------- ---------- ---------- ---------- ---------- ---------- ---------- ---------- ---------- ---------- ----------
AADN03001785              ---------- ---------- ---------- ---------- ---------- ---------- ---------- ---------- ---------- ---------- ----------
AADN03001786              ---------- ---------- ---------- ---------- ---------- ---------- ---------- ---------- ---------- ---------- ----------
AADN03001788              ---------- ---------- ---------- ---------- ---------- ---------- ---------- ---------- ---------- ---------- ----------
AADN03014081              ---------- ---------- ---------- ---------- ---------- ---------- ---------- ---------- ---------- ---------- ----------
AADN03015064              ---------- ---------- ---------- ---------- ---------- ---------- ---------- ---------- ---------- ---------- ----------
AADN03026634              ---------- ---------- ---------- ---------- ---------- ---------- ---------- ---------- ---------- ---------- ----------
NW_003775878              ---------- ---------- ---------- ---------- ---------- ---------- ---------- ---------- ---------- ---------- ----------
DQ018752                  ---------- ---------- ---------- ---------- ---------- ---------- ---------- ---------- ---------- ---------- ----------
DQ018757                  ---------- ---------- ---------- ---------- ---------- ---------- ---------- ---------- ---------- ---------- ----------
DQ112354                  ---------- ---------- ---------- ---------- ---------- ---------- ---------- ---------- ---------- ---------- ----------
FM165414                  ---------- ---------- ---------- ---------- ---------- ---------- ---------- ---------- ---------- ---------- ----------
FM165415                  ---------- ---------- ---------- ---------- ---------- ---------- ---------- ---------- ---------- ---------- ----------
AADN03000430              ---------- ---------- ---------- ---------- ---------- ---------- ---------- ---------- ---------- ---------- ----------
AADN03001784              ---------- ---------- ---------- ---------- ---------- ---------- ---------- ---------- ---------- ---------- ----------
AADN03001783              ---------- ---------- ---------- ---------- ---------- ---------- ---------- ---------- ---------- ---------- ----------
DQ018754                  ---------- ---------- ---------- ---------- ---------- ---------- ---------- ---------- ---------- ---------- ----------
AADN03001782              ---------- ---------- ---------- ---------- ---------- ---------- ---------- ---------- ---------- ---------- ----------
JN639848                  ---------- ---------- ---------- ---------- ---------- ---------- ---------- ---------- ---------- ---------- ----------
AADN03001670              ---------- ---------- ---------- ---------- ---------- ---------- ---------- ---------- ---------- ---------- ----------
HQ873432                  ---------- ---------- ---------- ---------- ---------- ---------- ---------- ---------- ---------- ---------- ----------
AADN03001774              ---------- ---------- ---------- ---------- ---------- ---------- ---------- ---------- ---------- ---------- ----------
AADN03001775              ---------- ---------- ---------- ---------- ---------- ---------- ---------- ---------- ---------- ---------- ----------
EF552813                  ---------- ---------- ---------- ---------- ---------- ---------- ---------- ---------- ---------- ---------- ----------
AADN03022685              ---------- ---------- ---------- ---------- ---------- ---------- ---------- ---------- ---------- ---------- ----------
AADN03019346              ---------- ---------- ---------- ---------- ---------- ---------- ---------- ---------- ---------- ---------- ----------
AADN03001776              ---------- ---------- ---------- ---------- ---------- ---------- ---------- ---------- ---------- ---------- ----------
DQ018756                  ---------- ---------- ---------- ---------- ---------- ---------- ---------- ---------- ---------- ---------- ----------
AF173612                  ---------- ---------- ---------- ---------- ---------- ---------- ---------- ---------- ---------- ---------- ----------
Gallus_gallus_KT445934.2  CGCTCGCTCC GGCCGGCCTC GCCCGCGTAC GGAGCGGGCC GAGACGCGGG TCTCGGCCCG GCGCCCGCCG CTCCCCGCGG CGGTGCGTTC CGCGGCCTCC CCGCCGCGCG

                          5555555555 5555555555 5555555555 5555555555 5555555555 5555555555 5555555555 5555555555 5555555555 5555555555 5555555555
                          2222222222 2222222223 3333333333 3333333333 3333333333 3333333333 3333333333 3333333333 3333333333 3333333333 3333333333
                          8888888889 9999999990 0000000001 1111111112 2222222223 3333333334 4444444445 5555555556 6666666667 7777777778 8888888889
                          1234567890 1234567890 1234567890 1234567890 1234567890 1234567890 1234567890 1234567890 1234567890 1234567890 1234567890
AADN03001677              ---------- ---------- ---------- ---------- ---------- ---------- ---------- ---------- ---------- ---------- ----------
AADN03001778              ---------- ---------- ---------- ---------- ---------- ---------- ---------- ---------- ---------- ---------- ----------
AADN03001785              ---------- ---------- ---------- ---------- ---------- ---------- ---------- ---------- ---------- ---------- ----------
AADN03001786              ---------- ---------- ---------- ---------- ---------- ---------- ---------- ---------- ---------- ---------- ----------
AADN03001788              ---------- ---------- ---------- ---------- ---------- ---------- ---------- ---------- ---------- ---------- ----------
AADN03014081              ---------- ---------- ---------- ---------- ---------- ---------- ---------- ---------- ---------- ---------- ----------
AADN03015064              ---------- ---------- ---------- ---------- ---------- ---------- ---------- ---------- ---------- ---------- ----------
AADN03026634              ---------- ---------- ---------- ---------- ---------- ---------- ---------- ---------- ---------- ---------- ----------
NW_003775878              ---------- ---------- ---------- ---------- ---------- ---------- ---------- ---------- ---------- ---------- ----------
DQ018752                  ---------- ---------- ---------- ---------- ---------- ---------- ---------- ---------- ---------- ---------- ----------
DQ018757                  ---------- ---------- ---------- ---------- ---------- ---------- ---------- ---------- ---------- ---------- ----------
DQ112354                  ---------- ---------- ---------- ---------- ---------- ---------- ---------- ---------- ---------- ---------- ----------
FM165414                  ---------- ---------- ---------- ---------- ---------- ---------- ---------- ---------- ---------- ---------- ----------
FM165415                  ---------- ---------- ---------- ---------- ---------- ---------- ---------- ---------- ---------- ---------- ----------
AADN03000430              ---------- ---------- ---------- ---------- ---------- ---------- ---------- ---------- ---------- ---------- ----------
```

```
AADN03001784               ---------- ---------- ---------- ---------- ---------- ---------- ---------- ---------- ---------- ---------- ----------
AADN03001783               ---------- ---------- ---------- ---------- ---------- ---------- ---------- ---------- ---------- ---------- ----------
DQ018754                   ---------- ---------- ---------- ---------- ---------- ---------- ---------- ---------- ---------- ---------- ----------
AADN03001782               ---------- ---------- ---------- ---------- ---------- ---------- ---------- ---------- ---------- ---------- ----------
JN639848                   ---------- ---------- ---------- ---------- ---------- ---------- ---------- ---------- ---------- ---------- ----------
AADN03001670               ---------- ---------- ---------- ---------- ---------- ---------- ---------- ---------- ---------- ---------- ----------
HQ873432                   ---------- ---------- ---------- ---------- ---------- ---------- ---------- ---------- ---------- ---------- ----------
AADN03001774               ---------- ---------- ---------- ---------- ---------- ---------- ---------- ---------- ---------- ---------- ----------
AADN03001775               ---------- ---------- ---------- ---------- ---------- ---------- ---------- ---------- ---------- ---------- ----------
EF552813                   ---------- ---------- ---------- ---------- ---------- ---------- ---------- ---------- ---------- ---------- ----------
AADN03022685               ---------- ---------- ---------- ---------- ---------- ---------- ---------- ---------- ---------- ---------- ----------
AADN03019346               ---------- ---------- ---------- ---------- ---------- ---------- ---------- ---------- ---------- ---------- ----------
AADN03001776               ---------- ---------- ---------- ---------- ---------- ---------- ---------- ---------- ---------- ---------- ----------
DQ018756                   ---------- ---------- ---------- ---------- ---------- ---------- ---------- ---------- ---------- ---------- ----------
AF173612                   ---------- ---------- ---------- ---------- ---------- ---------- ---------- ---------- ---------- ---------- ----------
Gallus_gallus_KT445934.2   CGGCCGGCGG GACGGCGAGC CGGCCGTCCC GCCCGCGCCA GCCGCGGCGC CGGCGGTTCC GCTCCGCCGG TCCGCCCCGC GTGCGTCCGC ACGCCCGGCC TCCTGCCCTC

                           5555555555 5555555555 5555555555 5555555555 5555555555 5555555555 5555555555 5555555555 5555555555 5555555555 5555555555
                           3333333334 4444444444 4444444444 4444444444 4444444444 4444444444 4444444444 4444444444 4444444444 4444444444 4444444445
                           9999999990 0000000001 1111111112 2222222223 3333333334 4444444445 5555555556 6666666667 7777777778 8888888889 9999999990
                           1234567890 1234567890 1234567890 1234567890 1234567890 1234567890 1234567890 1234567890 1234567890 1234567890 1234567890
AADN03001677               ---------- ---------- ---------- ---------- ---------- ---------- ---------- ---------- ---------- ---------- ----------
AADN03001778               ---------- ---------- ---------- ---------- ---------- ---------- ---------- ---------- ---------- ---------- ----------
AADN03001785               ---------- ---------- ---------- ---------- ---------- ---------- ---------- ---------- ---------- ---------- ----------
AADN03001786               ---------- ---------- ---------- ---------- ---------- ---------- ---------- ---------- ---------- ---------- ----------
AADN03001788               ---------- ---------- ---------- ---------- ---------- ---------- ---------- ---------- ---------- ---------- ----------
AADN03014081               ---------- ---------- ---------- ---------- ---------- ---------- ---------- ---------- ---------- ---------- ----------
AADN03015064               ---------- ---------- ---------- ---------- ---------- ---------- ---------- ---------- ---------- ---------- ----------
AADN03026634               ---------- ---------- ---------- ---------- ---------- ---------- ---------- ---------- ---------- ---------- ----------
NW_003775878               ---------- ---------- ---------- ---------- ---------- ---------- ---------- ---------- ---------- ---------- ----------
DQ018752                   ---------- ---------- ---------- ---------- ---------- ---------- ---------- ---------- ---------- ---------- ----------
DQ018757                   ---------- ---------- ---------- ---------- ---------- ---------- ---------- ---------- ---------- ---------- ----------
DQ112354                   ---------- ---------- ---------- ---------- ---------- ---------- ---------- ---------- ---------- ---------- ----------
FM165414                   ---------- ---------- ---------- ---------- ---------- ---------- ---------- ---------- ---------- ---------- ----------
FM165415                   ---------- ---------- ---------- ---------- ---------- ---------- ---------- ---------- ---------- ---------- ----------
AADN03000430               ---------- ---------- ---------- ---------- ---------- ---------- ---------- ---------- ---------- ---------- ----------
AADN03001784               ---------- ---------- ---------- ---------- ---------- ---------- ---------- ---------- ---------- ---------- ----------
AADN03001783               ---------- ---------- ---------- ---------- ---------- ---------- ---------- ---------- ---------- ---------- ----------
DQ018754                   ---CGGGCCT CGCCGGCCGT TTCCCCTTCC GTCGCAAGCC GCGTCCTCTC CTTCGTCCCC GCCGCCGTCG CCTTCCCACC GCGCTT-CGC C-TCGGCCTC GCCGGCCGCG
AADN03001782               ---------- ---------- ---------- ---------- ---------- ---------- ---------- ---------- ---------- CCTCGGCCTC GCCGGCCGCG
JN639848                   ---------- ---------- ---------- ---------- ---------- ---------- ---------- ---------- ---------- ---------- ----------
AADN03001670               ---------- ---------- ---------- ---------- ---------- ---------- ---------- ---------- ---------- ---------- ----------
HQ873432                   ---------- ---------- ---------- ---------- ---------- ---------- ---------- ---------- ---------- ---------- ----------
AADN03001774               ---------- ---------- ---------- ---------- ---------- ---------- ---------- ---------- ---------- ---------- ----------
AADN03001775               ---------- ---------- ---------- ---------- ---------- ---------- ---------- ---------- ---------- ---------- ----------
EF552813                   ---------- ---------- ---------- ---------- ---------- ---------- ---------- ---------- ---------- ---------- ----------
AADN03022685               ---------- ---------- ---------- ---------- ---------- ---------- ---------- ---------- ---------- ---------- ----------
AADN03019346               ---------- ---------- ---------- ---------- ---------- ---------- ---------- ---------- ---------- ---------- ----------
AADN03001776               ---------- ---------- ---------- ---------- ---------- ---------- ---------- ---------- ---------- ---------- ----------
DQ018756                   ---------- ---------- ---------- ---------- ---------- ---------- ---------- ---------- ---------- ---------- ----------
AF173612                   ---------- ---------- ---------- ---------- ---------- ---------- ---------- ---------- ---------- ---------- ----------
Gallus_gallus_KT445934.2   CCTCGGGGCC TCGCCGCCGT TTCCCCCTTC CGTCGCAAGC CGCGTCCTCT CCTTCGTCCC CGCCGCCGTC GCCTCCCACC GCGCTTTCGC CCTCGGCCTC GCCGGCCGCG
```

```
                           5555555555 5555555555 5555555555 5555555555 5555555555 5555555555 5555555555 5555555555 5555555555 5555555555 5555555555
                           5555555555 5555555555 5555555555 5555555555 5555555555 5555555555 5555555555 5555555555 5555555555 5555555556 6666666666
                           0000000001 1111111112 2222222223 3333333334 4444444445 5555555556 6666666667 7777777778 8888888889 9999999990 0000000001
                           1234567890 1234567890 1234567890 1234567890 1234567890 1234567890 1234567890 1234567890 1234567890 1234567890 1234567890
AADN03001677               ---------- ---------- ---------- ---------- ---------- ---------- ---------- ---------- ---------- ---------- ----------
AADN03001778               ---------- ---------- ---------- ---------- ---------- ---------- ---------- ---------- ---------- ---------- ----------
AADN03001785               ---------- ---------- ---------- ---------- ---------- ---------- ---------- ---------- ---------- ---------- ----------
AADN03001786               ---------- ---------- ---------- ---------- ---------- ---------- ---------- ---------- ---------- ---------- ----------
AADN03001788               ---------- ---------- ---------- ---------- ---------- ---------- ---------- ---------- ---------- ---------- ----------
AADN03014081               ---------- ---------- ---------- ---------- ---------- ---------- ---------- ---------- ---------- ---------- ----------
AADN03015064               ---------- ---------- ---------- ---------- ---------- ---------- ---------- ---------- ---------- ---------- ----------
AADN03026634               ---------- ---------- ---------- ---------- ---------- ---------- ---------- ---------- ---------- ---------- ----------
NW_003775878               ---------- ---------- ---------- ---------- ---------- ---------- ---------- ---------- ---------- ---------- ----------
DQ018752                   ---------- ---------- ---------- ---------- ---------- ---------- ---------- ---------- ---------- ---------- ----------
DQ018757                   ---------- ---------- ---------- ---------- ---------- ---------- ---------- ---------- ---------- ---------- ----------
DQ112354                   ---------- ---------- ---------- ---------- ---------- ---------- ---------- ---------- ---------- ---------- ----------
FM165414                   ---------- ---------- ---------- ---------- ---------- ---------- ---------- ---------- ---------- ---------- ----------
FM165415                   ---------- ---------- ---------- ---------- ---------- ---------- ---------- ---------- ---------- ---------- ----------
AADN03000430               ---------- ---------- ---------- ---------- ---------- ---------- ---------- ---------- ---------- ---------- ----------
AADN03001784               ---------- ---------- ---------- ---------- ---------- ---------- ---------- ---------- ---------- ---------- ----------
AADN03001783               ---------- ---------- ---------- ---------- ---------- ---------- ---------- ---------- ---------- ---------- ----------
DQ018754                   CCGGTCGTGC GAGCGGGAGG TCCGGCGTGG GGCGTCCCGC AGCCGGTCTC CGCGCGGAGG CGCGGGGAGC GGGCGCCGCT CCCGAATCCG TCCCCGTCCC GCCCGCCGCC
AADN03001782               CCGGTCGTGC GAGCGGGAGG TCCGGCGTGG GGCGTCCCGC AGCCGGTCTC CGCGCGGAGG CGCGGGGAGC GGGCGCCGCT CCCGAATCCG TCCCCGTCCC GCCCGCCGCC
JN639848                   ---------- ---------- ---------- ---------- ---------- ---------- ---------- ---------- ---------- ---------- ----------
AADN03001670               ---------- ---------- --CGGCGTGG GGCGTCCCGC AGCCGGTCTC CGCGCGGAGG CGCGGGGAGC GGGCGCCGCT CCCGAATCCT TCCCCGTCCC GCCCGCCGCC
HQ873432                   ---------- ---------- ---------- ---------- ---------- ---------- ---------- ---------- ---------- ---------- ----------
AADN03001774               ---------- ---------- ---------- ---------- ---------- ---------- ---------- ---------- ---------- ---------- ----------
AADN03001775               ---------- ---------- ---------- ---------- ---------- ---------- ---------- ---------- ---------- ---------- ----------
EF552813                   ---------- ---------- ---------- ---------- ---------- ---------- ---------- ---------- ---------- ---------- ----------
AADN03022685               ---------- ---------- ---------- ---------- ---------- ---------- ---------- ---------- ---------- ---------- ----------
AADN03019346               ---------- ---------- ---------- ---------- ---------- ---------- ---------- ---------- ---------- ---------- ----------
AADN03001776               ---------- ---------- ---------- ---------- ---------- ---------- ---------- ---------- ---------- ---------- ----------
DQ018756                   ---------- ---------- ---------- ---------- ---------- ---------- ---------- ---------- ---------- ---------- ----------
AF173612                   ---------- ---------- ---------- ---------- ---------- ---------- ---------- ---------- ---------- ---------- ----------
Gallus_gallus_KT445934.2   CCGGTCGTGC GAGCGGGAGG TCCGGCGTGG GGCGTCCCGC AGCCGGTCTC CGCGCGGAGG CGCGGGGAGC GGGCGCCGCT CCCGAATCCG TCCCCGTCCC GCCCGCCGCC

                           5555555555 5555555555 5555555555 5555555555 5555555555 5555555555 5555555555 5555555555 5555555555 5555555555 5555555555
                           6666666666 6666666666 6666666666 6666666666 6666666666 6666666666 6666666666 6666666666 6666666667 7777777777 7777777777
                           1111111112 2222222223 3333333334 4444444445 5555555556 6666666667 7777777778 8888888889 9999999990 0000000001 1111111112
                           1234567890 1234567890 1234567890 1234567890 1234567890 1234567890 1234567890 1234567890 1234567890 1234567890 1234567890
AADN03001677               ---------- ---------- ---------- ---------- ---------- ---------- ---------- ---------- ---------- ---------- ----------
AADN03001778               ---------- ---------- ---------- ---------- ---------- ---------- ---------- ---------- ---------- ---------- ----------
AADN03001785               ---------- ---------- ---------- ---------- ---------- ---------- ---------- ---------- ---------- ---------- ----------
AADN03001786               ---------- ---------- ---------- ---------- ---------- ---------- ---------- ---------- ---------- ---------- ----------
AADN03001788               ---------- ---------- ---------- ---------- ---------- ---------- ---------- ---------- ---------- ---------- ----------
AADN03014081               ---------- ---------- ---------- ---------- ---------- ---------- ---------- ---------- ---------- ---------- ----------
AADN03015064               ---------- ---------- ---------- ---------- ---------- ---------- ---------- ---------- ---------- ---------- ----------
AADN03026634               ---------- ---------- ---------- ---------- ---------- ---------- ---------- ---------- ---------- ---------- ----------
NW_003775878               ---------- ---------- ---------- ---------- ---------- ---------- ---------- ---------- ---------- ---------- ----------
DQ018752                   ---------- ---------- ---------- ---------- ---------- ---------- ---------- ---------- ---------- ---------- ----------
DQ018757                   ---------- ---------- ---------- ---------- ---------- ---------- ---------- ---------- ---------- ---------- ----------
DQ112354                   ---------- ---------- ---------- ---------- ---------- ---------- ---------- ---------- ---------- ---------- ----------
FM165414                   ---------- ---------- ---------- ---------- ---------- ---------- ---------- ---------- ---------- ---------- ----------
FM165415                   ---------- ---------- ---------- ---------- ---------- ---------- ---------- ---------- ---------- ---------- ----------
AADN03000430               ---------- ---------- ---------- ---------- ---------- ---------- ---------- ---------- ---------- ---------- ----------
```

```
AADN03001784           ---------- ---------- ---------- ---------- ---------- ---------- ---------- ---------- ---------- ---------- ----------
AADN03001783           ---------- ---------- ---------- ---------- ---------- ---------- ---------- ---------- ---------- ---------- ----------
DQ018754               GTGCGCGCGT CCGCCGCGGG CGCGCCGCCA GGGCGAGCGA GAGGAGGAGG CGTCGGAGGA CGAGGGGCGG GGGAGGAAGG TGAGAGGCGG CGGGGGCGTT TCGGTGCGCG
AADN03001782           GTGCGCGCGT CCGCCGCGGG CGCGCCGCCA GGGCGAGCGA GAGGAGGAGG CGTCGGAGGA CGAGGGGCGG GGGAGGAAGG TGAGAGGCGG CGGGGGCGTT TCGGTGCGCG
JN639848               ---------- ---------- ---------- ---------- ---------- ---------- ---------- ---------- ---------- ---------- ----------
AADN03001670           GTGCGCGCGT CCGCCGCGGG CGCGCCGCCA GGGCGAGCGA GAGGAGGAGG CGTCGGAGGA CGAGGGGCGG GGGAGGAAGG TGAGAGGCGG CGGGGGCGTT TCGGTGCGCG
HQ873432               ---------- ---------- ---------- ---------- ---------- ---------- ---------- ---------- ---------- ---------- ----------
AADN03001774           ---------- ---------- ---------- ---------- ---------- ---------- ---------- ---------- ---------- ---------- ----------
AADN03001775           ---------- ---------- ---------- ---------- ---------- ---------- ---------- ---------- ---------- ---------- ----------
EF552813               ---------- ---------- ---------- ---------- ---------- ---------- ---------- ---------- ---------- ---------- ----------
AADN03022685           ---------- ---------- ---------- ---------- ---------- ---------- ---------- ---------- ---------- ---------- ----------
AADN03019346           ---------- ---------- ---------- ---------- ---------- ---------- ---------- ---------- ---------- ---------- ----------
AADN03001776           ---------- ---------- ---------- ---------- ---------- ---------- ---------- ---------- ---------- ---------- ----------
DQ018756               ---------- ---------- ---------- ---------- ---------- ---------- ---------- ---------- ---------- ---------- ----------
AF173612               ---------- ---------- ---------- ---------- ---------- ---------- ---------- ---------- ---------- ---------- ----------
Gallus_gallus_KT445934.2    GTGCGCGCGT CCGCCGCGGG CGCGCCGCCA GGGCGAGCGA GAGGAGGAGG CGTCGGAGGA CGAGGGGCGG GGGAGGAAGG TGAGAGGCGG CGGGGGCGTT TCGGTGCGCG

                       5555555555 5555555555 5555555555 5555555555 5555555555 5555555555 5555555555 5555555555 5555555555 5555555555 5555555555
                       7777777777 7777777777 7777777777 7777777777 7777777777 7777777777 7777777777 7777777778 8888888888 8888888888 8888888888
                       2222222223 3333333334 4444444445 5555555556 6666666667 7777777778 8888888889 9999999990 0000000001 1111111112 2222222223
                       1234567890 1234567890 1234567890 1234567890 1234567890 1234567890 1234567890 1234567890 1234567890 1234567890 1234567890
AADN03001677           ---------- ---------- ---------- ---------- ---------- ---------- ---------- ---------- ---------- ---------- ----------
AADN03001778           ---------- ---------- ---------- ---------- ---------- ---------- ---------- ---------- ---------- ---------- ----------
AADN03001785           ---------- ---------- ---------- ---------- ---------- ---------- ---------- ---------- ---------- ---------- ----------
AADN03001786           ---------- ---------- ---------- ---------- ---------- ---------- ---------- ---------- ---------- ---------- ----------
AADN03001788           ---------- ---------- ---------- ---------- ---------- ---------- ---------- ---------- ---------- ---------- ----------
AADN03014081           ---------- ---------- ---------- ---------- ---------- ---------- ---------- ---------- ---------- ---------- ----------
AADN03015064           ---------- ---------- ---------- ---------- ---------- ---------- ---------- ---------- ---------- ---------- ----------
AADN03026634           ---------- ---------- ---------- ---------- ---------- ---------- ---------- ---------- ---------- ---------- ----------
NW_003775878           ---------- ---------- ---------- ---------- ---------- ---------- ---------- ---------- ---------- ---------- ----------
DQ018752               ---------- ---------- ---------- ---------- ---------- ---------- ---------- ---------- ---------- ---------- ----------
DQ018757               ---------- ---------- ---------- ---------- ---------- ---------- ---------- ---------- ---------- ---------- ----------
DQ112354               ---------- ---------- ---------- ---------- ---------- ---------- ---------- ---------- ---------- ---------- ----------
FM165414               ---------- ---------- ---------- ---------- ---------- ---------- ---------- ---------- ---------- ---------- ----------
FM165415               ---------- ---------- ---------- ---------- ---------- ---------- ---------- ---------- ---------- ---------- ----------
AADN03000430           ---------- ---------- ---------- ---------- ---------- ---------- ---------- ---------- ---------- ---------- ----------
AADN03001784           ---------- ---------- ---------- ---------- ---------- ---------- ---------- ---------- ---------- ---------- ----------
AADN03001783           ---------- ---------- ---------- ---------- ---------- ---------- ---------- ---------- ---------- ---------- ----------
DQ018754               CGTCTCCCGC ACGGCGAGGA AGGGGCCGAG GTCGGCGCGG GCGCCGTCGG GCGGTCCGGC GCGAGCGCGG TCCGGCGGCG CCGCCGGCGC GGGCGGGGGC CCGGTCTGCT
AADN03001782           CGTCTCCCGC ACGGCGAGGA AGGGGCCGAG GTCGGTGCGG GCGCCGTCGG GCGGTCCGGC GCGGGCGCGG GCCGGCGGCG CCGCCGGCGC GGGCGGGGGC CTGGTCTGCT
JN639848               ---------- ---------- ---------- ---------- ---------- ---------- ---------- ---------- ---------- ---------- ----------
AADN03001670           CGTCTCCCGC ACGGCGAGGA AGGGGCCGAG GTCGGTGCGG GCGCCGTCGG GCGGTCCGGC GCGGGCGCGG TCCGGCGGCG CCGCCGGCGC GGGCGGGGGC CTGGTCTGCT
HQ873432               ---------- ---------- ---------- ---------- ---------- ---------- ---------- ---------- ---------- ---------- ----------
AADN03001774           ---------- ---------- ---------- ---------- ---------- ---------- ---------- ---------- ---------- ---------- ----------
AADN03001775           ---------- ---------- ---------- ---------- ---------- ---------- ---------- ---------- ---------- ---------- ----------
EF552813               ---------- ---------- ---------- ---------- ---------- ---------- ---------- ---------- ---------- ---------- ----------
AADN03022685           ---------- ---------- ---------- ---------- ---------- ---------- ---------- ---------- ---------- ---------- ----------
AADN03019346           ---------- ---------- ---------- ---------- ---------- ---------- ---------- ---------- ---------- ---------- ----------
AADN03001776           ---------- ---------- ---------- ---------- ---------- ---------- ---------- ---------- ---------- ---------- ----------
DQ018756               ---------- ---------- ---------- ---------- ---------- ---------- ---------- ---------- ---------- ---------- ----------
AF173612               ---------- ---------- ---------- ---------- ---------- ---------- ---------- ---------- ---------- ---------- ----------
Gallus_gallus_KT445934.2    CGTCTCCCGC ACGGCGAGGA AGGGGCCGAG GTCGGCGCGG GCGCCGTCGG GCGGTCCGGC GCGGGCGCGG GCCGGCGGCG CCGCCGGCGC GGGCGGGGGC CTGGTCTGCT
```

```
                           5555555555 5555555555 5555555555 5555555555 5555555555 5555555555 5555555555 5555555555 5555555555 5555555555 5555555555
                           8888888888 8888888888 8888888888 8888888888 8888888888 8888888888 8888888889 9999999999 9999999999 9999999999 9999999999
                           3333333334 4444444445 5555555556 6666666667 7777777778 8888888889 9999999990 0000000001 1111111112 2222222223 3333333334
                           1234567890 1234567890 1234567890 1234567890 1234567890 1234567890 1234567890 1234567890 1234567890 1234567890 1234567890
AADN03001677               ---------- ---------- ---------- ---------- ---------- ---------- ---------- ---------- ---------- ---------- ----------
AADN03001778               ---------- ---------- ---------- ---------- ---------- ---------- ---------- ---------- ---------- ---------- ----------
AADN03001785               ---------- ---------- ---------- ---------- ---------- ---------- ---------- ---------- ---------- ---------- ----------
AADN03001786               ---------- ---------- ---------- ---------- ---------- ---------- ---------- ---------- ---------- ---------- ----------
AADN03001788               ---------- ---------- ---------- ---------- ---------- ---------- ---------- ---------- ---------- ---------- ----------
AADN03014081               ---------- ---------- ---------- ---------- ---------- ---------- ---------- ---------- ---------- ---------- ----------
AADN03015064               ---------- ---------- ---------- ---------- ---------- ---------- ---------- ---------- ---------- ---------- ----------
AADN03026634               ---------- ---------- ---------- ---------- ---------- ---------- ---------- ---------- ---------- ---------- ----------
NW_003775878               ---------- ---------- ---------- ---------- ---------- ---------- ---------- ---------- ---------- ---------- ----------
DQ018752                   ---------- ---------- ---------- ---------- ---------- ---------- ---------- ---------- ---------- ---------- ----------
DQ018757                   ---------- ---------- ---------- ---------- ---------- ---------- ---------- ---------- ---------- ---------- ----------
DQ112354                   ---------- ---------- ---------- ---------- ---------- ---------- ---------- ---------- ---------- ---------- ----------
FM165414                   ---------- ---------- ---------- ---------- ---------- ---------- ---------- ---------- ---------- ---------- ----------
FM165415                   ---------- ---------- ---------- ---------- ---------- ---------- ---------- ---------- ---------- ---------- ----------
AADN03000430               ---------- ---------- ---------- ---------- ---------- ---------- ---------- ---------- ---------- ---------- ----------
AADN03001784               ---------- ---------- ---------- ---------- ---------- ---------- ---------- ---------- ---------- ---------- ----------
AADN03001783               ---------- ---------- ---------- ---------- ---------- ---------- ---------- ---------- ---------- ---------- ----------
DQ018754                   CCCGTCCCCG TCGGTCGCGG CGGCGGCGGC GGCGGCGGTC CGTCGCGGCA GCGGGGCTTC GGCCGGGGCG GCGCGCGCCG TCCCGCGGGC GTCCGTGGCT CCTCCGCCCG
AADN03001782               CCCTTCCCCG TCGGTCGCGG CGGC------ ---------- ---------- ---------- ---------- ---------- ---------- ---------- ----------
JN639848                   ---------- ---------- ---------- ---------- ---------- ---------- ---------- ---------- ---------- ---------- ----------
AADN03001670               CCCGTCCCCG TCGGTCGCGG CGGCGGCGGC GGCGGCGGTC CGTCGCGGCA GCGGGGCTTC GGCCGGGGCG GCGCGCGCCG TCCCGCGGGC GTCCGCGGCT CCTCCGCCCG
HQ873432                   ---------- ---------- ---------- ---------- ---------- ---------- ---------- ---------- ---------- ---------- ----------
AADN03001774               ---------- ---------- ---------- ---------- ---------- ---------- ---------- ---------- ---------- ---------- ----------
AADN03001775               ---------- ---------- ---------- ---------- ---------- ---------- ---------- ---------- ---------- ---------- ----------
EF552813                   ---------- ---------- ---------- ---------- ---------- ---------- ---------- ---------- ---------- ---------- ----------
AADN03022685               ---------- ---------- ---------- ---------- ---------- ---------- ---------- ---------- ---------- ---------- ----------
AADN03019346               ---------- ---------- ---------- ---------- ---------- ---------- ---------- ---------- ---------- ---------- ----------
AADN03001776               ---------- ---------- ---------- ---------- ---------- ---------- ---------- ---------- ---------- ---------- ----------
DQ018756                   ---------- ---------- ---------- ---------- ---------- ---------- ---------- ---------- ---------- ---------- ----------
AF173612                   ---------- ---------- ---------- ---------- ---------- ---------- ---------- ---------- ---------- ---------- ----------
Gallus_gallus_KT445934.2   CCCGTCCCCG TCGGTCGCGG CGGCGGCGGC GGCGGCGGTC CGTCGCGGCA GCGGGGCTTC GGCCGGGGCG GCGCGCGCCG TCCCGCGGGC GTCCGCGGCT CCTCCGCCCG

                           5555555555 5555555555 5555555555 5555555555 5555555555 5555555556 6666666666 6666666666 6666666666 6666666666 6666666666
                           9999999999 9999999999 9999999999 9999999999 9999999999 9999999990 0000000000 0000000000 0000000000 0000000000 0000000000
                           4444444445 5555555556 6666666667 7777777778 8888888889 9999999990 0000000001 1111111112 2222222223 3333333334 4444444445
                           1234567890 1234567890 1234567890 1234567890 1234567890 1234567890 1234567890 1234567890 1234567890 1234567890 1234567890
AADN03001677               ---------- ---------- ---------- ---------- ---------- ---------- ---------- ---------- ---------- ---------- ----------
AADN03001778               ---------- ---------- ---------- ---------- ---------- ---------- ---------- ---------- ---------- ---------- ----------
AADN03001785               ---------- ---------- ---------- ---------- ---------- ---------- ---------- ---------- ---------- ---------- ----------
AADN03001786               ---------- ---------- ---------- ---------- ---------- ---------- ---------- ---------- ---------- ---------- ----------
AADN03001788               ---------- ---------- ---------- ---------- ---------- ---------- ---------- ---------- ---------- ---------- ----------
AADN03014081               ---------- ---------- ---------- ---------- ---------- ---------- ---------- ---------- ---------- ---------- ----------
AADN03015064               ---------- ---------- ---------- ---------- ---------- ---------- ---------- ---------- ---------- ---------- ----------
AADN03026634               ---------- ---------- ---------- ---------- ---------- ---------- ---------- ---------- ---------- ---------- ----------
NW_003775878               ---------- ---------- ---------- ---------- ---------- ---------- ---------- ---------- ---------- ---------- ----------
DQ018752                   ---------- ---------- ---------- ---------- ---------- ---------- ---------- ---------- ---------- ---------- ----------
DQ018757                   ---------- ---------- ---------- ---------- ---------- ---------- ---------- ---------- ---------- ---------- ----------
DQ112354                   ---------- ---------- ---------- ---------- ---------- ---------- ---------- ---------- ---------- ---------- ----------
FM165414                   ---------- ---------- ---------- ---------- ---------- ---------- ---------- ---------- ---------- ---------- ----------
FM165415                   ---------- ---------- ---------- ---------- ---------- ---------- ---------- ---------- ---------- ---------- ----------
AADN03000430               ---------- ---------- ---------- ---------- ---------- ---------- ---------- ---------- ---------- ---------- ----------
```

```
AADN03001784               ---------- ---------- ---------- ---------- ---------- ---------- ---------- ---------- ---------- ---------- ----------
AADN03001783               ---------- ---------- ---------- ---------- ---------- ---------- ---------- ---------- ---------- ---------- ----------
DQ018754                   GGCCGGGCCG AGCCGGGCGC CTGGTCCGTC CCCGAAGCGA GACAGGGTCG TTTCCCCAGG TCGGGAGCGA GGGCTCCCCG CCCTTCTCGT TCGGGTCGCG CTTCATTGCC
AADN03001782               ---------- ---------- ---------- ---------- ---------- ---------- ---------- ---------- ---------- ---------- ----------
JN639848                   ---------- ---------- ---------- ---------- ---------- ---------- ---------- ---------- ---------- ---------- ----------
AADN03001670               GGCCGGGCCG AGCCGGGCGC CTGGTCCGTC CCCGAAGCGA GACAGGGTCG TTTCCCCAGG TCGGGAGCGA GGGCTCCCCG CCCTTCTCGT TCGGGTCGCT TTTCATTGCC
HQ873432                   ---------- ---------- ---------- ---------- ---------- ---------- ---------- ---------- ---------- ---------- ----------
AADN03001774               ---------- ---------- ---------- ---------- ---------- ---------- ---------- ---------- ---------- ---------- ----------
AADN03001775               ---------- ---------- ---------- ---------- ---------- ---------- ---------- ---------- ---------- ---------- ----------
EF552813                   ---------- ---------- ---------- ---------- ---------- ---------- ---------- ---------- ---------- ---------- ----------
AADN03022685               ---------- ---------- ---------- ---------- ---------- ---------- ---------- ---------- ---------- ---------- ----------
AADN03019346               ---------- ---------- ---------- ---------- ---------- ---------- ---------- ---------- ---------- ---------- ----------
AADN03001776               ---------- ---------- ---------- ---------- ---------- ---------- ---------- ---------- ---------- ---------- ----------
DQ018756                   ---------- ---------- ---------- ---------- ---------- ---------- ---------- ---------- ---------- ---------- ----------
AF173612                   ---------- ---------- ---------- ---------- ---------- ---------- ---------- ---------- ---------- ---------- ----------
Gallus_gallus_KT445934.2   GGCCGGGCCG AGCCGGGCGC CTGGTCCGTC CCCGAAGCGA GACAGGGTCG TTTCCCCAGG TCGGGAGCGA GGGCTCCCCG CCCTTCTCGT TCGGGTCGCG CTTCATTGCC

                           6666666666 6666666666 6666666666 6666666666 6666666666 6666666666 6666666666 6666666666 6666666666 6666666666 6666666666
                           0000000000 0000000000 0000000000 0000000000 0000000001 1111111111 1111111111 1111111111 1111111111 1111111111 1111111111
                           5555555556 6666666667 7777777778 8888888889 9999999990 0000000001 1111111112 2222222223 3333333334 4444444445 5555555556
                           1234567890 1234567890 1234567890 1234567890 1234567890 1234567890 1234567890 1234567890 1234567890 1234567890 1234567890
AADN03001677               ---------- ---------- ---------- ---------- ---------- ---------- ---------- ---------- ---------- ---------- ----------
AADN03001778               ---------- ---------- ---------- ---------- ---------- ---------- ---------- ---------- ---------- ---------- ----------
AADN03001785               ---------- ---------- ---------- ---------- ---------- ---------- ---------- ---------- ---------- ---------- ----------
AADN03001786               ---------- ---------- ---------- ---------- ---------- ---------- ---------- ---------- ---------- ---------- ----------
AADN03001788               ---------- ---------- ---------- ---------- ---------- ---------- ---------- ---------- ---------- ---------- ----------
AADN03014081               ---------- ---------- ---------- ---------- ---------- ---------- ---------- ---------- ---------- ---------- ----------
AADN03015064               ---------- ---------- ---------- ---------- ---------- ---------- ---------- ---------- ---------- ---------- ----------
AADN03026634               ---------- ---------- ---------- ---------- ---------- ---------- ---------- ---------- ---------- ---------- ----------
NW_003775878               ---------- ---------- ---------- ---------- ---------- ---------- ---------- ---------- ---------- ---------- ----------
DQ018752                   ---------- ---------- ---------- ---------- ---------- ---------- ---------- ---------- ---------- ---------- ----------
DQ018757                   ---------- ---------- ---------- ---------- ---------- ---------- ---------- ---------- ---------- ---------- ----------
DQ112354                   ---------- ---------- ---------- ---------- ---------- ---------- ---------- ---------- ---------- ---------- ----------
FM165414                   ---------- ---------- ---------- ---------- ---------- ---------- ---------- ---------- ---------- ---------- ----------
FM165415                   ---------- ---------- ---------- ---------- ---------- ---------- ---------- ---------- ---------- ---------- ----------
AADN03000430               ---------- ---------- ---------- ---------- ---------- ---------- ---------- ---------- ---------- ---------- ----------
AADN03001784               ---------- ---------- ---------- ---------- ---------- ---------- ---------- ---------- ---------- ---------- ----------
AADN03001783               ---------- ---------- ---------- ---------- ---------- ---------- ---------- ---------- ---------- ---------- --CCCCGGCG
DQ018754                   GGCCGGCCG- -------TCG CCGGCTTTTT TTT-CCCTCC CGCATCCGAT ATTCGTGTGC TCGTACGGTC AGCGGAGGCG ACGCTCGTCC GCCCCGCGGT CGCCCCGGCG
AADN03001782               ---------- ---------- ---------- ---------- ---------- ---------- ---------- ---------- ---------- ---------- ----------
JN639848                   ---------- ---------- ---------- ---------- ---------- ---------- ---------- ---------- ---------- ---------- ----------
AADN03001670               GGCCGGCCG- -------TCG CCGGCTTTTT TTTTCCCTCC CGCATCCGAT ATTCGTGTGC TCGTACGGTC AGCGGAGGCG ACGCTCGTCC GCCCCGCGGT CGCCCCGGCG
HQ873432                   ---------- ---------- ---------- ---------- ---------- ---------- ---------- ---------- ---------- ---------- ----------
AADN03001774               ---------- ---------- ---------- ---------- ---------- ---------- ---------- ---------- ---------- ---------- ----------
AADN03001775               ---------- ---------- ---------- ---------- ---------- ---------- ---------- ---------- ---------- ---------- ----------
EF552813                   ---------- ---------- ---------- ---------- ---------- ---------- ---------- ---------- ---------- ---------- ----------
AADN03022685               ---------- ---------- ---------- ---------- ---------- ---------- ---------- ---------- ---------- ---------- ----------
AADN03019346               ---------- ---------- ---------- ---------- ---------- ---------- ---------- ---------- ---------- ---------- ----------
AADN03001776               ---------- ---------- ---------- ---------- ---------- ---------- ---------- ---------- ---------- ---------- ----------
DQ018756                   ---------- ---------- ---------- ---------- ---------- ---------- ---------- ---------- ---------- ---------- ----------
AF173612                   ---------- ---------- ---------- ---------- ---------- ---------- ---------- ---------- ---------- ---------- ----------
Gallus_gallus_KT445934.2   GGCCGGCCGG CCGGCCGTCG CCGGCTTTTT TTTTCCCTCC CGCATCCGAT ATTCGTGTGC TCGTACGGTC AGCGGAGGCG ACGCTCGTCC GCCCCGCGGT CGCCCCGGCG
```

```
                            6666666666 6666666666 6666666666 6666666666 6666666666 6666666666 6666666666 6666666666 6666666666 6666666666 6666666666
                            1111111111 1111111111 1111111111 1111111112 2222222222 2222222222 2222222222 2222222222 2222222222 2222222222 2222222222
                            6666666667 7777777778 8888888889 9999999990 0000000001 1111111112 2222222223 3333333334 4444444445 5555555556 6666666667
                            1234567890 1234567890 1234567890 1234567890 1234567890 1234567890 1234567890 1234567890 1234567890 1234567890 1234567890
AADN03001677                ---------- ---------- ---------- ---------- ---------- ---------- ---------- ---------- ---------- ---------- ----------
AADN03001778                ---------- ---------- ---------- ---------- ---------- ---------- ---------- ---------- ---------- ---------- ----------
AADN03001785                ---------- ---------- ---------- ---------- ---------- ---------- ---------- ---------- ---------- ---------- ----------
AADN03001786                ---------- ---------- ---------- ---------- ---------- ---------- ---------- ---------- ---------- ---------- ----------
AADN03001788                ---------- ---------- ---------- ---------- ---------- ---------- ---------- ---------- ---------- ---------- ----------
AADN03014081                ---------- ---------- ---------- ---------- ---------- ---------- ---------- ---------- ---------- ---------- ----------
AADN03015064                ---------- ---------- ---------- ---------- ---------- ---------- ---------- ---------- ---------- ---------- ----------
AADN03026634                ---------- ---------- ---------- ---------- ---------- ---------- ---------- ---------- ---------- ---------- ----------
NW_003775878                ---------- ---------- ---------- ---------- ---------- ---------- ---------- ---------- ---------- ---------- ----------
DQ018752                    ---------- ---------- ---------- ---------- ---------- ---------- ---------- ---------- ---------- ---------- ----------
DQ018757                    ---------- ---------- ---------- ---------- ---------- ---------- ---------- ---------- ---------- ---------- ----------
DQ112354                    ---------- ---------- ---------- ---------- ---------- ---------- ---------- ---------- ---------- ---------- ----------
FM165414                    ---------- ---------- ---------- ---------- ---------- ---------- ---------- ---------- ---------- ---------- ----------
FM165415                    ---------- ---------- ---------- ---------- ---------- ---------- ---------- ---------- ---------- ---------- ----------
AADN03000430                ---------- ---------- ---------- ---------- ---------- ---------- ---------- ---------- ---------- ---------- ----------
AADN03001784                ---------- ---------- ---------- ---------- ---------- ---------- ---------- ---------- ---------- ---------- ----------
AADN03001783                TCGGGGCTGG CCGCGGGCGC GGGCCGAGCG CCTTCGGGCA AGGCGAGAGA GAACGAGAGC GGTCCCCCCG CGCGCGCGGG GCGGTGCCGA AAGTCAGACA ACTCTTAGCG
DQ018754                    TCGGGGCTGG CCGCGGGCGC GGGCCGAGCG CCTTCGGGCA AGGCGAGAGA GAACGAGAGC GGTCCCCCCG CGCGCGCGGG GCGGTGCCGA AAGTCAGACA ACTCTTAGCG
AADN03001782                ---------- ---------- ---------- ---------- ---------- ---------- ---------- ---------- ---------- ---------- ----------
JN639848                    ---------- ---------- ---------- ---------- ---------- ---------- ---------- ---------- ---------- ---------- ----------
AADN03001670                TCGGGGCTGG CCGCGGGCGC GGGCGGAGCG CCTTCGGGCA AGGCGAGAGA GAACGAGAGC GGTCCCCCCG CGCGCGCGGG GCGGTGCCGA AAGTCAGACA ACTCTTAGCG
HQ873432                    ---------- ---------- ---------- ---------- ---------- ---------- ---------- ---------- ---------- ---------- ----------
AADN03001774                ---------- ---------- ---------- ---------- ---------- ---------- ---------- ---------- ---------- ---------- ----------
AADN03001775                ---------- ---------- ---------- ---------- ---------- ---------- ---------- ---------- ---------- ---------- ----------
EF552813                    ---------- ---------- ---------- ---------- ---------- ---------- ---------- ---------- ---------- ---------- ----------
AADN03022685                ---------- ---------- ---------- ---------- ---------- ---------- ---------- ---------- ---------- ---------- ----------
AADN03019346                ---------- ---------- ---------- ---------- ---------- ---------- ---------- ---------- ---------- ---------- ----------
AADN03001776                ---------- ---------- ---------- ---------- ---------- ---------- ---------- ---------- ---------- ---------- ----------
DQ018756                    ---------- ---------- ---------- ---------- ---------- ---------- ---------- ---------- ---------- ---------- ----------
AF173612                    ---------- ---------- ---------- ---------- ---------- ---------- ---------- ---------- ---------- ---------- ----------
Gallus_gallus_KT445934.2    TCGGGGCTGG CCGCGGGCGC GGGCCGAGCG CCTTCGGGCA AGGCGAGAGA GAACGAGAGC GGTCCCCCCG CGCGCGCGGG GCGGTGCCGA AAGTCAGACA ACTCTTAGCG

                            6666666666 6666666666 6666666666 6666666666 6666666666 6666666666 6666666666 6666666666 6666666666 6666666666 6666666666
                            2222222222 2222222222 2222222223 3333333333 3333333333 3333333333 3333333333 3333333333 3333333333 3333333333 3333333333
                            7777777778 8888888889 9999999990 0000000001 1111111112 2222222223 3333333334 4444444445 5555555556 6666666667 7777777778
                            1234567890 1234567890 1234567890 1234567890 1234567890 1234567890 1234567890 1234567890 1234567890 1234567890 1234567890
AADN03001677                ---------- ---------- ---------- ---------- ---------- ---------- ---------- ---------- ---------- ---------- ----------
AADN03001778                ---------- ---------- ---------- ---------- ---------- ---------- ---------- ---------- ---------- ---------- ----------
AADN03001785                ---------- ---------- ---------- ---------- ---------- ---------- ---------- ---------- ---------- ---------- ----------
AADN03001786                ---------- ---------- ---------- ---------- ---------- ---------- ---------- ---------- ---------- ---------- ----------
AADN03001788                ---------- ---------- ---------- ---------- ---------- ---------- ---------- ---------- ---------- ---------- ----------
AADN03014081                ---------- ---------- ---------- ---------- ---------- ---------- ---------- ---------- ---------- ---------- ----------
AADN03015064                ---------- ---------- ---------- ---------- ---------- ---------- ---------- ---------- ---------- ---------- ----------
AADN03026634                ---------- ---------- ---------- ---------- ---------- ---------- ---------- ---------- ---------- ---------- ----------
NW_003775878                ---------- ---------- ---------- ---------- ---------- ---------- ---------- ---------- ---------- ---------- ----------
DQ018752                    ---------- ---------- ---------- ---------- ---------- ---------- ---------- ---------- ---------- ---------- ----------
DQ018757                    ---------- ---------- ---------- ---------- ---------- ---------- ---------- ---------- ---------- ---------- ----------
DQ112354                    ---------- ---------- ---------- ---------- ---------- ---------- ---------- ---------- ---------- ---------- ----------
FM165414                    ---------- ---------- ---------- ---------- ---------- ---------- ---------- ---------- ---------- ---------- ----------
FM165415                    ---------- ---------- ---------- ---------- ---------- ---------- ---------- ---------- ---------- ---------- ----------
AADN03000430                ---------- ---------- ---------- ---------- ---------- ---------- ---------- ---------- ---------- ---------- ----------
```

```
AADN03001784               ---------- ---------- ---------- ---------- ---------- ---------- ---------- ---------- ---------- ---------- ----------
AADN03001783               GTGGATCACT CGGCTCGTGC GTCGATGAAG AACGCAGCTA GCTGCGAGAA TTAATGTGAA TTGCAGGACA CATTGATCAT CGACACTTCG AACGCACTTG CGGCCCCGGG
DQ018754                   GTGGATCACT CGGCTCGTGC G-CGATGAAG AACGCAGCTA GCTGCGAGAA CTAATGTGAA TTGCAGGACA CATTGATCAT CGACACTTCG AA-------- ----------
AADN03001782               ---------- ---------- ---------- ---------- ---------- ---------- ---------- ---------- ---------- ---------- ----------
JN639848                   ---------- ---------- ---------- ---------- ---------- ---------- ---------- ---------- ---------- ---------- ----------
AADN03001670               GTGGATCACT CGGCTCGTGC GTCGATGAAG AACGCAGCTA GCTGCGAGAA TTAATGTGAA TTGCAGGACA CATTGATCAT CGACACTTCG AACGCACTTG CGGCCCCGGG
HQ873432                   ---------- ---------- ---------- ---------- ---------- ---------- ---------- ---------- ---------- ---------- ----------
AADN03001774               ---------- ---------- ---------- ---------- ---------- ---------- ---------- ---------- ---------- ---------- ----------
AADN03001775               ---------- ---------- ---------- ---------- ---------- ---------- ---------- ---------- ---------- ---------- ----------
EF552813                   ---------- ---------- ---------- ---------- ---------- ---------- ---------- ---------- ---------- ---------- ----------
AADN03022685               ---------- ---------- ---------- ---------- ---------- ---------- ---------- ---------- ---------- ---------- ----------
AADN03019346               ---------- ---------- ---------- ---------- ---------- ---------- ---------- ---------- ---------- ---------- ----------
AADN03001776               ---------- ---------- ---------- ---------- ---------- ---------- ---------- ---------- ---------- ---------- ----------
DQ018756                   ---------- ---------- ---------- ---------- ---------- ---------- ---------- ---------- ---------- ---------- ----------
AF173612                   ---------- ---------- ---------- ---------- ---------- ---------- ---------- ---------- ---------- ---------- ----------
Gallus_gallus_KT445934.2   GTGGATCACT CGGCTCGTGC GTCGATGAAG AACGCAGCTA GCTGCGAGAA TTAATGTGAA TTGCAGGACA CATTGATCAT CGACACTTCG AACGCACTTG CGGCCCCGGG

                           6666666666 6666666666 6666666666 6666666666 6666666666 6666666666 6666666666 6666666666 6666666666 6666666666 6666666666
                           3333333333 3333333334 4444444444 4444444444 4444444444 4444444444 4444444444 4444444444 4444444444 4444444444 4444444444
                           8888888889 9999999990 0000000001 1111111112 2222222223 3333333334 4444444445 5555555556 6666666667 7777777778 8888888889
                           1234567890 1234567890 1234567890 1234567890 1234567890 1234567890 1234567890 1234567890 1234567890 1234567890 1234567890
AADN03001677               ---------- ---------- ---------- ---------- ---------- ---------- ---------- ---------- ---------- ---------- ----------
AADN03001778               ---------- ---------- ---------- ---------- ---------- ---------- ---------- ---------- ---------- ---------- ----------
AADN03001785               ---------- ---------- ---------- ---------- ---------- ---------- ---------- ---------- ---------- ---------- ----------
AADN03001786               ---------- ---------- ---------- ---------- ---------- ---------- ---------- ---------- ---------- ---------- ----------
AADN03001788               ---------- ---------- ---------- ---------- ---------- ---------- ---------- ---------- ---------- ---------- ----------
AADN03014081               ---------- ---------- ---------- ---------- ---------- ---------- ---------- ---------- ---------- ---------- ----------
AADN03015064               ---------- ---------- ---------- ---------- ---------- ---------- ---------- ---------- ---------- ---------- ----------
AADN03026634               ---------- ---------- ---------- ---------- ---------- ---------- ---------- ---------- ---------- ---------- ----------
NW_003775878               ---------- ---------- ---------- ---------- ---------- ---------- ---------- ---------- ---------- ---------- ----------
DQ018752                   ---------- ---------- ---------- ---------- ---------- ---------- ---------- ---------- ---------- ---------- ----------
DQ018757                   ---------- ---------- ---------- ---------- ---------- ---------- ---------- ---------- ---------- ---------- ----------
DQ112354                   ---------- ---------- ---------- ---------- ---------- ---------- ---------- ---------- ---------- ---------- ----------
FM165414                   ---------- ---------- ---------- ---------- ---------- ---------- ---------- ---------- ---------- ---------- ----------
FM165415                   ---------- ---------- ---------- ---------- ---------- ---------- ---------- ---------- ---------- ---------- ----------
AADN03000430               ---------- ---------- ---------- ---------- ---------- ---------- ---------- ---------- ---------- ---------- ----------
AADN03001784               ---------- ---------- ---------- ---------- ---------- ---------- ---------- ---------- ---------- ---------- ----------
AADN03001783               TTCCTCCCGG GGCTACGCCT GCCTGAGCGT CGCTTGACGG TCAATCGCCG ACGGCCGCCG TCCGCGGCGG CCGCGCGGCG CGGCTGGGGC GCCTCGCAGG CCCGCGCGCC
DQ018754                   ---------- ---------- ---------- ---------- ---------- ---------- ---------- ---------- ---------- ---------- ----------
AADN03001782               ---------- ---------- ---------- ---------- ---------- ---------- ---------- ---------- ---------- ---------- ----------
JN639848                   ---------- ---------- ---------- ---------- ---------- ---------- ---------- ---------- ---------- ---------- ----------
AADN03001670               TTCCTCCCGG GGCTACGCCT GCC------- ---------- ---------- ---------- ---------- ---------- ---------- ---------- ----------
HQ873432                   ---------- ---------- ---------- ---------- ---------- ---------- ---------- ---------- ---------- ---------- ----------
AADN03001774               ---------- ---------- ---------- ---------- ---------- ---------- ---------- ---------- ---------- ---------- ----------
AADN03001775               ---------- ---------- ---------- ---------- ---------- ---------- ---------- ---------- ---------- ---------- ----------
EF552813                   ---------- ---------- ---------- ---------- ---------- ---------- ---------- ---------- ---------- ---------- ----------
AADN03022685               ---------- ---------- ---------- ---------- ---------- ---------- ---------- ---------- ---------- ---------- ----------
AADN03019346               ---------- ---------- ---------- ---------- ---------- ---------- ---------- ---------- ---------- ---------- ----------
AADN03001776               ---------- ---------- ---------- ---------- ---------- ---------- ---------- ---------- ---------- ---------- ----------
DQ018756                   ---------- ---------- ---------- ---------- ---------- ---------- ---------- ---------- ---------- ---------- ----------
AF173612                   ---------- ---------- ---------- ---------- ---------- ---------- ---------- ---------- ---------- ---------- ----------
Gallus_gallus_KT445934.2   TTCCTCCCGG GGCTACGCCT GCCTGAGCGT CGCTTGACGG TCAATCGCCG ATGGCCGCCG TCCGCGGCGG CCGCGCGGCG CGGCTGGGGC GCCTCGCAGG CCCGCGCGCC
```

```
                            6666666666 6666666666 6666666666 6666666666 6666666666 6666666666 6666666666 6666666666 6666666666 6666666666 6666666666
                            4444444445 5555555555 5555555555 5555555555 5555555555 5555555555 5555555555 5555555555 5555555555 5555555555 5555555556
                            9999999990 0000000001 1111111112 2222222223 3333333334 4444444445 5555555556 6666666667 7777777778 8888888889 9999999990
                            1234567890 1234567890 1234567890 1234567890 1234567890 1234567890 1234567890 1234567890 1234567890 1234567890 1234567890
AADN03001677                ---------- ---------- ---------- ---------- ---------- ---------- ---------- ---------- ---------- ---------- ----------
AADN03001778                ---------- ---------- ---------- ---------- ---------- ---------- ---------- ---------- ---------- ---------- ----------
AADN03001785                ---------- ---------- ---------- ---------- ---------- ---------- ---------- ---------- ---------- ---------- ----------
AADN03001786                ---------- ---------- ---------- ---------- ---------- ---------- ---------- ---------- ---------- ---------- ----------
AADN03001788                ---------- ---------- ---------- ---------- ---------- ---------- ---------- ---------- ---------- ---------- ----------
AADN03014081                ---------- ---------- ---------- ---------- ---------- ---------- ---------- ---------- ---------- ---------- ----------
AADN03015064                ---------- ---------- ---------- ---------- ---------- ---------- ---------- ---------- ---------- ---------- ----------
AADN03026634                ---------- ---------- ---------- ---------- ---------- ---------- ---------- ---------- ---------- ---------- ----------
NW_003775878                ---------- ---------- ---------- ---------- ---------- ---------- ---------- ---------- ---------- ---------- ----------
DQ018752                    ---------- ---------- ---------- ---------- ---------- ---------- ---------- ---------- ---------- ---------- ----------
DQ018757                    ---------- ---------- ---------- ---------- ---------- ---------- ---------- ---------- ---------- ---------- ----------
DQ112354                    ---------- ---------- ---------- ---------- ---------- ---------- ---------- ---------- ---------- ---------- ----------
FM165414                    ---------- ---------- ---------- ---------- ---------- ---------- ---------- ---------- ---------- ---------- ----------
FM165415                    ---------- ---------- ---------- ---------- ---------- ---------- ---------- ---------- ---------- ---------- ----------
AADN03000430                ---------- ---------- ---------- ---------- ---------- ---------- ---------- ---------- ---------- ---------- ----------
AADN03001784                ---------- ---------- ---------- ---------- ---------- ---------- ---------- ---------- ---------- ---------- ----------
AADN03001783                CCGCCGGAGG CGGGTCGCGA GGGGGGGGGC CGCCGTCCGT CCGTCCGTCC GCCCGTCGGT CGGTCGGTTC GGGCGCCCGG ATTCCCTCCC CCGCACCCCC TCCGAGCGGC
DQ018754                    ---------- ---------- ---------- ---------- ---------- ---------- ---------- ---------- ---------- ---------- ----------
AADN03001782                ---------- ---------- ---------- ---------- ---------- ---------- ---------- ---------- ---------- ---------- ----------
JN639848                    ---------- ---------- ---------- ---------- ---------- ---------- ---------- ---------- ---------- ---------- ----------
AADN03001670                ---------- ---------- ---------- ---------- ---------- ---------- ---------- ---------- ---------- ---------- ----------
HQ873432                    ---------- ---------- ---------- ---------- ---------- ---------- ---------- ---------- ---------- ---------- ----------
AADN03001774                ---------- ---------- ---------- ---------- ---------- ---------- ---------- ---------- ---------- ---------- ----------
AADN03001775                ---------- ---------- ---------- ---------- ---------- ---------- ---------- ---------- ---------- ---------- ----------
EF552813                    ---------- ---------- ---------- ---------- ---------- ---------- ---------- ---------- ---------- ---------- ----------
AADN03022685                ---------- ---------- ---------- ---------- ---------- ---------- ---------- ---------- ---------- ---------- ----------
AADN03019346                ---------- ---------- ---------- ---------- ---------- ---------- ---------- ---------- ---------- ---------- ----------
AADN03001776                ---------- ---------- ---------- ---------- ---------- ---------- ---------- ---------- ---------- ---------- ----------
DQ018756                    ---------- ---------- ---------- ---------- ---------- ---------- ---------- ---------- ---------- ---------- ----------
AF173612                    ---------- ---------- ---------- ---------- ---------- ---------- ---------- ---------- ---------- ---------- ----------
Gallus_gallus_KT445934.2    CCGCCGGAGG CGGGTCGCGA GGGGGGGGGC CGCCGTCCGT CCGTCCGTCC GCCCGTCGGT CGGTCGGTTC GGGCGCCCGG ATTCCCTCCC CCGCACCCCC TCCGAGCGGC

                            6666666666 6666666666 6666666666 6666666666 6666666666 6666666666 6666666666 6666666666 6666666666 6666666666 6666666666
                            6666666666 6666666666 6666666666 6666666666 6666666666 6666666666 6666666666 6666666666 6666666666 6666666667 7777777777
                            0000000001 1111111112 2222222223 3333333334 4444444445 5555555556 6666666667 7777777778 8888888889 9999999990 0000000001
                            1234567890 1234567890 1234567890 1234567890 1234567890 1234567890 1234567890 1234567890 1234567890 1234567890 1234567890
AADN03001677                ---------- ---------- ---------- ---------- ---------- ---------- ---------- ---------- ---------- ---------- ----------
AADN03001778                ---------- ---------- ---------- ---------- ---------- ---------- ---------- ---------- ---------- ---------- ----------
AADN03001785                ---------- ---------- ---------- ---------- ---------- ---------- ---------- ---------- ---------- ---------- ----------
AADN03001786                ---------- ---------- ---------- ---------- ---------- ---------- ---------- ---------- ---------- ---------- ----------
AADN03001788                ---------- ---------- ---------- ---------- ---------- ---------- ---------- ---------- ---------- ---------- ----------
AADN03014081                ---------- ---------- ---------- ---------- ---------- ---------- ---------- ---------- ---------- ---------- ----------
AADN03015064                ---------- ---------- ---------- ---------- ---------- ---------- ---------- ---------- ---------- ---------- ----------
AADN03026634                ---------- ---------- ---------- ---------- ---------- ---------- ---------- ---------- ---------- ---------- ----------
NW_003775878                ---------- ---------- ---------- ---------- ---------- ---------- ---------- ---------- ---------- ---------- ----------
DQ018752                    ---------- ---------- ---------- ---------- ---------- ---------- ---------- ---------- ---------- ---------- ----------
DQ018757                    ---------- ---------- ---------- ---------- ---------- ---------- ---------- ---------- ---------- ---------- ----------
DQ112354                    ---------- ---------- ---------- ---------- ---------- ---------- ---------- ---------- ---------- ---------- ----------
FM165414                    ---------- ---------- ---------- ---------- ---------- ---------- ---------- ---------- ---------- ---------- ----------
FM165415                    ---------- ---------- ---------- ---------- ---------- ---------- ---------- ---------- ---------- ---------- ----------
AADN03000430                ---------- ---------- ---------- ---------- ---------- ---------- ---------- ---------- ---------- ---------- ----------
```

```
AADN03001784             ---------- ---------- ---------- ---------- ---------- ---------- ---------- ---------- ---------- ---------- ----------
AADN03001783             GTCGCGCCGC GGGCCTTCGT CCCCCTAAGT GGAGACCCAG GTCGGGGAGC TCGCCGAGCT CCCCGCGCTC CCGGAGCGCC CGCTTTGGCC GAGCTCGTCC CCACGGGGCG
DQ018754                 ---------- ---------- ---------- ---------- ---------- ---------- ---------- ---------- ---------- ---------- ----------
AADN03001782             ---------- ---------- ---------- ---------- ---------- ---------- ---------- ---------- ---------- ---------- ----------
JN639848                 ---------- ---------- ---------- ---------- ---------- ---------- ---------- ---------- ---------- ---------- ----------
AADN03001670             ---------- ---------- ---------- ---------- ---------- ---------- ---------- ---------- ---------- ---------- ----------
HQ873432                 ---------- ---------- ---------- ---------- ---------- ---------- ---------- ---------- ---------- ---------- ----------
AADN03001774             ---------- ---------- ---------- ---------- ---------- ---------- ---------- ---------- ---------- ---------- ----------
AADN03001775             ---------- ---------- ---------- ---------- ---------- ---------- ---------- ---------- ---------- ---------- ----------
EF552813                 ---------- ---------- ---------- ---------- ---------- ---------- ---------- ---------- ---------- ---------- ----------
AADN03022685             ---------- ---------- ---------- ---------- ---------- ---------- ---------- ---------- ---------- ---------- ----------
AADN03019346             ---------- ---------- ---------- ---------- ---------- ---------- ---------- ---------- ---------- ---------- ----------
AADN03001776             ---------- ---------- ---------- ---------- ---------- ---------- ---------- ---------- ---------- ---------- ----------
DQ018756                 ---------- ---------- ---------- ---------- ---------- ---------- ---------- ---------- ---------- ---------- ----------
AF173612                 ---------- ---------- ---------- ---------- ---------- ---------- ---------- ---------- ---------- ---------- ----------
Gallus_gallus_KT445934.2 GTCGCGCCGC GGGCCTTCGT CCCCCTAAGT GGAGACCCAG GTCGGGGAGC TCGCCGAGCT CCCCGCGCTC CCGGAGCGCC CGCTTTGGCC GAGCTCGTCC CCACGGGGCG

                         6666666666 6666666666 6666666666 6666666666 6666666666 6666666666 6666666666 6666666666 6666666666 6666666666 6666666666
                         7777777777 7777777777 7777777777 7777777777 7777777777 7777777777 7777777777 7777777777 7777777778 8888888888 8888888888
                         1111111112 2222222223 3333333334 4444444445 5555555556 6666666667 7777777778 8888888889 9999999990 0000000001 1111111112
                         1234567890 1234567890 1234567890 1234567890 1234567890 1234567890 1234567890 1234567890 1234567890 1234567890 1234567890
AADN03001677             ---------- ---------- ---------- ---------- ---------- ---------- ---------- ---------- ---------- ---------- ----------
AADN03001778             ---------- ---------- ---------- ---------- ---------- ---------- ---------- ---------- ---------- ---------- ----------
AADN03001785             ---------- ---------- ---------- ---------- ---------- ---------- ---------- ---------- ---------- ---------- ----------
AADN03001786             ---------- ---------- ---------- ---------- ---------- ---------- ---------- ---------- ---------- ---------- ----------
AADN03001788             ---------- ---------- ---------- ---------- ---------- ---------- ---------- ---------- ---------- ---------- ----------
AADN03014081             ---------- ---------- ---------- ---------- ---------- ---------- ---------- ---------- ---------- ---------- ----------
AADN03015064             ---------- ---------- ---------- ---------- ---------- ---------- ---------- ---------- ---------- ---------- ----------
AADN03026634             ---------- ---------- ---------- ---------- ---------- ---------- ---------- ---------- ---------- ---------- ----------
NW_003775878             ---------- ---------- ---------- ---------- ---------- ---------- ---------- ---------- ---------- ---------- ----------
DQ018752                 ---------- ---------- ---------- ---------- ---------- ---------- ---------- ---------- ---------- ---------- ----------
DQ018757                 ---------- ---------- ---------- ---------- ---------- ---------- ---------- ---------- ---------- ---------- ----------
DQ112354                 ---------- ---------- ---------- ---------- ---------- ---------- ---------- ---------- ---------- ---------- ----------
FM165414                 ---------- ---------- ---------- ---------- ---------- ---------- ---------- ---------- ---------- ---------- ----------
FM165415                 ---------- ---------- ---------- ---------- ---------- ---------- ---------- ---------- ---------- ---------- ----------
AADN03000430             ---------- ---------- ---------- ---------- ---------- ---------- ---------- ---------- ---------- ---------- ----------
AADN03001784             ---------- ---------- ---------- ---------- ---------- ---------- ---------- ---------- ---------C ACCTCGCCCT CCCCGGCCCC
AADN03001783             GCCGGGCTTT CCGGTCGGTC GCGCGGCGCA GCGCGGCGGG GCCGGACGTT CGTTCGTTCG TTCGTCCGGC CCCCCGCCCC GGAGGAGCGC ACCTCGCCCT CCCCGGCCCC
DQ018754                 ---------- ---------- ---------- ---------- ---------- ---------- ---------- ---------- ---------- ---------- ----------
AADN03001782             ---------- ---------- ---------- ---------- ---------- ---------- ---------- ---------- ---------- ---------- ----------
JN639848                 ---------- ---------- ---------- ---------- ---------- ---------- ---------- ---------- ---------- ---------- ----------
AADN03001670             ---------- ---------- ---------- ---------- ---------- ---------- ---------- ---------- ---------- ---------- ----------
HQ873432                 ---------- ---------- ---------- ---------- ---------- ---------- ---------- ---------- ---------- ---------- ----------
AADN03001774             ---------- ---------- ---------- ---------- ---------- ---------- ---------- ---------- ---------- ---------- ----------
AADN03001775             ---------- ---------- ---------- ---------- ---------- ---------- ---------- ---------- ---------- ---------- ----------
EF552813                 ---------- ---------- ---------- ---------- ---------- ---------- ---------- ---------- ---------- ---------- ----------
AADN03022685             ---------- ---------- ---------- ---------- ---------- ---------- ---------- ---------- ---------- ---------- ----------
AADN03019346             ---------- ---------- ---------- ---------- ---------- ---------- ---------- ---------- ---------- ---------- ----------
AADN03001776             ---------- ---------- ---------- ---------- ---------- ---------- ---------- ---------- ---------- ---------- ----------
DQ018756                 ---------- ---------- ---------- ---------- ---------- ---------- ---------- ---------- ---------- ---------- ----------
AF173612                 ---------- ---------- ---------- ---------- ---------- ---------- ---------- ---------- ---------- ---------- ----------
Gallus_gallus_KT445934.2 GCCGGGCTTT CCGGTCGGTC GCGCGGCGCA GCGCGGCGGG GCCGGACGTT CGTTCGTTCG TTCGTCCGGC CCCCCGCCCC GGAGGAGCGC TCCTCGCCCT CCCCGGCCCC
```

```
                           6666666666 6666666666 6666666666 6666666666 6666666666 6666666666 6666666666 6666666666 6666666666 6666666666 6666666666
                           8888888888 8888888888 8888888888 8888888888 8888888888 8888888888 8888888888 8888888889 9999999999 9999999999 9999999999
                           2222222223 3333333334 4444444445 5555555556 6666666667 7777777778 8888888889 9999999990 0000000001 1111111112 2222222223
                           1234567890 1234567890 1234567890 1234567890 1234567890 1234567890 1234567890 1234567890 1234567890 1234567890 1234567890
AADN03001677               ---------- ---------- ---------- ---------- ---------- ---------- ---------- ---------- ---------- ---------- ----------
AADN03001778               ---------- ---------- ---------- ---------- ---------- ---------- ---------- ---------- ---------- ---------- ----------
AADN03001785               ---------- ---------- ---------- ---------- ---------- ---------- ---------- ---------- ---------- ---------- ----------
AADN03001786               ---------- ---------- ---------- ---------- ---------- ---------- ---------- ---------- ---------- ---------- ----------
AADN03001788               ---------- ---------- ---------- ---------- ---------- ---------- ---------- ---------- ---------- ---------- ----------
AADN03014081               ---------- ---------- ---------- ---------- ---------- ---------- ---------- ---------- ---------- ---------- ----------
AADN03015064               ---------- ---------- ---------- ---------- ---------- ---------- ---------- ---------- ---------- ---------- ----------
AADN03026634               ---------- ---------- ---------- ---------- ---------- ---------- ---------- ---------- ---------- ---------- ----------
NW_003775878               ---------- ---------- ---------- ---------- ---------- ---------- ---------- ---------- ---------- ---------- ----------
DQ018752                   ---------- ---------- ---------- ---------- ---------- ---------- ---------- ---------- ---------- ---------- ----------
DQ018757                   ---------- ---------- ---------- ---------- ---------- ---------- ---------- ---------- ---------- ---------- ----------
DQ112354                   ---------- ---------- ---------- ---------- ---------- ---------- ---------- ---------- ---------- ---------- ----------
FM165414                   ---------- ---------- ---------- ---------- ---------- ---------- ---------- ---------- ---------- ---------- ----------
FM165415                   ---------- ---------- ---------- ---------- ---------- ---------- ---------- ---------- ---------- ---------- ----------
AADN03000430               ---------- ---------- ---------- ---------- ---------- ---------- ---------- ---------- ---------- ---------- ----------
AADN03001784               GCGCGCGGCT GCATGCGGGT GGCGTTACCG GCGGCGGTAA CGCGCCGTGC TGCCGCGCGC GTGGCGGTCC GGGTCGGGGC GAGGCTGCCG GCCTCCGGTC GTCCGCCCGT
AADN03001783               GCGCGCGGCT GCCTGCGGGT CGCGTTACCG GCGGCGGTAA CGCGCCGTGC TGCCGCGCGC GTGGCGGTCC GGGTCGGGGC GAGGCTGCCG GCCTCCGGTC GTCCGCCCGT
DQ018754                   ---------- ---------- ---------- ---------- ---------- ---------- ---------- ---------- ---------- ---------- ----------
AADN03001782               ---------- ---------- ---------- ---------- ---------- ---------- ---------- ---------- ---------- ---------- ----------
JN639848                   ---------- ---------- ---------- ---------- ---------- ---------- ---------- ---------- ---------- ---------- ----------
AADN03001670               ---------- ---------- ---------- ---------- ---------- ---------- ---------- ---------- ---------- ---------- ----------
HQ873432                   ---------- ---------- ---------- ---------- ---------- ---------- ---------- ---------- ---------- ---------- ----------
AADN03001774               ---------- ---------- ---------- ---------- ---------- ---------- ---------- ---------- ---------- ---------- ----------
AADN03001775               ---------- ---------- ---------- ---------- ---------- ---------- ---------- ---------- ---------- ---------- ----------
EF552813                   ---------- ---------- ---------- ---------- ---------- ---------- ---------- ---------- ---------- ---------- ----------
AADN03022685               ---------- ---------- ---------- ---------- ---------- ---------- ---------- ---------- ---------- ---------- ----------
AADN03019346               ---------- ---------- ---------- ---------- ---------- ---------- ---------- ---------- ---------- ---------- ----------
AADN03001776               ---------- ---------- ---------- ---------- ---------- ---------- ---------- ---------- ---------- ---------- ----------
DQ018756                   ---------- ---------- ---------- ---------- ---------- ---------- ---------- ---------- ---------- ---------- ----------
AF173612                   ---------- ---------- ---------- ---------- ---------- ---------- ---------- ---------- ---------- ---------- ----------
Gallus_gallus_KT445934.2   GCGCGCGGCT GCCTGCGGGT CGCGTTACCG GCGGCGGTAA CGCGCCGTGC TGCCGCGCGC GTGGCGGTCC GGGTCGGGGC GAGGCTGCCG GCCTCCGGTC GTCCGCCCGT

                           6666666666 6666666666 6666666666 6666666666 6666666666 6666666666 6666666667 7777777777 7777777777 7777777777 7777777777
                           9999999999 9999999999 9999999999 9999999999 9999999999 9999999999 9999999990 0000000000 0000000000 0000000000 0000000000
                           3333333334 4444444445 5555555556 6666666667 7777777778 8888888889 9999999990 0000000001 1111111112 2222222223 3333333334
                           1234567890 1234567890 1234567890 1234567890 1234567890 1234567890 1234567890 1234567890 1234567890 1234567890 1234567890
AADN03001677               ---------- ---------- ---------- ---------- ---------- ---------- ---------- ---------- ---------- ---------- ----------
AADN03001778               ---------- ---------- ---------- ---------- ---------- ---------- ---------- ---------- ---------- ---------- ----------
AADN03001785               ---------- ---------- ---------- ---------- ---------- ---------- ---------- ---------- ---------- ---------- ----------
AADN03001786               ---------- ---------- ---------- ---------- ---------- ---------- ---------- ---------- ---------- ---------- ----------
AADN03001788               ---------- ---------- ---------- ---------- ---------- ---------- ---------- ---------- ---------- ---------- ----------
AADN03014081               ---------- ---------- ---------- ---------- ---------- ---------- ---------- ---------- ---------- ---------- ----------
AADN03015064               ---------- ---------- ---------- ---------- ---------- ---------- ---------- ---------- ---------- ---------- ----------
AADN03026634               ---------- ---------- ---------- ---------- ---------- ---------- ---------- ---------- ---------- ---------- ----------
NW_003775878               ---------- ---------- ---------- ---------- ---------- ---------- ---------- ---------- ---------- ---------- ----------
DQ018752                   ---------- ---------- ---------- ---------- ---------- ---------- ---------- ---------- ---------- ---------- ----------
DQ018757                   ---------- ---------- ---------- ---------- ---------- ---------- ---------- ---------- ---------- ---------- ----------
DQ112354                   ---------- ---------- ---------- ---------- ---------- ---------- ---------- ---------- ---------- ---------- ----------
FM165414                   ---------- ---------- ---------- ---------- ---------- ---------- ---------- ---------- ---------- ---------- ----------
FM165415                   ---------- ---------- ---------- ---------- ---------- ---------- ---------- ---------- ---------- ---------- ----------
AADN03000430               ---------- ---------- ---------- ---------- ---------- ---------- ---------- ---------- ---------- ---------- ----------
```

```
AADN03001784                CCGTCCGGCC GAGCCCGGCG CGCGTCCCCG CGGGTCCGTC TCCGGCCACC GTGCGCCGGC GGCGGCGGCG GCGGTGCGAA CCGCCGGCGG CGCGCCGGCT CCCCCGTCCG
AADN03001783                CCGTCCGGCC GAGCCCGGCG CGCGTCCCCG CGGGTCCGTC TCCGGCCACC GTGCGCCGGC GGCGGCGGCG GCGGTGCGAA CCGCCGGCGG CGCGCCGGCT CCCCCGTCCG
DQ018754                    ---------- ---------- ---------- ---------- ---------- ---------- ---------- ---------- ---------- ---------- ----------
AADN03001782                ---------- ---------- ---------- ---------- ---------- ---------- ---------- ---------- ---------- ---------- ----------
JN639848                    ---------- ---------- ---------- ---------- ---------- ---------- ---------- ---------- ---------- ---------- ----------
AADN03001670                ---------- ---------- ---------- ---------- ---------- ---------- ---------- ---------- ---------- ---------- ----------
HQ873432                    ---------- ---------- ---------- ---------- ---------- ---------- ---------- ---------- ---------- ---------- ----------
AADN03001774                ---------- ---------- ---------- ---------- ---------- ---------- ---------- ---------- ---------- ---------- ----------
AADN03001775                ---------- ---------- ---------- ---------- ---------- ---------- ---------- ---------- ---------- ---------- ----------
EF552813                    ---------- ---------- ---------- ---------- ---------- ---------- ---------- ---------- ---------- ---------- ----------
AADN03022685                ---------- ---------- ---------- ---------- ---------- ---------- ---------- ---------- ---------- ---------- ----------
AADN03019346                ---------- ---------- ---------- ---------- ---------- ---------- ---------- ---------- ---------- ---------- ----------
AADN03001776                ---------- ---------- ---------- ---------- ---------- ---------- ---------- ---------- ---------- ---------- ----------
DQ018756                    ---------- ---------- ---------- ---------- ---------- ---------- ---------- ---------- ---------- ---------- ----------
AF173612                    ---------- ---------- ---------- ---------- ---------- ---------- ---------- ---------- ---------- ---------- ----------
Gallus_gallus_KT445934.2    CCGTCCGGCC GAGCCCGGCG CGCGTCCCCG CGGGTCCGTC TCCGGCCACC GTGCGCCGGC GGCGGCGGCG GCGGTGCGAA CCGCCGGCGG CGCGCCGGCT CCCCCGTCCG

                            7777777777 7777777777 7777777777 7777777777 7777777777 7777777777 7777777777 7777777777 7777777777 7777777777 7777777777
                            0000000000 0000000000 0000000000 0000000000 0000000000 0000000001 1111111111 1111111111 1111111111 1111111111 1111111111
                            4444444445 5555555556 6666666667 7777777778 8888888889 9999999990 0000000001 1111111112 2222222223 3333333334 4444444445
                            1234567890 1234567890 1234567890 1234567890 1234567890 1234567890 1234567890 1234567890 1234567890 1234567890 1234567890
AADN03001677                ---------- ---------- ---------- ---------- ---------- ---------- ---------- ---------- ---------- ---------- ----------
AADN03001778                ---------- ---------- ---------- ---------- ---------- ---------- ---------- ---------- ---------- ---------- ----------
AADN03001785                ---------- ---------- ---------- ---------- ---------- ---------- ---------- ---------- ---------- ---------- ----------
AADN03001786                ---------- ---------- ---------- ---------- ---------- ---------- ---------- ---------- ---------- ---------- ----------
AADN03001788                ---------- ---------- ---------- ---------- ---------- ---------- ---------- ---------- ---------- ---------- ----------
AADN03014081                ---------- ---------- ---------- ---------- ---------- ---------- ---------- ---------- ---------- ---------- ----------
AADN03015064                ---------- ---------- ---------- ---------- ---------- ---------- ---------- ---------- ---------- ---------- ----------
AADN03026634                ---------- ---------- ---------- ---------- ---------- ---------- ---------- ---------- ---------- ---------- ----------
NW_003775878                ---------- ---------- ---------- ---------- ---------- ---------- ---------- ---------- ---------- ---------- ----------
DQ018752                    ---------- ---------- ---------- ---------- ---------- ---------- ---------- ---------- ---------- ---------- ----------
DQ018757                    ---------- ---------- ---------- ---------- ---------- ---------- ---------- ---------- ---------- ---------- ----------
DQ112354                    ---------- ---------- ---------- ---------- ---------- ---------- ---------- ---------- ---------- ---------- ----------
FM165414                    ---------- ---------- ---------- ---------- ---------- ---------- ---------- ---------- ---------- ---------- ----------
FM165415                    ---------- ---------- ---------- ---------- ---------- ---------- ---------- ---------- ---------- ---------- ----------
AADN03000430                ---------- ---------- ---------- ---------- ---------- ---------- ---------- ---------- ---------- ---------- ----------
AADN03001784                GGCGTTCCTC CCTCGGCAGC GCCGGGAGCA GCCGCTTGGC GTCCGAAGGC GGGTGGCCGG GCGAGCGCGG GCTCGCCCGG GGCCCGGCGT TCGGGCCCCG TTTTCCGATC
AADN03001783                GGCGT----- ---------- ---------- ---------- ---------- ---------- ---------- ---------- ---------- ---------- ----------
DQ018754                    ---------- ---------- ---------- ---------- ---------- ---------- ---------- ---------- ---------- ---------- ----------
AADN03001782                ---------- ---------- ---------- ---------- ---------- ---------- ---------- ---------- ---------- ---------- ----------
JN639848                    ---------- ---------- ---------- ---------- ---------- ---------- ---------- ---------- ---------- ---------- ----------
AADN03001670                ---------- ---------- ---------- ---------- ---------- ---------- ---------- ---------- ---------- ---------- ----------
HQ873432                    ---------- ---------- ---------- ---------- ---------- ---------- ---------- ---------- ---------- ---------- ----------
AADN03001774                ---------- ---------- ---------- ---------- ---------- ---------- ---------- ---------- ---------- ---------- ----------
AADN03001775                ---------- ---------- ---------- ---------- ---------- ---------- ---------- ---------- ---------- ---------- ----------
EF552813                    ---------- ---------- ---------- ---------- ---------- ---------- ---------- ---------- ---------- ---------- ----------
AADN03022685                ---------- ---------- ---------- ---------- ---------- ---------- ---------- ---------- ---------- ---------- ----------
AADN03019346                ---------- ---------- ---------- ---------- ---------- ---------- ---------- ---------- ---------- ---------- ----------
AADN03001776                ---------- ---------- ---------- ---------- ---------- ---------- ---------- ---------- ---------- ---------- ----------
DQ018756                    ---------- ---------- ---------- ---------- ---------- ---------- ---------- ---------- ---------- ---------- ----------
AF173612                    ---------- ---------- ---------- ---------- ---------- ---------- ---------- ---------- ---------- ---------- ----------
Gallus_gallus_KT445934.2    GGCGTTCCTC CCTCGGCAGC GCCGGGAGCA GCCGCTTGGC GTCCGAAGGC GGGTGGCCGG GCGAGCGCGG GCTCGCCCGG GGCCCGGCGT TCGGGCCCCG TTT-CCGATC
```

```
                           7777777777 7777777777 7777777777 7777777777 7777777777 7777777777 7777777777 7777777777 7777777777 7777777777 7777777777
                           1111111111 1111111111 1111111111 1111111111 1111111112 2222222222 2222222222 2222222222 2222222222 2222222222 2222222222
                           5555555556 6666666667 7777777778 8888888889 9999999990 0000000001 1111111112 2222222223 3333333334 4444444445 5555555556
                           1234567890 1234567890 1234567890 1234567890 1234567890 1234567890 1234567890 1234567890 1234567890 1234567890 1234567890
AADN03001677               ---------- ---------- ---------- ---------- ---------- ---------- ---------- ---------- ---------- ---------- ----------
AADN03001778               ---------- ---------- ---------- ---------- ---------- ---------- ---------- ---------- ---------- ---------- ----------
AADN03001785               ---------- ---------- ---------- ---------- ---------- ---------- ---------- ---------- ---------- ---------- ----------
AADN03001786               ---------- ---------- ---------- ---------- ---------- ---------- ---------- ---------- ---------- ---------- ----------
AADN03001788               ---------- ---------- ---------- ---------- ---------- ---------- ---------- ---------- ---------- ---------- ----------
AADN03014081               ---------- ---------- ---------- ---------- ---------- ---------- ---------- ---------- ---------- ---------- ----------
AADN03015064               ---------- ---------- ---------- ---------- ---------- ---------- ---------- ---------- ---------- ---------- ----------
AADN03026634               ---------- ---------- ---------- ---------- ---------- ---------- ---------- ---------- ---------- ---------- ----------
NW_003775878               ---------- ---------- ---------- ---------- ---------- ---------- ---------- ---------- ---------- ---------- ----------
DQ018752                   ---------- ---------- ---------- ---------- ---------- ---------- ---------- ---------- ---------- ---------- ----------
DQ018757                   ---------- ---------- ---------- ---------- ---------- ---------- ---------- ---------- ---------- ---------- ----------
DQ112354                   ---------- ---------- ---------- ---------- ---------- ---------- ---------- ---------- ---------- ---------- ----------
FM165414                   ---------- ---------- ---------- ---------- ---------- ---------- ---------- ---------- ---------- ---------- ----------
FM165415                   ---------- ---------- ---------- ---------- ---------- ---------- ---------- ---------- ---------- ---------- ----------
AADN03000430               ---------- ---------- ---------- ---------- ---------- ---------- ---------- ---------- ---------- ---------- ----------
AADN03001784               GCGACCTCAG GTCAGACGTG GCGACCCGCT GAATTTAAGC ATATTAGTCA GCGGAGGAAA AGAAACTAAC GAGGATTCCC TCAGTAACGG CGAGTGAAGA GGGAAGAGCC
AADN03001783               ---------- ---------- ---------- ---------- ---------- ---------- ---------- ---------- ---------- ---------- ----------
DQ018754                   ---------- ---------- ---------- ---------- ---------- ---------- ---------- ---------- ---------- ---------- ----------
AADN03001782               ---------- ---------- ---------- ---------- ---------- ---------- ---------- ---------- ---------- ---------- ----------
JN639848                   ---------- ---------- ---------- ---------- ---------- ---------- ---------- ---------- ---------- ---------- ----------
AADN03001670               ---------- ---------- ---------- ---------- ---------- ---------- ---------- ---------- ---------- ---------- ----------
HQ873432                   ---------- ---------- ---------- ---------- ---------- ---------- ---------- ---------- ---------- ---------- ----------
AADN03001774               ---------- ---------- ---------- ---------- ---------- ---------- ---------- ---------- ---------- ---------- ----------
AADN03001775               ---------- ---------- ---------- ---------- ---------- ---------- ---------- ---------- ---------- ---------- ----------
EF552813                   ---------- ---------- ---------- ---------- ---------- ---------- ---------- ---------- ---------- ---------- ----------
AADN03022685               ---------- ---------- ---------- ---------- ---------- ---------- ---------- ---------- ---------- ---------- ----------
AADN03019346               ---------- ---------- ---------- ---------- ---------- ---------- ---------- ---------- ---------- ---------- ----------
AADN03001776               ---------- ---------- ---------- ---------- ---------- ---------- ---------- ---------- ---------- ---------- ----------
DQ018756                   ---------- ---------- ---------- ---------- ---------- ---------- ---------- ---------- ---------- ---------- ----------
AF173612                   ---------- ---------- ---------- ---------- ---------- ---------- ---------- ---------- ---------- ---------- ----------
Gallus_gallus_KT445934.2   GCGACCTCAG GTCAGACGTG GCGACCCGCT GAATTTAAGC ATATTAGTCA GCGGAGGAAA AGAAACTAAC GAGGATTCCC TCAGTAACGG CGAGTGAAGA GGGAAGAGCC

                           7777777777 7777777777 7777777777 7777777777 7777777777 7777777777 7777777777 7777777777 7777777777 7777777777 7777777777
                           2222222222 2222222222 2222222222 2222222223 3333333333 3333333333 3333333333 3333333333 3333333333 3333333333 3333333333
                           6666666667 7777777778 8888888889 9999999990 0000000001 1111111112 2222222223 3333333334 4444444445 5555555556 6666666667
                           1234567890 1234567890 1234567890 1234567890 1234567890 1234567890 1234567890 1234567890 1234567890 1234567890 1234567890
AADN03001677               ---------- ---------- ---------- ---------- ---------- ---------- ---------- ---------- ---------- ---------- ----------
AADN03001778               ---------- ---------- ---------- ---------- ---------- ---------- ---------- ---------- ---------- ---------- ----------
AADN03001785               ---------- ---------- ---------- ---------- ---------- ---------- ---------- ---------- ---------- ---------- ----------
AADN03001786               ---------- ---------- ---------- ---------- ---------- ---------- ---------- ---------- ---------- ---------- ----------
AADN03001788               ---------- ---------- ---------- ---------- ---------- ---------- ---------- ---------- ---------- ---------- ----------
AADN03014081               ---------- ---------- ---------- ---------- ---------- ---------- ---------- ---------- ---------- ---------- ----------
AADN03015064               ---------- ---------- ---------- ---------- ---------- ---------- ---------- ---------- ---------- ---------- ----------
AADN03026634               ---------- ---------- ---------- ---------- ---------- ---------- ---------- ---------- ---------- ---------- ----------
NW_003775878               ---------- ---------- ---------- ---------- ---------- ---------- ---------- ---------- ---------- ---------- ----------
DQ018752                   ---------- ---------- ---------- ---------- ---------- ---------- ---------- ---------- ---------- ---------- ----------
DQ018757                   ---------- ---------- ---------- ---------- ---------- ---------- ---------- ---------- ---------- ---------- ----------
DQ112354                   ---------- ---------- ---------- ---------- ---------- ---------- ---------- ---------- ---------- ---------- ----------
FM165414                   ---------- ---------- ---------- ---------- ---------- ---------- ---------- ---------- ---------- ---------- ----------
FM165415                   ---------- ---------- ---------- ---------- ---------- ---------- ---------- ---------- ---------- ---------- ----------
AADN03000430               ---------- ---------- ---------- ---------- ---------- ---------- ---------- ---------- ---------- ---------- ----------
```

```
AADN03001784              CAGCGCCGAA TCCCCGCCCC GCGGTGGGGC GCGGGAGGTG TGGCGTACGG AAGCCCCCAT CCCCGGCGCC GCTCTCGGGG GGCCCAAGTC CTTCTGATCG AGGCCCAGCC
AADN03001783              ---------- ---------- ---------- ---------- ---------- ---------- ---------- ---------- ---------- ---------- ----------
DQ018754                  ---------- ---------- ---------- ---------- ---------- ---------- ---------- ---------- ---------- ---------- ----------
AADN03001782              ---------- ---------- ---------- ---------- ---------- ---------- ---------- ---------- ---------- ---------- ----------
JN639848                  ---------- ---------- ---------- ---------- ---------- ---------- ---------- ---------- ---------- ---------- ----------
AADN03001670              ---------- ---------- ---------- ---------- ---------- ---------- ---------- ---------- ---------- ---------- ----------
HQ873432                  ---------- ---------- ---------- ---------- ---------- ---------- ---------- ---------- ---------- ---------- ----------
AADN03001774              ---------- ---------- ---------- ---------- ---------- ---------- ---------- ---------- ---------- ---------- ----------
AADN03001775              ---------- ---------- ---------- ---------- ---------- ---------- ---------- ---------- ---------- ---------- ----------
EF552813                  ---------- ---------- ---------- ---------- ---------- ---------- ---------- ---------- ---------- ---------- ----------
AADN03022685              ---------- ---------- ---------- ---------- ---------- ---------- ---------- ---------- ---------- ---------- ----------
AADN03019346              ---------- ---------- ---------- ---------- ---------- ---------- ---------- ---------- ---------- ---------- ----------
AADN03001776              ---------- ---------- ---------- ---------- ---------- ---------- ---------- ---------- ---------- ---------- ----------
DQ018756                  ---------- ---------- -CGGTGGGGC GCGGGAGGTG TGGCGTACGG AAGCCCCCAT CCCCGGCGCC GCTCTCGGGG GGCCCAAGTC CTTCTGATCG AGGCCCAGCC
AF173612                  ---------- ---------- ---------- ---------- ---------- ---------- ---------- ---------- ---------- ---------- ----------
Gallus_gallus_KT445934.2  CAGCGCCGAA TCCCCGCCCC GCGGTGGGGC GCGGGAGGTG TGGCGTACGG AAGCCCCCAT CCCCGGCGCC GCTCTCGGGG GGCCCAAGTC CTTCTGATCG AGGCCCAGCC

                          7777777777 7777777777 7777777777 7777777777 7777777777 7777777777 7777777777 7777777777 7777777777 7777777777 7777777777
                          3333333333 3333333333 3333333334 4444444444 4444444444 4444444444 4444444444 4444444444 4444444444 4444444444 4444444444
                          7777777778 8888888889 9999999990 0000000001 1111111112 2222222223 3333333334 4444444445 5555555556 6666666667 7777777778
                          1234567890 1234567890 1234567890 1234567890 1234567890 1234567890 1234567890 1234567890 1234567890 1234567890 1234567890
AADN03001677              ---------- ---------- ---------- ---------- ---------- ---------- ---------- ---------- ---------- ---------- ----------
AADN03001778              ---------- ---------- ---------- ---------- ---------- ---------- ---------- ---------- ---------- ---------- ----------
AADN03001785              ---------- ---------- ---------- ---------- ---------- ---------- ---------- ---------- ---------- ---------- ----------
AADN03001786              ---------- ---------- ---------- ---------- ---------- ---------- ---------- ---------- ---------- ---------- ----------
AADN03001788              ---------- ---------- ---------- ---------- ---------- ---------- ---------- ---------- ---------- ---------- ----------
AADN03014081              ---------- ---------- ---------- ---------- ---------- ---------- ---------- ---------- ---------- ---------- ----------
AADN03015064              ---------- ---------- ---------- ---------- ---------- ---------- ---------- ---------- ---------- ---------- ----------
AADN03026634              ---------- ---------- ---------- ---------- ---------- ---------- ---------- ---------- ---------- ---------- ----------
NW_003775878              ---------- ---------- ---------- ---------- ---------- ---------- ---------- ---------- ---------- ---------- ----------
DQ018752                  ---------- ---------- ---------- ---------- ---------- ---------- ---------- ---------- ---------- ---------- ----------
DQ018757                  ---------- ---------- ---------- ---------- ---------- ---------- ---------- ---------- ---------- ---------- ----------
DQ112354                  ---------- ---------- ---------- ---------- ---------- ---------- ---------- ---------- ---------- ---------- ----------
FM165414                  ---------- ---------- ---------- ---------- ---------- ---------- ---------- ---------- ---------- ---------- ----------
FM165415                  ---------- ---------- ---------- ---------- ---------- ---------- ---------- ---------- ---------- ---------- ----------
AADN03000430              ---------- ---------- ---------- ---------- ---------- ---------- ---------- ---------- ---------- ---------- ----------
AADN03001784              CGCGGACGGT GTGAGGCCGG TAGCGGCCCC CCGGCGCGCC GGGCCCGGGG CTTCTCGGAG TCGGGTTGCT TGGGAATGCA GCCCAAAGCG GGTGGTAAAC TCCATCTAAG
AADN03001783              ---------- ---------- ---------- ---------- ---------- ---------- ---------- ---------- ---------- ---------- ----------
DQ018754                  ---------- ---------- ---------- ---------- ---------- ---------- ---------- ---------- ---------- ---------- ----------
AADN03001782              ---------- ---------- ---------- ---------- ---------- ---------- ---------- ---------- ---------- ---------- ----------
JN639848                  ---------- ---------- ---------- ---------- ---------- ---------- ---------- ---------- ---------- ---------- ----------
AADN03001670              ---------- ---------- ---------- ---------- ---------- ---------- ---------- ---------- ---------- ---------- ----------
HQ873432                  ---------- ---------- ---------- ---------- ---------- ---------- ---------- ---------- ---------- ---------- ----------
AADN03001774              ---------- ---------- ---------- ---------- ---------- ---------- ---------- ---------- ---------- ---------- ----------
AADN03001775              ---------- ---------- ---------- ---------- ---------- ---------- ---------- ---------- ---------- ---------- ----------
EF552813                  ---------- ---------- ---------- ---------- ---------- ---------- ---------- ---------- ---------- ---------- ----------
AADN03022685              ---------- ---------- ---------- ---------- ---------- ---------- ---------- ---------- ---------- ---------- ----------
AADN03019346              ---------- ---------- ---------- ---------- ---------- ---------- ---------- ---------- ---------- ---------- ----------
AADN03001776              ---------- ---------- ---------- ---------- ---------- ---------- ---------- ---------- ---------- ---------- ----------
DQ018756                  CGCGGACGGT GTGAGGCCGG TAGCGGCCCC CCGGCGCGCC GGGCCCGGGG CTTCTCGGAG TCGGGTTGCT TGGGAATGCA GCCCAAAGCG GGTGGTAAAC TCCATCTAAG
AF173612                  ---------- ---------- ---------- ---------- ---------- ---------- ---------- ---------- ---------- ---------- ----------
Gallus_gallus_KT445934.2  CGCGGACGGT GTGAGGCCGG TAGCGGCCCC CCGGCGCGCC GGGCCCGGGG CTTCTCGGAG TCGGGTTGCT TGGGAATGCA GCCCAAAGCG GGTGGTAAAC TCCATCTAAG
```

```
                            7777777777 7777777777 7777777777 7777777777 7777777777 7777777777 7777777777 7777777777 7777777777 7777777777 7777777777
                            4444444444 4444444445 5555555555 5555555555 5555555555 5555555555 5555555555 5555555555 5555555555 5555555555 5555555555
                            8888888889 9999999990 0000000001 1111111112 2222222223 3333333334 4444444445 5555555556 6666666667 7777777778 8888888889
                            1234567890 1234567890 1234567890 1234567890 1234567890 1234567890 1234567890 1234567890 1234567890 1234567890 1234567890
AADN03001677                ---------- ---------- ---------- ---------- ---------- ---------- ---------- ---------- ---------- ---------- ----------
AADN03001778                ---------- ---------- ---------- ---------- ---------- ---------- ---------- ---------- ---------- ---------- ----------
AADN03001785                ---------- ---------- ---------- ---------- ---------- ---------- ---------- ---------- ---------- ---------- ----------
AADN03001786                ---------- ---------- ---------- ---------- ---------- ---------- ---------- ---------- ---------- ---------- ----------
AADN03001788                ---------- ---------- ---------- ---------- ---------- ---------- ---------- ---------- ---------- ---------- ----------
AADN03014081                ---------- ---------- ---------- ---------- ---------- ---------- ---------- ---------- ---------- ---------- ----------
AADN03015064                ---------- ---------- ---------- ---------- ---------- ---------- ---------- ---------- ---------- ---------- ----------
AADN03026634                ---------- ---------- ---------- ---------- ---------- ---------- ---------- ---------- ---------- ---------- ----------
NW_003775878                ---------- ---------- ---------- ---------- ---------- ---------- ---------- ---------- ---------- ---------- ----------
DQ018752                    ---------- ---------- ---------- ---------- ---------- ---------- ---------- ---------- ---------- ---------- ----------
DQ018757                    ---------- ---------- ---------- ---------- ---------- ---------- ---------- ---------- ---------- ---------- ----------
DQ112354                    ---------- ---------- ---------- ---------- ---------- ---------- ---------- ---------- ---------- ---------- ----------
FM165414                    ---------- ---------- ---------- ---------- ---------- ---------- ---------- ---------- ---------- ---------- ----------
FM165415                    ---------- ---------- ---------- ---------- ---------- ---------- ---------- ---------- ---------- ---------- ----------
AADN03000430                ---------- ---------- ---------- ---------- ---------- ---------- ---------- ---------- ---------- ---------- ----------
AADN03001784                GCTAAATACC GGCACGAGAC CGATAGCCAA CAAGTACCGT AAGGGAAAGT TGAAAAGAAC TTTGAAGAGA GAGTTCAAGA GGGCGTGAAA CCGTTAAGAG GTAAACGGGT
AADN03001783                ---------- ---------- ---------- ---------- ---------- ---------- ---------- ---------- ---------- ---------- ----------
DQ018754                    ---------- ---------- ---------- ---------- ---------- ---------- ---------- ---------- ---------- ---------- ----------
AADN03001782                ---------- ---------- ---------- ---------- ---------- ---------- ---------- ---------- ---------- ---------- ----------
JN639848                    ---------- ---------- ---------- ---------- ---------- ---------- ---------- ---------- ---------- ---------- ----------
AADN03001670                ---------- ---------- ---------- ---------- ---------- ---------- ---------- ---------- ---------- ---------- ----------
HQ873432                    ---------- ---------- ---------- ---------- ---------- ---------- ---------- ---------- ---------- ---------- ----------
AADN03001774                ---------- ---------- ---------- ---------- ---------- ---------- ---------- ---------- ---------- ---------- ----------
AADN03001775                ---------- ---------- ---------- ---------- ---------- ---------- ---------- ---------- ---------- ---------- ----------
EF552813                    ---------- ---------- ---------- ---------- ---------- ---------- ---------- ---------- ---------- ---------- ----------
AADN03022685                ---------- ---------- ---------- ---------- ---------- ---------- ---------- ---------- ---------- ---------- ----------
AADN03019346                ---------- ---------- ---------- ---------- ---------- ---------- ---------- ---------- ---------- ---------- ----------
AADN03001776                ---------- ---------- ---------- ---------- ---------- ---------- ---------- ---------- ---------- ---------- ----------
DQ018756                    GCTAAATACC GGCACGAGAC CGATAGCCAA CAAGTACCGT AAGGGAAAGT TGAAAAGAAC TTTGAAGAGA GAGTTCAAGA GGGCGTGAAA CCGTTAAGAG GTAAACGGGT
AF173612                    ---------- ---------- ---------- ---------- ---------- ---------- ---------- ---------- ---------- ---------- ----------
Gallus_gallus_KT445934.2    GCTAAATACC GGCACGAGAC CGATAGCCAA CAAGTACCGT AAGGGAAAGT TGAAAAGAAC TTTGAAGAGA GAGTTCAAGA GGGCGTGAAA CCGTTAAGAG GTAAACGGGT

                            7777777777 7777777777 7777777777 7777777777 7777777777 7777777777 7777777777 7777777777 7777777777 7777777777 7777777777
                            5555555556 6666666666 6666666666 6666666666 6666666666 6666666666 6666666666 6666666666 6666666666 6666666666 6666666667
                            9999999990 0000000001 1111111112 2222222223 3333333334 4444444445 5555555556 6666666667 7777777778 8888888889 9999999990
                            1234567890 1234567890 1234567890 1234567890 1234567890 1234567890 1234567890 1234567890 1234567890 1234567890 1234567890
AADN03001677                ---------- ---------- ---------- ---------- ---------- ---------- ---------- ---------- ---------- ---------- ----------
AADN03001778                ---------- ---------- ---------- ---------- ---------- ---------- ---------- ---------- ---------- ---------- ----------
AADN03001785                ---------- ---------- ---------- ---------- ---------- ---------- ---------- ---------- ---------- ---------- ----------
AADN03001786                ---------- ---------- ---------- ---------- ---------- ---------- ---------- ---------- ---------- ---------- ----------
AADN03001788                ---------- ---------- ---------- ---------- ---------- ---------- ---------- ---------- ---------- ---------- ----------
AADN03014081                ---------- ---------- ---------- ---------- ---------- ---------- ---------- ---------- ---------- ---------- ----------
AADN03015064                ---------- ---------- ---------- ---------- ---------- ---------- ---------- ---------- ---------- ---------- ----------
AADN03026634                ---------- ---------- ---------- ---------- ---------- ---------- ---------- ---------- ---------- ---------- ----------
NW_003775878                ---------- ---------- ---------- ---------- ---------- ---------- ---------- ---------- ---------- ---------- ----------
DQ018752                    ---------- ---------- ---------- ---------- ---------- ---------- ---------- ---------- ---------- ---------- ----------
DQ018757                    ---------- ---------- ---------- ---------- ---------- ---------- ---------- ---------- ---------- ---------- ----------
DQ112354                    ---------- ---------- ---------- ---------- ---------- ---------- ---------- ---------- ---------- ---------- ----------
FM165414                    ---------- ---------- ---------- ---------- ---------- ---------- ---------- ---------- ---------- ---------- ----------
FM165415                    ---------- ---------- ---------- ---------- ---------- ---------- ---------- ---------- ---------- ---------- ----------
AADN03000430                ---------- ---------- ---------- ---------- ---------- ---------- ---------- ---------- ---------- ---------- ----------
```

```
AADN03001784                GGGGTCCGCG CAGTCGGCCC GGAGGATTCA ACCCGGCGGG CCAAGGTCGG CCGGCGCGGG CGCCGTCGGA TCCCCGCCTC CGCCTCCCCT CCGTCCCTCC CCTTCGCCGG
AADN03001783                ---------- ---------- ---------- ---------- ---------- ---------- ---------- ---------- ---------- ---------- ----------
DQ018754                    ---------- ---------- ---------- ---------- ---------- ---------- ---------- ---------- ---------- ---------- ----------
AADN03001782                ---------- ---------- ---------- ---------- ---------- ---------- ---------- ---------- ---------- ---------- ----------
JN639848                    ---------- ---------- ---------- ---------- ---------- ---------- ---------- ---------- ---------- ---------- ----------
AADN03001670                ---------- ---------- ---------- ---------- ---------- ---------- ---------- ---------- ---------- ---------- ----------
HQ873432                    ---------- ---------- ---------- ---------- ---------- ---------- ---------- ---------- ---------- ---------- ----------
AADN03001774                ---------- ---------- ---------- ---------- ---------- ---------- ---------- ---------- ---------- ---------- ----------
AADN03001775                ---------- ---------- ---------- ---------- ---------- ---------- ---------- ---------- ---------- ---------- ----------
EF552813                    ---------- ---------- ---------- ---------- ---------- ---------- ---------- ---------- ---------- ---------- ----------
AADN03022685                ---------- ---------- ---------- ---------- ---------- ---------- ---------- ---------- ---------- ---------- ----------
AADN03019346                ---------- ---------- ---------- ---------- ---------- ---------- ---------- ---------- ---------- ---------- ----------
AADN03001776                ---------- ---------- ---------- ---------- ---------- ---------- ---------- ---------- ---------- ---------- ----------
DQ018756                    GGGGTCCGCG CGGTCGGCCC GGAGGATTCG ACCCGGCGGG CCAAGGTCGG CCGGCGCGGG CGCCGTCGGA TCCCCGCCTC CGCCTCCCCT CCGTCCCTCC CCTTCGCCGG
AF173612                    ---------- ---------- ---------- ---------- ---------- ---------- ---------- ---------- ---------- ---------- ----------
Gallus_gallus_KT445934.2    GGGGTCCGCG CAGTCGGCCC GGAGGATTCA ACCCGGCGGG CCAAGGTCGG CCGGCGCGGG CGCCGTCGGA TCCCCGCCTC CGCCTCCCCT CCGTCCCTCC CCTTCGCCGG

                            7777777777 7777777777 7777777777 7777777777 7777777777 7777777777 7777777777 7777777777 7777777777 7777777777 7777777777
                            7777777777 7777777777 7777777777 7777777777 7777777777 7777777777 7777777777 7777777777 7777777777 7777777778 8888888888
                            0000000001 1111111112 2222222223 3333333334 4444444445 5555555556 6666666667 7777777778 8888888889 9999999990 0000000001
                            1234567890 1234567890 1234567890 1234567890 1234567890 1234567890 1234567890 1234567890 1234567890 1234567890 1234567890
AADN03001677                ---------- ---------- ---------- ---------- ---------- ---------- ---------- ---------- ---------- ---------- ----------
AADN03001778                ---------- ---------- ---------- ---------- ---------- ---------- ---------- ---------- ---------- ---------- ----------
AADN03001785                ---------- ---------- ---------- ---------- ---------- ---------- ---------- ---------- ---------- ---------- ----------
AADN03001786                ---------- ---------- ---------- ---------- ---------- ---------- ---------- ---------- ---------- ---------- ----------
AADN03001788                ---------- ---------- ---------- ---------- ---------- ---------- ---------- ---------- ---------- ---------- ----------
AADN03014081                ---------- ---------- ---------- ---------- ---------- ---------- ---------- ---------- ---------- ---------- ----------
AADN03015064                ---------- ---------- ---------- ---------- ---------- ---------- ---------- ---------- ---------- ---------- ----------
AADN03026634                ---------- ---------- ---------- ---------- ---------- ---------- ---------- ---------- ---------- ---------- ----------
NW_003775878                ---------- ---------- ---------- ---------- ---------- ---------- ---------- ---------- ---------- ---------- ----------
DQ018752                    ---------- ---------- ---------- ---------- ---------- ---------- ---------- ---------- ---------- ---------- ----------
DQ018757                    ---------- ---------- ---------- ---------- ---------- ---------- ---------- ---------- ---------- ---------- ----------
DQ112354                    ---------- ---------- ---------- ---------- ---------- ---------- ---------- ---------- ---------- ---------- ----------
FM165414                    ---------- ---------- ---------- ---------- ---------- ---------- ---------- ---------- ---------- ---------- ----------
FM165415                    ---------- ---------- ---------- ---------- ---------- ---------- ---------- ---------- ---------- ---------- ----------
AADN03000430                ---------- ---------- ---------- ---------- ---------- ---------- ---------- ---------- ---------- ---------- ----------
AADN03001784                GGCGGGGCGG GCCCAGGGGG GGCGGGCGGG CCGGGGACCG CCGCCCGGCC GGCGTCCGGC CCCCGTCGGG CGCATTTCCT CCGCGGCGGT GCGCCGCGAC CGGCTCCGGG
AADN03001783                ---------- ---------- ---------- ---------- ---------- ---------- ---------- ---------- ---------- ---------- ----------
DQ018754                    ---------- ---------- ---------- ---------- ---------- ---------- ---------- ---------- ---------- ---------- ----------
AADN03001782                ---------- ---------- ---------- ---------- ---------- ---------- ---------- ---------- ---------- ---------- ----------
JN639848                    ---------- ---------- ---------- ---------- ---------- ---------- ---------- ---------- ---------- ---------- ----------
AADN03001670                ---------- ---------- ---------- ---------- ---------- ---------- ---------- ---------- ---------- ---------- ----------
HQ873432                    ---------- ---------- ---------- ---------- ---------- ---------- ---------- ---------- ---------- ---------- ----------
AADN03001774                ---------- ---------- ---------- ---------- ---------- ---------- ---------- ---------- ---------- ---------- ----------
AADN03001775                ---------- ---------- ---------- ---------- ---------- ---------- ---------- ---------- ---------- ---------- ----------
EF552813                    ---------- ---------- ---------- ---------- ---------- ---------- ---------- ---------- ---------- ---------- ----------
AADN03022685                ---------- ---------- ---------- ---------- ---------- ---------- ---------- ---------- ---------- ---------- ----------
AADN03019346                ---------- ---------- ---------- ---------- ---------- ---------- ---------- ---------- ---------- ---------- ----------
AADN03001776                ---------- ---------- ---------- ---------- ---------- ---------- ---------- ---------- ---------- ---------- ----------
DQ018756                    GGCGGGGCGG GCCCAGGGGG GGCGGGCGGG CCGGGGACCG CCGCCCGGCC GGCGTCCGGC CCCCGTCGGG CGCATTTCCT CCGCGGCGGT GCGCCGCGAC CGGCTCCGGG
AF173612                    ---------- ---------- ---------- ---------- ---------- ---------- ---------- ---------- ---------- ---------- ----------
Gallus_gallus_KT445934.2    GGCGGGGCGG GCCCAGGGGG GGCGGGCGGG CCGGGGACCG CCGCCCGGCC GGCGTCCGGC CCCCGTCGGG CGCATTTCCT CCGCGGCGGT GCGCCGCGAC CGGCTCCGGG
```

```
                              7777777777 7777777777 7777777777 7777777777 7777777777 7777777777 7777777777 7777777777 7777777777 7777777777 7777777777
                              8888888888 8888888888 8888888888 8888888888 8888888888 8888888888 8888888888 8888888888 8888888889 9999999999 9999999999
                              1111111112 2222222223 3333333334 4444444445 5555555556 6666666667 7777777778 8888888889 9999999990 0000000001 1111111112
                              1234567890 1234567890 1234567890 1234567890 1234567890 1234567890 1234567890 1234567890 1234567890 1234567890 1234567890
AADN03001677                  ---------- ---------- ---------- ---------- ---------- ---------- ---------- ---------- ---------- ---------- ----------
AADN03001778                  ---------- ---------- ---------- ---------- ---------- ---------- ---------- ---------- ---------- ---------- ----------
AADN03001785                  ---------- ---------- ---------- ---------- ---------- ---------- ---------- ---------- ---------- ---------- ----------
AADN03001786                  ---------- ---------- ---------- ---------- ---------- ---------- ---------- ---------- ---------- ---------- ----------
AADN03001788                  ---------- ---------- ---------- ---------- ---------- ---------- ---------- ---------- ---------- ---------- ----------
AADN03014081                  ---------- ---------- ---------- ---------- ---------- ---------- ---------- ---------- ---------- ---------- ----------
AADN03015064                  ---------- ---------- ---------- ---------- ---------- ---------- ---------- ---------- ---------- ---------- ----------
AADN03026634                  ---------- ---------- ---------- ---------- ---------- ---------- ---------- ---------- ---------- ---------- ----------
NW_003775878                  ---------- ---------- ---------- ---------- ---------- ---------- ---------- ---------- ---------- ---------- ----------
DQ018752                      ---------- ---------- ---------- ---------- ---------- ---------- ---------- ---------- ---------- ---------- ----------
DQ018757                      ---------- ---------- ---------- ---------- ---------- ---------- ---------- ---------- ---------- ---------- ----------
DQ112354                      ---------- ---------- ---------- ---------- ---------- ---------- ---------- ---------- ---------- ---------- ----------
FM165414                      ---------- ---------- ---------- ---------- ---------- ---------- ---------- ---------- ---------- ---------- ----------
FM165415                      ---------- ---------- ---------- ---------- ---------- ---------- ---------- ---------- ---------- ---------- ----------
AADN03000430                  ---------- ---------- ---------- ---------- ---------- ---------- ---------- ---------- ---------- ---------- ----------
AADN03001784                  ACGGCTGGGA AGGGCTGCCG GCGGGCAGGT GGCCCGGCGC CGCGCGAGCG GCCGCCGGGT GTTATAGCCG CCGGGCCCGG ATCGTCGCCG AATCCCGGGG CCGAGGGAGA
AADN03001783                  ---------- ---------- ---------- ---------- ---------- ---------- ---------- ---------- ---------- ---------- ----------
DQ018754                      ---------- ---------- ---------- ---------- ---------- ---------- ---------- ---------- ---------- ---------- ----------
AADN03001782                  ---------- ---------- ---------- ---------- ---------- ---------- ---------- ---------- ---------- ---------- ----------
JN639848                      ---------- ---------- ---------- ---------- ---------- ---------- ---------- ---------- ---------- ---------- ----------
AADN03001670                  ---------- ---------- ---------- ---------- ---------- ---------- ---------- ---------- ---------- ---------- ----------
HQ873432                      ---------- ---------- ---------- ---------- ---------- ---------- ---------- ---------- ---------- ---------- ----------
AADN03001774                  ---------- ---------- ---------- ---------- ---------- ---------- ---------- ---------- ---------- ---------- ----------
AADN03001775                  ---------- ---------- ---------- ---------- ---------- ---------- ---------- ---------- ---------- ---------- ----------
EF552813                      ---------- ---------- ---------- ---------- ---------- ---------- ---------- ---------- ---------- ---------- ----------
AADN03022685                  ---------- ---------- ---------- ---------- ---------- ---------- ---------- ---------- ---------- ---------- ----------
AADN03019346                  ---------- ---------- ---------- ---------- ---------- ---------- ---------- ---------- ---------- ---------- ----------
AADN03001776                  ---------- ---------- ---------- ---------- ---------- ---------- ---------- ---------- ---------- ---------- ----------
DQ018756                      ACGGCTGGGA AGGGCTGCCG GCGGGCAGGT GGCCCGGCGC CGCGCGAGCG GCCGCCGGGT GTTATAGCCG CCGGGCCCGG GTCGTCGCCG AATCCCGGGG CCGAGGGAGA
AF173612                      ---------- ---------- ---------- ---------- ---------- ---------- ---------- ---------- ---------- ---------- ----------
Gallus_gallus_KT445934.2      ACGGCTGGGA AGGGCTGCCG GCGGGCAGGT GGCCCGGCGC CGCGCGAGCG GCCGCCGGGT GTTATAGCCG CCGGGCCCGG ATCGTCGCCG AATCCCGGGG CCGAGGGAGA

                              7777777777 7777777777 7777777777 7777777777 7777777777 7777777777 7777777777 7777777778 8888888888 8888888888 8888888888
                              9999999999 9999999999 9999999999 9999999999 9999999999 9999999999 9999999999 9999999990 0000000000 0000000000 0000000000
                              2222222223 3333333334 4444444445 5555555556 6666666667 7777777778 8888888889 9999999990 0000000001 1111111112 2222222223
                              1234567890 1234567890 1234567890 1234567890 1234567890 1234567890 1234567890 1234567890 1234567890 1234567890 1234567890
AADN03001677                  ---------- ---------- ---------- ---------- ---------- ---------- ---------- ---------- ---------- ---------- ----------
AADN03001778                  ---------- ---------- ---------- ---------- ---------- ---------- ---------- ---------- ---------- ---------- ----------
AADN03001785                  ---------- ---------- ---------- ---------- ---------- ---------- ---------- ---------- ---------- ---------- ----------
AADN03001786                  ---------- ---------- ---------- ---------- ---------- ---------- ---------- ---------- ---------- ---------- ----------
AADN03001788                  ---------- ---------- ---------- ---------- ---------- ---------- ---------- ---------- ---------- ---------- ----------
AADN03014081                  ---------- ---------- ---------- ---------- ---------- ---------- ---------- ---------- ---------- ---------- ----------
AADN03015064                  ---------- ---------- ---------- ---------- ---------- ---------- ---------- ---------- ---------- ---------- ----------
AADN03026634                  ---------- ---------- ---------- ---------- ---------- ---------- ---------- ---------- ---------- ---------- ----------
NW_003775878                  ---------- ---------- ---------- ---------- ---------- ---------- ---------- ---------- ---------- ---------- ----------
DQ018752                      ---------- ---------- ---------- ---------- ---------- ---------- ---------- ---------- ---------- ---------- ----------
DQ018757                      ---------- ---------- ---------- ---------- ---------- ---------- ---------- ---------- ---------- ---------- ----------
DQ112354                      ---------- ---------- ---------- ---------- ---------- ---------- ---------- ---------- ---------- ---------- ----------
FM165414                      ---------- ---------- ---------- ---------- ---------- ---------- ---------- ---------- ---------- ---------- ----------
FM165415                      ---------- ---------- ---------- ---------- ---------- ---------- ---------- ---------- ---------- ---------- ----------
AADN03000430                  ---------- ---------- ---------- ---------- ---------- ---------- ---------- ---------- ---------- ---------- ----------
```

```
AADN03001784                GGACCGCCGC CGCGCCCTCC CCCGGAGGGG GCGGCCCCCC GGAGGGCCCC CCGCGGCCGG ACCGGCGTCG GGCCGGCCGC GCCGCGCGCG CGTCCGCGCC GCCGCCGTAC
AADN03001783                ---------- ---------- ---------- ---------- ---------- ---------- ---------- ---------- ---------- ---------- ----------
DQ018754                    ---------- ---------- ---------- ---------- ---------- ---------- ---------- ---------- ---------- ---------- ----------
AADN03001782                ---------- ---------- ---------- ---------- ---------- ---------- ---------- ---------- ---------- ---------- ----------
JN639848                    ---------- ---------- ---------- ---------- ---------- ---------- ---------- ---------- ---------- ---------- ----------
AADN03001670                ---------- ---------- ---------- ---------- ---------- ---------- ---------- ---------- ---------- ---------- ----------
HQ873432                    ---------- ---------- ---------- ---------- ---------- ---------- ---------- ---------- ---------- ---------- ----------
AADN03001774                ---------- ---------- ---------- ---------- ---------- ---------- ---------- ---------- ---------- ---------- ----------
AADN03001775                ---------- ---------- ---------- ---------- ---------- ---------- ---------- ---------- ---------- ---------- ----------
EF552813                    ---------- ---------- ---------- ---------- ---------- ---------- ---------- ---------- ---------- ---------- ----------
AADN03022685                ---------- ---------- ---------- ---------- ---------- ---------- ---------- ---------- ---------- ---------- ----------
AADN03019346                ---------- ---------- ---------- ---------- ---------- ---------- ---------- ---------- ---------- ---------- ----------
AADN03001776                ---------- ---------- ---------- ---------- ---------- ---------- ---------- ---------- ---------- ---------- ----------
DQ018756                    GGACCGCCGC CGCGCCCTCC CCCGGAGGGG GCGGCCCCCC GGAGGGCCCC CCGCGGCCGG ACCGGCGTCG GGCCGGCCGC GCCGCGCGCG CGTCCGCGCC GCCGCTGTAC
AF173612                    ---------- ---------- ---------- ---------- ---------- ---------- ---------- ---------- ---------- ---------- ----------
Gallus_gallus_KT445934.2    GGACCGCCGC CGCGCCCTCC CCCGGAGGGG GCGGCCCCCC GGAGGGCCCC CCGCGGCCGG ACCGGCGTCG GGCCGGCCGC GCCGCGCGCG CGTCCGCGCC GCCGCCGTAC

                            8888888888 8888888888 8888888888 8888888888 8888888888 8888888888 8888888888 8888888888 8888888888 8888888888 8888888888
                            0000000000 0000000000 0000000000 0000000000 0000000000 0000000000 0000000001 1111111111 1111111111 1111111111 1111111111
                            3333333334 4444444445 5555555556 6666666667 7777777778 8888888889 9999999990 0000000001 1111111112 2222222223 3333333334
                            1234567890 1234567890 1234567890 1234567890 1234567890 1234567890 1234567890 1234567890 1234567890 1234567890 1234567890
AADN03001677                ---------- ---------- ---------- ---------- ---------- ---------- ---------- ---------- ---------- ---------- ----------
AADN03001778                ---------- ---------- ---------- ---------- ---------- ---------- ---------- ---------- ---------- ---------- ----------
AADN03001785                ---------- ---------- ---------- ---------- ---------- ---------- ---------- ---------- ---------- ---------- ----------
AADN03001786                ---------- ---------- ---------- ---------- ---------- ---------- ---------- ---------- ---------- ---------- ----------
AADN03001788                ---------- ---------- ---------- ---------- ---------- ---------- ---------- ---------- ---------- ---------- ----------
AADN03014081                ---------- ---------- ---------- ---------- ---------- ---------- ---------- ---------- ---------- ---------- ----------
AADN03015064                ---------- ---------- ---------- ---------- ---------- ---------- ---------- ---------- ---------- ---------- ----------
AADN03026634                ---------- ---------- ---------- ---------- ---------- ---------- ---------- ---------- ---------- ---------- ----------
NW_003775878                ---------- ---------- ---------- ---------- ---------- ---------- ---------- ---------- ---------- ---------- ----------
DQ018752                    ---------- ---------- ---------- ---------- ---------- ---------- ---------- ---------- ---------- ---------- ----------
DQ018757                    ---------- ---------- ---------- ---------- ---------- ---------- ---------- ---------- ---------- ---------- ----------
DQ112354                    ---------- ---------- ---------- ---------- ---------- ---------- ---------- ---------- ---------- ---------- ----------
FM165414                    ---------- ---------- ---------- ---------- ---------- ---------- ---------- ---------- ---------- ---------- ----------
FM165415                    ---------- ---------- ---------- ---------- ---------- ---------- ---------- ---------- ---------- ---------- ----------
AADN03000430                ---------- ---------- ---------- ---------- ---------- ---------- ---------- ---------- ---------- ---------- ----------
AADN03001784                GCCGCCGCTC GCTCTCTCTC CGTTCCCCGC CCCGGGTCCG TCCCGGGGCG CGGGGGCGGG GGGG------ ---------- ---------- ---------- ----------
AADN03001783                ---------- ---------- ---------- ---------- ---------- ---------- ---------- ---------- ---------- ---------- ----------
DQ018754                    ---------- ---------- ---------- ---------- ---------- ---------- ---------- ---------- ---------- ---------- ----------
AADN03001782                ---------- ---------- ---------- ---------- ---------- ---------- ---------- ---------- ---------- ---------- ----------
JN639848                    ---------- ---------- ---------- ---------- ---------- ---------- ---------- ---------- ---------- ---------- ----------
AADN03001670                ---------- ---------- ---------- ---------- ---------- ---------- ---------- ---------- ---------- ---------- ----------
HQ873432                    ---------- ---------- ---------- ---------- ---------- ---------- ---------- ---------- ---------- ---------- ----------
AADN03001774                ---------- ---------- ---------- ---------- ---------- ---------- ---------- ---------- ---------- ---------- ----------
AADN03001775                ---------- ---------- ---------- ---------- ---------- ---------- ---------- ---------- ---------- ---------- ----------
EF552813                    ---------- ---------- ---------- ---------- ---------- ---------- ---------- ---------- ---------- ---------- ----------
AADN03022685                ---------- ---------- ---------- ---------- ---------- ---------- ---------- ---------- ---------- ---------- ----------
AADN03019346                ---------- ---------- ---------- ---------- ---------- ---------- ---------- ---------- ---------- ---------- ----------
AADN03001776                ---------- ---------- ---------- ---------- ---------- ---------- ---------- ---------- ---------- ---------- ----------
DQ018756                    GCCGCCGCTC GCTCTCTCTC CGTTCCCCGC CCCGGGTCCG TCCCGGGGCG CGGGGGCGGG GGGGGTCGGC GGGTGTCCGG CGCGCGGCTC GGCGCGGCGC CGCGCGTGTG
AF173612                    ---------- ---------- ---------- ---------- ---------- ---------- ---------- ---------- ---------- ---------- ----------
Gallus_gallus_KT445934.2    GCCGCCGCTC GCTCTCTCTC CGTTCCCCGC CCCGGGTCCG TCCCGGGGCG CGGGGGCGGG GGGGGTC--- GGGTGTCCGG CGCGCGGCTC GGCGCGGCGC CGCGCGTGTG
```

```
                            8888888888 8888888888 8888888888 8888888888 8888888888 8888888888 8888888888 8888888888 8888888888 8888888888 8888888888
                            1111111111 1111111111 1111111111 1111111111 1111111111 1111111112 2222222222 2222222222 2222222222 2222222222 2222222222
                            4444444445 5555555556 6666666667 7777777778 8888888889 9999999990 0000000001 1111111112 2222222223 3333333334 4444444445
                            1234567890 1234567890 1234567890 1234567890 1234567890 1234567890 1234567890 1234567890 1234567890 1234567890 1234567890
AADN03001677                ---------- ---------- ---------- ---------- ---------- ---------- ---------- ---------- ---------- ---------- ----------
AADN03001778                ---------- ---------- ---------- ---------- ---------- ---------- ---------- ---------- ---------- ---------- ----------
AADN03001785                ---------- ---------- ---------- ---------- ---------- ---------- ---------- ---------- ---------- ---------- ----------
AADN03001786                ---------- ---------- ---------- ---------- ---------- ---------- ---------- ---------- ---------- ---------- ----------
AADN03001788                ---------- ---------- ---------- ---------- ---------- ---------- ---------- ---------- ---------- ---------- ----------
AADN03014081                ---------- ---------- ---------- ---------- ---------- ---------- ---------- ---------- ---------- ---------- ----------
AADN03015064                ---------- ---------- ---------- ---------- ---------- ---------- ---------- ---------- ---------- ---------- ----------
AADN03026634                ---------- ---------- ---------- ---------- ---------- ---------- ---------- ---------- ---------- ---------- ----------
NW_003775878                ---------- ---------- ---------- ---------- ---------- ---------- ---------- ---------- ---------- ---------- ----------
DQ018752                    ---------- ---------- ---------- ---------- ---------- ---------- ---------- ---------- ---------- ---------- ----------
DQ018757                    ---------- ---------- ---------- ---------- ---------- ---------- ---------- ---------- ---------- ---------- ----------
DQ112354                    ---------- ---------- ---------- ---------- ---------- ---------- ---------- ---------- ---------- ---------- ----------
FM165414                    ---------- ---------- ---------- ---------- ---------- ---------- ---------- ---------- ---------- ---------- ----------
FM165415                    ---------- ---------- ---------- ---------- ---------- ---------- ---------- ---------- ---------- ---------- ----------
AADN03000430                ---------- ---------- ---------- ---------- ---------- ---------- ---------- ---------- ---------- ---------- ----------
AADN03001784                ---------- ---------- ---------- ---------- ---------- ---------- ---------- ---------- ---------- ---------- ----------
AADN03001783                ---------- ---------- ---------- ---------- ---------- ---------- ---------- ---------- ---------- ---------- ----------
DQ018754                    ---------- ---------- ---------- ---------- ---------- ---------- ---------- ---------- ---------- ---------- ----------
AADN03001782                ---------- ---------- ---------- ---------- ---------- ---------- ---------- ---------- ---------- ---------- ----------
JN639848                    ---------- ---------- ---------- ---------- ---------- ---------- ---------- ---------- ---------- ---------- ----------
AADN03001670                ---------- ---------- ---------- ---------- ---------- ---------- ---------- ---------- ---------- ---------- ----------
HQ873432                    ---------- ---------- ---------- ---------- ---------- ---------- ---------- ---------- ---------- ---------- ----------
AADN03001774                ---------- ---------- ---------G CCGCGGGGGG CGCCGGGGGG GAACCTTCCC CCTTCTGTTC GGGCCGCCTC CGTTCCCGCG GGGGCGGCCC GTTCGGGGGA
AADN03001775                ---------- ---------- ---------- ---------- ---------- ---------- ---------- ---------- ---------- ---------- ----------
EF552813                    ---------- ---------- ---------- ---------- ---------- ---------- ---------- ---------- ---------- ---------- ----------
AADN03022685                ---------- ---------- ---------- ---------- ---------- ---------- ---------- ---------- ---------- ---------- ----------
AADN03019346                ---------- ---------- ---------- ---------- ---------- ---------- ---------- ---------- ---------- ---------- ----------
AADN03001776                ---------- ---------- ---------- ---------- ---------- ---------- ---------- ---------- ---------- ---------- ----------
DQ018756                    GCGCGCGCCT CCAGCCCGGC GCGGGCGAGG CCGCGGGGGG CGCCGGGGGG GAACCTTCCC CCTTCTGTTC GGGCCGCCTC CGTTCCCGCG GGGGCGGCCC GTTCGGGGGA
AF173612                    ---------- ---------- ---------- ---------- ---------- ---------- ---------- ---------- ---------- ---------- ----------
Gallus_gallus_KT445934.2    GCGCGCGCCT CCAGCCCGGC GCGGGCGAGG CCGCGGGGGG CGCCGGGGGG GAACCTTCCC CCTTCTGTTC GGGCCGCCTC CGTTCCCGCG GGGGCGGCCC GTTCGGGGGA

                            8888888888 8888888888 8888888888 8888888888 8888888888 8888888888 8888888888 8888888888 8888888888 8888888888 8888888888
                            2222222222 2222222222 2222222222 2222222222 2222222223 3333333333 3333333333 3333333333 3333333333 3333333333 3333333333
                            5555555556 6666666667 7777777778 8888888889 9999999990 0000000001 1111111112 2222222223 3333333334 4444444445 5555555556
                            1234567890 1234567890 1234567890 1234567890 1234567890 1234567890 1234567890 1234567890 1234567890 1234567890 1234567890
AADN03001677                ---------- ---------- ---------- ---------- ---------- ---------- ---------- ---------- ---------- ---------- ----------
AADN03001778                ---------- ---------- ---------- ---------- ---------- ---------- ---------- ---------- ---------- ---------- ----------
AADN03001785                ---------- ---------- ---------- ---------- ---------- ---------- ---------- ---------- ---------- ---------- ----------
AADN03001786                ---------- ---------- ---------- ---------- ---------- ---------- ---------- ---------- ---------- ---------- ----------
AADN03001788                ---------- ---------- ---------- ---------- ---------- ---------- ---------- ---------- ---------- ---------- ----------
AADN03014081                ---------- ---------- ---------- ---------- ---------- ---------- ---------- ---------- ---------- ---------- ----------
AADN03015064                ---------- ---------- ---------- ---------- ---------- ---------- ---------- ---------- ---------- ---------- ----------
AADN03026634                ---------- ---------- ---------- ---------- ---------- ---------- ---------- ---------- ---------- ---------- ----------
NW_003775878                ---------- ---------- ---------- ---------- ---------- ---------- ---------- ---------- ---------- ---------- ----------
DQ018752                    ---------- ---------- ---------- ---------- ---------- ---------- ---------- ---------- ---------- ---------- ----------
DQ018757                    ---------- ---------- ---------- ---------- ---------- ---------- ---------- ---------- ---------- ---------- ----------
DQ112354                    ---------- ---------- ---------- ---------- ---------- ---------- ---------- ---------- ---------- ---------- ----------
FM165414                    ---------- ---------- ---------- ---------- ---------- ---------- ---------- ---------- ---------- ---------- ----------
FM165415                    ---------- ---------- ---------- ---------- ---------- ---------- ---------- ---------- ---------- ---------- ----------
AADN03000430                ---------- ---------- ---------- ---------- ---------- ---------- ---------- ---------- ---------- ---------- ----------
```

```
AADN03001784               ---------- ---------- ---------- ---------- ---------- ---------- ---------- ---------- ---------- ---------- ----------
AADN03001783               ---------- ---------- ---------- ---------- ---------- ---------- ---------- ---------- ---------- ---------- ----------
DQ018754                   ---------- ---------- ---------- ---------- ---------- ---------- ---------- ---------- ---------- ---------- ----------
AADN03001782               ---------- ---------- ---------- ---------- ---------- ---------- ---------- ---------- ---------- ---------- ----------
JN639848                   ---------- ---------- ---------- ---------- ---------- ---------- ---------- ---------- ---------- ---------- ----------
AADN03001670               ---------- ---------- ---------- ---------- ---------- ---------- ---------- ---------- ---------- ---------- ----------
HQ873432                   ---------- ---------- ---------- ---------- ---------- ---------- ---------- ---------- ---------- ---------- ----------
AADN03001774               CGGGCCCGCC GGCCCCCGGC GCCGCTGTCC GACCGGGGCG GACTGCGCTC AGTGCGCCCC GACCGCGCGG CGCCGCCGGG CCGGGCTCGG GCCACGCCAG GGCGCCCGGG
AADN03001775               ---------- ---------- ---------- ---------- ---------- ---------- ---------- ---------- ---------- ---------- ----------
EF552813                   ---------- ---------- ---------- ---------- ---------- ---------- ---------- ---------- ---------- ---------- ----------
AADN03022685               ---------- ---------- ---------- ---------- ---------- ---------- ---------- ---------- ---------- ---------- ----------
AADN03019346               ---------- ---------- ---------- ---------- ---------- ---------- ---------- ---------- ---------- ---------- ----------
AADN03001776               ---------- ---------- ---------- ---------- ---------- ---------- ---------- ---------- ---------- ---------- ----------
DQ018756                   CGGGCCCGCC GGCCCCCGGC GCCGCTGTCC GACCGGGGCG GACTGCGCTC AGTGCGCCCC GACCGCGCGG CGCCGCCGGG CCGGGCTCGG GCCACGCCAG GGCGCCCGGG
AF173612                   ---------- ---------- ---------- ---------- ---------- ---------- ---------- ---------- ---------- ---------- ----------
Gallus_gallus_KT445934.2   CGGGCCCGCC GGCCCCCGGC GCCGCTGTCC GACCGGGGCG GACTGCGCTC AGTGCGCCCC GACCGCGCGG CGCCGCCGGG CCGGGCTCGG GCCACGCCAG GGCGCCCGGG

                           8888888888 8888888888 8888888888 8888888888 8888888888 8888888888 8888888888 8888888888 8888888888 8888888888 8888888888
                           3333333333 3333333333 3333333333 3333333334 4444444444 4444444444 4444444444 4444444444 4444444444 4444444444 4444444444
                           6666666667 7777777778 8888888889 9999999990 0000000001 1111111112 2222222223 3333333334 4444444445 5555555556 6666666667
                           1234567890 1234567890 1234567890 1234567890 1234567890 1234567890 1234567890 1234567890 1234567890 1234567890 1234567890
AADN03001677               ---------- ---------- ---------- ---------- ---------- ---------- ---------- ---------- ---------- ---------- ----------
AADN03001778               ---------- ---------- ---------- ---------- ---------- ---------- ---------- ---------- ---------- ---------- ----------
AADN03001785               ---------- ---------- ---------- ---------- ---------- ---------- ---------- ---------- ---------- ---------- ----------
AADN03001786               ---------- ---------- ---------- ---------- ---------- ---------- ---------- ---------- ---------- ---------- ----------
AADN03001788               ---------- ---------- ---------- ---------- ---------- ---------- ---------- ---------- ---------- ---------- ----------
AADN03014081               ---------- ---------- ---------- ---------- ---------- ---------- ---------- ---------- ---------- ---------- ----------
AADN03015064               ---------- ---------- ---------- ---------- ---------- ---------- ---------- ---------- ---------- ---------- ----------
AADN03026634               ---------- ---------- ---------- ---------- ---------- ---------- ---------- ---------- ---------- ---------- ----------
NW_003775878               ---------- ---------- ---------- ---------- ---------- ---------- ---------- ---------- ---------- ---------- ----------
DQ018752                   ---------- ---------- ---------- ---------- ---------- ---------- ---------- ---------- ---------- ---------- ----------
DQ018757                   ---------- ---------- ---------- ---------- ---------- ---------- ---------- ---------- ---------- ---------- ----------
DQ112354                   ---------- ---------- ---------- ---------- ---------- ---------- ---------- ---------- ---------- ---------- ----------
FM165414                   ---------- ---------- ---------- ---------- ---------- ---------- ---------- ---------- ---------- ---------- ----------
FM165415                   ---------- ---------- ---------- ---------- ---------- ---------- ---------- ---------- ---------- ---------- ----------
AADN03000430               ---------- ---------- ---------- ---------- ---------- ---------- ---------- ---------- ---------- ---------- ----------
AADN03001784               ---------- ---------- ---------- ---------- ---------- ---------- ---------- ---------- ---------- ---------- ----------
AADN03001783               ---------- ---------- ---------- ---------- ---------- ---------- ---------- ---------- ---------- ---------- ----------
DQ018754                   ---------- ---------- ---------- ---------- ---------- ---------- ---------- ---------- ---------- ---------- ----------
AADN03001782               ---------- ---------- ---------- ---------- ---------- ---------- ---------- ---------- ---------- ---------- ----------
JN639848                   ---------- ---------- ---------- ---------- ---------- ---------- ---------- ---------- ---------- ---------- ----------
AADN03001670               ---------- ---------- ---------- ---------- ---------- ---------- ---------- ---------- ---------- ---------- ----------
HQ873432                   ---------- ---------- ---------- ---------- ---------- ---------- ---------- ---------- ---------- ---------- ----------
AADN03001774               GTCCGCGGCG ACGTCGGCTA CCCACCCGAC CCGTCTTGAA ACACGGACCA AGGAGTCTAG CACGCGCGCG AGTCGGCGGC TCGCGCGAAA GCCCGCGGCG CAATGAAGGT
AADN03001775               ---------- ---------- ---------- ---------- ---------- ---------- ---------- ---------- ---------- ---------- ----------
EF552813                   ---------- ---------- ---------- ---------- ---------- ---------- ---------- ---------- ---------- ---------- ----------
AADN03022685               ---------- ---------- ---------- ---------- ---------- ---------- ---------- ---------- ---------- ---------- ----------
AADN03019346               ---------- ---------- ---------- ---------- ---------- ---------- ---------- ---------- ---------- ---------- ----------
AADN03001776               ---------- ---------- ---------- ---------- ---------- ---------- ---------- ---------- ---------- ---------- ----------
DQ018756                   GTCCGCGGCG ACGTCGGCTA CCCACCCGAC CCGTCTTGAA ACACGGACCA AGGAGTCTAG CACGCGCGCG AGTCGGCGGC TCGCGCGAAA GCCCGCGGCG CAATGAAGGT
AF173612                   ---------- ---------- ---------- ---------- ---------- ---------- ---------- ---------- ---------- ---------- ----------
Gallus_gallus_KT445934.2   GTCCGCGGCG ACGTCGGCTA CCCACCCGAC CCGTCTTGAA ACACGGACCA AGGAGTCTAG CACGCGCGCG AGTCGGCGGC TCGCGCGAAA GCCCGCGGCG CAATGAAGGT
```

```
                            8888888888 8888888888 8888888888 8888888888 8888888888 8888888888 8888888888 8888888888 8888888888 8888888888 8888888888
                            4444444444 4444444444 4444444445 5555555555 5555555555 5555555555 5555555555 5555555555 5555555555 5555555555 5555555555
                            7777777778 8888888889 9999999990 0000000001 1111111112 2222222223 3333333334 4444444445 5555555556 6666666667 7777777778
                            1234567890 1234567890 1234567890 1234567890 1234567890 1234567890 1234567890 1234567890 1234567890 1234567890 1234567890
AADN03001677                ---------- ---------- ---------- ---------- ---------- ---------- ---------- ---------- ---------- ---------- ----------
AADN03001778                ---------- ---------- ---------- ---------- ---------- ---------- ---------- ---------- ---------- ---------- ----------
AADN03001785                ---------- ---------- ---------- ---------- ---------- ---------- ---------- ---------- ---------- ---------- ----------
AADN03001786                ---------- ---------- ---------- ---------- ---------- ---------- ---------- ---------- ---------- ---------- ----------
AADN03001788                ---------- ---------- ---------- ---------- ---------- ---------- ---------- ---------- ---------- ---------- ----------
AADN03014081                ---------- ---------- ---------- ---------- ---------- ---------- ---------- ---------- ---------- ---------- ----------
AADN03015064                ---------- ---------- ---------- ---------- ---------- ---------- ---------- ---------- ---------- ---------- ----------
AADN03026634                ---------- ---------- ---------- ---------- ---------- ---------- ---------- ---------- ---------- ---------- ----------
NW_003775878                ---------- ---------- ---------- ---------- ---------- ---------- ---------- ---------- ---------- ---------- ----------
DQ018752                    ---------- ---------- ---------- ---------- ---------- ---------- ---------- ---------- ---------- ---------- ----------
DQ018757                    ---------- ---------- ---------- ---------- ---------- ---------- ---------- ---------- ------TCGC CGGGGAGGTG GAGCATGAGC
DQ112354                    ---------- ---------- ---------- ---------- ---------- ---------- ---------- ---------- ---------- ---------- ----------
FM165414                    ---------- ---------- ---------- ---------- ---------- ---------- ---------- ---------- ---------- ---------- ----------
FM165415                    ---------- ---------- ---------- ---------- ---------- ---------- ---------- ---------- ---------- ---------- ----------
AADN03000430                ---------- ---------- ---------- ---------- ---------- ---------- ---------- ---------- ---------- ---------- ----------
AADN03001784                ---------- ---------- ---------- ---------- ---------- ---------- ---------- ---------- ---------- ---------- ----------
AADN03001783                ---------- ---------- ---------- ---------- ---------- ---------- ---------- ---------- ---------- ---------- ----------
DQ018754                    ---------- ---------- ---------- ---------- ---------- ---------- ---------- ---------- ---------- ---------- ----------
AADN03001782                ---------- ---------- ---------- ---------- ---------- ---------- ---------- ---------- ---------- ---------- ----------
JN639848                    ---------- ---------- ---------- ---------- ---------- ---------- ---------- ---------- ---------- ---------- ----------
AADN03001670                ---------- ---------- ---------- ---------- ---------- ---------- ---------- ---------- ---------- ---------- ----------
HQ873432                    ---------- ---------- ---------- ---------- ---------- ---------- ---------- ---------- ---------- ---------- ----------
AADN03001774                GAGGGCCGGC GCGCGCCGGC TGAGGTGGGA TCCCGGGGCG GCAGGCCGGA AGGCCCCGGG CG-ACCACCG GCCCGTCTCG CCCGCCTCGC CGGGGAGGTG GAGCATGAGC
AADN03001775                ---------- ---------- ---------- ---------- ---------- ---------- ---------- ---------- ---------- ---------- ----------
EF552813                    ---------- ---------- ---------- ---------- ---------- ---------- ---------- ---------- ---------- ---------- ----------
AADN03022685                ---------- ---------- ---------- ---------- ---------- ---------- ---------- ---------- ---------- ---------- ----------
AADN03019346                ---------- ---------- ---------- ---------- ---------- ---------- ---------- ---------- ---------- ---------- ----------
AADN03001776                ---------- ---------- ---------- ---------- ---------- ---------- ---------- ---------- ---------- ---------- ----------
DQ018756                    GAGGGCCGGC GCGCGCCGGC TGAGGTGGGA TCCCGGGGCG GCAGGCCGGA AGGCCCCAGG CG-ACCACCG GCCCGTCTCG CCCGCCTCGC CGGGGAGGTG GAGCATGAGC
AF173612                    ---------- ---------- ---------- ---------- ---------- ---------- ---------- ---------- ---------- ---------- ----------
Gallus_gallus_KT445934.2    GAGGGCCGGC GCGCGCCGGC TGAGGTGGGA TCCCGGGGCG GCAGGCCGGA AGGCCCCGGG CGCACCACCG GCCCGTCTCG CCCGCCTCGC CGGGGAGGTG GAGCATGAGC

                            8888888888 8888888888 8888888888 8888888888 8888888888 8888888888 8888888888 8888888888 8888888888 8888888888 8888888888
                            5555555555 5555555556 6666666666 6666666666 6666666666 6666666666 6666666666 6666666666 6666666666 6666666666 6666666666
                            8888888889 9999999990 0000000001 1111111112 2222222223 3333333334 4444444445 5555555556 6666666667 7777777778 8888888889
                            1234567890 1234567890 1234567890 1234567890 1234567890 1234567890 1234567890 1234567890 1234567890 1234567890 1234567890
AADN03001677                ---------- ---------- ---------- ---------- ---------- ---------- ---------- ---------- ---------- ---------- ----------
AADN03001778                ---------- ---------- ---------- ---------- ---------- ---------- ---------- ---------- ---------- ---------- ----------
AADN03001785                ---------- ---------- ---------- ---------- ---------- ---------- ---------- ---------- ---------- ---------- ----------
AADN03001786                ---------- ---------- ---------- ---------- ---------- ---------- ---------- ---------- ---------- ---------- ----------
AADN03001788                ---------- ---------- ---------- ---------- ---------- ---------- ---------- ---------- ---------- ---------- ----------
AADN03014081                ---------- ---------- ---------- ---------- ---------- ---------- ---------- ---------- ---------- ---------- ----------
AADN03015064                ---------- ---------- ---------- ---------- ---------- ---------- ---------- ---------- ---------- ---------- ----------
AADN03026634                ---------- ---------- ---------- ---------- ---------- ---------- ---------- ---------- ---------- ---------- ----------
NW_003775878                ---------- ---------- ---------- ---------- ---------- ---------- ---------- ---------- ---------- ---------- ----------
DQ018752                    ---------- ---------- ---------- ---------- ---------- ---------- ---------- ---------- ---------- ---------- ----------
DQ018757                    GCGCGTGCTA GGACCCGAAA GATGGTGAAC TATGCCTGGG CAGGGCGAAG CCAGAGGAAA CTCTGGTGGA GGTCCGTAGC GGTCCTGACG TGCAAATCGG TCGTCCGACC
DQ112354                    ---------- ---------- ---------- ---------- ---------- ---------- ---------- ---------- ---------- ---------- ----------
FM165414                    ---------- ---------- ---------- ---------- ---------- ---------- ---------- ---------- ---------- ---------- ----------
FM165415                    ---------- ---CCCGAAA GATGGTGAAC TATGCCTGGG CAGGGCGAAG CCAGAGGAAA CTCTGGTGGA GGTCCGTAGC GGTCCTGACG TGCAAATCGG TCGTCCGACC
AADN03000430                ---------- ---------- ---------- ---------- ---------- ---------- ---------- ---------- ---------- ---------- ----------
```

```
AADN03001784              ---------- ---------- ---------- ---------- ---------- ---------- ---------- ---------- ---------- ---------- ----------
AADN03001783              ---------- ---------- ---------- ---------- ---------- ---------- ---------- ---------- ---------- ---------- ----------
DQ018754                  ---------- ---------- ---------- ---------- ---------- ---------- ---------- ---------- ---------- ---------- ----------
AADN03001782              ---------- ---------- ---------- ---------- ---------- ---------- ---------- ---------- ---------- ---------- ----------
JN639848                  ---------- ---------- ---------- ---------- ---------- ---------- ---------- ---------- ---------- ---------- ----------
AADN03001670              ---------- ---------- ---------- ---------- ---------- ---------- ---------- ---------- ---------- ---------- ----------
HQ873432                  ---------- ---------- ---------- ---------- ---------- ---------- ---------- ---------- ---------- ---------- ----------
AADN03001774              GCGCGTGCTA GGACCCGAAA GATGGTGAAC TATGCCTGGG CAGGGCGAAG CCAGAGGAAA CTCTGGTGGA GGTCCGTAGC GGTCCTGACG TGCAAATCGG TCGTCCGACC
AADN03001775              ---------- ---------- ---------- ---------- ---------- ---------- ---------- ---------- ---------- ---------- ----------
EF552813                  ---------- ---------- ---------- ---------- ---------- ---------- ---------- ---------- ---------- ---------- ----------
AADN03022685              ---------- ---------- ---------- ---------- ---------- ---------- ---------- ---------- ---------- ---------- ----------
AADN03019346              ---------- ---------- ---------- ---------- ---------- ---------- ---------- ---------- ---------- ---------- ----------
AADN03001776              ---------- ---------- ---------- ---------- ---------- ---------- ---------- ---------- ---------- ---------- ----------
DQ018756                  GCGCGTGCTA GGACCCGAAA GATGGTGAAC TATGCCTGGG CAGGGCGAAG CCAGAGGAAA CTCTGGTGGA GGTCCGTAGC GGTCCTGACG TGCAAATCGG TCGTCCGACC
AF173612                  ---------- ---------- ---------- ---------- ---------- ---------- ---------- ---------- ---------- ---------- ----------
Gallus_gallus_KT445934.2  GCGCGTGCTA GGACCCGAAA GATGGTGAAC TATGCCTGGG CAGGGCGAAG CCAGAGGAAA CTCTGGTGGA GGTCCGTAGC GGTCCTGACG TGCAAATCGG TCGTCCGACC

                          8888888888 8888888888 8888888888 8888888888 8888888888 8888888888 8888888888 8888888888 8888888888 8888888888 8888888888
                          6666666667 7777777777 7777777777 7777777777 7777777777 7777777777 7777777777 7777777777 7777777777 7777777777 7777777778
                          9999999990 0000000001 1111111112 2222222223 3333333334 4444444445 5555555556 6666666667 7777777778 8888888889 9999999990
                          1234567890 1234567890 1234567890 1234567890 1234567890 1234567890 1234567890 1234567890 1234567890 1234567890 1234567890
AADN03001677              ---------- ---------- ---------- ---------- ---------- ---------- ---------- ---------- ---------- ---------- ----------
AADN03001778              ---------- ---------- ---------- ---------- ---------- ---------- ---------- ---------- ---------- ---------- ----------
AADN03001785              ---------- ---------- ---------- ---------- ---------- ---------- ---------- ---------- ---------- ---------- ----------
AADN03001786              ---------- ---------- ---------- ---------- ---------- ---------- ---------- ---------- ---------- ---------- ----------
AADN03001788              ---------- ---------- ---------- ---------- ---------- ---------- ---------- ---------- ---------- ---------- ----------
AADN03014081              ---------- ---------- ---------- ---------- ---------- ---------- ---------- ---------- ---------- ---------- ----------
AADN03015064              ---------- ---------- ---------- ---------- ---------- ---------- ---------- ---------- ---------- ---------- ----------
AADN03026634              ---------- ---------- ---------- ---------- ---------- ---------- ---------- ---------- ---------- ---------- ----------
NW_003775878              ---------- ---------- ---------- ---------- ---------- ---------- ---------- ---------- ---------- ---------- ----------
DQ018752                  ---------- ---------- ---------- ---------- ---------- ---------- ---------- ---------- ---------- ---------- ----------
DQ018757                  CGGGTATAGG GGCGAAAGAC TAATCGAACC ATCTAGTAGC TGGTTCCCTC CGAAGTTTCC CTCAGGATAG CTGGCGCTCG GGGCGGCGGT GCAGTTTTAC CCGGTAAAGC
DQ112354                  ---------- ---------- ---------- ---------- ---------- ---------- ---------- ---------- ---------- ---------- ----------
FM165414                  ---------- ---------- ---------- ---------- ---------- ---------- ---------- ---------- ---------- ---------- ----------
FM165415                  CGGGTATAGG GGCGAAAGAC TAATCGAACC ATCTAGTAGC TGGTTCCCTC CGAAGTTTCC CTCAGGATAG CTGGCGCTCG GGGCGGCGGT GCAGTTTTAC CCGGTAAAGC
AADN03000430              ---------- ---------- ---------- ---------- ---------- ---------- ---------- ---------- ---------- ---------- ----------
AADN03001784              ---------- ---------- ---------- ---------- ---------- ---------- ---------- ---------- ---------- ---------- ----------
AADN03001783              ---------- ---------- ---------- ---------- ---------- ---------- ---------- ---------- ---------- ---------- ----------
DQ018754                  ---------- ---------- ---------- ---------- ---------- ---------- ---------- ---------- ---------- ---------- ----------
AADN03001782              ---------- ---------- ---------- ---------- ---------- ---------- ---------- ---------- ---------- ---------- ----------
JN639848                  ---------- ---------- ---------- ---------- ---------- ---------- ---------- ---------- ---------- ---------- ----------
AADN03001670              ---------- ---------- ---------- ---------- ---------- ---------- ---------- ---------- ---------- ---------- ----------
HQ873432                  ---------- ---------- ---------- ---------- ---------- ---------- ---------- ---------- ---------- ---------- ----------
AADN03001774              CGGGTATAGG GGCGAAAGAC TAATCGAACC ATCTAGTAGC TGGTTCCCTC CGAAGTTTCC CTCAGGATAG CTGGCGCTCG GGGCGGCGGT GCAGTTTTAC CCGGTAAAGC
AADN03001775              ---------- ---------- ---------- ---------- ---------- ---------- ---------- ---------- ---------- ---------- ----------
EF552813                  ---------- ---------- ---------- ---------- ---------- ---------- ---------- ---------- ---------- ---------- ----------
AADN03022685              ---------- ---------- ---------- ---------- ---------- ---------- ---------- ---------- ---------- ---------- ----------
AADN03019346              ---------- ---------- ---------- ---------- ---------- ---------- ---------- ---------- ---------- ---------- ----------
AADN03001776              ---------- ---------- ---------- ---------- ---------- ---------- ---------- ---------- ---------- ---------- ----------
DQ018756                  CGGGTATAGG GGCGAAAGAC TAATCGAACC ATCTAGTAGC TGGTTCCCTC CGAAGTTTCC CTCAGGATAG CTGGCGCTCG GGGCGGCGGT GCAGTTTTAC CCGGTAAAGC
AF173612                  ---------- ---------- ---------- ---------- ---------- ---------- ---------- ---------- ---------- ---------- ----------
Gallus_gallus_KT445934.2  CGGGTATAGG GGCGAAAGAC TAATCGAACC ATCTAGTAGC TGGTTCCCTC CGAAGTTTCC CTCAGGATAG CTGGCGCTCG GGGCGGCGGT GCAGTTTTAC CCGGTAAAGC
```

```
                             8888888888 8888888888 8888888888 8888888888 8888888888 8888888888 8888888888 8888888888 8888888888 8888888888 8888888888
                             8888888888 8888888888 8888888888 8888888888 8888888888 8888888888 8888888888 8888888888 8888888888 8888888889 9999999999
                             0000000001 1111111112 2222222223 3333333334 4444444445 5555555556 6666666667 7777777778 8888888889 9999999990 0000000001
                             1234567890 1234567890 1234567890 1234567890 1234567890 1234567890 1234567890 1234567890 1234567890 1234567890 1234567890
AADN03001677                 ---------- ---------- ---------- ---------- ---------- ---------- ---------- ---------- ---------- ---------- ----------
AADN03001778                 ---------- ---------- ---------- ---------- ---------- ---------- ---------- ---------- ---------- ---------- ----------
AADN03001785                 ---------- ---------- ---------- ---------- ---------- ---------- ---------- ---------- ---------- ---------- ----------
AADN03001786                 ---------- ---------- ---------- ---------- ---------- ---------- ---------- ---------- ---------- ---------- ----------
AADN03001788                 ---------- ---------- ---------- ---------- ---------- ---------- ---------- ---------- ---------- ---------- ----------
AADN03014081                 ---------- ---------- ---------- ---------- ---------- ---------- ---------- ---------- ---------- ---------- ----------
AADN03015064                 ---------- ---------- ---------- ---------- ---------- ---------- ---------- ---------- ---------- ---------- ----------
AADN03026634                 ---------- ---------- ---------- ---------- ---------- ---------- ---------- ---------- ---------- ---------- ----------
NW_003775878                 ---------- ---------- ---------- ---------- ---------- ---------- ---------- ---------- ---------- ---------- ----------
DQ018752                     ---------- ---------- ---------- ---------- ---------- ---------- ---------- ---------- ---------- ---------- ----------
DQ018757                     GAATGATTAG AGGTCTTGGG GCCGAAACGA TCTCAACCTA TTCTCAAACT TTCAATGGGT AAGACGCCCG GCTCGCTGGC GTGGAGCCGG GCCGTGGAAT GCGAGCGCTC
DQ112354                     ---------- ---------- ---------- ---------- ---------- ---------- ---------- ---------- ---------- ---------- ----------
FM165414                     ---------- ---------- ---------- ---------- ---------- ---------- ---------- ---------- ---------- ---------- ----------
FM165415                     GAATGATTAG AGGTCTTGGG GCCGAAACGA TCTCAACCTA TTCTCAAACT TTCAATGGGT AAGACGCCCG GCTCGCTGGC GTGGAGCCGG GCCGTGGAAT GCGAGCGCTC
AADN03000430                 ---------- ---------- ---------- ---------- ---------- ---------- ---------- ---------- ---------- ---------- ----------
AADN03001784                 ---------- ---------- ---------- ---------- ---------- ---------- ---------- ---------- ---------- ---------- ----------
AADN03001783                 ---------- ---------- ---------- ---------- ---------- ---------- ---------- ---------- ---------- ---------- ----------
DQ018754                     ---------- ---------- ---------- ---------- ---------- ---------- ---------- ---------- ---------- ---------- ----------
AADN03001782                 ---------- ---------- ---------- ---------- ---------- ---------- ---------- ---------- ---------- ---------- ----------
JN639848                     ---------- ---------- ---------- ---------- ---------- ---------- ---------- ---------- ---------- ---------- ----------
AADN03001670                 ---------- ---------- ---------- ---------- ---------- ---------- ---------- ---------- ---------- ---------- ----------
HQ873432                     ---------- ---------- ---------- ---------- ---------- ---------- ---------- ---------- ---------- ---------- ----------
AADN03001774                 GAATGATTAG AGGTCTTGGG GCCGAAACGA TCTCAACCTA TTCTCAAACT TTCAATGGGT AAGACGCCCG GCTCGCTGGC GTGGAGCCGG GCCGTGGAAT GCGAGCGCTC
AADN03001775                 ---------- ---------- ---------- ---------- ---------- ---------- ---------- ---------- ---------- ---------- ----------
EF552813                     ---------- ---------- ---------- ---------- ---------- ---------- ---------- ---------- ---------- ---------- ----------
AADN03022685                 ---------- ---------- ---------- ---------- ---------- ---------- ---------- ---------- ---------- ---------- ----------
AADN03019346                 ---------- ---------- ---------- ---------- ---------- ---------- ---------- ---------- ---------- ---------- ----------
AADN03001776                 ---------- ---------- ---------- ---------- ---------- ---------- ---------- ---------- ---------- ---------- ----------
DQ018756                     GAATGATTAG AGGTCTTGGG GCCGAAACGA TCTCAACCTA TTCTCAAACT TTCAATGGGT AAGACGCCCG GCTCGCTGG- ---------- ---------- ----------
AF173612                     ---------- ---------- ---------- ---------- ---------- ---------- ---------- ---------- ---------- ---------- ----------
Gallus_gallus_KT445934.2     GAATGATTAG AGGTCTTGGG GCCGAAACGA TCTCAACCTA TTCTCAAACT TTCAATGGGT AAGACGCCCG GCTCGCTGGC GTGGAGCCGG GCCGTGGAAT GCGAGCGCTC

                             8888888888 8888888888 8888888888 8888888888 8888888888 8888888888 8888888888 8888888888 8888888889 9999999999 9999999999
                             9999999999 9999999999 9999999999 9999999999 9999999999 9999999999 9999999999 9999999999 9999999990 0000000000 0000000000
                             1111111112 2222222223 3333333334 4444444445 5555555556 6666666667 7777777778 8888888889 9999999990 0000000001 1111111112
                             1234567890 1234567890 1234567890 1234567890 1234567890 1234567890 1234567890 1234567890 1234567890 1234567890 1234567890
AADN03001677                 ---------- ---------- ---------- ---------- ---------- ---------- ---------- ---------- ---------- ---------- ----------
AADN03001778                 ---------- ---------- ---------- ---------- ---------- ---------- ---------- ---------- ---------- ---------- ----------
AADN03001785                 ---------- ---------- ---------- ---------- ---------- ---------- ---------- ---------- ---------- ---------- ----------
AADN03001786                 ---------- ---------- ---------- ---------- ---------- ---------- ---------- ---------- ---------- ---------- ----------
AADN03001788                 ---------- ---------- ---------- ---------- ---------- ---------- ---------- ---------- ---------- ---------- ----------
AADN03014081                 ---------- ---------- ---------- ---------- ---------- ---------- ---------- ---------- ---------- ---------- ----------
AADN03015064                 ---------- ---------- ---------- ---------- ---------- ---------- ---------- ---------- ---------- ---------- ----------
AADN03026634                 ---------- ---------- ---------- ---------- ---------- ---------- ---------- ---------- ---------- ---------- ----------
NW_003775878                 ---------- ---------- ---------- ---------- ---------- ---------- ---------- ---------- ---------- ---------- ----------
DQ018752                     ---------- ---------- ---------- ---------- ---------- ---------- ---------- ---------- ---------- ---------- ----------
DQ018757                     AGTGGGCCAC TTTTGGTAAG CAGAACTGGC GCTGCGGGAT GAACCGAACG CCGGGTTAAG GCGCCCGATG CCGACGCTCA TCAGAGCCCA GAAAAGGTGT TGGTTGATCT
DQ112354                     ---------- ---------- ---------- ---------- ---------- ---------- ---------- ---------- ---------- ---------- ----------
FM165414                     ---------- ---------- ---------- ---------- ---------- ---------- ---------- ---------- ---------- ---------- ----------
FM165415                     AGTGGGCCAC TTTTGGTAAG CAGAACTGGC GCTGCGGGAT GAACCGAACG CCGGGTTAAG GCGCCCGATG CCGACGCTCA TCAGAGCCCA GAAAAGGTGT TGGTTGATCT
AADN03000430                 ---------- ---------- ---------- ---------- ---------- ---------- ---------- ---------- ---------- ---------- ----------
```

```
AADN03001784              ---------- ---------- ---------- ---------- ---------- ---------- ---------- ---------- ---------- ---------- ----------
AADN03001783              ---------- ---------- ---------- ---------- ---------- ---------- ---------- ---------- ---------- ---------- ----------
DQ018754                  ---------- ---------- ---------- ---------- ---------- ---------- ---------- ---------- ---------- ---------- ----------
AADN03001782              ---------- ---------- ---------- ---------- ---------- ---------- ---------- ---------- ---------- ---------- ----------
JN639848                  ---------- ---------- ---------- ---------- ---------- ---------- ---------- ---------- ---------- ---------- ----------
AADN03001670              ---------- ---------- ---------- ---------- ---------- ---------- ---------- ---------- ---------- ---------- ----------
HQ873432                  ---------- ---------- ---------- ---------- ---------- ---------- ---------- ---------- ---------- ---------- ----------
AADN03001774              AGTGGGCCAC TTTTGGTAAG CAGAACTGGC GCTGCGGGAT GAACCGAACG CCGGGTTAAG GCGCCCGATG CCGACGCTCA TCAGAGCCCA GAAAAGGTGT TGGTTGATCT
AADN03001775              ---------- ---------- ---------- ---------- ---------- ---------- ---------- ---------- ---------- ---------- ----------
EF552813                  ---------- ---------- ---------- ---------- ---------- ---------- ---------- ---------- ---------- ---------- ----------
AADN03022685              ---------- ---------- ---------- ---------- ---------- ---------- ---------- ---------- ---------- ---------- ----------
AADN03019346              ---------- ---------- ---------- ---------- ---------- ---------- ---------- ---------- ---------- ---------- ----------
AADN03001776              ---------- ---------- ---------- ---------- ---------- ---------- ---------- ---------- ---------- ---------- ----------
DQ018756                  ---------- ---------- ---------- ---------- ---------- ---------- ---------- ---------- ---------- ---------- ----------
AF173612                  ---------- ---------- ---------- ---------- ---------- ---------- ---------- ---------- ---------- ---------- ----------
Gallus_gallus_KT445934.2  AGTGGGCCAC TTTTGGTAAG CAGAACTGGC GCTGCGGGAT GAACCGAACG CCGGGTTAAG GCGCCCGATG CCGACGCTCA TCAGAGCCCA GAAAAGGTGT TGGTTGATCT

                          9999999999 9999999999 9999999999 9999999999 9999999999 9999999999 9999999999 9999999999 9999999999 9999999999 9999999999
                          0000000000 0000000000 0000000000 0000000000 0000000000 0000000000 0000000000 0000000001 1111111111 1111111111 1111111111
                          2222222223 3333333334 4444444445 5555555556 6666666667 7777777778 8888888889 9999999990 0000000001 1111111112 2222222223
                          1234567890 1234567890 1234567890 1234567890 1234567890 1234567890 1234567890 1234567890 1234567890 1234567890 1234567890
AADN03001677              ---------- ---------- ---------- ---------- ---------- ---------- ---------- ---------- ---------- ---------- ----------
AADN03001778              ---------- ---------- ---------- ---------- ---------- ---------- ---------- ---------- ---------- ---------- ----------
AADN03001785              ---------- ---------- ---------- ---------- ---------- ---------- ---------- ---------- ---------- ---------- ----------
AADN03001786              ---------- ---------- ---------- ---------- ---------- ---------- ---------- ---------- ---------- ---------- ----------
AADN03001788              ---------- ---------- ---------- ---------- ---------- ---------- ---------- ---------- ---------- ---------- ----------
AADN03014081              ---------- ---------- ---------- ---------- ---------- ---------- ---------- ---------- ---------- ---------- ----------
AADN03015064              ---------- ---------- ---------- ---------- ---------- ---------- ---------- ---------- ---------- ---------- ----------
AADN03026634              ---------- ---------- ---------- ---------- ---------- ---------- ---------- ---------- ---------- ---------- ----------
NW_003775878              ---------- ---------- ---------- ---------- ---------- ---------- ---------- ---------- ---------- ---------- ----------
DQ018752                  ---------- ---------- ---------- ---------- ---------- ---------- ---------- ---------- ---------- ---------- ----------
DQ018757                  AGACAGCAGG ACGGTGGCCA TGGAAGTCGG AGCCCGCTAA GGAGTGTGTA ACAACTCACC TGCCGAATCA ACTAGCCCTG AAAATGGATG GCGCTGGAGC GTCGGGCCCA
DQ112354                  ---------- ---------- ---------- ---------- ---------- ---------- ---------- ---------- ---------- ---------- ----------
FM165414                  ---------- ---------- ---------- ---------- ---------- ---------- ---------- ---------- ---------- ---------- ----------
FM165415                  AGACAGCAGG ACGGTGGCCA TGGAAGTCGG AACCCGCTAA GGAGTGTGTA ACAACTCACC TGCCGAATCA ACTAGCCCTG AAAATGGATG GCGCTGGAGC GTCAGGCCCA
AADN03000430              ---------- ---------- ---------- ---------- ---------- ---------- ---------- ---------- ---------- ---------- ----------
AADN03001784              ---------- ---------- ---------- ---------- ---------- ---------- ---------- ---------- ---------- ---------- ----------
AADN03001783              ---------- ---------- ---------- ---------- ---------- ---------- ---------- ---------- ---------- ---------- ----------
DQ018754                  ---------- ---------- ---------- ---------- ---------- ---------- ---------- ---------- ---------- ---------- ----------
AADN03001782              ---------- ---------- ---------- ---------- ---------- ---------- ---------- ---------- ---------- ---------- ----------
JN639848                  ---------- ---------- ---------- ---------- ---------- ---------- ---------- ---------- ---------- ---------- ----------
AADN03001670              ---------- ---------- ---------- ---------- ---------- ---------- ---------- ---------- ---------- ---------- ----------
HQ873432                  ---------- ---------- ---------- ---------- ---------- ---------- ---------- ---------- ---------- ---------- ----------
AADN03001774              AGACAGCAGG ACGGTGGCCA TGGAAGTCGG AACCCGCTAA GGAGTGTGTA ACAACTCACC TGCCGAATCA ACTAGCCCTG AAAATGGATG GCGCTGGAGC GTCGGGCCCA
AADN03001775              ---------- ---------- ---------- ---------- ---------- ---------- ---------- ---------- ---------- ---------- ----------
EF552813                  ---------- ---------- ---------- ---------- ---------- ---------- ---------- ---------- ---------- ---------- ----------
AADN03022685              ---------- ---------- ---------- ---------- ---------- ---------- ---------- ---------- ---------- ---------- ----------
AADN03019346              ---------- ---------- ---------- ---------- ---------- ---------- ---------- ---------- ---------- ---------- ----------
AADN03001776              ---------- ---------- ---------- ---------- ---------- ---------- ---------- ---------- ---------- ---------- ----------
DQ018756                  ---------- ---------- ---------- ---------- ---------- ---------- ---------- ---------- ---------- ---------- ----------
AF173612                  ---------- ---------- ---------- ---------- ---------- ---------- ---------- ---------- ---------- ---------- ----------
Gallus_gallus_KT445934.2  AGACAGCAGG ACGGTGGCCA TGGAAGTCGG AACCCGCTAA GGAGTGTGTA ACAACTCACC TGCCGAATCA ACTAGCCCTG AAAATGGATG GCGCTGGAGC GTCGGGCCCA
```

```
                             9999999999 9999999999 9999999999 9999999999 9999999999 9999999999 9999999999 9999999999 9999999999 9999999999 9999999999
                             1111111111 1111111111 1111111111 1111111111 1111111111 1111111111 1111111112 2222222222 2222222222 2222222222 2222222222
                             3333333334 4444444445 5555555556 6666666667 7777777778 8888888889 9999999990 0000000001 1111111112 2222222223 3333333334
                             1234567890 1234567890 1234567890 1234567890 1234567890 1234567890 1234567890 1234567890 1234567890 1234567890 1234567890
AADN03001677                 ---------- ---------- ---------- ---------- ---------- ---------- ---------- ---------- ---------- ---------- ----------
AADN03001778                 ---------- ---------- ---------- ---------- ---------- ---------- ---------- ---------- ---------- ---------- ----------
AADN03001785                 ---------- ---------- ---------- ---------- ---------- ---------- ---------- ---------- ---------- ---------- ----------
AADN03001786                 ---------- ---------- ---------- ---------- ---------- ---------- ---------- ---------- ---------- ---------- ----------
AADN03001788                 ---------- ---------- ---------- ---------- ---------- ---------- ---------- ---------- ---------- ---------- ----------
AADN03014081                 ---------- ---------- ---------- ---------- ---------- ---------- ---------- ---------- ---------- ---------- ----------
AADN03015064                 ---------- ---------- ---------- ---------- ---------- ---------- ---------- ---------- ---------- ---------- ----------
AADN03026634                 ---------- ---------- ---------- ---------- ---------- ---------- ---------- ---------- ---------- ---------- ----------
NW_003775878                 ---------- ---------- ---------- ---------- ---------- ---------- ---------- ---------- ---------- ---------- ----------
DQ018752                     ---------- ---------- ---------- ---------- ---------- ---------- ---------- ---------- ---------- ---------- ----------
DQ018757                     TACCCGGCCG TCGCCGGCGG TGCGGAGCCG CGGGGGCTAC GCCGCGACGA GTAGGAGGGC CGCTGCGGTG CGCCTGGAAG CCTGGGGCGC GGGCCCGGGT GGAGCCGCCG
DQ112354                     ---------- ---------- ---------- ---------- ---------- ---------- ---------- ---------- ---------- ---------- ----------
FM165414                     ---------- ---------- ---------- ---------- ---------- ---------- ---------- ---------- ---------- ---------- ----------
FM165415                     TACCCGGCCG TCGCCGGCGG TGCGGAGCCG CGGGGGCTAC GCCGCGACGA GTAGGAGGGC CGCTGCGGTG CGCCTGGAAG CCTGGGGCGC GGGCCCGGGT GGAGCCGCCG
AADN03000430                 ---------- ---------- ---------- ---------- ---------- ---------- ---------- ---------- ---------- ---------- ----------
AADN03001784                 ---------- ---------- ---------- ---------- ---------- ---------- ---------- ---------- ---------- ---------- ----------
AADN03001783                 ---------- ---------- ---------- ---------- ---------- ---------- ---------- ---------- ---------- ---------- ----------
DQ018754                     ---------- ---------- ---------- ---------- ---------- ---------- ---------- ---------- ---------- ---------- ----------
AADN03001782                 ---------- ---------- ---------- ---------- ---------- ---------- ---------- ---------- ---------- ---------- ----------
JN639848                     ---------- ---------- ---------- ---------- ---------- ---------- ---------- ---------- ---------- ---------- ----------
AADN03001670                 ---------- ---------- ---------- ---------- ---------- ---------- ---------- ---------- ---------- ---------- ----------
HQ873432                     ---------- ---------- ---------- ---------- ---------- ---------- ---------- ---------- ---------- ---------- ----------
AADN03001774                 TACCCGGCCG TCGCCGGCGG TGCGGAGCCG CGGGGGCTAC GCCGCGACGA GTAGGAGGGC CGCTGCGGTG CGCCTGGAAG CCTGGGGCGC GGGCCCGGGT GGAGCCGCCG
AADN03001775                 ---------- ---------- ---------- ---------- ---------- ---------- ---------- ---------- ---------- ---------- ----------
EF552813                     ---------- ---------- ---------- ---------- ---------- ---------- ---------- ---------- ---------- ---------- ----------
AADN03022685                 ---------- ---------- ---------- ---------- ---------- ---------- ---------- ---------- ---------- ---------- ----------
AADN03019346                 ---------- ---------- ---------- ---------- ---------- ---------- ---------- ---------- ---------- ---------- ----------
AADN03001776                 ---------- ---------- ---------- ---------- ---------- ---------- ---------- ---------- ---------- ---------- ----------
DQ018756                     ---------- ---------- ---------- ---------- ---------- ---------- ---------- ---------- ---------- ---------- ----------
AF173612                     ---------- ---------- ---------- ---------- ---------- ---------- ---------- ---------- ---------- ---------- ----------
Gallus_gallus_KT445934.2     TACCCGGCCG TCGCCGGCGG TGCGGAGCCG CGGGGGCTAC GCCGCGACGA GTAGGAGGGC CGCTGCGGTG CGCCTGGAAG CCTGGGGCGC GGGCCCGGGT GGAGCCGCCG

                             9999999999 9999999999 9999999999 9999999999 9999999999 9999999999 9999999999 9999999999 9999999999 9999999999 9999999999
                             2222222222 2222222222 2222222222 2222222222 2222222222 2222222223 3333333333 3333333333 3333333333 3333333333 3333333333
                             4444444445 5555555556 6666666667 7777777778 8888888889 9999999990 0000000001 1111111112 2222222223 3333333334 4444444445
                             1234567890 1234567890 1234567890 1234567890 1234567890 1234567890 1234567890 1234567890 1234567890 1234567890 1234567890
AADN03001677                 ---------- ---------- ---------- ---------- ---------- ---------- ---------- ---------- ---------- ---------- ----------
AADN03001778                 ---------- ---------- ---------- ---------- ---------- ---------- ---------- ---------- ---------- ---------- ----------
AADN03001785                 ---------- ---------- ---------- ---------- ---------- ---------- ---------- ---------- ---------- ---------- ----------
AADN03001786                 ---------- ---------- ---------- ---------- ---------- ---------- ---------- ---------- ---------- ---------- ----------
AADN03001788                 ---------- ---------- ---------- ---------- ---------- ---------- ---------- ---------- ---------- ---------- ----------
AADN03014081                 ---------- ---------- ---------- ---------- ---------- ---------- ---------- ---------- ---------- ---------- ----------
AADN03015064                 ---------- ---------- ---------- ---------- ---------- ---------- ---------- ---------- ---------- ---------- ----------
AADN03026634                 ---------- ---------- ---------- ---------- ---------- ---------- ---------- ---------- ---------- ---------- ----------
NW_003775878                 ---------- ---------- ---------- ---------- ---------- ---------- ---------- ---------- ---------- ---------- ----------
DQ018752                     ---------- ---------- ---------- ---------- ---------- ---------- ---------- ---------- ---------- ---------- ----------
DQ018757                     CAGGTGCAGA TCTTGGTGGT AGTAGCAACT ATTCAAACGA GAGCTTTGAA GGCCGAAGTG GAGCAGGGTT CCATGTGAAC AGCAGTTGAA CATGGGTCAG TCGGTCCTAA
DQ112354                     ---------- ---------- ---------- ---------- ---------- ---------- ---------- ---------- ---------- ---------- ----------
FM165414                     ---------- ---------- ---------- ---------- ---------- ---------- ---------- ---------- ---------- ---------- ----------
FM165415                     CAGGTGCAGA TCTTGGTGGT AGTAGCAACT ATTCAAACGA GAGCTTTGAA GGCCGAAGTG GAGCAGGGTT CCATGTGAAC AGCAGTTGAA CATGGGTCAG TTGGTCCTAA
AADN03000430                 ---------- ---------- ---------- ---------- ---------- ---------- ---------- ---------- ---------- ---------- ----------
```

```
AADN03001784                 ---------- ---------- ---------- ---------- ---------- ---------- ---------- ---------- ---------- ---------- ----------
AADN03001783                 ---------- ---------- ---------- ---------- ---------- ---------- ---------- ---------- ---------- ---------- ----------
DQ018754                     ---------- ---------- ---------- ---------- ---------- ---------- ---------- ---------- ---------- ---------- ----------
AADN03001782                 ---------- ---------- ---------- ---------- ---------- ---------- ---------- ---------- ---------- ---------- ----------
JN639848                     -AGGTGCAGA TCTTGGTGGT AGTA-CAACT ATTCAAACGA GAGCTTTGAA GGCCGAAGTG GAGCAGGGTT CCATGTGAAC AGCAGTTGAA CATGGGTCAG TCGGTCCTAA
AADN03001670                 ---------- ---------- ---------- ---------- ---------- ---------- ---------- ---------- ---------- ---------- ----------
HQ873432                     ---------- ---------- ---------- ---------- ---------- ---------- ---------- ---------- ---------- ---------- ----------
AADN03001774                 CAGGTGCAGA TCTTGGTGGT AGTAGCAACT ATTCAAACGA GAGCTTTGAA GGCCGAAGTG GAGCAGGGTT CCATGTGAAC AGCAGTTGAA CATGGGTCAG TCGGTCCTAA
AADN03001775                 ---------- ---------- ---------- ----AAACGA GAGCTTTGAA GGCCGAAGTG GAGCAGGGTT CCATGTGAAC AGCAGTTGAA CATGGGTCAG TCGGTCCTAA
EF552813                     ---------- ---------- ---------- ---------- ---------- ---------- ---------- ---------- ---------- ---------- ----------
AADN03022685                 ---------- ---------- ---------- ---------- ---------- ---------- ---------- ---------- ---------- ---------- ----------
AADN03019346                 ---------- ---------- ---------- ---------- ---------- ---------- ---------- ---------- ---------- ---------- ----------
AADN03001776                 ---------- ---------- ---------- ---------- ---------- ---------- ---------- ---------- ---------- ---------- ----------
DQ018756                     ---------- ---------- ---------- ---------- ---------- ---------- ---------- ---------- ---------- ---------- ----------
AF173612                     ---------- ---------- ---------- ---------- ---------- ---------- ---------- ---------- ---------- ---------- ----------
Gallus_gallus_KT445934.2     CAGGTGCAGA TCTTGGTGGT AGTAGCAACT ATTCAAACGA GAGCTTTGAA GGCCGAAGTG GAGCAGGGTT CCATGTGAAC AGCAGTTGAA CATGGGTCAG TCGGTCCTAA

                             9999999999 9999999999 9999999999 9999999999 9999999999 9999999999 9999999999 9999999999 9999999999 9999999999 9999999999
                             3333333333 3333333333 3333333333 3333333333 3333333334 4444444444 4444444444 4444444444 4444444444 4444444444 4444444444
                             5555555556 6666666667 7777777778 8888888889 9999999990 0000000001 1111111112 2222222223 3333333334 4444444445 5555555556
                             1234567890 1234567890 1234567890 1234567890 1234567890 1234567890 1234567890 1234567890 1234567890 1234567890 1234567890
AADN03001677                 ---------- ---------- ---------- ---------- ---------- ---------- ---------- ---------- ---------- ---------- ----------
AADN03001778                 ---------- ---------- ---------- ---------- ---------- ---------- ---------- ---------- ---------- ---------- ----------
AADN03001785                 ---------- ---------- ---------- ---------- ---------- ---------- ---------- ---------- ---------- ---------- ----------
AADN03001786                 ---------- ---------- ---------- ---------- ---------- ---------- ---------- ---------- ---------- ---------- ----------
AADN03001788                 ---------- ---------- ---------- ---------- ---------- ---------- ---------- ---------- ---------- ---------- ----------
AADN03014081                 ---------- ---------- ---------- ---------- ---------- ---------- ---------- ---------- ---------- ---------- ----------
AADN03015064                 ---------- ---------- ---------- ---------- ---------- ---------- ---------- ---------- ---------- ---------- ----------
AADN03026634                 ---------- ---------- ---------- ---------- ---------- ---------- ---------- ---------- ---------- ---------- ----------
NW_003775878                 ---------- ---------- ---------- ---------- ---------- ---------- ---------- ---------- ---------- ---------- ----------
DQ018752                     ---------- ---------- ---------- ---------- ---------- ---------- ---------- ---------- ---------- ---------- ----------
DQ018757                     GCGATAGGCG AGCGCCGTTC CGAAGG-ACG GGCGATGGCC TCCGTTGCCC TCAGCCGATC GAAAGGGAGT CGGGTTCAGA TCCCCGAATC CGGAGCGGCG GAGACGGGCG
DQ112354                     ---------- ---------- ---------- ---------- ---------- ---------- ---------- ---------- ---------- ---------- ----------
FM165414                     ---------- ---------- ---------- ---------- ---------- ---------- ---------- ---------- ---------- ---------- ----------
FM165415                     GCGATAGGCG AGCGCCGTTC CGAAGGGACG GGCGATAGCC TCCGTTGCCC TCAGCCGATC GAAAGGGAGT CGGGTTCAGA TCCCCGAATC CGGGGCGGCG GAGACGGGCG
AADN03000430                 ---------- ---------- ---------- ---------- ---------- ---------- ---------- ---------- ---------- ---------- ----------
AADN03001784                 ---------- ---------- ---------- ---------- ---------- ---------- ---------- ---------- ---------- ---------- ----------
AADN03001783                 ---------- ---------- ---------- ---------- ---------- ---------- ---------- ---------- ---------- ---------- ----------
DQ018754                     ---------- ---------- ---------- ---------- ---------- ---------- ---------- ---------- ---------- ---------- ----------
AADN03001782                 ---------- ---------- ---------- ---------- ---------- ---------- ---------- ---------- ---------- ---------- ----------
JN639848                     GCGATAGGCG AGCGCCGTTC CGAAGGGACG GGCGATGGCC TCCGTTGCCC TCAGCCGATC GAAAGGGAGT CGGGTTCAGA TCCCCGAATC CGGAGCGGCG GAGACGGGCG
AADN03001670                 ---------- ---------- ---------- ---------- ---------- ---------- ---------- ---------- ---------- ---------- ----------
HQ873432                     ---------- ---------- ---------- ---------- ---------- ---------- ---------- ---------- ---------- ---------- ----------
AADN03001774                 GCGATAGGCG AGCGCCGTTC CGAAGGGACG GGCGATGGCC TCCGTTGCCC TCAGCCGATC GAAAGGGAGT CGGGTTCAGA TCCCCGAATC CGGAGCGGCG GAGACGGGCG
AADN03001775                 GCGATAGGCG AGCGCCGTTC CGAAGGGACG GGCGATGGCC TCCGTTGCCC TCAGCCGATC GAAAGGGAGT CGGGTTCAGA TCCCCGAATC CGGAGCGGCG GAGACGGGCG
EF552813                     ---------- ---------- ---------- ---------- ---------- ---------- ---------- ---------- ---------- ---------- ----------
AADN03022685                 ---------- ---------- ---------- ---------- ---------- ---------- ---------- ---------- ---------- ---------- ----------
AADN03019346                 ---------- ---------- ---------- ---------- ---------- ---------- ---------- ---------- ---------- ---------- ----------
AADN03001776                 ---------- ---------- ---------- ---------- ---------- ---------- ---------- ---------- ---------- ---------- ----------
DQ018756                     ---------- ---------- ---------- ---------- ---------- ---------- ---------- ---------- ---------- ---------- ----------
AF173612                     ---------- ---------- ---------- ---------- ---------- ---------- ---------- ---------- ---------- ---------- ----------
Gallus_gallus_KT445934.2     GCGATAGGCG AGCGCCGTTC CGAAGGGACG GGCGATGGCC TCCGTTGCCC TCAGCCGATC GAAAGGGAGT CGGGTTCAGA TCCCCGAATC CGGAGCGGCG GAGACGGGCG
```

```
                            9999999999 9999999999 9999999999 9999999999 9999999999 9999999999 9999999999 9999999999 9999999999 9999999999 9999999999
                            4444444444 4444444444 4444444444 4444444445 5555555555 5555555555 5555555555 5555555555 5555555555 5555555555 5555555555
                            6666666667 7777777778 8888888889 9999999990 0000000001 1111111112 2222222223 3333333334 4444444445 5555555556 6666666667
                            1234567890 1234567890 1234567890 1234567890 1234567890 1234567890 1234567890 1234567890 1234567890 1234567890 1234567890
AADN03001677                ---------- ---------- ---------- ---------- ---------- ---------- ---------- ---------- ---------- ---------- ----------
AADN03001778                ---------- ---------- ---------- ---------- ---------- ---------- ---------- ---------- ---------- ---------- ----------
AADN03001785                ---------- ---------- ---------- ---------- ---------- ---------- ---------- ---------- ---------- ---------- ----------
AADN03001786                ---------- ---------- ---------- ---------- ---------- ---------- ---------- ---------- ---------- ---------- ----------
AADN03001788                ---------- ---------- ---------- ---------- ---------- ---------- ---------- ---------- ---------- ---------- ----------
AADN03014081                ---------- ---------- ---------- ---------- ---------- ---------- ---------- ---------- ---------- ---------- ----------
AADN03015064                ---------- ---------- ---------- ---------- ---------- ---------- ---------- ---------- ---------- ---------- ----------
AADN03026634                ---------- ---------- ---------- ---------- ---------- ---------- ---------- ---------- ---------- ---------- ----------
NW_003775878                ---------- ---------- ---------- ---------- ---------- ---------- ---------- ---------- ---------- ---------- ----------
DQ018752                    ---------- ---------- ---------- ---------- ---------- ---------- ---------- ---------- ---------- ---------- ----------
DQ018757                    CCGCGAGGCG CCCAGTGCGG TAACGCAAGC GATCCCGGAG AAGCCAGCGG GAGCCCCGGG GAGAGTTCTC TTTTCTTTGT GAAGGGCCGG GCGCCCTGGA ACGGGTTCGC
DQ112354                    ---------- ---------- ---------- ---------- ---------- ---------- ---------- ---------- ---------- ---------- ----------
FM165414                    ---------- ---------- ---------- ---------- ---------- ---------- ---------- ---------- ---------- ---------- ----------
FM165415                    CCGCGAGGCG CCCAGTGCGG TAACGCAAGC GATCCCGGAG AAGCCGGCGG GAGCCCCGGG GAGAGTTCTC TTTTCTTTGT GAAGGGCCGG GCGCCCTGGA ACGGGTTCGC
AADN03000430                ---------- ---------- ---------- ---------- ---------- ---------- ---------- ---------- ---------- ---------- ----------
AADN03001784                ---------- ---------- ---------- ---------- ---------- ---------- ---------- ---------- ---------- ---------- ----------
AADN03001783                ---------- ---------- ---------- ---------- ---------- ---------- ---------- ---------- ---------- ---------- ----------
DQ018754                    ---------- ---------- ---------- ---------- ---------- ---------- ---------- ---------- ---------- ---------- ----------
AADN03001782                ---------- ---------- ---------- ---------- ---------- ---------- ---------- ---------- ---------- ---------- ----------
JN639848                    CCGCGA---- ---------- ---------- ---------- ---------- ---------- ---------- ---------- ---------- ---------- ----------
AADN03001670                ---------- ---------- ---------- ---------- ---------- ---------- ---------- ---------- ---------- ---------- ----------
HQ873432                    ---------- ---------- ---------- ---------- ---------- ---------- ---------- ---------- ---------- ---------- ----------
AADN03001774                CCGCGAGGCG CCCAGTGCGG TAACGCAAGC GATCCCGGAG AAGCCGGCGG GAGCCCCGGG GAGAGTTCTC TTTTCTTTGT GAAGGGCCGG GCGCCCTGGA ACGGGTTCGC
AADN03001775                CCGCGAGGCG CCCAGTGCGG TAACGCAAGC GATCCCGGAG AAGCCGGCGG GAGCCCCGGG GAGAGTTCTC TTTTCTTTGT GAAGGGCCGG GCGCCCTGGA ACGGGTTCGC
EF552813                    ---------- ---------- ---------- ---------- ---------- ---------- ---------- ---------- ---------- ---------- ----------
AADN03022685                ---------- ---------- ---------- ---------- ---------- ---------- ---------- ---------- ---------- ---------- ----------
AADN03019346                ---------- ---------- ---------- ---------- ---------- ---------- ---------- ---------- ---------- ---------- ----------
AADN03001776                ---------- ---------- ---------- ---------- ---------- ---------- ---------- ---------- ---------- ---------- ----------
DQ018756                    ---------- ---------- ---------- ---------- ---------- ---------- ---------- ---------- ---------- ---------- ----------
AF173612                    ---------- ---------- ---------- ---------- ---------- ---------- ---------- ---------- ---------- ---------- ----------
Gallus_gallus_KT445934.2    CCGCGAGGCG CCCAGTGCGG TAACGCAAGC GATCCCGGAG AAGCCGGCGG GAGCCCCGGG GAGAGTTCTC TTTTCTTTGT GAAGGGCCGG GCGCCCTGGA ACGGGTTCGC

                            9999999999 9999999999 9999999999 9999999999 9999999999 9999999999 9999999999 9999999999 9999999999 9999999999 9999999999
                            5555555555 5555555555 5555555556 6666666666 6666666666 6666666666 6666666666 6666666666 6666666666 6666666666 6666666666
                            7777777778 8888888889 9999999990 0000000001 1111111112 2222222223 3333333334 4444444445 5555555556 6666666667 7777777778
                            1234567890 1234567890 1234567890 1234567890 1234567890 1234567890 1234567890 1234567890 1234567890 1234567890 1234567890
AADN03001677                ---------- ---------- ---------- ---------- ---------- ---------- ---------- ---------- ---------- ---------- ----------
AADN03001778                ---------- ---------- ---------- ---------- ---------- ---------- ---------- ---------- ---------- ---------- ----------
AADN03001785                ---------- ---------- ---------- ---------- ---------- ---------- ---------- ---------- ---------- ---------- ----------
AADN03001786                ---------- ---------- ---------- ---------- ---------- ---------- ---------- ---------- ---------- ---------- ----------
AADN03001788                ---------- ---------- ---------- ---------- ---------- ---------- ---------- ---------- ---------- ---------- ----------
AADN03014081                ---------- ---------- ---------- ---------- ---------- ---------- ---------- ---------- ---------- ---------- ----------
AADN03015064                ---------- ---------- ---------- ---------- ---------- ---------- ---------- ---------- ---------- ---------- ----------
AADN03026634                ---------- ---------- ---------- ---------- ---------- ---------- ---------- ---------- ---------- ---------- ----------
NW_003775878                ---------- ---------- ---------- ---------- ---------- ---------- ---------- ---------- ---------- ---------- ----------
DQ018752                    ---------- ---------- ---------- ---------- ---------- ---------- ---------- ---------- ---------- ---------- ----------
DQ018757                    CCCGAGAGAG GGGCCCGCGC CTTGGAAAGC GTCGCGGTTC CGGCGGCGTC CGGTGAGCTC TCGCTGGCCC GTGAAAATCC GGGGGAGAGG GTGTAAATCT CGCGCCGGGC
DQ112354                    ---------- ---------- ---------- ---------- ---------- ---------- ---------- ---------- ---------- ---------- ----------
FM165414                    ---------- ---------- ---------- ---------- ---------- ---------- ---------- ---------- ---------- ---------- ----------
FM165415                    CCCGAGAGAG GGGCCCGCGC CTTGGAAAGC GTCGCGGTTC CGGCGGCGTC CGGTGAGCTC TCGCTGGCCC GTGAAAATCC GGGGGAGAGG GTGTAAATCT CGCGCCGGGC
AADN03000430                ---------- ---------- ---------- ---------- ---------- ---------- ---------- ---------- ---------- ---------- ----------
```

```
AADN03001784              ---------- ---------- ---------- ---------- ---------- ---------- ---------- ---------- ---------- ---------- ----------
AADN03001783              ---------- ---------- ---------- ---------- ---------- ---------- ---------- ---------- ---------- ---------- ----------
DQ018754                  ---------- ---------- ---------- ---------- ---------- ---------- ---------- ---------- ---------- ---------- ----------
AADN03001782              ---------- ---------- ---------- ---------- ---------- ---------- ---------- ---------- ---------- ---------- ----------
JN639848                  ---------- ---------- ---------- ---------- ---------- ---------- ---------- ---------- ---------- ---------- ----------
AADN03001670              ---------- ---------- ---------- ---------- ---------- ---------- ---------- ---------- ---------- ---------- ----------
HQ873432                  ---------- ---------- ---------- ---------- ---------- ---------- ---------- ---------- ---------- ---------- ----------
AADN03001774              CCCGAGAGAG GGGCCCGCGC CTTGGAAAGC GTCGCGGTTC CGGCGGCGTC CGGTGAGCTC TCGCTGGCCC GTGAAAATCC GGGGGAGAGG GTGTAAATCT CGCGCCGGGC
AADN03001775              CCCGAGAGAG GGGCCCGCGC CTTGGAAAGC GTCGCGGTTC CGGCGGCGTC CGGTGAGCTC TCGCTGGCCC GTGAAAATCC GGGGGAGAGG GTGTAAATCT CGCGCCGGGC
EF552813                  ---------- ---------- ---------- ---------- ---------- ---------- ---------- ---------- ---------- ---------- ----------
AADN03022685              ---------- ---------- ---------- ---------- ---------- ---------- ---------- ---------- ---------- ---------- ----------
AADN03019346              ---------- ---------- ---------- ---------- ---------- ---------- ---------- ---------- ---------- ---------- ----------
AADN03001776              ---------- ---------- ---------- ---------- ---------- ---------- ---------- ---------- ---------- ---------- ----------
DQ018756                  ---------- ---------- ---------- ---------- ---------- ---------- ---------- ---------- ---------- ---------- ----------
AF173612                  ---------- ---------- ---------- ---------- ---------- ---------- ---------- ---------- ---------- ---------- ----------
Gallus_gallus_KT445934.2  CCCGAGAGAG GGGCCCGCGC CTTGGAAAGC GTCGCGGTTC CGGCGGCGTC CGGTGAGCTC TCGCTGGCCC GTGAAAATCC GGGGGAGAGG GTGTAAATCT CGCGCCGGGC

                          9999999999 9999999999 9999999999 9999999999 9999999999 9999999999 9999999999 9999999999 9999999999 9999999999 9999999999
                          6666666666 6666666667 7777777777 7777777777 7777777777 7777777777 7777777777 7777777777 7777777777 7777777777 7777777777
                          8888888889 9999999990 0000000001 1111111112 2222222223 3333333334 4444444445 5555555556 6666666667 7777777778 8888888889
                          1234567890 1234567890 1234567890 1234567890 1234567890 1234567890 1234567890 1234567890 1234567890 1234567890 1234567890
AADN03001677              ---------- ---------- ---------- ---------- ---------- ---------- ---------- ---------- ---------- ---------- ----------
AADN03001778              ---------- ---------- ---------- ---------- ---------- ---------- ---------- ---------- ---------- ---------- ----------
AADN03001785              ---------- ---------- ---------- ---------- ---------- ---------- ---------- ---------- ---------- ---------- ----------
AADN03001786              ---------- ---------- ---------- ---------- ---------- ---------- ---------- ---------- ---------- ---------- ----------
AADN03001788              ---------- ---------- ---------- ---------- ---------- ---------- ---------- ---------- ---------- ---------- ----------
AADN03014081              ---------- ---------- ---------- ---------- ---------- ---------- ---------- ---------- ---------- ---------- ----------
AADN03015064              ---------- ---------- ---------- ---------- ---------- ---------- ---------- ---------- ---------- ---------- ----------
AADN03026634              ---------- ---------- ---------- ---------- ---------- ---------- ---------- ---------- ---------- ---------- ----------
NW_003775878              ---------- ---------- ---------- ---------- ---------- ---------- ---------- ---------- ---------- ---------- ----------
DQ018752                  ---------- ---------- ---------- ---------- ---------- ---------- ---------- ---------- ---------- ---------- ----------
DQ018757                  ---------- ---------- ---------- ---------- ---------- ---------- ---------- ---------- ---------- ---------- ----------
DQ112354                  ---------- ---------- ---------- ---------- ---------- ---------- ---------- ---------- ---------- ---------- ----------
FM165414                  ---------- ---------- ---------- ---------- ---------- ---------- ---------- ---------- ---------- ---------- ----------
FM165415                  CGTACCCATA TCCGCAGCAG GTCTCCAAGG T--------- ---------- ---------- ---------- ---------- ---------- ---------- ----------
AADN03000430              ---------- ---------- ---------- ---------- ---------- ---------- ---------- ---------- ---------- ---------- ----------
AADN03001784              ---------- ---------- ---------- ---------- ---------- ---------- ---------- ---------- ---------- ---------- ----------
AADN03001783              ---------- ---------- ---------- ---------- ---------- ---------- ---------- ---------- ---------- ---------- ----------
DQ018754                  ---------- ---------- ---------- ---------- ---------- ---------- ---------- ---------- ---------- ---------- ----------
AADN03001782              ---------- ---------- ---------- ---------- ---------- ---------- ---------- ---------- ---------- ---------- ----------
JN639848                  ---------- ---------- ---------- ---------- ---------- ---------- ---------- ---------- ---------- ---------- ----------
AADN03001670              ---------- ---------- ---------- ---------- ---------- ---------- ---------- ---------- ---------- ---------- ----------
HQ873432                  ---------- ---------- ---------- ---------- ---------- ---------- ---------- ---------- ---------- ---------- ----------
AADN03001774              CGTACCCATA TCCGCAGCAG GTCTCCAAGG TGAACAGCCT CTGGCATGTT GGACCAATGT AGGTAAGGGA AGTCGGCAAG CCGGATCCGT AACTTCGGGA TAAGGATTGG
AADN03001775              CGTACCCATA TCCGCAGCAG GTCTCCAAGG TGAACAGCCT CTGGCATGTT GGACCAATGT AGGTAAGGGA AGTCGGCAAG CCGGATCCGT AACTTCGGGA TAAGGATTGG
EF552813                  ---------- ---------- ---------- ---------- ---------- ---------- ---------- ---------- ---------- ---------- ----------
AADN03022685              ---------- ---------- ---------- ---------- ---------- ---------- ---------- ---------- ---------- ---------- ----------
AADN03019346              ---------- ---------- ---------- ---------- ---------- ---------- ---------- ---------- ---------- ---------- ----------
AADN03001776              ---------- ---------- ---------- --------CT CTGGCATGTT GGACCAATGT AGGTAAGGGA AGTCGGCAAG CCGGATCCGT AACTTCGGGA TAAGGATTGG
DQ018756                  ---------- ---------- ---------- ---------- ---------- ---------- ---------- ---------- ---------- ---------- ----------
AF173612                  ---------- ---------- ---------- ---------- ---------- ---------- ---------- ---------- ---------- ---------- ----------
Gallus_gallus_KT445934.2  CGTACCCATA TCCGCAGCAG GTCTCCAAGG TGAACAGCCT CTGGCATGTT GGACCAATGT AGGTAAGGGA AGTCGGCAAG CCGGATCCGT AACTTCGGGA TAAGGATTGG
```

```
                              9999999999 9999999999 9999999999 9999999999 9999999999 9999999999 9999999999 9999999999 9999999999 9999999999 9999999999
                              7777777778 8888888888 8888888888 8888888888 8888888888 8888888888 8888888888 8888888888 8888888888 8888888888 8888888889
                              9999999990 0000000001 1111111112 2222222223 3333333334 4444444445 5555555556 6666666667 7777777778 8888888889 9999999990
                              1234567890 1234567890 1234567890 1234567890 1234567890 1234567890 1234567890 1234567890 1234567890 1234567890 1234567890
AADN03001677                  ---------- ---------- ---------- ---------- ---------- ---------- ---------- ---------- ---------- ---------- ----------
AADN03001778                  ---------- ---------- ---------- ---------- ---------- ---------- ---------- ---------- ---------- ---------- ----------
AADN03001785                  ---------- ---------- ---------- ---------- ---------- ---------- ---------- ---------- ---------- ---------- ----------
AADN03001786                  ---------- ---------- ---------- ---------- ---------- ---------- ---------- ---------- ---------- ---------- ----------
AADN03001788                  ---------- ---------- ---------- ---------- ---------- ---------- ---------- ---------- ---------- ---------- ----------
AADN03014081                  ---------- ---------- ---------- ---------- ---------- ---------- ---------- ---------- ---------- ---------- ----------
AADN03015064                  ---------- ---------- ---------- ---------- ---------- ---------- ---------- ---------- ---------- ---------- ----------
AADN03026634                  ---------- ---------- ---------- ---------- ---------- ---------- ---------- ---------- ---------- ---------- ----------
NW_003775878                  ---------- ---------- ---------- ---------- ---------- ---------- ---------- ---------- ---------- ---------- ----------
DQ018752                      ---------- ---------- ---------- ---------- ---------- ---------- ---------- ---------- ---------- ---------- ----------
DQ018757                      ---------- ---------- ---------- ---------- ---------- ---------- ---------- ---------- ---------- ---------- ----------
DQ112354                      ---------- ---------- ---------- ---------- ---------- ---------- ---------- ---------- ---------- ---------- ----------
FM165414                      ---------- ---------- ---------- ---------- ---------- ---------- ---------- ---------- ---------- ---------- ----------
FM165415                      ---------- ---------- ---------- ---------- ---------- ---------- ---------- ---------- ---------- ---------- ----------
AADN03000430                  ---------- ---------- ---------- ---------- ---------- ---------- ---------- ---------- ---------- ---------- ----------
AADN03001784                  ---------- ---------- ---------- ---------- ---------- ---------- ---------- ---------- ---------- ---------- ----------
AADN03001783                  ---------- ---------- ---------- ---------- ---------- ---------- ---------- ---------- ---------- ---------- ----------
DQ018754                      ---------- ---------- ---------- ---------- ---------- ---------- ---------- ---------- ---------- ---------- ----------
AADN03001782                  ---------- ---------- ---------- ---------- ---------- ---------- ---------- ---------- ---------- ---------- ----------
JN639848                      ---------- ---------- ---------- ---------- ---------- ---------- ---------- ---------- ---------- ---------- ----------
AADN03001670                  ---------- ---------- ---------- ---------- ---------- ---------- ---------- ---------- ---------- ---------- ----------
HQ873432                      ---------- ---------- ---------- ---------- ---------- ---------- ---------- ---------- ---------- ---------- ----------
AADN03001774                  CTCTAAGGGC TGGGTCGGTC GGGCTGGGGC GCGAAGCGGG GCTGGGCGCG CGCCGCG--- ---------- ---------- ---------- ---------- ----------
AADN03001775                  CTCTAAGGGC TGGGTCGGTC GGGCTGGGGC GCGAAGCGGG GCTGGGCGCG CGCCGCGGCT GGACGAGGCG CCGCCCGCCC CCGCCCCCCC TTTCCCCGCT CCCGCTCGCC
EF552813                      ---------- ---------- ---------- ---------- ---------- ---------- ---------- ---------- ---------- ---------- ----------
AADN03022685                  ---------- ---------- ---------- ---------- ---------- ---------- ---------- ---------- ---------- ---------- ----------
AADN03019346                  ---------- ---------- ---------- ---------- ---------- ---------- ---------- ---------- ---------- ---------- ----------
AADN03001776                  CTCTAAGGGC TGGGTCGGTC GGGCTGGGGC GCGAAGCGGG GCTGGGCGCG CGCCGCGGCT GGACGAGGCG CCGCCCGCCC CCGCCCCCCC TTTCCCCGCT CCCGCTCGCC
DQ018756                      ---------- ---------- ---------- ---------- ---------- ---------- ---------- ---------- ---------- ---------- ----------
AF173612                      ---------- ---------- ---------- ---------- ---------- ---------- ---------- ---------- ---------- ---------- ----------
Gallus_gallus_KT445934.2      CTCTAAGGGC TGGGTCGGTC GGGCTGGGGC GCGAAGCGGG GCTGGGCGCG CGCCGCGGCT GGACGAGGCG CCGCCCGCCC CCGCCCCCCC TTTCCCCGCT CCCGCTCGCC

                                                                                                                                 1 1111111111
                              9999999999 9999999999 9999999999 9999999999 9999999999 9999999999 9999999999 9999999999 9999999999 9999999990 0000000000
                              9999999999 9999999999 9999999999 9999999999 9999999999 9999999999 9999999999 9999999999 9999999999 9999999990 0000000000
                              0000000001 1111111112 2222222223 3333333334 4444444445 5555555556 6666666667 7777777778 8888888889 9999999990 0000000001
                              1234567890 1234567890 1234567890 1234567890 1234567890 1234567890 1234567890 1234567890 1234567890 1234567890 1234567890
AADN03001677                  ---------- ---------- ---------- ---------- ---------- ---------- ---------- ---------- ---------- ---------- ----------
AADN03001778                  ---------- ---------- ---------- ---------- ---------- ---------- ---------- ---------- ---------- ---------- ----------
AADN03001785                  ---------- ---------- ---------- ---------- ---------- ---------- ---------- ---------- ---------- ---------- ----------
AADN03001786                  ---------- ---------- ---------- ---------- ---------- ---------- ---------- ---------- ---------- ---------- ----------
AADN03001788                  ---------- ---------- ---------- ---------- ---------- ---------- ---------- ---------- ---------- ---------- ----------
AADN03014081                  ---------- ---------- ---------- ---------- ---------- ---------- ---------- ---------- ---------- ---------- ----------
AADN03015064                  ---------- ---------- ---------- ---------- ---------- ---------- ---------- ---------- ---------- ---------- ----------
AADN03026634                  ---------- ---------- ---------- ---------- ---------- ---------- ---------- ---------- ---------- ---------- ----------
NW_003775878                  ---------- ---------- ---------- ---------- ---------- ---------- ---------- ---------- ---------- ---------- ----------
DQ018752                      ---------- ---------- ---------- ---------- ---------- ---------- ---------- ---------- ---------- ---------- ----------
DQ018757                      ---------- ---------- ---------- ---------- ---------- ---------- ---------- ---------- ---------- ---------- ----------
DQ112354                      ---------- ---------- ---------- ---------- ---------- ---------- ---------- ---------- ---------- ---------- ----------
FM165414                      ---------- ---------- ---------- ---------- ---------- ---------- ---------- ---------- ---------- ---------- ----------
FM165415                      ---------- ---------- ---------- ---------- ---------- ---------- ---------- ---------- ---------- ---------- ----------
```

```
AADN03000430             ---------- ---------- ---------- ---------- ---------- ---------- ---------- ---------- ---------- ---------- ----------
AADN03001784             ---------- ---------- ---------- ---------- ---------- ---------- ---------- ---------- ---------- ---------- ----------
AADN03001783             ---------- ---------- ---------- ---------- ---------- ---------- ---------- ---------- ---------- ---------- ----------
DQ018754                 ---------- ---------- ---------- ---------- ---------- ---------- ---------- ---------- ---------- ---------- ----------
AADN03001782             ---------- ---------- ---------- ---------- ---------- ---------- ---------- ---------- ---------- ---------- ----------
JN639848                 ---------- ---------- ---------- ---------- ---------- ---------- ---------- ---------- ---------- ---------- ----------
AADN03001670             ---------- ---------- ---------- ---------- ---------- ---------- ---------- ---------- ---------- ---------- ----------
HQ873432                 ---------- ---------- ---------- ---------- ---------- ---------- ---------- ---------- ---------- ---------- ----------
AADN03001774             ---------- ---------- ---------- ---------- ---------- ---------- ---------- ---------- ---------- ---------- ----------
AADN03001775             GGGGCGCCGG GGGGGGGGGT CAGCGGGCGG CGCGGCGGCG GCGACTCTGG ACGCGCGCCG GGCCCTTCCC GTGGATCGCC CCAGCTGCGG CGGGCGCCGC TCGCCCCCCT
EF552813                 ---------- ---------- ---------- ---------- ---------- ---------- ---------- ---------- ---------- ---------- ----------
AADN03022685             ---------- ---------- ---------- ---------- ---------- ---------- ---------- ---------- ---------- ---------- ----------
AADN03019346             ---------- ---------- ---------- ---------- ---------- ---------- ---------- ---------- ---------- ---------- ----------
AADN03001776             GGGGCGCCGG GGGGGGGGGT CAGCGGGCGG CGCGGCGGCG GCGACTCTGG ACGCGCGCCG GGCCCTTCCC GTGGATCGCC CCAGCTGCGG CGGGCGCCGC TCGCCCCCCT
DQ018756                 ---------- ---------- ---------- ---------- ---------- ---------- ---------- ---------- ---------- ---------- ----------
AF173612                 ---------- ---------- ---------- ---------- ---------- ---------- ---------- ---------- ---------- ---------- ----------
Gallus_gallus_KT445934.2 GGGGCGCCGG GGGGGGGGGT CAGCGGGCGG CGCGGCGGCG GCGACTCTGG ACGCGCGCCG GGCCCTTCCC GTGGATCGCC CCAGCTGCGG CGGGCGCCGC TCGCCCCCCT

                         1111111111 1111111111 1111111111 1111111111 1111111111 1111111111 1111111111 1111111111 1111111111 1111111111 1111111111
                         0000000000 0000000000 0000000000 0000000000 0000000000 0000000000 0000000000 0000000000 0000000000 0000000000 0000000000
                         0000000000 0000000000 0000000000 0000000000 0000000000 0000000000 0000000000 0000000000 0000000001 1111111111 1111111111
                         1111111112 2222222223 3333333334 4444444445 5555555556 6666666667 7777777778 8888888889 9999999990 0000000001 1111111112
                         1234567890 1234567890 1234567890 1234567890 1234567890 1234567890 1234567890 1234567890 1234567890 1234567890 1234567890
AADN03001677             ---------- ---------- ---------- ---------- ---------- ---------- ---------- ---------- ---------- ---------- ----------
AADN03001778             ---------- ---------- ---------- ---------- ---------- ---------- ---------- ---------- ---------- ---------- ----------
AADN03001785             ---------- ---------- ---------- ---------- ---------- ---------- ---------- ---------- ---------- ---------- ----------
AADN03001786             ---------- ---------- ---------- ---------- ---------- ---------- ---------- ---------- ---------- ---------- ----------
AADN03001788             ---------- ---------- ---------- ---------- ---------- ---------- ---------- ---------- ---------- ---------- ----------
AADN03014081             ---------- ---------- ---------- ---------- ---------- ---------- ---------- ---------- ---------- ---------- ----------
AADN03015064             ---------- ---------- ---------- ---------- ---------- ---------- ---------- ---------- ---------- ---------- ----------
AADN03026634             ---------- ---------- ---------- ---------- ---------- ---------- ---------- ---------- ---------- ---------- ----------
NW_003775878             ---------- ---------- ---------- ---------- ---------- ---------- ---------- ---------- ---------- ---------- ----------
DQ018752                 ---------- ---------- ---------- ---------- ---------- ---------- ---------- ---------- ---------- ---------- ----------
DQ018757                 ---------- ---------- ---------- ---------- ---------- ---------- ---------- ---------- ---------- ---------- ----------
DQ112354                 ---------- ---------- ---------- ---------- ---------- ---------- ---------- ---------- ---------- ---------- ----------
FM165414                 ---------- ---------- ---------- ---------- ---------- ---------- ---------- ---------- ---------- ---------- ----------
FM165415                 ---------- ---------- ---------- ---------- ---------- ---------- ---------- ---------- ---------- ---------- ----------
AADN03000430             ---------- ---------- ---------- ---------- ---------- ---------- ---------- ---------- ---------- ---------- ----------
AADN03001784             ---------- ---------- ---------- ---------- ---------- ---------- ---------- ---------- ---------- ---------- ----------
AADN03001783             ---------- ---------- ---------- ---------- ---------- ---------- ---------- ---------- ---------- ---------- ----------
DQ018754                 ---------- ---------- ---------- ---------- ---------- ---------- ---------- ---------- ---------- ---------- ----------
AADN03001782             ---------- ---------- ---------- ---------- ---------- ---------- ---------- ---------- ---------- ---------- ----------
JN639848                 ---------- ---------- ---------- ---------- ---------- ---------- ---------- ---------- ---------- ---------- ----------
AADN03001670             ---------- ---------- ---------- ---------- ---------- ---------- ---------- ---------- ---------- ---------- ----------
HQ873432                 ---------- ---------- ---------- ---------- ---------- ---------- ---------- ---------- ---------- ---------- ----------
AADN03001774             ---------- ---------- ---------- ---------- ---------- ---------- ---------- ---------- ---------- ---------- ----------
AADN03001775             CCTTGCCCCT CCGCCCCCCC GCTCCCGGCG CCCCTCCCGT CGGCCGTCGT CCCGGCCGCC CCC------- ---------- ---------- ---------- ----------
EF552813                 ---------- ---------- ---------- ---------- ---------- ---------- ---------- ---------- ---------- ---------- ----------
AADN03022685             ---------- ---------- ---------- ---------- ---------- ---------- ---------- ---------- ---------- ---------- ----------
AADN03019346             ---------- ---------- ---------- ---------- ---------- ---------- ---------- ---------- ---------- ---------- ----------
AADN03001776             CCTTGCCCCT CCGCCCCCCC GCTCCCGGCG CCCCTCCCGT CGGCCGTCGT CCCGGCCGCC CCCCGTCCCG AGCGCCCTCC CCGCGAGGGC GCGAGGGGCG GCGGCGGCGG
DQ018756                 ---------- ---------- ---------- ---------- ---------- ---------- ---------- ---------- ---------- ---------- ----------
AF173612                 ---------- ---------- ---------- ---------- ---------- ---------- ---------- ---------- ---------- ---------- ----------
Gallus_gallus_KT445934.2 CCTTGCCCCT CCGCCCCCCC GCTCCCGGCG CCCCTCCCGT CGGCCGTCGT CCCGGCCGCC CCCCGTCCCG AGCGCCCTCC CCGCGAGGGC GCGAGGGGCG GCGGCGGCGG
```

```
                              1111111111 1111111111 1111111111 1111111111 1111111111 1111111111 1111111111 1111111111 1111111111 1111111111 1111111111
                              0000000000 0000000000 0000000000 0000000000 0000000000 0000000000 0000000000 0000000000 0000000000 0000000000 0000000000
                              1111111111 1111111111 1111111111 1111111111 1111111111 1111111111 1111111111 1111111112 2222222222 2222222222 2222222222
                              2222222223 3333333334 4444444445 5555555556 6666666667 7777777778 8888888889 9999999990 0000000001 1111111112 2222222223
                              1234567890 1234567890 1234567890 1234567890 1234567890 1234567890 1234567890 1234567890 1234567890 1234567890 1234567890
AADN03001677                  ---------- ---------- ---------- ---------- ---------- ---------- ---------- ---------- ---------- ---------- ----------
AADN03001778                  ---------- ---------- ---------- ---------- ---------- ---------- ---------- ---------- ---------- ---------- ----------
AADN03001785                  ---------- ---------- ---------- ---------- ---------- ---------- ---------- ---------- ---------- ---------- ----------
AADN03001786                  ---------- ---------- ---------- ---------- ---------- ---------- ---------- ---------- ---------- ---------- ----------
AADN03001788                  ---------- ---------- ---------- ---------- ---------- ---------- ---------- ---------- ---------- ---------- ----------
AADN03014081                  ---------- ---------- ---------- ---------- ---------- ---------- ---------- ---------- ---------- ---------- ----------
AADN03015064                  ---------- ---------- ---------- ---------- ---------- ---------- ---------- ---------- ---------- ---------- ----------
AADN03026634                  ---------- ---------- ---------- ---------- ---------- ---------- ---------- ---------- ---------- ---------- ----------
NW_003775878                  ---------- ---------- ---------- ---------- ---------- ---------- ---------- ---------- ---------- ---------- ----------
DQ018752                      ---------- ---------- ---------- ---------- ---------- ---------- ---------- ---------- ---------- ---------- ----------
DQ018757                      ---------- ---------- ---------- ---------- ---------- ---------- ---------- ---------- ---------- ---------- ----------
DQ112354                      ---------- ---------- ---------- ---------- ---------- ---------- ---------- ---------- ---------- ---------- ----------
FM165414                      ---------- ---------- ---------- ---------- ---------- ---------- ---------- ---------- ---------- ---------- ----------
FM165415                      ---------- ---------- ---------- ---------- ---------- ---------- ---------- ---------- ---------- ---------- ----------
AADN03000430                  ---------- ---------- ---------- ---------- ---------- ---------- ---------- ---------- ---------- ---------- ----------
AADN03001784                  ---------- ---------- ---------- ---------- ---------- ---------- ---------- ---------- ---------- ---------- ----------
AADN03001783                  ---------- ---------- ---------- ---------- ---------- ---------- ---------- ---------- ---------- ---------- ----------
DQ018754                      ---------- ---------- ---------- ---------- ---------- ---------- ---------- ---------- ---------- ---------- ----------
AADN03001782                  ---------- ---------- ---------- ---------- ---------- ---------- ---------- ---------- ---------- ---------- ----------
JN639848                      ---------- ---------- ---------- ---------- ---------- ---------- ---------- ---------- ---------- ---------- ----------
AADN03001670                  ---------- ---------- ---------- ---------- ---------- ---------- ---------- ---------- ---------- ---------- ----------
HQ873432                      ---------- ---------- ---------- ---------- ---------- ---------- ---------- ---------- ---------- ---------- ----------
AADN03001774                  ---------- ---------- ---------- ---------- ---------- ---------- ---------- ---------- ---------- ---------- ----------
AADN03001775                  ---------- ---------- ---------- ---------- ---------- ---------- ---------- ---------- ---------- ---------- ----------
EF552813                      ---------- ---------- ---------- ---------- ---------- ---------- ---------- ---------- ---------- ---------- ----------
AADN03022685                  ---------- ---------- ---------- ---------- ---------- ---------- ---------- ---------- ---------- ---------- ----------
AADN03019346                  ---------- ---------- ---------- ---------- ---------- ---------- ---------- ---------- ---------- ---------- --CTAGCAGC
AADN03001776                  CCGCGGGCGC GGCGGCGGCG GGGGGGGGCC CGCCGGCGGC GCCGGGCGGG GCGGTCCCGG GCGGGGGGGG TCTCCGGGCC GGCGCCCCGC CTCGGCCGGC GCCTAGCAGC
DQ018756                      ---------- ---------- ---------- ---------- ---------- ---------- ---------- ---------- ---------- ---------- ----------
AF173612                      ---------- ---------- ---------- ---------- ---------- ---------- ---------- ---------- ---------- ---------- ----------
Gallus_gallus_KT445934.2      CCGCGGGCGC GGCGGCGGCG GGGGGGGGCC CGCCGGCGGC GCCGGGCGGG GCGGTCCCGG GCGGGGGGGG TCTCCGGGCC GGCGCCCCGC CTCGGCCGGC GCCTAGCAGC

                              1111111111 1111111111 1111111111 1111111111 1111111111 1111111111 1111111111 1111111111 1111111111 1111111111 1111111111
                              0000000000 0000000000 0000000000 0000000000 0000000000 0000000000 0000000000 0000000000 0000000000 0000000000 0000000000
                              2222222222 2222222222 2222222222 2222222222 2222222222 2222222222 2222222223 3333333333 3333333333 3333333333 3333333333
                              3333333334 4444444445 5555555556 6666666667 7777777778 8888888889 9999999990 0000000001 1111111112 2222222223 3333333334
                              1234567890 1234567890 1234567890 1234567890 1234567890 1234567890 1234567890 1234567890 1234567890 1234567890 1234567890
AADN03001677                  ---------- ---------- ---------- ---------- ---------- ---------- ---------- ---------- ---------- ---------- ----------
AADN03001778                  ---------- ---------- ---------- ---------- ---------- ---------- ---------- ---------- ---------- ---------- ----------
AADN03001785                  ---------- ---------- ---------- ---------- ---------- ---------- ---------- ---------- ---------- ---------- ----------
AADN03001786                  ---------- ---------- ---------- ---------- ---------- ---------- ---------- ---------- ---------- ---------- ----------
AADN03001788                  ---------- ---------- ---------- ---------- ---------- ---------- ---------- ---------- ---------- ---------- ----------
AADN03014081                  ---------- ---------- ---------- ---------- ---------- ---------- ---------- ---------- ---------- ---------- ----------
AADN03015064                  ---------- ---------- ---------- ---------- ---------- ---------- ---------- ---------- ---------- ---------- ----------
AADN03026634                  ---------- ---------- ---------- ---------- ---------- ---------- ---------- ---------- ---------- ---------- ----------
NW_003775878                  ---------- ---------- ---------- ---------- ---------- ---------- ---------- ---------- ---------- ---------- ----------
DQ018752                      ---------- ---------- ---------- ---------- ---------- ---------- ---------- ---------- ---------- ---------- ----------
DQ018757                      ---------- ---------- ---------- ---------- ---------- ---------- ---------- ---------- ---------- ---------- ----------
DQ112354                      ---------- ---------- ---------- ---------- ---------- ---------- ---------- ---------- ---------- ---------- ----------
FM165414                      ---------- ---------- ---------- ---------- ---------- ---------- ---------- ---------- ---------- ---------- ----------
```

```
FM165415                     ---------- ---------- ---------- ---------- ---------- ---------- ---------- ---------- ---------- ---------- ----------
AADN03000430                 ---------- ---------- ---------- ---------- ---------- ---------- ---------- ---------- ---------- ---------- ----------
AADN03001784                 ---------- ---------- ---------- ---------- ---------- ---------- ---------- ---------- ---------- ---------- ----------
AADN03001783                 ---------- ---------- ---------- ---------- ---------- ---------- ---------- ---------- ---------- ---------- ----------
DQ018754                     ---------- ---------- ---------- ---------- ---------- ---------- ---------- ---------- ---------- ---------- ----------
AADN03001782                 ---------- ---------- ---------- ---------- ---------- ---------- ---------- ---------- ---------- ---------- ----------
JN639848                     ---------- ---------- ---------- ---------- ---------- ---------- ---------- ---------- ---------- ---------- ----------
AADN03001670                 ---------- ---------- ---------- ---------- ---------- ---------- ---------- ---------- ---------- ---------- ----------
HQ873432                     ---------- ---------- ---------- ---------- ---------- ---------- ---------- ---------- ---------- ---------- ----------
AADN03001774                 ---------- ---------- ---------- ---------- ---------- ---------- ---------- ---------- ---------- ---------- ----------
AADN03001775                 ---------- ---------- ---------- ---------- ---------- ---------- ---------- ---------- ---------- ---------- ----------
EF552813                     ---------- ---------- ---------- ---------- ---------- ---------- ---------- ---------- ---------- ---------- ----------
AADN03022685                 ---------- ---------- ---------- ----CTGTTT AATTAAAACA AAGCATCGCG ATGGCCGCGA ACGGGTGTTG ACACGATGTG ATTTCTGCCC AGTGCTCTGA
AADN03019346                 CGGCTTAGAA CTGGTGCGGA CCAGGGGAAT CCGACTGTTT AATTAAAACA AAGCATCGCG ATGGCCGCGA ACGGGTGTTG ACACGATGTG ATTTCTGCCC AGTGCTCTGA
AADN03001776                 CGGCTTAGAA CTGGTGCGGA CCAGGGGAAT CCGACTGTTT AATTAAAACA AAGCATCGCG AAGGCCCGCG GCGGGTGTTG ACGCGATGTG ATTTCTGCCC AGTGCTCTGA
DQ018756                     ---------- ---------- ---------- ---------- ---------- ---------- ---------- ---------- ---------- ---------- ----------
AF173612                     ---------- ---------- ---------- ---------- ---------- ---------- ---------- ---------- ---------- ---------- ----------
Gallus_gallus_KT445934.2     CGGCTTAGAA CTGGTGCGGA CCAGGGGAAT CCGACTGTTT AATTAAAACA AAGCATCGCG AAGGCCCGCG GCGGGTGTTG ACGCGATGTG ATTTCTGCCC AGTGCTCTGA

                             1111111111 1111111111 1111111111 1111111111 1111111111 1111111111 1111111111 1111111111 1111111111 1111111111 1111111111
                             0000000000 0000000000 0000000000 0000000000 0000000000 0000000000 0000000000 0000000000 0000000000 0000000000 0000000000
                             3333333333 3333333333 3333333333 3333333333 3333333333 3333333334 4444444444 4444444444 4444444444 4444444444 4444444444
                             4444444445 5555555556 6666666667 7777777778 8888888889 9999999990 0000000001 1111111112 2222222223 3333333334 4444444445
                             1234567890 1234567890 1234567890 1234567890 1234567890 1234567890 1234567890 1234567890 1234567890 1234567890 1234567890
AADN03001677                 ---------- ---------- ---------- ---------- ---------- ---------- ---------- ---------- ---------- ---------- ----------
AADN03001778                 ---------- ---------- ---------- ---------- ---------- ---------- ---------- ---------- ---------- ---------- ----------
AADN03001785                 ---------- ---------- ---------- ---------- ---------- ---------- ---------- ---------- ---------- ---------- ----------
AADN03001786                 ---------- ---------- ---------- ---------- ---------- ---------- ---------- ---------- ---------- ---------- ----------
AADN03001788                 ---------- ---------- ---------- ---------- ---------- ---------- ---------- ---------- ---------- ---------- ----------
AADN03014081                 ---------- ---------- ---------- ---------- ---------- ---------- ---------- ---------- ---------- ---------- ----------
AADN03015064                 ---------- ---------- ---------- ---------- ---------- ---------- ---------- ---------- ---------- ---------- ----------
AADN03026634                 ---------- ---------- ---------- ---------- ---------- ---------- ---------- ---------- ---------- ---------- ----------
NW_003775878                 ---------- ---------- ---------- ---------- ---------- ---------- ---------- ---------- ---------- ---------- ----------
DQ018752                     ---------- ---------- ---------- ---------- ---------- ---------- ---------- ---------- ---------- ---------- ----------
DQ018757                     ---------- ---------- ---------- ---------- ---------- ---------- ---------- ---------- ---------- ---------- ----------
DQ112354                     ---------- ---------- ---------- ---------- ---------- ---------- ---------- ---------- ---------- ---------- ----------
FM165414                     ---------- ---------- ---------- ---------- ---------- ---------- ---------- ---------- ---------- ---------- ----------
FM165415                     ---------- ---------- ---------- ---------- ---------- ---------- ---------- ---------- ---------- ---------- ----------
AADN03000430                 ---------- ---------- ---------- ---------- ---------- ---------- ---------- ---------- ---------- ---------- ----------
AADN03001784                 ---------- ---------- ---------- ---------- ---------- ---------- ---------- ---------- ---------- ---------- ----------
AADN03001783                 ---------- ---------- ---------- ---------- ---------- ---------- ---------- ---------- ---------- ---------- ----------
DQ018754                     ---------- ---------- ---------- ---------- ---------- ---------- ---------- ---------- ---------- ---------- ----------
AADN03001782                 ---------- ---------- ---------- ---------- ---------- ---------- ---------- ---------- ---------- ---------- ----------
JN639848                     ---------- ---------- ---------- ---------- ---------- ---------- ---------- ---------- ---------- ---------- ----------
AADN03001670                 ---------- ---------- ---------- ---------- ---------- ---------- ---------- ---------- ---------- ---------- ----------
HQ873432                     ---------- ---------- ---------- ---------- ---------- ---------- ---------- ---------- ---------- ---------- ----------
AADN03001774                 ---------- ---------- ---------- ---------- ---------- ---------- ---------- ---------- ---------- ---------- ----------
AADN03001775                 ---------- ---------- ---------- ---------- ---------- ---------- ---------- ---------- ---------- ---------- ----------
EF552813                     ---------- ---------- ---------- ---------- ---------- ---------- ---------- ---------- ------TAAT TAGTGACGCG CATGAATGGA
AADN03022685                 ATGTCAAAGT GAAGAAATTC AATGAAGCGC GGGTAAACGG CGGGAGTAAC TATGACTCTC TTAAGGTAGC CAAATGCCTC GTCATCTAAT TAGTGACGCG CATGAATGGA
AADN03019346                 ATGTCAAAGT GAAGAAATTC AATGAAGCGC GGGTAAACGG CGGGAGTAAC TATGACTCTC TTAAG----- ---------- ---------- ---------- ----------
AADN03001776                 ATGTCAAAGT GAAGAAATT- ---------- ---------- ---------- ---------- ---------- ---------- ---------- ---------- ----------
DQ018756                     ---------- ---------- ---------- ---------- ---------- ---------- ---------- ---------- ---------- ---------- ----------
AF173612                     ---------- ---------- ---------- ---------- ---------- ---------- ---------- ---------- ---------- ---------- ----------
Gallus_gallus_KT445934.2     ATGTCAAAGT GAAGAAATTC AATGAAGCGC GGGTAAACGG CGGGAGTAAC TATGACTCTC TTAAGGTAGC CAAATGCCTC GTCATCTAAT TAGTGACGCG CATGAATGGA
```

```
                              1111111111 1111111111 1111111111 1111111111 1111111111 1111111111 1111111111 1111111111 1111111111 1111111111 1111111111
                              0000000000 0000000000 0000000000 0000000000 0000000000 0000000000 0000000000 0000000000 0000000000 0000000000 0000000000
                              4444444444 4444444444 4444444444 4444444444 4444444445 5555555555 5555555555 5555555555 5555555555 5555555555 5555555555
                              5555555556 6666666667 7777777778 8888888889 9999999990 0000000001 1111111112 2222222223 3333333334 4444444445 5555555556
                              1234567890 1234567890 1234567890 1234567890 1234567890 1234567890 1234567890 1234567890 1234567890 1234567890 1234567890
AADN03001677                  ---------- ---------- ---------- ---------- ---------- ---------- ---------- ---------- ---------- ---------- ----------
AADN03001778                  ---------- ---------- ---------- ---------- ---------- ---------- ---------- ---------- ---------- ---------- ----------
AADN03001785                  ---------- ---------- ---------- ---------- ---------- ---------- ---------- ---------- ---------- ---------- ----------
AADN03001786                  ---------- ---------- ---------- ---------- ---------- ---------- ---------- ---------- ---------- ---------- ----------
AADN03001788                  ---------- ---------- ---------- ---------- ---------- ---------- ---------- ---------- ---------- ---------- ----------
AADN03014081                  ---------- ---------- ---------- ---------- ---------- ---------- ---------- ---------- ---------- ---------- ----------
AADN03015064                  ---------- ---------- ---------- ---------- ---------- ---------- ---------- ---------- ---------- ---------- ----------
AADN03026634                  ---------- ---------- ---------- ---------- ---------- ---------- ---------- ---------- ---------- ---------- ----------
NW_003775878                  ---------- ---------- ---------- ---------- ---------- ---------- ---------- ---------- ---------- ---------- ----------
DQ018752                      ---------- ---------- ---------- ---------- ---------- ---------- ---------- ---------- ---------- ---------- ----------
DQ018757                      ---------- ---------- ---------- ---------- ---------- ---------- ---------- ---------- ---------- ---------- ----------
DQ112354                      ---------- ---------- ---------- ---------- ---------- ---------- ---------- ---------- ---------- ---------- ----------
FM165414                      ---------- ---------- ---------- ---------- ---------- ---------- ---------- ---------- ---------- ---------- ----------
FM165415                      ---------- ---------- ---------- ---------- ---------- ---------- ---------- ---------- ---------- ---------- ----------
AADN03000430                  ---------- ---------- ---------- ---------- ---------- ---------- ---------- ---------- ---------- ---------- ----------
AADN03001784                  ---------- ---------- ---------- ---------- ---------- ---------- ---------- ---------- ---------- ---------- ----------
AADN03001783                  ---------- ---------- ---------- ---------- ---------- ---------- ---------- ---------- ---------- ---------- ----------
DQ018754                      ---------- ---------- ---------- ---------- ---------- ---------- ---------- ---------- ---------- ---------- ----------
AADN03001782                  ---------- ---------- ---------- ---------- ---------- ---------- ---------- ---------- ---------- ---------- ----------
JN639848                      ---------- ---------- ---------- ---------- ---------- ---------- ---------- ---------- ---------- ---------- ----------
AADN03001670                  ---------- ---------- ---------- ---------- ---------- ---------- ---------- ---------- ---------- ---------- ----------
HQ873432                      ---------- ---------- ---------- ---------- ---------- ---------- ---------- ---------- ---------- ---------- ----------
AADN03001774                  ---------- ---------- ---------- ---------- ---------- ---------- ---------- ---------- ---------- ---------- ----------
AADN03001775                  ---------- ---------- ---------- ---------- ---------- ---------- ---------- ---------- ---------- ---------- ----------
EF552813                      TGAACGAGAT TCCCACTGTC CCTACCTACT CTCCAGCGAA ACCACAGCCA AGGGAACGGG CTTGGCGGAA TCAGCGGGGA AAGAAGACCC TGTTGAGCTT GACTCTAGTC
AADN03022685                  TGAACGAGAT TCCCACTGTC CCTATCTACT ATCCAGCGAA ACCACAGCCA AGGGAACGGG CTTGGCGGAA TCAGCGGGGA AAGAAGACCC TGTTGAGCTT GACTCTAG--
AADN03019346                  ---------- ---------- ---------- ---------- ---------- ---------- ---------- ---------- ---------- ---------- ----------
AADN03001776                  ---------- ---------- ---------- ---------- ---------- ---------- ---------- ---------- ---------- ---------- ----------
DQ018756                      ---------- ---------- ---------- ---------- ---------- ---------- ---------- ---------- ---------- ---------- ----------
AF173612                      ---------- ---------- ---------- ---------- ---------- ---------- ---------- ---------- ---------- ---------- ----------
Gallus_gallus_KT445934.2      TGAACGAGAT TCCCACTGTC CCTATCTACT ATCCAGCGAA ACCACAGCCA AGGGAACGGG CTTGGCGGAA TCAGCGGGGA AAGAAGACCC TGTTGAGCTT GACTCTAGTC

                              1111111111 1111111111 1111111111 1111111111 1111111111 1111111111 1111111111 1111111111 1111111111 1111111111 1111111111
                              0000000000 0000000000 0000000000 0000000000 0000000000 0000000000 0000000000 0000000000 0000000000 0000000000 0000000000
                              5555555555 5555555555 5555555555 5555555556 6666666666 6666666666 6666666666 6666666666 6666666666 6666666666 6666666666
                              6666666667 7777777778 8888888889 9999999990 0000000001 1111111112 2222222223 3333333334 4444444445 5555555556 6666666667
                              1234567890 1234567890 1234567890 1234567890 1234567890 1234567890 1234567890 1234567890 1234567890 1234567890 1234567890
AADN03001677                  ---------- ---------- ---------- ---------- ---------- ---------- ---------- ---------- ---------- ---------- ----------
AADN03001778                  ---------- ---------- ---------- ---------- ---------- ---------- ---------- ---------- ---------- ---------- ----------
AADN03001785                  ---------- ---------- ---------- ---------- ---------- ---------- ---------- ---------- ---------- ---------- ----------
AADN03001786                  ---------- ---------- ---------- ---------- ---------- ---------- ---------- ---------- ---------- ---------- ----------
AADN03001788                  ---------- ---------- ---------- ---------- ---------- ---------- ---------- ---------- ---------- ---------- ----------
AADN03014081                  ---------- ---------- ---------- ---------- ---------- ---------- ---------- ---------- ---------- ---------- ----------
AADN03015064                  ---------- ---------- ---------- ---------- ---------- ---------- ---------- ---------- ---------- ---------- ----------
AADN03026634                  ---------- ---------- ---------- ---------- ---------- ---------- ---------- ---------- ---------- ---------- ----------
NW_003775878                  ---------- ---------- ---------- ---------- ---------- ---------- ---------- ---------- ---------- ---------- ----------
DQ018752                      ---------- ---------- ---------- ---------- ---------- ---------- ---------- ---------- ---------- ---------- ----------
DQ018757                      ---------- ---------- ---------- ---------- ---------- ---------- ---------- ---------- ---------- ---------- ----------
DQ112354                      ---------- ---------- ---------- ---------- ---------- ---------- ---------- ---------- ---------- ---------- ----------
```

```
FM165414                    ---------- ---------- ---------- ---------- ---------- ---------- ---------- ---------- ---------- ---------- ----------
FM165415                    ---------- ---------- ---------- ---------- ---------- ---------- ---------- ---------- ---------- ---------- ----------
AADN03000430                ---------- ---------- ---------- ---------- ---------- ---------- ---------- ---------- ---------- ---------- ----------
AADN03001784                ---------- ---------- ---------- ---------- ---------- ---------- ---------- ---------- ---------- ---------- ----------
AADN03001783                ---------- ---------- ---------- ---------- ---------- ---------- ---------- ---------- ---------- ---------- ----------
DQ018754                    ---------- ---------- ---------- ---------- ---------- ---------- ---------- ---------- ---------- ---------- ----------
AADN03001782                ---------- ---------- ---------- ---------- ---------- ---------- ---------- ---------- ---------- ---------- ----------
JN639848                    ---------- ---------- ---------- ---------- ---------- ---------- ---------- ---------- ---------- ---------- ----------
AADN03001670                ---------- ---------- ---------- ---------- ---------- ---------- ---------- ---------- ---------- ---------- ----------
HQ873432                    ---------- ---------- ---------- ---------- ---------- ---------- ---------- ---------- ---------- ---------- ----------
AADN03001774                ---------- ---------- ---------- ---------- ---------- ---------- ---------- ---------- ---------- ---------- ----------
AADN03001775                ---------- ---------- ---------- ---------- ---------- ---------- ---------- ---------- ---------- ---------- ----------
EF552813                    TGGCGCTGTG AAGAGACATG AGAGGTGTAG AATAAGTGGG AGGCCCCGCG GTCGCGCGAC CCGCGCCGCG GCCCGGCCGC CGGTGAAATA CCACTACTCT GATCGTTTTT
AADN03022685                ---------- ---------- ---------- ---------- ---------- ---------- ---------- ---------- ---------- ---------- ----------
AADN03019346                ---------- ---------- ---------- ---------- ---------- ---------- ---------- ---------- ---------- ---------- ----------
AADN03001776                ---------- ---------- ---------- ---------- ---------- ---------- ---------- ---------- ---------- ---------- ----------
DQ018756                    ---------- ---------- ---------- ---------- ---------- ---------- ---------- ---------- ---------- ---------- ----------
AF173612                    ---------- ---------- ---------- ---------- ---------- ---------- ---------- ---------- ---------- ---------- ----------
Gallus_gallus_KT445934.2    TGGCGCTGTG AAGAGACATG AGAGGTGTAG AATAAGTGGG AGGCCCCGCG GTCGCGCGAC CCGCGCCGCG GCCCGGCCGC CGGTGAAATA CCACTACTCT GATCGTTTTT

                            1111111111 1111111111 1111111111 1111111111 1111111111 1111111111 1111111111 1111111111 1111111111 1111111111 1111111111
                            0000000000 0000000000 0000000000 0000000000 0000000000 0000000000 0000000000 0000000000 0000000000 0000000000 0000000000
                            6666666666 6666666666 6666666667 7777777777 7777777777 7777777777 7777777777 7777777777 7777777777 7777777777 7777777777
                            7777777778 8888888889 9999999990 0000000001 1111111112 2222222223 3333333334 4444444445 5555555556 6666666667 7777777778
                            1234567890 1234567890 1234567890 1234567890 1234567890 1234567890 1234567890 1234567890 1234567890 1234567890 1234567890
AADN03001677                ---------- ---------- ---------- ---------- ---------- ---------- ---------- ---------- ---------- ---------- ----------
AADN03001778                ---------- ---------- ---------- ---------- ---------- ---------- ---------- ---------- ---------- ---------- ----------
AADN03001785                ---------- ---------- ---------- ---------- ---------- ---------- ---------- ---------- ---------- ---------- ----------
AADN03001786                ---------- ---------- ---------- ---------- ---------- ---------- ---------- ---------- ---------- ---------- ----------
AADN03001788                ---------- ---------- ---------- ---------- ---------- ---------- ---------- ---------- ---------- ---------- ----------
AADN03014081                ---------- ---------- ---------- ---------- ---------- ---------- ---------- ---------- ---------- ---------- ----------
AADN03015064                ---------- ---------- ---------- ---------- ---------- ---------- ---------- ---------- ---------- ---------- ----------
AADN03026634                ---------- ---------- ---------- ---------- ---------- ---------- ---------- ---------- ---------- ---------- ----------
NW_003775878                ---------- ---------- ---------- ---------- ---------- ---------- ---------- ---------- ---------- ---------- ----------
DQ018752                    ---------- ---------- ---------- ---------- ---------- ---------- ---------- ---------- ---------- ---------- ----------
DQ018757                    ---------- ---------- ---------- ---------- ---------- ---------- ---------- ---------- ---------- ---------- ----------
DQ112354                    ---------- ---------- ---------- ---------- ---------- ---------- ---------- ---------- ---------- ---------- ----------
FM165414                    ---------- ---------- ---------- ---------- ---------- ---------- ---------- ---------- ---------- ---------- ----------
FM165415                    ---------- ---------- ---------- ---------- ---------- ---------- ---------- ---------- ---------- ---------- ----------
AADN03000430                ---------- ---------- ---------- ---------- ---------- ---------- ---------- ---------- ---------- ---------- ----------
AADN03001784                ---------- ---------- ---------- ---------- ---------- ---------- ---------- ---------- ---------- ---------- ----------
AADN03001783                ---------- ---------- ---------- ---------- ---------- ---------- ---------- ---------- ---------- ---------- ----------
DQ018754                    ---------- ---------- ---------- ---------- ---------- ---------- ---------- ---------- ---------- ---------- ----------
AADN03001782                ---------- ---------- ---------- ---------- ---------- ---------- ---------- ---------- ---------- ---------- ----------
JN639848                    ---------- ---------- ---------- ---------- ---------- ---------- ---------- ---------- ---------- ---------- ----------
AADN03001670                ---------- ---------- ---------- ---------- ---------- ---------- ---------- ---------- ---------- ---------- ----------
HQ873432                    ---------- ---------- ---------- ---------- ---------- ---------- ---------- ---------- ---------- ---------- ----------
AADN03001774                ---------- ---------- ---------- ---------- ---------- ---------- ---------- ---------- ---------- ---------- ----------
AADN03001775                ---------- ---------- ---------- ---------- ---------- ---------- ---------- ---------- ---------- ---------- ----------
EF552813                    TCACTTACCC GGTGAGGCGG GGGGGCGAGC CCCGAGGGGC TCTCGCTTCT GGCGCCAAGC GCCCGGCGCG CGCCGGGCGC GACCCGCTCC GGGGACAGCG TCAGGTGGGG
AADN03022685                ---------- ---------- ---------- ---------- ---------- ---------- ---------- ---------- ---------- ---------- ----------
AADN03019346                ---------- ---------- ---------- ---------- ---------- ---------- ---------- ---------- ---------- ---------- ----------
AADN03001776                ---------- ---------- ---------- ---------- ---------- ---------- ---------- ---------- ---------- ---------- ----------
DQ018756                    ---------- ---------- ---------- ---------- ---------- ---------- ---------- ---------- ---------- ---------- ----------
AF173612                    ---------- ---------- ---------- ---------- ---------- ---------- ---------- ---------- ---------- ---------- ----------
```

```
Gallus_gallus_KT445934.2    TCACTTACCC GGTGAGGCGG GGGGGCGAGC CCCGAGGGGC TCTCGCTTCT GGCGCCAAGC GCCCGGCGCG CGCCGGGCGC GACCCGCTCC GGGGACAGCG TCAGGTGGGG

                            1111111111 1111111111 1111111111 1111111111 1111111111 1111111111 1111111111 1111111111 1111111111 1111111111 1111111111
                            0000000000 0000000000 0000000000 0000000000 0000000000 0000000000 0000000000 0000000000 0000000000 0000000000 0000000000
                            7777777777 7777777778 8888888888 8888888888 8888888888 8888888888 8888888888 8888888888 8888888888 8888888888 8888888888
                            8888888889 9999999990 0000000001 1111111112 2222222223 3333333334 4444444445 5555555556 6666666667 7777777778 8888888889
                            1234567890 1234567890 1234567890 1234567890 1234567890 1234567890 1234567890 1234567890 1234567890 1234567890 1234567890
AADN03001677                ---------- ---------- ---------- ---------- ---------- ---------- ---------- ---------- ---------- ---------- ----------
AADN03001778                ---------- ---------- ---------- ---------- ---------- ---------- ---------- ---------- ---------- ---------- ----------
AADN03001785                ---------- ---------- ---------- ---------- ---------- ---------- ---------- ---------- ---------- ---------- ----------
AADN03001786                ---------- ---------- ---------- ---------- ---------- ---------- ---------- ---------- ---------- ---------- ----------
AADN03001788                ---------- ---------- ---------- ---------- ---------- ---------- ---------- ---------- ---------- ---------- ----------
AADN03014081                ---------- ---------- ---------- ---------- ---------- ---------- ---------- ---------- ---------- ---------- ----------
AADN03015064                ---------- ---------- ---------- ---------- ---------- ---------- ---------- ---------- ---------- ---------- ----------
AADN03026634                ---------- ---------- ---------- ---------- ---------- ---------- ---------- ---------- ---------- ---------- ----------
NW_003775878                ---------- ---------- ---------- ---------- ---------- ---------- ---------- ---------- ---------- ---------- ----------
DQ018752                    ---------- ---------- ---------- ---------- ---------- ---------- ---------- ---------- ---------- ---------- ----------
DQ018757                    ---------- ---------- ---------- ---------- ---------- ---------- ---------- ---------- ---------- ---------- ----------
DQ112354                    ---------- ---------- ---------- ---------- ---------- ---------- ---------- ---------- ---------- ---------- ----------
FM165414                    ---------- ---------- ---------- ---------- ---------- ---------- ---------- ---------- ---------- ---------- ----------
FM165415                    ---------- ---------- ---------- ---------- ---------- ---------- ---------- ---------- ---------- ---------- ----------
AADN03000430                ---------- ---------- ---------- ---------- ---------- ---------- ---------- ---------- ---------- ---------- ----------
AADN03001784                ---------- ---------- ---------- ---------- ---------- ---------- ---------- ---------- ---------- ---------- ----------
AADN03001783                ---------- ---------- ---------- ---------- ---------- ---------- ---------- ---------- ---------- ---------- ----------
DQ018754                    ---------- ---------- ---------- ---------- ---------- ---------- ---------- ---------- ---------- ---------- ----------
AADN03001782                ---------- ---------- ---------- ---------- ---------- ---------- ---------- ---------- ---------- ---------- ----------
JN639848                    ---------- ---------- ---------- ---------- ---------- ---------- ---------- ---------- ---------- ---------- ----------
AADN03001670                ---------- ---------- ---------- ---------- ---------- ---------- ---------- ---------- ---------- ---------- ----------
HQ873432                    ---------- ---------- ---------- ---------- ---------- ---------- ---------- ---------- ---------- ---------- ----------
AADN03001774                ---------- ---------- ---------- ---------- ---------- ---------- ---------- ---------- ---------- ---------- ----------
AADN03001775                ---------- ---------- ---------- ---------- ---------- ---------- ---------- ---------- ---------- ---------- ----------
EF552813                    AGTTTGACTG GGGCGGTACA CCTGTCAAAG CGTAACGCAG GTGTCCTAAG GCGAGCTCAG GGAGGCCAGA AACCTCCCGT GGAGCAGAAG GGCAAAAGCT CGCTTGATCT
AADN03022685                ---------- ---------- ---------- ---------- ---------- ---------- ---------- ---------- ---------- ---------- ----------
AADN03019346                ---------- ---------- ---------- ---------- ---------- ---------- ---------- ---------- ---------- ---------- ----------
AADN03001776                ---------- ---------- ---------- ---------- ---------- ---------- ---------- ---------- ---------- ---------- ----------
DQ018756                    ---------- ---------- ---------- ---------- ---------- ---------- ---------- ---------- ---------- ---------- ----------
AF173612                    ---------- ---------- ---------- ---------- ---------- ---------- ---------- ---------- ---------- ---------- ----------
Gallus_gallus_KT445934.2    AGTTTGACTG GGGCGGTACA CCTGTCAAAG CGTAACGCAG GTGTCCTAAG GCGAGCTCAG GGAGGCCAGA AACCTCCCGT GGAGCAGAAG GGCAAAAGCT CGCTTGATCT

                            1111111111 1111111111 1111111111 1111111111 1111111111 1111111111 1111111111 1111111111 1111111111 1111111111 1111111111
                            0000000000 0000000000 0000000000 0000000000 0000000000 0000000000 0000000000 0000000000 0000000000 0000000000 0000000001
                            8888888889 9999999999 9999999999 9999999999 9999999999 9999999999 9999999999 9999999999 9999999999 9999999999 9999999990
                            9999999990 0000000001 1111111112 2222222223 3333333334 4444444445 5555555556 6666666667 7777777778 8888888889 9999999990
                            1234567890 1234567890 1234567890 1234567890 1234567890 1234567890 1234567890 1234567890 1234567890 1234567890 1234567890
AADN03001677                ---------- ---------- ---------- ---------- ---------- ---------- ---------- ---------- ---------- ---------- ----------
AADN03001778                ---------- ---------- ---------- ---------- ---------- ---------- ---------- ---------- ---------- ---------- ----------
AADN03001785                ---------- ---------- ---------- ---------- ---------- ---------- ---------- ---------- ---------- ---------- ----------
AADN03001786                ---------- ---------- ---------- ---------- ---------- ---------- ---------- ---------- ---------- ---------- ----------
AADN03001788                ---------- ---------- ---------- ---------- ---------- ---------- ---------- ---------- ---------- ---------- ----------
AADN03014081                ---------- ---------- ---------- ---------- ---------- ---------- ---------- ---------- ---------- ---------- ----------
AADN03015064                ---------- ---------- ---------- ---------- ---------- ---------- ---------- ---------- ---------- ---------- ----------
AADN03026634                ---------- ---------- ---------- ---------- ---------- ---------- ---------- ---------- ---------- ---------- ----------
NW_003775878                ---------- ---------- ---------- ---------- ---------- ---------- ---------- ---------- ---------- ---------- ----------
DQ018752                    ---------- ---------- ---------- ---------- ---------- ---------- ---------- ---------- ---------- ---------- ----------
DQ018757                    ---------- ---------- ---------- ---------- ---------- ---------- ---------- ---------- ---------- ---------- ----------
```

```
DQ112354                     ---------- ---------- ---------- ---------- ---------- ---------- ---------- ---------- ---------- ---------- ----------
FM165414                     ---------- ---------- ---------- ---------- ---------- ---------- ---------- ---------- ---------- ---------- ----------
FM165415                     ---------- ---------- ---------- ---------- ---------- ---------- ---------- ---------- ---------- ---------- ----------
AADN03000430                 ---------- ---------- ---------- ---------- ---------- ---------- ---------- ---------- ---------- ---------- ----------
AADN03001784                 ---------- ---------- ---------- ---------- ---------- ---------- ---------- ---------- ---------- ---------- ----------
AADN03001783                 ---------- ---------- ---------- ---------- ---------- ---------- ---------- ---------- ---------- ---------- ----------
DQ018754                     ---------- ---------- ---------- ---------- ---------- ---------- ---------- ---------- ---------- ---------- ----------
AADN03001782                 ---------- ---------- ---------- ---------- ---------- ---------- ---------- ---------- ---------- ---------- ----------
JN639848                     ---------- ---------- ---------- ---------- ---------- ---------- ---------- ---------- ---------- ---------- ----------
AADN03001670                 ---------- ---------- ---------- ---------- ---------- ---------- ---------- ---------- ---------- ---------- ----------
HQ873432                     ---------- ---------- ---------- ---------- ---------- ---------- ---------- ---------- ---------- ---------- ----------
AADN03001774                 ---------- ---------- ---------- ---------- ---------- ---------- ---------- ---------- ---------- ---------- ----------
AADN03001775                 ---------- ---------- ---------- ---------- ---------- ---------- ---------- ---------- ---------- ---------- ----------
EF552813                     TGATTTTCAG TACGAATACA GACCGTGAAA GCGGGGCCTC ACGATCCTTC TGACTTTTTG GGTTTTAAGC AGGAGGTGTC AGAAAAGTTA CCACAGGGAT AACTGGCTTG
AADN03022685                 ---------- ---------- ---------- ---------- ---------- ---------- ---------- ---------- ---------- ---------- ----------
AADN03019346                 ---------- ---------- ---------- ---------- ---------- ---------- ---------- ---------- ---------- ---------- ----------
AADN03001776                 ---------- ---------- ---------- ---------- ---------- ---------- ---------- ---------- ---------- ---------- ----------
DQ018756                     ---------- ---------- ---------- ---------- ---------- ---------- ---------- ---------- ---------- ---------- ----------
AF173612                     ---------- ---------- ---------- ---------- ---------- ---------- ---------- ---------- ---------- ---------- ----------
Gallus_gallus_KT445934.2     TGATTTTCAG TACGAATACA GACCGTGAAA GCGGGGCCTC ACGATCCTTC TGACTTTTTG GGTTTTAAGC AGGAGGTGTC AGAAAAGTTA CCACAGGGAT AACTGGCTTG

                             1111111111 1111111111 1111111111 1111111111 1111111111 1111111111 1111111111 1111111111 1111111111 1111111111 1111111111
                             1111111111 1111111111 1111111111 1111111111 1111111111 1111111111 1111111111 1111111111 1111111111 1111111111 1111111111
                             0000000000 0000000000 0000000000 0000000000 0000000000 0000000000 0000000000 0000000000 0000000000 0000000001 1111111111
                             0000000001 1111111112 2222222223 3333333334 4444444445 5555555556 6666666667 7777777778 8888888889 9999999990 0000000001
                             1234567890 1234567890 1234567890 1234567890 1234567890 1234567890 1234567890 1234567890 1234567890 1234567890 1234567890
AADN03001677                 ---------- ---------- ---------- ---------- ---------- ---------- ---------- ---------- ---------- ---------- ----------
AADN03001778                 ---------- ---------- ---------- ---------- ---------- ---------- ---------- ---------- ---------- ---------- ----------
AADN03001785                 ---------- ---------- ---------- ---------- ---------- ---------- ---------- ---------- ---------- ---------- ----------
AADN03001786                 ---------- ---------- ---------- ---------- ---------- ---------- ---------- ---------- ---------- ---------- ----------
AADN03001788                 ---------- ---------- ---------- ---------- ---------- ---------- ---------- ---------- ---------- ---------- ----------
AADN03014081                 ---------- ---------- ---------- ---------- ---------- ---------- ---------- ---------- ---------- ---------- ----------
AADN03015064                 ---------- ---------- ---------- ---------- ---------- ---------- ---------- ---------- ---------- ---------- ----------
AADN03026634                 ---------- ---------- ---------- ---------- ---------- ---------- ---------- ---------- ---------- ---------- ----------
NW_003775878                 ---------- ---------- ---------- ---------- ---------- ---------- ---------- ---------- ---------- ---------- ----------
DQ018752                     ---------- ---------- ---------- ---------- ---------- ---------- ---------- ---------- ---------- ---------- ----------
DQ018757                     ---------- ---------- ---------- ---------- ---------- ---------- ---------- ---------- ---------- ---------- ----------
DQ112354                     ---------- ---------- ---------- ---------- ---------- ---------- ---------- ---------- ---------- ---------- ----------
FM165414                     ---------- ---------- ---------- ---------- ---------- ---------- ---------- ---------- ---------- ---------- ----------
FM165415                     ---------- ---------- ---------- ---------- ---------- ---------- ---------- ---------- ---------- ---------- ----------
AADN03000430                 ---------- ---------- ---------- ---------- ---------- ---------- ---------- ---------- ---------- ---------- ----------
AADN03001784                 ---------- ---------- ---------- ---------- ---------- ---------- ---------- ---------- ---------- ---------- ----------
AADN03001783                 ---------- ---------- ---------- ---------- ---------- ---------- ---------- ---------- ---------- ---------- ----------
DQ018754                     ---------- ---------- ---------- ---------- ---------- ---------- ---------- ---------- ---------- ---------- ----------
AADN03001782                 ---------- ---------- ---------- ---------- ---------- ---------- ---------- ---------- ---------- ---------- ----------
JN639848                     ---------- ---------- ---------- ---------- ---------- ---------- ---------- ---------- ---------- ---------- ----------
AADN03001670                 ---------- ---------- ---------- ---------- ---------- ---------- ---------- ---------- ---------- ---------- ----------
HQ873432                     ---------- ---------- ---------- ---------- ---------- ---------- ---------- ---------- ---------- ---------- ----------
AADN03001774                 ---------- ---------- ---------- ---------- ---------- ---------- ---------- ---------- ---------- ---------- ----------
AADN03001775                 ---------- ---------- ---------- ---------- ---------- ---------- ---------- ---------- ---------- ---------- ----------
EF552813                     TGGCGGCCAA GCGTTCATAG CGACGTCGCT TTTTGATCCT TCGATGTCGG CTCTTCCTAT CATTGTGAAG CAGAATTCAC CAAGCGTTGG AT-------- ----------
AADN03022685                 ---------- ---------- ---------- ---------- ---------- ---------- ---------- ---------- ---------- ---------- ----------
AADN03019346                 ---------- ---------- ---------- ---------- ---------- ---------- ---------- ---------- ---------- ---------- ----------
AADN03001776                 ---------- ---------- ---------- ---------- ---------- ---------- ---------- ---------- ---------- ---------- ----------
DQ018756                     ---------- ---------- ---------- ---------- ---------- ---------- ---------- ---------- ---------- ---------- ----------
```

```
AF173612                    ---------- ---------- ---------- ---------- ---------- ---------- ---------- ---------- ---------- ---------- ----------
Gallus_gallus_KT445934.2    TGGCGGCCAA GCGTTCATAG CGACGTCGCT TTTTGATCCT TCGATGTCGG CTCTTCCTAT CATTGTGAAG CAGAATTCAC CAAGCGTTGG ATTGTTCACC CACTAATAGG

                            1111111111 1111111111 1111111111 1111111111 1111111111 1111111111 1111111111 1111111111 1111111111 1111111111 1111111111
                            1111111111 1111111111 1111111111 1111111111 1111111111 1111111111 1111111111 1111111111 1111111111 1111111111 1111111111
                            1111111111 1111111111 1111111111 1111111111 1111111111 1111111111 1111111111 1111111111 1111111112 2222222222 2222222222
                            1111111112 2222222223 3333333334 4444444445 5555555556 6666666667 7777777778 8888888889 9999999990 0000000001 1111111112
                            1234567890 1234567890 1234567890 1234567890 1234567890 1234567890 1234567890 1234567890 1234567890 1234567890 1234567890
AADN03001677                ---------- ---------- ---------- ---------- ---------- ---------- ---------- ---------- ---------- ---------- ----------
AADN03001778                ---------- ---------- ---------- ---------- ---------- ---------- ---------- ---------- ---------- ---------- ----------
AADN03001785                ---------- ---------- ---------- ---------- ---------- ---------- ---------- ---------- ---------- ---------- ----------
AADN03001786                ---------- ---------- ---------- ---------- ---------- ---------- ---------- ---------- ---------- ---------- ----------
AADN03001788                ---------- ---------- ---------- ---------- ---------- ---------- ---------- ---------- ---------- ---------- ----------
AADN03014081                ---------- ---------- ---------- ---------- ---------- ---------- ---------- ---------- ---------- ---------- ----------
AADN03015064                ---------- ---------- ---------- ---------- ---------- ---------- ---------- ---------- ---------- ---------- ----------
AADN03026634                ---------- ---------- ---------- ---------- ---------- ---------- ---------- ---------- ---------- ---------- ----------
NW_003775878                ---------- ---------- ---------- ---------- ---------- ---------- ---------- ---------- ---------- ---------- ----------
DQ018752                    ---------- ---------- ---------- ---------- ---------- ---------- ---------- ---------- ---------- ---------- ----------
DQ018757                    ---------- ---------- ---------- ---------- ---------- ---------- ---------- ---------- ---------- ---------- ----------
DQ112354                    ---------- ---------- ---------- ---------- ---------- ---------- ---------- ---------- ---------- ---------- ----------
FM165414                    ---------- ---------- ---------- ---------- ---------- ---------- ---------- ---------- ---------- ---------- ----------
FM165415                    ---------- ---------- ---------- ---------- ---------- ---------- ---------- ---------- ---------- ---------- ----------
AADN03000430                ---------- ---------- ---------- ---------- ---------- ---------- ---------- ---------- ---------- ---------- ----------
AADN03001784                ---------- ---------- ---------- ---------- ---------- ---------- ---------- ---------- ---------- ---------- ----------
AADN03001783                ---------- ---------- ---------- ---------- ---------- ---------- ---------- ---------- ---------- ---------- ----------
DQ018754                    ---------- ---------- ---------- ---------- ---------- ---------- ---------- ---------- ---------- ---------- ----------
AADN03001782                ---------- ---------- ---------- ---------- ---------- ---------- ---------- ---------- ---------- ---------- ----------
JN639848                    ---------- ---------- ---------- ---------- ---------- ---------- ---------- ---------- ---------- ---------- ----------
AADN03001670                ---------- ---------- ---------- ---------- ---------- ---------- ---------- ---------- ---------- ---------- ----------
HQ873432                    ---------- ---------- ---------- ---------- ---------- ---------- ---------- ---------- ---------- ---------- ----------
AADN03001774                ---------- ---------- ---------- ---------- ---------- ---------- ---------- ---------- ---------- ---------- ----------
AADN03001775                ---------- ---------- ---------- ---------- ---------- ---------- ---------- ---------- ---------- ---------- ----------
EF552813                    ---------- ---------- ---------- ---------- ---------- ---------- ---------- ---------- ---------- ---------- ----------
AADN03022685                ---------- ---------- ---------- ---------- ---------- ---------- ---------- ---------- ---------- ---------- ----------
AADN03019346                ---------- ---------- ---------- ---------- ---------- ---------- ---------- ---------- ---------- ---------- ----------
AADN03001776                ---------- ---------- ---------- ---------- ---------- ---------- ---------- ---------- ---------- ---------- ----------
DQ018756                    ---------- ---------- ---------- ---------- ---------- ---------- ---------- ---------- ---------- ---------- ----------
AF173612                    ---------- ---------- ---------- ---------- ---------- ---------- ---------- ---------- ---------- ---------- ----------
Gallus_gallus_KT445934.2    GAACGTGAGC TGGGTTTAGA CCGTCGTGAG ACAGGTTAGT TTTACCCTAC TGATGATGTG TTGTTGCGCT AGTAATCCTG CTCAGTACGA GAGGAACCGC AGGTTCAGAC

                            1111111111 1111111111 1111111111 1111111111 1111111111 1111111111 1111111111 1111111111 1111111111 1111111111 1111111111
                            1111111111 1111111111 1111111111 1111111111 1111111111 1111111111 1111111111 1111111111 1111111111 1111111111 1111111111
                            2222222222 2222222222 2222222222 2222222222 2222222222 2222222222 2222222222 2222222222 3333333333 3333333333 3333333333
                            2222222223 3333333334 4444444445 5555555556 6666666667 7777777778 8888888889 9999999990 0000000001 1111111112 2222222223
                            1234567890 1234567890 1234567890 1234567890 1234567890 1234567890 1234567890 1234567890 1234567890 1234567890 1234567890
AADN03001677                ---------- ---------- ---------- ---------- ---------- ---------- ---------- ---------- ---------- ---------- ----------
AADN03001778                ---------- ---------- ---------- ---------- ---------- ---------- ---------- ---------- ---------- ---------- ----------
AADN03001785                ---------- ---------- ---------- ---------- ---------- ---------- ---------- ---------- ---------- ---------- ----------
AADN03001786                ---------- ---------- ---------- ---------- ---------- ---------- ---------- ---------- ---------- ---------- ----------
AADN03001788                ---------- ---------- ---------- ---------- ---------- ---------- ---------- ---------- ---------- ---------- ----------
AADN03014081                ---------- ---------- ---------- ---------- ---------- ---------- ---------- ---------- ---------- ---------- ----------
AADN03015064                ---------- ---------- ---------- ---------- ---------- ---------- ---------- ---------- ---------- ---------- ----------
AADN03026634                ---------- ---------- ---------- ---------- ---------- ---------- ---------- ---------- ---------- ---------- ----------
NW_003775878                ---------- ---------- ---------- ---------- ---------- ---------- ---------- ---------- ---------- ---------- ----------
DQ018752                    ---------- ---------- ---------- ---------- ---------- ---------- ---------- ---------- ---------- ---------- ----------
```

```
DQ018757                     ---------- ---------- ---------- ---------- ---------- ---------- ---------- ---------- ---------- ---------- ----------
DQ112354                     ---------- ---------- ---------- ---------- ---------- ---------- ---------- ---------- ---------- ---------- ----------
FM165414                     ---------- ---------- ---------- ---------- ---------- ---------- ---------- ---------- ---------- ---------- ----------
FM165415                     ---------- ---------- ---------- ---------- ---------- ---------- ---------- ---------- ---------- ---------- ----------
AADN03000430                 ---------- ---------- ---------- ---------- ---------- ---------- ---------- ---------- ---------- ---------- ----------
AADN03001784                 ---------- ---------- ---------- ---------- ---------- ---------- ---------- ---------- ---------- ---------- ----------
AADN03001783                 ---------- ---------- ---------- ---------- ---------- ---------- ---------- ---------- ---------- ---------- ----------
DQ018754                     ---------- ---------- ---------- ---------- ---------- ---------- ---------- ---------- ---------- ---------- ----------
AADN03001782                 ---------- ---------- ---------- ---------- ---------- ---------- ---------- ---------- ---------- ---------- ----------
JN639848                     ---------- ---------- ---------- ---------- ---------- ---------- ---------- ---------- ---------- ---------- ----------
AADN03001670                 ---------- ---------- ---------- ---------- ---------- ---------- ---------- ---------- ---------- ---------- ----------
HQ873432                     ---------- ---------- ---------- ---------- ---------- ---------- ---------- ---------- ---------- ---------- ----------
AADN03001774                 ---------- ---------- ---------- ---------- ---------- ---------- ---------- ---------- ---------- ---------- ----------
AADN03001775                 ---------- ---------- ---------- ---------- ---------- ---------- ---------- ---------- ---------- ---------- ----------
EF552813                     ---------- ---------- ---------- ---------- ---------- ---------- ---------- ---------- ---------- ---------- ----------
AADN03022685                 ---------- ---------- ---------- ---------- ---------- ---------- ---------- ---------- ---------- ---------- ----------
AADN03019346                 ---------- ---------- ---------- ---------- ---------- ---------- ---------- ---------- ---------- ---------- ----------
AADN03001776                 ---------- ---------- ---------- ---------- ---------- ---------- ---------- ---------- ---------- ---------- ----------
DQ018756                     ---------- ---------- ---------- ---------- ---------- ---------- ---------- ---------- ---------- ---------- ----------
AF173612                     ---------- ---------- ---------- ---------- ---------- ---------- ---------- ---------- ---------- ---------- ----------
Gallus_gallus_KT445934.2     ATTTGGTGTA TGTGCTTGGC TGAGGAGCCA CTGGAGCGAG GCTACCATCT GTGGGATTAT GACTGAACGC CTCTAAGTCA GAATCCCCCC TAAACGTAGC GATACCGCAG

                             1111111111 1111111111 1111111111 1111111111 1111111111 1111111111 1111111111 1111111111 1111111111 1111111111 1111111111
                             1111111111 1111111111 1111111111 1111111111 1111111111 1111111111 1111111111 1111111111 1111111111 1111111111 1111111111
                             3333333333 3333333333 3333333333 3333333333 3333333333 3333333333 3333333334 4444444444 4444444444 4444444444 4444444444
                             3333333334 4444444445 5555555556 6666666667 7777777778 8888888889 9999999990 0000000001 1111111112 2222222223 3333333334
                             1234567890 1234567890 1234567890 1234567890 1234567890 1234567890 1234567890 1234567890 1234567890 1234567890 1234567890
AADN03001677                 ---------- ---------- ---------- ---------- ---------- ---------- ---------- ---------- ---------- ---------- ----------
AADN03001778                 ---------- ---------- ---------- ---------- ---------- ---------- ---------- ---------- ---------- ---------- ----------
AADN03001785                 ---------- ---------- ---------- ---------- ---------- ---------- ---------- ---------- ---------- ---------- ----------
AADN03001786                 ---------- ---------- ---------- ---------- ---------- ---------- ---------- ---------- ---------- ---------- ----------
AADN03001788                 ---------- ---------- ---------- ---------- ---------- ---------- ---------- ---------- ---------- ---------- ----------
AADN03014081                 ---------- ---------- ---------- ---------- ---------- ---------- ---------- ---------- ---------- ---------- ----------
AADN03015064                 ---------- ---------- ---------- ---------- ---------- ---------- ---------- ---------- ---------- ---------- ----------
AADN03026634                 ---------- ---------- ---------- ---------- ---------- ---------- ---------- ---------- ---------- ---------- ----------
NW_003775878                 ---------- ---------- ---------- ---------- ---------- ---------- ---------- ---------- ---------- ---------- ----------
DQ018752                     ---------- ---------- ---------- ---------- ---------- ---------- ---------- ---------- ---------- ---------- ----------
DQ018757                     ---------- ---------- ---------- ---------- ---------- ---------- ---------- ---------- ---------- ---------- ----------
DQ112354                     ---------- ---------- ---------- ---------- ---------- ---------- ---------- ---------- ---------- ---------- ----------
FM165414                     ---------- ---------- ---------- ---------- ---------- ---------- ---------- ---------- ---------- ---------- ----------
FM165415                     ---------- ---------- ---------- ---------- ---------- ---------- ---------- ---------- ---------- ---------- ----------
AADN03000430                 ---------- ---------- ---------- ---------- ---------- ---------- ---------- ---------- ---------- ---------- ----------
AADN03001784                 ---------- ---------- ---------- ---------- ---------- ---------- ---------- ---------- ---------- ---------- ----------
AADN03001783                 ---------- ---------- ---------- ---------- ---------- ---------- ---------- ---------- ---------- ---------- ----------
DQ018754                     ---------- ---------- ---------- ---------- ---------- ---------- ---------- ---------- ---------- ---------- ----------
AADN03001782                 ---------- ---------- ---------- ---------- ---------- ---------- ---------- ---------- ---------- ---------- ----------
JN639848                     ---------- ---------- ---------- ---------- ---------- ---------- ---------- ---------- ---------- ---------- ----------
AADN03001670                 ---------- ---------- ---------- ---------- ---------- ---------- ---------- ---------- ---------- ---------- ----------
HQ873432                     ---------- ---------- ---------- ---------- ---------- ---------- ---------- ---------- ---------- ---------- ----------
AADN03001774                 ---------- ---------- ---------- ---------- ---------- ---------- ---------- ---------- ---------- ---------- ----------
AADN03001775                 ---------- ---------- ---------- ---------- ---------- ---------- ---------- ---------- ---------- ---------- ----------
EF552813                     ---------- ---------- ---------- ---------- ---------- ---------- ---------- ---------- ---------- ---------- ----------
AADN03022685                 ---------- ---------- ---------- ---------- ---------- ---------- ---------- ---------- ---------- ---------- ----------
AADN03019346                 ---------- ---------- ---------- ---------- ---------- ---------- ---------- ---------- ---------- ---------- ----------
AADN03001776                 ---------- ---------- ---------- ---------- ---------- ---------- ---------- ---------- ---------- ---------- ----------
```

```
DQ018756                      ---------- ---------- ---------- ---------- ---------- ---------- ---------- ---------- ---------- ---------- ----------
AF173612                      ---------- ---------- ---------- ---------- ---------- ---------- ---------- ---------- ---------- ---------- ----------
Gallus_gallus_KT445934.2      CGCCGAGGCG CCTCGGTGGG CTCGCGATAG CCGGCCGCCG CCCCCCTCGG GCGGGCGGTC GGTGCGGAGC GCCGCTCGTG GTCGGGACCG GAGCGCGGAC AGATGTGGCG

                              1111111111 1111111111 1111111111 1111111111 1111111111 1111111111 1111111111 1111111111 1111111111 1111111111 1111111111
                              1111111111 1111111111 1111111111 1111111111 1111111111 1111111111 1111111111 1111111111 1111111111 1111111111 1111111111
                              4444444444 4444444444 4444444444 4444444444 4444444444 4444444445 5555555555 5555555555 5555555555 5555555555 5555555555
                              4444444445 5555555556 6666666667 7777777778 8888888889 9999999990 0000000001 1111111112 2222222223 3333333334 4444444445
                              1234567890 1234567890 1234567890 1234567890 1234567890 1234567890 1234567890 1234567890 1234567890 1234567890 1234567890
AADN03001677                  ---------- ---------- ---------- ---------- ---------- ---------- ---------- ---------- ---------- ---------- ----------
AADN03001778                  ---------- ---------- ---------- ---------- ---------- ---------- ---------- ---------- ---------- ---------- ----------
AADN03001785                  ---------- ---------- ---------- ---------- ---------- ---------- ---------- ---------- ---------- ---------- ----------
AADN03001786                  ---------- ---------- ---------- ---------- ---------- ---------- ---------- ---------- ---------- ---------- ----------
AADN03001788                  ---------- ---------- ---------- ---------- ---------- ---------- ---------- ---------- ---------- ---------- ----------
AADN03014081                  ---------- ---------- ---------- ---------- ---------- ---------- ---------- ---------- ---------- ---------- ----------
AADN03015064                  ---------- ---------- ---------- ---------- ---------- ---------- ---------- ---------- ---------- ---------- ----------
AADN03026634                  ---------- ---------- ---------- ---------- ---------- ---------- ---------- ---------- ---------- ---------- ----------
NW_003775878                  ---------- ---------- ---------- ---------- ---------- ---------- ---------- ---------- ---------- ---------- ----------
DQ018752                      ---------- ---------- ---------- ---------- ---------- ---------- ---------- ---------- ---------- ---------- ----------
DQ018757                      ---------- ---------- ---------- ---------- ---------- ---------- ---------- ---------- ---------- ---------- ----------
DQ112354                      ---------- ---------- ---------- ---------- ---------- ---------- ---------- ---------- ---------- ---------- ----------
FM165414                      ---------- ---------- ---------- ---------- ---------- ---------- ---------- ---------- ---------- ---------- ----------
FM165415                      ---------- ---------- ---------- ---------- ---------- ---------- ---------- ---------- ---------- ---------- ----------
AADN03000430                  ---------- ---------- ---------- ---------- ---------- ---------- ---------- ---------- ---------- ---------- ----------
AADN03001784                  ---------- ---------- ---------- ---------- ---------- ---------- ---------- ---------- ---------- ---------- ----------
AADN03001783                  ---------- ---------- ---------- ---------- ---------- ---------- ---------- ---------- ---------- ---------- ----------
DQ018754                      ---------- ---------- ---------- ---------- ---------- ---------- ---------- ---------- ---------- ---------- ----------
AADN03001782                  ---------- ---------- ---------- ---------- ---------- ---------- ---------- ---------- ---------- ---------- ----------
JN639848                      ---------- ---------- ---------- ---------- ---------- ---------- ---------- ---------- ---------- ---------- ----------
AADN03001670                  ---------- ---------- ---------- ---------- ---------- ---------- ---------- ---------- ---------- ---------- ----------
HQ873432                      ---------- ---------- ---------- ---------- ---------- ---------- ---------- ---------- ---------- ---------- ----------
AADN03001774                  ---------- ---------- ---------- ---------- ---------- ---------- ---------- ---------- ---------- ---------- ----------
AADN03001775                  ---------- ---------- ---------- ---------- ---------- ---------- ---------- ---------- ---------- ---------- ----------
EF552813                      ---------- ---------- ---------- ---------- ---------- ---------- ---------- ---------- ---------- ---------- ----------
AADN03022685                  ---------- ---------- ---------- ---------- ---------- ---------- ---------- ---------- ---------- ---------- ----------
AADN03019346                  ---------- ---------- ---------- ---------- ---------- ---------- ---------- ---------- ---------- ---------- ----------
AADN03001776                  ---------- ---------- ---------- ---------- ---------- ---------- ---------- ---------- ---------- ---------- ----------
DQ018756                      ---------- ---------- ---------- ---------- ---------- ---------- ---------- ---------- ---------- ---------- ----------
AF173612                      ---------- ---------- ---------- ---------- ---------- ---------- ---------- ---------- ---------- ---------- ----------
Gallus_gallus_KT445934.2      CCGCCTCTCC CCCGCCGCGT ACCGCATGTT CGTGGGGAAC CCGGTGCTAA ATCATTCGTA GACGACCTGA TTCTGGGTCG GGGTTTCGTA CGTAGCAGAG CAGCTCCCTC

                              1111111111 1111111111 1111111111 1111111111 1111111111 1111111111 1111111111 1111111111 1111111111 1111111111 1111111111
                              1111111111 1111111111 1111111111 1111111111 1111111111 1111111111 1111111111 1111111111 1111111111 1111111111 1111111111
                              5555555555 5555555555 5555555555 5555555555 5555555556 6666666666 6666666666 6666666666 6666666666 6666666666 6666666666
                              5555555556 6666666667 7777777778 8888888889 9999999990 0000000001 1111111112 2222222223 3333333334 4444444445 5555555556
                              1234567890 1234567890 1234567890 1234567890 1234567890 1234567890 1234567890 1234567890 1234567890 1234567890 1234567890
AADN03001677                  ---------- ---------- ---------- ---------- ---------- ---------- ---------- ---------- ---------- ---------- ----------
AADN03001778                  ---------- ---------- ---------- ---------- ---------- ---------- ---------- ---------- ---------- ---------- ----------
AADN03001785                  ---------- ---------- ---------- ---------- ---------- ---------- ---------- ---------- ---------- ---------- ----------
AADN03001786                  ---------- ---------- ---------- ---------- ---------- ---------- ---------- ---------- ---------- ---------- ----------
AADN03001788                  ---------- ---------- ---------- ---------- ---------- ---------- ---------- ---------- ---------- ---------- ----------
AADN03014081                  ---------- ---------- ---------- ---------- ---------- ---------- ---------- ---------- ---------- ---------- ----------
AADN03015064                  ---------- ---------- ---------- ---------- ---------- ---------- ---------- ---------- ---------- ---------- ----------
AADN03026634                  ---------- ---------- ---------- ---------- ---------- ---------- ---------- ---------- ---------- ---------- ----------
NW_003775878                  ---------- ---------- ---------- ---------- ---------- ---------- ---------- ---------- ---------- ---------- ----------
```

```
DQ018752                     ---------- ---------- ---------- ---------- ---------- ---------- ---------- ---------- ---------- ---------- ----------
DQ018757                     ---------- ---------- ---------- ---------- ---------- ---------- ---------- ---------- ---------- ---------- ----------
DQ112354                     ---------- ---------- ---------- ---------- ---------- ---------- ---------- ---------- ---------- ---------- ----------
FM165414                     ---------- ---------- ---------- ---------- ---------- ---------- ---------- ---------- ---------- ---------- ----------
FM165415                     ---------- ---------- ---------- ---------- ---------- ---------- ---------- ---------- ---------- ---------- ----------
AADN03000430                 ---------- ---------- ---------- ---------- ---------- ---------- ---------- ---------- ---------- ---------- ----------
AADN03001784                 ---------- ---------- ---------- ---------- ---------- ---------- ---------- ---------- ---------- ---------- ----------
AADN03001783                 ---------- ---------- ---------- ---------- ---------- ---------- ---------- ---------- ---------- ---------- ----------
DQ018754                     ---------- ---------- ---------- ---------- ---------- ---------- ---------- ---------- ---------- ---------- ----------
AADN03001782                 ---------- ---------- ---------- ---------- ---------- ---------- ---------- ---------- ---------- ---------- ----------
JN639848                     ---------- ---------- ---------- ---------- ---------- ---------- ---------- ---------- ---------- ---------- ----------
AADN03001670                 ---------- ---------- ---------- ---------- ---------- ---------- ---------- ---------- ---------- ---------- ----------
HQ873432                     ---------- ---------- ---------- ---------- ---------- ---------- ---------- ---------- ---------- ---------- ----------
AADN03001774                 ---------- ---------- ---------- ---------- ---------- ---------- ---------- ---------- ---------- ---------- ----------
AADN03001775                 ---------- ---------- ---------- ---------- ---------- ---------- ---------- ---------- ---------- ---------- ----------
EF552813                     ---------- ---------- ---------- ---------- ---------- ---------- ---------- ---------- ---------- ---------- ----------
AADN03022685                 ---------- ---------- ---------- ---------- ---------- ---------- ---------- ---------- ---------- ---------- ----------
AADN03019346                 ---------- ---------- ---------- ---------- ---------- ---------- ---------- ---------- ---------- ---------- ----------
AADN03001776                 ---------- ---------- ---------- ---------- ---------- ---------- ---------- ---------- ---------- ---------- ----------
DQ018756                     ---------- ---------- ---------- ---------- ---------- ---------- ---------- ---------- ---------- ---------- ----------
AF173612                     ---------- ---------- ---------- ---------- ---------- ---------- ---------- ---------- ---------- ---------- ----------
Gallus_gallus_KT445934.2     GCTGCGATCT ATTGAGAGTC AGCCCTCGAC ACAAGCTTTT GTCGGAGCGC GGAGCGCGCG CGCGCGCGCG CGTGGCGGCG CCCCGGCGCG GGGCCGGGTC CGGCGGGCCA

                             1111111111 1111111111 1111111111 1111111111 1111111111 1111111111 1111111111 1111111111 1111111111 1111111111 1111111111
                             1111111111 1111111111 1111111111 1111111111 1111111111 1111111111 1111111111 1111111111 1111111111 1111111111 1111111111
                             6666666666 6666666666 6666666666 6666666667 7777777777 7777777777 7777777777 7777777777 7777777777 7777777777 7777777777
                             6666666667 7777777778 8888888889 9999999990 0000000001 1111111112 2222222223 3333333334 4444444445 5555555556 6666666667
                             1234567890 1234567890 1234567890 1234567890 1234567890 1234567890 1234567890 1234567890 1234567890 1234567890 1234567890
AADN03001677                 ---------- ---------- ---------- ---------- ---------- ---------- ---------- ---------- ---------- ---------- ----------
AADN03001778                 ---------- ---------- ---------- ---------- ---------- ---------- ---------- ---------- ---------- ---------- ----------
AADN03001785                 ---------- ---------- ---------- ---------- ---------- ---------- ---------- ---------- ---------- ---------- ----------
AADN03001786                 ---------- ---------- ---------- ---------- ---------- ---------- ---------- ---------- ---------- ---------- ----------
AADN03001788                 ---------- ---------- ---------- ---------- ---------- ---------- ---------- ---------- ---------- ---------- ----------
AADN03014081                 ---------- ---------- ---------- ---------- ---------- ---------- ---------- ---------- ---------- ---------- ----------
AADN03015064                 ---------- ---------- ---------- ---------- ---------- ---------- ---------- ---------- ---------- ---------- ----------
AADN03026634                 ---------- ---------- ---------- ---------- ---------- ---------- ---------- ---------- ---------- ---------- ----------
NW_003775878                 ---------- ---------- ---------- ---------- ---------- ---------- ---------- ---------- ---------- ---------- ----------
DQ018752                     ---------- ---------- ---------- ---------- ---------- ---------- ---------- ---------- ---------- ---------- ----------
DQ018757                     ---------- ---------- ---------- ---------- ---------- ---------- ---------- ---------- ---------- ---------- ----------
DQ112354                     ---------- ---------- ---------- ---------- ---------- ---------- ---------- ---------- ---------- ---------- ----------
FM165414                     ---------- ---------- ---------- ---------- ---------- ---------- ---------- ---------- ---------- ---------- ----------
FM165415                     ---------- ---------- ---------- ---------- ---------- ---------- ---------- ---------- ---------- ---------- ----------
AADN03000430                 ---------- ---------- ---------- ---------- ---------- ---------- ---------- ---------- ---------- ---------- ----------
AADN03001784                 ---------- ---------- ---------- ---------- ---------- ---------- ---------- ---------- ---------- ---------- ----------
AADN03001783                 ---------- ---------- ---------- ---------- ---------- ---------- ---------- ---------- ---------- ---------- ----------
DQ018754                     ---------- ---------- ---------- ---------- ---------- ---------- ---------- ---------- ---------- ---------- ----------
AADN03001782                 ---------- ---------- ---------- ---------- ---------- ---------- ---------- ---------- ---------- ---------- ----------
JN639848                     ---------- ---------- ---------- ---------- ---------- ---------- ---------- ---------- ---------- ---------- ----------
AADN03001670                 ---------- ---------- ---------- ---------- ---------- ---------- ---------- ---------- ---------- ---------- ----------
HQ873432                     ---------- ---------- ---------- ---------- ---------- ---------- ---------- ---------- ---------- ---------- ----------
AADN03001774                 ---------- ---------- ---------- ---------- ---------- ---------- ---------- ---------- ---------- ---------- ----------
AADN03001775                 ---------- ---------- ---------- ---------- ---------- ---------- ---------- ---------- ---------- ---------- ----------
EF552813                     ---------- ---------- ---------- ---------- ---------- ---------- ---------- ---------- ---------- ---------- ----------
AADN03022685                 ---------- ---------- ---------- ---------- ---------- ---------- ---------- ---------- ---------- ---------- ----------
AADN03019346                 ---------- ---------- ---------- ---------- ---------- ---------- ---------- ---------- ---------- ---------- ----------
```

```
AADN03001776                   ---------- ---------- ---------- ---------- ---------- ---------- ---------- ---------- ---------- ---------- ----------
DQ018756                       ---------- ---------- ---------- ---------- ---------- ---------- ---------- ---------- ---------- ---------- ----------
AF173612                       ---------- ---------- ---------- ---------- ---------- ---------- ---------- ---------- ---------- ---------- ----------
Gallus_gallus_KT445934.2       GTCGGTCGGC TCCCCGCGCC GCTCCGTTTG TTCCTGGGTT CGTTCGTTCG TTCGTTCCTT CCTTCCCCGG CCCCGCGCCG GCGCCGGCGC GGGGTTGGAA AGAGGGGGAG

                               1111111111 1111111111 1111111111 1111111111 1111111111 1111111111 1111111111 1111111111 1111111111 1111111111 1111111111
                               1111111111 1111111111 1111111111 1111111111 1111111111 1111111111 1111111111 1111111111 1111111111 1111111111 1111111111
                               7777777777 7777777777 7777777778 8888888888 8888888888 8888888888 8888888888 8888888888 8888888888 8888888888 8888888888
                               7777777778 8888888889 9999999990 0000000001 1111111112 2222222223 3333333334 4444444445 5555555556 6666666667 7777777778
                               1234567890 1234567890 1234567890 1234567890 1234567890 1234567890 1234567890 1234567890 1234567890 1234567890 1234567890
AADN03001677                   ---------- ---------- ---------- ---------- ---------- ---------- ---------- ---------- ---------- ---------- ----------
AADN03001778                   ---------- ---------- ---------- ---------- ---------- ---------- ---------- ---------- ---------- ---------- ----------
AADN03001785                   ---------- ---------- ---------- ---------- ---------- ---------- ---------- ---------- ---------- ---------- ----------
AADN03001786                   ---------- ---------- ---------- ---------- ---------- ---------- ---------- ---------- ---------- ---------- ----------
AADN03001788                   ---------- ---------- ---------- ---------- ---------- ---------- ---------- ---------- ---------- ---------- ----------
AADN03014081                   ---------- ---------- ---------- ---------- ---------- ---------- ---------- ---------- ---------- ---------- ----------
AADN03015064                   ---------- ---------- ---------- ---------- ---------- ---------- ---------- ---------- ---------- ---------- ----------
AADN03026634                   ---------- ---------- ---------- ---------- ---------- ---------- ---------- ---------- ---------- ---------- ----------
NW_003775878                   ---------- ---------- ---------- ---------- ---------- ---------- ---------- ---------- ---------- ---------- ----------
DQ018752                       ---------- ---------- ---------- ---------- ---------- ---------- ---------- ---------- ---------- ---------- ----------
DQ018757                       ---------- ---------- ---------- ---------- ---------- ---------- ---------- ---------- ---------- ---------- ----------
DQ112354                       ---------- ---------- ---------- ---------- ---------- ---------- ---------- ---------- ---------- ---------- ----------
FM165414                       ---------- ---------- ---------- ---------- ---------- ---------- ---------- ---------- ---------- ---------- ----------
FM165415                       ---------- ---------- ---------- ---------- ---------- ---------- ---------- ---------- ---------- ---------- ----------
AADN03000430                   ---------- ---------- ---------- ---------- ---------- ---------- ---------- ---------- ---------- ---------- ----------
AADN03001784                   ---------- ---------- ---------- ---------- ---------- ---------- ---------- ---------- ---------- ---------- ----------
AADN03001783                   ---------- ---------- ---------- ---------- ---------- ---------- ---------- ---------- ---------- ---------- ----------
DQ018754                       ---------- ---------- ---------- ---------- ---------- ---------- ---------- ---------- ---------- ---------- ----------
AADN03001782                   ---------- ---------- ---------- ---------- ---------- ---------- ---------- ---------- ---------- ---------- ----------
JN639848                       ---------- ---------- ---------- ---------- ---------- ---------- ---------- ---------- ---------- ---------- ----------
AADN03001670                   ---------- ---------- ---------- ---------- ---------- ---------- ---------- ---------- ---------- ---------- ----------
HQ873432                       ---------- ---------- ---------- ---------- ---------- ---------- ---------- ---------- ---------- ---------- ----------
AADN03001774                   ---------- ---------- ---------- ---------- ---------- ---------- ---------- ---------- ---------- ---------- ----------
AADN03001775                   ---------- ---------- ---------- ---------- ---------- ---------- ---------- ---------- ---------- ---------- ----------
EF552813                       ---------- ---------- ---------- ---------- ---------- ---------- ---------- ---------- ---------- ---------- ----------
AADN03022685                   ---------- ---------- ---------- ---------- ---------- ---------- ---------- ---------- ---------- ---------- ----------
AADN03019346                   ---------- ---------- ---------- ---------- ---------- ---------- ---------- ---------- ---------- ---------- ----------
AADN03001776                   ---------- ---------- ---------- ---------- ---------- ---------- ---------- ---------- ---------- ---------- ----------
DQ018756                       ---------- ---------- ---------- ---------- ---------- ---------- ---------- ---------- ---------- ---------- ----------
AF173612                       ---------- ---------- ---------- ---------- ---------- ---------- ---------- ---------- ---------- ---------- ----------
Gallus_gallus_KT445934.2       AGGGGCGGGG GGCGCGGCCG GCCCCCTTCC CCGTTTCCGT CCCCGCGGCG CGTGCCGTGG GACGGGCTCC CTCCGTTTTA CCCGAGCCCG GGGGTTGACC TGGCGGCCGG

                               1111111111 1111111111 1111111111 1111111111 1111111111 1111111111 1111111111 1111111111 1111111111 1111111111 1111111111
                               1111111111 1111111111 1111111111 1111111111 1111111111 1111111111 1111111111 1111111111 1111111111 1111111111 1111111111
                               8888888888 8888888889 9999999999 9999999999 9999999999 9999999999 9999999999 9999999999 9999999999 9999999999 9999999999
                               8888888889 9999999990 0000000001 1111111112 2222222223 3333333334 4444444445 5555555556 6666666667 7777777778 8888888889
                               1234567890 1234567890 1234567890 1234567890 1234567890 1234567890 1234567890 1234567890 1234567890 1234567890 1234567890
AADN03001677                   ---------- ---------- ---------- ---------- ---------- ---------- ---------- ---------- ---------- ---------- ----------
AADN03001778                   ---------- ---------- ---------- ---------- ---------- ---------- ---------- ---------- ---------- ---------- ----------
AADN03001785                   ---------- ---------- ---------- ---------- ---------- ---------- ---------- ---------- ---------- ---------- ----------
AADN03001786                   ---------- ---------- ---------- ---------- ---------- ---------- ---------- ---------- ---------- ---------- ----------
AADN03001788                   ---------- ---------- ---------- ---------- ---------- ---------- ---------- ---------- ---------- ---------- ----------
AADN03014081                   ---------- ---------- ---------- ---------- ---------- ---------- ---------- ---------- ---------- ---------- ----------
AADN03015064                   ---------- ---------- ---------- ---------- ---------- ---------- ---------- ---------- ---------- ---------- ----------
AADN03026634                   ---------- ---------- ---------- ---------- ---------- ---------- ---------- ---------- ---------- ---------- ----------
```

```
NW_003775878               ---------- ---------- ---------- ---------- ---------- ---------- ---------- ---------- ---------- ---------- ----------
DQ018752                   ---------- ---------- ---------- ---------- ---------- ---------- ---------- ---------- ---------- ---------- ----------
DQ018757                   ---------- ---------- ---------- ---------- ---------- ---------- ---------- ---------- ---------- ---------- ----------
DQ112354                   ---------- ---------- ---------- ---------- ---------- ---------- ---------- ---------- ---------- ---------- ----------
FM165414                   ---------- ---------- ---------- ---------- ---------- ---------- ---------- ---------- ---------- ---------- ----------
FM165415                   ---------- ---------- ---------- ---------- ---------- ---------- ---------- ---------- ---------- ---------- ----------
AADN03000430               ---------- ---------- ---------- ---------- ---------- ---------- ---------- ---------- ---------- ---------- ----------
AADN03001784               ---------- ---------- ---------- ---------- ---------- ---------- ---------- ---------- ---------- ---------- ----------
AADN03001783               ---------- ---------- ---------- ---------- ---------- ---------- ---------- ---------- ---------- ---------- ----------
DQ018754                   ---------- ---------- ---------- ---------- ---------- ---------- ---------- ---------- ---------- ---------- ----------
AADN03001782               ---------- ---------- ---------- ---------- ---------- ---------- ---------- ---------- ---------- ---------- ----------
JN639848                   ---------- ---------- ---------- ---------- ---------- ---------- ---------- ---------- ---------- ---------- ----------
AADN03001670               ---------- ---------- ---------- ---------- ---------- ---------- ---------- ---------- ---------- ---------- ----------
HQ873432                   ---------- ---------- ---------- ---------- ---------- ---------- ---------- ---------- ---------- ---------- ----------
AADN03001774               ---------- ---------- ---------- ---------- ---------- ---------- ---------- ---------- ---------- ---------- ----------
AADN03001775               ---------- ---------- ---------- ---------- ---------- ---------- ---------- ---------- ---------- ---------- ----------
EF552813                   ---------- ---------- ---------- ---------- ---------- ---------- ---------- ---------- ---------- ---------- ----------
AADN03022685               ---------- ---------- ---------- ---------- ---------- ---------- ---------- ---------- ---------- ---------- ----------
AADN03019346               ---------- ---------- ---------- ---------- ---------- ---------- ---------- ---------- ---------- ---------- ----------
AADN03001776               ---------- ---------- ---------- ---------- ---------- ---------- ---------- ---------- ---------- ---------- ----------
DQ018756                   ---------- ---------- ---------- ---------- ---------- ---------- ---------- ---------- ---------- ---------- ----------
AF173612                   ---------- ---------- ---------- ---------- ---------- ---------- ---------- ---------- ---------- ---------- ----------
Gallus_gallus_KT445934.2   GCGGCCGGGC TAGGGGGCGC TCCGCGTCCC CCTTCGGGGG GTTGACCTGT CGGGCGTTTT TTTTTTATTT TTTTCTCCCT AGGCGGGTCC GGGGGTAGCC CTGTCGGCCG

                           1111111111 1111111111 1111111111 1111111111 1111111111 1111111111 1111111111 1111111
                           1111111112 2222222222 2222222222 2222222222 2222222222 2222222222 2222222222 2222222
                           9999999990 0000000000 0000000000 0000000000 0000000000 0000000000 0000000000 0000000
                           9999999990 0000000001 1111111112 2222222223 3333333334 4444444445 5555555556 6666666
                           1234567890 1234567890 1234567890 1234567890 1234567890 1234567890 1234567890 1234567
AADN03001677               ---------- ---------- ---------- ---------- ---------- ---------- ---------- -------
AADN03001778               ---------- ---------- ---------- ---------- ---------- ---------- ---------- -------
AADN03001785               ---------- ---------- ---------- ---------- ---------- ---------- ---------- -------
AADN03001786               ---------- ---------- ---------- ---------- ---------- ---------- ---------- -------
AADN03001788               ---------- ---------- ---------- ---------- ---------- ---------- ---------- -------
AADN03014081               ---------- ---------- ---------- ---------- ---------- ---------- ---------- -------
AADN03015064               ---------- ---------- ---------- ---------- ---------- ---------- ---------- -------
AADN03026634               ---------- ---------- ---------- ---------- ---------- ---------- ---------- -------
NW_003775878               ---------- ---------- ---------- ---------- ---------- ---------- ---------- -------
DQ018752                   ---------- ---------- ---------- ---------- ---------- ---------- ---------- -------
DQ018757                   ---------- ---------- ---------- ---------- ---------- ---------- ---------- -------
DQ112354                   ---------- ---------- ---------- ---------- ---------- ---------- ---------- -------
FM165414                   ---------- ---------- ---------- ---------- ---------- ---------- ---------- -------
FM165415                   ---------- ---------- ---------- ---------- ---------- ---------- ---------- -------
AADN03000430               ---------- ---------- ---------- ---------- ---------- ---------- ---------- -------
AADN03001784               ---------- ---------- ---------- ---------- ---------- ---------- ---------- -------
AADN03001783               ---------- ---------- ---------- ---------- ---------- ---------- ---------- -------
DQ018754                   ---------- ---------- ---------- ---------- ---------- ---------- ---------- -------
AADN03001782               ---------- ---------- ---------- ---------- ---------- ---------- ---------- -------
JN639848                   ---------- ---------- ---------- ---------- ---------- ---------- ---------- -------
AADN03001670               ---------- ---------- ---------- ---------- ---------- ---------- ---------- -------
HQ873432                   ---------- ---------- ---------- ---------- ---------- ---------- ---------- -------
AADN03001774               ---------- ---------- ---------- ---------- ---------- ---------- ---------- -------
AADN03001775               ---------- ---------- ---------- ---------- ---------- ---------- ---------- -------
EF552813                   ---------- ---------- ---------- ---------- ---------- ---------- ---------- -------
AADN03022685               ---------- ---------- ---------- ---------- ---------- ---------- ---------- -------
```

```
AADN03019346              ---------- ---------- ---------- ---------- ---------- ---------- ---------- -------
AADN03001776              ---------- ---------- ---------- ---------- ---------- ---------- ---------- -------
DQ018756                  ---------- ---------- ---------- ---------- ---------- ---------- ---------- -------
AF173612                  ---------- ---------- ---------- ---------- ---------- ---------- ---------- -------
Gallus_gallus_KT445934.2  CCCGGCCCGG CCCAGCACGC CCCCCCGCCG GCAAGTGGCT GCGGTGCCGA GGTGGCGGGT AGACCTGGCG GCCGGCA
```
