# Supplementary material for: Chicken rRNA Gene Cluster Structure
Source: PLoS One. 2016 Jun 14;11(6):e0157464. doi: 10.1371/journal.pone.0157464 (PMC4907446; doi:10.1371/journal.pone.0157464)
Supplement: S2 Table — (PDF) [file pone.0157464.s011.pdf]

S2 Table

Multiple alignment matrix for chicken rRNA gene cluster boundaries search using annotated fragments of ribosomal clusters of *Homo sapiens, Rattus norvegicus, Mus musculus, Xenopus laevis* and *Crocodylus porosus*

```
                                                                                                                                1 1111111111
                                           1 1111111112 2222222223 3333333334 4444444445 5555555556 6666666667 7777777778 8888888889 9999999990 0000000001
                               1234567890 1234567890 1234567890 1234567890 1234567890 1234567890 1234567890 1234567890 1234567890 1234567890 1234567890
Crocodylus_porosus_EU727191    ---------- ---------- ---------- ---------- ---------- ---------- ---------- ---------- ---------- ---------- ----------
Xenopus_laevis_X02995          ---------- ---------- ---------- ---------- ---------- ---------- ---------- ---------- ---------- ---------- ----------
Mus_musculus_NR_046233         ACTGACACGC TGTCCTTTCC CTATTAACAC TAAAGGACAC TATAAAGAGA CCCTTTCGAT TTAAGGCTGT TTTGCTTGTC CAGCCTATTC TTTTTACTGG CTTGGGTCTG
Homo_sapiens_U13369            ---------- ---------- ---------- ---------- ---------- ---------- ---------- ---------- ---------- ---------- ----------
Rattus_norvegicus_NR_046239    ---------- ---------- ---------- ---------- ---------- ---------- ---------- ---------- ---------- ---------- ----------
Gallus_gallus_KT445934.2       ---------- ---------- ---------- ---------- ---------- ---------- ---------- ---------- ---------- ---------- ----------

                               1111111111 1111111111 1111111111 1111111111 1111111111 1111111111 1111111111 1111111111 1111111112 2222222222 2222222222
                               1111111112 2222222223 3333333334 4444444445 5555555556 6666666667 7777777778 8888888889 9999999990 0000000001 1111111112
                               1234567890 1234567890 1234567890 1234567890 1234567890 1234567890 1234567890 1234567890 1234567890 1234567890 1234567890
Crocodylus_porosus_EU727191    ---------- ---------- ---------- ---------- ---------- ---------- ---------- ---------- ---------- ---------- ----------
Xenopus_laevis_X02995          ---------- ---------- ---------- ---------- ---------- ---------- ---------- ---------- ---------- ---------- ----------
Mus_musculus_NR_046233         TCGCGGTGCC TGAAGCTGTC CCCGAGCCAC GCTTCCTGCT TTCCCGGGCT TGCTGCTTGC GTGTGCTTGC TGTGGGCAGC TTGTGACAAC TGGGCGCTGT GACTTTGCTG
Homo_sapiens_U13369            ---------- ---------- ---------- ---------- ---------- ---------- ---------- ---------- ---------- ---------- ----------
Rattus_norvegicus_NR_046239    ---------- ---------- ---------- ---------- ---------- ---------- ---------- ---------- ---------- ---------- ----------
Gallus_gallus_KT445934.2       ---------- ---------- ---------- ---------- ---------- ---------- ---------- ---------- ---------- ---------- ----------

                               2222222222 2222222222 2222222222 2222222222 2222222222 2222222222 2222222222 2222222223 3333333333 3333333333 3333333333
                               2222222223 3333333334 4444444445 5555555556 6666666667 7777777778 8888888889 9999999990 0000000001 1111111112 2222222223
                               1234567890 1234567890 1234567890 1234567890 1234567890 1234567890 1234567890 1234567890 1234567890 1234567890 1234567890
Crocodylus_porosus_EU727191    ---------- ---------- ---------- ---------- ---------- ---------- ---------- ---------- ---------- ---------- ----------
Xenopus_laevis_X02995          ---------- ---------- ---------- ---------- ---------- ---------- ---------- ---------- ---------- ---------- ----------
Mus_musculus_NR_046233         CGTGTCAGAC GTTTTTCCCG ATTTCCCCGA GGTGTCGTTG TCACACCTGT CCCGGTTGGA ATGGTGGAGC CAGCTGTGGT TGAGGGCCAC CTTATTTCGG CTCACTTTTT
Homo_sapiens_U13369            ---------- ---------- ---------- ---------- ---------- ---------- ---------- ---------- ---------- ---------- ----------
Rattus_norvegicus_NR_046239    ---------- ---------- ---------- ---------- ---------- ---------- ---------- ---------- ---------- ---------- ----------
Gallus_gallus_KT445934.2       ---------- ---------- ---------- ---------- ---------- ---------- ---------- ---------- ---------- ---------- ----------

                               3333333333 3333333333 3333333333 3333333333 3333333333 3333333333 3333333334 4444444444 4444444444 4444444444 4444444444
                               3333333334 4444444445 5555555556 6666666667 7777777778 8888888889 9999999990 0000000001 1111111112 2222222223 3333333334
                               1234567890 1234567890 1234567890 1234567890 1234567890 1234567890 1234567890 1234567890 1234567890 1234567890 1234567890
Crocodylus_porosus_EU727191    ---------- ---------- ---------- ---------- ---------- ---------- ---------- ---------- ---------- ---------- ----------
Xenopus_laevis_X02995          ---------- ---------- ---------- ---------- ---------- ---------- ---------- ---------- ---------- ---------- ----------
Mus_musculus_NR_046233         TTTTTTTTTT TTCTCTTGGA GTCCCGAACC TCCGCTCTTT TCTCTTCCCG GTCTTTCTTC CACATGCCTC CCGAGTGCAT TTCTTTTTGT TTTTTTTCTT TTTTTTTTTT
Homo_sapiens_U13369            ---------- ---------- GCTGACACGC TGTCCTCTGG CGACCTGTCG TCGGAGAGGT TGGGCCTCCG GATGCGCGCG GGGCTCTGGC CTCACGGTGA CCGGCTAGCC
Rattus_norvegicus_NR_046239    ---------- ---------- ---------- ---------- ---------- ---------- ---------- ---------- ---------- ---------- ----------
Gallus_gallus_KT445934.2       ---------- ---------- ---------- ---------- ---------- ---------- ---------- ---------- ---------- ---------- ----------

                               4444444444 4444444444 4444444444 4444444444 4444444444 4444444445 5555555555 5555555555 5555555555 5555555555 5555555555
                               4444444445 5555555556 6666666667 7777777778 8888888889 9999999990 0000000001 1111111112 2222222223 3333333334 4444444445
                               1234567890 1234567890 1234567890 1234567890 1234567890 1234567890 1234567890 1234567890 1234567890 1234567890 1234567890
Crocodylus_porosus_EU727191    ---------- ---------- ---------- ---------- ---------- ---------- ---------- ---------- ---------- ---------- ----------
Xenopus_laevis_X02995          ---------- ---------- ---------- ---------- ---------- ---------- ---------- ---------- ---------- ---------- ----------
Mus_musculus_NR_046233         TTTTTTTGGG GAGGTGGAGA GTCCCGAGTA CTTCACTCCT GTCTGTGGTG TCCAAGTGTT CATGCCACGT GCCTCCCGAG TGCACTTTTT TTTGTGGCAG TCGCTCGTTG
Homo_sapiens_U13369            GGCCGCGCTC CTGCCTTGAG CCGCCTGCCG CGGCCCGCGG GCCTGCTGTT CTCTCGCGCG TCCGAGCGTC CCGACTCCCG GTGCCGGCCC GGGTCCGGGT CTCTGACCCA
Rattus_norvegicus_NR_046239    ---------- ---------- ---------- ---------- ---------- ---------- ---------- ---------- ---------- ---------- ----------
```

```
Gallus_gallus_KT445934.2           ---------- ---------- ---------- ---------- ---------- ---------- ---------- ---------- ---------- ---------- ----------

                                   5555555555 5555555555 5555555555 5555555555 5555555556 6666666666 6666666666 6666666666 6666666666 6666666666 6666666666
                                   5555555556 6666666667 7777777778 8888888889 9999999990 0000000001 1111111112 2222222223 3333333334 4444444445 5555555556
                                   1234567890 1234567890 1234567890 1234567890 1234567890 1234567890 1234567890 1234567890 1234567890 1234567890 1234567890
Crocodylus_porosus_EU727191        ---------- ---------- ---------- ---------- ---------- ---------- ---------- ---------- ---------- ---------- ----------
Xenopus_laevis_X02995              ---------- ---------- ---------- ---------- ---------- ---------- ---------- ---------- ---------- ---------- ----------
Mus_musculus_NR_046233             TGTTCTCTTG TTCTGTGTCT GCCCGTATCA GTAACTGTCT TGCCCCGCGT GTAAGACATT CCTATCTCGC TTGTTTCTCC CGATTGCGCG TCGTTGCTCA CTCTTAGATC
Homo_sapiens_U13369                CCCGGGGGCG GCGGGGAAGG CGGCGAGGGC CACCGTGCCC CGTGCGCTCT CCGCTGCGGG CGCCCGGGGC GCCGCACAAC CCCACCCGCT GGCTCCGTGC CGTGCGTGTC
Rattus_norvegicus_NR_046239        ---------- ---------- ---------- ---------- ---------- ---------- ---------- ---------- ---------- ---------- ----------
Gallus_gallus_KT445934.2           ---------- ---------- ---------- ---------- ---------- ---------- ---------- ---------- ---------- ---------- ----------

                                   6666666666 6666666666 6666666666 6666666667 7777777777 7777777777 7777777777 7777777777 7777777777 7777777777 7777777777
                                   6666666667 7777777778 8888888889 9999999990 0000000001 1111111112 2222222223 3333333334 4444444445 5555555556 6666666667
                                   1234567890 1234567890 1234567890 1234567890 1234567890 1234567890 1234567890 1234567890 1234567890 1234567890 1234567890
Crocodylus_porosus_EU727191        ---------- ---------- ---------- ---------- ---------- ---------- ---------- ---------- ---------- ---------- ----------
Xenopus_laevis_X02995              ---------- ---------- ---------- ---------- ---------- ---------- ---------- ---------- ---------- ---------- ----------
Mus_musculus_NR_046233             GATGTGGTGC TCCGGAGTTC TCTTCGGGCC AGGGCCAAGC CGCGCCAGGC GAGGGACGGA CATTCATGGC GAATGGCGGC CGCTCTTCTC GTTCTGCCAG CGGGCCCTCG
Homo_sapiens_U13369                AGGCGTTCTC GTCTCCGCGG GGTTGTCCGC CGCCCCTTCC CCGGAGTGGG GGGTGGCCGG AGCCGATCGG CTCGCTGGCC GGCCGGCCTC CGCTCCCGGG GGGCTCTTCG
Rattus_norvegicus_NR_046239        ---------- ---------- ---------- ---------- ---------- ---------- ---------- ---------- ---------- ---------- ----------
Gallus_gallus_KT445934.2           ---------- ---------- ---------- ---------- ---------- ---------- ---------- ---------- ---------- ---------- ----------

                                   7777777777 7777777777 7777777778 8888888888 8888888888 8888888888 8888888888 8888888888 8888888888 8888888888 8888888888
                                   7777777778 8888888889 9999999990 0000000001 1111111112 2222222223 3333333334 4444444445 5555555556 6666666667 7777777778
                                   1234567890 1234567890 1234567890 1234567890 1234567890 1234567890 1234567890 1234567890 1234567890 1234567890 1234567890
Crocodylus_porosus_EU727191        ---------- ---------- ---------- ---------- ---------- ---------- ---------- ---------- ---------- ---------- ----------
Xenopus_laevis_X02995              ---------- ---------- ---------- ---------- ---------- ---------- ---------- ---------- ---------- ---------- ----------
Mus_musculus_NR_046233             TCTCTCCACC CCATCCGTCT GCCGGTGGTG TGTGGAAGGC AGGGGTGCGG CTCTCCGGCC CGACGCTGCC CCGCGCGCAC TTTTCTCAGT GGTTCGCGTG GTCCTTGTGG
Homo_sapiens_U13369                ATCGATGTGG TGACGTCGTG CTCTCCCGGG CCGGGTCCGA GCCGCGACGG GCGAGGGGCG GACGTTCGTG GCGAACGGGA CCGTCCTTCT CGCTCCGCCC GCGCGGTCCC
Rattus_norvegicus_NR_046239        ---------- ---------- ---------- ---------- ---------- ---------- ---------- ---------- ---------- ---------- ----------
Gallus_gallus_KT445934.2           ---------- ---------- ---------- ---------- ---------- ---------- ---------- ---------- ---------- ---------- ----------

                                   8888888888 8888888889 9999999999 9999999999 9999999999 9999999999 9999999999 9999999999 9999999999 9999999999 9999999999
                                   8888888889 9999999990 0000000001 1111111112 2222222223 3333333334 4444444445 5555555556 6666666667 7777777778 8888888889
                                   1234567890 1234567890 1234567890 1234567890 1234567890 1234567890 1234567890 1234567890 1234567890 1234567890 1234567890
Crocodylus_porosus_EU727191        ---------- ---------- ---------- ---------- ---------- ---------- ---------- ---------- ---------- ---------- ----------
Xenopus_laevis_X02995              ---------- ---------- ---------- ---------- ---------- ---------- ---------- ---------- ---------- ---------- ----------
Mus_musculus_NR_046233             ATGTGTGAGG CGCCCGGTTG TGCCCTCACG TGTTTCACTT TGGTCGTGTC TCGCTTGACC ATGTTCCCAG AGTCGGTGGA TGTGGCCGGT GGCGTTGCAT ACCCTTCCCG
Homo_sapiens_U13369                CTCGTCTGCT CCTCTCCCCG CCCGCCGGCC GGCGTGTGGG AAGGCGTGGG GTGCGGACCC CGGCCCGACC TCGCCGTCCC GCCCGCCGCC TTCGCTTCGC GGGTGCGGGC
Rattus_norvegicus_NR_046239        ---------- ---------- ---------- ---------- ---------- ---------- ---------- ---------- ---------- ---------- ----------
Gallus_gallus_KT445934.2           ---------- ---------- ---------- ---------- ---------- ---------- ---------- ---------- ---------- ---------- ----------

                                            1 1111111111 1111111111 1111111111 1111111111 1111111111 1111111111 1111111111 1111111111 1111111111 1111111111
                                   9999999990 0000000000 0000000000 0000000000 0000000000 0000000000 0000000000 0000000000 0000000000 0000000000 0000000001
                                   9999999990 0000000001 1111111112 2222222223 3333333334 4444444445 5555555556 6666666667 7777777778 8888888889 9999999990
                                   1234567890 1234567890 1234567890 1234567890 1234567890 1234567890 1234567890 1234567890 1234567890 1234567890 1234567890
Crocodylus_porosus_EU727191        ---------- ---------- ---------- ---------- ---------- ---------- ---------- ---------- ---------- ---------- ----------
Xenopus_laevis_X02995              ---------- ---------- ---------- ---------- ---------- ---------- ---------- ---------- ---------- ---------- ----------
Mus_musculus_NR_046233             TCTGGTGTGT GCACGCGCTG TTTCTTGTAA GCGTCGAGGT GCTCCTGGAG CGTTCCAGGT TTGTCTCCTA GGTGCCTGCT TCTGAGCTGG TGGTGGCGCT CCCCATTCCC
Homo_sapiens_U13369                CGGCGGGGTC CTCTGACGCG GCAGACAGCC CTGCCTGTCG CCTCCAGTGG TTGTCGACTT GCGGGCGGCC CCCCTCCGCG GCGGTGGGGG TGCCGTCCCG CCGGCCCGTC
Rattus_norvegicus_NR_046239        ---------- ---------- ---------- ---------- ---------- ---------- ---------- ---------- ---------- ---------- ----------
Gallus_gallus_KT445934.2           ---------- ---------- ---------- ---------- ---------- ---------- ---------- ---------- ---------- ---------- ----------
```

```
                              1111111111 1111111111 1111111111 1111111111 1111111111 1111111111 1111111111 1111111111 1111111111 1111111111 1111111111
                              1111111111 1111111111 1111111111 1111111111 1111111111 1111111111 1111111111 1111111111 1111111111 1111111112 2222222222
                              0000000001 1111111112 2222222223 3333333334 4444444445 5555555556 6666666667 7777777778 8888888889 9999999990 0000000001
                              1234567890 1234567890 1234567890 1234567890 1234567890 1234567890 1234567890 1234567890 1234567890 1234567890 1234567890
Crocodylus_porosus_EU727191   ---------- ---------- ---------- ---------- ---------- ---------- ---------- ---------- ---------- ---------- ----------
Xenopus_laevis_X02995         ---------- ---------- ---------- ---------- ---------- ---------- ---------- ---------- ---------- ---------- ----------
Mus_musculus_NR_046233        TGGTGTGCCT CCGGTGCTCC GTCTGGCTGT GTGCCTTCCC GTTTGTGTCT GAGAAGCCCG TGAGAGGGGG GTCGAGGAGA GAAGGAGGGG CAAGACCCCC CTTCTTCGTC
Homo_sapiens_U13369           GTGCTGCCCT CTCGGGGGGG GTTTGCGCGA GCGTCGGCTC CGCCTGGGCC CTTGCGGTGC TCCTGGAGCG CTCCGGGTTG TCCCTCAGGT GCCCGAGGCC GAACGGTGGT
Rattus_norvegicus_NR_046239   ---------- ---------- ---------- ---------- ---------- ---------- ---------- ---------- ---------- ---------- ----------
Gallus_gallus_KT445934.2      ---------- ---------- ---------- ---------- ---------- ---------- ---------- ---------- ---------- ---------- ----------

                              1111111111 1111111111 1111111111 1111111111 1111111111 1111111111 1111111111 1111111111 1111111111 1111111111 1111111111
                              2222222222 2222222222 2222222222 2222222222 2222222222 2222222222 2222222222 2222222222 2222222223 3333333333 3333333333
                              1111111112 2222222223 3333333334 4444444445 5555555556 6666666667 7777777778 8888888889 9999999990 0000000001 1111111112
                              1234567890 1234567890 1234567890 1234567890 1234567890 1234567890 1234567890 1234567890 1234567890 1234567890 1234567890
Crocodylus_porosus_EU727191   ---------- ---------- ---------- ---------- ---------- ---------- ---------- ---------- ---------- ---------- ----------
Xenopus_laevis_X02995         ---------- ---------- ---------- ---------- ---------- ---------- ---------- ---------- ---------- ---------- ----------
Mus_musculus_NR_046233        GGGTGAGGCG CCCACCCCGC GACTAGTACG CCTGTGCGTA GGGCTGGTGC TGAGCGGTCG CGGCTGGGGT TGGAAAGTTT CTCGAGAGAC TCATTGCTTT CCCGTGGGGA
Homo_sapiens_U13369           GTGTCGTTCC CGCCCCCGGC GCCCCCTCCT CCGGTCGCCG CCGCGGTGTC CGCGCGTGGG TCCTGAGGGA GCTCGTCGGT GTGGGGTTCG AGGCGGTTTG AGTGAGACGA
Rattus_norvegicus_NR_046239   ---------- ---------- ---------- ---------- ---------- ---------- ---------- ---------- ---------- ---------- ----------
Gallus_gallus_KT445934.2      ---------- ---------- ---------- ---------- ---------- ---------- ---------- ---------- ---------- ---------- ----------

                              1111111111 1111111111 1111111111 1111111111 1111111111 1111111111 1111111111 1111111111 1111111111 1111111111 1111111111
                              3333333333 3333333333 3333333333 3333333333 3333333333 3333333333 3333333333 3333333334 4444444444 4444444444 4444444444
                              2222222223 3333333334 4444444445 5555555556 6666666667 7777777778 8888888889 9999999990 0000000001 1111111112 2222222223
                              1234567890 1234567890 1234567890 1234567890 1234567890 1234567890 1234567890 1234567890 1234567890 1234567890 1234567890
Crocodylus_porosus_EU727191   ---------- ---------- ---------- ---------- ---------- ---------- ---------- ---------- ---------- ---------- ----------
Xenopus_laevis_X02995         ---------- ---------- ---------- ---------- ---------- ---------- ---------- ---------- ---------- ---------- ----------
Mus_musculus_NR_046233        GCTTTGAGAG GCCTGGCTTT CGGGGGGGAC CGGTTGCAGG GTCTCCCCTG TCCGCGGATG CTCAGAATGC CCTTGGAAGA GAACCTTCCT GTTGCCGCAG ACCCCCCCGC
Homo_sapiens_U13369           GACGAGACGC GCCCCTCCCA CGCGGGGAAG GGCGCCCGCC TGCTCTCGGT GAGCGCACGT CCCGTGCTCC CCTCTGGCGG GTGCGCGCGG GCCGTGTGAG CGATCGCGGT
Rattus_norvegicus_NR_046239   ---------- ---------- ---------- ---------- ---------- ---------- ---------- ---------- ---------- ---------- ----------
Gallus_gallus_KT445934.2      ---------- ---------- ---------- ---------- ---------- ---------- ---------- ---------- ---------- ---------- ----------

                              1111111111 1111111111 1111111111 1111111111 1111111111 1111111111 1111111111 1111111111 1111111111 1111111111 1111111111
                              4444444444 4444444444 4444444444 4444444444 4444444444 4444444444 4444444445 5555555555 5555555555 5555555555 5555555555
                              3333333334 4444444445 5555555556 6666666667 7777777778 8888888889 9999999990 0000000001 1111111112 2222222223 3333333334
                              1234567890 1234567890 1234567890 1234567890 1234567890 1234567890 1234567890 1234567890 1234567890 1234567890 1234567890
Crocodylus_porosus_EU727191   ---------- ---------- ---------- ---------- ---------- ---------- ---------- ---------- ---------- ---------- ----------
Xenopus_laevis_X02995         ---------- ---------- ---------- ---------- ---------- ---------- ---------- ---------- ---------- ---------- ----------
Mus_musculus_NR_046233        GCGGTCGCCC GCGTGTTGGT CTTCTGGTTT CCCTGTGTGC TCGTCGCATG CATCCTCTCT CGGTGGCCGG GGCTCGTCGG GGTTTTGGGT CCGTCCCGCC CTCAGTGAGA
Homo_sapiens_U13369           GGGTTCGGGC CGGTGTGACG CGTGCGCCGG CCGGCCGCCG AGGGGCTGCC GTTCTGCCTC CGACCGGTCG TGTGTGGGTT GACTTCGGAG GCGCTCTGCC TCGGAAGGAA
Rattus_norvegicus_NR_046239   ---------- ---------- ---------- ---------- ---------- ---------- ---------- ---------- ---------- ---------- ----------
Gallus_gallus_KT445934.2      ---------- ---------- ---------- ---------- ---------- ---------- ---------- ---------- ---------- ---------- ----------

                              1111111111 1111111111 1111111111 1111111111 1111111111 1111111111 1111111111 1111111111 1111111111 1111111111 1111111111
                              5555555555 5555555555 5555555555 5555555555 5555555555 5555555556 6666666666 6666666666 6666666666 6666666666 6666666666
                              4444444445 5555555556 6666666667 7777777778 8888888889 9999999990 0000000001 1111111112 2222222223 3333333334 4444444445
                              1234567890 1234567890 1234567890 1234567890 1234567890 1234567890 1234567890 1234567890 1234567890 1234567890 1234567890
Crocodylus_porosus_EU727191   ---------- ---------- ---------- ---------- ---------- ---------- ---------- ---------- ---------- ---------- ----------
Xenopus_laevis_X02995         ---------- ---------- ---------- ---------- ---------- ---------- ---------- ---------- ---------- ---------- ----------
Mus_musculus_NR_046233        AAGTTTCCTT CTCTAGCTAT CTTCCGGAAA GGGTGCGGGC TTCTTACGGT CTCGAGGGGT CTCTCCCGAA TGGTCCCCTG GAGGGCTCGC CCCCTGACCG CCTCCCGCGC
Homo_sapiens_U13369           GGAGGTGGGT GGACGGGGGG GCCTGGTGGG GTTGCGCGCA CGCGCGCACC GGCCGGGCCC CCGCCCTGAA CGCGAACGCT CGAGGTGGCC GCGCGCAGGT GTTTCCTCGT
Rattus_norvegicus_NR_046239   ---------- ---------- ---------- ---------- ---------- ---------- ---------- ---------- ---------- ---------- ----------
Gallus_gallus_KT445934.2      ---------- ---------- ---------- ---------- ---------- ---------- ---------- ---------- ---------- ---------- ----------
```

```
                            1111111111 1111111111 1111111111 1111111111 1111111111 1111111111 1111111111 1111111111 1111111111 1111111111 1111111111
                            6666666666 6666666666 6666666666 6666666666 6666666667 7777777777 7777777777 7777777777 7777777777 7777777777 7777777777
                            5555555556 6666666667 7777777778 8888888889 9999999990 0000000001 1111111112 2222222223 3333333334 4444444445 5555555556
                            1234567890 1234567890 1234567890 1234567890 1234567890 1234567890 1234567890 1234567890 1234567890 1234567890 1234567890
Crocodylus_porosus_EU727191 ---------- ---------- ---------- ---------- ---------- ---------- ---------- ---------- ---------- ---------- ----------
Xenopus_laevis_X02995       ---------- ---------- ---------- ---------- ---------- ---------- ---------- ---------- ---------- ---------- ----------
Mus_musculus_NR_046233      GCGCAGCGTT TGCTCTCTCG TCTACCGCGG CCCGCGGCCT CCCCGCTCCG AGTTCGGGGA GGGATCACGC GGGGCAGAGC CTGTCTGTCG TCCTGCCGTT GCTGCGGAGC
Homo_sapiens_U13369         ACCGCAGGGC CCCCTCCCTT CCCCAGGCGT CCCTCGGCGC CTCTGCGGGC CCGAGGAGGA GCGGCTGGCG GGTGGGGGGA GTGTGACCCA CCCTCGGTGA GAAAAGCCTT
Rattus_norvegicus_NR_046239 ---------- ---------- ---------- ---------- ---------- ---------- ---------- ---------- ---------- ---------- ----------
Gallus_gallus_KT445934.2    ---------- ---------- ---------- ---------- ---------- ---------- ---------- ---------- ---------- ---------- ----------

                            1111111111 1111111111 1111111111 1111111111 1111111111 1111111111 1111111111 1111111111 1111111111 1111111111 1111111111
                            7777777777 7777777777 7777777777 7777777778 8888888888 8888888888 8888888888 8888888888 8888888888 8888888888 8888888888
                            6666666667 7777777778 8888888889 9999999990 0000000001 1111111112 2222222223 3333333334 4444444445 5555555556 6666666667
                            1234567890 1234567890 1234567890 1234567890 1234567890 1234567890 1234567890 1234567890 1234567890 1234567890 1234567890
Crocodylus_porosus_EU727191 ---------- ---------- ---------- ---------- ---------- ---------- ---------- ---------- ---------- ---------- ----------
Xenopus_laevis_X02995       ---------- ---------- ---------- ---------- ---------- ---------- ---------- ---------- ---------- ---------- ----------
Mus_musculus_NR_046233      ATGTGGCTCG GCTTGTGTGG TTGGTGGCTG GGGAGAGGGC TCCGTGCACA CCCCCGCGTG CGCGTACTTT CCTCCCCTCC TGAGGGCCGC CGTGCGGACG GGGTGTGGGT
Homo_sapiens_U13369         CTCTAGCGAT CTGAGAGGCG TGCCTTGGGG GTACCGGATC CCCCGGGCCG CCGCCTCTGT CTCTGCCTCC GTTATGGTAG CGCTGCCGTA GCGACCCGCT CGCAGAGGAC
Rattus_norvegicus_NR_046239 ---------- ---------- ---------- ---------- ---------- ---------- ---------- ---------- ---------- ---------- ----------
Gallus_gallus_KT445934.2    ---------- ---------- ---------- ---------- ---------- ---------- ---------- ---------- ---------- ---------- ----------

                            1111111111 1111111111 1111111111 1111111111 1111111111 1111111111 1111111111 1111111111 1111111111 1111111111 1111111111
                            8888888888 8888888888 8888888889 9999999999 9999999999 9999999999 9999999999 9999999999 9999999999 9999999999 9999999999
                            7777777778 8888888889 9999999990 0000000001 1111111112 2222222223 3333333334 4444444445 5555555556 6666666667 7777777778
                            1234567890 1234567890 1234567890 1234567890 1234567890 1234567890 1234567890 1234567890 1234567890 1234567890 1234567890
Crocodylus_porosus_EU727191 ---------- ---------- ---------- ---------- ---------- ---------- ---------- ---------- ---------- ---------- ----------
Xenopus_laevis_X02995       ---------- ---------- ---------- ---------- ---------- ---------- ---------- ---------- ---------- ---------- ----------
Mus_musculus_NR_046233      AGGCGACGGT GGGCTCCCGG GTCCCCACCC GTCTTCCCGT GCCTCACCCG TGCCTTCCGT CGCGTGCGTC CCTCTCGCTC GCGTCCACGA CTTTGGCCGC TCCCGCGACG
Homo_sapiens_U13369         CCTCCTCCGC TTCCCCCTCG ACGGGGTTGG GGGGGAGAAG CGAGGGTTCC GCCGGCCACC GCGGTGGTGG CCGAGTGCGG CTCGTCGCCT ACTGTGGCCC GCGCCTCCCC
Rattus_norvegicus_NR_046239 ---------- ---------- ---------- ---------- ---------- ---------- ---------- ---------- ---------- ---------- ----------
Gallus_gallus_KT445934.2    ---------- ---------- ---------- ---------- ---------- ---------- ---------- ---------- ---------- ---------- ----------

                            1111111111 1111111112 2222222222 2222222222 2222222222 2222222222 2222222222 2222222222 2222222222 2222222222 2222222222
                            9999999999 9999999990 0000000000 0000000000 0000000000 0000000000 0000000000 0000000000 0000000000 0000000000 0000000000
                            8888888889 9999999990 0000000001 1111111112 2222222223 3333333334 4444444445 5555555556 6666666667 7777777778 8888888889
                            1234567890 1234567890 1234567890 1234567890 1234567890 1234567890 1234567890 1234567890 1234567890 1234567890 1234567890
Crocodylus_porosus_EU727191 ---------- ---------- ---------- ---------- ---------- ---------- ---------- ---------- ---------- ---------- ----------
Xenopus_laevis_X02995       ---------- ---------- ---------- ---------- ---------- ---------- ---------- ---------- ---------- ---------- ----------
Mus_musculus_NR_046233      GCGGCCTGCG CCGCGCGTGG TGCGTGCTGT GTGCTTCTCG GGCTGTGTGG TTGTGTCGCC TCGCCCCCCC CTTCCCGCGG CAGCGTTCCC ACGGCTGGCG AAATCGCGGG
Homo_sapiens_U13369         CTTCCGAGTC GGGGGAGGAT CCCGCCGGGC CGGGCCCGGC GCTCCCACCC AGCGGGTTGG GACGCGGCGG CCGGCGGGCG GTGGGTGTGC GCGCCCGGCG CTCTGTCCGG
Rattus_norvegicus_NR_046239 ---------- ---------- ---------- ---------- ---------- ---------- ---------- ---------- ---------- ---------- ----------
Gallus_gallus_KT445934.2    ---------- ---------- ---------- ---------- ---------- ---------- ---------- ---------- ---------- ---------- ----------

                            2222222222 2222222222 2222222222 2222222222 2222222222 2222222222 2222222222 2222222222 2222222222 2222222222 2222222222
                            0000000001 1111111111 1111111111 1111111111 1111111111 1111111111 1111111111 1111111111 1111111111 1111111111 1111111112
                            9999999990 0000000001 1111111112 2222222223 3333333334 4444444445 5555555556 6666666667 7777777778 8888888889 9999999990
                            1234567890 1234567890 1234567890 1234567890 1234567890 1234567890 1234567890 1234567890 1234567890 1234567890 1234567890
Crocodylus_porosus_EU727191 ---------- ---------- ---------- ---------- ---------- ---------- ---------- ---------- ---------- ---------- ----------
Xenopus_laevis_X02995       ---------- ---------- ---------- ---------- ---------- ---------- ---------- ---------- ---------- ---------- ----------
Mus_musculus_NR_046233      AGTCCTCCTT CCCCTCCTCG GGGTCGAGAG GGTCCGTGTC TGGCGTTGAT TGATCTCGCT CTCGGGGACG GGACCGTTCT GTGGGAGAAC GGCTGTTGGC CGCGTCCGGC
Homo_sapiens_U13369         CGCGTGACCC CCTCCGTCCG CGAGTCGGCT CTCCGCCCGC TCCCGTGCCG AGTCGTGACC GGTGCCGACG ACCGCGTTTG CGTGGCACGG GGTCGGGCCC GCCTGGCCCT
Rattus_norvegicus_NR_046239 ---------- ---------- ---------- ---------- ---------- ---------- ---------- ---------- ---------- ---------- ----------
```

```
Gallus_gallus_KT445934.2         ---------- ---------- ---------- ---------- ---------- ---------- ---------- -------AGG AGCGAGTGAG GACTCGGCTC CGGTAGTGGC

                                 2222222222 2222222222 2222222222 2222222222 2222222222 2222222222 2222222222 2222222222 2222222222 2222222222 2222222222
                                 2222222222 2222222222 2222222222 2222222222 2222222222 2222222222 2222222222 2222222222 2222222222 2222222223 3333333333
                                 0000000001 1111111112 2222222223 3333333334 4444444445 5555555556 6666666667 7777777778 8888888889 9999999990 0000000001
                                 1234567890 1234567890 1234567890 1234567890 1234567890 1234567890 1234567890 1234567890 1234567890 1234567890 1234567890
Crocodylus_porosus_EU727191      ---------- ---------- ---------- ---------- ---------- ---------- ---------- ---------- ---------- ---------- ----------
Xenopus_laevis_X02995            ---------- ---------- ---------- ---------- ---------- ---------- ---------- ---------- ---------- ---------- ----------
Mus_musculus_NR_046233           GCGACGTCGG ACGTGGGGAC CCACTGCCGC TCGGGGGTCT TCGTCGGTAG GCATCGGTGT GTCGGCATCG GTCTCTCTCT CGTGTCGGTG TCGCCTCCTC GGGCTCCCGG
Homo_sapiens_U13369              GGGAAAGCGT CCCACGGTGG GGGCGCGCCG GTCTCCCGGA GCGGGACCGG GTCGGAGGAT GGACGAGAAT CACGAGCGAC GGTGGTGGTG GCGTGTCGGG TTCGTGGCTG
Rattus_norvegicus_NR_046239      ---------- ---------- ---------- ---------- ---------- ---------- ---------- ---------- ---------- ---------- ----------
Gallus_gallus_KT445934.2         GGTGAGCGGG CGCTCGCGAG CAGGGTTGAC CGGCCGGCCG CCTAGAGAGG GGATCGGCGG GGGCGGCGGC GGC---TTTC TCGGGCATCG GTTCGTTCGA TCGGTCCGGT

                                 2222222222 2222222222 2222222222 2222222222 2222222222 2222222222 2222222222 2222222222 2222222222 2222222222 2222222222
                                 3333333333 3333333333 3333333333 3333333333 3333333333 3333333333 3333333333 3333333333 3333333334 4444444444 4444444444
                                 1111111112 2222222223 3333333334 4444444445 5555555556 6666666667 7777777778 8888888889 9999999990 0000000001 1111111112
                                 1234567890 1234567890 1234567890 1234567890 1234567890 1234567890 1234567890 1234567890 1234567890 1234567890 1234567890
Crocodylus_porosus_EU727191      ---------- ---------- ---------- ---------- ---------- ---------- ---------- ---------- ---------- ---------- ----------
Xenopus_laevis_X02995            ---------- ---------- ---------- ---------- ---------- ---------- ---------- ---------- ---------- ---------- ----------
Mus_musculus_NR_046233           GGGGCCGTCG TGTTTCGGGT CGGCTCGGCG CTGCAGGTGT GGTGGGACTG CTCAGGGGAG TGGTGCAGTG TGATTCCCGC CGGTTTTGCC TCGCGTGCCC TGACCGGTCC
Homo_sapiens_U13369              CGGTCGCTCC GGGGCCCCCG GTGGCGGGGC CCCGGGGCTC GCGAGGCGGT TCTCGGTGGG GGCCGAGGGC CGTCCGGCGT CCCAGGCGGG GCGCCGCGGG ACCGCCCTCG
Rattus_norvegicus_NR_046239      ---------- ---------- ---------- ---------- ---------- ---------- ---------- ---------- ---------- ---------- ----------
Gallus_gallus_KT445934.2         CGCTTCGGTT TGTCCGTCGC TCCTCATCCC GCAGCTCTGT CCTGGGCTAA GGCGGTTTTG CAGGCGAGCA GCGAAAAAAA GCCGGAGAAG GCGAGAGAGA GGCAAGAAGC

                                 2222222222 2222222222 2222222222 2222222222 2222222222 2222222222 2222222222 2222222222 2222222222 2222222222 2222222222
                                 4444444444 4444444444 4444444444 4444444444 4444444444 4444444444 4444444444 4444444445 5555555555 5555555555 5555555555
                                 2222222223 3333333334 4444444445 5555555556 6666666667 7777777778 8888888889 9999999990 0000000001 1111111112 2222222223
                                 1234567890 1234567890 1234567890 1234567890 1234567890 1234567890 1234567890 1234567890 1234567890 1234567890 1234567890
Crocodylus_porosus_EU727191      ---------- ---------- ---------- ---------- ---------- ---------- ---------- ---------- ---------- ---------- ----------
Xenopus_laevis_X02995            ---------- ---------- ---------- ---------- ---------- ---------- ---------- ---------- ---------- ---------- ----------
Mus_musculus_NR_046233           GACGCCCGAG CGGTCTCTCG GTCCCTTGTG AGGACCCCCT TCCGGGAGGG GCCCGTTTCG GCCGCCCTTG CCGTCGTCGC CGGCCCTCGT TCTGCTGTGT CGTTCCCCCC
Homo_sapiens_U13369              TGTCTGTGGC GGTGGGATCC CGCGGCCGTG TTTTCCTGGT GGCCCGGCCG TGCCTGAGGT TTCTCCCCGA GCCGCCGCCT CTGCGGGCTC CCGGGTGCCC TTGCCCTCGC
Rattus_norvegicus_NR_046239      ---------- ---------- ---------- ---------- ---------- ---------- ---------- ---------- ---------- ---------- ----------
Gallus_gallus_KT445934.2         AAGCCGGCTC CCGCGCCGCC AGGGCGAAGG CGAGAGAGAG AGGGAGACGA GAAGGGCACG GGCCGGTCTG CCGGCACCCG AACGTAGGAT GGCCGGGGGC GTCCCCGGCG

                                 2222222222 2222222222 2222222222 2222222222 2222222222 2222222222 2222222222 2222222222 2222222222 2222222222 2222222222
                                 5555555555 5555555555 5555555555 5555555555 5555555555 5555555555 5555555556 6666666666 6666666666 6666666666 6666666666
                                 3333333334 4444444445 5555555556 6666666667 7777777778 8888888889 9999999990 0000000001 1111111112 2222222223 3333333334
                                 1234567890 1234567890 1234567890 1234567890 1234567890 1234567890 1234567890 1234567890 1234567890 1234567890 1234567890
Crocodylus_porosus_EU727191      ---------- ---------- ---------- ---------- ---------- ---------- ---------- ---------- ---------- ---------- ----------
Xenopus_laevis_X02995            ---------- ---------- ---------- ---------- ---------- ---------- ---------- ---------- ---------- ---------- ----------
Mus_musculus_NR_046233           TCCCCGCTCG CCGCAGCCGG TCTTTTTTCC TCTCTCCCCC CCTCTCCTCT GACTGACCCG TGGCCGTGCT GTCGGACCCC CCGCATGGGG GCGGCCGGGC ACGTACGCGT
Homo_sapiens_U13369              GGTCCCCGGC CCTCGCCCGT CTGTGCCCTC TTCCCCGCCC GCCGCCCGCC GATCCTCTTC TTCCCCCCGA GCGGCTCACC GGCTTCACGT CCGTTGGTGG CCCCGCCTGG
Rattus_norvegicus_NR_046239      ---------- ---------- ---------- ---------- ---------- ---------- ---------- ---------- ---------- ---------- ----------
Gallus_gallus_KT445934.2         GGTCCCGCCG CGATGGAAGA GGGGGACCCG GAGGTCGTAG GTCGTGGCGG CGTCGCCTCG TCCTCCTTTC GCACCGCATT CTCACCCGCA CGCGGGAGCC CCGGCCGATT

                                 2222222222 2222222222 2222222222 2222222222 2222222222 2222222222 2222222222 2222222222 2222222222 2222222222 2222222222
                                 6666666666 6666666666 6666666666 6666666666 6666666666 6666666667 7777777777 7777777777 7777777777 7777777777 7777777777
                                 4444444445 5555555556 6666666667 7777777778 8888888889 9999999990 0000000001 1111111112 2222222223 3333333334 4444444445
                                 1234567890 1234567890 1234567890 1234567890 1234567890 1234567890 1234567890 1234567890 1234567890 1234567890 1234567890
Crocodylus_porosus_EU727191      ---------- ---------- ---------- ---------- ---------- ---------- ---------- ---------- ---------- ---------- ----------
Xenopus_laevis_X02995            ---------- ---------- ---------- ---------- ---------- ---------- ---------- ---------- ---------- ---------- ----------
Mus_musculus_NR_046233           CCGGGCGGTC ACCGGGGTCT TGGGGGGGGG CCGAGGGGTA AGAAAGTCGG CTCGGCGGGC GGGAGGAGCT GTGGTTTGGA GGGCGTCCCG GCCCCGCGGC CGTGGCGGTG
Homo_sapiens_U13369              GACCGAACCC GGCACCGCCT CGTGGGGCGC CGCCGCCGGC CACTGATCGG CCCGGCGTCC GCGTCCCCCG GCGCGCGCCT TGGGGACCGG GTCGGTGGCG CGCCGCGTGG
```

```
Rattus_norvegicus_NR_046239    ---------- ---------- ---------- ---------- ---------- ---------- ---------- ---------- ---------- ---------- ----------
Gallus_gallus_KT445934.2       CGTGGCGCTC CTCGGGCGCG TCGGGGAGGC TTCCCGGCGG GCCGGCTCTA TCCCGCTCCC CGGCTCGTTC GGGGTGGCGT GGGGCGGGCC GGTGTTCAGG CACGGGCGAG

                               2222222222 2222222222 2222222222 2222222222 2222222222 2222222222 2222222222 2222222222 2222222222 2222222222 2222222222
                               7777777777 7777777777 7777777777 7777777777 7777777778 8888888888 8888888888 8888888888 8888888888 8888888888 8888888888
                               5555555556 6666666667 7777777778 8888888889 9999999990 0000000001 1111111112 2222222223 3333333334 4444444445 5555555556
                               1234567890 1234567890 1234567890 1234567890 1234567890 1234567890 1234567890 1234567890 1234567890 1234567890 1234567890
Crocodylus_porosus_EU727191    ---------- ---------- ---------- ---------- ---------- ---------- ---------- ---------- ---------- ---------- ----------
Xenopus_laevis_X02995          ---------- ---------- ---------- ---------- ---------- ---------- ---------- ---------- ---------- ---------- ----------
Mus_musculus_NR_046233         TCTTGCGCGG TCTTGGAGAG GGCTGCGTGC GAGGGGAAAA GGTTGCCCCG CGAGGGCAAA GGGAAAGAGG CTAGCAGTGG TCATTGTCCC GACGGTGTGG TGGTCTGTTG
Homo_sapiens_U13369            GGCCCGGTGG GCTTCCCGGA GGGTTCCGGG GGTCGGCCTG CGGCGCGTGC GGGGGAGGAG ACGGTTCCGG GGGACCGGCC GCGGCTGCGG CGGCGGCGGT GGTGGGGGGA
Rattus_norvegicus_NR_046239    ---------- ---------- ---------- ---------- ---------- ---------- ---------- ---------- ---------- ---------- ----------
Gallus_gallus_KT445934.2       CACCTCTCGT CGGACGTTGC CCACGCACAC CCACCTGCAC GTGCGCGTGC GGTCTTTCCG CCGCGCCTGG GGGAAGGGCT CGCGCCTTCT CCCTCCTTTC TTTCTCCTCC

                               2222222222 2222222222 2222222222 2222222222 2222222222 2222222222 2222222222 2222222222 2222222222 2222222222 2222222222
                               8888888888 8888888888 8888888888 8888888889 9999999999 9999999999 9999999999 9999999999 9999999999 9999999999 9999999999
                               6666666667 7777777778 8888888889 9999999990 0000000001 1111111112 2222222223 3333333334 4444444445 5555555556 6666666667
                               1234567890 1234567890 1234567890 1234567890 1234567890 1234567890 1234567890 1234567890 1234567890 1234567890 1234567890
Crocodylus_porosus_EU727191    ---------- ---------- ---------- ---------- ---------- ---------- ---------- ---------- ---------- ---------- ----------
Xenopus_laevis_X02995          ---------- ---------- ---------- ---------- ---------- ---------- ---------- ---------- ---------- ---------- ----------
Mus_musculus_NR_046233         GCCGAGGTGC GTCTGGGGGG CTCGTCCGGC CCTGTCGTCC GTCGGGAAGG CGCGTGTTGG GGCCTGCCGG AGTGCCGAGG TGGGTACCCT GGCGGTGGGA TTAACCCCGC
Homo_sapiens_U13369            GCCGCGGGGA TCGCCGAGGG CCGGTCGGCC GCCCCGGGTG CCCCGCGGTG CCGCCGGCGG CGGTGAGGCC CCGCGCGTGT GTCCCGGCTG CGGTCGGCCG CGCTCGAGGG
Rattus_norvegicus_NR_046239    ---------- ---------- ---------- ---------- ---------- ---------- ---------- ---------- ---------- ---------- ----------
Gallus_gallus_KT445934.2       CCCCCACCCC CTTTCTCCCA CCGATCGATG AGGCCACTCG GGTCGCGTCG GAGAGGGCCC CCGGCGGGCC GGCGCTCTGC GCTCCCTGTC CCAGGGAAGC CGCGGCGGCG

                               2222222222 2222222222 2222222223 3333333333 3333333333 3333333333 3333333333 3333333333 3333333333 3333333333 3333333333
                               9999999999 9999999999 9999999990 0000000000 0000000000 0000000000 0000000000 0000000000 0000000000 0000000000 0000000000
                               7777777778 8888888889 9999999990 0000000001 1111111112 2222222223 3333333334 4444444445 5555555556 6666666667 7777777778
                               1234567890 1234567890 1234567890 1234567890 1234567890 1234567890 1234567890 1234567890 1234567890 1234567890 1234567890
Crocodylus_porosus_EU727191    ---------- ---------- ---------- ---------- ---------- ---------- ---------- ---------- ---------- ---------- ----------
Xenopus_laevis_X02995          -------CTG CAGCCCCACC GGGAGTTCCA GGAGCTCGGG CAGGGGGAGC CGGCTCGTCC CCCGGCACCG GAGGTCCCCG GGCCCTTTGG CGCCCGTTTT TTCGCAAAGT
Mus_musculus_NR_046233         GCGCGTGTCC CGGTGTGGCG GTGGGGGCTC CGGTCGATGT CTACCTCCCT CTCCCCGAGG TCTCAGGCCT TCTCCGCGCG GGCTCTCGGC CCTCCCCTCG TTCCTCCCTC
Homo_sapiens_U13369            GTCCCCGTGG CGTCCCCTTC CCCGCCGGCC GCCTTTCTCG CGCCTTCCCC GTCGCCCCGG CCTCGCCCGT GGTCTCTCGT CTTCTCCCGG CCCGCTCTTC CGAACCGGGT
Rattus_norvegicus_NR_046239    ---------- ---------- ---------- ---------- ---------- ---------- ---------- ---------- ---------- ---------- ----------
Gallus_gallus_KT445934.2       TCCGGTGTTC AGGCACGGGC GGCCTCCTCT CCAGTTCGCT TCCCGTCGTT CGCGAGGTGA GGCGCTCGCC CGCTTGGGCC GAGGGCGGCG GCGGCGGCGG CTTCGGGGCG

                               3333333333 3333333333 3333333333 3333333333 3333333333 3333333333 3333333333 3333333333 3333333333 3333333333 3333333333
                               0000000000 0000000001 1111111111 1111111111 1111111111 1111111111 1111111111 1111111111 1111111111 1111111111 1111111111
                               8888888889 9999999990 0000000001 1111111112 2222222223 3333333334 4444444445 5555555556 6666666667 7777777778 8888888889
                               1234567890 1234567890 1234567890 1234567890 1234567890 1234567890 1234567890 1234567890 1234567890 1234567890 1234567890
Crocodylus_porosus_EU727191    ---------- ---------- ---------- ---------- ---------- ---------- ---------- ---------- ---------- ---------- ----------
Xenopus_laevis_X02995          GCGGCGCCCG CGGGGACTTG CTCGGCCGGG CCGGGCCCCG GCGGCCCCGG GGCCCCGGGG CCCTCCCGCG GAGGCCCCGA TGAGGACGGA TTCGCCCGGC CCGCCCCGGC
Mus_musculus_NR_046233         TCGCGGGGTT CAAGTCGCTC GTCGACCTCC CCTCCTCCGT CCTTCCATCT CTCGCGCAAT GGCGCCGCCC GAGTTCACGG TGGGTTCGTC CTCCGCCTCC GCTTCTCGCC
Homo_sapiens_U13369            CGGCGCGTCC CCCGGGTGCG CCTCGCTTCC CGGGCCTGCC GCGGCCCTTC CCCGAGGCGT CCGTCCCGGG CGTCGGCGTC GGGGAGAGCC CGTCCTCCCC GCGTGGCGTC
Rattus_norvegicus_NR_046239    ---------- ---------- ---------- ---------- ---------- ---------- ---------- ---------- ---------- ---------- ----------
Gallus_gallus_KT445934.2       CGTGGCCTCG CCGTGCCGAC TCGTCTGTCC GCCCGCCCTG TCGGTGCCCC AGGGCTCGCC CGACCGAATC CAGCTGTGTG ACGGCCGAGC GGCCCCGCGA GCCGCAGGCG

                               3333333333 3333333333 3333333333 3333333333 3333333333 3333333333 3333333333 3333333333 3333333333 3333333333 3333333333
                               1111111112 2222222222 2222222222 2222222222 2222222222 2222222222 2222222222 2222222222 2222222222 2222222222 2222222223
                               9999999990 0000000001 1111111112 2222222223 3333333334 4444444445 5555555556 6666666667 7777777778 8888888889 9999999990
                               1234567890 1234567890 1234567890 1234567890 1234567890 1234567890 1234567890 1234567890 1234567890 1234567890 1234567890
Crocodylus_porosus_EU727191    ---------- ---------- ---------- ---------- ---------- ---------- ---------- ---------- ---------- ---------- ----------
Xenopus_laevis_X02995          CGGAGTTCCG GGAGCCCGGG GAGAGGAGCC GGCGGCCCGG CCTCTCGGGC CCCCCGCACG ACGCCTCCAT GCTACGCTTT TTTGGCATGT GCGGGCAGGA AGGTAGGGGA
Mus_musculus_NR_046233         GGGGGCTGGC CGCTGTCCGG TCTCTCCTGC CCGACCCCCG TTGGCGTGGT CTTCTCTCGC CGGCTTCGCG GACTCCTGGC TTCGCCCGGA GGGTCAGGGG GCTTCCCGGT
```

```
Homo_sapiens_U13369            GCCCCGTTCG GCGCGCGCGT GCGCCCGAGC GCGGCCCGGT GGTCCCTCCC GGACAGGCGT TCGTGCGACG TGTGGCGTGG GTCGACCTCC GCCTTGCCGG TCGCTCGCCC
Rattus_norvegicus_NR_046239    ---------- ---------- ---------- ---------- ---------- ---------- ---------- ---------- ---------- ---------- ----------
Gallus_gallus_KT445934.2       TACCCATTTC GTTGTGAGCG AGGCGTCGGC GCTGCCCTCG TTTCGGGGCC CGGCGAGTGC CGGCCGCGAG CAGCAAGCCG GCGGGGTGGC AACCGAGGGA AACCGCGGGG

                               3333333333 3333333333 3333333333 3333333333 3333333333 3333333333 3333333333 3333333333 3333333333 3333333333 3333333333
                               3333333333 3333333333 3333333333 3333333333 3333333333 3333333333 3333333333 3333333333 3333333333 3333333334 4444444444
                               0000000001 1111111112 2222222223 3333333334 4444444445 5555555556 6666666667 7777777778 8888888889 9999999990 0000000001
                               1234567890 1234567890 1234567890 1234567890 1234567890 1234567890 1234567890 1234567890 1234567890 1234567890 1234567890
Crocodylus_porosus_EU727191    ---------- ---------- ---------- ---------- ---------- ---------- ---------- ---------- ---------- ---------- ----------
Xenopus_laevis_X02995          AGACCGGCCC TCGGCGCGAC GGGCGCCCGA AAAAAGGACC GGGGCGTTTC CCGCCTCGGT CCCCGGTCTG GGAAGGCTCC GCGGTCGAGT CTCGCTCCCC GGCCCGATCG
Mus_musculus_NR_046233         TCCCCGACGT TGCGCCTCGC TGCTGTGTGC TTGGGGGGGG CCCGCTGCGG CCTCCGCCCG CCCGTGAGCC CCTGCCGCAC CCGCCGGTGT GCGGTTTCGC GCCGCGGTCA
Homo_sapiens_U13369            TCTCCCCGGG TCGGGGGGTG GGGCCCGGGC CGGGGCCTCG GCCCCGGTCG CTGCCTCCCG TCCCGGGCGG GGGCGGGCGC GCCGGCCGGC CTCGGTCGCC CTCCCTTGGC
Rattus_norvegicus_NR_046239    ---------- ---------- ---------- ---------- ---------- ---------- ---------- ---------- ---------- ---------- ----------
Gallus_gallus_KT445934.2       AACCGAGGCG AAGCGAGCAG CAGCAGAAGA AGGAACGAGA AGACAACGGG GGGCTGCGCC CGGCCGAGCG GGCGAGCCCG GAGCAGCGCG GCGCGTCCCG CTCCGGATCC

                               3333333333 3333333333 3333333333 3333333333 3333333333 3333333333 3333333333 3333333333 3333333333 3333333333 3333333333
                               4444444444 4444444444 4444444444 4444444444 4444444444 4444444444 4444444444 4444444444 4444444445 5555555555 5555555555
                               1111111112 2222222223 3333333334 4444444445 5555555556 6666666667 7777777778 8888888889 9999999990 0000000001 1111111112
                               1234567890 1234567890 1234567890 1234567890 1234567890 1234567890 1234567890 1234567890 1234567890 1234567890 1234567890
Crocodylus_porosus_EU727191    ---------- ---------- ---------- ---------- ---------- ---------- ---------- ---------- ---------- ---------- ----------
Xenopus_laevis_X02995          ATCTGGCAAC CCGCGCCCGG GCGGGGAGGG CCCTCTGCCC GGCCGACCCC CCGGCGGGGC GGCCGCTACA CGGGCAGGGA GGCTCCCTCC CGCCTCGCCC CGGGCGCGAC
Mus_musculus_NR_046233         GTTGGGCCCT GGCGTTGTGT CGCGTCGGGA GCGTGTCCGC CTCGCGGCGG CTAGACGCGG GTGTCGCCGG GCTCCGACGG GTGGCCTATC CAGGGCTCGC CCCCGCCGAC
Homo_sapiens_U13369            CGTCGTGTGG CGTGTGCCAC CCCTGCGCCG GCGCCCGCCG GCGGGGCTCG GAGCCGGGCT TCGGCCGGGC CCCGGGCCCT CGACCGGACC GGCTGCGCGG GCGCTGCGGC
Rattus_norvegicus_NR_046239    ---------- ---------- ---------- ---------- ---------- ---------- ---------- ---------- ---------- ---------- ----------
Gallus_gallus_KT445934.2       GTCGGGGTGT GGGGGCCGGG GGCGTCCGCC GGCCTCTCCT CCGCCTTCGG GCCGCCGCAG CCCGTGTCGG TTTTCCTGCC GCGTCCCCGC CCGCTGCGGA GCGTGCCGCC

                               3333333333 3333333333 3333333333 3333333333 3333333333 3333333333 3333333333 3333333333 3333333333 3333333333 3333333333
                               5555555555 5555555555 5555555555 5555555555 5555555555 5555555555 5555555555 5555555556 6666666666 6666666666 6666666666
                               2222222223 3333333334 4444444445 5555555556 6666666667 7777777778 8888888889 9999999990 0000000001 1111111112 2222222223
                               1234567890 1234567890 1234567890 1234567890 1234567890 1234567890 1234567890 1234567890 1234567890 1234567890 1234567890
Crocodylus_porosus_EU727191    ---------- ---------- ---------- ---------- ---------- ---------- ---------- ---------- ---------- ---------- ----------
Xenopus_laevis_X02995          CCCCGGACCC CCCCCCGGTC CGGTCCGCCG CCGGGCCCAC CCCCCCGGCC GCCCCGGCGG CCGGGGGGCG CCCGCGCCCC CCCCGTGAGT TCCCCCCGCA CCGTCCGACC
Mus_musculus_NR_046233         CCCCGCCTGC CCGTCCCGGT GGTGGTCGTT GGTGTGGGGA GTGAATGGTG CTACCGGTCA TTCCCTCCCG CGTGGTTTGA CTGTCTCGCC GGTGTCGCGC TTCTCTTTCC
Homo_sapiens_U13369            CGCACGGCGC GACTGTCCCC GGGCCGGGCA CCGCGGTCCG CCTCTCGCTC GCCGCCCGGA CGTCGGGGCC GCCCCGCGGG GCGGGCGGAG CGCCGTCCCC GCCTCGCCGC
Rattus_norvegicus_NR_046239    ---------- ---------- ---------- ---------- ---------- ---------- ---------- ---------- ---------- ---------- ----------
Gallus_gallus_KT445934.2       CCGGGAAAGG GTCTCCGATC GTGGGGTCGC GCCCGTCTCG AGGTCGCGTT CTCCTCTAGC ACGTCCGTCT CCGGCGGCGG GGCTTCGTTT CCCCGTCCGC TTCTCCGCCG

                               3333333333 3333333333 3333333333 3333333333 3333333333 3333333333 3333333333 3333333333 3333333333 3333333333 3333333333
                               6666666666 6666666666 6666666666 6666666666 6666666666 6666666666 6666666667 7777777777 7777777777 7777777777 7777777777
                               3333333334 4444444445 5555555556 6666666667 7777777778 8888888889 9999999990 0000000001 1111111112 2222222223 3333333334
                               1234567890 1234567890 1234567890 1234567890 1234567890 1234567890 1234567890 1234567890 1234567890 1234567890 1234567890
Crocodylus_porosus_EU727191    ---------- ---------- ---------- ---------- ---------- ---------- ---------- ---------- ---------- ---------- ----------
Xenopus_laevis_X02995          CCGGCAGGCG CCCGAAGAAG GCCCGGACAG GCGGGGAGCC CGCCCCGGGG GACCCCGCTT CCCCCGGCCG CGGACTCCTC TCCCCTGGCC CGGGCCGAGC ACCCCGTTTC
Mus_musculus_NR_046233         GCCAACCCCC ACGCCAACCC ACCACCCTGC TCTCCCGGCC CGGTGCGGTC GACGTTCCGG CTCTCCCGAT GCCGAGGGGT TCGGGATTTG TGCCGGGGAC GGAGGGGAGA
Homo_sapiens_U13369            CGCCCGCGGG CGCCGGCCGC GCGCGCGCGC GCGTGGCCGC CGGTCCCTCC CGGCCGCCGG GCGCGGGTCG GGCCGTCCGC CTCCTCGCGG GCGGGCGCGA CGAAGAAGCG
Rattus_norvegicus_NR_046239    ---------- ---------- ---------- ---------- ---------- ---------- ---------- ---------- ---------- ---------- ----------
Gallus_gallus_KT445934.2       GTCCCGGAGG GCGGGTCAGC CCCGGCCGGC CGTGCGGCGC GAGCGCGAGT CCGGCTCCCG CGGGGGGGGC CCGGAGCGTG CCGCCGAAAG CAGCTGCGCA GCGGTCCCCG

                               3333333333 3333333333 3333333333 3333333333 3333333333 3333333333 3333333333 3333333333 3333333333 3333333333 3333333333
                               7777777777 7777777777 7777777777 7777777777 7777777777 7777777778 8888888888 8888888888 8888888888 8888888888 8888888888
                               4444444445 5555555556 6666666667 7777777778 8888888889 9999999990 0000000001 1111111112 2222222223 3333333334 4444444445
                               1234567890 1234567890 1234567890 1234567890 1234567890 1234567890 1234567890 1234567890 1234567890 1234567890 1234567890
Crocodylus_porosus_EU727191    ---------- ---------- ---------- ---------- ---AAAAGCG GCCCGGGTGC CTGCCCCTTC CTTCCCCGCT GTCCTTCCCC ATGCTGCTGC CCTGTGGTCC
Xenopus_laevis_X02995          GCCCGACACC CGCAGAGCGA GAGAGAAAGA CGGAAAGAAA GGAGAGTAGG CCGCGGGCCC CGTCCCGGCC GCCGCCTCCC CCCCCCCTCC CCGGGGGGGG GGAGCGGCAG
```

```
Mus_musculus_NR_046233         GCGGGTAAGA GAGGTGTCGG AGAGCTGTCC CGGGGCGACG CTCGGGTTGG CTTTGCCGCG TGCGTGTGCT CGCGGACGGG TTTTGTCGGA CCCCGACGGG GTCGGTCCGG
Homo_sapiens_U13369            TCGCGGGTCT GTGGCGCGGG GCCCCCGGTG GTCGTGTCGC GTGGGGGGCG GGTGGTTGGG GCGTCCGGTT CGCCGCGCCC CGCCCCGGCC CCACCGGTCC CGGCCGCCGC
Rattus_norvegicus_NR_046239    ---------- ---------- ---------- ---------- ---------- ---------- ---------- ---------- ---------- ---------- ----------
Gallus_gallus_KT445934.2       CTCCTTCCCC GCGGGGGGGA GGTCGGCGGG GCCGCCCCGG GGATCGGGCG CGCCTCTCCG TCGTGGTCGG CGAGCGAGCG AGCGAGCGAG GGAACGACGG AGGGCCGCCC

                               3333333333 3333333333 3333333333 3333333333 3333333333 3333333333 3333333333 3333333333 3333333333 3333333333 3333333333
                               8888888888 8888888888 8888888888 8888888888 8888888889 9999999999 9999999999 9999999999 9999999999 9999999999 9999999999
                               5555555556 6666666667 7777777778 8888888889 9999999990 0000000001 1111111112 2222222223 3333333334 4444444445 5555555556
                               1234567890 1234567890 1234567890 1234567890 1234567890 1234567890 1234567890 1234567890 1234567890 1234567890 1234567890
Crocodylus_porosus_EU727191    CCGCACCTTC CCTGGCCCCG GGAAAGGGGC CCTATGGGAC CACCGAGGGG CGGGGGGCTG CGGGCAGGAA AGTCGCTGGC CCTTGCCTGG GGGCCAGTGG GCGCCTGTCC
Xenopus_laevis_X02995          GCCGGGCGGG GCCCCCGGCC CGGACGGGAG GGCCCGGGCG CCGGGAGCGC CGCCGAGGGG ACGGGCCCGG GTGACGCCTC AGGGCGCCGA CCCGCCGCCC CCCCCCCCCG
Mus_musculus_NR_046233         CCGCATGCAC TCTCCCGTTC CGCGCGAGCG CCCGCCCGGC TCACCCCCGG TTTGTCCTCC CGCGAGGCTC TCCGCCGCCG CCGCCTCCTC CTCCTCTCTC GCGCTCTCTG
Homo_sapiens_U13369            CCCCGCGCCC GCTCGCTCCC TCCCGTCCGC CCGTCCGCGG CCCGTCCGTC CGTCCGTCCG TCGTCCTCCT CGCTTGCGGG GCGCCGGGCC CGTCCTCGCG AGGCCCCCCG
Rattus_norvegicus_NR_046239    ---------- ---------- ---------- ---------- ---------- ---------- ---------- ---------- ---------- ---------- ----------
Gallus_gallus_KT445934.2       GCCCCGCCGA GAGGCGTTCG CCCCGGCGGC CGCCGCCGTC GACCCGGCAA GGGCCAGACG GGAAAGCCGA GCGAGCAGGC GAGAGAGAGA GAGAGGGAAG GAGCGAGAGC

                               3333333333 3333333333 3333333333 3333333334 4444444444 4444444444 4444444444 4444444444 4444444444 4444444444 4444444444
                               9999999999 9999999999 9999999999 9999999990 0000000000 0000000000 0000000000 0000000000 0000000000 0000000000 0000000000
                               6666666667 7777777778 8888888889 9999999990 0000000001 1111111112 2222222223 3333333334 4444444445 5555555556 6666666667
                               1234567890 1234567890 1234567890 1234567890 1234567890 1234567890 1234567890 1234567890 1234567890 1234567890 1234567890
Crocodylus_porosus_EU727191    GTGAGGCTAC CCCTTGCCCC ACTCTCCCTC ACCCAAGGAG CATGGC---T TCCTGGTTGA TCCTGCCAGT AGCATATGCT TGTCTCAAAG ATTAAGCCAT GAATGTCTAA
Xenopus_laevis_X02995          GCCGCCCCCG CGCCCGCCCG CCCGCGCCGG GCCCGGGAAA GGTGGC---T ACCTGGTTGA TCCTGCCAGT AGCATATGCT TGTCTCAAAG ATTAAGCCAT GCACGTGTAA
Mus_musculus_NR_046233         TCCCGCCTGG TCCTGTCCCA CCCCCGACGC TCCGCTCGCG CTTCCT---T ACCTGGTTGA TCCTGCCAGT AGCATATGCT TGTCTCAAAG ATTAAGCCAT GCATGTCTAA
Homo_sapiens_U13369            GCCGGCCGTC CGGCCGCGTC GGGGGCTCGC CGCGCTCTAC CTTACC---T ACCTGGTTGA TCCTGCCAGT AGCATATGCT TGTCTCAAAG ATTAAGCCAT GCATGTCTAA
Rattus_norvegicus_NR_046239    ---------- ---------- ---------- ---------- ---------T ACCTGGTTGA TCCTGCCAGT AGCATATGCT TGTCTCAAAG ATTAAGCCAT GCATGTCTAA
Gallus_gallus_KT445934.2       GGTCGGCGGC GGGCCGGGCC CGTCGGGTCG TGCCCCGTGG CGCGGC---T ACCTGGTTGA TCCTGCCAGT AGCATATGCT TGTCTCAAAG ATTAAGCCAT GCATGTCTAA

                               4444444444 4444444444 4444444444 4444444444 4444444444 4444444444 4444444444 4444444444 4444444444 4444444444 4444444444
                               0000000000 0000000000 0000000001 1111111111 1111111111 1111111111 1111111111 1111111111 1111111111 1111111111 1111111111
                               7777777778 8888888889 9999999990 0000000001 1111111112 2222222223 3333333334 4444444445 5555555556 6666666667 7777777778
                               1234567890 1234567890 1234567890 1234567890 1234567890 1234567890 1234567890 1234567890 1234567890 1234567890 1234567890
Crocodylus_porosus_EU727191    ATACACATGG CCAGTACAGT GAAACTGCGA ATGGCTCATT AAATCAGTTA TGGTTCCTTT GGTCGCTC-- -CAACCGTTA CTTGGATAAC TGTGGTAATT CTAGAGCAAA
Xenopus_laevis_X02995          GTACGCACGG CCGGTACAGT GAAACTGCGA ATGGCTCATT AAATCAGTTA TGGTTCCTTT GATCGCTC-- -CATCTGTTA CTTGGATAAC TGTGGTAATT CTAGAGCTAA
Mus_musculus_NR_046233         GTACGCACGG CCGGTACAGT GAAACTGCGA ATGGCTCATT AAATCAGTTA TGGTTCCTTT GGTCGCTCGC TCCTCTCCTA CTTGGATAAC TGTGGTAATT CTAGAGCTAA
Homo_sapiens_U13369            GTACGCACGG CCGGTACAGT GAAACTGCGA ATGGCTCATT AAATCAGTTA TGGTTCCTTT GGTCGCTCGC TCCTCTCCTA CTTGGATAAC TGTGGTAATT CTAGAGCTAA
Rattus_norvegicus_NR_046239    GTACGCACGG CCGGTACAGT GAAACTGCGA ATGGCTCATT AAATCAGTTA TGGTTCCTTT GGTCGCTCGC TCCTCTCCTA CTTGGATAAC TGTGGTAATT CTAGAGCTAA
Gallus_gallus_KT445934.2       GTACACACGG GCGGTACAGT GAAACTGCGA ATGGCTCATT AAATCAGTTA TGGTTCCTTT GGTCGCTC-C CCTCCCGTTA CTTGGATAAC TGTGGTAATT CTAGAGCTAA

                               4444444444 4444444444 4444444444 4444444444 4444444444 4444444444 4444444444 4444444444 4444444444 4444444444 4444444444
                               1111111111 1111111112 2222222222 2222222222 2222222222 2222222222 2222222222 2222222222 2222222222 2222222222 2222222222
                               8888888889 9999999990 0000000001 1111111112 2222222223 3333333334 4444444445 5555555556 6666666667 7777777778 8888888889
                               1234567890 1234567890 1234567890 1234567890 1234567890 1234567890 1234567890 1234567890 1234567890 1234567890 1234567890
Crocodylus_porosus_EU727191    TACATGCTGA TGAGTGCTGA CCTCT----- ------GGGG ATGTGTGCAT TTATCAGACC AAAACCAACC CAG---GTTC AC-------- ---------- --------CC
Xenopus_laevis_X02995          TACATGCCGA CGAGCGCTGA CCCCC----- ------AGGG ATGCGTGCAT TTATCAGACC AAAACCAATC CGG--GGCCC CC-------- ---------- ---GCGCCCC
Mus_musculus_NR_046233         TACATGCCGA CGGGCGCTGA CCCCCCTTCC CG-GGGGGGG ATGCGTGCAT TTATCAGATC AAAACCAACC CGGTGAGCTC CCTCCCGGCT CCGGCCGGGG GTCGGGCGCC
Homo_sapiens_U13369            TACATGCCGA CGGGCGCTGA CCCCCTTCGC ---GGGGGGG ATGCGTGCAT TTATCAGATC AAAACCAACC CGGTCAGCCC CTCTCCGGCC CCGGCCGGGG GGCGGGCGCC
Rattus_norvegicus_NR_046239    TACATGCCGA CGGGCGCTGA CCCCCCTTCC CGTGGGGGGA ACGCGTGCAT TTATCAGATC AAAACCAACC CGGTCAGCCC CCTCCCGGCT CCGGCCGGGG GTCGGGCGCC
Gallus_gallus_KT445934.2       TACATGCCGA CGAGCGCCGA CCTCC----- ------GGGG ACGCGTGCAT TTATCAGACC AAAACCAACC CGG---GCTC GC-------- ---------- --------CC

                               4444444444 4444444444 4444444444 4444444444 4444444444 4444444444 4444444444 4444444444 4444444444 4444444444 4444444444
                               2222222223 3333333333 3333333333 3333333333 3333333333 3333333333 3333333333 3333333333 3333333333 3333333333 3333333334
                               9999999990 0000000001 1111111112 2222222223 3333333334 4444444445 5555555556 6666666667 7777777778 8888888889 9999999990
                               1234567890 1234567890 1234567890 1234567890 1234567890 1234567890 1234567890 1234567890 1234567890 1234567890 1234567890
Crocodylus_porosus_EU727191    GCTGGCTTTG GTGACTCTAG ATAACCTAGG GCCAATCGCA TGCCCCT-GT GGTGGTGATA ATGCATTTGA ATGTCTGCGC TATCAACTTT CAGGGGTACT TTCTGTGCCT
```

```
Xenopus_laevis_X02995           GGCCGCTTTG GTGACTCTAG ATAACCTCGG GCCGATCGCA CGTCCCC-GT GACGGCGACG ATACATTCGG ATGTCTGCCC TATCAACTTT CGATGGTACT TTCTGCGCCT
Mus_musculus_NR_046233          GGCGGCTT-G GTGACTCTAG ATAACCTCGG GCCGATCGCA CGCCCCCCGT GGCGGCGACG ACCCATTCGA ACGTCTGCCC TATCAACTTT CGATGGTAGT CGCCGTGCCT
Homo_sapiens_U13369             GGCGGCTTTG GTGACTCTAG ATAACCTCGG GCCGATCGCA CGCCCCCCGT GGCGGCGACG ACCCATTCGA ACGTCTGCCC TATCAACTTT CGATGGTAGT CGCCGTGCCT
Rattus_norvegicus_NR_046239     GGCGGCTTTG GTGACTCTAG ATAACCTCGG GCCGATCGCA CGTCCCC-GT GGCGGCGACG ACCCATTCGA ACGTCTGCCC TATCAACTTT CGATGGTAGT CGCCGTGCCT
Gallus_gallus_KT445934.2        GGCGGCTTTG GTGACTCTAG ATAACCTCGA GCCGATCGCA CGCCCCC-GT GGCGGCGACG ACCCATTCGA ATGTCTGCCC TATCAACTTT CGATGGTACT GTCTGTGCCT

                                4444444444 4444444444 4444444444 4444444444 4444444444 4444444444 4444444444 4444444444 4444444444 4444444444 4444444444
                                4444444444 4444444444 4444444444 4444444444 4444444444 4444444444 4444444444 4444444444 4444444444 4444444445 5555555555
                                0000000001 1111111112 2222222223 3333333334 4444444445 5555555556 6666666667 7777777778 8888888889 9999999990 0000000001
                                1234567890 1234567890 1234567890 1234567890 1234567890 1234567890 1234567890 1234567890 1234567890 1234567890 1234567890
Crocodylus_porosus_EU727191     ACCATGGTGA CCATGGGTAA CAGGGAATCA GGGTTTGCTT CCAGAGAGGG AGCCTGAGAA ATGGCTACCA CATCCAGCAG GAAGGCAGCA GGTGTGCAAA TTACCCACTC
Xenopus_laevis_X02995           ACCATGGTGA CCACGGGTAA CGGGGAATCA GGGTTCGATT CCGGAGAGGG AGCCTGAGAA ACGGCTACCA CATCCA--AG GAAGGCAGCA GGCGCGCAAA TTACCCACTC
Mus_musculus_NR_046233          ACCATGGTGA CCACGGGTGA CGGGGAATCA GGGTTCGATT CCGGAGAGGG AGCCTGAGAA ACGGCTACCA CATCCA--AG GAAGGCAGCA GGCGCGCAAA TTACCCACTC
Homo_sapiens_U13369             ACCATGGTGA CCACGGGTGA CGGGGAATCA GGGTTCGATT CCGGAGAGGG AGCCTGAGAA ACGGCTACCA CATCCA--AG GAAGGCAGCA GGCGCGCAAA TTACCCACTC
Rattus_norvegicus_NR_046239     ACCATGGTGA CCACGGGTGA CGGGGAATCA GGGTTCGATT CCGGAGAGGG AGCCTGAGAA ACGGCTACCA CATCCA--AG GAAGGCAGCA GGCGCGCAAA TTACCCACTC
Gallus_gallus_KT445934.2        ACCATGGTGA CCACGGGTAA CGGGGAATCA GGGTTCGATT CCGGAGAGGG AGCCTGAGAA ACGGCTACCA CATCCA--AG GAAGGCAGCA GGCGCGCAAA TTACCCACTC

                                4444444444 4444444444 4444444444 4444444444 4444444444 4444444444 4444444444 4444444444 4444444444 4444444444 4444444444
                                5555555555 5555555555 5555555555 5555555555 5555555555 5555555555 5555555555 5555555555 5555555556 6666666666 6666666666
                                1111111112 2222222223 3333333334 4444444445 5555555556 6666666667 7777777778 8888888889 9999999990 0000000001 1111111112
                                1234567890 1234567890 1234567890 1234567890 1234567890 1234567890 1234567890 1234567890 1234567890 1234567890 1234567890
Crocodylus_porosus_EU727191     CTGACCCAGG AAGGTAGTGA AGAAAAATAA CAATACAGGA CTCTTTTGAG CCCCTGTAAT TGGAAGGAGT ACACTTTAAA TCCTTTAATG AGGATCTATT GGAGGGCAAG
Xenopus_laevis_X02995           CCGACGCGGG GAGGTAGTGA CGAAAAATAA CAATACAGGA CTCTTTCGAG GCCCTGTAAT TGGAATGAGT ACACTTTAAA TCCTTTAACG AGGATCTATT GGAGGGCAAG
Mus_musculus_NR_046233          CCGACCCGGG GAGGTAGTGA CGAAAAATAA CAATACAGGA CTCTTTCGAG GCCCTGTAAT TGGAATGAGT CCACTTTAAA TCCTTTAACG AGGATCCATT GGAGGGCAAG
Homo_sapiens_U13369             CCGACCCGGG GAGGTAGTGA CGAAAAATAA CAATACAGGA CTCTTTCGAG GCCCTGTAAT TGGAATGAGT CCACTTTAAA TCCTTTAACG AGGATCCATT GGAGGGCAAG
Rattus_norvegicus_NR_046239     CCGACCCGGG GAGGTAGTGA CGAAAAATAA CAATACAGGA CTCTTTCGAG GCCCTGTAAT TGGAATGAGT CCACTTTAAA TCCTTTAACG AGGATCCATT GGAGGGCAAG
Gallus_gallus_KT445934.2        CCGACCCGGG GAGGTAGTGA CGAAAAATAA CAATACAGGA CTCTTTCGAG GCCCTGTAAT TGGAATGAGT CCACTTTAAA TCCTTTAACG AGGATCCATT GGAGGGCAAG

                                4444444444 4444444444 4444444444 4444444444 4444444444 4444444444 4444444444 4444444444 4444444444 4444444444 4444444444
                                6666666666 6666666666 6666666666 6666666666 6666666666 6666666666 6666666666 6666666667 7777777777 7777777777 7777777777
                                2222222223 3333333334 4444444445 5555555556 6666666667 7777777778 8888888889 9999999990 0000000001 1111111112 2222222223
                                1234567890 1234567890 1234567890 1234567890 1234567890 1234567890 1234567890 1234567890 1234567890 1234567890 1234567890
Crocodylus_porosus_EU727191     TCTGGTGCCA GCAGCCATGT TAATTCCAGC TCCAATAGTG TATATTAAAG TTGCTGCAAT TAAAAAGCTC GTAGTTGGAT CTTGGGATCG AGCTGGCGGT CCGCCGCGAG
Xenopus_laevis_X02995           TCTGGTGCCA GCAGCCGCGG TAATTCCAGC TCCAATAGCG TATATTAAAG TTGCTGCAGT TAAAAAGCTC GTAGTTGGAT CTTGGGATCG AGCTGGCGGT CCGCCGCGAG
Mus_musculus_NR_046233          TCTGGTGCCA GCAGCCGCGG TAATTCCAGC TCCAATAGCG TATATTAAAG TTGCTGCAGT TAAAAAGCTC GTAGTTGGAT CTTGGGAGCG GGCGGGCGGT CCGCCGCGAG
Homo_sapiens_U13369             TCTGGTGCCA GCAGCCGCGG TAATTCCAGC TCCAATAGCG TATATTAAAG TTGCTGCAGT TAAAAAGCTC GTAGTTGGAT CTTGGGAGCG GGCGGGCGGT CCGCCGCGAG
Rattus_norvegicus_NR_046239     TCTGGTGCCA GCAGCCGCGG TAATTCCAGC TCCAATAGCG TATATTAAAG TTGCTGCAGT TAAAAAGCTC GTAGTTGGAT CTTGGGAGCG GGCGGGCGGT CCGCCGCGAG
Gallus_gallus_KT445934.2        TCTGGTGCCA GCAGCCGCGG TAATTCCAGC TCCAATAGCG TATATTAAAG TTGCTGCAGT TAAAAAGCTC GTAGTTGGAT CTTGGGATCG AGCTGGCGGT CCGCCGCGAG

                                4444444444 4444444444 4444444444 4444444444 4444444444 4444444444 4444444444 4444444444 4444444444 4444444444 4444444444
                                7777777777 7777777777 7777777777 7777777777 7777777777 7777777777 7777777778 8888888888 8888888888 8888888888 8888888888
                                3333333334 4444444445 5555555556 6666666667 7777777778 8888888889 9999999990 0000000001 1111111112 2222222223 3333333334
                                1234567890 1234567890 1234567890 1234567890 1234567890 1234567890 1234567890 1234567890 1234567890 1234567890 1234567890
Crocodylus_porosus_EU727191     GCGAGC-TAC CGCCT--GTC CCAGCCCCTG CCTCTCGGCG CTCCCTTGAT GCTCTTAACT GAGTGTCCTG -GGGGTCCGA AGCGTTTACT TTGAAAAAAT TAGAGTGTTC
Xenopus_laevis_X02995           GCGGCT--AC CGCCT--GTC CCAGCCCCTG CCTCTCGGCG CCTCCCCGAT GCTCTTGACT GAGTGTCCCG -GGGGCCCGA AGCGTTTACT TTGAAAAAAT TAGAGTGTTC
Mus_musculus_NR_046233          GCGAGT-CAC CGCCC-GTCC CCGCCCCTTG CCTCTCGGCG CCCCCTCGAT GCTCTTAGCT GAGTGTCCCG CGGGGCCCGA AGCGTTTACT TTGAAAAAAT TAGAGTGTTC
Homo_sapiens_U13369             GCGAGC-CAC CGCCC-GTCC CCGCCCCTTG CCTCTCGGCG CCCCCTCGAT GCTCTTAGCT GAGTGTCCCG CGGGGCCCGA AGCGTTTACT TTGAAAAAAT TAGAGTGTTC
Rattus_norvegicus_NR_046239     GCGAGCTCAC CGCCCTGTCC CCAGCCCCTG CCTCTCGGCG CCCCCTCGAT GCTCTTAGCT GAGTGTCCCG CGGGGCCCGA AGCGTTTACT TTGAAAAAAT TAGAGTGTTC
Gallus_gallus_KT445934.2        GCGAGC-TAC CGCCT--GTC CCAGCCCCTG TCTCTCGGCG CCCCCTCGAT GCTCTTAACT GAGTGTCCCG CGGGGCCCGA AGCGTTTACT TTGAAAAAAT TAGAGTGTTC
```

```
                             4444444444 4444444444 4444444444 4444444444 4444444444 4444444444 4444444444 4444444444 4444444444 4444444444 4444444444
                             8888888888 8888888888 8888888888 8888888888 8888888888 8888888889 9999999999 9999999999 9999999999 9999999999 9999999999
                             4444444445 5555555556 6666666667 7777777778 8888888889 9999999990 0000000001 1111111112 2222222223 3333333334 4444444445
                             1234567890 1234567890 1234567890 1234567890 1234567890 1234567890 1234567890 1234567890 1234567890 1234567890 1234567890
Crocodylus_porosus_EU727191  AAAGCAGGCT --GGTCGCCG GAATACTCCA GCTAGGAATA ATGGAATAGG ACTCCCGTTC TATTTTGTTG GTTTTCGGAA CTGGGGCCAT GATTAAGAGG GACGGCCGGG
Xenopus_laevis_X02995        CAAGCAGGCC -GCGTCGCCT GGATACTTCA GCTAGGAATA ATGGAATAGG ACTCCGGTTC TATTTTGTTG GTTTTCGGAA CTGGGGCCAT GATTAAGAGG GACGGCCGGG
Mus_musculus_NR_046233       AAAGCAGGCC CGAGCCGCCT GGATACCGCA GCTAGGAATA ATGGAATAGG ACCGCGGTTC TATTTTGTTG GTTTTCGGAA CTGAGGCCAT GATTAAGAGG GACGGCCGGG
Homo_sapiens_U13369          AAAGCAGGCC CGAGCCGCCT GGATACCGCA GCTAGGAATA ATGGAATAGG ACCGCGGTTC TATTTTGTTG GTTTTCGGAA CTGAGGCCAT GATTAAGAGG GACGGCCGGG
Rattus_norvegicus_NR_046239  AAAGCAGGCC CGAGCCGCCT GGATACCGCA GCTAGGAATA ATGGAATAGG ACCGCGGTTC TATTTTGTTG GTTTTCGGAA CTGAGGCCAT GATTAAGAGG GACGGCCGGG
Gallus_gallus_KT445934.2     AAAGCAGGCT --GGCCGCCG GAATACTCCA GCTAGGAATA ATGGAATAGG ACTCCGGTTC TATTTTGTTG GTTTTCGGAA ACGGGGCCAT GATTAAGAGG GACGGCCGGG

                             4444444444 4444444444 4444444444 4444444444 4444444445 5555555555 5555555555 5555555555 5555555555 5555555555 5555555555
                             9999999999 9999999999 9999999999 9999999999 9999999990 0000000000 0000000000 0000000000 0000000000 0000000000 0000000000
                             5555555556 6666666667 7777777778 8888888889 9999999990 0000000001 1111111112 2222222223 3333333334 4444444445 5555555556
                             1234567890 1234567890 1234567890 1234567890 1234567890 1234567890 1234567890 1234567890 1234567890 1234567890 1234567890
Crocodylus_porosus_EU727191  GGCATTCGTA TTGTGCCGCT AGAGGTGAAA TTCTTGGACC GGCGCAAGAC GGACCAAAGC GAAAGCATTT GCCAAGAATG TTTTCATTAA TCAAGAACGA AAGTCGGAGG
Xenopus_laevis_X02995        GGCATTCGTA TTGTGCCGCT AGAGGTGAAA TTCTTGGACC GGCGCAAGAC GAACCAAAGC GAAAGCATTT GCCAAGAATG TTTTCATTAA TCAAGAACGA AAGTCGGAGG
Mus_musculus_NR_046233       GGCATTCGTA TTGCGCCGCT AGAGGTGAAA TTCTTGGACC GGCGCAAGAC GGACCAGAGC GAAAGCATTT GCCAAGAATG TTTTCATTAA TCAAGAACGA AAGTCGGAGG
Homo_sapiens_U13369          GGCATTCGTA TTGCGCCGCT AGAGGTGAAA TTCTTGGACC GGCGCAAGAC GGACCAGAGC GAAAGCATTT GCCAAGAATG TTTTCATTAA TCAAGAACGA AAGTCGGAGG
Rattus_norvegicus_NR_046239  GGCATTCGTA TTGCGCCGCT AGAGGTGAAA TTCTTGGACC GGCGCAAGAC GAACCAGAGC GAAAGCATTT GCCAAGAATG TTTTCATTAA TCAAGAACGA AAGTCGGAGG
Gallus_gallus_KT445934.2     GGCATTCGTA TTGTGCCGCT AGAGGTGAAA TTCTTGGACC GGCGCAAGAC GAACTAAAGC GAAAGCATTT GCCAAGAATG TTTTCATTAA TCAAGAACGA AAGTCGGAGG

                             5555555555 5555555555 5555555555 5555555555 5555555555 5555555555 5555555555 5555555555 5555555555 5555555555 5555555555
                             0000000000 0000000000 0000000000 0000000001 1111111111 1111111111 1111111111 1111111111 1111111111 1111111111 1111111111
                             6666666667 7777777778 8888888889 9999999990 0000000001 1111111112 2222222223 3333333334 4444444445 5555555556 6666666667
                             1234567890 1234567890 1234567890 1234567890 1234567890 1234567890 1234567890 1234567890 1234567890 1234567890 1234567890
Crocodylus_porosus_EU727191  TTCGAAGACG ATCAGATACC GTCGTAGTTC CGACCATAAA CGATGCCGAC TAGCGATCCG GCGGCGTTAT TCCCATGACC CGCCGGGCAG CTTCCGGGAA ACCAAAGTCT
Xenopus_laevis_X02995        TTCGAAGACG ATCAGATACC GTCGTAGTTC CGACCATAAA CGATGCCGAC TAGCGATCCG GCGGCGTTAT TCCCATGACC CGCCGAGCAG CTTCCGGGAA ACCAAAGTCT
Mus_musculus_NR_046233       TTCGAAGACG ATCAGATACC GTCGTAGTTC CGACCATAAA CGATGCCGAC TGGCGATGCG GCGGCGTTAT TCCCATGACC CGCCGGGCAG CTTCCGGGAA ACCAAAGTCT
Homo_sapiens_U13369          TTCGAAGACG ATCAGATACC GTCGTAGTTC CGACCATAAA CGATGCCGAC CGGCGATGCG GCGGCGTTAT TCCCATGACC CGCCGGGCAG CTTCCGGGAA ACCAAAGTCT
Rattus_norvegicus_NR_046239  TTCGAAGACG ATCAGATACC GTCGTAGTTC CGACCATAAA CGATGCCGAC TGGCGATGCG GCGGCGTTAT TCCCATGACC CGCCGGGCAG CTTCCGGGAA ACCAAAGTCT
Gallus_gallus_KT445934.2     TTCGAAGACG ATCAGATACC GTCGTAGTTC CGACCATAAA CGATGCCGAC TCGCGATCCG GCGGCGTTAT TCCCATGACC CGCCGGGCAG CTCCCGGGAA ACCCAAGTCT

                             5555555555 5555555555 5555555555 5555555555 5555555555 5555555555 5555555555 5555555555 5555555555 5555555555 5555555555
                             1111111111 1111111111 1111111112 2222222222 2222222222 2222222222 2222222222 2222222222 2222222222 2222222222 2222222222
                             7777777778 8888888889 9999999990 0000000001 1111111112 2222222223 3333333334 4444444445 5555555556 6666666667 7777777778
                             1234567890 1234567890 1234567890 1234567890 1234567890 1234567890 1234567890 1234567890 1234567890 1234567890 1234567890
Crocodylus_porosus_EU727191  CTGGGTTCCG GGGGGAGTAT GGTTGCAAAG CTGAAACTTA AAGGAATTGA CGGAAGGGCA CCACCAGGAG TGGATCCTGC GGCTTAATTT GACTCAACAT GGGATACCTC
Xenopus_laevis_X02995        TTGGGTTCCG GGGGGAGTAT GGTTGCAAAG CTGAAACTTA AAGGAATTGA CGGAAGGGCA CCACCAGGAG TGGAGCCTGC GGCTTAATTT GACTCAACAC GGGAAACCTC
Mus_musculus_NR_046233       TTGGGTTCCG GGGGGAGTAT GGTTGCAAAG CTGAAACTTA AAGGAATTGA CGGAAGGGCA CCACCAGGAG TGG-GCCTGC GGCTTAATTT GACTCAACAC GGGAAACCTC
Homo_sapiens_U13369          TTGGGTTCCG GGGGGAGTAT GGTTGCAAAG CTGAAACTTA AAGGAATTGA CGGAAGGGCA CCACCAGGAG TGGAGCCTGC GGCTTAATTT GACTCAACAC GGGAAACCTC
Rattus_norvegicus_NR_046239  TTGGGTTCCG GGGGGAGTAT GGTTGCAAAG CTGAAACTTA AAGGAATTGA CGGAAGGGCA CCACCAGGAG TGGAGCCTGC GGCTTAATTT GACTCAACAC GGGAAACCTC
Gallus_gallus_KT445934.2     TTGGGTTCCG GGGGGAGTAT GGTTGCAAAG CTGAAACTTA AAGGAATTGA CGGAAGGGCA CCACCAGGAG TGGAGCCTGC GGCTTAATTT GACTCAACAC GGGAAACCTC

                             5555555555 5555555555 5555555555 5555555555 5555555555 5555555555 5555555555 5555555555 5555555555 5555555555 5555555555
                             2222222222 2222222223 3333333333 3333333333 3333333333 3333333333 3333333333 3333333333 3333333333 3333333333 3333333333
                             8888888889 9999999990 0000000001 1111111112 2222222223 3333333334 4444444445 5555555556 6666666667 7777777778 8888888889
                             1234567890 1234567890 1234567890 1234567890 1234567890 1234567890 1234567890 1234567890 1234567890 1234567890 1234567890
Crocodylus_porosus_EU727191  ACCTGACCCG GCCATGGAAA GGATTG---- ---------- ---------- ----TGGGTG GTGGTGCATG GCCGTTCTTA GTTGGTGGAG TGATTTGTCT GGTTAATTCT
Xenopus_laevis_X02995        ACCCGGCCCG GACACGGAAA GGATTGACAG ATTGATAGCT CTTTCTCGAT TCTGTGGGTG GTGGTGCATG GCCGTTCTTA GTTGGTGGAG CGATTTGTCT GGTTAATTCC
Mus_musculus_NR_046233       ACCCGGCCCG GACACGGACA GGATTGACAG ATTGATAGCT CTTTCTCGAT TCCGTGGGTG GTGGTGCATG GCCGTTCTTA GTTGGTGGAG CGATTTGTCT GGTTAATTCC
Homo_sapiens_U13369          ACCCGGCCCG GACACGGACA GGATTGACAG ATTGATAGCT CTTTCTCGAT TCCGTGGGTG GTGGTGCATG GCCGTTCTTA GTTGGTGGAG CGATTTGTCT GGTTAATTCC
Rattus_norvegicus_NR_046239  ACCCGGCCCG GACACGGACA GGATTGACAG ATTGATAGCT CTTTCTCGAT TCCGTGGGTG GTGGTGCATG GCCGTTCTTA GTTGGTGGAG CGATTTGTCT GGTTAATTCC
Gallus_gallus_KT445934.2     ACCCGGCCCG GACACGGACA GGATTGACAG ATTGAGAGCT CTTTCTCGAT TCCGTGGGTG GTGGTGCATG GCCGTTCTTA GTTGGTGGAG CGATTTGTCT GGTTAATTCC
```

```
                              5555555555 5555555555 5555555555 5555555555 5555555555 5555555555 5555555555 5555555555 5555555555 5555555555 5555555555
                              3333333334 4444444444 4444444444 4444444444 4444444444 4444444444 4444444444 4444444444 4444444444 4444444444 4444444445
                              9999999990 0000000001 1111111112 2222222223 3333333334 4444444445 5555555556 6666666667 7777777778 8888888889 9999999990
                              1234567890 1234567890 1234567890 1234567890 1234567890 1234567890 1234567890 1234567890 1234567890 1234567890 1234567890
Crocodylus_porosus_EU727191   GATAACCAAT GAGACTCT-G GCATGCTAAC TAGTTATGCG ACCCTCGAGC GGTCGCTGTC C---AACTTC TTAGAGGGAC AAGTGGCATT CAGCCACCTG AGATTGAGCA
Xenopus_laevis_X02995         GATAACGAAC GAGACTCC-T CCATGCTAAC TAGTTACGCG ACCCCCG-GC GGTCGGCGTC C---AACTTC TTAGAGGGAC AAGTGGCGTT CAGCCACACG AGATCGAGCA
Mus_musculus_NR_046233        GATAACGAAC GAGACTCT-G GCATGCTAAC TAGTTACGCG ACCCCCGAGC GGTCGGCGTC CCCCAACTTC TTAGAGGGAC AAGTGGCGTT CAGCCACCCG AGATTGAGCA
Homo_sapiens_U13369           GATAACGAAC GAGACTCT-G GCATGCTAAC TAGTTACGCG ACCCCCGAGC GGTCGGCGTC CCCCAACTTC TTAGAGGGAC AAGTGGCGTT CAGCCACCCG AGATTGAGCA
Rattus_norvegicus_NR_046239   GATAACGAAC GAGACTCTCG GCATGCTAAC TAGTTACGCG ACCCCCGAGC GGTCGGCGTC CCCCAACTTC TTAGAGGGAC AAGTGGCGTT CAGCCACC-G AGATTGAGCA
Gallus_gallus_KT445934.2      GATAACGAAC GAGACTCT-G GCATGCTAAC TAGTTACGCG ACCCCCGAGC GGTCGGCGTC C---AACTTC TTAGAGGGAC AAGTGGCGTT CAGCCACCCG AGATTGAGCA

                              5555555555 5555555555 5555555555 5555555555 5555555555 5555555555 5555555555 5555555555 5555555555 5555555555 5555555555
                              5555555555 5555555555 5555555555 5555555555 5555555555 5555555555 5555555555 5555555555 5555555555 5555555556 6666666666
                              0000000001 1111111112 2222222223 3333333334 4444444445 5555555556 6666666667 7777777778 8888888889 9999999990 0000000001
                              1234567890 1234567890 1234567890 1234567890 1234567890 1234567890 1234567890 1234567890 1234567890 1234567890 1234567890
Crocodylus_porosus_EU727191   ATAACAGGTC TGTGATGCCC TTAGGTGTCC AGGGCTGCAC GCGCACTACA CTGA-CTGGC TCAGGGTGTG TCTACCCTAT GCCAACAGGT GCAAGTAACC CATTGAACCC
Xenopus_laevis_X02995         ATAACAGGTC TGTGATGCCC TTAGATGTCC GGGGCTGCAC GCGCGCTACA CTGA-ACGGA TCAGCGTGTG TCTACCCTGC GCCGACAGGT GCGGGTAACC CGCTGAACCC
Mus_musculus_NR_046233        ATAACAGGTC TGTGATGCCC TTAGATGTCC GGGGCTGCAC GCGCGCTACA CTGA-CTGGC TCAGCGTGTG CCTACCCTGC GCCGGCAGGC GCGGGTAACC CGTTGAACCC
Homo_sapiens_U13369           ATAACAGGTC TGTGATGCCC TTAGATGTCC GGGGCTGCAC GCGCGCTACA CTGA-CTGGC TCAGCGTGTG CCTACCCTAC GCCGGCAGGC GCGGGTAACC CGTTGAACCC
Rattus_norvegicus_NR_046239   ATAACAGGTC TGTGATGCCC TTAGATGTCC GGGGCTGCAC GCGCGCTACA CTGAACTGGC TCAGCGTGTG CCTACCCTAC GCCGGCAGGC GCGGGTAACC CGTTGAACCC
Gallus_gallus_KT445934.2      ATAACAGGTC TGTGATGCCC TTAGATGTCC GGGGCTGCAC GCGCGCTACA CTGA-CTGGC TCAGCTTGTG TCTACCCTAC GCCGGCAGGC GCGGGTAACC CGTTGAACCC

                              5555555555 5555555555 5555555555 5555555555 5555555555 5555555555 5555555555 5555555555 5555555555 5555555555 5555555555
                              6666666666 6666666666 6666666666 6666666666 6666666666 6666666666 6666666666 6666666666 6666666667 7777777777 7777777777
                              1111111112 2222222223 3333333334 4444444445 5555555556 6666666667 7777777778 8888888889 9999999990 0000000001 1111111112
                              1234567890 1234567890 1234567890 1234567890 1234567890 1234567890 1234567890 1234567890 1234567890 1234567890 1234567890
Crocodylus_porosus_EU727191   CATTTGAGAT GGGGATTGGG GATTACAATT ATTCCCCATG AAAGAGG-AA TTCCC-AGTA AGTACAGGTC ACAAGCTTGC ATTGATTAAG TCCCTGCCTT TTGTACACGC
Xenopus_laevis_X02995         CGTTCGTGAT AGGGATCGGG GATTGCAATT ATTTCCCATG AACGAGG-AA TTCCC-AGTA AGTGCGGGTC ATAAGCTCGC GTTGATTAAG TCCCTGCCCT TTGTACACAC
Mus_musculus_NR_046233        CATTCGTGAT GGGGATCGGG GATTGCAATT ATTCCCCATG AACGAGG-AA TTCCC-AGTA AGTGCGGGTC ATAAGCTTGC GTTGATTAAG TCCCTGCCCT TTGTACACAC
Homo_sapiens_U13369           CATTCGTGAT GGGGATCGGG GATTGCAATT ATTCCCCATG AACGAGGGAA TTCCCGAGTA AGTGCGGGTC ATAAGCTTGC GTTGATTAAG TCCCTGCCCT TTGTACACAC
Rattus_norvegicus_NR_046239   CATTCGTGAT GGGGATCGGG GATTGCAATT ATTCCCCATG AACGAGG-AA TTCCC-AGTA AGTGCGGGTC ATAAGCTTGC GTTGATTAAG TCCCTGCCCT TTGTACACAC
Gallus_gallus_KT445934.2      CATTCGTGAT GGGGATCGGG GATTGCAATT ATTCCCCATG AACGAGG-AA TTCCC-AGTA AGTGCGGGTC ATAAGCTCGC GTTGATTAAG TCCCTGCCCT TTGTACACAC

                              5555555555 5555555555 5555555555 5555555555 5555555555 5555555555 5555555555 5555555555 5555555555 5555555555 5555555555
                              7777777777 7777777777 7777777777 7777777777 7777777777 7777777777 7777777777 7777777778 8888888888 8888888888 8888888888
                              2222222223 3333333334 4444444445 5555555556 6666666667 7777777778 8888888889 9999999990 0000000001 1111111112 2222222223
                              1234567890 1234567890 1234567890 1234567890 1234567890 1234567890 1234567890 1234567890 1234567890 1234567890 1234567890
Crocodylus_porosus_EU727191   CACCCGTTGC TACTACCAAT TGGATGGTTT AGTGAGGTCC TCGGATTGAC CCCGCCAGCG TTGGTC-ATG GCCCTGGCAG TGTGCCGATA AGACGGTCGA ACATGACTAT
Xenopus_laevis_X02995         CGCCCGTCGC TACTACCGAT TGGATGGTTT AGTGAGGTCC TCGGATCGGC CCCGCCGGGG TCGGCC-ACG GCCCTGGCGG AGCGCCGAGA AGACGATCAA ACTTGACTAT
Mus_musculus_NR_046233        CGCCCGTCGC TACTACCGAT TGGATGGTTT AGTGAGGCCC TCGGATCGGC CCCGCCGGGG TCGGCCCACG GCCCTGGCGG AGCGCTGAGA AGACGGTCGA ACTTGACTAT
Homo_sapiens_U13369           CGCCCGTCGC TACTACCGAT TGGATGGTTT AGTGAGGCCC TCGGATCGGC CCCGCCGGGG TCGGCCCACG GCCCTGGCGG AGCGCTGAGA AGACGGTCGA ACTTGACTAT
Rattus_norvegicus_NR_046239   CGCCCGTCGC TACTACCGAT TGGATGGTTT AGTGAGGCCC TCGGATCGGC CCCGCCGGGG TCGGCCCACG GCCTTGGCGG AGGCCTGAGA AGACGGTCGA ACTTGACTAT
Gallus_gallus_KT445934.2      CGCCCGTCGC TACTACCGAT TGGATGGTTT AGTGAGGTCC TCGGATCGGC CCCGGCGGGG TCGGCC-ACG GCCCTGCCGG AGCGTCGAGA AGACGGTCGA ACTTGACTAT

                              5555555555 5555555555 5555555555 5555555555 5555555555 5555555555 5555555555 5555555555 5555555555 5555555555 5555555555
                              8888888888 8888888888 8888888888 8888888888 8888888888 8888888888 8888888889 9999999999 9999999999 9999999999 9999999999
                              3333333334 4444444445 5555555556 6666666667 7777777778 8888888889 9999999990 0000000001 1111111112 2222222223 3333333334
                              1234567890 1234567890 1234567890 1234567890 1234567890 1234567890 1234567890 1234567890 1234567890 1234567890 1234567890
Crocodylus_porosus_EU727191   CTAGAAAAAG TAAAAGTTGC AACAAGGTTT CCGTAGGTGA ACCTGTGGAA GGATCATTA- --ATGGGGTC ACCGTGCATG GTGCCAGCTC AGCAGGCATG GGGTGGAGGG
Xenopus_laevis_X02995         CTAGAGGAAG TAAAAGTCGT AACAAGGTTT CCGTAGGTGA ACCTGCGGAA GGATCATTA- --ACGAGACC CCCCTCACCC GGAGAGAGGG AAGGCGCCCG CCGCACCCTC
Mus_musculus_NR_046233        CTAGAGGAAG TAAAAGTCGT AACAAGGTTT CCGTAGGTGA ACCTGCGGAA GGATCATTA- --AACGGGA- GACTGTGGAG GAGCGGCGGC GTGGCCCGCT CTCCCCGTCT
Homo_sapiens_U13369           CTAGAGGAAG TAAAAGTCGT AACAAGGTTT CCGTAGGTGA ACCTGCGGAA GGATCATTA- --ACGGAGCC CGGAGGGCGA GGCCCGCGGC GGCGCCGCCG CCGCCGCGCG
Rattus_norvegicus_NR_046239   CTAGAGGAAG TAAAAGTCGT AACAAGGTTT CCGTAGGTGA ACCTGCGGAA GGATCATTA- --ACGGAGAA GGCCGAGGGG GGTCGTGCCC GTCCCCTCTT GGGCCTGTGT
```

```
Gallus_gallus_KT445934.2       CTAGAGGAAG TAAAAGTCGT AACAAGGTTT CCGTAGGTGA ACCTGCGGAA GGATCATTA- --CCGGGGCC GAGGCCGGGC GTCCGGCCGA GCCGTGGCAC GAGCGCGCGC

                               5555555555 5555555555 5555555555 5555555555 5555555555 5555555556 6666666666 6666666666 6666666666 6666666666 6666666666
                               9999999999 9999999999 9999999999 9999999999 9999999999 9999999990 0000000000 0000000000 0000000000 0000000000 0000000000
                               4444444445 5555555556 6666666667 7777777778 8888888889 9999999990 0000000001 1111111112 2222222223 3333333334 4444444445
                               1234567890 1234567890 1234567890 1234567890 1234567890 1234567890 1234567890 1234567890 1234567890 1234567890 1234567890
Crocodylus_porosus_EU727191    CCATGCTCCC CCATCCCCAT CTCTGGCAGC CTTGTGTGCA TGCATGCCCG CTGTGTCTGG GCTTCCAGGA CCTCATGCAG ACCAGTCCTG GGCTGCACAG CTCAGAGTGC
Xenopus_laevis_X02995          CCCGCGGAGA GAGAGAGAGA CGCCCGCCCC GGAGCGGAGA CCGCCCCCCC CCCACGGGGG GGGGGCGGCC GCCCCGAAAG GGACGACGAG GAACCCCCAG ACGGCCCCGG
Mus_musculus_NR_046233         T-GTGTGTGT C---CTCGCC GGGAGGCGCG T--------- -------GCG TCCCGGGTCC CGTCGCC--- -CGCGTGTGG AGCGAGGTGT CTGGAGTGAG GTGAGAGAAG
Homo_sapiens_U13369            CTTCCCTCCG CACACCCACC CCCCCACCGC GACGCGGCGC GTGCGCGGGC GGGGCCCGCG TGCCCGTTCG TTCGCTCGCT CGTTCGTTCG CCGCCCGGCC CCGCCGCCGC
Rattus_norvegicus_NR_046239    GAGTGTTCCT CCTTCTCGCC GGGAGGCGCG TCCCCGGGTG GGTCCCCGTG TCCGGCGTCC TGGCGTCGTG GCGCGTGCCG TGGCCGGCCG GCGGAGGGGG TTTCGGGACA
Gallus_gallus_KT445934.2       GGGCGCGCAG CCTTCCCTTC CCTTCCCCGA GCCCGCTCCG CGCGGAGCGC GGCTCCTCTC CCCCGGTCGA AACGGGGAAA GAAAAAAAAA ACACCGCAAG TCGCTCCGCG

                               6666666666 6666666666 6666666666 6666666666 6666666666 6666666666 6666666666 6666666666 6666666666 6666666666 6666666666
                               0000000000 0000000000 0000000000 0000000000 0000000001 1111111111 1111111111 1111111111 1111111111 1111111111 1111111111
                               5555555556 6666666667 7777777778 8888888889 9999999990 0000000001 1111111112 2222222223 3333333334 4444444445 5555555556
                               1234567890 1234567890 1234567890 1234567890 1234567890 1234567890 1234567890 1234567890 1234567890 1234567890 1234567890
Crocodylus_porosus_EU727191    AGGACAGGTC CCCCAGCCGT GGGGGGCCCA GGGTCTGAGT GGCTCCCCTG GATCCCCCGA GTGGTCTCCT CCACCCCTAC TCCCCTCCCT TCAGGATCTG AGGGGAGACA
Xenopus_laevis_X02995          CGAGGGGGCG GCGGCGGCCC CGGGTCCACC CCGGGCCCGC CCGCCCGCCT CCCCGCCGCG GGCCCGCCCG GGTACCTAGC CGGGGCCGGG GCGCGGGGGC TGGCGCGGGA
Mus_musculus_NR_046233         GG------GT GGGTGGG-GT CGGTCTGGGT CCGTC-TGGG ACCGCCTCCG ATTTCCCCTC CCCCTCCCCT CTCCCTCGTC CGGCTCTGAC CTCGCCACCC TACCGCGGC-
Homo_sapiens_U13369            GAGAGCCGAG AACTCGGGAG GGAGACGGGG GGGAGAGAGA GAGAGAGAGA GAGAGAGAGA GAGAGAGAGA GAGAAAGAAG GGCGTGTCGT TGGTGTGCGC GTGTCGTGGG
Rattus_norvegicus_NR_046239    GGTGTGCGGT GGCAGGGTGT CGGGTCTGGT CCGCCGCGGG ACCTCCTCCG TTTTCTCGCT CTTCCCTCGA CGCCTCCGTG CG--CCCGCC TCCGCCTCC- -GCCGCTGCC
Gallus_gallus_KT445934.2       CGCCTGCCGG CGAGAGAGAA GGGAGACGAG GGCGCGGAGC GCAGCTCCGG GGGGGGAGGC GCGTGTGGGG CGCGCGCTCC GGCGCGTCTC TCCCCCCCCG GCGCCGGTCC

                               6666666666 6666666666 6666666666 6666666666 6666666666 6666666666 6666666666 6666666666 6666666666 6666666666 6666666666
                               1111111111 1111111111 1111111111 1111111112 2222222222 2222222222 2222222222 2222222222 2222222222 2222222222 2222222222
                               6666666667 7777777778 8888888889 9999999990 0000000001 1111111112 2222222223 3333333334 4444444445 5555555556 6666666667
                               1234567890 1234567890 1234567890 1234567890 1234567890 1234567890 1234567890 1234567890 1234567890 1234567890 1234567890
Crocodylus_porosus_EU727191    AGGACTGGGG TCCCTGGAGG GGCTCCTGCC CAGTGCCAGG GATTTTCTTT CAGCAGCATT GGTCCATTGC TGGGCCACCC ACCCCGCCAC GTGGCAGTGC CCTTTCCCAC
Xenopus_laevis_X02995          GCGGGGCGGC CCCAGGGCCG TCCGGCCTCC CCGCGTCCGC CTCCCGCGAC CCGCCCCGGG CGGTTCGAAG ACCCCGCCCG CCGGGCGGCG GGAGGGCCGG GAGGGAGCCG
Mus_musculus_NR_046233         GGCGGCTGCT CGCGGGCGTC TTGCCTCTTT CCCGTCCGGC TC-----TTC CGTGTCTACG AGGGGCGGTA CGTCGTTACG GGTTTTTGAC CCGTCCCGGG GGCGTTCGGT
Homo_sapiens_U13369            GCCGGCGGGC GGCGGGGAGC GGTCCCCGGC CGCGGCCCCG ACGACGTGGG TGTCGGCGGG CGCGGGGGCG GTTCTCGGCG GCGTCGCGGC GGGTCTGGGG GGGTCTCGGT
Rattus_norvegicus_NR_046239    GTCGGCAGC- -GAGGGCGTT CTGCCTCTTT CCCGACCGGC TCCGTGATCT CGTGTGCACC GGGGGTGGTA CGTGATCTC- --TCCGGGCG GTGGCCGGGA CGCGCTCGCT
Gallus_gallus_KT445934.2       GCCGTCGGTC CGCACGCCGC GGGTCCGGTC CGTCCGGTCG CCTCGCCGGC GCGCGCCCGC GCGCGCGCGT CCCGCGGGCC TCGCCCGGGT CGCCGCGCTC CGGAGCGTCC

                               6666666666 6666666666 6666666666 6666666666 6666666666 6666666666 6666666666 6666666666 6666666666 6666666666 6666666666
                               2222222222 2222222222 2222222223 3333333333 3333333333 3333333333 3333333333 3333333333 3333333333 3333333333 3333333333
                               7777777778 8888888889 9999999990 0000000001 1111111112 2222222223 3333333334 4444444445 5555555556 6666666667 7777777778
                               1234567890 1234567890 1234567890 1234567890 1234567890 1234567890 1234567890 1234567890 1234567890 1234567890 1234567890
Crocodylus_porosus_EU727191    TCTCACATCT GTGTCGCCCC AGCCTCCCTT TGGGCCGCAT GCCCCAGCAG GCTGGTCAGC TGGTCTGCGC CCCTTCCTCG TGTCCCCAGG TTTCCACCCT GGCCTCCTCA
Xenopus_laevis_X02995          GGGAGGGGAG GGGGGGAGGC GGCGGCGAGC CCGGCCGGGC GCCGCCGCCG CAGGACCCCC GTCCCCGTCC CGCGCCGCCC CCGCCGGCCC GGGGCGCCCG GGCCGCGACC
Mus_musculus_NR_046233         CGTCGGGGCG CGCGCTTTGC TCTCCCGGCA CCCATCCCCG CCGCGGCTCT GGCTTTTCTA CGTTGGCTGG GGCGGTTGTC GCGTGTGGGG GGATGTG--- -AGTGTCGCG
Homo_sapiens_U13369            GCCCTCCTCC CCGCCGGGGC CCGTCGTCCG GCCCCGCCGC GCCGGCTCCC CGTCTTCGGG GCCGGCCGGA TTCCCGTCGC CTCCGCCGCG CCGCTCCGCG CCGCCGGGCA
Rattus_norvegicus_NR_046239    CTCCTCGAGG CGCCTGCCGC TCCTCCCCCG CCGCGGCTCT CCG--GCACT GACGGTGC-- CGGTGGCTCC GCCGCGTGCG GTGGGGGGGG GTGTGTGTGC CGGTGCCGCG
Gallus_gallus_KT445934.2       CGCGGCCGAG TCCCGCTCCG ACCGCGGGGT CGGGGTCGGG AGGTGGCGGC GGTGCGGAGG GTGGAAGGAC GGCTCCCCGC TTCGTCGCTC GGCCGGAAAC TCGCCACCCG

                               6666666666 6666666666 6666666666 6666666666 6666666666 6666666666 6666666666 6666666666 6666666666 6666666666 6666666666
                               3333333333 3333333334 4444444444 4444444444 4444444444 4444444444 4444444444 4444444444 4444444444 4444444444 4444444444
                               8888888889 9999999990 0000000001 1111111112 2222222223 3333333334 4444444445 5555555556 6666666667 7777777778 8888888889
                               1234567890 1234567890 1234567890 1234567890 1234567890 1234567890 1234567890 1234567890 1234567890 1234567890 1234567890
Crocodylus_porosus_EU727191    GCCCAGCTGA GTGGGGGCAG GAAAGGGACT CAGGGTTTGG CGGGGTGCAT GGCTGTCAGC CACCACCATG GGCGGGCATC CGTGGTGGCT GGGATGGCTG GGATGGGATG
Xenopus_laevis_X02995          GCCTCAGCGG CAGCACCGGT AGCCCTGCCG AGACCGAAAA GGAAAACCGA CCGACGCGTC GGCGAGAGCT CG-------- ---------- ---------- ----------
Mus_musculus_NR_046233         TGTGGGCTCG CCCGTCCCGA TGCCACGCTT TTCTGGCCTC GCGTGTCCTC CCCGCTCCTG TCCCGGGTAC CTAGCTGTCG CGTTCCGGCG CGGAGGTTTA AGGACCCCGG
Homo_sapiens_U13369            CGGCCCCGCT CGCTCTCCCC GGCCTTCCCG CTAGGGCGTC TCGAGGGTCG GGGGCCGGAC GCCGGTCCCC TCCCCCGCCT CCTCGTCCGC CCCCCCGCCG TCCAGGTACC
```

```
Rattus_norvegicus_NR_046239    GGTGGGCTCG CGCGCCCCG- --CCGCGTCC TTCTGGGCCT CCGTGTCCGC CCCGCTCCTG TCC-GGGTAC CT-GCTCTCG CGTTCCGGCG CGGAGGTTTC GCGACCCCGG
Gallus_gallus_KT445934.2       CCCCCGCCGC TGTCGACGCC GGCACCCCGA GTCCGCTCGG AGGGAAGCCG CGCGGGCGGC CGCGCGCGGG GGAGGCGGCG GGCGGCGGGT CCGAGCGCGG GGCGCGGGAA

                               6666666666 6666666666 6666666666 6666666666 6666666666 6666666666 6666666666 6666666666 6666666666 6666666666 6666666666
                               4444444445 5555555555 5555555555 5555555555 5555555555 5555555555 5555555555 5555555555 5555555555 5555555555 5555555556
                               9999999990 0000000001 1111111112 2222222223 3333333334 4444444445 5555555556 6666666667 7777777778 8888888889 9999999990
                               1234567890 1234567890 1234567890 1234567890 1234567890 1234567890 1234567890 1234567890 1234567890 1234567890 1234567890
Crocodylus_porosus_EU727191    GGGAGGGCCC GTCTGGCGGG GCGGCCCCAG CTGTGTCAGC CCCCAGGGAA ACAGCTAAGA CCTGAGAGAA ACCCCAGCCC CCCAAATGGG AAGGCGCTGG CCATGGCAGT
Xenopus_laevis_X02995          ---------- ---------- ---------- ---------- ---------- ---------- ---------- ---------- ---------- ---------- ----------
Mus_musculus_NR_046233         GGGGGTCGCC CTGCCGCCCC CAGGGTCGGG GGG-CGGTGG GGCCCGTAGG GAAGTCGGTC GTTCGGG--- ---------- ---------- -----CGGCT CTCCCTCAGA
Homo_sapiens_U13369            TAGCGCGTTC CGGCGCGGAG GTTTAAAGAC CCCTTGGGGG GATCGCCCGT CCGCCCGTGG GTCGGGGGCG GTGGTGGGCC CGCGGGGGAG TCCCGTCGGG AGGGGCCCGG
Rattus_norvegicus_NR_046239    GGGGGTCGCC CTACCGTCCC CGGGGTCGGG GGGGCGGTGG GGCCCGTAGG GACGTCGGTC GTCCGCGGGA GGGCTTCCCG GTCCCTCCCC GCGTCCGGCG CTCCCTCAGA
Gallus_gallus_KT445934.2       GTCGGCCGCT TCCCCCGGCC TCACCCCCCA CCCCCTTCGC CCGGCCCGTC GCGGGGACGG GGCCGGGTCG CGGGCGGCTG CGGAGCCGGC CGACTCCGGG CGAGCGCCGG

                               6666666666 6666666666 6666666666 6666666666 6666666666 6666666666 6666666666 6666666666 6666666666 6666666666 6666666666
                               6666666666 6666666666 6666666666 6666666666 6666666666 6666666666 6666666666 6666666666 6666666666 6666666667 7777777777
                               0000000001 1111111112 2222222223 3333333334 4444444445 5555555556 6666666667 7777777778 8888888889 9999999990 0000000001
                               1234567890 1234567890 1234567890 1234567890 1234567890 1234567890 1234567890 1234567890 1234567890 1234567890 1234567890
Crocodylus_porosus_EU727191    GAGTCCTGGG CGCTGCCCTC CGGGTGGATG CTTCTCCCCG CCTATGGGTT CACAGAGCTG GCTGGAGGAT GCCAGGCTCA ACTCCACGTC CCCCTCCAGT GTCTGTCCCT
Xenopus_laevis_X02995          ---------- ---------- ---------- ---------- ---------- ---------- ---------- ---------- ---------- ---------- ----------
Mus_musculus_NR_046233         CTCCATGACC CTCCTCCCCC CGCTGCCGCC G-TTCCCGAG GCGGCGGTCG TGTGGGGGGG TGGATGTCTG GAGCCCCCTC -GGGCGCCGT GGGGG--CCC GACCCGCGCC
Homo_sapiens_U13369            CCCCTCCCGC GCCTCCACCG CGGACTCCGC TCCCCGGCCG GGGCCGCGCC GCCGCCGCCG CCGCGGCGGC CGTCGGGTGG GGGCTTTACC CGGCGGCCGT CGCGCGCCTG
Rattus_norvegicus_NR_046239    CTCAGCGGCG CACC--CCTC TGCCGCCGGC GCTTCCCGAG GCGGCGGTT- --CGGGGGGG CG--TGTCGC GAGCCCCCTC TGGGCGCCCG AGGGGTTCCC GGCCCGAGCG
Gallus_gallus_KT445934.2       AGGGACGCGC GCGCCGCGTA CGCGCGGCAG GCGCGAGGTG CCCCGGGCGG CTTCGGTCCC GCGCGGGCGG TCCGAGCCTC GCGGCTCCTC CCGGGTGCAG CTGCCGCCCG

                               6666666666 6666666666 6666666666 6666666666 6666666666 6666666666 6666666666 6666666666 6666666666 6666666666 6666666666
                               7777777777 7777777777 7777777777 7777777777 7777777777 7777777777 7777777777 7777777777 7777777778 8888888888 8888888888
                               1111111112 2222222223 3333333334 4444444445 5555555556 6666666667 7777777778 8888888889 9999999990 0000000001 1111111112
                               1234567890 1234567890 1234567890 1234567890 1234567890 1234567890 1234567890 1234567890 1234567890 1234567890 1234567890
Crocodylus_porosus_EU727191    TGCTGTCCCA TCTGCGGAGG CGAGGCATTG TCCGGAAGGT GGGGGTTGGG ACCACCTGGA GTTCAGAACC ATTTCCCTCA CCCAAGCTAC CAGGTACCTA GTGCTCCACC
Xenopus_laevis_X02995          ---------- ---------- ---------- ---------- ---------- ---------- ---------- ---------- ---------- ---------- ----------
Mus_musculus_NR_046233         GCCGGCTTGC CCGATT--TC CGCGGGTCGG TCCTGTCGGT GCCGG--TCG TGGGTTCCCG TGTCGT---T CCCGTGTTTT TCCGCTCCCG ACCCT--TTT TTTTTCCTCC
Homo_sapiens_U13369            CCGCGCGTGT GGCGTGCGCC CCGCGCCGTG GGGGCGGGAA CCCCCGGGCG CCTGTGGGGT GGTGTCCGCG CTCGCCCCCG CGTGGGCGGC GCGCGCCTCC CCGTGGTGTG
Rattus_norvegicus_NR_046239    CCTGGCTTGT CCCGCCCGTT GGCGGGCCGC CGCCG--GGT GCCGGGTTCG TGGCGTCCCG TGTCCCGTGT GTCCCGCCTT TCCGTCTCCG ACCCTGTCTT TTTTTTTTTA
Gallus_gallus_KT445934.2       GCGCCGGGTT GCCGAGGGAA ACCCCGGGCC CCGGGAGGAA CGCGAGGTGG TGGCGGCGGA CGTCGGGCGC GCCCCCGCGG GCGGACGCTC CCCCGAGGGG CGCCGGGGCC

                               6666666666 6666666666 6666666666 6666666666 6666666666 6666666666 6666666666 6666666666 6666666666 6666666666 6666666666
                               8888888888 8888888888 8888888888 8888888888 8888888888 8888888888 8888888888 8888888889 9999999999 9999999999 9999999999
                               2222222223 3333333334 4444444445 5555555556 6666666667 7777777778 8888888889 9999999990 0000000001 1111111112 2222222223
                               1234567890 1234567890 1234567890 1234567890 1234567890 1234567890 1234567890 1234567890 1234567890 1234567890 1234567890
Crocodylus_porosus_EU727191    CCTCAACTCG CCCTGCTCCA CGGGGCAGGC AGCGGGGTGG GTGGGCGAAG GTTCAAAGAT TTGTGCGTCC TGAGGGGTCT GCGCGGGCAG CCACAGGAGG TCTGCCGAGG
Xenopus_laevis_X02995          ---------- ---------- ---------- ---------- ---------- ---------- ---------- ---------- ---------- ---------- ----------
Mus_musculus_NR_046233         CCCCCACACG TGTCTCGTTT CGTTCC-TGC TGGCCGGCCT GAGGCTA--- CCCCTCG--G TCCATCTG-- TTCTCCTCTC TCTCCGGGGA GAGGAGGGCG GTGGTCGTTG
Homo_sapiens_U13369            AAACCTTCCG ACCCCTCTCC GGAGTCCGGT CCCGTTTGCT GTCTCGTCTG GCCGGCCTGA GGCAACCCCC TCTCCTCTTG GGCGGGGGGG GCGGGGGGAC GTGCCGCGCC
Rattus_norvegicus_NR_046239    TTTTCTTACG TGTGCCGTTT CGTTTCGCGC TGGCCGGCCT GAGGCGGAGC CCCCTCGCCG TCCGTCGGGC CCCTCCCATC CCGCGCAGGA GGGGCGGGCG GGGGCGCTTG
Gallus_gallus_KT445934.2       GGCTGGCGGG TGCCGGGTCT CCCCTCGGCG CCCCGTCCCG CCCCGCCGAG CGGGGCGGGC GGGGGAGGCA CCCCCGCGGG GCCTTCGGGT CGTTTCCCTC ACCCCAGGGC

                               6666666666 6666666666 6666666666 6666666666 6666666666 6666666666 6666666667 7777777777 7777777777 7777777777 7777777777
                               9999999999 9999999999 9999999999 9999999999 9999999999 9999999999 9999999990 0000000000 0000000000 0000000000 0000000000
                               3333333334 4444444445 5555555556 6666666667 7777777778 8888888889 9999999990 0000000001 1111111112 2222222223 3333333334
                               1234567890 1234567890 1234567890 1234567890 1234567890 1234567890 1234567890 1234567890 1234567890 1234567890 1234567890
Crocodylus_porosus_EU727191    GAGGGTGGGC ATGTGCAGAT GTGCCTGTGC CTTTGACACT CCCTGCCCAT GTGGCTCCTG GAATGGAGAG GGGTCACCCT GCCTCCCTCT CTGCAGAGGG GCCTGAAAGC
Xenopus_laevis_X02995          ---------- ---------- ---------- ---------- ---------- ---------- ---------- ---------- ---------- ---------- ----------
Mus_musculus_NR_046233         GGGGACTGTG CCGTCGTCAG CACCCGTGAG TTCGCTCACA CCCGAAATAC CGATA----- ---------- ---------- ---------- ---------- ----------
```

```
Homo_sapiens_U13369            AGGAAGGGCC TCCTCCCGGT GCGTCGTCGG GAGCGCCCTC GCCAAATCGA CCTCGTA--- ---------- ---------- ---------- ---------- ----------
Rattus_norvegicus_NR_046239    TTGCGGTCGT CAGCACCCCG TGTGCGGAGT TCCGCTCACA CCTCAGATAA CCGTA----- ---------- ---------- ---------- ---------- ----------
Gallus_gallus_KT445934.2       CAGGTACCTA GCGTCCGCGC CTCCGCGCGT CCGGGGGGCG GGGAGGAAGG AGCGCGGCGC CGGTCCCGAG CGGGCCGCGT CGCCCACACC CCCCTCCTCC CCCCGGGCCG

                               7777777777 7777777777 7777777777 7777777777 7777777777 7777777777 7777777777 7777777777 7777777777 7777777777 7777777777
                               0000000000 0000000000 0000000000 0000000000 0000000000 0000000001 1111111111 1111111111 1111111111 1111111111 1111111111
                               4444444445 5555555556 6666666667 7777777778 8888888889 9999999990 0000000001 1111111112 2222222223 3333333334 4444444445
                               1234567890 1234567890 1234567890 1234567890 1234567890 1234567890 1234567890 1234567890 1234567890 1234567890 1234567890
Crocodylus_porosus_EU727191    TGATTGCTTG TGGGCTGTGG CTCCCTTGCC AATGTCATCC ACCCTGCCTA TCGCCATCAC CCCCACTGCA CCCCTGGGTG GGGCAAGGGC TGGTCAAGGG GAGCGGAAGG
Xenopus_laevis_X02995          ---------- ---------- ---------- ---------- ---------- ---------- ---------- ---------- ---------- ---------- ----------
Mus_musculus_NR_046233         ---------- ---------- ---------- ---------- ---------- ---------- ---------- ---------- ---------- ---------- ----------
Homo_sapiens_U13369            ---------- ---------- ---------- ---------- ---------- ---------- ---------- ---------- ---------- ---------- ----------
Rattus_norvegicus_NR_046239    ---------- ---------- ---------- ---------- ---------- ---------- ---------- ---------- ---------- ---------- ----------
Gallus_gallus_KT445934.2       CGGAGCCGGG CGGAGGTTTA AAGACTCGGG CGGCCCGCGG CGCGCGCCGC GAGGTCGGGG GCCGGGGGCG GTCTTCTGCC CGCCGGCGGG ACGCCGGGAT GGAAGAGAGG

                               7777777777 7777777777 7777777777 7777777777 7777777777 7777777777 7777777777 7777777777 7777777777 7777777777 7777777777
                               1111111111 1111111111 1111111111 1111111111 1111111112 2222222222 2222222222 2222222222 2222222222 2222222222 2222222222
                               5555555556 6666666667 7777777778 8888888889 9999999990 0000000001 1111111112 2222222223 3333333334 4444444445 5555555556
                               1234567890 1234567890 1234567890 1234567890 1234567890 1234567890 1234567890 1234567890 1234567890 1234567890 1234567890
Crocodylus_porosus_EU727191    GGTGCATGGT CCCAGGTGCA GGCGCAGGAG CGCTGCGGAG CTGCTGGAGC CTGTGGCACG GCTGGCCGTG CCCCCCTAAG CTGAGCACCT GGACTGAGCC TGCAGCGGGG
Xenopus_laevis_X02995          ---------- ---------- ---------- ---------- ---------- ---------- ---------- ---------- ---------- ---------- ----------
Mus_musculus_NR_046233         ---------- ---------- ---------- ---------- ---------- ---------- ---------- ---------- ---------- ---------- ----------
Homo_sapiens_U13369            ---------- ---------- ---------- ---------- ---------- ---------- ---------- ---------- ---------- ---------- ----------
Rattus_norvegicus_NR_046239    ---------- ---------- ---------- ---------- ---------- ---------- ---------- ---------- ---------- ---------- ----------
Gallus_gallus_KT445934.2       ACTCCGGGCG GGGCGCGGCG GCGCGCCCCG CCGGCCCTCT CCCTCCCGAG CCCGCCGGCG GCGTCGGCCG TCGCCGCGCC CTCGGTCCTC CGCGGGGCGG GCCCGGGCCG

                               7777777777 7777777777 7777777777 7777777777 7777777777 7777777777 7777777777 7777777777 7777777777 7777777777 7777777777
                               2222222222 2222222222 2222222222 2222222223 3333333333 3333333333 3333333333 3333333333 3333333333 3333333333 3333333333
                               6666666667 7777777778 8888888889 9999999990 0000000001 1111111112 2222222223 3333333334 4444444445 5555555556 6666666667
                               1234567890 1234567890 1234567890 1234567890 1234567890 1234567890 1234567890 1234567890 1234567890 1234567890 1234567890
Crocodylus_porosus_EU727191    GGCTCACCTT TGTGGCCATG GCCCTCTCTC AGGTTGTTAA ACCTCTCTCC TGTGTTTGCC TGGTCTGTAG GACTCCTGCC AAGGGAAGGC AGACAACCGG CTGGGCAGGA
Xenopus_laevis_X02995          ---------- ---------- ---------- ---------- ---------- ---------- ---------- ---------- ---------- ---------- ----------
Mus_musculus_NR_046233         ---------- ---------- ---------- ---------- ---------- ---------- ---------- ---------- ---------- ---------- ----------
Homo_sapiens_U13369            ---------- ---------- ---------- ---------- ---------- ---------- ---------- ---------- ---------- ---------- ----------
Rattus_norvegicus_NR_046239    ---------- ---------- ---------- ---------- ---------- ---------- ---------- ---------- ---------- ---------- ----------
Gallus_gallus_KT445934.2       GAGAGGGGGT CATCCCGTCC CCCCTCTCCG CGGCCTCGGT CTCGGGCGGA GAGCTCGGCG CGCGCGCGGG CGCGCGCTCG CTCCGGCCGG CCTCGCCCGC GTACGGAGCG

                               7777777777 7777777777 7777777777 7777777777 7777777777 7777777777 7777777777 7777777777 7777777777 7777777777 7777777777
                               3333333333 3333333333 3333333334 4444444444 4444444444 4444444444 4444444444 4444444444 4444444444 4444444444 4444444444
                               7777777778 8888888889 9999999990 0000000001 1111111112 2222222223 3333333334 4444444445 5555555556 6666666667 7777777778
                               1234567890 1234567890 1234567890 1234567890 1234567890 1234567890 1234567890 1234567890 1234567890 1234567890 1234567890
Crocodylus_porosus_EU727191    ATACCTCCCC TCCCATGACC CCTGCCAGGT TGCTGCAGCA GGGGAGGAAG GTCAGCTCAG GGACCCTGTC CTGACCTCGT GCAGACCCAG AAGCCCACCC TCCCTCTGGC
Xenopus_laevis_X02995          ---------- ---------- ---------- ---------- ---------- ---------- ---------- ---------- ---------- ---------- ----------
Mus_musculus_NR_046233         ---------- ---------- ---------- ---------- ---------- ---------- ---------- ---------- ---------- ---------- ----------
Homo_sapiens_U13369            ---------- ---------- ---------- ---------- ---------- ---------- ---------- ---------- ---------- ---------- ----------
Rattus_norvegicus_NR_046239    ---------- ---------- ---------- ---------- ---------- ---------- ---------- ---------- ---------- ---------- ----------
Gallus_gallus_KT445934.2       GGCCGAGACG CGGGTCTCGG CCCGGCGCCC GCCGCTCCCC GCGGCGGTGC GTTCCGCGGC CTCCCCGCCG CGCGCGGCCG GCGGGACGGC GAGCCGGCCG TCCCGCCCGC

                               7777777777 7777777777 7777777777 7777777777 7777777777 7777777777 7777777777 7777777777 7777777777 7777777777 7777777777
                               4444444444 4444444445 5555555555 5555555555 5555555555 5555555555 5555555555 5555555555 5555555555 5555555555 5555555555
                               8888888889 9999999990 0000000001 1111111112 2222222223 3333333334 4444444445 5555555556 6666666667 7777777778 8888888889
                               1234567890 1234567890 1234567890 1234567890 1234567890 1234567890 1234567890 1234567890 1234567890 1234567890 1234567890
Crocodylus_porosus_EU727191    TTGGCTTCTG CCATGCGGCG AGGTGGCAAC CGTGGAGAAA AGCCTGTGCA AAAAAAGTGA ---------- ---------- ---------- ---------- ----------
Xenopus_laevis_X02995          ---------- ---------- ---------- ---------- ---------- ---------- ---------- ---------- ---------- ---------- ----------
```

```
Mus_musculus_NR_046233          ---------- ---------- ---------- ---------- ---------- ---------- ---------- ---------- ---------- ---------- ----------
Homo_sapiens_U13369             ---------- ---------- ---------- ---------- ---------- ---------- ---------- ---------- ---------- ---------- ----------
Rattus_norvegicus_NR_046239     ---------- ---------- ---------- ---------- ---------- ---------- ---------- ---------- ---------- ---------- ----------
Gallus_gallus_KT445934.2        GCCAGCCGCG GCGCCGGCGG TTCCGCTCCG CCGGTCCGCC CCGCGTGCGT CCGCACGCCC GGCCTCCTGC CCTCCCTCGG GGCCTCGCCG CCGTTTCCCC CTTCCGTCGC

                                7777777777 7777777777 7777777777 7777777777 7777777777 7777777777 7777777777 7777777777 7777777777 7777777777 7777777777
                                5555555556 6666666666 6666666666 6666666666 6666666666 6666666666 6666666666 6666666666 6666666666 6666666666 6666666667
                                9999999990 0000000001 1111111112 2222222223 3333333334 4444444445 5555555556 6666666667 7777777778 8888888889 9999999990
                                1234567890 1234567890 1234567890 1234567890 1234567890 1234567890 1234567890 1234567890 1234567890 1234567890 1234567890
Crocodylus_porosus_EU727191     ---------- ---------- ---------- ---------- ---------- ---------- ---------- ---------- ---------- ---------- ----------
Xenopus_laevis_X02995           ---------- ---------- ---------- ---------- ---------- ---------- ---------- ---------- ---------- ---------- ----------
Mus_musculus_NR_046233          ---------- ---------- ---------- ---------- ---------- ---------- ---------- ---------- ---------- ---------- ----------
Homo_sapiens_U13369             ---------- ---------- ---------- ---------- ---------- ---------- ---------- ---------- ---------- ---------- ----------
Rattus_norvegicus_NR_046239     ---------- ---------- ---------- ---------- ---------- ---------- ---------- ---------- ---------- ---------- ----------
Gallus_gallus_KT445934.2        AAGCCGCGTC CTCTCCTTCG TCCCCGCCGC CGTCGCCTCC CACCGCGCTT TCGCCCTCGG CCTCGCCGGC CGCGCCGGTC GTGCGAGCGG GAGGTCCGGC GTGGGGCGTC

                                7777777777 7777777777 7777777777 7777777777 7777777777 7777777777 7777777777 7777777777 7777777777 7777777777 7777777777
                                7777777777 7777777777 7777777777 7777777777 7777777777 7777777777 7777777777 7777777777 7777777777 7777777778 8888888888
                                0000000001 1111111112 2222222223 3333333334 4444444445 5555555556 6666666667 7777777778 8888888889 9999999990 0000000001
                                1234567890 1234567890 1234567890 1234567890 1234567890 1234567890 1234567890 1234567890 1234567890 1234567890 1234567890
Crocodylus_porosus_EU727191     ---------- ---------- ---------- ---------- ---------- ---------- ---------- ---------- ---------- ---------- ----------
Xenopus_laevis_X02995           ---------- ---------- ---------- ---------- ---------- ---------- ---------- ---------- ---------- ---------- ----------
Mus_musculus_NR_046233          ---------- ---------- ---------- ---------- ---------- ---------- ---------- ---------- ---------- ---------- ----------
Homo_sapiens_U13369             ---------- ---------- ---------- ---------- ---------- ---------- ---------- ---------- ---------- ---------- ----------
Rattus_norvegicus_NR_046239     ---------- ---------- ---------- ---------- ---------- ---------- ---------- ---------- ---------- ---------- ----------
Gallus_gallus_KT445934.2        CCGCAGCCGG TCTCCGCGCG GAGGCGCGGG GAGCGGGCGC CGCTCCCGAA TCCGTCCCCG TCCCGCCCGC CGCCGTGCGC GCGTCCGCCG CGGGCGCGCC GCCAGGGCGA

                                7777777777 7777777777 7777777777 7777777777 7777777777 7777777777 7777777777 7777777777 7777777777 7777777777 7777777777
                                8888888888 8888888888 8888888888 8888888888 8888888888 8888888888 8888888888 8888888888 8888888889 9999999999 9999999999
                                1111111112 2222222223 3333333334 4444444445 5555555556 6666666667 7777777778 8888888889 9999999990 0000000001 1111111112
                                1234567890 1234567890 1234567890 1234567890 1234567890 1234567890 1234567890 1234567890 1234567890 1234567890 1234567890
Crocodylus_porosus_EU727191     ---------- ---------- ---------- ---------- ---------- ---------- ---------- ---------- ---------- ---------- ----------
Xenopus_laevis_X02995           ---------- ---------- ---------- ---------- ---------- ---------- ---------- ---------- ---------- ---------- ----------
Mus_musculus_NR_046233          ---------- ---------- ---------- ---------- ---------- ---------- ---------- ---------- ---------- ---------- ----------
Homo_sapiens_U13369             ---------- ---------- ---------- ---------- ---------- ---------- ---------- ---------- ---------- ---------- ----------
Rattus_norvegicus_NR_046239     ---------- ---------- ---------- ---------- ---------- ---------- ---------- ---------- ---------- ---------- ----------
Gallus_gallus_KT445934.2        GCGAGAGGAG GAGGCGTCGG AGGACGAGGG GCGGGGGAGG AAGGTGAGAG GCGGCGGGGG CGTTTCGGTG CGCGCGTCTC CCGCACGGCG AGGAAGGGGC CGAGGTCGGC

                                7777777777 7777777777 7777777777 7777777777 7777777777 7777777777 7777777777 7777777778 8888888888 8888888888 8888888888
                                9999999999 9999999999 9999999999 9999999999 9999999999 9999999999 9999999999 9999999990 0000000000 0000000000 0000000000
                                2222222223 3333333334 4444444445 5555555556 6666666667 7777777778 8888888889 9999999990 0000000001 1111111112 2222222223
                                1234567890 1234567890 1234567890 1234567890 1234567890 1234567890 1234567890 1234567890 1234567890 1234567890 1234567890
Crocodylus_porosus_EU727191     ---------- ---------- ---------- ---------- ---------- ---------- ---------- ---------- ---------- ---------- ----------
Xenopus_laevis_X02995           ---------- ---------- ---------- ---------- ---------- ---------- ---------- ---------- ---------- ---------- ----------
Mus_musculus_NR_046233          ---------- ---------- ---------- ---------- ---------- ---------- ---------- ---------- ---------- ---------- ----------
Homo_sapiens_U13369             ---------- ---------- ---------- ---------- ---------- ---------- ---------- ---------- ---------- ---------- ----------
Rattus_norvegicus_NR_046239     ---------- ---------- ---------- ---------- ---------- ---------- ---------- ---------- ---------- ---------- ----------
Gallus_gallus_KT445934.2        GCGGGCGCCG TCGGGCGGTC CGGCGCGGGC GCGGGCCGGC GGCGCCGCCG GCGCGGGCGG GGGCCTGGTC TGCTCCCGTC CCCGTCGGTC GCGGCGGCGG CGGCGGCGGC
```

```
                            8888888888 8888888888 8888888888 8888888888 8888888888 8888888888 8888888888 8888888888 8888888888 8888888888 8888888888
                            0000000000 0000000000 0000000000 0000000000 0000000000 0000000000 0000000001 1111111111 1111111111 1111111111 1111111111
                            3333333334 4444444445 5555555556 6666666667 7777777778 8888888889 9999999990 0000000001 1111111112 2222222223 3333333334
                            1234567890 1234567890 1234567890 1234567890 1234567890 1234567890 1234567890 1234567890 1234567890 1234567890 1234567890
Crocodylus_porosus_EU727191 ---------- ---------- ---------- ---------- ---------- ---------- ---------- ---------- ---------- ---------- ----------
Xenopus_laevis_X02995       ---------- ---------- ---------- ---------- ---------- ---------- ---------- ---------- ---------- ---------- ----------
Mus_musculus_NR_046233      ---------- ---------- ---------- ---------- ---------- ---------- ---------- ---------- ---------- ---------- ----------
Homo_sapiens_U13369         ---------- ---------- ---------- ---------- ---------- ---------- ---------- ---------- ---------- ---------- ----------
Rattus_norvegicus_NR_046239 ---------- ---------- ---------- ---------- ---------- ---------- ---------- ---------- ---------- ---------- ----------
Gallus_gallus_KT445934.2    GGTCCGTCGC GGCAGCGGGG CTTCGGCCGG GGCGGCGCGC GCCGTCCCGC GGGCGTCCGC GGCTCCTCCG CCCGGGCCGG GCCGAGCCGG GCGCCTGGTC CGTCCCCGAA

                            8888888888 8888888888 8888888888 8888888888 8888888888 8888888888 8888888888 8888888888 8888888888 8888888888 8888888888
                            1111111111 1111111111 1111111111 1111111111 1111111111 1111111112 2222222222 2222222222 2222222222 2222222222 2222222222
                            4444444445 5555555556 6666666667 7777777778 8888888889 9999999990 0000000001 1111111112 2222222223 3333333334 4444444445
                            1234567890 1234567890 1234567890 1234567890 1234567890 1234567890 1234567890 1234567890 1234567890 1234567890 1234567890
Crocodylus_porosus_EU727191 ---------- ---------- ---------- ---------- ---------- ---------- ---------- ---------- ---------- ---------- ----------
Xenopus_laevis_X02995       ---------- ---------- ---------- ---------- ---------- ---------- ---------- ---------- ---------- ---------- ----------
Mus_musculus_NR_046233      ---------- ---------- ---------- ---------- ---------- ---------- ---------- ---------- ---------- ---------- ----------
Homo_sapiens_U13369         ---------- ---------- ---------- ---------- ---------- ---------- ---------- ---------- ---------- ---------- ----------
Rattus_norvegicus_NR_046239 ---------- ---------- ---------- ---------- ---------- ---------- ---------- ---------- ---------- ---------- ----------
Gallus_gallus_KT445934.2    GCGAGACAGG GTCGTTTCCC CAGGTCGGGA GCGAGGGCTC CCCGCCCTTC TCGTTCGGGT CGCGCTTCAT TGCCGGCCGG CCGGCCGGCC GTCGCCGGCT TTTTTTTTCC

                            8888888888 8888888888 8888888888 8888888888 8888888888 8888888888 8888888888 8888888888 8888888888 8888888888 8888888888
                            2222222222 2222222222 2222222222 2222222222 2222222223 3333333333 3333333333 3333333333 3333333333 3333333333 3333333333
                            5555555556 6666666667 7777777778 8888888889 9999999990 0000000001 1111111112 2222222223 3333333334 4444444445 5555555556
                            1234567890 1234567890 1234567890 1234567890 1234567890 1234567890 1234567890 1234567890 1234567890 1234567890 1234567890
Crocodylus_porosus_EU727191 ---------- ---------- ---------- ---------- ---------- ---------- ---------- ---------- ---------- ---------- ----------
Xenopus_laevis_X02995       ---------- ---------- ---------- ---------- ---------- ---------- ---------- ---------- ---------- ---------- ----------
Mus_musculus_NR_046233      ---------- ---------- ---------- ---------- ---------- ---------- ---------- ---------- ---------- ---------- ----------
Homo_sapiens_U13369         ---------- ---------- ---------- ---------- ---------- ---------- ---------- ---------- ---------- ---------- ----------
Rattus_norvegicus_NR_046239 ---------- ---------- ---------- ---------- ---------- ---------- ---------- ---------- ---------- ---------- ----------
Gallus_gallus_KT445934.2    CTCCCGCATC CGATATTCGT GTGCTCGTAC GGTCAGCGGA GGCGACGCTC GTCCGCCCCG CGGTCGCCCC GGCGTCGGGG CTGGCCGCGG GCGCGGGCCG AGCGCCTTCG

                            8888888888 8888888888 8888888888 8888888888 8888888888 8888888888 8888888888 8888888888 8888888888 8888888888 8888888888
                            3333333333 3333333333 3333333333 3333333334 4444444444 4444444444 4444444444 4444444444 4444444444 4444444444 4444444444
                            6666666667 7777777778 8888888889 9999999990 0000000001 1111111112 2222222223 3333333334 4444444445 5555555556 6666666667
                            1234567890 1234567890 1234567890 1234567890 1234567890 1234567890 1234567890 1234567890 1234567890 1234567890 1234567890
Crocodylus_porosus_EU727191 ---------- ---------- ---------- ---------- ---------- ---------- -----CAACT CTCAGCGGTG GATCACTTGG CTCATGCATT GATGAAGAAT
Xenopus_laevis_X02995       ---------- ---------- ---------- ---------- ---------- ---------- -----CGACT CTTAGCGGTG GATCACTCGG CTCGTGCGTC GATGAAGAAC
Mus_musculus_NR_046233      ---------- ---------- ---------- ---------- ---------- ---------- -----CGACT CTTAGCGGTG GATCACTCGG CTCGTGCGTC GATGAAGAAC
Homo_sapiens_U13369         ---------- ---------- ---------- ---------- ---------- ---------- -----CGACT CTTAGCGGTG GATCACTCGG CTCGTGCGTC GATGAAGAAC
Rattus_norvegicus_NR_046239 ---------- ---------- ---------- ---------- ---------- ---------- -----CGACT CTTAGCGGTG GATCACTCGG CTCGTGCGTC GATGAAGAAC
Gallus_gallus_KT445934.2    GGCAAGGCGA GAGAGAACGA GAGCGGTCCC CCCGCGCGCG CGGGGCGGTG CCGAAAGTCA GA---CAACT CTTAGCGGTG GATCACTCGG CTCGTGCGTC GATGAAGAAC

                            8888888888 8888888888 8888888888 8888888888 8888888888 8888888888 8888888888 8888888888 8888888888 8888888888 8888888888
                            4444444444 4444444444 4444444445 5555555555 5555555555 5555555555 5555555555 5555555555 5555555555 5555555555 5555555555
                            7777777778 8888888889 9999999990 0000000001 1111111112 2222222223 3333333334 4444444445 5555555556 6666666667 7777777778
                            1234567890 1234567890 1234567890 1234567890 1234567890 1234567890 1234567890 1234567890 1234567890 1234567890 1234567890
Crocodylus_porosus_EU727191 CCAGCTATCT GCAAGAATTA ATGTGATTTG CAGGACACAC TGATCATTGA CACTTCGAAC GCAC-TTGCG GCCCTGGGTT CCTCCTGGGG CTATGCCGGT CTGAGCATCG
Xenopus_laevis_X02995       GCAGCTAGCT GCGAGAATTA GTGTGAATTG CAGGACACAT TGATCATCGA CACTTCGAAC GCACCTTGCG GCCCCGGGTT CCTCCCGGGG CCACGCCTGT CTGAGGGTCG
Mus_musculus_NR_046233      GCAGCTAGCT GCGAGAATTA ATGTGAATTG CAGGACACAT TGATCATCGA CACTTCGAAC GCAC-TTGCG GCCCCGGGTT CCTCCCGGGG CTACGCCTGT CTGAGCGTCG
Homo_sapiens_U13369         GCAGCTAGCT GCGAGAATTA ATGTGAATTG CAGGACACAT TGATCATCGA CACTTCGAAC GCAC-TTGCG GCCCCGGGTT CCTCCCGGGG CTACGCCTGT CTGAGCGTCG
Rattus_norvegicus_NR_046239 GCAGCTAGCT GCGAGAATTA ATGTGAATTG CAGGACACAT TGATCATCGA CACTTCGAAC GCAC-TTGCG GCCCCGGGTT CCTCCCGGGG CTACGCCTGT CTGAGCGTCG
Gallus_gallus_KT445934.2    GCAGCTAGCT GCGAGAATTA ATGTGAATTG CAGGACACAT TGATCATCGA CACTTCGAAC GCAC-TTGCG GCCCCGGGTT CCTCCCGGGG CTACGCCTGC CTGAGCGTCG
```

```
                            8888888888 8888888888 8888888888 8888888888 8888888888 8888888888 8888888888 8888888888 8888888888 8888888888 8888888888
                            5555555555 5555555556 6666666666 6666666666 6666666666 6666666666 6666666666 6666666666 6666666666 6666666666 6666666666
                            8888888889 9999999990 0000000001 1111111112 2222222223 3333333334 4444444445 5555555556 6666666667 7777777778 8888888889
                            1234567890 1234567890 1234567890 1234567890 1234567890 1234567890 1234567890 1234567890 1234567890 1234567890 1234567890
Crocodylus_porosus_EU727191 CTT---GAAG GTCAATCACC TATGCGGTGT GTGTGAGGCC AGTGATGCTG GCTGTCCGGT CCCCGCTGCC ACTGAGTGCA GCTAGGGTGT CTCGCAGGCA ACCCGAGGTT
Xenopus_laevis_X02995       CTC---CGAC GTCCATCGCC CCCGCCGGGT CCCGTCCCGG CGCGGAGGCG CGGCTGGGGC CGTCGCAGGG GCGCGCCGCT CCCCTTCGTC CCCCCAAGGC CAGACCCCCG
Mus_musculus_NR_046233      GTT---GACG ATCAATCGCG TCACCCGCTG CGGT------ -------GGG ------TGCT GCGCGGCTGG GAGTTTGCTC GCAGGGCCAA CCCCCCAACC CGGGTCGGGC
Homo_sapiens_U13369         CTT---GCCG ATCAATCGCC CCGGGGGTGC CTCCGGGCTC CTCGGGGTGC GCGGCTGGGG GTTCCCTCGC AGGGCCCGCC GGGGGCCCTC CGTCCCCCTA AGCGCAGACC
Rattus_norvegicus_NR_046239 CTT---GACG ATCAATCGCC GCCCCCCTTG CGGTTTCGCC GCCGCGGGGG GGATCGCGTC GCGCGGCTGG GAGTCTGCTC GCAGGGCCCG CTCCCCG--- ------GGGC
Gallus_gallus_KT445934.2    CTT---GACG GTCAATCGCC GATGGCCGCC GTCCGCGGCG GCCGCGCGGC GCGGCTGGGG CGCCTCGCAG GCCCGCGCGC CCCGCCGGAG GCGGGTCGCG AGGGGGGGGG

                            8888888888 8888888888 8888888888 8888888888 8888888888 8888888888 8888888888 8888888888 8888888888 8888888888 8888888888
                            6666666667 7777777777 7777777777 7777777777 7777777777 7777777777 7777777777 7777777777 7777777777 7777777777 7777777778
                            9999999990 0000000001 1111111112 2222222223 3333333334 4444444445 5555555556 6666666667 7777777778 8888888889 9999999990
                            1234567890 1234567890 1234567890 1234567890 1234567890 1234567890 1234567890 1234567890 1234567890 1234567890 1234567890
Crocodylus_porosus_EU727191 CCTTGGGCCC GCCTCTGCTT GCCCTTGTTG GGGAAGAGGG GCAGCCTGAG CCCCTGAATG CCTTCGTCCC CCTAAGGTCA GACATGATGC CTTAAGAGTG CCTGCCTCAG
Xenopus_laevis_X02995       GCCCGGCGCC CGGGCCCCGG CCCGGCCGGC GGCGGCTGTC TGTGGATCCC TTCACGGCTG CCGCCCCGGC CGGCCCCCCG GGGCCCCGGC CCGCCGGCGG GAGCGGGCCC
Mus_musculus_NR_046233      CCTCCGTCTC CCGAAGTTCA GACGTGTGGG CGGTTGTCGG TGTGGCGCGC GCGCCCGCGT CGCGGAGCCT GGTCTCCCCC GCGCATCCGC GCTCGCGGCT TCTTCCCGCT
Homo_sapiens_U13369         CGGCGGCGTC CGCCCTCCTC TTGCCGCCGC GCCCGCCCCT TCCCCCTCCC CCCGCGGGCC CTGCGTGGTC ACGCGTCGGG TGGCGGGGGG GAGAGGGGGG CGCGCCCGGC
Rattus_norvegicus_NR_046239 CCTCCGTCTC CCGAAGTTCC GACGTGGG-- -------CGG CGTCCGGCGT TCGCCCGCG- ---------- ------CCCC GTGGGTCCGC GCGGGCTCGT CCTCCCTGC-
Gallus_gallus_KT445934.2    CCGCCGTCCG TCCGTCCGTC CGCCCGTCGG TCGGTCGGTT CGGGCGCCCG GATTCCCTCC CCCGCACCCC CTCCGAGCGG CGTCGCGCCG CGGGCCTTCG TCCCCCTAAG

                            8888888888 8888888888 8888888888 8888888888 8888888888 8888888888 8888888888 8888888888 8888888888 8888888888 8888888888
                            8888888888 8888888888 8888888888 8888888888 8888888888 8888888888 8888888888 8888888888 8888888888 8888888889 9999999999
                            0000000001 1111111112 2222222223 3333333334 4444444445 5555555556 6666666667 7777777778 8888888889 9999999990 0000000001
                            1234567890 1234567890 1234567890 1234567890 1234567890 1234567890 1234567890 1234567890 1234567890 1234567890 1234567890
Crocodylus_porosus_EU727191 GGAGCTCAAC CTGCTCGTGG AGGACTGTTG TAGCAGTCTG ACCCATGCTT CAGCTGGGTC GGCCATGGCG CCCGCTCCTG GGGTTCCAGG GGGAACCAGG CCTACCCCGT
Xenopus_laevis_X02995       GGCCCCCCCC CCCGGGCCGC GGCCCCGCGC CCCCCCCCCC CCCCACGAC- ---------- ---------- ---------- ---------- ---------- ----------
Mus_musculus_NR_046233      CCGCCGTTCC CGCCCTCGCC CGTGCACCCC GGTCCTGGCC TCGCGTCGGC GCCTCCCGGA CCGCTGCCTC ACCAGTCTTT CTCGGTCCCG TGCCCCGTGG GAACCCACCG
Homo_sapiens_U13369         TGAGAGAGAC GGGGAGGGCG GCGCCGCCGC CGGAAGACGG AGAGGGAAAG AGAGAGCCGG CTCGGGCCGA GTTCCCGTGG CCGCCGCCTG CGGTCCGGGT TCCTCCCTCG
Rattus_norvegicus_NR_046239 CCGCCGTCGT CGTGC--GGC CGGGC----- GGCCCT--CC TCGCGGC--T CCTTCCCCGC GCGCTCCGCC GC-------T CCCGCTCCCG TCCTC----- --GCCCGCT-
Gallus_gallus_KT445934.2    TGGAGACCCA GGTCGGGGAG CTCGCCGAGC TCCCCGCGCT CCCGGAGCGC CCGCTTTGGC CGAGCTCGTC CCCACGGGGC GGCCGGGCTT TCCGGTCGGT CGCGCGGCGC

                            8888888888 8888888888 8888888888 8888888888 8888888888 8888888888 8888888888 8888888888 8888888889 9999999999 9999999999
                            9999999999 9999999999 9999999999 9999999999 9999999999 9999999999 9999999999 9999999999 9999999990 0000000000 0000000000
                            1111111112 2222222223 3333333334 4444444445 5555555556 6666666667 7777777778 8888888889 9999999990 0000000001 1111111112
                            1234567890 1234567890 1234567890 1234567890 1234567890 1234567890 1234567890 1234567890 1234567890 1234567890 1234567890
Crocodylus_porosus_EU727191 GCGGCTGTCT CAGGCCCGCG ATCCTACCCC CTGAGAACAA CACGTGCCTG TGAGTGCGCC GGGGCCTCAG CAAGGGAAGC AGCAAGGAAG GGAGCACACA GTGGGGTTCA
Xenopus_laevis_X02995       ---------- ---------- ---------- ---------- ---------- ---------- ---------- ---------- ---------- ---------- ----------
Mus_musculus_NR_046233      CGCCCCCGTG GCGCCCGGGG GTGGGCGCGT CCGCATCTGC TCTGGTCGAG GTTGGCGGTT GAGGGTGTGC GTGCGCCGAG GTGGTGGTCG GTCCCCTGCG GCCGCGGGGT
Homo_sapiens_U13369         GGGGGCTCCC TCGCGCCGCG CGCGGCTCGG GGTTCGGGGT TCGTCGGCCC CGGCCGGGTG GAAGGTCCCG TGCCCGTCGT CGTCGTCGTC GCGCGTCGTC GGCGGTGGGG
Rattus_norvegicus_NR_046239 CTCCCCCGCC GTG------- --GGACGCGT CGGC------ -------GGG GC---CGGTC GGGTGC-TGG GCGCGGAGAG GGGGGGGAAA GTCGC----- --------GT
Gallus_gallus_KT445934.2    AGCGCGGCGG GGCCGGACGT TCGTTCGTTC GTTCGTCCGG CCCCCCGCCC CGGAGGAGCG CTCCTCGCCC TCCCCGGCCC CGCGCGCGGC TGCCTGCGGG TCGCGTTACC

                            9999999999 9999999999 9999999999 9999999999 9999999999 9999999999 9999999999 9999999999 9999999999 9999999999 9999999999
                            0000000000 0000000000 0000000000 0000000000 0000000000 0000000000 0000000000 0000000001 1111111111 1111111111 1111111111
                            2222222223 3333333334 4444444445 5555555556 6666666667 7777777778 8888888889 9999999990 0000000001 1111111112 2222222223
                            1234567890 1234567890 1234567890 1234567890 1234567890 1234567890 1234567890 1234567890 1234567890 1234567890 1234567890
Crocodylus_porosus_EU727191 GACACTCAGC TGGTGTGGGG GCGAGTTCAG CAGGCAGGCA GCAGGCAGGT GCGGGCAGCT GGGGCCTTTG CCTGTGCCTC TGCCTGCCCA GTGACCGGGA CTGTCCTCTC
Xenopus_laevis_X02995       ---------- ---------- ---------- ---------- ---------- ---------- ---------- ---------- ---------- ---------- ----------
Mus_musculus_NR_046233      TGTCGGGGTG GCGGTCGACG AGGGCCGGTC GGTCGCCTGC GGTGGTTGTC TGTGTGTGTT TGGGTCTTGC GCTGGGGGAG GCGGGGTCGA CCGCTCGCGG GGTTGGCGCG
Homo_sapiens_U13369         GCGTGTTGCG TGCGGTGTGG TGGTGGGGGA GGAGGAAGGC GGGTCCGGAA GGGGAAGGGT GCCGGCGGGG AGAGAGGGTC GGGGGAGCGC GTCCCGGTCG CCGCGGTTCC
Rattus_norvegicus_NR_046239 CGCCGGGGT- -CGTTCGCCG ---------- ---CGCCTCC GGTGG----- ---------- --GGT----- ----GGGGGG CCGGGACC-- CCGCCCGCGG GGCCGGCGCG
```

```
Gallus_gallus_KT445934.2       GGCGGCGGTA ACGCGCCGTG CTGCCGCGCG CGTGGCGGTC CGGGTCGGGG CGAGGCTGCC GGCCTCCGGT CGTCCGCCCG TCCGTCCGGC CGAGCCCGGC GCGCGTCCCC

                               9999999999 9999999999 9999999999 9999999999 9999999999 9999999999 9999999999 9999999999 9999999999 9999999999 9999999999
                               1111111111 1111111111 1111111111 1111111111 1111111111 1111111111 1111111112 2222222222 2222222222 2222222222 2222222222
                               3333333334 4444444445 5555555556 6666666667 7777777778 8888888889 9999999990 0000000001 1111111112 2222222223 3333333334
                               1234567890 1234567890 1234567890 1234567890 1234567890 1234567890 1234567890 1234567890 1234567890 1234567890 1234567890
Crocodylus_porosus_EU727191    CCCACTGTCT CTGCTGCCTT CCCTCTGCCG GGCCCCTCCT GCCCCACGCG TTCCCGGCCT TCACGCCAGG GTGTGCCGTA TGCGGCTGCT CCTCCCTCAG GGCTGTTCTC
Xenopus_laevis_X02995          ---------- ---------- ---------- ---------- ---------- ---------- ---------- ---------- ---------- ---------- ----------
Mus_musculus_NR_046233         GTCGCCCGGC GCCGCGCACC C-TCCGGCTT GTGTGGAGGG AGAGCGAGGG CGAGAACGGA GAGAGGTGGT ATCCCCGGTG GCGTTGCGAG GGAGGGTTTG GCGTCCCGCG
Homo_sapiens_U13369            GCCGCCCGCC CCCGGTGGCG GCCCGGCGTC CGGCCGACCG GCCGCTCCCC GCGCCCCTCC TCCTCCCCGC CGCCCCTCCT CCGAGGCCCC GCCCGTCCTC CTCGCCCTCC
Rattus_norvegicus_NR_046239    TCCGCC-GCC GCCGCGCGCC CGCCCGGTGC GTGCGGAGAG AGAGAGAGAG -------AGA GAGA------ ---CCCGCGA GGGTCGTTCG GGGGGGC--- -CGTACCGCG
Gallus_gallus_KT445934.2       GCGGGTCCGT CTCCGGCCAC CGTGCGCCGG CGGCGGCGGC GGCGGTGCGA ACCGCCGGCG GCGCGCCGGC TCCCCCGTCC GGGCGTTCCT CCCTCGGCAG CGCCGGGAGC

                               9999999999 9999999999 9999999999 9999999999 9999999999 9999999999 9999999999 9999999999 9999999999 9999999999 9999999999
                               2222222222 2222222222 2222222222 2222222222 2222222222 2222222223 3333333333 3333333333 3333333333 3333333333 3333333333
                               4444444445 5555555556 6666666667 7777777778 8888888889 9999999990 0000000001 1111111112 2222222223 3333333334 4444444445
                               1234567890 1234567890 1234567890 1234567890 1234567890 1234567890 1234567890 1234567890 1234567890 1234567890 1234567890
Crocodylus_porosus_EU727191    CCCAGGTTCC TTCCATCGGG CCTCCTTTGG AGACGGCGCA GTCTCCTGTG GCTGTGGTGG GTAGGACTGC ACATGTGCAC AGCATGCTGA GAGACCAGGC CGGCCCTGTC
Xenopus_laevis_X02995          ---------- ---------- ---------- ---------- ---------- ---------- ---------- ---------- ---------- ---------- ----------
Mus_musculus_NR_046233         TCCGTCCGTC CCTCCCTCCC TCGGTGGGCG CCTTCGCGCC GCACGCGGCC GCTAGGGGCG GTCGGGGCCC GTGGCCCCCG TGGCTCTTCT TCGTCTCCGC TTCTCCTTCA
Homo_sapiens_U13369            CCGCGCGTAC GCGCGCGCGC CCGCCCGCCC GGCTCGCCTC GCGGCGCGTC GGCCGGGGCC GGGAGCCCGC CCCGCCGCCC GCCCGTGGCC GCGGCGCCGG GGTTCGCGTG
Rattus_norvegicus_NR_046239    CCCG------ ---------- ---------- ---------- ---------- ---------- --CGGGGCCG GT--CTCGCG TCCCTCTCCT CC---CCCGC TCCCTCT--A
Gallus_gallus_KT445934.2       AGCCGCTTGG CGTCCGAAGG CGGGTGGCCG GGCGAGCGCG GGCTCGCCCG GGGCCCGGCG TTCGGGCCCC GTTTCCGAT- ---------- ---------- ----------

                               9999999999 9999999999 9999999999 9999999999 9999999999 9999999999 9999999999 9999999999 9999999999 9999999999 9999999999
                               3333333333 3333333333 3333333333 3333333333 3333333334 4444444444 4444444444 4444444444 4444444444 4444444444 4444444444
                               5555555556 6666666667 7777777778 8888888889 9999999990 0000000001 1111111112 2222222223 3333333334 4444444445 5555555556
                               1234567890 1234567890 1234567890 1234567890 1234567890 1234567890 1234567890 1234567890 1234567890 1234567890 1234567890
Crocodylus_porosus_EU727191    CGGTACCGGC TTTCTCGGCC ATCCCGCCTC TCAAACCTCT CCCACACCTG CACCCTCCCA TCCAGC---- ---------- ---------- ---------- ----------
Xenopus_laevis_X02995          ---------- ---------- ---------- ---------- ---------- ---------- ---------- ---------- ---------- ---------- ----------
Mus_musculus_NR_046233         CCCGGGCGGT ACCCGCTCCG GCGCCGGCCC GCGGGACGCC GCGGCGTCCG TGCGCCGATG CGAGTCACCC CCGGGTGTTG CGA--GTTCG GGGAGGGAGA GGGCCTCGCT
Homo_sapiens_U13369            TCCCCGGCGG CGACCCGCGG GACGCCGCGG TGTCGTCCGC CGTCGCGCGC CCGCCTCCGG CTCGCGGCCG CGCCGCGCCG CGCCGGGGCC CCGTCCCGAG CTTCCGCGTC
Rattus_norvegicus_NR_046239    CCGGGGCCGA --CCGCTCCG GCGCCGGCCC GCGG--CGCC GCGGCGTCCG TGCGCCGACG CGACTTGCCC CCGGGATGTG GGGCCGGCCG GCGAAGGAGA GG---TCGCG
Gallus_gallus_KT445934.2       ---------- ---------- ---------- ---------- ---------- ---------- ---------- ---------- ---------- ---------- ----------

                               9999999999 9999999999 9999999999 9999999999 9999999999 9999999999 9999999999 9999999999 9999999999 9999999999 9999999999
                               4444444444 4444444444 4444444444 4444444445 5555555555 5555555555 5555555555 5555555555 5555555555 5555555555 5555555555
                               6666666667 7777777778 8888888889 9999999990 0000000001 1111111112 2222222223 3333333334 4444444445 5555555556 6666666667
                               1234567890 1234567890 1234567890 1234567890 1234567890 1234567890 1234567890 1234567890 1234567890 1234567890 1234567890
Crocodylus_porosus_EU727191    ---------- ---------- ---------- ---------- ---------- ---------- ---------- ---------- ---------- ---------- ----------
Xenopus_laevis_X02995          ---------- ---------- ---------- ---------- ---------- ---------- ---------- ---------- ---------- ---------- ----------
Mus_musculus_NR_046233         GACCCGTTGC GTCCCGGCTT CCCTGGGGGG GACCCGGCGT CTGTGGGCTG TGCGTCCCGG GGGTTGCGTG TGAGTAAGAT CCTCCACCCC CGCCGCCCTC CCCTCCCGCC
Homo_sapiens_U13369            GGGGCGGCGC GGCTCCGCCG CCGCGTCCTC GGACCCGTCC CCCCGACCTC CGCGGGGGAG ACGCGCCGGG GCGTGCGGCG CCCGTCCCGC CCCCGGCCCG TGCCCCTCCC
Rattus_norvegicus_NR_046239    GGCTC----C GGCCTGGCTC CCC------- ----CGGCGC CCTCGCCCCG T-CGTCCCGT CGTTCTCG-- ---------- ------CTCT CGCTCTCCTC TCCTC-----
Gallus_gallus_KT445934.2       ---------- ---------- ---------- ---------- ---------- ---------- ---------- ---------- ---------- ---------- ----------

                               9999999999 9999999999 9999999999 9999999999 9999999999 9999999999 9999999999 9999999999 9999999999 9999999999 9999999999
                               5555555555 5555555555 5555555556 6666666666 6666666666 6666666666 6666666666 6666666666 6666666666 6666666666 6666666666
                               7777777778 8888888889 9999999990 0000000001 1111111112 2222222223 3333333334 4444444445 5555555556 6666666667 7777777778
                               1234567890 1234567890 1234567890 1234567890 1234567890 1234567890 1234567890 1234567890 1234567890 1234567890 1234567890
Crocodylus_porosus_EU727191    ---------- ---------- ---------- ---------- ---------- ---------- ---------- ---------- ---------- ---------- ----------
Xenopus_laevis_X02995          ---------- ---------- ---------- ---------- ---------- ---------- ---------- ---------- ---------- ---------- ----------
Mus_musculus_NR_046233         GGCCTCTCGG GGACCCCCTG AGACGGTTCG CCGGCTCGTC CTCCCGTGCC GCCGGGTGCC GTCTCTTTCC CGCCCGCCTC CTCGCTCTCT TCTTCCCGCG GCTGGGCGCG
Homo_sapiens_U13369            TCCGGTCGTC CCGCTCCGGC GGGGCGGCGC GGGGGCGCCG TCGGCCGCGC GCTCTCTCTC CCGTCGCCTC TCCCCCTCGC CGGGCCCGTC TCCCGACGGA GCGTCGGGCG
```

```
Rattus_norvegicus_NR_046239    ---------- ---------- ---------- --------TC CTCTCCTTCC GTCG------ --------CC CGCGCGCGCC CAC-CTCTCC TCCT------ ----------
Gallus_gallus_KT445934.2       ---------- ---------- ---------- ---------- ---------- ---------- ---------- ---------- ---------- ---------- ----------

                               9999999999 9999999999 9999999999 9999999999 9999999999 9999999999 9999999999 9999999999 9999999999 9999999999 9999999999
                               6666666666 6666666667 7777777777 7777777777 7777777777 7777777777 7777777777 7777777777 7777777777 7777777777 7777777777
                               8888888889 9999999990 0000000001 1111111112 2222222223 3333333334 4444444445 5555555556 6666666667 7777777778 8888888889
                               1234567890 1234567890 1234567890 1234567890 1234567890 1234567890 1234567890 1234567890 1234567890 1234567890 1234567890
Crocodylus_porosus_EU727191    ---------- ---------- ---------- ---------- ---------- ---------- ----TGTGAC CTCAGATTAG ACTTGGTGAC CTGCTGAATT TAAGCATATT
Xenopus_laevis_X02995          ---------- ---------- ---------- ---------- ---------- ---------- ----TCAGAC CTCAGATCAG ACGCGGCGAC CCGCTGAATT TAAGCATATT
Mus_musculus_NR_046233         TGTCCCCCCT TTCTGAC--- ---------- ---------- ---------- ---------- ----CGCGAC CTCAGATCAG ACGTGGCGAC CCGCTGAATT TAAGCATATT
Homo_sapiens_U13369            GGCGGTCGGG CCGGCGCGAT TCCGTCCGTC CGTCCGCCGA GCGGCCCGTC CCCCTCCGAG A---CGCGAC CTCAGATCAG ACGTGGCGAC CCGCTGAATT TAAGCATATT
Rattus_norvegicus_NR_046239    --TCTCCTCC TCTGGAC--- ---------- ---------- ---------- ---------- ----CGCGAC CTCAGATCAG ACGTGGCGAC CCGCTGAATT TAAGCATATT
Gallus_gallus_KT445934.2       ---------- ---------- ---------- ---------- ---------- ---------- ----CGCGAC CTCAGGTCAG ACGTGGCGAC CCGCTGAATT TAAGCATATT

                               9999999999 9999999999 9999999999 9999999999 9999999999 9999999999 9999999999 9999999999 9999999999 9999999999 9999999999
                               7777777778 8888888888 8888888888 8888888888 8888888888 8888888888 8888888888 8888888888 8888888888 8888888888 8888888889
                               9999999990 0000000001 1111111112 2222222223 3333333334 4444444445 5555555556 6666666667 7777777778 8888888889 9999999990
                               1234567890 1234567890 1234567890 1234567890 1234567890 1234567890 1234567890 1234567890 1234567890 1234567890 1234567890
Crocodylus_porosus_EU727191    AGTCAGTGGA GGAAAAGAAA CTAACCAGGA TTCCCTCAGT AACGGTGAGT GAACAGGGAA GAGCCCAGCG CCGAATCCCC ATCCTGCAGT AGTGTGTAGG AAATGTGGTG
Xenopus_laevis_X02995          ACTAAGCGGA GGAAAAGAAA CTAACCAGGA TTCCCCCAGT AACGGCGAGT GAAGAGGGAA GAGCCCAGCG CCGAATCCCG CGCCCGC--- CGGGCGCGGG ACGTGTGGCG
Mus_musculus_NR_046233         AGTCAGCGGA GGAAAAGAAA CTAACCAGGA TTCCCTCAGT AACGGCGAGT GAACAGGGAA GAGCCCAGCG CCGAATCCCC GCCGCGCGTC GCGGCGTGGG AAATGTGGCG
Homo_sapiens_U13369            AGTCAGCGGA GGAAAAGAAA CTAACCAGGA TTCCCTCAGT AACGGCGAGT GAACAGGGAA GAGCCCAGCG CCGAATCCCC GCCCCGC--- GGGGCGCGGG ACATGTGGCG
Rattus_norvegicus_NR_046239    AGTCAGCGGA GGAAAAGAAA CTAACCAGGA TTCCCTCAGT AACGGCGAGT GAACAGGGAA GAGCCCAGCG CCGAATCCCC GCCGCGCGCC GCGGCGCGGG AAATGTGGCG
Gallus_gallus_KT445934.2       AGTCAGCGGA GGAAAAGAAA CTAACGAGGA TTCCCTCAGT AACGGCGAGT GAAGAGGGAA GAGCCCAGCG CCGAATCCCC GCCCCGCGGT GGGGCGCGGG AGGTGTGGCG

                                                                                                                                             1 1111111111
                               9999999999 9999999999 9999999999 9999999999 9999999999 9999999999 9999999999 9999999999 9999999999 9999999990 0000000000
                               9999999999 9999999999 9999999999 9999999999 9999999999 9999999999 9999999999 9999999999 9999999999 9999999990 0000000000
                               0000000001 1111111112 2222222223 3333333334 4444444445 5555555556 6666666667 7777777778 8888888889 9999999990 0000000001
                               1234567890 1234567890 1234567890 1234567890 1234567890 1234567890 1234567890 1234567890 1234567890 1234567890 1234567890
Crocodylus_porosus_EU727191    GTGTACAGAA GACCCA--CT CCCCAGTGCC GCTCTCGGGG GCCCCAAGTC CTTCTGATCA AGGCACAGCC CGTGGATGGT GTGAGGGCAG TAG-TGGCCC CTGGTGCGCT
Xenopus_laevis_X02995          ---TACGGGA GACCGGACCC CCCCGGCGCG GCTCG---GG GGCCCAAGTC CTTCTGATCG AGGCCCAGCC CGCGGACGGT GTTAGGCCGG TGGGCGGCCC CCGGCGCGGC
Mus_musculus_NR_046233         ---TACGGAA GACCCA--CT CCCCGGCGCC GCTCGTGGGG GGCCCAAGTC CTTCTGATCG AGGCCCAGCC CGTGGACGGT GTGAGGCCGG TAG-CGGCCC -CGGCGCGCC
Homo_sapiens_U13369            ---TACGGAA GACCCG--CT CCCCGGCGCC GCTCGTGGGG GGCCCAAGTC CTTCTGATCG AGGCCCAGCC CGTGGACGGT GTGAGGCCGG TAG-CGGC-- -CGGCGCGC-
Rattus_norvegicus_NR_046239    ---TACGGAA GACCCA--CT CCCCGGCGCC GCTCGTGGGG GGCCCAAGTC CTTCTGATCG AGGCCCAGCC CGTGGACGGT GTGAGGCCGG TAG-CGGCCC -CGGCGCGCC
Gallus_gallus_KT445934.2       ---TACGGAA GCCCCC--AT CCCCGGCGCC GCTCTCGGGG GGCCCAAGTC CTTCTGATCG AGGCCCAGCC CGCGGACGGT GTGAGGCCGG TAGCGGCCCC CCGGCGCGCC

                               1111111111 1111111111 1111111111 1111111111 1111111111 1111111111 1111111111 1111111111 1111111111 1111111111 1111111111
                               0000000000 0000000000 0000000000 0000000000 0000000000 0000000000 0000000000 0000000000 0000000000 0000000000 0000000000
                               0000000000 0000000000 0000000000 0000000000 0000000000 0000000000 0000000000 0000000000 0000000001 1111111111 1111111111
                               1111111112 2222222223 3333333334 4444444445 5555555556 6666666667 7777777778 8888888889 9999999990 0000000001 1111111112
                               1234567890 1234567890 1234567890 1234567890 1234567890 1234567890 1234567890 1234567890 1234567890 1234567890 1234567890
Crocodylus_porosus_EU727191    GGGAGTGGGT CTTCTCAGAG TTGGGTTGCT TGGCAATGAA GCCCAAAGCA GGTGGTAAAC TC-ATCTAAG GCTAAATACT AGCACAAGGC CAATAGTCAG CAAGTACTGT
Xenopus_laevis_X02995          GGGACCCGGT CTCCTCGGAG TCGGGTTGTT TGGGAATGCA GCCCAAAGCG GGTGGTAAAC TCCATCTAAG GCTAAATACC GGCACGAGAC CGATAGCGGA CAAGTACCGT
Mus_musculus_NR_046233         GGGCTCGGGT CTTCCCGGAG TCGGGTTGCT TGGGAATGCA GCCCAAAGCG GGTGGTAAAC TCCATCTAAG GCTAAATACC GGCACGAGAC CGATAGTCAA CAAGTACCGT
Homo_sapiens_U13369            --GCCCGGGT CTTCCCGGAG TCGGGTTGCT TGGGAATGCA GCCCAAAGCG GGTGGTAAAC TCCATCTAAG GCTAAATACC GGCACGAGAC CGATAGTCAA CAAGTACCGT
Rattus_norvegicus_NR_046239    GGGCCCGGGT CTTCCCGGAG TCGGGTTGCT TGGGAATGCA GCCCAAAGCG GGTGGTAAAC TCCATCTAAG GCTAAATACC GGCACGAGAC CGATAGCCAA CAAGTACCGT
Gallus_gallus_KT445934.2       GGGCCCGGGG CTTCTCGGAG TCGGGTTGCT TGGGAATGCA GCCCAAAGCG GGTGGTAAAC TCCATCTAAG GCTAAATACC GGCACGAGAC CGATAGCCAA CAAGTACCGT
```

```
                            1111111111 1111111111 1111111111 1111111111 1111111111 1111111111 1111111111 1111111111 1111111111 1111111111 1111111111
                            0000000000 0000000000 0000000000 0000000000 0000000000 0000000000 0000000000 0000000000 0000000000 0000000000 0000000000
                            1111111111 1111111111 1111111111 1111111111 1111111111 1111111111 1111111111 1111111112 2222222222 2222222222 2222222222
                            2222222223 3333333334 4444444445 5555555556 6666666667 7777777778 8888888889 9999999990 0000000001 1111111112 2222222223
                            1234567890 1234567890 1234567890 1234567890 1234567890 1234567890 1234567890 1234567890 1234567890 1234567890 1234567890
Crocodylus_porosus_EU727191 AAGAGAAAGT TTAAAAGAAC TTTGAGGATA GAGTTCAAGA GGGTGTGAAA CCGTTAAGAG TTAAACGGGT GGGGTCTGTG CAGTCTGCCC GAAGGATTCA ACCCAGTGG-
Xenopus_laevis_X02995       AAGG-AAAGT TGAAAAGAAC TTTGAAGAGA GAGTTCAAGA GGGCGTGAAA CCGTTAAGAG GTAAACGGGT GGGG-CCGTG CGGTCCGCCC GGAGGATTCA ACCCGGCGGG
Mus_musculus_NR_046233      AAGGGAAAGT TGAAAAGAAC TTTGAAGAGA GAGTTCAAGA GGGCGTGAAA CCGTTAAGAG GTAAACGGGT GGGGTCCGCG CAGTCCGCCC GGAGGATTCA ACCCGGCGGC
Homo_sapiens_U13369         AAGGGAAAGT TGAAAAGAAC TTTGAAGAGA GAGTTCAAGA GGGCGTGAAA CCGTTAAGAG GTAAACGGGT GGGGTCCGCG CAGTCCGCCC GGAGGATTCA ACCCGGCGGC
Rattus_norvegicus_NR_046239 AAGGGAAAGT TGAAAAGAAC TTTGAAGAGA GAGTTCAAGA GGGCGTGAAA CCGTTAAGAG GTAAACGGGT GGGGTCCGCG CAGTCCGCCC GGAGGATTCA ACCCGGCGGC
Gallus_gallus_KT445934.2    AAGGGAAAGT TGAAAAGAAC TTTGAAGAGA GAGTTCAAGA GGGCGTGAAA CCGTTAAGAG GTAAACGGGT GGGGTCCGCG CAGTCGGCCC GGAGGATTCA ACCCGGCGGG

                            1111111111 1111111111 1111111111 1111111111 1111111111 1111111111 1111111111 1111111111 1111111111 1111111111 1111111111
                            0000000000 0000000000 0000000000 0000000000 0000000000 0000000000 0000000000 0000000000 0000000000 0000000000 0000000000
                            2222222222 2222222222 2222222222 2222222222 2222222222 2222222222 2222222223 3333333333 3333333333 3333333333 3333333333
                            3333333334 4444444445 5555555556 6666666667 7777777778 8888888889 9999999990 0000000001 1111111112 2222222223 3333333334
                            1234567890 1234567890 1234567890 1234567890 1234567890 1234567890 1234567890 1234567890 1234567890 1234567890 1234567890
Crocodylus_porosus_EU727191 GTTTGGTCAG CTGGCCTGGG A-----TGAC AGATC----- ---------- ---------- ---------- ---------- --CCGCTC-- ---------- --------AG
Xenopus_laevis_X02995       TCAGCGCCGC CGGGACCGGG CCA-CTCGGC GGACC----- ---------- ---------- ---------- ---------- CCCCCGCC-- ---------- --------GG
Mus_musculus_NR_046233      GCGCGTCCGG CCGTGCCCGG TGGTCCCGGC GGATCTTTCC CGCTCCCCGT TCCTCCCGAC CCCTCCACCC GCGCGTCGTT CCCCTCTT-- ---------- -------CCT
Homo_sapiens_U13369         GGGT--CCGG CCGTGTC-GG CGG-CCCGGC GGATCTTTCC CGCCCCCCGT TCCTCCCGAC CCCTCCACCC GC---CCTCC CTTCCCCC-- ---------- ------GCCG
Rattus_norvegicus_NR_046239 GCGCG-CCGG CCG-GCC-GG TGGTCCCGGC GGATCTTTCC CGCTCCCCGT TCCTCCCGAC CCCTCCACCC GCGCGTCTCT CTCCCCCCTC CCCGCGTCCC GCCGTCGCCG
Gallus_gallus_KT445934.2    CCAAGGTCGG CCGGCGCGGG CG---CCGTC GGATC---CC CGCCTCCG-- ---------- ---------- ---------C CTCCCCTC-- ---------- --------CG

                            1111111111 1111111111 1111111111 1111111111 1111111111 1111111111 1111111111 1111111111 1111111111 1111111111 1111111111
                            0000000000 0000000000 0000000000 0000000000 0000000000 0000000000 0000000000 0000000000 0000000000 0000000000 0000000000
                            3333333333 3333333333 3333333333 3333333333 3333333333 3333333334 4444444444 4444444444 4444444444 4444444444 4444444444
                            4444444445 5555555556 6666666667 7777777778 8888888889 9999999990 0000000001 1111111112 2222222223 3333333334 4444444445
                            1234567890 1234567890 1234567890 1234567890 1234567890 1234567890 1234567890 1234567890 1234567890 1234567890 1234567890
Crocodylus_porosus_EU727191 CCCCTCCCCC ---------- ---------- ---------- ---------- ---------- ---------- ---------- --------AG GGCAGGT-GA GGACCGCCGC
Xenopus_laevis_X02995       CCCCCTCCCC TCG--CCGGG AGGG------ ---------- ---------- ---------- ---------- ---------- ----CGGTCG CGCGGGGGGG ACGCGG---C
Mus_musculus_NR_046233      CCCCGCGTCC GGCGCCTCCG GCGG------ CGGGCGC--- GGGGGGTGGT GTGGTG---- ---GTGGCGC GCGGGCGGGG CCGGGGGTGG GGTCGGCGGG GGACCG-CCC
Homo_sapiens_U13369         CCCCTCCTCC TCCTCCCCGG AGGG----GG CGGGCTC--- --CGGCGGGT GCGGGG---- ------GTGG GCGGGCGGGG CCGGGGGTGG GGTCGGCGGG GGACCGTCCC
Rattus_norvegicus_NR_046239 TCCCCGCTCC TCCCTCCGGG GGGGTGTCGG CGGGCGCTCC GGCGGCGGGC GCGGGGTGTG GTGGGGGCGC GCGGGCGGGG CCGGGGGTGG GGTCGGCGGG GGACCG-CCC
Gallus_gallus_KT445934.2    TCCCTCCCCT TCG--CCGGG GCGG------ ---------- ---------- -----G---- ---------- GCGGGCCCAG --GGGGGGCG GGCGGGCCGG GGACCGCCGC

                            1111111111 1111111111 1111111111 1111111111 1111111111 1111111111 1111111111 1111111111 1111111111 1111111111 1111111111
                            0000000000 0000000000 0000000000 0000000000 0000000000 0000000000 0000000000 0000000000 0000000000 0000000000 0000000000
                            4444444444 4444444444 4444444444 4444444444 4444444445 5555555555 5555555555 5555555555 5555555555 5555555555 5555555555
                            5555555556 6666666667 7777777778 8888888889 9999999990 0000000001 1111111112 2222222223 3333333334 4444444445 5555555556
                            1234567890 1234567890 1234567890 1234567890 1234567890 1234567890 1234567890 1234567890 1234567890 1234567890 1234567890
Crocodylus_porosus_EU727191 CTGGATGGC- CTCGGTCCTT GTTGGGCGCA TCTCCACCGT GGCAGTGCAC CATGACTGGC TCTGGGTTGA CTGGGAAGGC CTGGCAG--- GCAGGTGGCT CACCGCT---
Xenopus_laevis_X02995       CCG---GGCG CCCGGC---- ----GCCGCG TTTCCTCCGC GGCGGTGCGC -GCG-CCGGC TCCGGGCCGC GTGGGAAGGC CCAGGGG--- --------TT CAGGGGA---
Mus_musculus_NR_046233      CCGGCCGGCG ACCGGCCGCC GCCGGGCGCA CTTCCACCGT GGCGGTGCGC CGCGACCGGC TCCGGGACGG CCGGGAAGGC CCGGTGG--G GAAGGTGGCT CGGGGGG---
Homo_sapiens_U13369         CCGACCGGCG ACCGGCCGCC GCCGGGCGCA TTTCCACCGC GGCGGTGCGC CGCGACCGGC TCCGGGACGG CTGGGAAGGC CCGGCGG--G GAAGGTGGCT CGGGGGGCCC
Rattus_norvegicus_NR_046239 CCGGTCGGCG ACCGGCCGCC GCCGGGCGCA CTTCCACCGT GGCGGTGCGC CGCGACCGGC TCCGGGACGG CTGGGAAGGC CCGGCGG--G GAAGGTGGCT CGGGGGG---
Gallus_gallus_KT445934.2    CCGGCCGGCG TCCGGCCCCC GTCGGGCGCA TTTCCTCCGC GGCGGTGCGC CGCGACCGGC TCCGGGACGG CTGGGAAGGG CTGCCGGCGG GCAGGTGGCC C---------
```

```
                               1111111111 1111111111 1111111111 1111111111 1111111111 1111111111 1111111111 1111111111 1111111111 1111111111 1111111111
                               0000000000 0000000000 0000000000 0000000000 0000000000 0000000000 0000000000 0000000000 0000000000 0000000000 0000000000
                               5555555555 5555555555 5555555555 5555555556 6666666666 6666666666 6666666666 6666666666 6666666666 6666666666 6666666666
                               6666666667 7777777778 8888888889 9999999990 0000000001 1111111112 2222222223 3333333334 4444444445 5555555556 6666666667
                               1234567890 1234567890 1234567890 1234567890 1234567890 1234567890 1234567890 1234567890 1234567890 1234567890 1234567890
Crocodylus_porosus_EU727191    ---------- ---------- ---------- ---------- ---------- ---------- ---------- ---------- ---------- ---------- ----------
Xenopus_laevis_X02995          ---------- ---------- ---------- ---------- ---------- ---------- ---------- ---------- ---------- ---------- ----------
Mus_musculus_NR_046233         ---------- ---------- ---------- ---------- ---------- ---------- ---------- ---------- ---------- ---------- GGCGGCGCGT
Homo_sapiens_U13369            CGTCCGTCCG TCCGTCCTCC TCCTCCCCCG TCTCCGCCCC CCGGCCCCGC GTCCTCCCTC GGGAGGGCGC GCGGGTCGGG GCGGCGGCGG CGGCGGCGGT GGCGGCG-GC
Rattus_norvegicus_NR_046239    ---------- ---------- ---------- ---------- ---------- ---------- ---------- ---------- ---------- ---------- GGCGGCG-TC
Gallus_gallus_KT445934.2       ---------- ---------- ---------- ---------- ---------- ---------- ---------- ---------- ---------- ---------- ----------

                               1111111111 1111111111 1111111111 1111111111 1111111111 1111111111 1111111111 1111111111 1111111111 1111111111 1111111111
                               0000000000 0000000000 0000000000 0000000000 0000000000 0000000000 0000000000 0000000000 0000000000 0000000000 0000000000
                               6666666666 6666666666 6666666667 7777777777 7777777777 7777777777 7777777777 7777777777 7777777777 7777777777 7777777777
                               7777777778 8888888889 9999999990 0000000001 1111111112 2222222223 3333333334 4444444445 5555555556 6666666667 7777777778
                               1234567890 1234567890 1234567890 1234567890 1234567890 1234567890 1234567890 1234567890 1234567890 1234567890 1234567890
Crocodylus_porosus_EU727191    ---------- ---------- TTGTGGCGGC AAGTGTTACA GTCCCCA--- GGCAGCAGCT CTTGCTGCAT CCCAGGGTCG AGGGA----G ATGACCGTCG CCACGCCTTC
Xenopus_laevis_X02995          -----AGGTG GCCGGCC--- GCCCCCGCGC GCG-GCTACA GCCCCCCCCC AGCAGCAGCA CTCGCCGTCG CCCGGGGCCG AGGGA----G ACGCCGGCCT CCGCG--GTC
Mus_musculus_NR_046233         CTCAGGGCGC GCCGAACC-- ACCTCACCCC GAGTGTTACA GCCCTC--C- GGCCGC-GCT TTCGCCGAAT CCCGGGGCCG AGGAAGCCAG ATACCCGTCG CCGCGCTCTC
Homo_sapiens_U13369            GGCGGGGGCG GCGGGACCGA AACCCCCCCC GAGTGTTACA GCCCCC--CC GGCAGCAGCA CTCGCCGAAT CCCGGGGCCG AGGGAGC--G AGACCCGTCG CCGCGCTCTC
Rattus_norvegicus_NR_046239    ACCCGTGGGC GCCGGACC-- ACCCCGCCCC GAGTGTTACA GCCCCC--C- GGCAGCAGCG CTCGCCGAAT CCCGGGGCCG AGGGAGCCGG ATACCCGTCG CCGCGCTCTC
Gallus_gallus_KT445934.2       ---------G GCGCCGCGCG AGCGGCCGCC GGGTGTTATA GCC---GCCG GGCCCGGATC GTCGCCGAAT CCCGGGGCCG AGGGAG---- AGGACCGCCG CCGCGCCCTC

                               1111111111 1111111111 1111111111 1111111111 1111111111 1111111111 1111111111 1111111111 1111111111 1111111111 1111111111
                               0000000000 0000000000 0000000000 0000000000 0000000000 0000000000 0000000000 0000000000 0000000000 0000000000 0000000000
                               7777777777 7777777778 8888888888 8888888888 8888888888 8888888888 8888888888 8888888888 8888888888 8888888888 8888888888
                               8888888889 9999999990 0000000001 1111111112 2222222223 3333333334 4444444445 5555555556 6666666667 7777777778 8888888889
                               1234567890 1234567890 1234567890 1234567890 1234567890 1234567890 1234567890 1234567890 1234567890 1234567890 1234567890
Crocodylus_porosus_EU727191    CCCCATGGCT C--CCCGTCC CCTC------ CTCCTCATGG TGG------- ---------- ---------- ---------- ---------G GGCCCCCTCG CCCCTGGTGC
Xenopus_laevis_X02995          CTCCTCCCCG GAGCGCGTCC CGCCGCTC-- CCCCCCGGGG GGGCG----- ------GCGC GCGGGGCGGG GAA------- --GGGGGAAG GGGCCCCCCG CTCCCGGCGC
Mus_musculus_NR_046233         CCTCTCCCC- ----CCGTCC GCCT------ ---CCCGGGC GGG------- ---------- ---CGTGGGG GT-------- --GGGGGCCG GGCCGCCCCT CCCACGGCGC
Homo_sapiens_U13369            CCCCCTCCCG GCGCCCACCC CCGCGGGGAA TCCCCCGCGA GGGGGGTCTC CCCCGCGGGG GCGCGCCGGC GTCTCCTCGT GGGGGGGCCG GGCCACCCCT CCCACGGCGC
Rattus_norvegicus_NR_046239    CCCCCGGCCT CTCCCCTCCC GCCC------ CTCCCCGTGG GGTGG----- ---CGGAAAG GGGGGCGGTC GC-------- --GGGGGCCG GGCCGCCCCT CCCACGGCGC
Gallus_gallus_KT445934.2       CCCCGGAGGG GGCGGCCCCC CGGAGGGCCC CCCGCGGCCG GACCGGCGTC GGGCCGGCCG CGCCGCGCGC GCGTCCGCGC CGCCGCCGTA CGCCGCCGCT CGCTCTCTCT

                               1111111111 1111111111 1111111111 1111111111 1111111111 1111111111 1111111111 1111111111 1111111111 1111111111 1111111111
                               0000000000 0000000000 0000000000 0000000000 0000000000 0000000000 0000000000 0000000000 0000000000 0000000000 0000000001
                               8888888889 9999999999 9999999999 9999999999 9999999999 9999999999 9999999999 9999999999 9999999999 9999999999 9999999990
                               9999999990 0000000001 1111111112 2222222223 3333333334 4444444445 5555555556 6666666667 7777777778 8888888889 9999999990
                               1234567890 1234567890 1234567890 1234567890 1234567890 1234567890 1234567890 1234567890 1234567890 1234567890 1234567890
Crocodylus_porosus_EU727191    AACTGTCAAT ---------- ---------- ---------- ---------- ---------- ---------- ---------- ---------- ---------- ----------
Xenopus_laevis_X02995          GGCTGTCAAC ---------- ---------- ---------- ---------- ---------- ---------- ---------- ---------- ---------- ----------
Mus_musculus_NR_046233         GACCGCTCTC CCACCCC--- --------CC TCCGTCGCCT CTCTCGGGGC CCGG------ ------T--- ---------- ---------- ---------- ----------
Homo_sapiens_U13369            GACCGCTCTC CCACCCCTCC TCCCCGCGCC CCCGCCCCGG CGACGGGGGG GGTGCCGCGC GCGGGTC--- ---------- ---------- ---------- ----------
Rattus_norvegicus_NR_046239    GACCGCTCTC CCACCCCCCG TC-----GCC TCCGTCGTCC CTCTCGGGGG TCCG------ GGGGCCC--- ---------- ---------- ---------- ----------
Gallus_gallus_KT445934.2       CCGTTCCCCG CCCCGG-GTC CGTCCCGGGG CGCGGGGGCG GGGGGGGTC- --GGGTGTCC GGCGCGCGGC TCGGCGCGGC GCCGCGCGTG TGGCGCGCGC CTCCAGCCCG
```

```
                             1111111111 1111111111 1111111111 1111111111 1111111111 1111111111 1111111111 1111111111 1111111111 1111111111 1111111111
                             1111111111 1111111111 1111111111 1111111111 1111111111 1111111111 1111111111 1111111111 1111111111 1111111111 1111111111
                             0000000000 0000000000 0000000000 0000000000 0000000000 0000000000 0000000000 0000000000 0000000000 0000000001 1111111111
                             0000000001 1111111112 2222222223 3333333334 4444444445 5555555556 6666666667 7777777778 8888888889 9999999990 0000000001
                             1234567890 1234567890 1234567890 1234567890 1234567890 1234567890 1234567890 1234567890 1234567890 1234567890 1234567890
Crocodylus_porosus_EU727191  ---------- ---------- ---------- ---------- ---------- ---------- ---------- ---------- ---------- ---------- ----------
Xenopus_laevis_X02995        ---------- ---------- ---------- ---------- ---------- ---------- ---------- ---------- ---------- ---------- ----------
Mus_musculus_NR_046233       ---------- ---------- ---------- ---------- ---------- ---------- ---------- ---------- ---------- ---------- ----------
Homo_sapiens_U13369          ---------- ---------- ---------- ---------- ---------- ---------- ---------- ---------- ---------- ---------- ----------
Rattus_norvegicus_NR_046239  ---------- ---------- ---------- ---------- ---------- ---------- ---------- ---------- ---------- ---------- ----------
Gallus_gallus_KT445934.2     GCGCGGGCGA GGCCGCGGGG GGCGCCGGGG GGGAACCTTC CCCCTTCTGT TCGGGCCGCC TCCGTTCCCG CGGGGGCGGC CCGTTCGGGG GACGGGCCCG CCGGCCCCCG

                             1111111111 1111111111 1111111111 1111111111 1111111111 1111111111 1111111111 1111111111 1111111111 1111111111 1111111111
                             1111111111 1111111111 1111111111 1111111111 1111111111 1111111111 1111111111 1111111111 1111111111 1111111111 1111111111
                             1111111111 1111111111 1111111111 1111111111 1111111111 1111111111 1111111111 1111111111 1111111112 2222222222 2222222222
                             1111111112 2222222223 3333333334 4444444445 5555555556 6666666667 7777777778 8888888889 9999999990 0000000001 1111111112
                             1234567890 1234567890 1234567890 1234567890 1234567890 1234567890 1234567890 1234567890 1234567890 1234567890 1234567890
Crocodylus_porosus_EU727191  ---------- -----CAGGG CGGACTGTTC TCAGTATGCT CCGACCGT-G TCGTGCTGCT GGGCAGGGAG GG-------- ---------- ---------- --CC------
Xenopus_laevis_X02995        ---------- -----CGGGG CGGACTGCCC CCAGTGCGCC CCGTCCGC-G CCGCGCCGCC GAGGCGGGAG GG-------- ---------- ---------- CCGCCGG---
Mus_musculus_NR_046233       ---------G GGGGGCGGGG CGGACTGTCC CCAGTGCGCC CCGGGCGTCG TCGCGCCGTC GGGTCCCGGG GGG------- ----ACCGTC GGTCACGCGT CTCC------
Homo_sapiens_U13369          ---------G GGGGGCGGGG CGGACTGTCC CCAGTGCGCC CCGGGCGG-G TCGCGCCGTC GGGCCCGGGG GAG--GTTCT CT-CGGGGCC ACGCGCGCGT CCCCCGAAGA
Rattus_norvegicus_NR_046239  ---------G GGGGGCGGGG CGGACTGTCC CCAGTGCGCC CCGGGCGTCG TCGCGCCGTC GGGCCCGGGG GGGCCGTCGT CA-CGCGCTC TCCCTCCCCT TCTC-GGGGT
Gallus_gallus_KT445934.2     GCGCCGCTGT CCGACCGGGG CGGACTGCGC TCAGTGCGCC CCGACCGC-G CGGCGCCGCC GGGCCGGG-- ---------- ---------- ---------- ----------

                             1111111111 1111111111 1111111111 1111111111 1111111111 1111111111 1111111111 1111111111 1111111111 1111111111 1111111111
                             1111111111 1111111111 1111111111 1111111111 1111111111 1111111111 1111111111 1111111111 1111111111 1111111111 1111111111
                             2222222222 2222222222 2222222222 2222222222 2222222222 2222222222 2222222222 2222222223 3333333333 3333333333 3333333333
                             2222222223 3333333334 4444444445 5555555556 6666666667 7777777778 8888888889 9999999990 0000000001 1111111112 2222222223
                             1234567890 1234567890 1234567890 1234567890 1234567890 1234567890 1234567890 1234567890 1234567890 1234567890 1234567890
Crocodylus_porosus_EU727191  ---------- -ATGCTGGGC ACCTGGGGTC TGCAGCGATG TTGGCTGCCC ACCCAACCCG TCTTGAAACA CGGACCAAGG AGTCTAACAT GTGTGCAAGT CAG-AGGCTC
Xenopus_laevis_X02995        ---------- ------GAGC CGCCAGGGTC CGCGGCGATG TCGGTGTCCC ACCCGACCCG TCTTGAAACA CGGACCAAGG AGTCTAACGC GCGCGCGAGT CGGAGGGACT
Mus_musculus_NR_046233       ------CGAC GAAGCCGAGC GCACGGGGTC GGCGGCGATG TCGGCTACCC ACCCGACCCG TCTTGAAACA CGGACCAAGG AGTCTAACGC GTGCGCGAGT CAG-GGGCTC
Homo_sapiens_U13369          GGGGGACGGC GGAGC-GAGC GCACGGGGTC GGCGGCGACG TCGGCTACCC ACCCGACCCG TCTTGAAACA CGGACCAAGG AGTCTAACAC GTGCGCGAGT CGG-GGGCTC
Rattus_norvegicus_NR_046239  GGGGGGGAGC GAAGCCGAGC GCACGGGGTC GGCGGCGATG TCGGCTACCC ACCCGACCCG TCTTGAAACA CGGACCAAGG AGTCTAACGC GTGCGCGAGT CAG-GGGCTC
Gallus_gallus_KT445934.2     --CTCGGGCC ACGCCAGGGC GCCCGGGGTC CGCGGCGACG TCGGCTACCC ACCCGACCCG TCTTGAAACA CGGACCAAGG AGTCTAGCAC GCGCGCGAGT CGG-CGGCTC

                             1111111111 1111111111 1111111111 1111111111 1111111111 1111111111 1111111111 1111111111 1111111111 1111111111 1111111111
                             1111111111 1111111111 1111111111 1111111111 1111111111 1111111111 1111111111 1111111111 1111111111 1111111111 1111111111
                             3333333333 3333333333 3333333333 3333333333 3333333333 3333333333 3333333334 4444444444 4444444444 4444444444 4444444444
                             3333333334 4444444445 5555555556 6666666667 7777777778 8888888889 9999999990 0000000001 1111111112 2222222223 3333333334
                             1234567890 1234567890 1234567890 1234567890 1234567890 1234567890 1234567890 1234567890 1234567890 1234567890 1234567890
Crocodylus_porosus_EU727191  GAACGAAAGC C--CATGACG CAATGAAGGT GAGGGTCGGT GAGCAC--CA GCT--GAGGT GGGATCCCGA GGCCACTGT- ------TTGC AGAGGGCG-- ----------
Xenopus_laevis_X02995        CTGCGCGAAA CCCTGTGGCG CAATGAAGGT GAGGGCCGGG GCGCCC--CG GCT--GAGGT GGGATCCCGC CGCCCCTCCC TCCGCCCCCC CGGGGGCGGG GGGGGGGCGC
Mus_musculus_NR_046233       GTCCGAAAGC CGCCGTGGCG CAATGAAGGT GAAGGGCCCC G--CCCGGGG GCCC-GAGGT GGGATCCCGA GGCCTCTCC- ---AGTCCGC CGAGGGCG-- ----------
Homo_sapiens_U13369          GCACGAAAGC CGCCGTGGCG CAATGAAGGT GAAGGCCGGC GCGCTCGCCG GCC--GAGGT GGGATCCCGA GGCCTCTCC- ---AGTCCGC CGAGGGCG-- ----------
Rattus_norvegicus_NR_046239  GTCCGAAAGC CGCCGTGGCG CAATGAAGGT GAAGGGCCCC GTTCCCGGGG GCCCCGAGGT GGGATCCCGA GGCCTCTCC- ---AGTCCGC CGAGGGCG-- ----------
Gallus_gallus_KT445934.2     GCGCGAAAGC C--CGCGGCG CAATGAAGGT GAGGGCCGGC GCGCGC--CG GCT--GAGGT GGGATCCCGG GGCGGCAGGC --CGGAAGGC CCCGGGCG-- ----------
```

```
                              1111111111 1111111111 1111111111 1111111111 1111111111 1111111111 1111111111 1111111111 1111111111 1111111111 1111111111
                              1111111111 1111111111 1111111111 1111111111 1111111111 1111111111 1111111111 1111111111 1111111111 1111111111 1111111111
                              4444444444 4444444444 4444444444 4444444444 4444444444 4444444445 5555555555 5555555555 5555555555 5555555555 5555555555
                              4444444445 5555555556 6666666667 7777777778 8888888889 9999999990 0000000001 1111111112 2222222223 3333333334 4444444445
                              1234567890 1234567890 1234567890 1234567890 1234567890 1234567890 1234567890 1234567890 1234567890 1234567890 1234567890
Crocodylus_porosus_EU727191   --------CA CCACTGACCT GTCTCGCCCA CCCCGTCAGG GAGGCGGAGC GTGAGCATAC GTG-CTAGGA CCTGAAAGAT GGTGAACTAT GCCTAGGAAG GGCAAAGCCA
Xenopus_laevis_X02995         CGGCGGGCCA CCACCGGCCC GTCTCGCCCG CCCCGTCGGG G-GGTGG-GC GTGAGCGCGC GCGATTAGGA CCCGAAAGAT GGTGAACTAT GCCTGGGCAG G-CGAAGCCA
Mus_musculus_NR_046233        --------CA CCACCGGCCC GTCTCGCCCG CCGCGCCGGG GAGGTGGAGC ACGAGCGTAC GCG-TTAGGA CCCGAAAGAT GGTGAACTAT GCTTGGGCAG GGCGAAGCCA
Homo_sapiens_U13369           --------CA CCACCGGCCC GTCTCGCCCG CCGCGCCGGG GAGGTGGAGC ACGAGCGCAC GTG-TTAGGA CCCGAAAGAT GGTGAACTAT GCCTGGGCAG GGCGAAGCCA
Rattus_norvegicus_NR_046239   --------CA CCACCGGCCC GTCTCGCCCG CCGCGCCGGG GAGGTGGAGC ACGAGCGTAC GCG-TTAGGA CCCGAAAGAT GGTGAACTAT GCTTGGGCAG GGCGAAGC-A
Gallus_gallus_KT445934.2      --------CA CCACCGGCCC GTCTCGCCCG CCTCGCCGGG GAGGTGGAGC ATGAGCGCGC GTG-CTAGGA CCCGAAAGAT GGTGAACTAT GCCTGGGCAG GGCGAAGCCA

                              1111111111 1111111111 1111111111 1111111111 1111111111 1111111111 1111111111 1111111111 1111111111 1111111111 1111111111
                              1111111111 1111111111 1111111111 1111111111 1111111111 1111111111 1111111111 1111111111 1111111111 1111111111 1111111111
                              5555555555 5555555555 5555555555 5555555555 5555555556 6666666666 6666666666 6666666666 6666666666 6666666666 6666666666
                              5555555556 6666666667 7777777778 8888888889 9999999990 0000000001 1111111112 2222222223 3333333334 4444444445 5555555556
                              1234567890 1234567890 1234567890 1234567890 1234567890 1234567890 1234567890 1234567890 1234567890 1234567890 1234567890
Crocodylus_porosus_EU727191   AAGGAAACTC TGGTGGAGGT CCATAGCAGT ACTGACATGC AAATCGGTCA TCTGACCTGG GTATAGGGGC GAAAGACTAA TTGAATCATC TAGTAGCTGG TTCCCTC---
Xenopus_laevis_X02995         GAGGAAACTC TGGTGGAGGT CCGTAGCGGT CCTGACGTGC AAATCGGTCG TCCGACCTGG -TATAGGGGC GAAAGACGAA TCGAACCATC TAGTAGCTGG TTCCCTCCGA
Mus_musculus_NR_046233        GAGGAAACTC TGGTGGAGGT CCGTAGCGGT CCTGACGTGC AAATCGGTCG TCCGACCTGG GTATAGGGGC GAAAGACTAA TCGAACCATC TAGTAGCTGG TTCCCTCCGA
Homo_sapiens_U13369           GAGGAAACTC TGGTGGAGGT CCGTAGCGGT CCTGACGTGC AAATCGGTCG TCCGACCTGG GTATAGGGGC GAAAGACTAA TCGAACCATC TAGTAGCTGG TTCCCTCCGA
Rattus_norvegicus_NR_046239   GAGGAAACTC TGGTGGAGGT CCGTAGCGGT CCTGACGTGC AAATCGGTCG TCCGACTTGG GTATAGGGGC GAAAGACTAA TCGAACCATC TAGTAGCTGG TTCCCTCCGA
Gallus_gallus_KT445934.2      GAGGAAACTC TGGTGGAGGT CCGTAGCGGT CCTGACGTGC AAATCGGTCG TCCGACCCGG GTATAGGGGC GAAAGACTAA TCGAACCATC TAGTAGCTGG TTCCCTCCGA

                              1111111111 1111111111 1111111111 1111111111 1111111111 1111111111 1111111111 1111111111 1111111111 1111111111 1111111111
                              1111111111 1111111111 1111111111 1111111111 1111111111 1111111111 1111111111 1111111111 1111111111 1111111111 1111111111
                              6666666666 6666666666 6666666666 6666666667 7777777777 7777777777 7777777777 7777777777 7777777777 7777777777 7777777777
                              6666666667 7777777778 8888888889 9999999990 0000000001 1111111112 2222222223 3333333334 4444444445 5555555556 6666666667
                              1234567890 1234567890 1234567890 1234567890 1234567890 1234567890 1234567890 1234567890 1234567890 1234567890 1234567890
Crocodylus_porosus_EU727191   ---------- AGGATAGCTG GCACTCACC- --------CA T-------CT CCGCAGTTTT ATTTGTTAAA GTGAATGATT AGAGGTCTTG GGGCCAAAA- CAATCTCAAC
Xenopus_laevis_X02995         AGTTTCCCTC AGGATAGCTG GCGCTGTC-- --------CG T--------- -CGCAGTTTT ATCCGGTAAA GCGAATGATT AGAGGTCTTG GGGCCGAAAT CGATCTCAAC
Mus_musculus_NR_046233        AGTTTCCCTC AGGATAGCTG GCGCTCTCG- --------CT C-CCGACG-T ACGCAGTTTT ATCCGGTAAA GCGAATGATT AGAGGTCTTG GGGCCGAAA- CGATCTCAAC
Homo_sapiens_U13369           AGTTTCCCTC AGGATAGCTG GCGCTCTCGC AGACCCGACG CACCCCCGCC ACGCAGTTTT ATCCGGTAAA GCGAATGATT AGAGGTCTTG GGGCCGAAA- CGATCTCAAC
Rattus_norvegicus_NR_046239   AGTTTCCCTC AGGATAGCTG GCGCTCTCGC AACGCGTTCG C-TCGACAAC CCGCAGTTTT ATCCGGTAAA GCGAATGATT AGAGGTCTTG GGGCCGAAA- CGATCTCAAC
Gallus_gallus_KT445934.2      AGTTTCCCTC AGGATAGCTG GCGCTCGGG- ---------G C-----GGCG GTGCAGTTTT ACCCGGTAAA GCGAATGATT AGAGGTCTTG GGGCCGAAA- CGATCTCAAC

                              1111111111 1111111111 1111111111 1111111111 1111111111 1111111111 1111111111 1111111111 1111111111 1111111111 1111111111
                              1111111111 1111111111 1111111111 1111111111 1111111111 1111111111 1111111111 1111111111 1111111111 1111111111 1111111111
                              7777777777 7777777777 7777777778 8888888888 8888888888 8888888888 8888888888 8888888888 8888888888 8888888888 8888888888
                              7777777778 8888888889 9999999990 0000000001 1111111112 2222222223 3333333334 4444444445 5555555556 6666666667 7777777778
                              1234567890 1234567890 1234567890 1234567890 1234567890 1234567890 1234567890 1234567890 1234567890 1234567890 1234567890
Crocodylus_porosus_EU727191   CCATTCTCAA ACTTTAAATG GGTAAGAAGC CTGGCTCACT GGTGTGGAGC CTGG-TGTGG AATGCGA-GT GCC-TAGTGG GCCACTTGTG GTAAGCGGAG CTGGTGCTGC
Xenopus_laevis_X02995         CTATTCTCAA ACTTTAAATG GGTAAGAAGC CCGGCTCGCT GGCTTGGAGC CGGGGCGTGG AATGCGGCAC GCCATAGTGG GCCACTTTTG GTAAGCAGAA CTGGCGCTGC
Mus_musculus_NR_046233        CTATTCTCAA ACTTTAAATG GGTAAGAAGC CCGGCTCGCT GGCGTGGAGC CGGG-CGTGG AATGCGA-GT GCC-TAGTGG GCCACTTTTG GTAAGCAGAA CTGGCGCTGC
Homo_sapiens_U13369           CTATTCTCAA ACTTTAAATG GGTAAGAAGC CCGGCTCGCT GGCGTGGAGC CGGG-CGTGG AATGCGA-GT GCC-TAGTGG GCCACTTTTG GTAAGCAGAA CTGGCGCTGC
Rattus_norvegicus_NR_046239   CTATTCTCAA ACTTTAAATG GGTAAGAAGC CCGGCTCGCT GGCGTGGAGC CGGG-CGTGG A-TGCGA-GT GCC-TAGTGG GCCACTTTTG GTAAGCAGAA CTGGCGCTGC
Gallus_gallus_KT445934.2      CTATTCTCAA ACTTTCAATG GGTAAGACGC CCGGCTCGCT GGCGTGGAGC CGGGCCGTGG AATGCGA-GC GCT-CAGTGG GCCACTTTTG GTAAGCAGAA CTGGCGCTGC
```

```
                               1111111111 1111111111 1111111111 1111111111 1111111111 1111111111 1111111111 1111111111 1111111111 1111111111 1111111111
                               1111111111 1111111111 1111111111 1111111111 1111111111 1111111111 1111111111 1111111111 1111111111 1111111111 1111111111
                               8888888888 8888888889 9999999999 9999999999 9999999999 9999999999 9999999999 9999999999 9999999999 9999999999 9999999999
                               8888888889 9999999990 0000000001 1111111112 2222222223 3333333334 4444444445 5555555556 6666666667 7777777778 8888888889
                               1234567890 1234567890 1234567890 1234567890 1234567890 1234567890 1234567890 1234567890 1234567890 1234567890 1234567890
Crocodylus_porosus_EU727191    GGAATGAACT GAATGCGAGG TTAAGGTGCT CAATGCTGAC GCTCATCAGA CCCCAGAAAA GGTGTTGGTT GATATAGACC TCAGGACGGT GGCCATGGAA GTTGGAATCT
Xenopus_laevis_X02995          GGGATGAACC GAACGCCGGG TTAAGGCGCC CGATGCCGAC GCTCATCAGA CCCCAGAAAA GGTGTTGGTT GATATAGACA GCAGGACGGT GGCCATGGAA GTCGGAATCC
Mus_musculus_NR_046233         GGGATGAACC GAACGCCGGG TTAAGGCGCC CGATGCCGAC GCTCATCAGA CCCCAGAAAA GGTGTTGGTT GATATAGACA GCAGGACGGT GGCCATGGAA GTCGGAATCC
Homo_sapiens_U13369            GGGATGAACC GAACGCCGGG TTAAGGCGCC CGATGCCGAC GCTCATCAGA CCCCAGAAAA GGTGTTGGTT GATATAGACA GCAGGACGGT GGCCATGGAA GTCGGAATCC
Rattus_norvegicus_NR_046239    GGGATGAACC GAACGCCGGG TTAAGGCGCC CGATGCCGAC GCTCATCAGA CCCCAGAAAA GGTGTTGGTT GATATAGACA GCAGGACGGT GGCCATGGAA GTCGGAATCC
Gallus_gallus_KT445934.2       GGGATGAACC GAACGCCGGG TTAAGGCGCC CGATGCCGAC GCTCATCAGA GCCCAGAAAA GGTGTTGGTT GATCTAGACA GCAGGACGGT GGCCATGGAA GTCGGAACCC

                               1111111111 1111111111 1111111111 1111111111 1111111111 1111111111 1111111111 1111111111 1111111111 1111111111 1111111111
                               1111111112 2222222222 2222222222 2222222222 2222222222 2222222222 2222222222 2222222222 2222222222 2222222222 2222222222
                               9999999990 0000000000 0000000000 0000000000 0000000000 0000000000 0000000000 0000000000 0000000000 0000000000 0000000001
                               9999999990 0000000001 1111111112 2222222223 3333333334 4444444445 5555555556 6666666667 7777777778 8888888889 9999999990
                               1234567890 1234567890 1234567890 1234567890 1234567890 1234567890 1234567890 1234567890 1234567890 1234567890 1234567890
Crocodylus_porosus_EU727191    GCTAAAGAGT GTGTAACAAC TCACCTGCCA AATCAACTAG CCCTGCAAAT GGATGGTGCT AGAGTGTTGG GCCCATACCT GGCTTTTGCC G--------- ----------
Xenopus_laevis_X02995          GCTAAGGAGT GTGTAACAAC TCACCTGCCG AATCAACTAG CCCTGAAAAT GGATGGCGCT GGAGCGTCGG GCCCATACCC GGCCGTCGCC G--------- ----------
Mus_musculus_NR_046233         GCTAAGGAGT GTGTAACAAC TCACCTGCCG AATCAACTAG CCCTGAAAAT GGATGGCGCT GGAGCGTCGG GCCCATACCC GGCCGTCGCC G-CAGTCGGA ACGGAACGGG
Homo_sapiens_U13369            GCTAAGGAGT GTGTAACAAC TCACCTGCCG AATCAACTAG CCCTGAAAAT GGATGGCGCT GGAGCGTCGG GCCCATACCC GGCCGTCGCC GGCAGTCGAG AGTGGACGGG
Rattus_norvegicus_NR_046239    GCTA-GGAGT GTGTAACAAC TCACCTGCCG AATCAACTAG CCCTGAAAAT GGATGGCGCT GGAGCGTCGG GCCCATACCC GGCCGTCGCC GGCAGTCGGA ACGG-----G
Gallus_gallus_KT445934.2       GCTAAGGAGT GTGTAACAAC TCACCTGCCG AATCAACTAG CCCTGAAAAT GGATGGCGCT GGAGCGTCGG GCCCATACCC GGCCGTCGCC G--------- ----------

                               1111111111 1111111111 1111111111 1111111111 1111111111 1111111111 1111111111 1111111111 1111111111 1111111111 1111111111
                               2222222222 2222222222 2222222222 2222222222 2222222222 2222222222 2222222222 2222222222 2222222222 2222222222 2222222222
                               1111111111 1111111111 1111111111 1111111111 1111111111 1111111111 1111111111 1111111111 1111111111 1111111112 2222222222
                               0000000001 1111111112 2222222223 3333333334 4444444445 5555555556 6666666667 7777777778 8888888889 9999999990 0000000001
                               1234567890 1234567890 1234567890 1234567890 1234567890 1234567890 1234567890 1234567890 1234567890 1234567890 1234567890
Crocodylus_porosus_EU727191    ---------- ---------- ---------- ---------- ---------- ---------- ---------- ---------- ---------- ---------- ----------
Xenopus_laevis_X02995          ---------- ---------- ---------- ---------- ---------- ---------- ---------- ---------- ---------- ---------- ----------
Mus_musculus_NR_046233         A----CGGGA GCGGC-CGCG GGTGCGCGTC TCTCGGG--- GTCGGGGGTG CGTGGCGGGG GC-------- ---------- --------CC GTCCC----- -----CCGCC
Homo_sapiens_U13369            AGCGGCGGGG GCGGCGCGCG CGCGCGCGCG TGTGGTGTGC GTCGGAGGGC GGCGGCGGCG GCGGCGGCGG GGGTGTGGGG TCCTTCCCCC GCCCCCCCCC CCACGCCTCC
Rattus_norvegicus_NR_046239    A----CGGGA GCGGC-CGCG GGCGCGCGAC CCCGGGG--- GCCGGGCGGC GTCGGCTTCG GC-------- ---------- --------CG G--------- -----CCGCC
Gallus_gallus_KT445934.2       ---------- ---------- ---------- ---------- ---------- ---------- ---------- ---------- ---------- ---------- ----------

                               1111111111 1111111111 1111111111 1111111111 1111111111 1111111111 1111111111 1111111111 1111111111 1111111111 1111111111
                               2222222222 2222222222 2222222222 2222222222 2222222222 2222222222 2222222222 2222222222 2222222222 2222222222 2222222222
                               2222222222 2222222222 2222222222 2222222222 2222222222 2222222222 2222222222 2222222222 2222222223 3333333333 3333333333
                               1111111112 2222222223 3333333334 4444444445 5555555556 6666666667 7777777778 8888888889 9999999990 0000000001 1111111112
                               1234567890 1234567890 1234567890 1234567890 1234567890 1234567890 1234567890 1234567890 1234567890 1234567890 1234567890
Crocodylus_porosus_EU727191    ---------- ---------- --------GA AATGAGAG-- --CCATGGGG GCTAGGCTGT GAT-GAGTAG GAGGGCCTCT GTGCTGGGCC TTGAAGCCTA GGGCACGGGC
Xenopus_laevis_X02995          ---------- ---------- ------GCGC TGGGTCAG-- -TCCGCGGGG GCTAGGCCGC GACTGAGTAG GAGGGCCCCG GCGGTGGGCG CGGAAGCGC- GCGCGAGGGC
Mus_musculus_NR_046233         TCCCCTCCGC GCGCCGG-GT TCGCCCCCGC GGCGTCGG-G CCCCGCGGAG CCTACGCCGC GAC-GAGTAG GAGGGCCGCT GCGGTGAGCC TTGAAGCCTA GGGCGCGGGC
Homo_sapiens_U13369            TCCCCTCCTC CCGCCCACGC CCCGCTCCCC GCCCCCGGAG CCCCGCGGAC GCTACGCCGC GAC-GAGTAG GAGGGCCGCT GCGGTGAGCC TTGAAGCCTA GGGCGCGGGC
Rattus_norvegicus_NR_046239    GCCCGTCCAC CCCCGGG-GC TC-CCCCCGC GGCGTCGG-G CCCCGCGGAG CCTACGCCGC GAC-GAGTAG GAGGGCCGCT GCGGTGAGCC TTGAAGCCTA GGGCGCGGGC
Gallus_gallus_KT445934.2       ---------- ---------- --------GC GGTGCGGA-- -GCCGCGGGG GCTACGCCGC GAC-GAGTAG GAGGGCCGCT GCGGTGCGCC TGGAAGCCTG GGGCGCGGGC
```

```
                               1111111111 1111111111 1111111111 1111111111 1111111111 1111111111 1111111111 1111111111 1111111111 1111111111 1111111111
                               2222222222 2222222222 2222222222 2222222222 2222222222 2222222222 2222222222 2222222222 2222222222 2222222222 2222222222
                               3333333333 3333333333 3333333333 3333333333 3333333333 3333333333 3333333333 3333333334 4444444444 4444444444 4444444444
                               2222222223 3333333334 4444444445 5555555556 6666666667 7777777778 8888888889 9999999990 0000000001 1111111112 2222222223
                               1234567890 1234567890 1234567890 1234567890 1234567890 1234567890 1234567890 1234567890 1234567890 1234567890 1234567890
Crocodylus_porosus_EU727191    CTGGGTGGAA CCACTGCAGG TACAGATCTT GGTGGTAGTA GCAAATATTC AAATGAGAAC TTTGAAGGCC GAAATGGAGA AGGGTTCCAT GTGAACAGCA GTTGAACATG
Xenopus_laevis_X02995          CCGGGTGGAG CCGCCGCGGG TGCAGATCTT GGTGGTAGTA GCAAATATTC AAACGAGAAC TTTGAAGGCC GAAGTGGAGA AGGGTTCCAT GTGAACAGCA GTTGAACATG
Mus_musculus_NR_046233         CCGGGTGGAG CCGCCGCAGG TGCAGATCTT GGTGGTAGTA GCAAATATTC AAACGAGAAC TTTGAAGGCC GAAGTGGAGA AGGGTTCCAT GTGAACAGCA GTTGAACATG
Homo_sapiens_U13369            CCGGGTGGAG CCGCCGCAGG TGCAGATCTT GGTGGTAGTA GCAAATATTC AAACGAGAAC TTTGAAGGCC GAAGTGGAGA AGGGTTCCAT GTGAACAGCA GTTGAACATG
Rattus_norvegicus_NR_046239    CCGGGTGGAG CCGCCGCAGG TGCAGATCTT GGTGGTAGTA GCAAATATTC AAACGAGAAC TTTGAAGGCC GAAGTGGAGA AGGGTTCCAT GTGAACAGCA GTTGAACATG
Gallus_gallus_KT445934.2       CCGGGTGGAG CCGCCGCAGG TGCAGATCTT GGTGGTAGTA GCAACTATTC AAACGAGAGC TTTGAAGGCC GAAGTGGAGC AGGGTTCCAT GTGAACAGCA GTTGAACATG

                               1111111111 1111111111 1111111111 1111111111 1111111111 1111111111 1111111111 1111111111 1111111111 1111111111 1111111111
                               2222222222 2222222222 2222222222 2222222222 2222222222 2222222222 2222222222 2222222222 2222222222 2222222222 2222222222
                               4444444444 4444444444 4444444444 4444444444 4444444444 4444444444 4444444445 5555555555 5555555555 5555555555 5555555555
                               3333333334 4444444445 5555555556 6666666667 7777777778 8888888889 9999999990 0000000001 1111111112 2222222223 3333333334
                               1234567890 1234567890 1234567890 1234567890 1234567890 1234567890 1234567890 1234567890 1234567890 1234567890 1234567890
Crocodylus_porosus_EU727191    GGTTAGTCAG TCCTAAGAGA TAGGTGAGCG CCATTCCGAA GGGACGGGCA ATGGCCTCCG TTGCCCTCAG CCGATTGCAA GGGAGTCGTG TTCAGATCCC TGAATCCAGA
Xenopus_laevis_X02995          GGTCAGTCGG TCCTAAGAGA TGGGCGAGCG CCGTTCGGAA GGGACGGGCG ATGGCCTCCG TCGCCCTCGG CCGATCGAAA GGGAGTCGGG TTCAGATCCC CGAACCCGGA
Mus_musculus_NR_046233         GGTCAGTCGG TCCTGAGAGA TGGGCGAGTG CCGTTCCGAA GGGACGGGCG ATGGCCTCCG TTGCCCTCGG CCGATCGAAA GGGAGTCGGG TTCAGATCCC CGAATCCGGA
Homo_sapiens_U13369            GGTCAGTCGG TCCTGAGAGA TGGGCGAGCG CCGTTCCGAA GGGACGGGCG ATGGCCTCCG TTGCCCTCGG CCGATCGAAA GGGAGTCGGG TTCAGATCCC CGAATCCGGA
Rattus_norvegicus_NR_046239    GGTCAGTCGG TCCTGAGAGA TGGGCGAGTG CCGTTCCGAA GGGACGGGCG ATGGCCTCCG TTGCCCTCAG CCGATCGAAA GGGAGTCGGG TTCAGATCCC CGAATCCGGA
Gallus_gallus_KT445934.2       GGTCAGTCGG TCCTAAGCGA TAGGCGAGCG CCGTTCCGAA GGGACGGGCG ATGGCCTCCG TTGCCCTCAG CCGATCGAAA GGGAGTCGGG TTCAGATCCC CGAATCCGGA

                               1111111111 1111111111 1111111111 1111111111 1111111111 1111111111 1111111111 1111111111 1111111111 1111111111 1111111111
                               2222222222 2222222222 2222222222 2222222222 2222222222 2222222222 2222222222 2222222222 2222222222 2222222222 2222222222
                               5555555555 5555555555 5555555555 5555555555 5555555555 5555555556 6666666666 6666666666 6666666666 6666666666 6666666666
                               4444444445 5555555556 6666666667 7777777778 8888888889 9999999990 0000000001 1111111112 2222222223 3333333334 4444444445
                               1234567890 1234567890 1234567890 1234567890 1234567890 1234567890 1234567890 1234567890 1234567890 1234567890 1234567890
Crocodylus_porosus_EU727191    GTGGT----- --GGTGCC-- ---------- ---------- -----ATGAG G--------- CGTCCAGTGC -AGTAATGCA ACTGATCCTG GAGAAGCCGG CGGGAGCCCC
Xenopus_laevis_X02995          GTGGCGGAGA CGGGCGCCCG CGGCCCCCCC CGACGCCTCG CGGCGGCGGG GGGGCGGGGG CGTCCAGTGC -GGCGACGCG ACCGATCCCG GAGAAGCCGG -GGGAGCCCG
Mus_musculus_NR_046233         GTGGCGGAGA TGGGCGCC-- ---------- ---------- -----GCGAG G--------- ---CCAGTGC -GGTAACGCG ACCGATCCCG GAGAAGCCGG CGGGAGGCCT
Homo_sapiens_U13369            GTGGCGGAGA TGGGCGCC-- ---------- ---------- -----GCGAG G--------- CGTCCAGTGC -GGTAACGCG ACCGATCCCG GAGAAGCCGG CGGGAG-CCC
Rattus_norvegicus_NR_046239    GTGGCGGAGA TGGGCGCC-- ---------- ---------- -----GCGAG G--------- CGTCCAGTGC CGGTAACGCG ACCGATCCCG GAGAAGCCGG CGGGAG--CC
Gallus_gallus_KT445934.2       GCGGCGGAGA CGGGCGCC-- ---------- ---------- -----GCGAG G--------- CGCCCAGTGC -GGTAACGCA AGCGATCCCG GAGAAGCCGG CGGGAG-CCC

                               1111111111 1111111111 1111111111 1111111111 1111111111 1111111111 1111111111 1111111111 1111111111 1111111111 1111111111
                               2222222222 2222222222 2222222222 2222222222 2222222222 2222222222 2222222222 2222222222 2222222222 2222222222 2222222222
                               6666666666 6666666666 6666666666 6666666666 6666666667 7777777777 7777777777 7777777777 7777777777 7777777777 7777777777
                               5555555556 6666666667 7777777778 8888888889 9999999990 0000000001 1111111112 2222222223 3333333334 4444444445 5555555556
                               1234567890 1234567890 1234567890 1234567890 1234567890 1234567890 1234567890 1234567890 1234567890 1234567890 1234567890
Crocodylus_porosus_EU727191    CAGGGAGAGT TCTCCTTTCT TTGTGAAGGG CAGGGGGCCC TGAATCGGGT TTGCCCCAAG GGACGG---- -------TGA AAAGCATCGC GGTTCCAGCA GCGTGTGGTG
Xenopus_laevis_X02995          GGAGAAGAGT TCTCTTTTCT TTGTGAAGGG CAGGGCCGCC CTGAATGGGT TCGCCCCGAG AGAGGGGCCC GCGCCGTTGG AAAGCGTCGC GGTTCCGGCG GCGTCCGGTG
Mus_musculus_NR_046233         CGGGGAGAGT TCTCTTTTCT TTGTGAAGGG CAGGGCGCCC TGGAATGGGT TCGCCCCGAG AGAGGGGCCC GTGCC-TTGG AAAGCGTCGC GGTTCCGGCG GCGTCCGGTG
Homo_sapiens_U13369            CGGGGAGAGT TCTCTTTTCT TTGTGAAGGG CAGGGCGCCC TGGAATGGGT TCGCCCCGAG AGAGGGGCCC GTGCC-TTGG AAAGCGTCGC GGTTCCGGCG GCGTCCGGTG
Rattus_norvegicus_NR_046239    CGGGGAGAGT TCTCTTTTCT TTGTGAAGGG CAGGGCGCCT TGGAATGGGT TCGCCCCGAG AGAGGGGCCC GTGCC-TTGG AAAGCGTCGC AGTTCCGGCG GCGTCCGGTG
Gallus_gallus_KT445934.2       CGGGGAGAGT TCTCTTTTCT TTGTGAAGGG CCGGGCGCCC TGGAACGGGT TCGCCCCGAG AGAGGGGCCC GCGCC-TTGG AAAGCGTCGC GGTTCCGGCG GCGTCCGGTG
```

```
                              1111111111 1111111111 1111111111 1111111111 1111111111 1111111111 1111111111 1111111111 1111111111 1111111111 1111111111
                              2222222222 2222222222 2222222222 2222222222 2222222222 2222222222 2222222222 2222222222 2222222222 2222222222 2222222222
                              7777777777 7777777777 7777777777 7777777778 8888888888 8888888888 8888888888 8888888888 8888888888 8888888888 8888888888
                              6666666667 7777777778 8888888889 9999999990 0000000001 1111111112 2222222223 3333333334 4444444445 5555555556 6666666667
                              1234567890 1234567890 1234567890 1234567890 1234567890 1234567890 1234567890 1234567890 1234567890 1234567890 1234567890
Crocodylus_porosus_EU727191   AGTT-CTCAC GGGTCCTTGA AAATCGGGGG GAGAAGGTGT AAATCTC-AG GCTGGGCCGT ACCCATATCC ACAGCAGGTC TCCAAGGTGA ACAGCCTCTG GCGTGTTAGA
Xenopus_laevis_X02995         AGCTTCTCGC TGGCCCTTGA AAATCCGGGG GAGAGGGTGT AAATCTCTGC GCCGGGCCGT ACCCATATCC GCAGCAGGTC TCCAAGGTGA ACAGCCTCTG GCATGTTAGA
Mus_musculus_NR_046233        AGCT-CTCGC TGGCCCTTGA AAATCCGGGG GAGAGGGTGT AAATCTC-GC GCCGGGCCGT ACCCATATCC GCAGCAGGTC TCCAAGGTGA ACAGCCTCTG GCATGTTGGA
Homo_sapiens_U13369           AGCT-CTCGC TGGCCCTTGA AAATCCGGGG GAGAGGGTGT AAATCTC-GC GCCGGGCCGT ACCCATATCC GCAGCAGGTC TCCAAGGTGA ACAGCCTCTG GCATGTTGGA
Rattus_norvegicus_NR_046239   AGCT-CTCGC TGGCCCTTGA AAATCCGGGG GAGAGGGTGT AAATCTC-GC GCCGGGCCGT ACCCATATCC GCAGCAGGTC TCCAAGGTGA AC-GCCTCTG GCATGTTGGA
Gallus_gallus_KT445934.2      AGCT-CTCGC TGGCCCGTGA AAATCCGGGG GAGAGGGTGT AAATCTC-GC GCCGGGCCGT ACCCATATCC GCAGCAGGTC TCCAAGGTGA ACAGCCTCTG GCATGTTGGA

                              1111111111 1111111111 1111111111 1111111111 1111111111 1111111111 1111111111 1111111111 1111111111 1111111111 1111111111
                              2222222222 2222222222 2222222222 2222222222 2222222222 2222222222 2222222222 2222222222 2222222222 2222222222 2222222222
                              8888888888 8888888888 8888888889 9999999999 9999999999 9999999999 9999999999 9999999999 9999999999 9999999999 9999999999
                              7777777778 8888888889 9999999990 0000000001 1111111112 2222222223 3333333334 4444444445 5555555556 6666666667 7777777778
                              1234567890 1234567890 1234567890 1234567890 1234567890 1234567890 1234567890 1234567890 1234567890 1234567890 1234567890
Crocodylus_porosus_EU727191   ACAGTGTAGG TAAGGGAAGT CAGCAAGCCG GATCTGTAAC TTTGGGATAA AGATTGGCTC TAAGGGCTGG GTTGGTTGGG ATGGGGCACA AAG-TAGAGC TGGGCATGAG
Xenopus_laevis_X02995         ACAATGTAGG TAAGGGAAGT CGGCAAGTCA GATCCGTAAC TTCGGGATAA GGATTGGCTC TAAGGGCTGG GTCGGTCGGG CTGGGGCGCG AAG-CGGGGC TGGGC-CGCG
Mus_musculus_NR_046233        ACAATGTAGG TAAGGGAAGT CGGCAAGCCG GATCCGTAAC TTCGGGATAA GGATTGGCTC TAAGGGCTGG GTCGGTCGGG CTGGGGCGCG AAG-CGGGGC TGGGCGCGCG
Homo_sapiens_U13369           ACAATGTAGG TAAGGGAAGT CGGCAAGCCG GATCCGTAAC TTCGGGATAA GGATTGGCTC TAAGGGCTGG GTCGGTCGGG CTGGGGCGCG AAG-CGGGGC TGGGCGCGCG
Rattus_norvegicus_NR_046239   ACAATGTAGG TAAGGGAAG- CGGCAAGCCG GATCCGTAAC TTCGGGATAA GGATTGGCTC TAAGGGCTGG GTCGGTCGGG CTGGGGCGCG AAGGCGGGGC TGGGCGCGCG
Gallus_gallus_KT445934.2      CCAATGTAGG TAAGGGAAGT CGGCAAGCCG GATCCGTAAC TTCGGGATAA GGATTGGCTC TAAGGGCTGG GTCGGTCGGG CTGGGGCGCG AAG-CGGGGC TGGGCGCGCG

                              1111111111 1111111111 1111111111 1111111111 1111111111 1111111111 1111111111 1111111111 1111111111 1111111111 1111111111
                              2222222222 2222222223 3333333333 3333333333 3333333333 3333333333 3333333333 3333333333 3333333333 3333333333 3333333333
                              9999999999 9999999990 0000000000 0000000000 0000000000 0000000000 0000000000 0000000000 0000000000 0000000000 0000000000
                              8888888889 9999999990 0000000001 1111111112 2222222223 3333333334 4444444445 5555555556 6666666667 7777777778 8888888889
                              1234567890 1234567890 1234567890 1234567890 1234567890 1234567890 1234567890 1234567890 1234567890 1234567890 1234567890
Crocodylus_porosus_EU727191   CTGTGGCTGG ATGAGGCA-- ----CCTAC- ---------- ---------- ---------- ---------- ---------- ---------- ---------- ----------
Xenopus_laevis_X02995         CCGCGGCTGG ACGAAGG--- --CGCCCCC- ---------- GTGGCGCTC- ---------- ---------- ---------- ---------- ---------- ----------
Mus_musculus_NR_046233        CCGCGGCTGG ACGAGGCGCC GCCGCCCTC- TCCCACGTCC GGGGAGACCC CCCGTCCTTT CCGCCCGGGC CCGCCCTCCC CTCTT-CCCC GCGGGGCCCC GTCGTCCCCC
Homo_sapiens_U13369           CCGCGGCTGG ACGAGGCGCG --CGCCCCC- -CCCACGCCC GGGGCACCCC CC-----TCG CGGCCCTCCC CCGCCCCACC CGCGCGCGCC GCTCGCTCCC TCCCCACCCC
Rattus_norvegicus_NR_046239   CCGCGGCTGG ACGAGGCGCC GCCGCCCCCC TCCCACGTCC GGGGAGACCC CC--TCCTTT CCGCCCGGGC CCGCCCTCCC CTCT--CCCC GCGGGGCCCC GCCGTCCCCC
Gallus_gallus_KT445934.2      CCGCGGCTGG ACGAGGCGC- --CGCCCGC- ---------- ---------- ---------- ---------- ---------- ---------- ---------- ----------

                              1111111111 1111111111 1111111111 1111111111 1111111111 1111111111 1111111111 1111111111 1111111111 1111111111 1111111111
                              3333333333 3333333333 3333333333 3333333333 3333333333 3333333333 3333333333 3333333333 3333333333 3333333333 3333333333
                              0000000001 1111111111 1111111111 1111111111 1111111111 1111111111 1111111111 1111111111 1111111111 1111111111 1111111112
                              9999999990 0000000001 1111111112 2222222223 3333333334 4444444445 5555555556 6666666667 7777777778 8888888889 9999999990
                              1234567890 1234567890 1234567890 1234567890 1234567890 1234567890 1234567890 1234567890 1234567890 1234567890 1234567890
Crocodylus_porosus_EU727191   ---------- ---------- ---------- -----TCCCC TTC------- ---------- ---------- ---------- ---------- ---------- ----------
Xenopus_laevis_X02995         ---------- ----TTTCCC TCCTCCCGCC CCCCTCTCTC TCC------- ---------- ---------- -GGCCCCCCC TCGCGGGGGG CCGCGGGGGC GGG-------
Mus_musculus_NR_046233        GCGTCGTCGC CAC--CTCTC TTCCCCCCTC CTTCTTCCC- GTC-----GG GGGGCGGGTC GG----GGGT CGGCGCGCG- GCGCG-GGCT CCGGGGCGGC GGGTCCAACC
Homo_sapiens_U13369           GCGCCCTCTC TCTCTCTCTC TCCCCCGCTC CCCGTCCTCC CCCCTCCCCG GGGGAGCGCC GCGTG-GGGG CGCGGCGGG- GGGAGAAGGG TCGGGGCGGC AGGGGCCGCG
Rattus_norvegicus_NR_046239   GCGTCGTCGC CGTGGTCCCC TCCTCCCTCC CTTCTTCCCC GTCCGCGGGG GGGACGGGGC GGGTGCGGGG GGGCGCGCGC GCGCGCGGCC CAGGGGCGGC GGGTCCAACC
Gallus_gallus_KT445934.2      ---------- ---------- CCCCGCCCCC CCTTTCCCCG CTC------- ---------- ---------- --CCGCTC-- ---------G CCGGGGCGCC GGG-------
```

```
                                1111111111 1111111111 1111111111 1111111111 1111111111 1111111111 1111111111 1111111111 1111111111 1111111111 1111111111
                                3333333333 3333333333 3333333333 3333333333 3333333333 3333333333 3333333333 3333333333 3333333333 3333333333 3333333333
                                2222222222 2222222222 2222222222 2222222222 2222222222 2222222222 2222222222 2222222222 2222222222 2222222223 3333333333
                                0000000001 1111111112 2222222223 3333333334 4444444445 5555555556 6666666667 7777777778 8888888889 9999999990 0000000001
                                1234567890 1234567890 1234567890 1234567890 1234567890 1234567890 1234567890 1234567890 1234567890 1234567890 1234567890
Crocodylus_porosus_EU727191     ---------- ---------- ---------- ---------- ---------- ---------- --CCAGGGGG --------GT GGTGGCAACT CTGGATGCAA TCCGGGCCCT
Xenopus_laevis_X02995           ---------- ---------- ---------- ---------- ---------G GGGCGCGGGG GGCCGGGAGC GCCCGGCGGC GGC---GACT CTGGACGCGC GCCGGGCCCT
Mus_musculus_NR_046233          CCGCGGGGGT TCCGGAGCGG GAGGAACCAG CGG-TCCCCG GTGGGGCGGG GGGCCCGGAC ACTCGGGGGG --CCGGCGGC GGCGGCGACT CTGGACGCGA GCCGGGCCCT
Homo_sapiens_U13369             CGGCGGCCG- -CCGGGGCGG CCGGCGGGGG CAGGTCCCCG CGAGGG---G GGCCCCGGGG ACCCGGGGGG --CCGGCGGC GGCGCGGACT CTGGACGCGA GCCGGGCCCT
Rattus_norvegicus_NR_046239     CCGCGCGGG- -CCGGAGCGG GGGGAACCCG CGGGCCCCCG GTGGGG--GG GGGCCCGGAC ACCCGGGGGG GACCGGCGGC GGCGGCGACT CTGGACGCGA GCCGGGCCCT
Gallus_gallus_KT445934.2        ---------- ---------- ---------- ---------- ---------- GGGGGGGGTC AGCGGGCGGC -----GCGGC GGCGGCGACT CTGGACGCGC GCCGGGCCCT

                                1111111111 1111111111 1111111111 1111111111 1111111111 1111111111 1111111111 1111111111 1111111111 1111111111 1111111111
                                3333333333 3333333333 3333333333 3333333333 3333333333 3333333333 3333333333 3333333333 3333333333 3333333333 3333333333
                                3333333333 3333333333 3333333333 3333333333 3333333333 3333333333 3333333333 3333333333 3333333334 4444444444 4444444444
                                1111111112 2222222223 3333333334 4444444445 5555555556 6666666667 7777777778 8888888889 9999999990 0000000001 1111111112
                                1234567890 1234567890 1234567890 1234567890 1234567890 1234567890 1234567890 1234567890 1234567890 1234567890 1234567890
Crocodylus_porosus_EU727191     TCCTGTGGAT TGCCTCAGCT GCAGCGGGCT TCAC----CT GCCTCT---- ---------- ---------- ---------- ---CC----- ---------- -TCTTCCAC-
Xenopus_laevis_X02995           TCCTGTGGAT CGCCCCAGCT GCGGCGCGCG --------CC TCTCCC---- ---------- ------CCGC GCCGTCCCC- -CTCC----- ----TGCGCC TCCCCCCGTC
Mus_musculus_NR_046233          TCCCGTGGAT CGCCTCAGCT GCGGCGGGCG TCGC--GGCC GCTCCCGGGG AGCCCGGCGG GTG--CCGGC GCGGGTCCC- -CTCCCCGCG GGGCCTCGCT CCACCCCCCC
Homo_sapiens_U13369             TCCCGTGGAT CGCCCCAGCT GCGGCGGGCG TCGC--GGCC GCCCCCGGGG AGCCCGGCGG -CGGCGCGGC GCGCCCCCCA CCCCCACCCC ACGTCTCGGT CGCGCGCGCG
Rattus_norvegicus_NR_046239     TCCCGTGGAT CGCCCCAGCT GCGGCGGGCG TCGC--GGCC GCTCCCGGGG AGCCCGGCGG GTCGCCCGGC GGGGTTTTC- -CTCC----- -GGCCTCGTC CTCCCCCTTC
Gallus_gallus_KT445934.2        TCCCGTGGAT CGCCCCAGCT GCGGCGGGCG CCGCTCGCCC CCCTCCTTGC CCCTCCGCCC CCCCGCTCCC GGCGCCCCTC CCGTCGGCCG TCGTCCCGGC CGCCCCCCGT

                                1111111111 1111111111 1111111111 1111111111 1111111111 1111111111 1111111111 1111111111 1111111111 1111111111 1111111111
                                3333333333 3333333333 3333333333 3333333333 3333333333 3333333333 3333333333 3333333333 3333333333 3333333333 3333333333
                                4444444444 4444444444 4444444444 4444444444 4444444444 4444444444 4444444444 4444444445 5555555555 5555555555 5555555555
                                2222222223 3333333334 4444444445 5555555556 6666666667 7777777778 8888888889 9999999990 0000000001 1111111112 2222222223
                                1234567890 1234567890 1234567890 1234567890 1234567890 1234567890 1234567890 1234567890 1234567890 1234567890 1234567890
Crocodylus_porosus_EU727191     ---------- ---------- ---------- ---------- ------GGGG GTGGG----- ---------- ---------- ---------- ---------- ----------
Xenopus_laevis_X02995           A--------- ---------- ---------- ---------- ------GGGG ACGGG----- ---------- ---------- ---------- --GCGCGG-- ----------
Mus_musculus_NR_046233          ATCGCC---- ---------- --------TC TCCCGAGGTG CGTGGCGGGG GCGGGCGGGC GTGTCCCGCG C--------- ---------- --GTGTGGGG GGAACCTC--
Homo_sapiens_U13369             TCCGCTGGGG GCGGGAGCGG TCGGGCGGCG GCGGTCGGCG GGCGGCGGGG CGGGGCGGTT CGTCCCCCCG CCCTACCCCC CCGGCCCCGT CCGCCCCCCG TTCCCCCCTC
Rattus_norvegicus_NR_046239     ----CC---- ---------- --------CC TCC------- ----GCGGGG TCGGGGG--- ---TTCCCGG G--------- ---------- --GTTCGGGG TTCTCCTC--
Gallus_gallus_KT445934.2        CCCGAGCG-- ---------- --------CC CTCCCCGCGA GGGCGCGAGG GGCGGCGGCG GCGGCCGCGG ---------- ---------- --GCGCGGCG GCGG------

                                1111111111 1111111111 1111111111 1111111111 1111111111 1111111111 1111111111 1111111111 1111111111 1111111111 1111111111
                                3333333333 3333333333 3333333333 3333333333 3333333333 3333333333 3333333333 3333333333 3333333333 3333333333 3333333333
                                5555555555 5555555555 5555555555 5555555555 5555555555 5555555555 5555555556 6666666666 6666666666 6666666666 6666666666
                                3333333334 4444444445 5555555556 6666666667 7777777778 8888888889 9999999990 0000000001 1111111112 2222222223 3333333334
                                1234567890 1234567890 1234567890 1234567890 1234567890 1234567890 1234567890 1234567890 1234567890 1234567890 1234567890
Crocodylus_porosus_EU727191     ---------G TGGGGGGAC- ---------- ---------- ---------- ---------- -----GGCAT TCC------- ---------- ---------- -----ACCTC
Xenopus_laevis_X02995           --------CG CGGGGCGGC- ---------- ---------- ---------- --------CG GGGCGGGCCC G--------- ---------- ---------- -----GCCTC
Mus_musculus_NR_046233          --------CG CGTCGGTGT- ---------- ---------- ---------- -----TCCCC CGCCGGGTCC GCCCCCCGGG CCGCGGTTTT CCGCGCGGCG CCCCCGCCTC
Homo_sapiens_U13369             CTCCTCGGCG CGCGGCGGCG GCGGCGGCAG GCGGCGGAGG GGCCGCGGGC CGGTCCCCCC CGCCGGGTCC GCCCCCGGGG CCGCGGTTCC GCGCG---CG CCTC-GCCTC
Rattus_norvegicus_NR_046239     --------CG CGCGGCGGT- ---------- ---------- ---------- -----TCCCC CGCCGGGTGC GCCCCCCGGG C-GCGGTTTC CCGCG-GCCG CCC--GCCTC
Gallus_gallus_KT445934.2        --------CG GGGGGGGGCC C--------- ---------- --------GC CGGCGGCGCC GGGCGGGGCG GTCCCGGGCG GGGGGGGTCT CCGGGCCGGC GCCCCGCCTC
```

```
                              1111111111 1111111111 1111111111 1111111111 1111111111 1111111111 1111111111 1111111111 1111111111 1111111111 1111111111
                              3333333333 3333333333 3333333333 3333333333 3333333333 3333333333 3333333333 3333333333 3333333333 3333333333 3333333333
                              6666666666 6666666666 6666666666 6666666666 6666666666 6666666667 7777777777 7777777777 7777777777 7777777777 7777777777
                              4444444445 5555555556 6666666667 7777777778 8888888889 9999999990 0000000001 1111111112 2222222223 3333333334 4444444445
                              1234567890 1234567890 1234567890 1234567890 1234567890 1234567890 1234567890 1234567890 1234567890 1234567890 1234567890
Crocodylus_porosus_EU727191   AGCCGGCACC TAGCCGCTGA CTTAGAACCA GCGCAGACCA GGGGAATCTG ACTGTTTAAT TCAAACAAAG CATCGCAAAG GCCTGCGGTG GGTGTTGACG CAATGTGATT
Xenopus_laevis_X02995         GGCCGGCGCC TAGCAGCTGA CTTAGAACTG GTGCGGAC-- AGGGAATCCG ACTGTTTAAT TAAAACAAAG CATCGCGAAG GCCCGAGGCG GGTGTTGACG CGATGTGATT
Mus_musculus_NR_046233        GGCCGGCGCC TAGCAGCCGA CTTAGAACTG GTGCGGACCA GGGGAATCCG ACTGTTTAAT TAAAACAAAG CATCGCGAAG GCCCGCGGCG GGTGTTGACG CGATGTGATT
Homo_sapiens_U13369           GGCCGGCGCC TAGCAGCCGA CTTAGAACTG GTGCGGACCA GGGGAATCCG ACTGTTTAAT TAAAACAAAG CATCGCGAAG GCCCGCGGCG GGTGTTGACG CGATGTGATT
Rattus_norvegicus_NR_046239   GGCCGGCGCC TAGCAGCCGA CTTAGAACTG GTGCGGACTA GGGGAATCCG ACTGTTTAAT TAAAACAAAG CATCGCGAAG GCCCGCGGCG GGTGTTGACG CGATGTGATT
Gallus_gallus_KT445934.2      GGCCGGCGCC TAGCAGCCGG CTTAGAACTG GTGCGGACCA GGGGAATCCG ACTGTTTAAT TAAAACAAAG CATCGCGAAG GCCCGCGGCG GGTGTTGACG CGATGTGATT

                              1111111111 1111111111 1111111111 1111111111 1111111111 1111111111 1111111111 1111111111 1111111111 1111111111 1111111111
                              3333333333 3333333333 3333333333 3333333333 3333333333 3333333333 3333333333 3333333333 3333333333 3333333333 3333333333
                              7777777777 7777777777 7777777777 7777777777 7777777778 8888888888 8888888888 8888888888 8888888888 8888888888 8888888888
                              5555555556 6666666667 7777777778 8888888889 9999999990 0000000001 1111111112 2222222223 3333333334 4444444445 5555555556
                              1234567890 1234567890 1234567890 1234567890 1234567890 1234567890 1234567890 1234567890 1234567890 1234567890 1234567890
Crocodylus_porosus_EU727191   TCTGCCCAGT GCTCTGAATG TCAAAGTGAA GAAATTCAAT GAAGCATGGG TAAACAGTGG GAGTAACTAT GACTCTCGTA ATATGACTCT ATTAAGGGAG GCGCCTTCCC
Xenopus_laevis_X02995         TCTGCCCAGT GCTCTGAATG TCAAAGTGAA GAAATTCAAT GAAGCGCGGG TAAACGGCGG GAGTAACTAT GACTCTCTTA A--------- ---------- ----------
Mus_musculus_NR_046233        TCTGCCCAGT GCTCTGAATG TCAAAGTGAA GAAATTCAAT GAAGCGCGGG TAAACGGCGG GAGTAACTAT GACTCTCTTA A--------- ---------- ----------
Homo_sapiens_U13369           TCTGCCCAGT GCTCTGAATG TCAAAGTGAA GAAATTCAAT GAAGCGCGGG TAAACGGCGG GAGTAACTAT GACTCTCTTA A--------- ---------- ----------
Rattus_norvegicus_NR_046239   TCTGCCCAGT GCTCAGAATG TCAAAGTGAA GAAATTCAAT GAAGCGCGGG TAAACGGCGG GAGTAACTAT GACTCTCTTA A--------- ---------- ----------
Gallus_gallus_KT445934.2      TCTGCCCAGT GCTCTGAATG TCAAAGTGAA GAAATTCAAT GAAGCGCGGG TAAACGGCGG GAGTAACTAT GACTCTCTTA A--------- ---------- ----------

                              1111111111 1111111111 1111111111 1111111111 1111111111 1111111111 1111111111 1111111111 1111111111 1111111111 1111111111
                              3333333333 3333333333 3333333333 3333333333 3333333333 3333333333 3333333333 3333333333 3333333333 3333333333 3333333333
                              8888888888 8888888888 8888888888 8888888889 9999999999 9999999999 9999999999 9999999999 9999999999 9999999999 9999999999
                              6666666667 7777777778 8888888889 9999999990 0000000001 1111111112 2222222223 3333333334 4444444445 5555555556 6666666667
                              1234567890 1234567890 1234567890 1234567890 1234567890 1234567890 1234567890 1234567890 1234567890 1234567890 1234567890
Crocodylus_porosus_EU727191   TAGGGGGAAA TGGTCTGGTT ACCCAGTTGC AGCAGGTTTG TGCCAGGTAC CTCCACGTGG TTCTCACTGG GTATCAAAGG CCTGTTGATA GCTCAACCTT GGTACAATGC
Xenopus_laevis_X02995         ---------- ---------- ---------- ---------- ---------- ---------- ---------- ---------- ---------- ---------- ----------
Mus_musculus_NR_046233        ---------- ---------- ---------- ---------- ---------- ---------- ---------- ---------- ---------- ---------- ----------
Homo_sapiens_U13369           ---------- ---------- ---------- ---------- ---------- ---------- ---------- ---------- ---------- ---------- ----------
Rattus_norvegicus_NR_046239   ---------- ---------- ---------- ---------- ---------- ---------- ---------- ---------- ---------- ---------- ----------
Gallus_gallus_KT445934.2      ---------- ---------- ---------- ---------- ---------- ---------- ---------- ---------- ---------- ---------- ----------

                              1111111111 1111111111 1111111111 1111111111 1111111111 1111111111 1111111111 1111111111 1111111111 1111111111 1111111111
                              3333333333 3333333333 3333333334 4444444444 4444444444 4444444444 4444444444 4444444444 4444444444 4444444444 4444444444
                              9999999999 9999999999 9999999990 0000000000 0000000000 0000000000 0000000000 0000000000 0000000000 0000000000 0000000000
                              7777777778 8888888889 9999999990 0000000001 1111111112 2222222223 3333333334 4444444445 5555555556 6666666667 7777777778
                              1234567890 1234567890 1234567890 1234567890 1234567890 1234567890 1234567890 1234567890 1234567890 1234567890 1234567890
Crocodylus_porosus_EU727191   AGCCAAGGAG TTTTTCCCAG GGACACTTCA CTCCCCACCA GCTGCCTCAC AAGCTCCTGG GGGTAACTGC CTCAATTGTG AAAAACTCAA AGCCTGATGG TGGGTGCTAG
Xenopus_laevis_X02995         ---------- ---------- ---------- ---------- ---------- ---------- ---------- ---------- ---------- ---------- ----------
Mus_musculus_NR_046233        ---------- ---------- ---------- ---------- ---------- ---------- ---------- ---------- ---------- ---------- ----------
Homo_sapiens_U13369           ---------- ---------- ---------- ---------- ---------- ---------- ---------- ---------- ---------- ---------- ----------
Rattus_norvegicus_NR_046239   ---------- ---------- ---------- ---------- ---------- ---------- ---------- ---------- ---------- ---------- ----------
Gallus_gallus_KT445934.2      ---------- ---------- ---------- ---------- ---------- ---------- ---------- ---------- ---------- ---------- ----------
```

```
                              1111111111 1111111111 1111111111 1111111111 1111111111 1111111111 1111111111 1111111111 1111111111 1111111111 1111111111
                              4444444444 4444444444 4444444444 4444444444 4444444444 4444444444 4444444444 4444444444 4444444444 4444444444 4444444444
                              0000000000 0000000001 1111111111 1111111111 1111111111 1111111111 1111111111 1111111111 1111111111 1111111111 1111111111
                              8888888889 9999999990 0000000001 1111111112 2222222223 3333333334 4444444445 5555555556 6666666667 7777777778 8888888889
                              1234567890 1234567890 1234567890 1234567890 1234567890 1234567890 1234567890 1234567890 1234567890 1234567890 1234567890
Crocodylus_porosus_EU727191   AGGAATCTTG CTGAAACTTG TTGGTGCCTG CAGTTGTCTG GCAGGTGGGG TGGGTGAAAC ATACTGGAAC TGACAACCAG GCAAGGGGGA TAGCCCCTCT TCTAGGGGTG
Xenopus_laevis_X02995         ---------- ---------- ---------- ---------- ---------- ---------- ---------- ---------- ---------- ---------- ----------
Mus_musculus_NR_046233        ---------- ---------- ---------- ---------- ---------- ---------- ---------- ---------- ---------- ---------- ----------
Homo_sapiens_U13369           ---------- ---------- ---------- ---------- ---------- ---------- ---------- ---------- ---------- ---------- ----------
Rattus_norvegicus_NR_046239   ---------- ---------- ---------- ---------- ---------- ---------- ---------- ---------- ---------- ---------- ----------
Gallus_gallus_KT445934.2      ---------- ---------- ---------- ---------- ---------- ---------- ---------- ---------- ---------- ---------- ----------

                              1111111111 1111111111 1111111111 1111111111 1111111111 1111111111 1111111111 1111111111 1111111111 1111111111 1111111111
                              4444444444 4444444444 4444444444 4444444444 4444444444 4444444444 4444444444 4444444444 4444444444 4444444444 4444444444
                              1111111112 2222222222 2222222222 2222222222 2222222222 2222222222 2222222222 2222222222 2222222222 2222222222 2222222223
                              9999999990 0000000001 1111111112 2222222223 3333333334 4444444445 5555555556 6666666667 7777777778 8888888889 9999999990
                              1234567890 1234567890 1234567890 1234567890 1234567890 1234567890 1234567890 1234567890 1234567890 1234567890 1234567890
Crocodylus_porosus_EU727191   GTGGCCTGGA GGGCAGAAGA CTTCTGTCCC CATGCCTTGT GCTGATAGAG GGGAGTCTCA GTGACCGGTG CAAGTGGAAC TTGAAGGTTA GTCAGTTGGG TGCAACATCC
Xenopus_laevis_X02995         ---------- ---------- ---------- ---------- ---------- ---------- ---------- ---------- ---------- ---------- ----------
Mus_musculus_NR_046233        ---------- ---------- ---------- ---------- ---------- ---------- ---------- ---------- ---------- ---------- ----------
Homo_sapiens_U13369           ---------- ---------- ---------- ---------- ---------- ---------- ---------- ---------- ---------- ---------- ----------
Rattus_norvegicus_NR_046239   ---------- ---------- ---------- ---------- ---------- ---------- ---------- ---------- ---------- ---------- ----------
Gallus_gallus_KT445934.2      ---------- ---------- ---------- ---------- ---------- ---------- ---------- ---------- ---------- ---------- ----------

                              1111111111 1111111111 1111111111 1111111111 1111111111 1111111111 1111111111 1111111111 1111111111 1111111111 1111111111
                              4444444444 4444444444 4444444444 4444444444 4444444444 4444444444 4444444444 4444444444 4444444444 4444444444 4444444444
                              3333333333 3333333333 3333333333 3333333333 3333333333 3333333333 3333333333 3333333333 3333333333 3333333334 4444444444
                              0000000001 1111111112 2222222223 3333333334 4444444445 5555555556 6666666667 7777777778 8888888889 9999999990 0000000001
                              1234567890 1234567890 1234567890 1234567890 1234567890 1234567890 1234567890 1234567890 1234567890 1234567890 1234567890
Crocodylus_porosus_EU727191   CGGCTGCATG CCCAGAAAGG TGGGGGGTGA GGATCAGGAC TACTCACCTC CAGTTCTGCT GAGCATAAAG CTAACCAGAA CTGATGATCA GGCAAGGCGG GCTCACCCCA
Xenopus_laevis_X02995         ---------- ---------- ---------- ---------- ---------- ---------- ---------- ---------- ---------- ---------- ----------
Mus_musculus_NR_046233        ---------- ---------- ---------- ---------- ---------- ---------- ---------- ---------- ---------- ---------- ----------
Homo_sapiens_U13369           ---------- ---------- ---------- ---------- ---------- ---------- ---------- ---------- ---------- ---------- ----------
Rattus_norvegicus_NR_046239   ---------- ---------- ---------- ---------- ---------- ---------- ---------- ---------- ---------- ---------- ----------
Gallus_gallus_KT445934.2      ---------- ---------- ---------- ---------- ---------- ---------- ---------- ---------- ---------- ---------- ----------

                              1111111111 1111111111 1111111111 1111111111 1111111111 1111111111 1111111111 1111111111 1111111111 1111111111 1111111111
                              4444444444 4444444444 4444444444 4444444444 4444444444 4444444444 4444444444 4444444444 4444444444 4444444444 4444444444
                              4444444444 4444444444 4444444444 4444444444 4444444444 4444444444 4444444444 4444444444 4444444445 5555555555 5555555555
                              1111111112 2222222223 3333333334 4444444445 5555555556 6666666667 7777777778 8888888889 9999999990 0000000001 1111111112
                              1234567890 1234567890 1234567890 1234567890 1234567890 1234567890 1234567890 1234567890 1234567890 1234567890 1234567890
Crocodylus_porosus_EU727191   CTTCCTGGAA TGGCAGCCTG GAATAATGGA TGGTTTCTGG CCCCCCCATC TTGTGCTCAC AGAGGGGAGC CTCAGTGACC GGTACAAGCT AAACTTGATG GTTAGTCAGT
Xenopus_laevis_X02995         ---------- ---------- ---------- ---------- --------GG TAGCCAAATG CCTCGTCATC TAATTAGTGA CGCGCATGAA TGGATGAACG AGATTCCCAC
Mus_musculus_NR_046233        ---------- ---------- ---------- ---------- --------GG TAGCCAAATG CCTCGTCATC TAATTAGTGA CGCGCATGAA TGGATGAACG AGATTCCCAC
Homo_sapiens_U13369           ---------- ---------- ---------- ---------- --------GG TAGCCAAATG CCTCGTCATC TAATTAGTGA CGCGCATGAA TGGATGAACG AGATTCCCAC
Rattus_norvegicus_NR_046239   ---------- ---------- ---------- ---------- --------GG TAGCCAAATG CCTCGTCATC TAATTAGTGA CGCGCATGAA TGGATGAACG AGATTCCCAC
Gallus_gallus_KT445934.2      ---------- ---------- ---------- ---------- --------GG TAGCCAAATG CCTCGTCATC TAATTAGTGA CGCGCATGAA TGGATGAACG AGATTCCCAC
```

```
                               1111111111 1111111111 1111111111 1111111111 1111111111 1111111111 1111111111 1111111111 1111111111 1111111111 1111111111
                               4444444444 4444444444 4444444444 4444444444 4444444444 4444444444 4444444444 4444444444 4444444444 4444444444 4444444444
                               5555555555 5555555555 5555555555 5555555555 5555555555 5555555555 5555555555 5555555556 6666666666 6666666666 6666666666
                               2222222223 3333333334 4444444445 5555555556 6666666667 7777777778 8888888889 9999999990 0000000001 1111111112 2222222223
                               1234567890 1234567890 1234567890 1234567890 1234567890 1234567890 1234567890 1234567890 1234567890 1234567890 1234567890
Crocodylus_porosus_EU727191    CAGATGCAAC ATCCTGGCTG CATGCCCACT GAAGCCTGTG ACTACCCATT CCTGAAGGCA TGAGGCATGT GTACTGTTCC AGGAGGGATG ACATCCTGTG ATTGGGACAG
Xenopus_laevis_X02995          TGTCCCTACC TACTATCTAG CGAAACCACA GCCAAGGGAA CGGGCTTGGC GGAATCAGCG GGGAAAGAAG ACCCTGTTGA GCTTGACTCT AGTCTGCAAC TGTGAAGAGA
Mus_musculus_NR_046233         TGTCCCTACC TACTATCCAG CGAAACCACA GCCAAGGGAA CGGGCTTGGC GGAATCAGCG GGGAAAGAAG ACCCTGTTGA GCTTGACTCT AGTCTGGCAC GGTGAAGAGA
Homo_sapiens_U13369            TGTCCCTACC TACTATCCAG CGAAACCACA GCCAAGGGAA CGGGCTTGGC GGAATCAGCG GGGAAAGAAG ACCCTGTTGA GCTTGACTCT AGTCTGGCAC GGTGAAGAGA
Rattus_norvegicus_NR_046239    TGTCCCTACC TACTATCCAG CGAAACCACA GCCAAGGGAA CGGGCTTGGC GGAATCAGCG GGGAAAGAAG ACCCTGTTGA GCTTGACTCT AGTCTGGCAC GGTGAAGAGA
Gallus_gallus_KT445934.2       TGTCCCTATC TACTATCCAG CGAAACCACA GCCAAGGGAA CGGGCTTGGC GGAATCAGCG GGGAAAGAAG ACCCTGTTGA GCTTGACTCT AGTCTGGCGC TGTGAAGAGA

                               1111111111 1111111111 1111111111 1111111111 1111111111 1111111111 1111111111 1111111111 1111111111 1111111111 1111111111
                               4444444444 4444444444 4444444444 4444444444 4444444444 4444444444 4444444444 4444444444 4444444444 4444444444 4444444444
                               6666666666 6666666666 6666666666 6666666666 6666666666 6666666666 6666666667 7777777777 7777777777 7777777777 7777777777
                               3333333334 4444444445 5555555556 6666666667 7777777778 8888888889 9999999990 0000000001 1111111112 2222222223 3333333334
                               1234567890 1234567890 1234567890 1234567890 1234567890 1234567890 1234567890 1234567890 1234567890 1234567890 1234567890
Crocodylus_porosus_EU727191    AACAGACTGA AGGGAAGCAC TTGACAAGAG TGTTTCTGGC TACTTCCAGA CTTGGAGTGG TGCTGTCCCA TTTGACATAC CCTATTGCTC CCTTTGGGCA GCAGAATTCA
Xenopus_laevis_X02995          CATGAGAGGT GTAGGATAAG TGGGAGGCCC CCG-CGCTCG ---------- TCGCAAAGG- ---------- --------GG C-------GC CGCCGGTGAA ATACCACTAC
Mus_musculus_NR_046233         CATGAGAGGT GTAGAATAAG TGGGAGGCCC CCGGCGCCCG GCCCCG-TCC TCGCGTCGG- ----GGTCGG GGCACGCCGG CCTCGCGGGC CGCCGGTGAA ATACCACTAC
Homo_sapiens_U13369            CATGAGAGGT GTAGAATAAG TGGGAGGCCC CCGGCGCCCC CCCGGTGTCC CCGCGAGGGG CCCGGGGCGG GGTCCGC-GG CCCTGCGGGC CGCCGGTGAA ATACCACTAC
Rattus_norvegicus_NR_046239    CATGAGAGGT GTAGAATAAG TGGGAGGCCC CCGGCGCCCC -CCCGT-TCC CCGCGAGGGG TC-GGGGCGG GGTCCGCCGG CCTCGCGGGC CGCCGGTGAA ATACCACTAC
Gallus_gallus_KT445934.2       CATGAGAGGT GTAGAATAAG TGGGAGGCCC C--GCGGTCG CGCGAC---- CCGCGCCGC- ---------- --------GG CCC----GGC CGCCGGTGAA ATACCACTAC

                               1111111111 1111111111 1111111111 1111111111 1111111111 1111111111 1111111111 1111111111 1111111111 1111111111 1111111111
                               4444444444 4444444444 4444444444 4444444444 4444444444 4444444444 4444444444 4444444444 4444444444 4444444444 4444444444
                               7777777777 7777777777 7777777777 7777777777 7777777777 7777777778 8888888888 8888888888 8888888888 8888888888 8888888888
                               4444444445 5555555556 6666666667 7777777778 8888888889 9999999990 0000000001 1111111112 2222222223 3333333334 4444444445
                               1234567890 1234567890 1234567890 1234567890 1234567890 1234567890 1234567890 1234567890 1234567890 1234567890 1234567890
Crocodylus_porosus_EU727191    TCCCAACCTC TTACCCACCC AAGTCTGTGC TTTGTTCTGC TATCCTGCAT TTCTGATCCG CCTTGCTGTC TCCCCCTCTT CGCTGCATTT CTCTCAAGTG GTTCAGTCTC
Xenopus_laevis_X02995          TCTTATCGTT TTTTCACTTA CCCGGTGAGG CGGGGG--CG AGCCCCGAGG GGCTCTCGCT TCTGGACCCA AGCG-----C CGGCCCCCGC G-CCGGGCGC GACCCGCTCC
Mus_musculus_NR_046233         TCTCATCGTT TTTTCACTGA CCCGGTGAGG CGGGGGGGCG AGCCCCGAGG GGCTCTCGCT TCTGGCGCCA AGCGTCCGTC CCGCGCGTGC GGGCGGGCGC GACCCGCTCC
Homo_sapiens_U13369            TCTGATCGTT TTTTCACTGA CCCGGTGAGG CGGGGGGGCG AGCCC-GAGG GGCTCTCGCT TCTGGCGCCA AGCG------ ---CCCGCCC GGCCGGGCGC GACCCGCTCC
Rattus_norvegicus_NR_046239    TCTCATCGTT TTTTCACTGA CCCGGTGAGG CGGGGGGGCG AGCCCCGAGG GGCTCTCGCT TCTGGCGCCG AACG--CGTC C-GCGCGCGC GGGCGGGCGC GACCCGCTCC
Gallus_gallus_KT445934.2       TCTGATCGTT TTTTCACTTA CCCGGTGAGG CGGGGGGGCG AGCCCCGAGG GGCTCTCGCT TCTGGCGCCA AGCG----CC CGGCGCGCGC ---CGGGCGC GACCCGCTCC

                               1111111111 1111111111 1111111111 1111111111 1111111111 1111111111 1111111111 1111111111 1111111111 1111111111 1111111111
                               4444444444 4444444444 4444444444 4444444444 4444444444 4444444444 4444444444 4444444444 4444444444 4444444444 4444444444
                               8888888888 8888888888 8888888888 8888888888 8888888889 9999999999 9999999999 9999999999 9999999999 9999999999 9999999999
                               5555555556 6666666667 7777777778 8888888889 9999999990 0000000001 1111111112 2222222223 3333333334 4444444445 5555555556
                               1234567890 1234567890 1234567890 1234567890 1234567890 1234567890 1234567890 1234567890 1234567890 1234567890 1234567890
Crocodylus_porosus_EU727191    GATATGATTA CCTCCCATGC TTCTGATCAG GGCTCAGGCC CACAGGACAG ATTGCTGGTG CATGTCAGTC ATCACGAGAC TACCAGACTC TTCGGTGGTT GCTATAGTCA
Xenopus_laevis_X02995          GAGGACAGTG GCAGGTGGGG AGTTTGACTG GGGCGGTACA CCTGTCAAAC CGTAACGCAG GTGTCCTAAG GCGAGCTCAG GCGAGCTACA GAAACCTCCC GTGGAGCAGA
Mus_musculus_NR_046233         GGGGACAGTG CCAGGTGGGG AGTTTGACTG GGGCGGTACA CCTGTCAAAC GGTAACGCAG GTGTCCTAAG GCGAGCTCAG GGAGG--ACA GAAACCTCCC GTGGAGCAGA
Homo_sapiens_U13369            GGGGACAGTG CCAGGTGGGG AGTTTGACTG GGGCGGTACA CCTGTCAAAC GGTAACGCAG GTGTCCTAAG GCGAGCTCAG GGAGG--ACA GAAACCTCCC GTGGAGCAGA
Rattus_norvegicus_NR_046239    GGGGACAGTG CCAGGTGGGG AGTTTGACTG GGGCGGTACA CCTGTCAAAC GGTAACGCAG GTGTCCTAAG GCGAGCTCAG GGAGG--ACA GAAACCTCCC GTGGAGCAGA
Gallus_gallus_KT445934.2       GGGGACAGCG TCAGGTGGGG AGTTTGACTG GGGCGGTACA CCTGTCAAAG CGTAACGCAG GTGTCCTAAG GCGAGCTCAG GGAGG--CCA GAAACCTCCC GTGGAGCAGA
```

```
                             1111111111 1111111111 1111111111 1111111111 1111111111 1111111111 1111111111 1111111111 1111111111 1111111111 1111111111
                             4444444444 4444444444 4444444444 4444444445 5555555555 5555555555 5555555555 5555555555 5555555555 5555555555 5555555555
                             9999999999 9999999999 9999999999 9999999990 0000000000 0000000000 0000000000 0000000000 0000000000 0000000000 0000000000
                             6666666667 7777777778 8888888889 9999999990 0000000001 1111111112 2222222223 3333333334 4444444445 5555555556 6666666667
                             1234567890 1234567890 1234567890 1234567890 1234567890 1234567890 1234567890 1234567890 1234567890 1234567890 1234567890
Crocodylus_porosus_EU727191  TGACTGCGTT GCATGGGGCA TGCTGAGCCA CTTAACCGAA CAACACTAAA TAACCCAAAA AAGGTACTCT TCAGGGTTCG GTAAAGAGCC AAATGCCTTG TCATCTAATT
Xenopus_laevis_X02995        AGGGCAAAAG CTCGCTTGAT CTTGATTTTC AGTATGAATA CAGACCGTGA AACGCGGGGC CTCACGATCC TTCTGACTTT TTGGGTTTTA AGCAGGAGGT GTCAGAAAAG
Mus_musculus_NR_046233       AGGGCAAAAG CTCGCTTGAT CTTGATTTTC AGTACGAATA CAGACCGTGA AA-GCGGGGC CTCACGATCC TTCTGACCTT TTGGGTTTTA AGCAGGAGGT GTCAGAAAAG
Homo_sapiens_U13369          AGGGCAAAAG CTCGCTTGAT CTTGATTTTC AGTACGAATA CAGACCGTGA AA-GCGGGGC CTCACGATCC TTCTGACCTT TTGGGTTTTA AGCAGGAGGT GTCAGAAAAG
Rattus_norvegicus_NR_046239  AGGGCAAAAG CTCGCTTGAT CTTGATTTTC AGTACGAATA CAGACCGTGA AA-GCGGGGC CTCACGATCC TTCTGACCTT TTGGGTTTTA AGCAGGAGGT GTCAGAAAAG
Gallus_gallus_KT445934.2     AGGGCAAAAG CTCGCTTGAT CTTGATTTTC AGTACGAATA CAGACCGTGA AA-GCGGGGC CTCACGATCC TTCTGACTTT TTGGGTTTTA AGCAGGAGGT GTCAGAAAAG

                             1111111111 1111111111 1111111111 1111111111 1111111111 1111111111 1111111111 1111111111 1111111111 1111111111 1111111111
                             5555555555 5555555555 5555555555 5555555555 5555555555 5555555555 5555555555 5555555555 5555555555 5555555555 5555555555
                             0000000000 0000000000 0000000001 1111111111 1111111111 1111111111 1111111111 1111111111 1111111111 1111111111 1111111111
                             7777777778 8888888889 9999999990 0000000001 1111111112 2222222223 3333333334 4444444445 5555555556 6666666667 7777777778
                             1234567890 1234567890 1234567890 1234567890 1234567890 1234567890 1234567890 1234567890 1234567890 1234567890 1234567890
Crocodylus_porosus_EU727191  AGTGTTGCAC ATGATAACTG TCTTGTGGTG GCCAAGCGTT CATAGCGATG TCACTTTCTA TCCTTCGATG TCAGCTCTTC CTATCATTGT GAAGCAGAAT TCACCAAGCA
Xenopus_laevis_X02995        TTACCACAGG GATAACTGGC TTGTGGCCGG CCAAGCGTTC ATAGCGACGT CGCTTTTTGA TCCTTCGATG TCGGCTCTTC CTATCATTGT GAAGCAGAAT TCACCAAGCG
Mus_musculus_NR_046233       TTACCACAGG GATAACTGGC TTGTGGC-GG CCAAGCGTTC ATAGCGACGT CGCTTTTTGA TCCTTCGATG TCGGCTCTTC CTATCATTGT GAAGCAGAAT TCACCAAGCG
Homo_sapiens_U13369          TTACCACAGG GATAACTGGC TTGTGGC-GG CCAAGCGTTC ATAGCGACGT CGCTTTTTGA TCCTTCGATG TCGGCTCTTC CTATCATTGT GAAGCAGAAT TCGCCAAGCG
Rattus_norvegicus_NR_046239  TTACCACAGG GATAACTGGC TTGTGGC-GG CCAAGCGTTC ATAGCGACGT CGCTTTTTGA TCCTTCGATG TCGGCTCTTC CTATCATTGT GAAGCAGAAT TCACCAAGCG
Gallus_gallus_KT445934.2     TTACCACAGG GATAACTGGC TTGTGGC-GG CCAAGCGTTC ATAGCGACGT CGCTTTTTGA TCCTTCGATG TCGGCTCTTC CTATCATTGT GAAGCAGAAT TCACCAAGCG

                             1111111111 1111111111 1111111111 1111111111 1111111111 1111111111 1111111111 1111111111 1111111111 1111111111 1111111111
                             5555555555 5555555555 5555555555 5555555555 5555555555 5555555555 5555555555 5555555555 5555555555 5555555555 5555555555
                             1111111111 1111111112 2222222222 2222222222 2222222222 2222222222 2222222222 2222222222 2222222222 2222222222 2222222222
                             8888888889 9999999990 0000000001 1111111112 2222222223 3333333334 4444444445 5555555556 6666666667 7777777778 8888888889
                             1234567890 1234567890 1234567890 1234567890 1234567890 1234567890 1234567890 1234567890 1234567890 1234567890 1234567890
Crocodylus_porosus_EU727191  TTGGATTGTT CCCCCACTAA TAGGGAACGT GAGCTGGGTT TAGACC---- ---------- ---------- ---------- ---------- ---------- ----------
Xenopus_laevis_X02995        TTGGATTGTT CACCCACTAA TAGGGAACGT GAGCTGGGTT TAGACCGTCG TGAGACAGGT TAGTTTTACC CTACTGATGA TGTGTTGTCG CAATAGTAAT CCTGCTCAGT
Mus_musculus_NR_046233       TTGGATTGTT CACCCACTAA TAGGGAACGT GAGCTGGGTT TAGACCGTCG TGAGACAGGT TAGTTTTACC CTACTGATGA TGTGTTGTTG CCATGGTAAT CCTGCTCAGT
Homo_sapiens_U13369          TTGGATTGTT CACCCACTAA TAGGGAACGT GAGCTGGGTT TAGACCGTCG TGAGACAGGT TAGTTTTACC CTACTGATGA TGTGTTGTTG CCATGGTAAT CCTGCTCAGT
Rattus_norvegicus_NR_046239  TTGGATTGTT CACCCACTAA TAGGGAACGT GAGCTGGGTT TAGACCGTCG TGAGACAGGT TAGTTTTACC CTACTGATGA TGTGTTGTTG CCATGGTAAT CCTGCTCAGT
Gallus_gallus_KT445934.2     TTGGATTGTT CACCCACTAA TAGGGAACGT GAGCTGGGTT TAGACCGTCG TGAGACAGGT TAGTTTTACC CTACTGATGA TGTGTTGTTG CGCTAGTAAT CCTGCTCAGT

                             1111111111 1111111111 1111111111 1111111111 1111111111 1111111111 1111111111 1111111111 1111111111 1111111111 1111111111
                             5555555555 5555555555 5555555555 5555555555 5555555555 5555555555 5555555555 5555555555 5555555555 5555555555 5555555555
                             2222222223 3333333333 3333333333 3333333333 3333333333 3333333333 3333333333 3333333333 3333333333 3333333333 3333333334
                             9999999990 0000000001 1111111112 2222222223 3333333334 4444444445 5555555556 6666666667 7777777778 8888888889 9999999990
                             1234567890 1234567890 1234567890 1234567890 1234567890 1234567890 1234567890 1234567890 1234567890 1234567890 1234567890
Crocodylus_porosus_EU727191  ---------- ---------- ---------- ---------- ---------- ---------- ---------- ---------- ---------- ---------- ----------
Xenopus_laevis_X02995        ACGAGAGGAA CCGCAGGTTC AGACATTTGG TGTATGTGCT TGGCTGAGGA GCCAATGGGG CGAAGCTACC ATCTGTGGGA TTATGACTGA ACGCCTCTAA GTCAGAATCC
Mus_musculus_NR_046233       ACGAGAGGAA CCGCAGGTTC AGACATTTGG TGTATGTGCT TGGCTGAGGA GCCAATGGGG CGAAGCTACC ATCTGTGGGA TTATGACTGA ACGCCTCTAA GTCAGAATCC
Homo_sapiens_U13369          ACGAGAGGAA CCGCAGGTTC AGACATTTGG TGTATGTGCT TGGCTGAGGA GCCAATGGGG CGAAGCTACC ATCTGTGGGA TTATGACTGA ACGCCTCTAA GTCAGAATCC
Rattus_norvegicus_NR_046239  ACGAGAGGAA CCGCAGGTTC AGACATTTGG TGTATGTGCT TGGCTGAGGA GCCAATGGGG CGAAGCTACC ATCTGTGGGA TTATGACTGA ACGCCTCTAA GTCAGAATCC
Gallus_gallus_KT445934.2     ACGAGAGGAA CCGCAGGTTC AGACATTTGG TGTATGTGCT TGGCTGAGGA GCCACTGGAG CGAGGCTACC ATCTGTGGGA TTATGACTGA ACGCCTCTAA GTCAGAATCC
```

```
                              1111111111 1111111111 1111111111 1111111111 1111111111 1111111111 1111111111 1111111111 1111111111 1111111111 1111111111
                              5555555555 5555555555 5555555555 5555555555 5555555555 5555555555 5555555555 5555555555 5555555555 5555555555 5555555555
                              4444444444 4444444444 4444444444 4444444444 4444444444 4444444444 4444444444 4444444444 4444444444 4444444445 5555555555
                              0000000001 1111111112 2222222223 3333333334 4444444445 5555555556 6666666667 7777777778 8888888889 9999999990 0000000001
                              1234567890 1234567890 1234567890 1234567890 1234567890 1234567890 1234567890 1234567890 1234567890 1234567890 1234567890
Crocodylus_porosus_EU727191   ---------- ---------- ---------- ---------- ---------- ---------- ---------- ---------- ---------- ---------- ----------
Xenopus_laevis_X02995         CCCCTAAACG TGACGATACC GCAGCGCCGC -GGAGCCTCG GTCGGCCTCG GATTAGCCGG CGCCCCCCCC G--------- ---GG----- ---------- ----------
Mus_musculus_NR_046233        GCCC-AAGCG GAACGATACG GCAGCGCCGA AGGAGCCTCG GTTGGCCCCG GAT-AGCCGG GTCCCCGTCC GTCCCGCTCG GCGGG--GTC CCCGCGTCG- ----CCCCGC
Homo_sapiens_U13369           CGCC-CAGGC GAACGATACG GCAGCGCCGC -GGAGCCTCG GTTGGCCTCG GAT-AGCCGG TCCCCCGCCT GTCCCCGCCG GCGGGCCGCC CCCCCCTCCA CGCGCCCCGC
Rattus_norvegicus_NR_046239   GCCC-AAGCG GAACGATACG GCAGCGCCGA AGGAGCCTCG GTTGGCCCCG GAT-AGCCGG CTCCCCGTCC GTCCCCGTCC GGCGG--GTC CCCGCCTCGT CGC-CCCCCC
Gallus_gallus_KT445934.2      CCCCTAAACG TAGCGATACC GCAGCGCCGA -GGCGCCTCG GTGGGCTCGC GAT-AGCCGG CCGCCGCCCC CCTC------ --GGG----- ---------- ----------

                              1111111111 1111111111 1111111111 1111111111 1111111111 1111111111 1111111111 1111111111 1111111111 1111111111 1111111111
                              5555555555 5555555555 5555555555 5555555555 5555555555 5555555555 5555555555 5555555555 5555555555 5555555555 5555555555
                              5555555555 5555555555 5555555555 5555555555 5555555555 5555555555 5555555555 5555555555 5555555556 6666666666 6666666666
                              1111111112 2222222223 3333333334 4444444445 5555555556 6666666667 7777777778 8888888889 9999999990 0000000001 1111111112
                              1234567890 1234567890 1234567890 1234567890 1234567890 1234567890 1234567890 1234567890 1234567890 1234567890 1234567890
Crocodylus_porosus_EU727191   ---------- ---------- ---------- ---------- ---------- ---------- ---------- ---------- ---------- ---------- ----------
Xenopus_laevis_X02995         ---------- ---------- ----GGGCG- ---------- --CCGGCGGG CAGAGCCGCT CGCCTCGGGA C-CGGAGCGC GGACG-AAAG GGGGCCGCC- TCTCTCCCGG
Mus_musculus_NR_046233        GGCGGCGCGG GGTCTCCCCC CGCCGGGCGT CGGGACCGGG GTCCGGTGCG GAGAGCCGTT CGTCTTGGGA AACGGGGTGC GGCCGGAAAG GGGGCCGCCC TCTCGCCCGT
Homo_sapiens_U13369           CGCGGGAGGG CGCGTGCCCC -GCCGCGCGC CGGGACCGGG GTCCGGTGCG GAGTGCCCTT CGTCCTGGGA AACGGGGCGC GGCCGGAAAG GCGGCCGCCC CCTCGCCCGT
Rattus_norvegicus_NR_046239   GGGTGCGGGG CGGGTCCCCC CGCCGGGCGT CGGGACCGGG GTCCGGTGCG GAGAGCCATT CGTCCCGGGA AACGGGGTGC GGCCGGAAAG GGGGCCGCCC TCTCGCCCGT
Gallus_gallus_KT445934.2      ---------- ---------- ---CGGGCGG ---------- --TCGGTGCG GAGCGCCGCT CGTGGTCGGG ACCGGAGCGC GGACAGATGT GGCGCCGCC- TCTCCCCCGC

                              1111111111 1111111111 1111111111 1111111111 1111111111 1111111111 1111111111 1111111111 1111111111 1111111111 1111111111
                              5555555555 5555555555 5555555555 5555555555 5555555555 5555555555 5555555555 5555555555 5555555555 5555555555 5555555555
                              6666666666 6666666666 6666666666 6666666666 6666666666 6666666666 6666666666 6666666667 7777777777 7777777777 7777777777
                              2222222223 3333333334 4444444445 5555555556 6666666667 7777777778 8888888889 9999999990 0000000001 1111111112 2222222223
                              1234567890 1234567890 1234567890 1234567890 1234567890 1234567890 1234567890 1234567890 1234567890 1234567890 1234567890
Crocodylus_porosus_EU727191   ---------- ---------- ---------- ---------- ---------- ---------- ---------- ---------- ---------- ---------- ----------
Xenopus_laevis_X02995         AGCGC--ACC GCACGTTCGT GGGGAACCTG GTGCTAAATC ATTCGTAGAC GACCTGATTC TGGGTCAGGG TTTCGTGCGT AGCAGAGCAG CTACCTCGCT GCGATCTATT
Mus_musculus_NR_046233        CACGTTGAAC GCACGTTCGT GTGGAACCTG GCGCTAAACC ATTCGTAGAC GACCTGCTTC TGGGTCGGGG TTTCGTACGT AGCAGAGCAG CTCCCTCGCT GCGATCTATT
Homo_sapiens_U13369           CACGC--ACC GCACGTTCGT GGGGAACCTG GCGCTAAACC ATTCGTAGAC GACCTGCTTC TGGGTCGGGG TTTCGTACGT AGCAGAGCAG CTCCCTCGCT GCGATCTATT
Rattus_norvegicus_NR_046239   CACGCTTAAC GCACGTTCGT GTGGAACTTG GCGCTAAACC ATTCGTAGAC GACCTGCTTC TGGGTCGGGG TTTCGTACGT AGCAGAGCAG CTCCCTCGCT GCGATCTATT
Gallus_gallus_KT445934.2      CGCGT--ACC GCATGTTCGT GGGGAACCCG GTGCTAAATC ATTCGTAGAC GACCTGATTC TGGGTCGGGG TTTCGTACGT AGCAGAGCAG CTCCCTCGCT GCGATCTATT

                              1111111111 1111111111 1111111111 1111111111 1111111111 1111111111 1111111111 1111111111 1111111111 1111111111 1111111111
                              5555555555 5555555555 5555555555 5555555555 5555555555 5555555555 5555555555 5555555555 5555555555 5555555555 5555555555
                              7777777777 7777777777 7777777777 7777777777 7777777777 7777777777 7777777778 8888888888 8888888888 8888888888 8888888888
                              3333333334 4444444445 5555555556 6666666667 7777777778 8888888889 9999999990 0000000001 1111111112 2222222223 3333333334
                              1234567890 1234567890 1234567890 1234567890 1234567890 1234567890 1234567890 1234567890 1234567890 1234567890 1234567890
Crocodylus_porosus_EU727191   ---------- ---------- ---------- ---------- ---------- ---------- ---------- ---------- ---------- ---------- ----------
Xenopus_laevis_X02995         GAAAGTCATC CCTTGGC-CA AGCTTTTGTC ---GGAAGGA GCAGGCCGGA AGGGCGCCCC CGCCGCCGGC CGGCGCGACG TCCCGTCCGC CCTCCCCGGC CTCCCGCCGC
Mus_musculus_NR_046233        GAAAGTCAGC CCTCGACACA AGGGTTTGTC ---TCTGCGG GCTTTCCCGT CGCACGCCCG CTCGCTCGCA CGCGACCGTG TCGCCGCCCG GGCGTCACGG GGGCGGTCGC
Homo_sapiens_U13369           GAAAGTCAGC CCTCGACACA AGGGTTTGTC ---CGCGCGC GCGTGCGTGC GGGGGGCCCG GCGGGCGTGC GCGTTCGGCG CCGTCCGTCC TTCCGTTCGT CTTCCTCCCT
Rattus_norvegicus_NR_046239   GAAAGTCAGC CCTCGACACA AGGGTTTGT- ---------- ---------- ---------- ---------- ---------- ---------- ---------- ----------
Gallus_gallus_KT445934.2      GAGAGTCAGC CCTCGACACA AGCTTTTGTC ---GGAGCGC GGAGCGCGCG CGCGCGCGCG CGTGGCGGCG CCCCGGCGCG GGGCCGGGTC CGGCGGGCCA GTCGGTCGGC
```

```
                               1111111111 1111111111 1111111111 1111111111 1111111111 1111111111 1111111111 1111111111 1111111111 1111111111 1111111111
                               5555555555 5555555555 5555555555 5555555555 5555555555 5555555555 5555555555 5555555555 5555555555 5555555555 5555555555
                               8888888888 8888888888 8888888888 8888888888 8888888888 8888888889 9999999999 9999999999 9999999999 9999999999 9999999999
                               4444444445 5555555556 6666666667 7777777778 8888888889 9999999990 0000000001 1111111112 2222222223 3333333334 4444444445
                               1234567890 1234567890 1234567890 1234567890 1234567890 1234567890 1234567890 1234567890 1234567890 1234567890 1234567890
Crocodylus_porosus_EU727191    ---------- ---------- ---------- ---------- ---------- ---------- ---------- ---------- ---------- ---------- ----------
Xenopus_laevis_X02995          GCTCCCCTTT CCGCGGGGGG GAGAGAGCGG CGGCGGGGCG GGGGGGAGGC GGCCGGGCGA CCGCCGCCGG CCCGGGGACC GTCCCGTCCC CCGGCCTCTC CCGCAGGGAG
Mus_musculus_NR_046233         CTCGGCCCCC GCGCGGTTGC CCGAACGACC GTGTGGTGGT TGGGGGGGGG ATCGTCTTCT CCTCCGTCTC CCGAGGACGG TTCGTTTCTC TTTCCCCTTC CGTCGCTCTC
Homo_sapiens_U13369            CCCGGCCTCT CCCGCCGACC GCGGCGTGGT GGTGGGGTGG GGGGGAGGGC GCGCGACCCC GGTCGGCCGC CCCGCTTCTT CGGTTCCCGC CTCCTCCCCG TTCACGCCGG
Rattus_norvegicus_NR_046239    ---------- ---------- ---------- ---------- ---------- ---------- ---------- ---------- ---------- ---------- ----------
Gallus_gallus_KT445934.2       TCCCCGCGCC GCTCCGTTTG TTCCTGGGTT CGTTCGTTCG TTCGTTCCTT CCTTCCCCGG CCCCGCGCCG GCGCCGGCGC GGGGTTGGAA AGAGGGGGAG AGGGGCGGGG

                               1111111111 1111111111 1111111111 1111111111 1111111111 1111111111 1111111111 1111111111 1111111111 1111111111 1111111111
                               5555555555 5555555555 5555555555 5555555555 5555555556 6666666666 6666666666 6666666666 6666666666 6666666666 6666666666
                               9999999999 9999999999 9999999999 9999999999 9999999990 0000000000 0000000000 0000000000 0000000000 0000000000 0000000000
                               5555555556 6666666667 7777777778 8888888889 9999999990 0000000001 1111111112 2222222223 3333333334 4444444445 5555555556
                               1234567890 1234567890 1234567890 1234567890 1234567890 1234567890 1234567890 1234567890 1234567890 1234567890 1234567890
Crocodylus_porosus_EU727191    ---------- ---------- ---------- ---------- ---------- ---------- ---------- ---------- ---------- ---------- ----------
Xenopus_laevis_X02995          GGGGCCGAAG GCCCGGCCCC CGCACCCCCC CTCCCTCCCG CC-------- ---------- ---------- ---------- ---------- ---------- ----------
Mus_musculus_NR_046233         CTTGGGTGTG GGAGCCTCGT GCCGTCGCGA CCGCGGCCTG CCGTCGCCTG CCGCCGCAGC CCCTTGCCCT CCGGCCTTGG CCAAGCCGGA GGGCGGAGGA GGGGGATCGG
Homo_sapiens_U13369            GGCGGCTCGT CCGCTCCGGG CCGGGACGGG GTCCGGGGAG CGTGGTTTGG GAGCCGCGGA GGCGCCGCGC CGAGCCGGGC CCCGTGGCCC GCCGGTCCCC GTCCCGGGGG
Rattus_norvegicus_NR_046239    ---------- ---------- ---------- ---------- ---------- ---------- ---------- ---------- ---------- ---------- ----------
Gallus_gallus_KT445934.2       GGCGCGGCCG GCCCCCTTCC CCGTTTCCGT CCCCGCGGCG CGTGCCGTGG GACGGGCTCC CTCCGTTTTA CCCGAGCCCG GGGGTTGACC TGGCGGCCGG GCGGCCGGGC

                               1111111111 1111111111 1111111111 1111111111 1111111111 1111111111 1111111111 1111111111 1111111111 1111111111 1111111111
                               6666666666 6666666666 6666666666 6666666666 6666666666 6666666666 6666666666 6666666666 6666666666 6666666666 6666666666
                               0000000000 0000000000 0000000000 0000000001 1111111111 1111111111 1111111111 1111111111 1111111111 1111111111 1111111111
                               6666666667 7777777778 8888888889 9999999990 0000000001 1111111112 2222222223 3333333334 4444444445 5555555556 6666666667
                               1234567890 1234567890 1234567890 1234567890 1234567890 1234567890 1234567890 1234567890 1234567890 1234567890 1234567890
Crocodylus_porosus_EU727191    ---------- ---------- ---------- ---------- ---------- ---------- ---------- ---------- ---------- ---------- ----------
Xenopus_laevis_X02995          ---------- ---------- ---------- ---------- ---------- ---------- ---------- ---------- ---------- ---------- ----------
Mus_musculus_NR_046233         CGGCGGCGGC GACCGCGGCG CGGTGACGCA CGGTGGGATC CCCATCCTCG GCGCGTCCGT CGGGGACGGC CGGTTGGAGG GGCGGGAGGG GTTTTTCCCG TGAACGCCGC
Homo_sapiens_U13369            TTGGCCGCGC GGCGCGGTGG GGGGCCACCC GGGGTCCCGG CCCTCGCG-- ---------- ---------- ---------- ---------- ---------- ----------
Rattus_norvegicus_NR_046239    ---------- ---------- ---------- ---------- ---------- ---------- ---------- ---------- ---------- ---------- ----------
Gallus_gallus_KT445934.2       TAGGGGGCGC TCCGCGTCCC CCTTCGGGGG GTTGACCTGT CGGGCG---- ---------- ---------- ---------- ---------- ---------- ----------

                               1111111111 1111111111 1111111111 1111111111 1111111111 1111111111 1111111111 1111111111 1111111111 1111111111 1111111111
                               6666666666 6666666666 6666666666 6666666666 6666666666 6666666666 6666666666 6666666666 6666666666 6666666666 6666666666
                               1111111111 1111111111 1111111112 2222222222 2222222222 2222222222 2222222222 2222222222 2222222222 2222222222 2222222222
                               7777777778 8888888889 9999999990 0000000001 1111111112 2222222223 3333333334 4444444445 5555555556 6666666667 7777777778
                               1234567890 1234567890 1234567890 1234567890 1234567890 1234567890 1234567890 1234567890 1234567890 1234567890 1234567890
Crocodylus_porosus_EU727191    ---------- ---------- ---------- ---------- ---------- ---------- ---------- ---------- ---------- ---------- ----------
Xenopus_laevis_X02995          ---------- ---------- ---------- ---------- ---------- ---------- ---------- ---------- ---------- ---------- ----------
Mus_musculus_NR_046233         GTTCGGCGCC AGGCCTCTGG CGGCCGGGGG GGCGCTCTCT CCGCCCGAGC ATCCCCACTC CCGCCCCTCC TCTTCGCGCG CCGCGGCGGC GACGTGCGTA CGAGGGGAGG
Homo_sapiens_U13369            ---------- ---------- ---------- ---------- ---------- ---------- ---------- ---------- ---------- ---------- ----------
Rattus_norvegicus_NR_046239    ---------- ---------- ---------- ---------- ---------- ---------- ---------- ---------- ---------- ---------- ----------
Gallus_gallus_KT445934.2       ---------- ---------- ---------- ---------- ---------- ---------- ---------- ---------- ---------- ---------- ----------
```

```
                              1111111111 1111111111 1111111111 1111111111 1111111111 1111111111 1111111111 1111111111 1111111111 1111111111 1111111111
                              6666666666 6666666666 6666666666 6666666666 6666666666 6666666666 6666666666 6666666666 6666666666 6666666666 6666666666
                              2222222222 2222222223 3333333333 3333333333 3333333333 3333333333 3333333333 3333333333 3333333333 3333333333 3333333333
                              8888888889 9999999990 0000000001 1111111112 2222222223 3333333334 4444444445 5555555556 6666666667 7777777778 8888888889
                              1234567890 1234567890 1234567890 1234567890 1234567890 1234567890 1234567890 1234567890 1234567890 1234567890 1234567890
Crocodylus_porosus_EU727191   ---------- ---------- ---------- ---------- ---------- ---------- ---------- ---------- ---------- ---------- ----------
Xenopus_laevis_X02995         ---------- ---------- ---------- ---------- ---------- ---------- ---------- ---------- ---------- ---------- ----------
Mus_musculus_NR_046233        ATGTCGCGGT GTGGAGGCGG AGAGGGTCCG GCGCGGCGCC TCTTCCA--- ---------- ---------- ---------- ---------- ---------- ----------
Homo_sapiens_U13369           ---------- ---------- ---------- ---------- ---------- CGTCCTTCCT CCTCGCTCCT CCGCACGGGT CGACCGACGA ACCGCGGGTG GCGGGCGGCG
Rattus_norvegicus_NR_046239   ---------- ---------- ---------- ---------- ---------- ---------- ---------- ---------- ---------- ---------- ----------
Gallus_gallus_KT445934.2      ---------- ---------- ---------- ---------- ---------- TTTTTTTTTT ATTTTTTTCT CCCTAGGCGG GTCCGGGGGT AGCCCTGTCG GCCGCCCGGC

                              1111111111 1111111111 1111111111 1111111111 1111111111 1111111111 1111111111 1
                              6666666666 6666666666 6666666666 6666666666 6666666666 6666666666 6666666666 6
                              3333333334 4444444444 4444444444 4444444444 4444444444 4444444444 4444444444 4
                              9999999990 0000000001 1111111112 2222222223 3333333334 4444444445 5555555556 6
                              1234567890 1234567890 1234567890 1234567890 1234567890 1234567890 1234567890 1
Crocodylus_porosus_EU727191   ---------- ---------- ---------- ---------- ---------- ---------- ---------- -
Xenopus_laevis_X02995         ---------- ---------- ---------- ---------- ---------- ---------- ---------- -
Mus_musculus_NR_046233        ---------- ---------- ---------- ---------- ---------- ---------- ---------- -
Homo_sapiens_U13369           GGCGGCGAGC CCCACGGGCG TCCCCGCACC CGGCCGACCT CCGCTCGCGA CCTCTCCTCG GTCGGGCCTC C
Rattus_norvegicus_NR_046239   ---------- ---------- ---------- ---------- ---------- ---------- ---------- -
Gallus_gallus_KT445934.2      CCGGCCCAGC ACGCCCCCCC GCCGGCAAGT GGCTGCGGTG CCGAGGTGGC GGGTAGACCT GGCGGCCGGC A
```
