# Supplementary material for: Chicken rRNA Gene Cluster Structure
Source: PLoS One. 2016 Jun 14;11(6):e0157464. doi: 10.1371/journal.pone.0157464 (PMC4907446; doi:10.1371/journal.pone.0157464)
Supplement: S3 Table — (PDF) [file pone.0157464.s012.pdf]

S3 Table

De novo and re-assembled rRNA gene cluster sequence alignment

```
                                                                                                                                         1 1111111111
                                      1 1111111112 2222222223 3333333334 4444444445 5555555556 6666666667 7777777778 8888888889 9999999990 0000000001
                             1234567890 1234567890 1234567890 1234567890 1234567890 1234567890 1234567890 1234567890 1234567890 1234567890 1234567890
De_novo_assembly_KT445934    GGTAGTCGGC GCCTATGGGG CTAGAACGTT TTTTTCGGAT GCCTTATATG TTCGTCTGTA GGAGCGAGTG AGGACTCGGC TCCGGTAGTG GCGGTGAGCG GGCGCTCGCG
Reference_assembly           GGTAGTCGGC GCCTATGGGA CTAGAACGTT TTTTTCGGAT GCCTTATATG TTCGTCTGTA GGAGCGAGTG AGGACTCGGC TCCGGTAGTG GCGGTGAGCG GGCGCTCGCG

                             1111111111 1111111111 1111111111 1111111111 1111111111 1111111111 1111111111 1111111111 1111111112 2222222222 2222222222
                             1111111112 2222222223 3333333334 4444444445 5555555556 6666666667 7777777778 8888888889 9999999990 0000000001 1111111112
                             1234567890 1234567890 1234567890 1234567890 1234567890 1234567890 1234567890 1234567890 1234567890 1234567890 1234567890
De_novo_assembly_KT445934    AGCAGGGTTG ACCGGCCGGC CGCCTAGAGA GGGGATCGGC GGGGGCGGCG GCGGCTTTCT CGGGCATCGG TTCGTTCGAT CGGTCCGGTC GCTTCGGTTT GTCCGTCGCT
Reference_assembly           AGCAGGGTTG ACCGGCCGGC CGCCTAGAGA GGGGATCGGC GGCGGCGGCG GCGGCTTTCT CGGGCATCGG TTCGTTCGAT CGGTCCGGTC GCTTCGGTTT GTCCGTCGCT

                             2222222222 2222222222 2222222222 2222222222 2222222222 2222222222 2222222222 2222222223 3333333333 3333333333 3333333333
                             2222222223 3333333334 4444444445 5555555556 6666666667 7777777778 8888888889 9999999990 0000000001 1111111112 2222222223
                             1234567890 1234567890 1234567890 1234567890 1234567890 1234567890 1234567890 1234567890 1234567890 1234567890 1234567890
De_novo_assembly_KT445934    CCTCATCCCG CAGCTCTGTC CTGGGCTAAG GCGGTTTTGC AGGCGAGCAG CGAAAAAAAG CCGGAGAAGG CGAGAGAGAG GCAAGAAGCA AGCCGGCTCC CGCGCCGCCA
Reference_assembly           CCTCATCCCG CAGCTCTGTC CTGGGCTAAG GCGGTTTTGC AGGCGAGCAG CGAAAAAAAG CCGGAGAAGG CGAGAGAGAG GCAAGAAGCA AGCCGGCTCC CGCGCCGCCA

                             3333333333 3333333333 3333333333 3333333333 3333333333 3333333333 3333333334 4444444444 4444444444 4444444444 4444444444
                             3333333334 4444444445 5555555556 6666666667 7777777778 8888888889 9999999990 0000000001 1111111112 2222222223 3333333334
                             1234567890 1234567890 1234567890 1234567890 1234567890 1234567890 1234567890 1234567890 1234567890 1234567890 1234567890
De_novo_assembly_KT445934    GGGCGAAGGC GAGAGAGAGA GGGAGACGAG AAGGGCACGG GCCGGTCTGC CGGCACCCGA ACGTAGGATG GCCGGGGGCG TCCCCGGCGG GTCCCGCCGC GATGGAAGAG
Reference_assembly           GGGCGAAGGC GAGAGAGAGA GGGAGACGAG AAGGGCACGG GCCGGTCTGC CGGCACCCGA ACGTAGGATG GCCGGGGGCG TCCCCGGCGG GTCCCGCCGC GATGGAAGAG

                             4444444444 4444444444 4444444444 4444444444 4444444444 4444444445 5555555555 5555555555 5555555555 5555555555 5555555555
                             4444444445 5555555556 6666666667 7777777778 8888888889 9999999990 0000000001 1111111112 2222222223 3333333334 4444444445
                             1234567890 1234567890 1234567890 1234567890 1234567890 1234567890 1234567890 1234567890 1234567890 1234567890 1234567890
De_novo_assembly_KT445934    GGGGACCCGG AGGTCGTAGG TCGTGGCGGC GTCGCCTCGT CCTCCTTTCG CACCGCATTC TCACCCGCAC GCGGGAGCCC CGGCCGATTC GTGGCGCTCC TCGGGCGCGT
Reference_assembly           GGGGACCCGG AGGTCGTAGG TCGTGGCGGC GTCGCCTCGT CCTCCTTTCG CACCGCATTC TCACCCGCAC GCGGGAGCCC CGGCCGATTC GTGGCGCTCC TCGGGCGCGT

                             5555555555 5555555555 5555555555 5555555555 5555555556 6666666666 6666666666 6666666666 6666666666 6666666666 6666666666
                             5555555556 6666666667 7777777778 8888888889 9999999990 0000000001 1111111112 2222222223 3333333334 4444444445 5555555556
                             1234567890 1234567890 1234567890 1234567890 1234567890 1234567890 1234567890 1234567890 1234567890 1234567890 1234567890
De_novo_assembly_KT445934    CGGGGAGGCT TCCCGGCGGG CCGGCTCTAT CCCGCTCCCC GGCTCGTTCG GGGTGGCGTG GGGCGGGCCG GTGTTCAGGC ACGGGCGAGC ACCTCTCGTC GGACGTTGCC
Reference_assembly           CGGGGAGGCT TCCCGGCGGG CCGGCTCTAT CCCGCTCCCC GGCTCGTTCG GGGTGGCGTG GGGCGGGCCG GTGTTCAGGC ACGGGCGAGC ACCTCTCGTC GGACGTTGCC

                             6666666666 6666666666 6666666666 6666666667 7777777777 7777777777 7777777777 7777777777 7777777777 7777777777 7777777777
                             6666666667 7777777778 8888888889 9999999990 0000000001 1111111112 2222222223 3333333334 4444444445 5555555556 6666666667
                             1234567890 1234567890 1234567890 1234567890 1234567890 1234567890 1234567890 1234567890 1234567890 1234567890 1234567890
De_novo_assembly_KT445934    CACGCACACC CACCTGCACG TGCGCGTGCG GTCTTTCCGC CGCGCCTGGG GGAAGGGCTC GCGCCTTCTC CCTCCTTTCT TTCTCCTCCC CCCCACCCCC TTTCTCCCAC
Reference_assembly           CACGCACACC CACCTGCACG TGCGCGTGCG GTCTTTCCGC CGCGCCTGGG GGAAGGGCTC GCGCCTTCTC CCTCCTTTCT TTCTCCTCCC CCCCACCCCC TTTCTCCCAC

                             7777777777 7777777777 7777777778 8888888888 8888888888 8888888888 8888888888 8888888888 8888888888 8888888888 8888888888
                             7777777778 8888888889 9999999990 0000000001 1111111112 2222222223 3333333334 4444444445 5555555556 6666666667 7777777778
                             1234567890 1234567890 1234567890 1234567890 1234567890 1234567890 1234567890 1234567890 1234567890 1234567890 1234567890
De_novo_assembly_KT445934    CGATCGATGA GGCCACTCGG GTCGCGTCGG AGAGGGCCCC CGGCGGGCCG GCGCTCTGCG CTCCCTGTCC CAGGGAAGCC GCGGCGGCGT CCGGTGTTCA GGCACGGGCG
Reference_assembly           CGATCGATGA GGCCACTCGG GTCGCGTCGG AGAGGGCCCC CGGCGGGCCG GCGCTCTGCG CTCCCTGTCC CAGGGAAGCC GCGGCGGCGT CCGGTGTTCA GGCACGGGCG
```

```
                                 8888888888 8888888889 9999999999 9999999999 9999999999 9999999999 9999999999 9999999999 9999999999 9999999999 9999999999
                                 8888888889 9999999990 0000000001 1111111112 2222222223 3333333334 4444444445 5555555556 6666666667 7777777778 8888888889
                                 1234567890 1234567890 1234567890 1234567890 1234567890 1234567890 1234567890 1234567890 1234567890 1234567890 1234567890
De_novo_assembly_KT445934        GCCTCCTCTC CAGTTCGCTT CCCGTCGTTC GCGAGGTGAG GCGCTCGCCC GCTTGGGCCG AGGGCGGCGG CGGCGGCGGC TTCGGGGCGC GTGGCCTCGC CGTGCCGACT
Reference_assembly               GCCTCCTCTC CAGTTCGCTT CCCGTCGTTC GCGAGGTGAG GCGCTCGCCC GCTTGGGCCG AGGGCGGCGG CGGCGGCGGC TTCGGGGCGC GTGGCCTCGC CGTGCCGACT

                                          1 1111111111 1111111111 1111111111 1111111111 1111111111 1111111111 1111111111 1111111111 1111111111 1111111111
                                 9999999990 0000000000 0000000000 0000000000 0000000000 0000000000 0000000000 0000000000 0000000000 0000000000 0000000001
                                 9999999990 0000000001 1111111112 2222222223 3333333334 4444444445 5555555556 6666666667 7777777778 8888888889 9999999990
                                 1234567890 1234567890 1234567890 1234567890 1234567890 1234567890 1234567890 1234567890 1234567890 1234567890 1234567890
De_novo_assembly_KT445934        CGTCTGTCCG CCCGCCCTGT CGGTGCCCCA GGGCTCGCCC GACCGAATCC AGCTGTGTGA CGGCCGAGCG GCCCCGCGAG CCGCAGGCGT ACCCATTTCG TTGTGAGCGA
Reference_assembly               CGTCTGTCCG CCCGCCCTGT CGGTGCCCCA GGGCTCGCCC GACCGAATCC AGCTGTGTGA CGGCCGAGCG GCCCCGCGAG CCGCAGGCGT ACCTATTTCG TTGTGAGCGA

                                 1111111111 1111111111 1111111111 1111111111 1111111111 1111111111 1111111111 1111111111 1111111111 1111111111 1111111111
                                 1111111111 1111111111 1111111111 1111111111 1111111111 1111111111 1111111111 1111111111 1111111111 1111111112 2222222222
                                 0000000001 1111111112 2222222223 3333333334 4444444445 5555555556 6666666667 7777777778 8888888889 9999999990 0000000001
                                 1234567890 1234567890 1234567890 1234567890 1234567890 1234567890 1234567890 1234567890 1234567890 1234567890 1234567890
De_novo_assembly_KT445934        GGCGTCGGCG CTGCCCTCGT TTCGGGGCCC GGCGAGTGCC GGCCGCGAGC AGCAAGCCGG CGGGGTGGCA ACCGAGGGAA ACCGCGGGGA ACCGAGGCGA AGCGAGCAGC
Reference_assembly               GGCGTCGGCG CTGCCCTCGT TTCGGGGCCC GGCGAGTGCC GGCCGCGAGC AGCAAGCCGG CGGGGTGGCA ACCGAGGGAA ACCGCGGGGA ACCGAGGCGA AGCGAGCAGC

                                 1111111111 1111111111 1111111111 1111111111 1111111111 1111111111 1111111111 1111111111 1111111111 1111111111 1111111111
                                 2222222222 2222222222 2222222222 2222222222 2222222222 2222222222 2222222222 2222222222 2222222223 3333333333 3333333333
                                 1111111112 2222222223 3333333334 4444444445 5555555556 6666666667 7777777778 8888888889 9999999990 0000000001 1111111112
                                 1234567890 1234567890 1234567890 1234567890 1234567890 1234567890 1234567890 1234567890 1234567890 1234567890 1234567890
De_novo_assembly_KT445934        AGCAGAAGAA GGAACGAGAA GACAACGGGG GGCTGCGCCC GGCCGAGCGG GCGAGCCCGG AGCAGCGCGG CGCGTCCCGC TCCGGATCCG TCGGGGTGTG GGGGCCGGGG
Reference_assembly               AGCAGAAGAA GGAACGAGAA GACAACGGGG GGCTGCGCCC GGCCGAGCGG GCGAGCCCGG AGCAGCGCGG CGCGTCCCGC TCCGGATCCG TCGGGGTGTG GGGGCCGGGG

                                 1111111111 1111111111 1111111111 1111111111 1111111111 1111111111 1111111111 1111111111 1111111111 1111111111 1111111111
                                 3333333333 3333333333 3333333333 3333333333 3333333333 3333333333 3333333333 3333333334 4444444444 4444444444 4444444444
                                 2222222223 3333333334 4444444445 5555555556 6666666667 7777777778 8888888889 9999999990 0000000001 1111111112 2222222223
                                 1234567890 1234567890 1234567890 1234567890 1234567890 1234567890 1234567890 1234567890 1234567890 1234567890 1234567890
De_novo_assembly_KT445934        GCGTCCGCCG GCCTCTCCTC CGCCTTCGGG CCGCCGCAGC CCGTGTCGGT TTTCCTGCCG CGTCCCCGCC CGCTGCGGAG CGTGCCGCCC CGGGAAAGGG TCTCCGATCG
Reference_assembly               GCGTCCGCCG GCCTCTCCTC CGCCTTCGGG CCGCCGCAGC CCGTGTCGGT TTTCCTGCCG CGTCCCCGCC CGCTGCGGAG CGTGCCGCCC CGGGAAAGGG TCTCCGATCG

                                 1111111111 1111111111 1111111111 1111111111 1111111111 1111111111 1111111111 1111111111 1111111111 1111111111 1111111111
                                 4444444444 4444444444 4444444444 4444444444 4444444444 4444444444 4444444445 5555555555 5555555555 5555555555 5555555555
                                 3333333334 4444444445 5555555556 6666666667 7777777778 8888888889 9999999990 0000000001 1111111112 2222222223 3333333334
                                 1234567890 1234567890 1234567890 1234567890 1234567890 1234567890 1234567890 1234567890 1234567890 1234567890 1234567890
De_novo_assembly_KT445934        TGGGGTCGCG CCCGTCTCGA GGTCGCGTTC TCCTCTAGCA CGTCCGTCTC CGGCGGCGGG GCTTCGTTTC CCCGTCCGCT TCTCCGCCGG TCCCGGAGGG CGGGTCAGCC
Reference_assembly               TGGGGTCGCG CCCGTCTCGA GGTCGCGTTC TCCTCTAGCA CGTCCGTCTC CGGCGGTGGG GCTTCGTTTC CCCGTCCGCT TCTCCGCCGG TCCCGGAGGG CGGGTCAGCC

                                 1111111111 1111111111 1111111111 1111111111 1111111111 1111111111 1111111111 1111111111 1111111111 1111111111 1111111111
                                 5555555555 5555555555 5555555555 5555555555 5555555555 5555555556 6666666666 6666666666 6666666666 6666666666 6666666666
                                 4444444445 5555555556 6666666667 7777777778 8888888889 9999999990 0000000001 1111111112 2222222223 3333333334 4444444445
                                 1234567890 1234567890 1234567890 1234567890 1234567890 1234567890 1234567890 1234567890 1234567890 1234567890 1234567890
De_novo_assembly_KT445934        CCGGCCGGCC GTGCGGCGCG AGCGCGAGTC CGGCTCCCGC GGGGGGGGCC CGGAGCGTGC CGCCGAAAGC AGCTGCGCAG CGGTCCCCGC TCCTTCCCCG CGGGGGGGAG
Reference_assembly               CCGGCCGGCC GTGCGGCGCG AGCGCGAGTC CGGCTCCCGC GGGGGGGGCC CGGAGCGTGC CGCCGAAAGC AGCTGCGCAG CGGTCCCCGC TCCTTCCCCG CGGGGGGGAG

                                 1111111111 1111111111 1111111111 1111111111 1111111111 1111111111 1111111111 1111111111 1111111111 1111111111 1111111111
                                 6666666666 6666666666 6666666666 6666666666 6666666667 7777777777 7777777777 7777777777 7777777777 7777777777 7777777777
                                 5555555556 6666666667 7777777778 8888888889 9999999990 0000000001 1111111112 2222222223 3333333334 4444444445 5555555556
                                 1234567890 1234567890 1234567890 1234567890 1234567890 1234567890 1234567890 1234567890 1234567890 1234567890 1234567890
De_novo_assembly_KT445934        GTCGGCGGGG CCGCCCCGGG GATCGGGCGC GCCTCTCCGT CGTGGTCGGC GAGCGAGCGA GCGAGCGAGG GAACGACGGA GGGCCGCCCG CCCCGCCGAG AGGCGTTCGC
Reference_assembly               GTCGGCGGGG CCGCCCCGGG GATCGGGCGC GCCTCTCCGT CGTGGTCGGC GAGCGAGCGA GCGAGCGAGG GAACGACGGA GGGCCGCCCG CCCCGCCGAG AGGCGTTCGC
```

```
                          1111111111 1111111111 1111111111 1111111111 1111111111 1111111111 1111111111 1111111111 1111111111 1111111111 1111111111
                          7777777777 7777777777 7777777777 7777777778 8888888888 8888888888 8888888888 8888888888 8888888888 8888888888 8888888888
                          6666666667 7777777778 8888888889 9999999990 0000000001 1111111112 2222222223 3333333334 4444444445 5555555556 6666666667
                          1234567890 1234567890 1234567890 1234567890 1234567890 1234567890 1234567890 1234567890 1234567890 1234567890 1234567890
De_novo_assembly_KT445934 CCCGGCGGCC GCCGCCGTCG ACCCGGCAAG GGCCAGACGG GAAAGCCGAG CGAGCAGGCG AGAGAGAGAG AGAGGGAAGG AGCGAGAGCG GTCGGCGGCG GGCCGGGCCC
Reference_assembly        CCCGGCGGCC GCCGCCGTCG ACCCGGCAAG GGCCAGACGG GAAAGCCGAG CGAGCAGGCG AGAGAGAGAG AGAGGGAAGG AGCGAGAGCG GTCGGCGGCG GGCCGGGCCC

                          1111111111 1111111111 1111111111 1111111111 1111111111 1111111111 1111111111 1111111111 1111111111 1111111111 1111111111
                          8888888888 8888888888 8888888889 9999999999 9999999999 9999999999 9999999999 9999999999 9999999999 9999999999 9999999999
                          7777777778 8888888889 9999999990 0000000001 1111111112 2222222223 3333333334 4444444445 5555555556 6666666667 7777777778
                          1234567890 1234567890 1234567890 1234567890 1234567890 1234567890 1234567890 1234567890 1234567890 1234567890 1234567890
De_novo_assembly_KT445934 GTCGGGTCGT GCCCCGTGGC GCGGCTACCT GGTTGATCCT GCCAGTAGCA TATGCTTGTC TCAAAGATTA AGCCATGCAT GTCTAAGTAC ACACGGGCGG TACAGTGAAA
Reference_assembly        GTCGGGTCGT GCCCCGTGGC GCGGCTACCT GGTTGATCCT GCCAGTAGCA TATGCTTGTC TCAAAGATTA AGCCATGCAT GTCTAAGTAC ACACGGGCGG TACAGTGAAA

                          1111111111 1111111112 2222222222 2222222222 2222222222 2222222222 2222222222 2222222222 2222222222 2222222222 2222222222
                          9999999999 9999999990 0000000000 0000000000 0000000000 0000000000 0000000000 0000000000 0000000000 0000000000 0000000000
                          8888888889 9999999990 0000000001 1111111112 2222222223 3333333334 4444444445 5555555556 6666666667 7777777778 8888888889
                          1234567890 1234567890 1234567890 1234567890 1234567890 1234567890 1234567890 1234567890 1234567890 1234567890 1234567890
De_novo_assembly_KT445934 CTGCGAATGG CTCATTAAAT CAGTTATGGT TCCTTTGGTC GCTCCCCTCC CGTTACTTGG ATAACTGTGG TAATTCTAGA GCTAATACAT GCCGACGAGC GCCGACCTCC
Reference_assembly        CTGCGAATGG CTCATTAAAT CAGTTATGGT TCCTTTGGTC GCTCCCCTCC CGTTACTTGG ATAACTGTGG TAATTCTAGA GCTAATACAT GCCGACGAGC GCCGACCTCC

                          2222222222 2222222222 2222222222 2222222222 2222222222 2222222222 2222222222 2222222222 2222222222 2222222222 2222222222
                          0000000001 1111111111 1111111111 1111111111 1111111111 1111111111 1111111111 1111111111 1111111111 1111111111 1111111112
                          9999999990 0000000001 1111111112 2222222223 3333333334 4444444445 5555555556 6666666667 7777777778 8888888889 9999999990
                          1234567890 1234567890 1234567890 1234567890 1234567890 1234567890 1234567890 1234567890 1234567890 1234567890 1234567890
De_novo_assembly_KT445934 GGGGACGCGT GCATTTATCA GACCAAAACC AACCCGGGCT CGCCCGGCGG CTTTGGTGAC TCTAGATAAC CTCGAGCCGA TCGCACGCCC CCGTGGCGGC GACGACCCAT
Reference_assembly        GGGGACGCGT GCATTTATCA GACCAAAACC AACCCGGGCT CGCCCGGCGG CTTTGGTGAC TCTAGATAAC CTCGAGCCGA TCGCACGCCC CCGTGGCGGC GACGACCCAT

                          2222222222 2222222222 2222222222 2222222222 2222222222 2222222222 2222222222 2222222222 2222222222 2222222222 2222222222
                          2222222222 2222222222 2222222222 2222222222 2222222222 2222222222 2222222222 2222222222 2222222222 2222222223 3333333333
                          0000000001 1111111112 2222222223 3333333334 4444444445 5555555556 6666666667 7777777778 8888888889 9999999990 0000000001
                          1234567890 1234567890 1234567890 1234567890 1234567890 1234567890 1234567890 1234567890 1234567890 1234567890 1234567890
De_novo_assembly_KT445934 TCGAATGTCT GCCCTATCAA CTTTCGATGG TACTGTCTGT GCCTACCATG GTGACCACGG GTAACGGGGA ATCAGGGTTC GATTCCGGAG AGGGAGCCTG AGAAACGGCT
Reference_assembly        TCGAATGTCT GCCCTATCAA CTTTCGATGG TACTGTCTGT GCCTACCATG GTGACCACGG GTAACGGGGA ATCAGGGTTC GATTCCGGAG AGGGAGCCTG AGAAACGGCT

                          2222222222 2222222222 2222222222 2222222222 2222222222 2222222222 2222222222 2222222222 2222222222 2222222222 2222222222
                          3333333333 3333333333 3333333333 3333333333 3333333333 3333333333 3333333333 3333333333 3333333334 4444444444 4444444444
                          1111111112 2222222223 3333333334 4444444445 5555555556 6666666667 7777777778 8888888889 9999999990 0000000001 1111111112
                          1234567890 1234567890 1234567890 1234567890 1234567890 1234567890 1234567890 1234567890 1234567890 1234567890 1234567890
De_novo_assembly_KT445934 ACCACATCCA AGGAAGGCAG CAGGCGCGCA AATTACCCAC TCCCGACCCG GGGAGGTAGT GACGAAAAAT AACAATACAG GACTCTTTCG AGGCCCTGTA ATTGGAATGA
Reference_assembly        ACCACATCCA AGGAAGGCAG CAGGCGCGCA AATTACCCAC TCCCGACCCG GGGAGGTAGT GACGAAAAAT AACAATACAG GACTCTTTCG AGGCCCTGTA ATTGGAATGA

                          2222222222 2222222222 2222222222 2222222222 2222222222 2222222222 2222222222 2222222222 2222222222 2222222222 2222222222
                          4444444444 4444444444 4444444444 4444444444 4444444444 4444444444 4444444444 4444444445 5555555555 5555555555 5555555555
                          2222222223 3333333334 4444444445 5555555556 6666666667 7777777778 8888888889 9999999990 0000000001 1111111112 2222222223
                          1234567890 1234567890 1234567890 1234567890 1234567890 1234567890 1234567890 1234567890 1234567890 1234567890 1234567890
De_novo_assembly_KT445934 GTCCACTTTA AATCCTTTAA CGAGGATCCA TTGGAGGGCA AGTCTGGTGC CAGCAGCCGC GGTAATTCCA GCTCCAATAG CGTATATTAA AGTTGCTGCA GTTAAAAAGC
Reference_assembly        GTCCACTTTA AATCCTTTAA CGAGGATCCA TTGGAGGGCA AGTCTGGTGC CAGCAGCCGC GGTAATTCCA GCTCCAATAG CGTATATTAA AGTTGCTGCA GTTAAAAAGC
```

```
                           2222222222 2222222222 2222222222 2222222222 2222222222 2222222222 2222222222 2222222222 2222222222 2222222222 2222222222
                           5555555555 5555555555 5555555555 5555555555 5555555555 5555555555 5555555556 6666666666 6666666666 6666666666 6666666666
                           3333333334 4444444445 5555555556 6666666667 7777777778 8888888889 9999999990 0000000001 1111111112 2222222223 3333333334
                           1234567890 1234567890 1234567890 1234567890 1234567890 1234567890 1234567890 1234567890 1234567890 1234567890 1234567890
De_novo_assembly_KT445934  TCGTAGTTGG ATCTTGGGAT CGAGCTGGCG GTCCGCCGCG AGGCGAGCTA CCGCCTGTCC CAGCCCCTGT CTCTCGGCGC CCCCTCGATG CTCTTAACTG AGTGTCCCGC
Reference_assembly         TCGTAGTTGG ATCTTGGGAT CGAGCTGGCG GTCCGCCGCG AGGCGAGCTA CCGCCTGTCC CAGCCCCTGT CTCTCGGCGC CCCCTCGATG CTCTTAACTG AGTGTCCCGC

                           2222222222 2222222222 2222222222 2222222222 2222222222 2222222222 2222222222 2222222222 2222222222 2222222222 2222222222
                           6666666666 6666666666 6666666666 6666666666 6666666666 6666666667 7777777777 7777777777 7777777777 7777777777 7777777777
                           4444444445 5555555556 6666666667 7777777778 8888888889 9999999990 0000000001 1111111112 2222222223 3333333334 4444444445
                           1234567890 1234567890 1234567890 1234567890 1234567890 1234567890 1234567890 1234567890 1234567890 1234567890 1234567890
De_novo_assembly_KT445934  GGGGCCCGAA GCGTTTACTT TGAAAAAATT AGAGTGTTCA AAGCAGGCTG GCCGCCGGAA TACTCCAGCT AGGAATAATG GAATAGGACT CCGGTTCTAT TTTGTTGGTT
Reference_assembly         GGGGCCCGAA GCGTTTACTT TGAAAAAATT AGAGTGTTCA AAGCAGGCTG GCCGCCGGAA TACTCCAGCT AGGAATAATG GAATAGGACT CCGGTTCTAT TTTGTTGGTT

                           2222222222 2222222222 2222222222 2222222222 2222222222 2222222222 2222222222 2222222222 2222222222 2222222222 2222222222
                           7777777777 7777777777 7777777777 7777777777 7777777778 8888888888 8888888888 8888888888 8888888888 8888888888 8888888888
                           5555555556 6666666667 7777777778 8888888889 9999999990 0000000001 1111111112 2222222223 3333333334 4444444445 5555555556
                           1234567890 1234567890 1234567890 1234567890 1234567890 1234567890 1234567890 1234567890 1234567890 1234567890 1234567890
De_novo_assembly_KT445934  TTCGGAAACG GGGCCATGAT TAAGAGGGAC GGCCGGGGGC ATTCGTATTG TGCCGCTAGA GGTGAAATTC TTGGACCGGC GCAAGACGAA CTAAAGCGAA AGCATTTGCC
Reference_assembly         TTCGGAAACG GGGCCATGAT TAAGAGGGAC GGCCGGGGGC ATTCGTATTG TGCCGCTAGA GGTGAAATTC TTGGACCGGC GCAAGACGAA CTAAAGCGAA AGCATTTGCC

                           2222222222 2222222222 2222222222 2222222222 2222222222 2222222222 2222222222 2222222222 2222222222 2222222222 2222222222
                           8888888888 8888888888 8888888888 8888888889 9999999999 9999999999 9999999999 9999999999 9999999999 9999999999 9999999999
                           6666666667 7777777778 8888888889 9999999990 0000000001 1111111112 2222222223 3333333334 4444444445 5555555556 6666666667
                           1234567890 1234567890 1234567890 1234567890 1234567890 1234567890 1234567890 1234567890 1234567890 1234567890 1234567890
De_novo_assembly_KT445934  AAGAATGTTT TCATTAATCA AGAACGAAAG TCGGAGGTTC GAAGACGATC AGATACCGTC GTAGTTCCGA CCATAAACGA TGCCGACTCG CGATCCGGCG GCGTTATTCC
Reference_assembly         AAGAATGTTT TCATTAATCA AGAACGAAAG TCGGAGGTTC GAAGACGATC AGATACCGTC GTAGTTCCGA CCATAAACGA TGCCGACTCG CGATCCGGCG GCGTTATTCC

                           2222222222 2222222222 2222222223 3333333333 3333333333 3333333333 3333333333 3333333333 3333333333 3333333333 3333333333
                           9999999999 9999999999 9999999990 0000000000 0000000000 0000000000 0000000000 0000000000 0000000000 0000000000 0000000000
                           7777777778 8888888889 9999999990 0000000001 1111111112 2222222223 3333333334 4444444445 5555555556 6666666667 7777777778
                           1234567890 1234567890 1234567890 1234567890 1234567890 1234567890 1234567890 1234567890 1234567890 1234567890 1234567890
De_novo_assembly_KT445934  CATGACCCGC CGGGCAGCTC CCGGGAAACC CAAGTCTTTG GGTTCCGGGG GGAGTATGGT TGCAAAGCTG AAACTTAAAG GAATTGACGG AAGGGCACCA CCAGGAGTGG
Reference_assembly         CATGACCCGC CGGGCAGCTC CCGGGAAACC CAAGTCTTTG GGTTCCGGGG GGAGTATGGT TGCAAAGCTG AAACTTAAAG GAATTGACGG AAGGGCACCA CCAGGAGTGG

                           3333333333 3333333333 3333333333 3333333333 3333333333 3333333333 3333333333 3333333333 3333333333 3333333333 3333333333
                           0000000000 0000000001 1111111111 1111111111 1111111111 1111111111 1111111111 1111111111 1111111111 1111111111 1111111111
                           8888888889 9999999990 0000000001 1111111112 2222222223 3333333334 4444444445 5555555556 6666666667 7777777778 8888888889
                           1234567890 1234567890 1234567890 1234567890 1234567890 1234567890 1234567890 1234567890 1234567890 1234567890 1234567890
De_novo_assembly_KT445934  AGCCTGCGGC TTAATTTGAC TCAACACGGG AAACCTCACC CGGCCCGGAC ACGGACAGGA TTGACAGATT GAGAGCTCTT TCTCGATTCC GTGGGTGGTG GTGCATGGCC
Reference_assembly         AGCCTGCGGC TTAATTTGAC TCAACACGGG AAACCTCACC CGGCCCGGAC ACGGACAGGA TTGACAGATT GAGAGCTCTT TCTCGATTCC GTGGGTGGTG GTGCATGGCC

                           3333333333 3333333333 3333333333 3333333333 3333333333 3333333333 3333333333 3333333333 3333333333 3333333333 3333333333
                           1111111112 2222222222 2222222222 2222222222 2222222222 2222222222 2222222222 2222222222 2222222222 2222222222 2222222223
                           9999999990 0000000001 1111111112 2222222223 3333333334 4444444445 5555555556 6666666667 7777777778 8888888889 9999999990
                           1234567890 1234567890 1234567890 1234567890 1234567890 1234567890 1234567890 1234567890 1234567890 1234567890 1234567890
De_novo_assembly_KT445934  GTTCTTAGTT GGTGGAGCGA TTTGTCTGGT TAATTCCGAT AACGAACGAG ACTCTGGCAT GCTAACTAGT TACGCGACCC CCGAGCGGTC GGCGTCCAAC TTCTTAGAGG
Reference_assembly         GTTCTTAGTT GGTGGAGCGA TTTGTCTGGT TAATTCCGAT AACGAACGAG ACTCTGGCAT GCTAACTAGT TACGCGACCC CCGAGCGGTC GGCGTCCAAC TTCTTAGAGG
```

```
                              3333333333 3333333333 3333333333 3333333333 3333333333 3333333333 3333333333 3333333333 3333333333 3333333333 3333333333
                              3333333333 3333333333 3333333333 3333333333 3333333333 3333333333 3333333333 3333333333 3333333333 3333333334 4444444444
                              0000000001 1111111112 2222222223 3333333334 4444444445 5555555556 6666666667 7777777778 8888888889 9999999990 0000000001
                              1234567890 1234567890 1234567890 1234567890 1234567890 1234567890 1234567890 1234567890 1234567890 1234567890 1234567890
De_novo_assembly_KT445934     GACAAGTGGC GTTCAGCCAC CCGAGATTGA GCAATAACAG GTCTGTGATG CCCTTAGATG TCCGGGGCTG CACGCGCGCT ACACTGACTG GCTCAGCTTG TGTCTACCCT
Reference_assembly            GACAAGTGGC GTTCAGCCAC CCGAGATTGA GCAATAACAG GTCTGTGATG CCCTTAGATG TCCGGGGCTG CACGCGCGCT ACACTGACTG GCTCAGCTTG TGTCTACCCT

                              3333333333 3333333333 3333333333 3333333333 3333333333 3333333333 3333333333 3333333333 3333333333 3333333333 3333333333
                              4444444444 4444444444 4444444444 4444444444 4444444444 4444444444 4444444444 4444444444 4444444445 5555555555 5555555555
                              1111111112 2222222223 3333333334 4444444445 5555555556 6666666667 7777777778 8888888889 9999999990 0000000001 1111111112
                              1234567890 1234567890 1234567890 1234567890 1234567890 1234567890 1234567890 1234567890 1234567890 1234567890 1234567890
De_novo_assembly_KT445934     ACGCCGGCAG GCGCGGGTAA CCCGTTGAAC CCCATTCGTG ATGGGGATCG GGGATTGCAA TTATTCCCCA TGAACGAGGA ATTCCCAGTA AGTGCGGGTC ATAAGCTCGC
Reference_assembly            ACGCCGGCAG GCGCGGGTAA CCCGTTGAAC CCCATTCGTG ATGGGGATCG GGGATTGCAA TTATTCCCCA TGAACGAGGA ATTCCCAGTA AGTGCGGGTC ATAAGCTCGC

                              3333333333 3333333333 3333333333 3333333333 3333333333 3333333333 3333333333 3333333333 3333333333 3333333333 3333333333
                              5555555555 5555555555 5555555555 5555555555 5555555555 5555555555 5555555555 5555555556 6666666666 6666666666 6666666666
                              2222222223 3333333334 4444444445 5555555556 6666666667 7777777778 8888888889 9999999990 0000000001 1111111112 2222222223
                              1234567890 1234567890 1234567890 1234567890 1234567890 1234567890 1234567890 1234567890 1234567890 1234567890 1234567890
De_novo_assembly_KT445934     GTTGATTAAG TCCCTGCCCT TTGTACACAC CGCCCGTCGC TACTACCGAT TGGATGGTTT AGTGAGGTCC TCGGATCGGC CCCGGCGGGG TCGGCCACGG CCCTGCCGGA
Reference_assembly            GTTGATTAAG TCCCTGCCCT TTGTACACAC CGCCCGTCGC TACTACCGAT TGGATGGTTT AGTGAGGTCC TCGGATCGGC CCCGGCGGGG TCGGCCACGG CCCTGCCGGA

                              3333333333 3333333333 3333333333 3333333333 3333333333 3333333333 3333333333 3333333333 3333333333 3333333333 3333333333
                              6666666666 6666666666 6666666666 6666666666 6666666666 6666666666 6666666667 7777777777 7777777777 7777777777 7777777777
                              3333333334 4444444445 5555555556 6666666667 7777777778 8888888889 9999999990 0000000001 1111111112 2222222223 3333333334
                              1234567890 1234567890 1234567890 1234567890 1234567890 1234567890 1234567890 1234567890 1234567890 1234567890 1234567890
De_novo_assembly_KT445934     GCGTCGAGAA GACGGTCGAA CTTGACTATC TAGAGGAAGT AAAAGTCGTA ACAAGGTTTC CGTAGGTGAA CCTGCGGAAG GATCATTACC GGGGCCGAGG CCGGGCGTCC
Reference_assembly            GCGTCGAGAA GACGGTCGAA CTTGACTATC TAGAGGAAGT AAAAGTCGTA ACAAGGTTTC CGTAGGTGAA CCTGCGGAAG GATCATTACC GGGGCCGAGG CCGGGCGTCC

                              3333333333 3333333333 3333333333 3333333333 3333333333 3333333333 3333333333 3333333333 3333333333 3333333333 3333333333
                              7777777777 7777777777 7777777777 7777777777 7777777777 7777777778 8888888888 8888888888 8888888888 8888888888 8888888888
                              4444444445 5555555556 6666666667 7777777778 8888888889 9999999990 0000000001 1111111112 2222222223 3333333334 4444444445
                              1234567890 1234567890 1234567890 1234567890 1234567890 1234567890 1234567890 1234567890 1234567890 1234567890 1234567890
De_novo_assembly_KT445934     GGCCGAGCCG TGGCACGAGC GCGCGCGGGC GCGCAGCCTT CCCTTCCCTT CCCCGAGCCC GCTCCGCGCG GAGCGCGGCT CCTCTCCCCC GGTCGAAACG GGGAAAGAAA
Reference_assembly            GGCCGAGCCG TGGCACGAGC GCGCGCGGGC GCGCAGCCTT CCCTTCCCTT CCCCGAGCCC GCTCCGCGCG GAGCGCGGCT CCTCTCCCCC GGTCGAAACG GGGAAAGAAA

                              3333333333 3333333333 3333333333 3333333333 3333333333 3333333333 3333333333 3333333333 3333333333 3333333333 3333333333
                              8888888888 8888888888 8888888888 8888888888 8888888889 9999999999 9999999999 9999999999 9999999999 9999999999 9999999999
                              5555555556 6666666667 7777777778 8888888889 9999999990 0000000001 1111111112 2222222223 3333333334 4444444445 5555555556
                              1234567890 1234567890 1234567890 1234567890 1234567890 1234567890 1234567890 1234567890 1234567890 1234567890 1234567890
De_novo_assembly_KT445934     AAAAAAACAC CGCAAGTCGC TCCGCGCGCC TGCCGGCGAG AGAGAAGGGA GACGAGGGCG CGGAGCGCAG CTCCGGGGGG GGAGGCGCGT GTGGGGCGC- ---GCGCTCC
Reference_assembly            AAAAAAACAC CGCAAGTCGC TCCGCGCGCC TGCCGGCGAG AGAGAAGGGA GACGAGGGCG CGGAGCGCAG CTCCGGGGGG GGAGGCGCGT GTGGGGCGCT CCGGCGCTCC

                              3333333333 3333333333 3333333333 3333333334 4444444444 4444444444 4444444444 4444444444 4444444444 4444444444 4444444444
                              9999999999 9999999999 9999999999 9999999990 0000000000 0000000000 0000000000 0000000000 0000000000 0000000000 0000000000
                              6666666667 7777777778 8888888889 9999999990 0000000001 1111111112 2222222223 3333333334 4444444445 5555555556 6666666667
                              1234567890 1234567890 1234567890 1234567890 1234567890 1234567890 1234567890 1234567890 1234567890 1234567890 1234567890
De_novo_assembly_KT445934     GGCGCGTCTC TCCCCCCCCG GCGCCGGTCC GCCGTCGGTC CGCACGCCGC GGGTCCGGTC CGTCCGGTCG CCTCGCCGGC GCGCGCCCGC GCGCGCGCGT CCCGCGGGCC
Reference_assembly            GGCGCGTCTC TCCCCCCC-G GCGCCGGTCC GCCGTCGGTC CGCACGCCGC GGGTCCGGTC CGTCCGGTCG CCTCGCCGGC GCGCGCCCGC GCGCGCGCGT CCCGCGGGCC
```

```
                              4444444444 4444444444 4444444444 4444444444 4444444444 4444444444 4444444444 4444444444 4444444444 4444444444 4444444444
                              0000000000 0000000000 0000000001 1111111111 1111111111 1111111111 1111111111 1111111111 1111111111 1111111111 1111111111
                              7777777778 8888888889 9999999990 0000000001 1111111112 2222222223 3333333334 4444444445 5555555556 6666666667 7777777778
                              1234567890 1234567890 1234567890 1234567890 1234567890 1234567890 1234567890 1234567890 1234567890 1234567890 1234567890
De_novo_assembly_KT445934     TCGCCCGGGT CGCCGCGCTC CGGAGCGTCC CGCGGCCGAG TCCCGCTCCG ACCGCGGGGT CGGGGTCGGG AGGTGGCGGC GGTGCGGAGG GTGGAAGGAC GGCTCCCCGC
Reference_assembly            TCGCCCGGGT CGCCGCGCTC CGGAGCGTCC CGCGGCCGAG TCCCGCTCCG ACCGCGGGGT CGGGGTCGGG AGGTGGCGGC GGTGCGGAGG GTGGAAGGAC GGCTCCCCGC

                              4444444444 4444444444 4444444444 4444444444 4444444444 4444444444 4444444444 4444444444 4444444444 4444444444 4444444444
                              1111111111 1111111112 2222222222 2222222222 2222222222 2222222222 2222222222 2222222222 2222222222 2222222222 2222222222
                              8888888889 9999999990 0000000001 1111111112 2222222223 3333333334 4444444445 5555555556 6666666667 7777777778 8888888889
                              1234567890 1234567890 1234567890 1234567890 1234567890 1234567890 1234567890 1234567890 1234567890 1234567890 1234567890
De_novo_assembly_KT445934     TTCGTCGCTC GGCCGGAAAC TCGCCACCCG CCCCCGCCGC TGTCGACGCC GGCACCCCGA GTCCGCTCGG AGGGAAGCCG CGCGGGCGGC CGCGCGCGGG GGAGGCGGCG
Reference_assembly            TTCGTCGCTC GGCCGGAAAC TCGCCACCGG CCCCCGCCGC TGTCGACGCC GGCACCCCGA GTCCGCTCGG AGGGAAGCCG CGCGGGCGGC CGCGCGCGGG GGAGGCGGCG

                              4444444444 4444444444 4444444444 4444444444 4444444444 4444444444 4444444444 4444444444 4444444444 4444444444 4444444444
                              2222222223 3333333333 3333333333 3333333333 3333333333 3333333333 3333333333 3333333333 3333333333 3333333333 3333333334
                              9999999990 0000000001 1111111112 2222222223 3333333334 4444444445 5555555556 6666666667 7777777778 8888888889 9999999990
                              1234567890 1234567890 1234567890 1234567890 1234567890 1234567890 1234567890 1234567890 1234567890 1234567890 1234567890
De_novo_assembly_KT445934     GGCGGCGGGT CCGAGCGCGG GGCGCGGGAA GTCGGCCGCT TCCCCCGGCC TCACCCCCCA CCCCCTTCGC CCGGCCCGTC GCGGGGACGG GGCCGGGTCG CGGGCGGCTG
Reference_assembly            GGCGGCGGGT CCGAGCGCGG GGCGCGGGAA GTCGGCCGCT TCCCCCGGCC TCACCCCCCA CCCCCTTCGC CCGGCCCGTC GCGGGGACGG GGCCGGGTCG CGGGCGGCTG

                              4444444444 4444444444 4444444444 4444444444 4444444444 4444444444 4444444444 4444444444 4444444444 4444444444 4444444444
                              4444444444 4444444444 4444444444 4444444444 4444444444 4444444444 4444444444 4444444444 4444444444 4444444445 5555555555
                              0000000001 1111111112 2222222223 3333333334 4444444445 5555555556 6666666667 7777777778 8888888889 9999999990 0000000001
                              1234567890 1234567890 1234567890 1234567890 1234567890 1234567890 1234567890 1234567890 1234567890 1234567890 1234567890
De_novo_assembly_KT445934     CGGAGCCGGC CGACTCCGGG CGAGCGCCGG AGGGACGCGC GCGCCGCGTA CGCGCGGCAG GCGCGAGGTG CCCCGGGCGG CTTCGGTCCC GCGCGGGCGG TCCGAGCCTC
Reference_assembly            CGGAGCCGGC CGACTCCGGG CGAGCGCCGG AGGGACGCGC GCGCCGCGTA CGCGCGGCAG GCGCGAGGTG CCCCGGGCGG CTTCGGTCCC GCGCGGGCGG TCCGAGCCTC

                              4444444444 4444444444 4444444444 4444444444 4444444444 4444444444 4444444444 4444444444 4444444444 4444444444 4444444444
                              5555555555 5555555555 5555555555 5555555555 5555555555 5555555555 5555555555 5555555555 5555555556 6666666666 6666666666
                              1111111112 2222222223 3333333334 4444444445 5555555556 6666666667 7777777778 8888888889 9999999990 0000000001 1111111112
                              1234567890 1234567890 1234567890 1234567890 1234567890 1234567890 1234567890 1234567890 1234567890 1234567890 1234567890
De_novo_assembly_KT445934     GCGGCTCCTC CCGGGTGCAG CTGCCGCCCG GCGCCGGGTT GCCGAGGGAA ACCCCGGGCC CCGGGAGGAA CGCGAGGTGG TGGCGGCGGA CGTCGGGCGC GCCCCCGCGG
Reference_assembly            GCGGCTCCTC CCGGGTGCAG CTGCCGCCCG GCGCCGGGTT GCCGAGGGAA ACCCCGGGCC CCGGGAGGAA CGCGAGGTGG TGGCGGCGGA CGTCGGGCGC GCCCCCGCGG

                              4444444444 4444444444 4444444444 4444444444 4444444444 4444444444 4444444444 4444444444 4444444444 4444444444 4444444444
                              6666666666 6666666666 6666666666 6666666666 6666666666 6666666666 6666666666 6666666667 7777777777 7777777777 7777777777
                              2222222223 3333333334 4444444445 5555555556 6666666667 7777777778 8888888889 9999999990 0000000001 1111111112 2222222223
                              1234567890 1234567890 1234567890 1234567890 1234567890 1234567890 1234567890 1234567890 1234567890 1234567890 1234567890
De_novo_assembly_KT445934     GCGGACGCTC CCCCGAGGGG CGCCGGGGCC GGCTGGCGGG TGCCGGGTCT CCCCTCGGCG CCCCGTCCCG CCCCGCCGAG CGGGGCGGGC GGGGGAGGCA CCCCCGCGGG
Reference_assembly            GCGGACGCTC CCCCGAGGGG CGCCGGGGCC GGCTGGCGGG TGCCGGGTCT CCCCTCGGCG CCCCGTCCCG CCCCGCCGAG CGGGGCGGGC GGGGGAGGCA CCCCCGCGGG

                              4444444444 4444444444 4444444444 4444444444 4444444444 4444444444 4444444444 4444444444 4444444444 4444444444 4444444444
                              7777777777 7777777777 7777777777 7777777777 7777777777 7777777777 7777777778 8888888888 8888888888 8888888888 8888888888
                              3333333334 4444444445 5555555556 6666666667 7777777778 8888888889 9999999990 0000000001 1111111112 2222222223 3333333334
                              1234567890 1234567890 1234567890 1234567890 1234567890 1234567890 1234567890 1234567890 1234567890 1234567890 1234567890
De_novo_assembly_KT445934     GCCTTCGGGT CGTTTCCCTC ACCCCAGGGC CAGGTACCTA GCGTCCGCGC CTCCGCGCGT CCGGGGGGCG GGGAGGAAGG AGCGCGGCGC CGGTCCCGAG CGGGCCGCGT
Reference_assembly            GCCTTCGGGT CGTTTCCCTC ACCCCAGGGC CAGGTACCTA GCGTCCGCGC CTCCGCGCGT CCGGGGGGCG GGGAGGAAGG AGCGCGGCGC CGGTCCCGAG CGGGCCGCGT
```

```
                              4444444444 4444444444 4444444444 4444444444 4444444444 4444444444 4444444444 4444444444 4444444444 4444444444 4444444444
                              8888888888 8888888888 8888888888 8888888888 8888888888 8888888889 9999999999 9999999999 9999999999 9999999999 9999999999
                              4444444445 5555555556 6666666667 7777777778 8888888889 9999999990 0000000001 1111111112 2222222223 3333333334 4444444445
                              1234567890 1234567890 1234567890 1234567890 1234567890 1234567890 1234567890 1234567890 1234567890 1234567890 1234567890
De_novo_assembly_KT445934     CGCCCACACC CCCCTCCTCC CCCCGGGCCG CGGAGCCGGG CGGAGGTTTA AAGACTCGGG CGGCCCGCGG CGCGCGCCGC GAGGTCGGGG GCCGGGGGCG GTCTTCTGCC
Reference_assembly            CGCCCACACC CCCCTCCTCC CCCCGGGCCG CGGAGCCGGG CGGAGGTTTA AAGACTCGGG CGGCCCGCGG CGCGCGCCGC GAGGTCGGGG GCCGGGGGCG GTCTTCTGCC

                              4444444444 4444444444 4444444444 4444444444 4444444445 5555555555 5555555555 5555555555 5555555555 5555555555 5555555555
                              9999999999 9999999999 9999999999 9999999999 9999999990 0000000000 0000000000 0000000000 0000000000 0000000000 0000000000
                              5555555556 6666666667 7777777778 8888888889 9999999990 0000000001 1111111112 2222222223 3333333334 4444444445 5555555556
                              1234567890 1234567890 1234567890 1234567890 1234567890 1234567890 1234567890 1234567890 1234567890 1234567890 1234567890
De_novo_assembly_KT445934     CGCCGGCGGG ACGCCGGGAT GGAAGAGAGG ACTCCGGGCG GGGCGCGGCG GCGCGCCCCG CCGGCCCTCT CCCTCCCGAG CCCGCCGGCG GCGTCGGCCG TCGCCGCGCC
Reference_assembly            CGCCGGCGGG ACGCCGGGAT GGAAGAGAGG AGTCCGGGCG GGGCGCGGCG GCGCGCCCCG CCGGCCCTCT CCCTCCCGAG CCCGCCGGCG GCGTCGGCCG TCGCCGCGCC

                              5555555555 5555555555 5555555555 5555555555 5555555555 5555555555 5555555555 5555555555 5555555555 5555555555 5555555555
                              0000000000 0000000000 0000000000 0000000001 1111111111 1111111111 1111111111 1111111111 1111111111 1111111111 1111111111
                              6666666667 7777777778 8888888889 9999999990 0000000001 1111111112 2222222223 3333333334 4444444445 5555555556 6666666667
                              1234567890 1234567890 1234567890 1234567890 1234567890 1234567890 1234567890 1234567890 1234567890 1234567890 1234567890
De_novo_assembly_KT445934     CTCGGTCCTC CGCGGGGCGG GCCCGGGCCG GAGAGGGGGT CATCCCGTCC CCCCTCTCCG CGGCCTCGGT CTCGGGCGGA GAGCTCGGCG CGCGCGCGGG CGCGCGCTCG
Reference_assembly            CTCGGTCCTC CGCGGGGCGG GCCCGGGCCG GAGAGGGGGT CATCCCGTCC CCCCTCTCCG CGGCCTCGGT CTCGGGCGGA GAGCTCGGCG CGCGCGCGGG CGCGCGCTCG

                              5555555555 5555555555 5555555555 5555555555 5555555555 5555555555 5555555555 5555555555 5555555555 5555555555 5555555555
                              1111111111 1111111111 1111111112 2222222222 2222222222 2222222222 2222222222 2222222222 2222222222 2222222222 2222222222
                              7777777778 8888888889 9999999990 0000000001 1111111112 2222222223 3333333334 4444444445 5555555556 6666666667 7777777778
                              1234567890 1234567890 1234567890 1234567890 1234567890 1234567890 1234567890 1234567890 1234567890 1234567890 1234567890
De_novo_assembly_KT445934     CTCCGGCCGG CCTCGCCCGC GTACGGAGCG GGCCGAGACG CGGGTCTCGG CCCGGCGCCC GCCGCTCCCC GCGGCGGTGC GTTCCGCGGC CTCCCCGCCG CGCGCGGCCG
Reference_assembly            CTCCGGCCGG CCTCGCCCGC GTACGGAGCG GGCCGAGACG CGGGTCTCGG CCCGGCGCCC GCCGCTCCCC GCGGCGGTGC GTTCCGCGGC CTCCCCGCCG CGCGCGGCCG

                              5555555555 5555555555 5555555555 5555555555 5555555555 5555555555 5555555555 5555555555 5555555555 5555555555 5555555555
                              2222222222 2222222223 3333333333 3333333333 3333333333 3333333333 3333333333 3333333333 3333333333 3333333333 3333333333
                              8888888889 9999999990 0000000001 1111111112 2222222223 3333333334 4444444445 5555555556 6666666667 7777777778 8888888889
                              1234567890 1234567890 1234567890 1234567890 1234567890 1234567890 1234567890 1234567890 1234567890 1234567890 1234567890
De_novo_assembly_KT445934     GCGGGACGGC GAGCCGGCCG TCCCGCCCGC GCCAGCCGCG GCGCCGGCGG TTCCGCTCCG CCGGTCCGCC CCGCGTGCGT CCGCACGCCC GGCCTCCTGC CCTCCCTCGG
Reference_assembly            GCGGGACGGC GAGCCGGCCG TCCCGCCCGC GCCAGCCGCG GCGCCGGCGG TTCCGCTCCG CCGGTCCGCC CGGCGTGCGT CCGCACGCCC GGCCTCCTGC CCTCCCTCGG

                              5555555555 5555555555 5555555555 5555555555 5555555555 5555555555 5555555555 5555555555 5555555555 5555555555 5555555555
                              3333333334 4444444444 4444444444 4444444444 4444444444 4444444444 4444444444 4444444444 4444444444 4444444444 4444444445
                              9999999990 0000000001 1111111112 2222222223 3333333334 4444444445 5555555556 6666666667 7777777778 8888888889 9999999990
                              1234567890 1234567890 1234567890 1234567890 1234567890 1234567890 1234567890 1234567890 1234567890 1234567890 1234567890
De_novo_assembly_KT445934     GGCCTCGCCG CCGTTTCCCC CTTCCGTCGC AAGCCGCGTC CTCTCCTTCG TCCCCGCCGC CGTCGCCTCC CACCGCGCTT TCGCCCTCGG CCTCGCCGGC CGCGCCGGTC
Reference_assembly            GGCCTCGCCG CCGTTTCCCC CTTCCGTCGC AAGCCGCGTC CTCTCCTTCG TCCCCGCCGC CGTCGCCTCC CACCGCGCTT TCGCCCTCGG CCTCGCCGGC CGCGCCGGTC

                              5555555555 5555555555 5555555555 5555555555 5555555555 5555555555 5555555555 5555555555 5555555555 5555555555 5555555555
                              5555555555 5555555555 5555555555 5555555555 5555555555 5555555555 5555555555 5555555555 5555555555 5555555556 6666666666
                              0000000001 1111111112 2222222223 3333333334 4444444445 5555555556 6666666667 7777777778 8888888889 9999999990 0000000001
                              1234567890 1234567890 1234567890 1234567890 1234567890 1234567890 1234567890 1234567890 1234567890 1234567890 1234567890
De_novo_assembly_KT445934     GTGCGAGCGG GAGGTCCGGC GTGGGGCGTC CCGCAGCCGG TCTCCGCGCG GAGGCGCGGG GAGCGGGCGC CGCTCCCGAA TCCGTCCCCG TCCCGCCCGC CGCCGTGCGC
Reference_assembly            GTGCGAGCGG GAGGTCCGGC GTGGGGCGTC CCGCAGCCGG TCTCCGCGCG GAGGCGCGGG GAGCGGGCGC CGCTCCCGAA TCCGTCCCCG TCCCGCCCGC CGCCGTGCGC
```

```
                           5555555555 5555555555 5555555555 5555555555 5555555555 5555555555 5555555555 5555555555 5555555555 5555555555 5555555555
                           6666666666 6666666666 6666666666 6666666666 6666666666 6666666666 6666666666 6666666666 6666666667 7777777777 7777777777
                           1111111112 2222222223 3333333334 4444444445 5555555556 6666666667 7777777778 8888888889 9999999990 0000000001 1111111112
                           1234567890 1234567890 1234567890 1234567890 1234567890 1234567890 1234567890 1234567890 1234567890 1234567890 1234567890
De_novo_assembly_KT445934  GCGTCCGCCG CGGGCGCGCC GCCAGGGCGA GCGAGAGGAG GAGGCGTCGG AGGACGAGGG GCGGGGGAGG AAGGTGAGAG GCGGCGGGGG CGTTTCGGTG CGCGCGTCTC
Reference_assembly         GCGTCCGCCG CGGGCGCGCC GCCAGGGCGA GCGAGAGGAG GAGGCGTCGG AGGACGAGGG GCGGGGGAGG AAGGTGAGAG GCGGCGGGGG CGTTTCGGTG CGCGCGTCTC

                           5555555555 5555555555 5555555555 5555555555 5555555555 5555555555 5555555555 5555555555 5555555555 5555555555 5555555555
                           7777777777 7777777777 7777777777 7777777777 7777777777 7777777777 7777777777 7777777778 8888888888 8888888888 8888888888
                           2222222223 3333333334 4444444445 5555555556 6666666667 7777777778 8888888889 9999999990 0000000001 1111111112 2222222223
                           1234567890 1234567890 1234567890 1234567890 1234567890 1234567890 1234567890 1234567890 1234567890 1234567890 1234567890
De_novo_assembly_KT445934  CCGCACGGCG AGGAAGGGGC CGAGGTCGGC GCGGGCGCCG TCGGGCGGTC CGGCGCGGGC GCGGGCCGGC GGCGCCGCCG GCGCGGGCGG GGGCCTGGTC TGCTCCCGTC
Reference_assembly         CCGCACGGCG AGGAAGGGGC CGAGGTCGGC GCGGGCGCCG TCGGGCGGTC CGGCGCGGGC GCGGGCCGGC GGCGCCGCCG GCGCGGGCGG GGGCCTGGTC TGCTCCCGTC

                           5555555555 5555555555 5555555555 5555555555 5555555555 5555555555 5555555555 5555555555 5555555555 5555555555 5555555555
                           8888888888 8888888888 8888888888 8888888888 8888888888 8888888888 8888888889 9999999999 9999999999 9999999999 9999999999
                           3333333334 4444444445 5555555556 6666666667 7777777778 8888888889 9999999990 0000000001 1111111112 2222222223 3333333334
                           1234567890 1234567890 1234567890 1234567890 1234567890 1234567890 1234567890 1234567890 1234567890 1234567890 1234567890
De_novo_assembly_KT445934  CCCGTCGGTC GCGGCGGCGG CGGCGGCGGC GGTCCGTCGC GGCAGCGGGG CTTCGGCCGG GGCGGCGCGC GCCGTCCCGC GGGCGTCCGC GGCTCCTCCG CCCGGGCCGG
Reference_assembly         CCCGTCGGTC GCGGCGGCGG CGGCGGCGGC GGTCCGTCGC GGCAGCGGGG CTTCGGCCGG GGCGGCGCGC GCCGTCCCGC GGGCGTCCGC GGCTCCTCCG CCCGGGCCGG

                           5555555555 5555555555 5555555555 5555555555 5555555555 5555555556 6666666666 6666666666 6666666666 6666666666 6666666666
                           9999999999 9999999999 9999999999 9999999999 9999999999 9999999990 0000000000 0000000000 0000000000 0000000000 0000000000
                           4444444445 5555555556 6666666667 7777777778 8888888889 9999999990 0000000001 1111111112 2222222223 3333333334 4444444445
                           1234567890 1234567890 1234567890 1234567890 1234567890 1234567890 1234567890 1234567890 1234567890 1234567890 1234567890
De_novo_assembly_KT445934  GCCGAGCCGG GCGCCTGGTC CGTCCCCGAA GCGAGACAGG GTCGTTTCCC CAGGTCGGGA GCGAGGGCTC CCCGCCCTTC TCGTTCGGGT CGCGCTTCAT TGCCGGCCGG
Reference_assembly         GCCGAGCCGG GCGCCTGGTC CGTCCCCGAA GCGAGACAGG GTCGTTTCCC CAGGTCGGGA GCGAGGGCTC CCCGCCCTTC TCGTTCGGGT CGCGCTTCAT TGCCGGCCGG

                           6666666666 6666666666 6666666666 6666666666 6666666666 6666666666 6666666666 6666666666 6666666666 6666666666 6666666666
                           0000000000 0000000000 0000000000 0000000000 0000000001 1111111111 1111111111 1111111111 1111111111 1111111111 1111111111
                           5555555556 6666666667 7777777778 8888888889 9999999990 0000000001 1111111112 2222222223 3333333334 4444444445 5555555556
                           1234567890 1234567890 1234567890 1234567890 1234567890 1234567890 1234567890 1234567890 1234567890 1234567890 1234567890
De_novo_assembly_KT445934  CCGGCCGGCC GTCGCCGGCT TTTTTTTTCC CTCCCGCATC CGATATTCGT GTGCTCGTAC GGTCAGCGGA GGCGACGCTC GTCCGCCCCG CGGTCGCCCC GGCGTCGGGG
Reference_assembly         CCGGCCGGCC GTCGCCGGCT TTTTTTTTCC CTCCCGCATC CGATATTCGT GTGCTCGTAC GGTCAGCGGA GGCGACGCTC GTCCGCCCCG CGGTCGCCCC GGCGTCGGGG

                           6666666666 6666666666 6666666666 6666666666 6666666666 6666666666 6666666666 6666666666 6666666666 6666666666 6666666666
                           1111111111 1111111111 1111111111 1111111112 2222222222 2222222222 2222222222 2222222222 2222222222 2222222222 2222222222
                           6666666667 7777777778 8888888889 9999999990 0000000001 1111111112 2222222223 3333333334 4444444445 5555555556 6666666667
                           1234567890 1234567890 1234567890 1234567890 1234567890 1234567890 1234567890 1234567890 1234567890 1234567890 1234567890
De_novo_assembly_KT445934  CTGGCCGCGG GCGCGGGCCG AGCGCCTTCG GGCAAGGCGA GAGAGAACGA GAGCGGTCCC CCCGCGCGCG CGGGGCGGTG CCGAAAGTCA GACAACTCTT AGCGGTGGAT
Reference_assembly         CTGGCCGCGG GCGCGGGCCG AGCGCCTTCG GGCAAGGCGA GAGAGAACGA GAGCGGTCCC CCCGCGCGCG CGGGGCGGTG CCGAAAGTCA GACAACTCTT AGCGGTGGAT

                           6666666666 6666666666 6666666666 6666666666 6666666666 6666666666 6666666666 6666666666 6666666666 6666666666 6666666666
                           2222222222 2222222222 2222222223 3333333333 3333333333 3333333333 3333333333 3333333333 3333333333 3333333333 3333333333
                           7777777778 8888888889 9999999990 0000000001 1111111112 2222222223 3333333334 4444444445 5555555556 6666666667 7777777778
                           1234567890 1234567890 1234567890 1234567890 1234567890 1234567890 1234567890 1234567890 1234567890 1234567890 1234567890
De_novo_assembly_KT445934  CACTCGGCTC GTGCGTCGAT GAAGAACGCA GCTAGCTGCG AGAATTAATG TGAATTGCAG GACACATTGA TCATCGACAC TTCGAACGCA CTTGCGGCCC CGGGTTCCTC
Reference_assembly         CACTCGGCTC GTGCGTCGAT GAAGAACGCA GCTAGCTGCG AGAATTAATG TGAATTGCAG GACACATTGA TCATCGACAC TTCGAACGCA CTTGCGGCCC CGGGTTCCTC
```

```
                           6666666666 6666666666 6666666666 6666666666 6666666666 6666666666 6666666666 6666666666 6666666666 6666666666 6666666666
                           3333333333 3333333334 4444444444 4444444444 4444444444 4444444444 4444444444 4444444444 4444444444 4444444444 4444444444
                           8888888889 9999999990 0000000001 1111111112 2222222223 3333333334 4444444445 5555555556 6666666667 7777777778 8888888889
                           1234567890 1234567890 1234567890 1234567890 1234567890 1234567890 1234567890 1234567890 1234567890 1234567890 1234567890
De_novo_assembly_KT445934  CCGGGGCTAC GCCTGCCTGA GCGTCGCTTG ACGGTCAATC GCCGATGGCC GCCGTCCGCG GCGGCCGCGC GGCGCGGCTG GGGCGCCTCG CAGGCCCGCG CGCCCCGCCG
Reference_assembly         CCGGGGCTAC GCCTGCCTGA GCGTCGCTTG ACGGTCAATC GCCGACGGCC GCCGTCCGCG GCGGCCGCGC GGCGCGGCTG GGGCGCCTCG CAGGCCCGCG CGCCCCGCCG

                           6666666666 6666666666 6666666666 6666666666 6666666666 6666666666 6666666666 6666666666 6666666666 6666666666 6666666666
                           4444444445 5555555555 5555555555 5555555555 5555555555 5555555555 5555555555 5555555555 5555555555 5555555555 5555555556
                           9999999990 0000000001 1111111112 2222222223 3333333334 4444444445 5555555556 6666666667 7777777778 8888888889 9999999990
                           1234567890 1234567890 1234567890 1234567890 1234567890 1234567890 1234567890 1234567890 1234567890 1234567890 1234567890
De_novo_assembly_KT445934  GAGGCGGGTC GCGAGGGGGG GGGCCGCCGT CCGTCCGTCC GTCCGCCCGT CGGTCGGTCG GTTCGGGCGC CCGGATTCCC TCCCCCGCAC CCCCTCCGAG CGGCGTCGCG
Reference_assembly         GAGGCGGGTC GCGAGGGGGG GGGCCGCCGT CCGTCCGTCC GTCCGCCCGT CGGTCGGTCG GTTCGGGCGC CCGGATTCCC TCCCCCGCAC CCCCTCCGAG CGGCGTCGCG

                           6666666666 6666666666 6666666666 6666666666 6666666666 6666666666 6666666666 6666666666 6666666666 6666666666 6666666666
                           6666666666 6666666666 6666666666 6666666666 6666666666 6666666666 6666666666 6666666666 6666666666 6666666667 7777777777
                           0000000001 1111111112 2222222223 3333333334 4444444445 5555555556 6666666667 7777777778 8888888889 9999999990 0000000001
                           1234567890 1234567890 1234567890 1234567890 1234567890 1234567890 1234567890 1234567890 1234567890 1234567890 1234567890
De_novo_assembly_KT445934  CCGCGGGCCT TCGTCCCCCT AAGTGGAGAC CCAGGTCGGG GAGCTCGCCG AGCTCCCCGC GCTCCCGGAG CGCCCGCTTT GGCCGAGCTC GTCCCCACGG GGCGGCCGGG
Reference_assembly         CCGCGGGCCT TCGTCCCCCT AAGTGGAGAC CCAGGTCGGG GAGCTCGCCG AGCTCCCCGC GCTCCCGGAG CGCCCGCTTT GGCCGAGCTC GTCCCCACGG GGCGGCCGGG

                           6666666666 6666666666 6666666666 6666666666 6666666666 6666666666 6666666666 6666666666 6666666666 6666666666 6666666666
                           7777777777 7777777777 7777777777 7777777777 7777777777 7777777777 7777777777 7777777777 7777777778 8888888888 8888888888
                           1111111112 2222222223 3333333334 4444444445 5555555556 6666666667 7777777778 8888888889 9999999990 0000000001 1111111112
                           1234567890 1234567890 1234567890 1234567890 1234567890 1234567890 1234567890 1234567890 1234567890 1234567890 1234567890
De_novo_assembly_KT445934  CTTTCCGGTC GGTCGCGCGG CGCAGCGCGG CGGGGCCGGA CGTTCGTTCG TTCGTTCGTC CGGCCCCCCG CCCCGGAGGA GCGCTCCTCG CCCTCCCCGG CCCCGCGCGC
Reference_assembly         CTTTCCGGTC GGTCGCGCGG CGCAGCGCGG CGGGGCCGGA CGTTCGTTCG TTCGTTCGTC CGGCCCCCCG CCCCGGAGGA GCGCACCTCG CCCTCCCCGG CCCCGCGCGC

                           6666666666 6666666666 6666666666 6666666666 6666666666 6666666666 6666666666 6666666666 6666666666 6666666666 6666666666
                           8888888888 8888888888 8888888888 8888888888 8888888888 8888888888 8888888888 8888888889 9999999999 9999999999 9999999999
                           2222222223 3333333334 4444444445 5555555556 6666666667 7777777778 8888888889 9999999990 0000000001 1111111112 2222222223
                           1234567890 1234567890 1234567890 1234567890 1234567890 1234567890 1234567890 1234567890 1234567890 1234567890 1234567890
De_novo_assembly_KT445934  GGCTGCCTGC GGGTCGCGTT ACCGGCGGCG GTAACGCGCC GTGCTGCCGC GCGCGTGGCG GTCCGGGTCG GGGCGAGGCT GCCGGCCTCC GGTCGTCCGC CCGTCCGTCC
Reference_assembly         GGCTGCCTGC GGGTCGCGTT ACCGGCGGCG GTAACGCGCC GTGCTGCCGC GCGCGTGGCG GTCCGGGTCG GGGCGAGGCT GCCGGCCTCC GGTCGTCCGC CCGTCCGTCC

                           6666666666 6666666666 6666666666 6666666666 6666666666 6666666666 6666666667 7777777777 7777777777 7777777777 7777777777
                           9999999999 9999999999 9999999999 9999999999 9999999999 9999999999 9999999990 0000000000 0000000000 0000000000 0000000000
                           3333333334 4444444445 5555555556 6666666667 7777777778 8888888889 9999999990 0000000001 1111111112 2222222223 3333333334
                           1234567890 1234567890 1234567890 1234567890 1234567890 1234567890 1234567890 1234567890 1234567890 1234567890 1234567890
De_novo_assembly_KT445934  GGCCGAGCCC GGCGCGCGTC CCCGCGGGTC CGTCTCCGGC CACCGTGCGC CGGCGGCGGC GGCGGCGG-- -TGCGAACCG CCGGCGGCGC GCCGGCTCCC CCGTCCGGGC
Reference_assembly         GGCCGAGCCC GGCGCGCGTC CCCGCGGGTC CGTCTCCGGC CACCGTGCGC CGGCGGCGGC GGCGGCGGCG GTGCGAACCG CCGGCGGCGC GCCGGCTCCC CCGTCCGGGC

                           7777777777 7777777777 7777777777 7777777777 7777777777 7777777777 7777777777 7777777777 7777777777 7777777777 7777777777
                           0000000000 0000000000 0000000000 0000000000 0000000000 0000000001 1111111111 1111111111 1111111111 1111111111 1111111111
                           4444444445 5555555556 6666666667 7777777778 8888888889 9999999990 0000000001 1111111112 2222222223 3333333334 4444444445
                           1234567890 1234567890 1234567890 1234567890 1234567890 1234567890 1234567890 1234567890 1234567890 1234567890 1234567890
De_novo_assembly_KT445934  GTTCCTCCCT CGGCAGCGCC GGGAGCAGCC GCTTGGCGTC CGAAGGCGGG TGGCCGGGCG AGCGCGGGCT CGCCCGGGGC CCGGCGTTCG GGCCCCGTTT CCGATCGCGA
Reference_assembly         GTTCCTCCCT CGGCAGCGCC GGGAGCAGCC GCTTGGCGTC CGAAGGCGGG TGGCCGGGCG AGCGCGGGCT CGCCCGGGGC CCGGCGTTCG GGCCCCGTTT CCGATCGCGA
```

```
                                  7777777777 7777777777 7777777777 7777777777 7777777777 7777777777 7777777777 7777777777 7777777777 7777777777 7777777777
                                  1111111111 1111111111 1111111111 1111111111 1111111112 2222222222 2222222222 2222222222 2222222222 2222222222 2222222222
                                  5555555556 6666666667 7777777778 8888888889 9999999990 0000000001 1111111112 2222222223 3333333334 4444444445 5555555556
                                  1234567890 1234567890 1234567890 1234567890 1234567890 1234567890 1234567890 1234567890 1234567890 1234567890 1234567890
De_novo_assembly_KT445934         CCTCAGGTCA GACGTGGCGA CCCGCTGAAT TTAAGCATAT TAGTCAGCGG AGGAAAAGAA ACTAACGAGG ATTCCCTCAG TAACGGCGAG TGAAGAGGGA AGAGCCCAGC
Reference_assembly                CCTCAGGTCA GACGTGGCGA CCCGCTGAAT TTAAGCATAT TAGTCAGCGG AGGAAAAGAA ACTAACGAGG ATTCCCTCAG TAACGGCGAG TGAAGAGGGA AGAGCCCAGC

                                  7777777777 7777777777 7777777777 7777777777 7777777777 7777777777 7777777777 7777777777 7777777777 7777777777 7777777777
                                  2222222222 2222222222 2222222222 2222222223 3333333333 3333333333 3333333333 3333333333 3333333333 3333333333 3333333333
                                  6666666667 7777777778 8888888889 9999999990 0000000001 1111111112 2222222223 3333333334 4444444445 5555555556 6666666667
                                  1234567890 1234567890 1234567890 1234567890 1234567890 1234567890 1234567890 1234567890 1234567890 1234567890 1234567890
De_novo_assembly_KT445934         GCCGAATCCC CGCCCCGCGG TGGGGCGCGG GAGGTGTGGC GTACGGAAGC CCCCATCCCC GGCGCCGCTC TCGGGGGGCC CAAGTCCTTC TGATCGAGGC CCAGCCCGCG
Reference_assembly                GCCGAATCCC CGCCCCGCGG TGGGGCGCGG GAGGTGTGGC GTACGGAAGC CCCCATCCCC GGCGCCGCTC TCGGGGGGCC CAAGTCCTTC TGATCGAGGC CCAGCCCGCG

                                  7777777777 7777777777 7777777777 7777777777 7777777777 7777777777 7777777777 7777777777 7777777777 7777777777 7777777777
                                  3333333333 3333333333 3333333334 4444444444 4444444444 4444444444 4444444444 4444444444 4444444444 4444444444 4444444444
                                  7777777778 8888888889 9999999990 0000000001 1111111112 2222222223 3333333334 4444444445 5555555556 6666666667 7777777778
                                  1234567890 1234567890 1234567890 1234567890 1234567890 1234567890 1234567890 1234567890 1234567890 1234567890 1234567890
De_novo_assembly_KT445934         GACGGTGTGA GGCCGGTAGC GGCCCCCCGG CGCGCCGGGC CCGGGGCTTC TCGGAGTCGG GTTGCTTGGG AATGCAGCCC AAAGCGGGTG GTAAACTCCA TCTAAGGCTA
Reference_assembly                GACGGTGTGA GGCCGGTAGC GGCCCCCCGG CGCGCCGGGC CCGGGGCTTC TCGGAGTCGG GTTGCTTGGG AATGCAGCCC AAAGCGGGTG GTAAACTCCA TCTAAGGCTA

                                  7777777777 7777777777 7777777777 7777777777 7777777777 7777777777 7777777777 7777777777 7777777777 7777777777 7777777777
                                  4444444444 4444444445 5555555555 5555555555 5555555555 5555555555 5555555555 5555555555 5555555555 5555555555 5555555555
                                  8888888889 9999999990 0000000001 1111111112 2222222223 3333333334 4444444445 5555555556 6666666667 7777777778 8888888889
                                  1234567890 1234567890 1234567890 1234567890 1234567890 1234567890 1234567890 1234567890 1234567890 1234567890 1234567890
De_novo_assembly_KT445934         AATACCGGCA CGAGACCGAT AGCCAACAAG TACCGTAAGG GAAAGTTGAA AAGAACTTTG AAGAGAGAGT TCAAGAGGGC GTGAAACCGT TAAGAGGTAA ACGGGTGGGG
Reference_assembly                AATACCGGCA CGAGACCGAT AGCCAACAAG TACCGTAAGG GAAAGTTGAA AAGAACTTTG AAGAGAGAGT TCAAGAGGGC GTGAAACCGT TAAGAGGTAA ACGGGTGGGG

                                  7777777777 7777777777 7777777777 7777777777 7777777777 7777777777 7777777777 7777777777 7777777777 7777777777 7777777777
                                  5555555556 6666666666 6666666666 6666666666 6666666666 6666666666 6666666666 6666666666 6666666666 6666666666 6666666667
                                  9999999990 0000000001 1111111112 2222222223 3333333334 4444444445 5555555556 6666666667 7777777778 8888888889 9999999990
                                  1234567890 1234567890 1234567890 1234567890 1234567890 1234567890 1234567890 1234567890 1234567890 1234567890 1234567890
De_novo_assembly_KT445934         TCCGCGCAGT CGGCCCGGAG GATTCAACCC GGCGGGCCAA GGTCGGCCGG CGCGGGCGCC GTCGGATCCC CGCCTCCGCC TCCCCTCCGT CCCTCCCCTT CGCCGGGGCG
Reference_assembly                TCCGCGCAGT CGGCCCGGAG GATTCAACCC GGCGGGCCAA GGTCGGCCGG CGCGGGCGCC GTCGGATCCC CGCCTCCGCC TCCCCTCCGT CCCTCCCCTT CGCCGGGGCG

                                  7777777777 7777777777 7777777777 7777777777 7777777777 7777777777 7777777777 7777777777 7777777777 7777777777 7777777777
                                  7777777777 7777777777 7777777777 7777777777 7777777777 7777777777 7777777777 7777777777 7777777777 7777777778 8888888888
                                  0000000001 1111111112 2222222223 3333333334 4444444445 5555555556 6666666667 7777777778 8888888889 9999999990 0000000001
                                  1234567890 1234567890 1234567890 1234567890 1234567890 1234567890 1234567890 1234567890 1234567890 1234567890 1234567890
De_novo_assembly_KT445934         GGGCGGGCCC AGGGGGGGCG GGCGGGCCGG GGACCGCCGC CCGGCCGGCG TCCGGCCCCC GTCGGGCGCA TTTCCTCCGC GGCGGTGCGC CGCGACCGGC TCCGGGACGG
Reference_assembly                GGGCGGGCCC AGGGG????? ????GGCCGG GGACCGCCGC CCGGCCGGCG TCCGGCCCCC GTCGGGCGCA TTTCCTCCGC GGCGGTGCGC CGCGACCGGC TCCGGGACGG

                                  7777777777 7777777777 7777777777 7777777777 7777777777 7777777777 7777777777 7777777777 7777777777 7777777777 7777777777
                                  8888888888 8888888888 8888888888 8888888888 8888888888 8888888888 8888888888 8888888888 8888888889 9999999999 9999999999
                                  1111111112 2222222223 3333333334 4444444445 5555555556 6666666667 7777777778 8888888889 9999999990 0000000001 1111111112
                                  1234567890 1234567890 1234567890 1234567890 1234567890 1234567890 1234567890 1234567890 1234567890 1234567890 1234567890
De_novo_assembly_KT445934         CTGGGAAGGG CTGCCGGCGG GCAGGTGGCC CGGCGCCGCG CGAGCGGCCG CCGGGTGTTA TAGCCGCCGG GCCCGGATCG TCGCCGAATC CCGGGGCCGA GGGAGAGGAC
Reference_assembly                CTGGGAAGGG CTGCCGGCGG GCAGGTGGCC CGGCGCCGCG CGAGCGGCCG CCGGGTGTTA TAGCCGCCGG GCCCGGATCG TCGCCGAATC CCGGGGCCGA GGGAGAGGAC
```

```
                            7777777777 7777777777 7777777777 7777777777 7777777777 7777777777 7777777777 7777777778 8888888888 8888888888 8888888888
                            9999999999 9999999999 9999999999 9999999999 9999999999 9999999999 9999999999 9999999990 0000000000 0000000000 0000000000
                            2222222223 3333333334 4444444445 5555555556 6666666667 7777777778 8888888889 9999999990 0000000001 1111111112 2222222223
                            1234567890 1234567890 1234567890 1234567890 1234567890 1234567890 1234567890 1234567890 1234567890 1234567890 1234567890
De_novo_assembly_KT445934   CGCCGCCGCG CCCTCCCCCG GAGGGGGCGG CCCCCCGGAG GGCCCCCCGC GGCCGGACCG GCGTCGGGCC GGCCGCGCCG CGCGCGCGTC CGCGCCGCCG CCGTACGCCG
Reference_assembly          CGCCGCCGCG CCCTCCCCCG GAGGGGGCGG CCCCCCGGAG GGCCCCCCGC GGCCGGACCG GCGTCGGGCC GGCCGCGCCG CGCGCGCGTC CGCGCCGCCG CCGTACGCCG

                            8888888888 8888888888 8888888888 8888888888 8888888888 8888888888 8888888888 8888888888 8888888888 8888888888 8888888888
                            0000000000 0000000000 0000000000 0000000000 0000000000 0000000000 0000000001 1111111111 1111111111 1111111111 1111111111
                            3333333334 4444444445 5555555556 6666666667 7777777778 8888888889 9999999990 0000000001 1111111112 2222222223 3333333334
                            1234567890 1234567890 1234567890 1234567890 1234567890 1234567890 1234567890 1234567890 1234567890 1234567890 1234567890
De_novo_assembly_KT445934   CCGCTCGCTC TCTCTCCGTT CCCCGCCCCG GGTCCGTCCC GGGGCGCGGG GGCGGGGGGG GTCGGGTGTC CGGCGCGCGG CTCGGCGCGG CGCCGCGCGT GTGGCGCGCG
Reference_assembly          CCGCTCGCTC TCTCTCCGTT CCCCGCCCCG GGTCCGTCCC GGGGCGCGGG GGCGGGGGGG GTCGGGTGTC CGGCGCGCGG CTCGGCGCGG CGCCGCGCGT GTGGCGCGCG

                            8888888888 8888888888 8888888888 8888888888 8888888888 8888888888 8888888888 8888888888 8888888888 8888888888 8888888888
                            1111111111 1111111111 1111111111 1111111111 1111111111 1111111112 2222222222 2222222222 2222222222 2222222222 2222222222
                            4444444445 5555555556 6666666667 7777777778 8888888889 9999999990 0000000001 1111111112 2222222223 3333333334 4444444445
                            1234567890 1234567890 1234567890 1234567890 1234567890 1234567890 1234567890 1234567890 1234567890 1234567890 1234567890
De_novo_assembly_KT445934   CCTCCAGCCC GGCGCGGGCG AGGCCGCGGG GGGCGCCGGG GGGGAACCTT CCCCCTTCTG TTCGGGCCGC CTCCGTTCCC GCGGGGGCGG CCCGTTCGGG GGACGGGCCC
Reference_assembly          CCTCCAGCCC GGCGCGGGCG AGGCCGCGGG GGGCGCCGGG GGGGAACCTT CCCCCTTCTG TTCGGGCCGC CTCCGTTCCC GCGGGGGCGG CCCGTTCGGG GGACGGGCCC

                            8888888888 8888888888 8888888888 8888888888 8888888888 8888888888 8888888888 8888888888 8888888888 8888888888 8888888888
                            2222222222 2222222222 2222222222 2222222222 2222222223 3333333333 3333333333 3333333333 3333333333 3333333333 3333333333
                            5555555556 6666666667 7777777778 8888888889 9999999990 0000000001 1111111112 2222222223 3333333334 4444444445 5555555556
                            1234567890 1234567890 1234567890 1234567890 1234567890 1234567890 1234567890 1234567890 1234567890 1234567890 1234567890
De_novo_assembly_KT445934   GCCGGCCCCC GGCGCCGCTG TCCGACCGGG GCGGACTGCG CTCAGTGCGC CCCGACCGCG CGGCGCCGCC GGGCCGGGCT CGGGCCACGC CAGGGCGCCC GGGGTCCGCG
Reference_assembly          GCCGGCCCCC GGCGCCGCTG TCCGACCGGG GCGGACTGCG CTCAGTGCGC CCCGACCGCG CGGCGCCGCC GGGCCGGGCT CGGGCCACGC CAGGGCGCCC GGGGTCCGCG

                            8888888888 8888888888 8888888888 8888888888 8888888888 8888888888 8888888888 8888888888 8888888888 8888888888 8888888888
                            3333333333 3333333333 3333333333 3333333334 4444444444 4444444444 4444444444 4444444444 4444444444 4444444444 4444444444
                            6666666667 7777777778 8888888889 9999999990 0000000001 1111111112 2222222223 3333333334 4444444445 5555555556 6666666667
                            1234567890 1234567890 1234567890 1234567890 1234567890 1234567890 1234567890 1234567890 1234567890 1234567890 1234567890
De_novo_assembly_KT445934   GCGACGTCGG CTACCCACCC GACCCGTCTT GAAACACGGA CCAAGGAGTC TAGCACGCGC GCGAGTCGGC GGCTCGCGCG AAAGCCCGCG GCGCAATGAA GGTGAGGGCC
Reference_assembly          GCGACGTCGG CTACCCACCC GACCCGTCTT GAAACACGGA CCAAGGAGTC TAGCACGCGC GCGAGTCGGC GGCTCGCGCG AAAGCCCGCG GCGCAATGAA GGTGAGGGCC

                            8888888888 8888888888 8888888888 8888888888 8888888888 8888888888 8888888888 8888888888 8888888888 8888888888 8888888888
                            4444444444 4444444444 4444444445 5555555555 5555555555 5555555555 5555555555 5555555555 5555555555 5555555555 5555555555
                            7777777778 8888888889 9999999990 0000000001 1111111112 2222222223 3333333334 4444444445 5555555556 6666666667 7777777778
                            1234567890 1234567890 1234567890 1234567890 1234567890 1234567890 1234567890 1234567890 1234567890 1234567890 1234567890
De_novo_assembly_KT445934   GGCGCGCGCC GGCTGAGGTG GGATCCCGGG GCGGCAGGCC GGAAGGCCCC GGGCGCACCA CCGGCCCGTC TCGCCCGCCT CGCCGGGGAG GTGGAGCATG AGCGCGCGTG
Reference_assembly          GGCGCGCGCC GGCTGAGGTG GGATCCCGGG GCGGCAGGCC GGAAGGCCCC GGGCGCACCA CCGGCCCGTC TCGCCCGCCT CGCCGGGGAG GTGGAGCATG AGCGCGCGTG

                            8888888888 8888888888 8888888888 8888888888 8888888888 8888888888 8888888888 8888888888 8888888888 8888888888 8888888888
                            5555555555 5555555556 6666666666 6666666666 6666666666 6666666666 6666666666 6666666666 6666666666 6666666666 6666666666
                            8888888889 9999999990 0000000001 1111111112 2222222223 3333333334 4444444445 5555555556 6666666667 7777777778 8888888889
                            1234567890 1234567890 1234567890 1234567890 1234567890 1234567890 1234567890 1234567890 1234567890 1234567890 1234567890
De_novo_assembly_KT445934   CTAGGACCCG AAAGATGGTG AACTATGCCT GGGCAGGGCG AAGCCAGAGG AAACTCTGGT GGAGGTCCGT AGCGGTCCTG ACGTGCAAAT CGGTCGTCCG ACCCGGGTAT
Reference_assembly          CTAGGACCCG AAAGATGGTG AACTATGCCT GGGCAGGGCG AAGCCAGAGG AAACTCTGGT GGAGGTCCGT AGCGGTCCTG ACGTGCAAAT CGGTCGTCCG ACCCGGGTAT
```

```
                                 8888888888 8888888888 8888888888 8888888888 8888888888 8888888888 8888888888 8888888888 8888888888 8888888888 8888888888
                                 6666666667 7777777777 7777777777 7777777777 7777777777 7777777777 7777777777 7777777777 7777777777 7777777777 7777777778
                                 9999999990 0000000001 1111111112 2222222223 3333333334 4444444445 5555555556 6666666667 7777777778 8888888889 9999999990
                                 1234567890 1234567890 1234567890 1234567890 1234567890 1234567890 1234567890 1234567890 1234567890 1234567890 1234567890
De_novo_assembly_KT445934        AGGGGCGAAA GACTAATCGA ACCATCTAGT AGCTGGTTCC CTCCGAAGTT TCCCTCAGGA TAGCTGGCGC TCGGGGCGGC GGTGCAGTTT TACCCGGTAA AGCGAATGAT
Reference_assembly               AGGGGCGAAA GACTAATCGA ACCATCTAGT AGCTGGTTCC CTCCGAAGTT TCCCTCAGGA TAGCTGGCGC TCGGGGCGGC GGTGCAGTTT TACCCGGTAA AGCGAATGAT

                                 8888888888 8888888888 8888888888 8888888888 8888888888 8888888888 8888888888 8888888888 8888888888 8888888888 8888888888
                                 8888888888 8888888888 8888888888 8888888888 8888888888 8888888888 8888888888 8888888888 8888888888 8888888889 9999999999
                                 0000000001 1111111112 2222222223 3333333334 4444444445 5555555556 6666666667 7777777778 8888888889 9999999990 0000000001
                                 1234567890 1234567890 1234567890 1234567890 1234567890 1234567890 1234567890 1234567890 1234567890 1234567890 1234567890
De_novo_assembly_KT445934        TAGAGGTCTT GGGGCCGAAA CGATCTCAAC CTATTCTCAA ACTTTCAATG GGTAAGACGC CCGGCTCGCT GGCGTGGAGC CGGGCCGTGG AATGCGAGCG CTCAGTGGGC
Reference_assembly               TAGAGGTCTT GGGGCCGAAA CGATCTCAAC CTATTCTCAA ACTTTCAATG GGTAAGACGC CCGGCTCGCT GGCGTGGAGC CGGGCCGTGG AATGCGAGCG CTCAGTGGGC

                                 8888888888 8888888888 8888888888 8888888888 8888888888 8888888888 8888888888 8888888888 8888888889 9999999999 9999999999
                                 9999999999 9999999999 9999999999 9999999999 9999999999 9999999999 9999999999 9999999999 9999999990 0000000000 0000000000
                                 1111111112 2222222223 3333333334 4444444445 5555555556 6666666667 7777777778 8888888889 9999999990 0000000001 1111111112
                                 1234567890 1234567890 1234567890 1234567890 1234567890 1234567890 1234567890 1234567890 1234567890 1234567890 1234567890
De_novo_assembly_KT445934        CACTTTTGGT AAGCAGAACT GGCGCTGCGG GATGAACCGA ACGCCGGGTT AAGGCGCCCG ATGCCGACGC TCATCAGAGC CCAGAAAAGG TGTTGGTTGA TCTAGACAGC
Reference_assembly               CACTTTTGGT AAGCAGAACT GGCGCTGCGG GATGAACCGA ACGCCGGGTT AAGGCGCCCG ATGCCGACGC TCATCAGAGC CCAGAAAAGG TGTTGGTTGA TCTAGACAGC

                                 9999999999 9999999999 9999999999 9999999999 9999999999 9999999999 9999999999 9999999999 9999999999 9999999999 9999999999
                                 0000000000 0000000000 0000000000 0000000000 0000000000 0000000000 0000000000 0000000001 1111111111 1111111111 1111111111
                                 2222222223 3333333334 4444444445 5555555556 6666666667 7777777778 8888888889 9999999990 0000000001 1111111112 2222222223
                                 1234567890 1234567890 1234567890 1234567890 1234567890 1234567890 1234567890 1234567890 1234567890 1234567890 1234567890
De_novo_assembly_KT445934        AGGACGGTGG CCATGGAAGT CGGAACCCGC TAAGGAGTGT GTAACAACTC ACCTGCCGAA TCAACTAGCC CTGAAAATGG ATGGCGCTGG AGCGTCGGGC CCATACCCGG
Reference_assembly               AGGACGGTGG CCATGGAAGT CGGAACCCGC TAAGGAGTGT GTAACAACTC ACCTGCCGAA TCAACTAGCC CTGAAAATGG ATGGCGCTGG AGCGTCGGGC CCATACCCGG

                                 9999999999 9999999999 9999999999 9999999999 9999999999 9999999999 9999999999 9999999999 9999999999 9999999999 9999999999
                                 1111111111 1111111111 1111111111 1111111111 1111111111 1111111111 1111111112 2222222222 2222222222 2222222222 2222222222
                                 3333333334 4444444445 5555555556 6666666667 7777777778 8888888889 9999999990 0000000001 1111111112 2222222223 3333333334
                                 1234567890 1234567890 1234567890 1234567890 1234567890 1234567890 1234567890 1234567890 1234567890 1234567890 1234567890
De_novo_assembly_KT445934        CCGTCGCCGG CGGTGCGGAG CCGCGGGGGC TACGCCGCGA CGAGTAGGAG GGCCGCTGCG GTGCGCCTGG AAGCCTGGGG CGCGGGCCCG GGTGGAGCCG CCGCAGGTGC
Reference_assembly               CCGTCGCCGG CGGTGCGGAG CCGCGGGGGC TACGCCGCGA CGAGTAGGAG GGCCGCTGCG GTGCGCCTGG AAGCCTGGGG CGCGGGCCCG GGTGGAGCCG CCGCAGGTGC

                                 9999999999 9999999999 9999999999 9999999999 9999999999 9999999999 9999999999 9999999999 9999999999 9999999999 9999999999
                                 2222222222 2222222222 2222222222 2222222222 2222222222 2222222223 3333333333 3333333333 3333333333 3333333333 3333333333
                                 4444444445 5555555556 6666666667 7777777778 8888888889 9999999990 0000000001 1111111112 2222222223 3333333334 4444444445
                                 1234567890 1234567890 1234567890 1234567890 1234567890 1234567890 1234567890 1234567890 1234567890 1234567890 1234567890
De_novo_assembly_KT445934        AGATCTTGGT GGTAGTAGCA ACTATTCAAA CGAGAGCTTT GAAGGCCGAA GTGGAGCAGG GTTCCATGTG AACAGCAGTT GAACATGGGT CAGTCGGTCC TAAGCGATAG
Reference_assembly               AGATCTTGGT GGTAGTAGCA ACTATTCAAA CGAGAGCTTT GAAGGCCGAA GTGGAGCAGG GTTCCATGTG AACAGCAGTT GAACATGGGT CAGTCGGTCC TAAGCGATAG

                                 9999999999 9999999999 9999999999 9999999999 9999999999 9999999999 9999999999 9999999999 9999999999 9999999999 9999999999
                                 3333333333 3333333333 3333333333 3333333333 3333333334 4444444444 4444444444 4444444444 4444444444 4444444444 4444444444
                                 5555555556 6666666667 7777777778 8888888889 9999999990 0000000001 1111111112 2222222223 3333333334 4444444445 5555555556
                                 1234567890 1234567890 1234567890 1234567890 1234567890 1234567890 1234567890 1234567890 1234567890 1234567890 1234567890
De_novo_assembly_KT445934        GCGAGCGCCG TTCCGAAGGG ACGGGCGATG GCCTCCGTTG CCCTCAGCCG ATCGAAAGGG AGTCGGGTTC AGATCCCCGA ATCCGGAGCG GCGGAGACGG GCGCCGCGAG
Reference_assembly               GCGAGCGCCG TTCCGAAGGG ACGGGCGATG GCCTCCGTTG CCCTCAGCCG ATCGAAAGGG AGTCGGGTTC AGATCCCCGA ATCCGGAGCG GCGGAGACGG GCGCCGCGAG
```

```
                               9999999999 9999999999 9999999999 9999999999 9999999999 9999999999 9999999999 9999999999 9999999999 9999999999 9999999999
                               4444444444 4444444444 4444444444 4444444445 5555555555 5555555555 5555555555 5555555555 5555555555 5555555555 5555555555
                               6666666667 7777777778 8888888889 9999999990 0000000001 1111111112 2222222223 3333333334 4444444445 5555555556 6666666667
                               1234567890 1234567890 1234567890 1234567890 1234567890 1234567890 1234567890 1234567890 1234567890 1234567890 1234567890
De_novo_assembly_KT445934      GCGCCCAGTG CGGTAACGCA AGCGATCCCG GAGAAGCCGG CGGGAGCCCC GGGGAGAGTT CTCTTTTCTT TGTGAAGGGC CGGGCGCCCT GGAACGGGTT CGCCCCGAGA
Reference_assembly             GCGCCCAGTG CGGTAACGCA AGCGATCCCG GAGAAGCCGG CGGGAGCCCC GGGGAGAGTT CTCTTTTCTT TGTGAAGGGC CGGGCGCCCT GGAACGGGTT CGCCCCGAGA

                               9999999999 9999999999 9999999999 9999999999 9999999999 9999999999 9999999999 9999999999 9999999999 9999999999 9999999999
                               5555555555 5555555555 5555555556 6666666666 6666666666 6666666666 6666666666 6666666666 6666666666 6666666666 6666666666
                               7777777778 8888888889 9999999990 0000000001 1111111112 2222222223 3333333334 4444444445 5555555556 6666666667 7777777778
                               1234567890 1234567890 1234567890 1234567890 1234567890 1234567890 1234567890 1234567890 1234567890 1234567890 1234567890
De_novo_assembly_KT445934      GAGGGGCCCG CGCCTTGGAA AGCGTCGCGG TTCCGGCGGC GTCCGGTGAG CTCTCGCTGG CCCGTGAAAA TCCGGGGGAG AGGGTGTAAA TCTCGCGCCG GGCCGTACCC
Reference_assembly             GAGGGGCCCG CGCCTTGGAA AGCGTCGCGG TTCCGGCGGC GTCCGGTGAG CTCTCGCTGG CCCGTGAAAA TCCGGGGGAG AGGGTGTAAA TCTCGCGCCG GGCCGTACCC

                               9999999999 9999999999 9999999999 9999999999 9999999999 9999999999 9999999999 9999999999 9999999999 9999999999 9999999999
                               6666666666 6666666667 7777777777 7777777777 7777777777 7777777777 7777777777 7777777777 7777777777 7777777777 7777777777
                               8888888889 9999999990 0000000001 1111111112 2222222223 3333333334 4444444445 5555555556 6666666667 7777777778 8888888889
                               1234567890 1234567890 1234567890 1234567890 1234567890 1234567890 1234567890 1234567890 1234567890 1234567890 1234567890
De_novo_assembly_KT445934      ATATCCGCAG CAGGTCTCCA AGGTGAACAG CCTCTGGCAT GTTGGACCAA TGTAGGTAAG GGAAGTCGGC AAGCCGGATC CGTAACTTCG GGATAAGGAT TGGCTCTAAG
Reference_assembly             ATATCCGCAG CAGGTCTCCA AGGTGAACAG CCTCTGGCAT GTTGGACCAA TGTAGGTAAG GGAAGTCGGC AAGCCGGATC CGTAACTTCG GGATAAGGAT TGGCTCTAAG

                               9999999999 9999999999 9999999999 9999999999 9999999999 9999999999 9999999999 9999999999 9999999999 9999999999 9999999999
                               7777777778 8888888888 8888888888 8888888888 8888888888 8888888888 8888888888 8888888888 8888888888 8888888888 8888888889
                               9999999990 0000000001 1111111112 2222222223 3333333334 4444444445 5555555556 6666666667 7777777778 8888888889 9999999990
                               1234567890 1234567890 1234567890 1234567890 1234567890 1234567890 1234567890 1234567890 1234567890 1234567890 1234567890
De_novo_assembly_KT445934      GGCTGGGTCG GTCGGGCTGG GGCGCGAAGC GGGGCTGGGC GCGCGCCGCG GCTGGACGAG GCGCCGCCCG CCCCCGCCCC CCCTTTCCCC GCTCCCGCTC GCCGGGGCGC
Reference_assembly             GGCTGGGTCG GTCGGGCTGG GGCGCGAAGC GGGGCTGGGC GCGCGCCGCG GCTGGACGAG GCGCCGCCCG CCCCCGCCCC CCCTTTCCC? ?????????? ??????????

                                                                                                                                          1 1111111111
                               9999999999 9999999999 9999999999 9999999999 9999999999 9999999999 9999999999 9999999999 9999999999 9999999990 0000000000
                               9999999999 9999999999 9999999999 9999999999 9999999999 9999999999 9999999999 9999999999 9999999999 9999999990 0000000000
                               0000000001 1111111112 2222222223 3333333334 4444444445 5555555556 6666666667 7777777778 8888888889 9999999990 0000000001
                               1234567890 1234567890 1234567890 1234567890 1234567890 1234567890 1234567890 1234567890 1234567890 1234567890 1234567890
De_novo_assembly_KT445934      CGGGGGGGGG GGTCAGCGGG CGGCGCGGCG GCGGCGACTC TGGACGCGCG CCGGGCCCTT CCCGTGGATC GCCCCAGCTG CGGCGGGCGC CGCTCGCCCC CCTCCTTGCC
Reference_assembly             ?????????? ???????GGG CGGCGCGGCG GCGGCGACTC TGGACGCGCG CCGGGCCCTT CCCGTGGATC GCCCCAGCTG CGGCGGGCGC CGCTC????? ??????????

                               1111111111 1111111111 1111111111 1111111111 1111111111 1111111111 1111111111 1111111111 1111111111 1111111111 1111111111
                               0000000000 0000000000 0000000000 0000000000 0000000000 0000000000 0000000000 0000000000 0000000000 0000000000 0000000000
                               0000000000 0000000000 0000000000 0000000000 0000000000 0000000000 0000000000 0000000000 0000000001 1111111111 1111111111
                               1111111112 2222222223 3333333334 4444444445 5555555556 6666666667 7777777778 8888888889 9999999990 0000000001 1111111112
                               1234567890 1234567890 1234567890 1234567890 1234567890 1234567890 1234567890 1234567890 1234567890 1234567890 1234567890
De_novo_assembly_KT445934      CCTCCGCCCC CCCGCTCCCG GCGCCCCTCC CGTCGGCCGT CGTCCCGGCC GCCCCCCGTC CCGAGCGCCC TCCCCGCGAG GGCGCGAGGG GCGGCGGCGG CGGCCGCGGG
Reference_assembly             ?????????? ?????????? ?????????? ?????????? ?????????? ?????????? ?????????? ?????????? ?????????? ?????????? ??????????

                               1111111111 1111111111 1111111111 1111111111 1111111111 1111111111 1111111111 1111111111 1111111111 1111111111 1111111111
                               0000000000 0000000000 0000000000 0000000000 0000000000 0000000000 0000000000 0000000000 0000000000 0000000000 0000000000
                               1111111111 1111111111 1111111111 1111111111 1111111111 1111111111 1111111111 1111111112 2222222222 2222222222 2222222222
                               2222222223 3333333334 4444444445 5555555556 6666666667 7777777778 8888888889 9999999990 0000000001 1111111112 2222222223
                               1234567890 1234567890 1234567890 1234567890 1234567890 1234567890 1234567890 1234567890 1234567890 1234567890 1234567890
De_novo_assembly_KT445934      CGCGGCGGCG GCGGGGGGGG GCCCGCCGGC GGCGCCGGGC GGGGCGGTCC CGGGCGGGGG GGGTCTCCGG GCCGGCGCCC CGCCTCGGCC GGCGCCTAGC AGCCGGCTTA
Reference_assembly             ?????????? ?????????? ?????????? ?GCGCCGGGC GGGGCGGTCC CGGGCGGGGG GGGTCTCCGG GCCGGCGCCC CGCCTCGGCC GGCGCCTAGC AGCCGGCTTA
```

```
                             1111111111 1111111111 1111111111 1111111111 1111111111 1111111111 1111111111 1111111111 1111111111 1111111111 1111111111
                             0000000000 0000000000 0000000000 0000000000 0000000000 0000000000 0000000000 0000000000 0000000000 0000000000 0000000000
                             2222222222 2222222222 2222222222 2222222222 2222222222 2222222222 2222222223 3333333333 3333333333 3333333333 3333333333
                             3333333334 4444444445 5555555556 6666666667 7777777778 8888888889 9999999990 0000000001 1111111112 2222222223 3333333334
                             1234567890 1234567890 1234567890 1234567890 1234567890 1234567890 1234567890 1234567890 1234567890 1234567890 1234567890
De_novo_assembly_KT445934    GAACTGGTGC GGACCAGGGG AATCCGACTG TTTAATTAAA ACAAAGCATC GCGAAGGCCC GCGGCGGGTG TTGACGCGAT GTGATTTCTG CCCAGTGCTC TGAATGTCAA
Reference_assembly           GAACTGGTGC GGACCAGGGG AATCCGACTG TTTAATTAAA ACAAAGCATC GCGAAGGCCC GCGGCGGGTG TTGACGCGAT GTGATTTCTG CCCAGTGCTC TGAATGTCAA

                             1111111111 1111111111 1111111111 1111111111 1111111111 1111111111 1111111111 1111111111 1111111111 1111111111 1111111111
                             0000000000 0000000000 0000000000 0000000000 0000000000 0000000000 0000000000 0000000000 0000000000 0000000000 0000000000
                             3333333333 3333333333 3333333333 3333333333 3333333333 3333333334 4444444444 4444444444 4444444444 4444444444 4444444444
                             4444444445 5555555556 6666666667 7777777778 8888888889 9999999990 0000000001 1111111112 2222222223 3333333334 4444444445
                             1234567890 1234567890 1234567890 1234567890 1234567890 1234567890 1234567890 1234567890 1234567890 1234567890 1234567890
De_novo_assembly_KT445934    AGTGAAGAAA TTCAATGAAG CGCGGGTAAA CGGCGGGAGT AACTATGACT CTCTTAAGGT AGCCAAATGC CTCGTCATCT AATTAGTGAC GCGCATGAAT GGATGAACGA
Reference_assembly           AGTGAAGAAA TTCAATGAAG CGCGGGTAAA CGGCGGGAGT AACTATGACT CTCTTAAGGT AGCCAAATGC CTCGTCATCT AATTAGTGAC GCGCATGAAT GGATGAACGA

                             1111111111 1111111111 1111111111 1111111111 1111111111 1111111111 1111111111 1111111111 1111111111 1111111111 1111111111
                             0000000000 0000000000 0000000000 0000000000 0000000000 0000000000 0000000000 0000000000 0000000000 0000000000 0000000000
                             4444444444 4444444444 4444444444 4444444444 4444444445 5555555555 5555555555 5555555555 5555555555 5555555555 5555555555
                             5555555556 6666666667 7777777778 8888888889 9999999990 0000000001 1111111112 2222222223 3333333334 4444444445 5555555556
                             1234567890 1234567890 1234567890 1234567890 1234567890 1234567890 1234567890 1234567890 1234567890 1234567890 1234567890
De_novo_assembly_KT445934    GATTCCCACT GTCCCTATCT ACTATCCAGC GAAACCACAG CCAAGGGAAC GGGCTTGGCG GAATCAGCGG GGAAAGAAGA CCCTGTTGAG CTTGACTCTA GTCTGGCGCT
Reference_assembly           GATTCCCACT GTCCCTACCT ACTCTCCAGC GAAACCACAG CCAAGGGAAC GGGCTTGGCG GAATCAGCGG GGAAAGAAGA CCCTGTTGAG CTTGACTCTA GTCTGGCGCT

                             1111111111 1111111111 1111111111 1111111111 1111111111 1111111111 1111111111 1111111111 1111111111 1111111111 1111111111
                             0000000000 0000000000 0000000000 0000000000 0000000000 0000000000 0000000000 0000000000 0000000000 0000000000 0000000000
                             5555555555 5555555555 5555555555 5555555556 6666666666 6666666666 6666666666 6666666666 6666666666 6666666666 6666666666
                             6666666667 7777777778 8888888889 9999999990 0000000001 1111111112 2222222223 3333333334 4444444445 5555555556 6666666667
                             1234567890 1234567890 1234567890 1234567890 1234567890 1234567890 1234567890 1234567890 1234567890 1234567890 1234567890
De_novo_assembly_KT445934    GTGAAGAGAC ATGAGAGGTG TAGAATAAGT GGGAGGCCCC GCGGTCGCGC GACCCGCGCC GCGGCCCGGC CGCCGGTGAA ATACCACTAC TCTGATCGTT TTTTCACTTA
Reference_assembly           GTGAAGAGAC ATGAGAGGTG TAGAATAAGT GGGAGGCCCC GCGGTCGCGC GACCCGCGCC GCGGCCCGGC CGCCGGTGAA ATACCACTAC TCTGATCGTT TTTTCACTTA

                             1111111111 1111111111 1111111111 1111111111 1111111111 1111111111 1111111111 1111111111 1111111111 1111111111 1111111111
                             0000000000 0000000000 0000000000 0000000000 0000000000 0000000000 0000000000 0000000000 0000000000 0000000000 0000000000
                             6666666666 6666666666 6666666667 7777777777 7777777777 7777777777 7777777777 7777777777 7777777777 7777777777 7777777777
                             7777777778 8888888889 9999999990 0000000001 1111111112 2222222223 3333333334 4444444445 5555555556 6666666667 7777777778
                             1234567890 1234567890 1234567890 1234567890 1234567890 1234567890 1234567890 1234567890 1234567890 1234567890 1234567890
De_novo_assembly_KT445934    CCCGGTGAGG CGGGGGGGCG AGCCCCGAGG GGCTCTCGCT TCTGGCGCCA AGCGCCCGGC GCGCGCCGGG CGCGACCCGC TCCGGGGACA GCGTCAGGTG GGGAGTTTGA
Reference_assembly           CCCGGTGAGG CGGGGGGGCG AGCCCCGAGG GGCTCTCGCT TCTGGCGCCA AGCGCCCGGC GCGCGCCGGG CGCGACCCGC TCCGGGGACA GCGTCAGGTG GGGAGTTTGA

                             1111111111 1111111111 1111111111 1111111111 1111111111 1111111111 1111111111 1111111111 1111111111 1111111111 1111111111
                             0000000000 0000000000 0000000000 0000000000 0000000000 0000000000 0000000000 0000000000 0000000000 0000000000 0000000000
                             7777777777 7777777778 8888888888 8888888888 8888888888 8888888888 8888888888 8888888888 8888888888 8888888888 8888888888
                             8888888889 9999999990 0000000001 1111111112 2222222223 3333333334 4444444445 5555555556 6666666667 7777777778 8888888889
                             1234567890 1234567890 1234567890 1234567890 1234567890 1234567890 1234567890 1234567890 1234567890 1234567890 1234567890
De_novo_assembly_KT445934    CTGGGGCGGT ACACCTGTCA AAGCGTAACG CAGGTGTCCT AAGGCGAGCT CAGGGAGGCC AGAAACCTCC CGTGGAGCAG AAGGGCAAAA GCTCGCTTGA TCTTGATTTT
Reference_assembly           CTGGGGCGGT ACACCTGTCA AAGCGTAACG CAGGTGTCCT AAGGCGAGCT CAGGGAGGCC AGAAACCTCC CGTGGAGCAG AAGGGCAAAA GCTCGCTTGA TCTTGATTTT
```

```
                            1111111111 1111111111 1111111111 1111111111 1111111111 1111111111 1111111111 1111111111 1111111111 1111111111 1111111111
                            0000000000 0000000000 0000000000 0000000000 0000000000 0000000000 0000000000 0000000000 0000000000 0000000000 0000000001
                            8888888889 9999999999 9999999999 9999999999 9999999999 9999999999 9999999999 9999999999 9999999999 9999999999 9999999990
                            9999999990 0000000001 1111111112 2222222223 3333333334 4444444445 5555555556 6666666667 7777777778 8888888889 9999999990
                            1234567890 1234567890 1234567890 1234567890 1234567890 1234567890 1234567890 1234567890 1234567890 1234567890 1234567890
De_novo_assembly_KT445934   CAGTACGAAT ACAGACCGTG AAAGCGGGGC CTCACGATCC TTCTGACTTT TTGGGTTTTA AGCAGGAGGT GTCAGAAAAG TTACCACAGG GATAACTGGC TTGTGGCGGC
Reference_assembly          CAGTACGAAT ACAGACCGTG AAAGCGGGGC CTCACGATCC TTCTGACTTT TTGGGTTTTA AGCAGGAGGT GTCAGAAAAG TTACCACAGG GATAACTGGC TTGTGGCGGC

                            1111111111 1111111111 1111111111 1111111111 1111111111 1111111111 1111111111 1111111111 1111111111 1111111111 1111111111
                            1111111111 1111111111 1111111111 1111111111 1111111111 1111111111 1111111111 1111111111 1111111111 1111111111 1111111111
                            0000000000 0000000000 0000000000 0000000000 0000000000 0000000000 0000000000 0000000000 0000000000 0000000001 1111111111
                            0000000001 1111111112 2222222223 3333333334 4444444445 5555555556 6666666667 7777777778 8888888889 9999999990 0000000001
                            1234567890 1234567890 1234567890 1234567890 1234567890 1234567890 1234567890 1234567890 1234567890 1234567890 1234567890
De_novo_assembly_KT445934   CAAGCGTTCA TAGCGACGTC GCTTTTTGAT CCTTCGATGT CGGCTCTTCC TATCATTGTG AAGCAGAATT CACCAAGCGT TGGATTGTTC ACCCACTAAT AGGGAACGTG
Reference_assembly          CAAGCGTTCA TAGCGACGTC GCTTTTTGAT CCTTCGATGT CGGCTCTTCC TATCATTGTG AAGCAGAATT CACCAAGCGT TGGATTGTTC ACCCACTAAT AGGGAACGTG

                            1111111111 1111111111 1111111111 1111111111 1111111111 1111111111 1111111111 1111111111 1111111111 1111111111 1111111111
                            1111111111 1111111111 1111111111 1111111111 1111111111 1111111111 1111111111 1111111111 1111111111 1111111111 1111111111
                            1111111111 1111111111 1111111111 1111111111 1111111111 1111111111 1111111111 1111111111 1111111112 2222222222 2222222222
                            1111111112 2222222223 3333333334 4444444445 5555555556 6666666667 7777777778 8888888889 9999999990 0000000001 1111111112
                            1234567890 1234567890 1234567890 1234567890 1234567890 1234567890 1234567890 1234567890 1234567890 1234567890 1234567890
De_novo_assembly_KT445934   AGCTGGGTTT AGACCGTCGT GAGACAGGTT AGTTTTACCC TACTGATGAT GTGTTGTTGC GCTAGTAATC CTGCTCAGTA CGAGAGGAAC CGCAGGTTCA GACATTTGGT
Reference_assembly          AGCTGGGTTT AGACCGTCGT GAGACAGGTT AGTTTTACCC TACTGATGAT GTGTTGTTGC GCTAGTAATC CTGCTCAGTA CGAGAGGAAC CGCAGGTTCA GACATTTGGT

                            1111111111 1111111111 1111111111 1111111111 1111111111 1111111111 1111111111 1111111111 1111111111 1111111111 1111111111
                            1111111111 1111111111 1111111111 1111111111 1111111111 1111111111 1111111111 1111111111 1111111111 1111111111 1111111111
                            2222222222 2222222222 2222222222 2222222222 2222222222 2222222222 2222222222 2222222223 3333333333 3333333333 3333333333
                            2222222223 3333333334 4444444445 5555555556 6666666667 7777777778 8888888889 9999999990 0000000001 1111111112 2222222223
                            1234567890 1234567890 1234567890 1234567890 1234567890 1234567890 1234567890 1234567890 1234567890 1234567890 1234567890
De_novo_assembly_KT445934   GTATGTGCTT GGCTGAGGAG CCACTGGAGC GAGGCTACCA TCTGTGGGAT TATGACTGAA CGCCTCTAAG TCAGAATCCC CCCTAAACGT AGCGATACCG CAGCGCCGAG
Reference_assembly          GTATGTGCTT GGCTGAGGAG CCACTGGAGC GAGGCTACCA TCTGTGGGAT TATGACTGAA CGCCTCTAAG TCAGAATCCC CCCTAAACGT AGCGATACCG CAGCGCCGAG

                            1111111111 1111111111 1111111111 1111111111 1111111111 1111111111 1111111111 1111111111 1111111111 1111111111 1111111111
                            1111111111 1111111111 1111111111 1111111111 1111111111 1111111111 1111111111 1111111111 1111111111 1111111111 1111111111
                            3333333333 3333333333 3333333333 3333333333 3333333333 3333333333 3333333334 4444444444 4444444444 4444444444 4444444444
                            3333333334 4444444445 5555555556 6666666667 7777777778 8888888889 9999999990 0000000001 1111111112 2222222223 3333333334
                            1234567890 1234567890 1234567890 1234567890 1234567890 1234567890 1234567890 1234567890 1234567890 1234567890 1234567890
De_novo_assembly_KT445934   GCGCCTCGGT GGGCTCGCGA TAGCCGGCCG CCGCCCCCCT CGGGCGGGCG GTCGGTGCGG AGCGCCGCTC GTGGTCGGGA CCGGAGCGCG GACAGATGTG GCGCCGCCTC
Reference_assembly          GCGCCTCGGT GGGCTCGCGA TAGCCGGCCG CCGCCCCCCT CGGGCGGGCG GTCGGTGCGG AGCGCCGCTC GTGGTCGGGA CCGGAGCGCG GACAGATGTG GCGCCGCCTC

                            1111111111 1111111111 1111111111 1111111111 1111111111 1111111111 1111111111 1111111111 1111111111 1111111111 1111111111
                            1111111111 1111111111 1111111111 1111111111 1111111111 1111111111 1111111111 1111111111 1111111111 1111111111 1111111111
                            4444444444 4444444444 4444444444 4444444444 4444444444 4444444445 5555555555 5555555555 5555555555 5555555555 5555555555
                            4444444445 5555555556 6666666667 7777777778 8888888889 9999999990 0000000001 1111111112 2222222223 3333333334 4444444445
                            1234567890 1234567890 1234567890 1234567890 1234567890 1234567890 1234567890 1234567890 1234567890 1234567890 1234567890
De_novo_assembly_KT445934   TCCCCCGCCG CGTACCGCAT GTTCGTGGGG AACCCGGTGC TAAATCATTC GTAGACGACC TGATTCTGGG TCGGGGTTTC GTACGTAGCA GAGCAGCTCC CTCGCTGCGA
Reference_assembly          TCCCCCGCCG CGTACCGCAT GTTCGTGGGG AACCCGGTGC TAAATCATTC GTAGACGACC TGATTCTGGG TCGGGGTTTC GTACGTAGCA GAGCAGCTCC CTCGCTGCGA
```

```
                              1111111111 1111111111 1111111111 1111111111 1111111111 1111111111 1111111111 1111111111 1111111111 1111111111 1111111111
                              1111111111 1111111111 1111111111 1111111111 1111111111 1111111111 1111111111 1111111111 1111111111 1111111111 1111111111
                              5555555555 5555555555 5555555555 5555555555 5555555556 6666666666 6666666666 6666666666 6666666666 6666666666 6666666666
                              5555555556 6666666667 7777777778 8888888889 9999999990 0000000001 1111111112 2222222223 3333333334 4444444445 5555555556
                              1234567890 1234567890 1234567890 1234567890 1234567890 1234567890 1234567890 1234567890 1234567890 1234567890 1234567890
De_novo_assembly_KT445934     TCTATTGAGA GTCAGCCCTC GACACAAGCT TTTGTCGGAG CGCGGAGCGC GCGCGCGCGC GCGCGTGGCG GCGCCCCGGC GCGGGGCCGG GTCCGGCGGG CCAGTCGGTC
Reference_assembly            TCTATTGAGA GTCAGCCCTC GACACAAGCT TTTGTCGGAG CGCGGAGCGC GCGCGCGCGC GCGCGTGGCG GCGCCCCGGC GCGGGGCCGG GTCCGGCGGG CCAGTCGGTC

                              1111111111 1111111111 1111111111 1111111111 1111111111 1111111111 1111111111 1111111111 1111111111 1111111111 1111111111
                              1111111111 1111111111 1111111111 1111111111 1111111111 1111111111 1111111111 1111111111 1111111111 1111111111 1111111111
                              6666666666 6666666666 6666666666 6666666667 7777777777 7777777777 7777777777 7777777777 7777777777 7777777777 7777777777
                              6666666667 7777777778 8888888889 9999999990 0000000001 1111111112 2222222223 3333333334 4444444445 5555555556 6666666667
                              1234567890 1234567890 1234567890 1234567890 1234567890 1234567890 1234567890 1234567890 1234567890 1234567890 1234567890
De_novo_assembly_KT445934     GGCTCCCCGC GCCGCTCCGT TTGTTCCTGG GTTCGTTCGT TCGTTCGTTC CTTCCTTCCC CGGCCCCGCG CCGGCGCCGG CGCGGGGTTG GAAAGAGGGG GAGAGGGGCG
Reference_assembly            GGCTCCCCGC GCCGCTCCGT TTGTTCCTGG GTTCGTTCGT TCGTTCGTTC CTTCCTTCCC CGGCCCCGCG CCGGCGCCGG CGCGGGGTTG GAAAGAGGGG GAGAGGGGCG

                              1111111111 1111111111 1111111111 1111111111 1111111111 1111111111 1111111111 1111111111 1111111111 1111111111 1111111111
                              1111111111 1111111111 1111111111 1111111111 1111111111 1111111111 1111111111 1111111111 1111111111 1111111111 1111111111
                              7777777777 7777777777 7777777778 8888888888 8888888888 8888888888 8888888888 8888888888 8888888888 8888888888 8888888888
                              7777777778 8888888889 9999999990 0000000001 1111111112 2222222223 3333333334 4444444445 5555555556 6666666667 7777777778
                              1234567890 1234567890 1234567890 1234567890 1234567890 1234567890 1234567890 1234567890 1234567890 1234567890 1234567890
De_novo_assembly_KT445934     GGGGGCGCGG CCGGCCCCCT TCCCCGTTTC CGTCCCCGCG GCGCGTGCCG TGGGACGGGC TCCCTCCGTT TTACCCGAGC CCGGGGGTTG ACCTGGCGGC CGGGCGGCCG
Reference_assembly            GGGGGCGCGG CCGGCCCCCT TCCCCGTTTC CGTCCCCGCG GCGCGTGCCG TGGGACGGGC TCCCTCCGTT TTACCCGAGC CCGGGGGTTG ACCTGGCGGC CGGGC-----

                              1111111111 1111111111 1111111111 1111111111 1111111111 1111111111 1111111111 1111111111 1111111111 1111111111 1111111111
                              1111111111 1111111111 1111111111 1111111111 1111111111 1111111111 1111111111 1111111111 1111111111 1111111111 1111111111
                              8888888888 8888888889 9999999999 9999999999 9999999999 9999999999 9999999999 9999999999 9999999999 9999999999 9999999999
                              8888888889 9999999990 0000000001 1111111112 2222222223 3333333334 4444444445 5555555556 6666666667 7777777778 8888888889
                              1234567890 1234567890 1234567890 1234567890 1234567890 1234567890 1234567890 1234567890 1234567890 1234567890 1234567890
De_novo_assembly_KT445934     GGCTAGGGGG CGCTCCGCGT CCCCCTTCGG GGGGTTGACC TGTCGGGCGT TTTTTTTTTA TTTTTTTCTC CCTAGGCGGG TCCGGGGGTA GCCCTGTCGG CCGCCCGGCC
Reference_assembly            ---TAGGGGG CGCTCCGCGT CCCCCTTCGG GGGGTTGACC TGTCGGGCGT TTTTTTTTTA TTTTTTTCTC CCTAGGCGGG TCCGGGGGTA GACCTGTCGG CCGGCCGGCC

                              1111111111 1111111111 1111111111 1111111111 1111111111 1111111111 1111111111 1
                              1111111112 2222222222 2222222222 2222222222 2222222222 2222222222 2222222222 2
                              9999999990 0000000000 0000000000 0000000000 0000000000 0000000000 0000000000 0
                              9999999990 0000000001 1111111112 2222222223 3333333334 4444444445 5555555556 6
                              1234567890 1234567890 1234567890 1234567890 1234567890 1234567890 1234567890 1
De_novo_assembly_KT445934     CGGCCCAGCA CGCCCCCCC- GCCGGCAAGT GGCTGCGGTG CCGAGGTGGC GGGTAGACCT GGCGGCCGGC A
Reference_assembly            CGGCCCAGCA CGCCCCCCCC GTCGGTAAGT GGCTGCGGTG CCGAGGTGGC GGGTAGACCT GGCGGCCGGC T
```
